# Single-position ligand modifications tune CB_2_R activity by targeting the toggle switch

**DOI:** 10.1039/d6sc00062b

**Published:** 2026-03-18

**Authors:** Rudolf L. Z. Ganzoni, Miroslav Kosar, Yongqi Han, Rosa Maria Vitale, Pietro Amodeo, Xiaoting Li, Zhonghua Zha, Kacper J. Patej, Bilal Kicin, Taddäus E. N. Strunden, Lisa Reichert, Uxía Gómez-Bouzó, Themiya P. Perera, Kenneth Atz, Wolfgang Guba, Christian Bartelmus, Raphael Bigler, Paolo Tosatti, Stephan Bachmann, Tian Hua, David A. Sykes, Dmitry B. Veprintsev, Uwe Grether, Erick M. Carreira

**Affiliations:** a Laboratorium für Organische Chemie, Eidgenössische Technische Hochschule Zürich Vladimir-Prelog-Weg 3 8093 Zürich Switzerland erickm.carreira@org.chem.ethz.ch; b Faculty of Medicine & Health Sciences, University of Nottingham, Centre of Membrane Proteins and Receptors (COMPARE) Nottingham NG7 2UH UK david.sykes@nottingham.ac.uk dmitry.veprintsev@nottingham.ac.uk; c Institute of Biomolecular Chemistry, National Research Council Via Campi Flegrei 34 80078 Pozzuoli Italy; d iHuman Institute, ShanghaiTech University Shanghai 201210 China; e Roche Pharma Research & Early Development, Roche Innovation Center Basel, F. Hoffmann-La Roche Ltd 4070 Basel Switzerland uwe.grether@roche.com; f Department of Process Chemistry and Catalysis, F. Hoffmann-La Roche Ltd Basel 4070 Switzerland

## Abstract

Cannabinoid receptor type 2 (CB_2_R) is a prominent class A G protein-coupled receptor (GPCR) and is a therapeutic target of interest for inflammatory diseases, pain management, and neurodegenerative disorders. We report the development of ligands based on HU-308 that share a single central scaffold but bear diverse sidechains, enabling controlled modulation of GPCR activation. Structural modifications at a single position of the parent ligand allow modulation of the single-residue toggle switch of CB_2_R, Trp258^6.48^, and thereby control over receptor activity. A continuum of functional outcomes is achieved through interaction of the ligands with the CB_2_R toggle switch, leading to full agonism, partial agonism, neutral antagonism, or partial inverse agonism. Several low-efficacy ligands display protean behavior across assays, underscoring context-dependent modulation of CB_2_R and its importance in profiling such ligands. A notable compound within this series is CF_3_-substituted (*S*)-1, which displays distinct CB_2_R affinity, potency, and a biased CB_2_R signaling profile. We provide a rationale based on molecular dynamics simulations for the unique pharmacological profile observed and suggest that stabilization of an active receptor conformation occurs by close-contact interaction of (*S*)-1 with the CB_2_R toggle switch. Our findings demonstrate that strategic structural modifications of class A GPCR ligands may, by targeting a receptor's toggle switch, shift ligands to different positions along the efficacy spectrum, independent of their parent scaffold's original functional profile.

## Abstract 2

This study, exemplified at CB_2_R, showcases how strategic reutilization of a single ligand scaffold fine-tunes activity of a class A GPCR by virtue of targeting its toggle switch.

## Graphical abstract

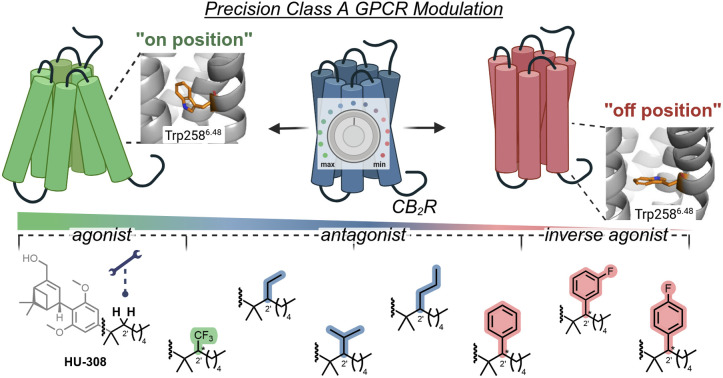

## Introduction

G protein-coupled receptors (GPCRs) represent the largest family of membrane proteins in the human genome and are targets for approximately one-third of all approved drugs by the Food and Drug Administration (FDA),^1,2^ with class A GPCR subpopulations constituting approximately 86% of GPCR drug targets.^3^ Class A GPCRs have attracted substantial interest and investment,^3–5^ with the market for small-molecule modulators projected to grow significantly in the coming years.^6^ However, the challenge associated with discovering new ligands for a target is significant,^7^ and developing suitable leads into a drug is underscored by its prohibitive cost.^8–10^ The process of lead identification for GPCR targets is particularly difficult because of a variety of different binding and activation modes and the associated, diverse functional outcomes at the receptor.^7,11^

The cannabinoid receptors are prominent examples of class A GPCRs which hold promise for treatment of various ailments^12^ but have fallen short of that promise so far.^13^ In recent years, cannabinoid receptor 2 (CB_2_R) especially has garnered significant interest as a therapeutic target. As CB_2_R is mainly expressed in immune cells in the periphery,^14,15^ selective pharmacological intervention at CB_2_R offers potential for the treatment of neuroinflammation,^16^ autoimmune disorders,^17,18^ chronic pain,^19,20^ as well as cancer^21,22^ while circumventing adverse effects associated with cannabinoid receptor 1 (CB_1_R) modulation.^23^ The promising therapeutic potential of selective CB_2_R modulation, however, has not yet successfully translated to the clinic,^24,25^ with recent discontinuation of development programs aimed at treatment of cancer^26^ and irritable bowel syndrome.^27^ Most recently, development of ajulemic acid (Lenabasum), a CB_1_R/CB_2_R dual agonist for the treatment of cystic fibrosis and dermatomyositis, was discontinued as of 2023.^28^

The apparent difficulty of advancing CB_2_R selective compounds in the clinic underpins the need for a better understanding of structural determinants of receptor activation. Indeed, there is a paucity of fine-tunable ligands to activate or deactivate CB_2_R over the entirety of its efficacy spectrum: agonists ↔ partial agonists ↔ neutral antagonists ↔ inverse agonists.

Despite the abundance of known CB_2_R agonists, CB_2_R-selective partial agonists are scarce.^30–32^ This scarcity reflects the challenges in designing ligands that finely tune receptor activity without inducing maximal activation or complete inhibition. Receptor density and coupling efficiency are two key factors known to influence the apparent degree of agonism.^33,34^ Consequently, the pharmacological behavior of partial agonists is highly context dependent: the same ligand may function as an agonist, neutral antagonist, or inverse agonist, depending on basal activity and cellular environment in native as well as overexpressing systems. In this respect, context dependence of ligand efficacy has been referred to as protean agonism. For example, as described in pioneering studies, in systems with high constitutive CB_2_R activity, low-efficacy partial agonists (AM-1241 and L768242) may function as neutral antagonists or inverse agonists, whereas in systems with reduced basal activity, the same ligands can activate the receptor.^35^

In the clinic, it has been suggested that partial agonists may offer better control of context-dependent modulation, thereby achieving desired therapeutic effects.^4,36,37^ For example, Δ^9^-tetrahydrocannabinol (THC), a CB_1_R/CB_2_R partial agonist, elicits milder psychotropic adverse effects compared to synthetic compounds known to be full agonists of CB_1_R (*e.g.* CP-55940, HU-210).^38,39^ In the context of CB_2_R, the partial CB_2_R-selective agonist LEI-102 was shown to effectively protect against cisplatin induced kidney injury in mice.^40^ This finding is particularly relevant given that CB_2_R activation by full agonists has negative implications for immunosuppression, leading to infection and sepsis.^41,42^ Analogously, limited downmodulation by partial inverse agonists may offer opportunities in ameliorating conditions which result from increased basal tone, without completely abolishing signaling and thereby disrupting the receptor's role in maintaining homeostasis.^43,44^

**Fig. 1:**
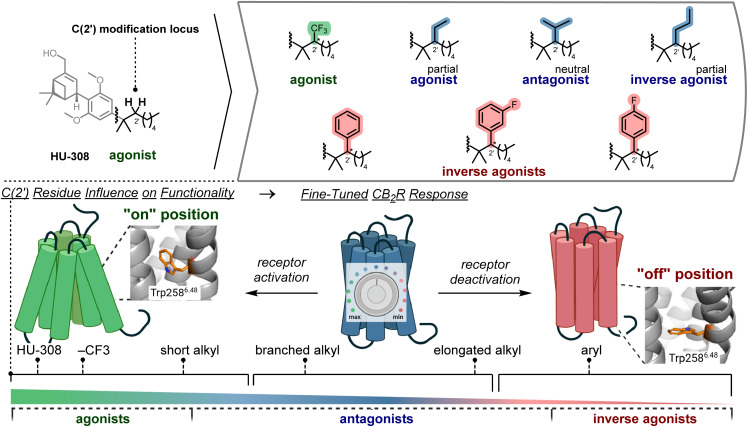
Ligand design based on the HU-308 scaffold and modification of the C(2′) position. Fine-tuned actuation of the CB_2_R Trp258^6.48^ single-residue toggle switch leads to a continuum of functional outcomes at the receptor.^29^

Previous work from our group focused on fluorescent imaging of CB_2_R.^45^ We detailed a C(2′) phenyl-substituted HU-308 (ref. 46) derivative that displayed inverse agonist activity. Notably, the ligand retains its inverse agonist functional profile, affinity, and selectivity independent of its conjugation to a range of fluorescent reporters (*e.g.* DY-480XL, fluorescein, Alexa488). We wondered whether variation of substituents at C(2′) (Fig. 1) would offer a venue for a novel approach to ligand design that leverages reutilization of known scaffolds for the purpose of covering a broader and fine-tuned range of accessible functionality.

Herein, we provide a basis for strategic structural alterations at an established class A GPCR ligand to venture beyond switch-like, binary functional responses. Inspired by recent reports showcasing intermediate receptor states of class A GPCRs that populate an active-to-inactive continuum,^47^ we sought to elicit gradual functional outcomes at CB_2_R. It has recently been appreciated that the activity of many class A GPCRs hinges on conformational flexibility of residues within the receptor that function as molecular toggle switches.^2,48^ Targeting the precise modulation of a receptor's toggle switch therefore presents an opportunity to access its efficacy spectrum and elicit fine-tuned functional responses. In this respect, we employ a single, exemplary molecular scaffold to elicit functional outcomes spanning the efficacy spectrum of the receptor by virtue of targeted modulation of Tryptophan 258^6.48^ (Trp258^6.48^)_,_ the single-residue toggle switch of CB_2_R.^40,49,50^

Based on the canonical CB_2_R ligand HU-308 (ref. 46) and intuitive design principles guided by docking studies, we synthesized and studied a collection of CB_2_R ligands. The ligands we describe display high CB_2_R affinity and excellent selectivity for CB_2_R over CB_1_R. Profiling in a panel of *in vitro* (G protein recruitment) as well as *in cellulo* (cyclic adenosine monophosphate (cAMP) production, β-arrestin recruitment, G protein recruitment) functional assays revealed a spectrum of accessible ligand efficacy compared to its parent scaffold spanning agonist, partial agonist, neutral antagonist, and inverse agonist functionality. The assays were benchmarked with canonical balanced agonist CP-55,940 (ref. 51–53) and CB_2_R-selective inverse agonist SR-144,528.^51^ An exemplary compound within the series synthesized, C(2′) CF_3_-derivative (*S*)-1, a high-affinity partial agonist, was investigated by molecular dynamics (MD) simulations. This revealed a close contact interaction between (*S*)-1 and Trp258^6.48^ in the CB_2_R/ligand complex.

## Results and discussion

### *In silico* ligand design

**Fig. 2:**
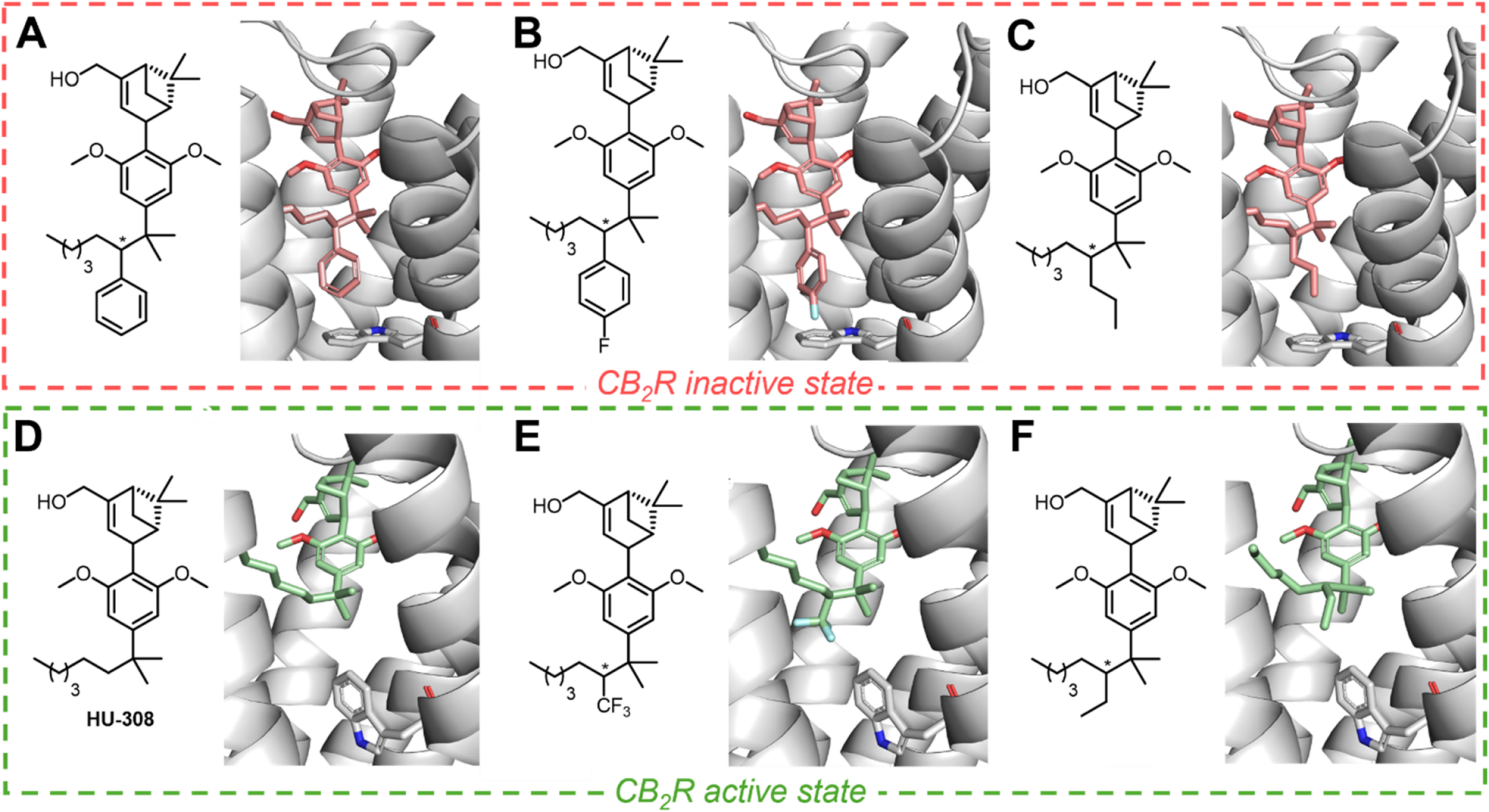
Docking studies of a representative panel of candidate molecules (A) phenyl (light red), (B) *p*-F-phenyl (light red), and (C) *n*-propyl (light red)-substituted HU-308 derivatives docked into the inactive state of the receptor (PDB: 5ZTY), displaying no major steric clashes. (D) HU-308 parent compound (mint green), (E) CF_3_-substituted derivative (mint green), and (F) ethyl (mint green)-substituted HU-308 derivatives only fit the active state of CB_2_R (PDB: 8GUS). Protein backbone atoms are represented as ribbons colored in light gray. Nitrogen and oxygen are colored blue and red, respectively.

Our studies were based on the hypothesis that varying substituent size at the C(2′) position of the HU-308-derived ligands would affect the functional outcome at the receptor. At the onset of our investigation, we conducted docking studies to rapidly evaluate the influence of various substitutions at the homobenzylic C(2′) position of the HU-308 sidechain. This approach was aimed at qualitatively assessing the potential to modulate the Trp258^6.48^ toggle switch by employing a simple-to-use model based on substituent size and to provide an initial gauge of the hypothesis' viability (Fig. 2, see SI). We focused on HU-308 derivatives where substitution at the C(2′) position would incrementally alter the steric bulk in the binding pocket and thereby affect the fit of these molecules in either the active or inactive state of CB_2_R. We sought to explore the extent to which larger or modified aryl groups could be accommodated in the cleft hosting the CB_2_R toggle switch and to assess their potential to elicit a more efficacious inverse agonist response. Accordingly, we expected smaller substituents to exhibit partial agonist or neutral antagonist activity, as they could be accommodated by both the active and inactive receptor states.

The docking studies indicated that even moderately more spatially demanding groups than phenyl at C(2′), such as benzyl, would exceed the limits of the binding pocket (in both the active and the inactive receptor state) and likely diminish affinity for the receptor. Consequently, phenyl and *para*/*meta*-fluorine-substituted phenyl groups were selected as possessing maximal substituent volume likely to maintain a favorable fit, as judged by computational modeling (Fig. 2A and B). Our qualitative docking studies suggested that aryl modifications, as well as C(2′) *n*-propyl substitution (Fig. 2C) could be accommodated only within the inactive state of CB_2_R, whereas smaller aliphatic side chains were predicted to fit both the active and inactive receptor conformation (Fig. 2D–F). Based on this initial viability check, we proceeded to compile a focused panel of potential ligands.

To the best of our knowledge, the only other reported modification at the analogous C(2′) position involves the study of Δ^8^-THC-derivatives with CH_3_/H substitution at C(2′) and H/H substitution at C(1′), which is associated with CB_2_R selectivity over CB_1_R.^54^ Beyond that, there is a lack of studies elucidating how small aliphatic groups at this position influence receptor affinity or what drives the compounds' functional profiles. This further motivated our investigation into rational structural modification beyond methyl at the C(2′) position of HU-308, shifting ligand functionality away from full agonism. This emerged as a working model to tune ligand functionality across a broad efficacy spectrum.

### Synthesis

**Scheme 1:**
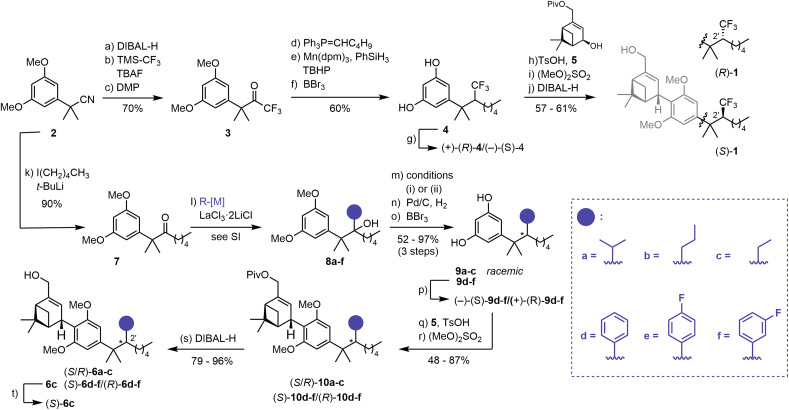
Synthesis of HU-308-derived C(2′)-substituted CB_2_R ligands. (a) DIBAL-H, CH_2_Cl_2_, –78 °C to rt, 96%; (b) TMS-CF_3_, TBAF, −78 °C to rt, 94%; (c) DMP, CH_2_Cl_2_, 0 °C to rt, 78%; (d) Ph_3_P(C_5_H_11_)Br, *n*-BuLi, −78 °C to rt, 73%; (e) Mn(dpm)_3_, PhSiH_3_, TBHP, *i*-PrOH, 83%; (f) BBr_3_, CH_2_Cl_2_, 0 °C to rt, 99%; (g) semi-preparative SFC, see SI for details; (h) TsOH·H_2_O, 5, CH_2_Cl_2_, 74–78%; (i) (MeO)_2_SO_2_, K_2_CO_3_, acetone, 94–97%; (j) DIBAL-H, CH_2_Cl_2_, 0 °C, 81–82%; (k) I(CH_2_)_4_CH_3_, *t*-BuLi, Et_2_O, pentane, −78 °C to rt, 90%; (l) R-[M], LaCl_3_·LiCl, THF, 0 °C, 71–99%, see SI for details; (m) (i) KHMDS, CS_2_, MeI, THF, −78 °C to rt, then 40 °C, or (ii) SOCl_2_, DMAP, pyridine, 0 °C to rt, see SI for details; (n) Pd/C, H_2_ (7–75 atm); (o) BBr_3_, CH_2_Cl_2_, 0 °C, 52–97% over 3 steps; (p) semi-preparative SFC, see SI for details; (q) TsOH·H_2_O, 5, CH_2_Cl_2_; (r) (MeO)_2_SO_2_, K_2_CO_3_, acetone, 48–87% over 2 steps; (s) DIBAL-H, CH_2_Cl_2_, 0 °C, 79–96%; (t) semi-preparative SFC, see SI for details.

A modular synthetic approach was employed to access a panel of C(2′) substituted HU-308 derivatives. In the synthesis of fluorescent inverse agonist probes based on HU-308 amide derivatives, a dialkyl ketone readily underwent addition by phenyl lithium to give a tertiary alcohol that was subjected to dehydration and reduction.^45^ Implementation of this strategy to access CF_3_-substituted derivatives (*R*)-1 and (*S*)-1 failed and required a new approach. The synthesis of (*R*)-1/(*S*)-1 commenced with reduction of nitrile 2 to the corresponding aldehyde, which was then treated with TMS-CF_3_ (ref. 55) and tetrabutylammonium fluoride (TBAF) (Scheme 1).^56^ Subsequent oxidation with Dess-Martin Periodinane (DMP) afforded CF_3_-ketone 3 (70%, 3 steps). Introduction of the *n*-pentyl fragment proved problematic, as ketone reduction by the corresponding organometal reagent^57,58^ occurred preferentially over 1,2-addition. However, treatment of 3 with Ph_3_P=CHC_4_H_9_ effected efficient olefination. Attempted olefin reduction under classical hydrogenation conditions (Pd/C, H_2_) proved challenging, as no conversion was observed at 1 atm, and reaction at elevated pressure (7 atm) was accompanied by week-long reaction times and overreduction of the resorcinol to the corresponding cyclohexane. Consequently, we employed a modified hydrogen atom transfer (HAT) procedure^59^ to effect smooth reduction of the unusual CF_3_-olefin. Subsequent demethylation afforded resorcinol 4 in 60% yield (3 steps). Separation by semi-preparative supercritical fluid chromatography (SFC) furnished the two enantiomers (+)-(*R*)-4 and (−)-(*S*)-4 in excellent enantiomeric excess (ee) (>99% and 98%, respectively). The absolute configuration of the C(2′) stereocenter was unambiguously established by X-ray crystallographic studies of a derivative (see SI and Schemes S1 and S2). The parallel synthesis of (*R*)-1/(*S*)-1 was completed by Friedel–Crafts allylation of (*R*)-4 and (*S*)-4 with allylic alcohol 5 in the presence of TsOH·H_2_O. This was followed by methylation and pivalate removal, affording the CF_3_-bearing HU-308-derivatives in 57 to 61% yield over three steps.

The synthesis of a collection of HU-308 derived ligands, (*S*/*R*)-6a–c, (*S*)-6c, (*S*)-6d–f, and (*R*)-6d–f was also initiated from common nitrile precursor 2 (Scheme 1). Treatment of 2 with *n*-pentyl lithium, followed by imine hydrolysis, afforded dimethyl benzyl ketone 7. A variety of substituents were introduced by organometal 1,2-addition mediated by LaCl_3_·2LiCl,^60^ essential for the high efficiency of this step, providing tertiary alcohols 8a–f. Subsequent elimination of the alcohols generated the respective olefins. Cleavage of the methyl ethers and hydrogenation mediated by Pd/C afforded resorcinols 9a–f in good yields (52 to 97%, 3 steps). We carried out extensive efforts to develop an enantioselective hydrogenation protocol. However, the highest ee achieved was 80% (see SI, Table S1). Separation of 9a–f by semi-preparative SFC proved effective for isolation of enantiomers (−)- and (+)-9d–f (98–>99% ee). The assignment of the absolute configuration of (−)-(*S*)-9d and (+)-(*R*)-9d was carried out by independent synthesis from a previously characterized compound^45^ (see SI). For (−)-(*S*)-9e/(+)-(*R*)-9e and (−)-(*S*)-9f/(+)-(*R*)-9f the configuration is assigned by analogy based on SFC retention times and optical rotation (see SI).

Attempts at separation of the enantiomers for 9a–c were not met with success. More than 20 chiral SFC columns were screened, and separation was attempted at multiple synthetic stages. However, efforts to resolve the enantiomers were mostly unsuccessful. To nevertheless assess the influence of these intermediate-size C(2′) sidechains, 9a–c were then employed as racemates, affording inseparable C(2′) diastereomer mixtures in the subsequent steps. With resorcinols (−)-(*S*)-9d–f, (+)-(*R*)-9d–f and 9a–c in hand, we set our sights on the completion of the synthesis in analogy to (*R*)-1/(*S*)-1: Friedel–Crafts allylation with 5 proceeded smoothly and furnished, after methylation, pivalates (*S*)-10d–f, (*R*)-10d–f, and (*S*/*R*)-10a–c in good yields (48 to 87%, 2 steps). In a last step, reductive cleavage of the pivalate afforded HU-308 analogs (*S*)-6d–f, (*R*)-6d–f, and (*S*/*R*)-6a–c (79 to 96%). At this stage, one diastereomer of 6c could be separated by semi-preparative SFC, affording (+)-6c (99% ee), whose configuration was tentatively assigned as (*S*) by analogy to (*R*)-1/(*S*)-1 (see SI).

### Affinity profiling: competition binding assay

To validate that the HU-308 derivatives synthesized interact with CB_2_R and retain the high selectivity of the parent compound, we determined their binding affinities at CB_2_R and CB_1_R. To that end, we employed our validated time-resolved Förster resonance energy transfer (TR-FRET) binding assay.^45,61–64^ HEK293 membrane preparations of SNAP-Lumi4-Tb labelled hCB_2_R and hCB_1_R were incubated with a validated fluorescent probe (see SI) and the novel ligands (Table 1).^64^ In accord with our previous findings with phenyl-modified HU-308 derivatives, compounds (*S*)-6d and (*R*)-6d showed high affinity for CB_2_R (p*K*_i_ = 7.15 and 8.88, respectively), as well as excellent selectivity for CB_2_R over CB_1_R in the case of (*R*)-6d (243-fold). Similarly, CF_3_-bearing derivatives (*R*)-1 and (*S*)-1 displayed selectivity for CB_2_R (18- and 3615-fold, respectively) with (*S*)-1 standing out with its high affinity (p*K*_i_ = 9.57). In contrast, (*R*)-1 showed attenuated CB_2_R affinity (p*K*_i_ = 6.56). The trend of high binding affinities continued for derivatives bearing aliphatic sidechains, as apparent for (*S*/*R*)-6a (p*K*_i_ = 9.87) as well as (*S*)-6c and (*S*/*R*)-6c (p*K*_i_ = 8.96 and 9.76, respectively). (*S*)-6c Displayed six-fold reduced affinity compared to (*S*/*R*)-6c. (*S*/*R*)-6b Displayed good binding and selectivity at CB_2_R (p*K*_i_ = 8.62, *K*_i_ ratio = 1996).

**Table 1:** TR-FRET-based profiling of binding affinity^a^

Ligand	C(2′)-R	p*K*_i_ [nM]		
		CB_2_R	CB_1_R	*K*_i_ ratio (CB_1_R/CB_2_R)
(*R*)-1	CF_3_	6.56 (±0.11)	5.37 (±0.20)	18
(*S*)-1	CF_3_	9.57 (±0.42)	5.84 (±0.30)	3615
(*S*/*R*)-6a^b^	*i*-Pr	9.87 (±0.21)	5.50 (±0.12)	20 912
(*S*/*R*)-6b^b^	*n*-Pr	8.62 (±0.18)	5.50 (±0.10)	1996
(*S*)-6c^c^	Et	8.96 (±0.16)	5.93 (±0.20)	1189
(*S*/*R*)-6c^b^	Et	9.76(±0.16)	5.75 (±0.37)	19 098
(*S*)-6d	Ph	7.15 (±0.07)	6.30 (±0.26)	10
(*R*)-6d	Ph	8.88 (±0.03)	6.66 (±0.26)	243
(*S*)-6e^c^	*p*-F-Ph	6.13 (±0.07)	6.36 (±0.27)	1
(*R*)-6e^c^	*p*-F-Ph	7.41 (±0.33)	6.27 (±0.26)	11
(*S*)-6f^c^	*m*-F-Ph	7.01(±0.18)	5.85 (±0.20)	15
(*R*)-6f^c^	*m*-F-Ph	9.12 (±0.20)	6.26 (±0.05)	600
HU-308^d^	—	7.57^d^	5.41^d^	145^d^
CP-55,940^d^	—	7.51^d^	8.32^d^	6^d^
SR-144,528^d^	—	8.55^d^	6.43^d^	131^d^

a Competition binding data (p*K*_i_) were determined in a TR-FRET assay at 37 °C with membrane preparations from either hCB_2_R-HEK293 or hCB_1_R-HEK293 cells. Data shown as a mean, *N* = 3–4 (±SEM).

b C(2′) Diastereomeric mixture.

c C(2′) Configuration assigned by analogy.

d Previously reported in the same assay.^64^

Docking studies had predicted that fluorinated phenyl side chains would be accommodated within the CB_2_R binding pocket, and indeed we observed binding of these compounds at CB_2_R, albeit with a slight drop in affinity. Fluoroarene derivatives (*S*)-6e/(*R*)-6e (p*K*_i_ = 6.13 and 7.41, respectively) and (*S*)-6f/(*R*)-6f (p*K*_i_ = 7.01 and 9.12, respectively) exhibited reduced binding affinities compared to their non-fluorinated counterparts (*S*)-6d and (*R*)-6d. (*R*)-6f retained good selectivity (600-fold) and comparable affinity (p*K*_i_ = 9.12) to (*R*)-6d for CB_2_R. These findings suggest that the *para* fluorine substituents on the C(2′) phenyl sidechains introduce steric bulk that is not optimally accommodated by the pocket that harbors the single-residue toggle switch Trp258^6.48^. Such substitution may lead to unfavorable interactions within the binding pocket of the receptor. A decrease in selectivity of these fluorinated HU-308 congeners for CB_2_R over CB_1_R may stem from their decreased affinity for CB_2_R, especially in the case of *para*-fluorination.

With binding profiles established, we next proceeded to systematically characterize the pharmacological responses elicited across established CB_2_R signaling pathways. Since the parent compound HU-308 behaves as a potent full agonist at CB_2_R,^46,51^ we were particularly interested to examine whether we could observe fine-tuned functional outcomes differing from full agonism.

### Functional profiling: cAMP production, G protein recruitment, and β-arrestin recruitment

A canonical signaling pathway of CB_2_R involves its interaction with Gα_i/o_ proteins, which transiently inhibit adenylyl cyclase, leading to a reduction in cellular cAMP levels and the suppression of protein kinase A activity.^65^ Accordingly, we focused our attention on elucidating the functional profiles of the HU-308 derivatives in a homogeneous time-resolved fluorescence (HTRF) cAMP assay and a bioluminescence resonance energy transfer (BRET) G_i_-CASE assay (Table 2).^66^

**Table 2:** Functional characterization in CB_2_R HTRF cAMP^a^ and CB_2_R G_i_-CASE assays^b^

Ligand	C(2′)-R	HTRF cAMP assay		G_i_-CASE assay	
		pEC_50_	*E*_max_/%	pEC_50_	*E*_max_/%
(*R*)-1	CF_3_	6.59 (±0.12)	90 (±6)	6.48 (±0.13)	62 (±10)
(*S*)-1	CF_3_	7.97 (±0.09)	95 (±4)	9.42 (±0.19)	78 (±1)
(*S*/*R*)-6a^e^	*i*-Pr	—	—	8.31 (±0.20)	40 (±4)
(*S*/*R*)-6b^e^	*n*-Pr	7.01 (±0.23)^c^	−33 (±5) [−45 (±7)]^d^	7.15 (±0.03)	24 (±6)
(*S*)-6c^f^	Et	7.36 (±0.15)	69 (±6)	8.2 (±0.19)	80 (±17)
(*S*/*R*)-6c^e^	Et	6.87 (±0.11)	81 (±5)	8.72 (±0.25)	67 (±5)
(*S*)-6d	Ph	6.32 (±0.25)^c^	−25 (±4) [−34 (±5)]^d^	8.29 (±0.22)^c^	−28 (±8) [−41 (±12)]^d^
(*R*)-6d	Ph	6.61 (±0.17) c	−46 (±5) [−63 (±7)]^d^	8.47 (±0.12)^c^	−43 (±9) [−64 (±13)]^d^
(*S*)-6e^f^	*p*-F-Ph	5.66 (±0.31)^c^	−18 (±5) [−25 (±7)]^d^	6.82 (±0.29)^c^	−29 (±7) [−42 (±10)]^d^
(*R*)-6e^f^	*p*-F-Ph	—	—	7.22 (±0.10)	25 (±3)
(*S*)-6f^f^	*m*-F-Ph	5.97 (±0.41)^c^	−30 (±9) [−41 (±12)]^d^	7.37 (±0.17)^c^	−24 (±8) [−36 (±12)]^d^
(*R*)-6f^f^	*m*-F-Ph	6.76 (±0.13)^c^	−43 (±4) [−59 (±5)]^d^	8.62 (±0.29)^c^	−40 (±10) [−59 (±15)]^d^
HU-308	—	7.76 (±0.11)	89 (±5)	7.40 (±0.03)	91 (±4)
SR-144,528^d^	—	5.56 (±0.13)^c^	−73 (±8) [−100 (±11)]^d^	7.97 (±0.09)^c^	−68 (±4) [100 (±6)]^d^
CP-55,940	—	9.11 (±0.07)	100 (±4)	8.52 (±0.03)	100 (±2)

a Potency (pEC_50_) and efficacy (*E*_max_) data were obtained in a cAMP HTRF assay using hCB_2_R–CHO cells.

b Potency (pEC_50_) and *E*_max_ data were obtained in a G_i_-CASE BRET-based assay using membrane preparations from hCB_2_R-HEK293 T-REx cells. Data were normalized to agonist CP-55,940 response (100%) and basal level (0%).

c Entries reflect pIC_50_.

d Data were additionally normalized to inverse agonist SR-144,528 (ref. 51) (100%) and basal level (0%) and are shown in brackets. Data shown as a mean (±SEM), *N* = 3–4.

e C(2′) Diastereomeric mixture.

f C(2′) Configuration assigned by analogy.

In the cAMP assay, the derivatives synthesized spanned a range of functionality from agonists (Table 2, *e.g.* (*S*)-1) to inverse agonist (Table 2, *e.g.* (*R*)-6d), with efficacies (*E*_max_) ranging from 95 to −46%. Interestingly, both (*R*)-1 and (*S*)-1 displayed agonistic behavior, triggering decrease in cAMP levels, albeit with considerably differing potencies (pEC_50_ = 6.59 and 7.97, respectively).

Ethyl-substituted HU-308 derivatives (*S*)-6c and (*S*/*R*)-6c displayed partial agonist properties (*E*_max_ = 69% and 81%, respectively) in the assay together with high potencies (pEC_50_ = 7.36 and 6.87, respectively). HU-308 derivative (*S*/*R*)-6a with a branched *i*-Pr group displayed neutral antagonist behavior while elongated *n*-Pr-substituted derivative (*S*/*R*)-6b behaved as an inverse agonist (*E*_max_ = −33%). Substituents at the C(2′) position apparently mediate a fine-tuned grading of functionality from agonism to inverse agonism. These observations for the first time demonstrate the effect of relatively minor increases in sidechain size at C(2′) on the ligands' functional profiles.

#### Stringent size requirements to modulate CB_2_R toggle switch

(*R*)-6d Proved a more potent inverse agonist than (*S*)-6d (pEC_50_ = 6.61 and 6.32, respectively). Our initial hypothesis was that increasing size of the C(2′) phenyl substituent by means of fluorine substitution would increase inverse agonist activity, however, fluorinated derivatives did not show increased inverse agonist properties. *Meta*-F-substituted derivatives (*S*)-6f and (*R*)-6f (*E*_max_ = −30 and −43%, respectively) displayed inverse agonist character comparable to (*S*)-6d and (*R*)-6d, while *para*-F-substituted derivatives showed attenuated inverse agonist efficacy: (*S*)-6e (*E*_max_ = −18%) displayed reduced efficacy compared to (*S*)-6d, and (*R*)-6e displayed neutral antagonist character in the cAMP assay. We suspect that *para*-F substitution resulted in a pronounced reduction in inverse agonist efficacy due to increased steric bulk of the side chain, highlighting the stringent structural requirements for modulating the CB_2_R Trp258^6.48^ single-residue toggle switch. These results suggest that increases in the size of the C(2′) substituent may enhance inverse agonist efficacy only up to a threshold. Sidechains exceeding the pocket's capacity lead to a decrease in the ligands' inverse agonist efficacy and potency.

#### Evaluation across multiple assays hint at protean agonism

Next, we investigated the compounds' effects on G protein recruitment by CB_2_R. To that end, we employed our validated BRET G_i_-CASE assay.^66^ Membrane preparations from hCB_2_R-HEK293 T-REx cells that contain a genetically encoded fluorescence NanoLuciferase donor and Venus acceptor protein conjugated to the G_α_ and G_γ_ subunits, respectively, were co-incubated with the compounds and the *Δ* BRET signal was detected (Table 2). CB_2_R activation by agonists leads to dissociation of the G_α_ and G_βγ_ subunits, which in turn results in a reduction of BRET signal. Analogously, inverse agonists lead to an increase in BRET signal by G protein accumulation.

**Fig. 3:**
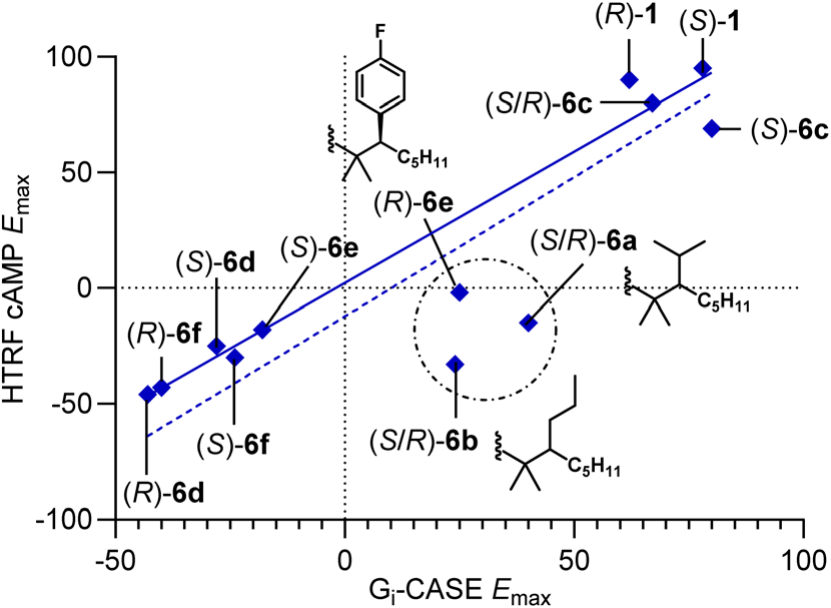
Efficacy correlation between HTRF cAMP and G_i_-CASE assays. Compounds enclosed within the circle (*S*/*R*)-6a, (*S*/*R*)-6b, and (*R*)-6e show protean behavior across the two assays. Solid blue line = correlation excluding compounds within the circle (*R* = 0.98). Dotted line = correlation including all compounds (*R* = 0.88). Data were normalized to agonist CP-55,940 response (100%) and basal level (0%).

The G_i_-CASE data follow the trends observed with functional data obtained from the HTRF cAMP assay (Table 2, solid blue line Fig. 3). Agonists (*R*)-1 and (*S*)-1 displayed comparable pEC_50_ values to the cAMP assay (6.48 and 9.42, respectively) with attenuated efficacy (*E*_max_ = 62 and 78%, respectively) compared to the HU-308 parent ligand (*E*_max_ = 91%). Interestingly, (*S*)-1 displayed 100-fold increased potency relative to HU-308 (pEC_50_ = 7.40) in the assay. (*S*/*R*)-6a (*E*_max_ = 40%), (*S*)-6c (*E*_max_ = 80%), and (*S*/*R*)-6c (*E*_max_ = 67%) retained their partial agonist character in the G_i_-CASE assay compared to the cAMP assay. However, in contrast to the cAMP functional readout as inverse agonist, (*S*/*R*)-6b displayed weak partial agonist properties (pEC_50_ = 7.15, *E*_max_ = 24%) in the G_i_-CASE assay. We hypothesize that the ligands that show low efficacy may display protean behavior in terms of functionality across assays. Differences in the basal activity across assays or tissues may move partial agonists into the neutral antagonist/inverse agonist realm (and *vice versa*) – a known phenomenon for class A GPCRs.^33,68,69^ Indeed, other protean ligands at CB_2_R have been reported in the literature.^35,70–72^ Additionally, specific conditions of the assays may exhibit a bias in terms of accessible receptor conformations because of the truncated or modified cannabinoid receptors employed.^49^

Ligands bearing aromatic sidechains (*S*)-6d (*E*_max_ = −28%), (*R*)-6d (*E*_max_ = −43%), (*S*)-6e (*E*_max_ = −29%), (*S*)-6f (*E*_max_ = −24%), and (*R*)-6f (*E*_max_ = −40%) corroborated the functional profiles established by the HTRF cAMP assay. Only the results for *para*-F substituted (*R*)-6e deviated from the trend (pEC_50_ = 7.22, *E*_max_ = 25%), suggesting that (*R*)-6e may act as protean agonists in a context dependent fashion. The compound's reduced affinity could be a driving factor behind its observed functionality.

#### Complementary assays reveal context-dependent function

We then turned our attention to further profile the identified agonists' and partial agonists' effects on β-arrestin recruitment, a further canonical and well-studied signaling pathway of CB_2_R that is G protein independent.^73,74^ To this end, we employed a BRET-based β-arrestin 2 (Arr2) recruitment assay. In parallel, we performed a mini-G_i_ recruitment assay to provide an additional, complementary G protein-based assay that is completely unamplified, measuring receptor–G_i_ association rather than heterotrimer dissociation. As the parent scaffold of our ligands, HU-308, is known to exhibit β-arrestin-biased signaling,^51^ in contrast to the functionally unbiased profile of the balanced agonist CP-55,940, we were also interested to investigate if the bias of the parent scaffold would translate to its C(2′)-modified derivatives. Biased signaling in class A GPCRs^75^ has been suggested to offer potential therapeutic advantages over balanced agonists due to their more focused signaling profile and has garnered significant attention in regard to CB_2_R^51,76–78^ as well as CB_1_R for agonists with attenuated side effects.^79^

Notably, compounds (*S*/*R*)-6a and (*S*/*R*)-6b did not induce measurable recruitment of effector proteins in the β-arrestin as well as in the mini-G_i_ assay (Table 3). This lack of recruitment is consistent with their low efficacy and assay-dependent behavior observed in cAMP and G_i_-CASE assays. (*S*)-6c and (*S*/*R*)-6c, more efficacious agonists in the previous assays, showed moderate potency and efficacy in the β-arrestin assay (pEC_50_ = 7.48, *E*_max_ = 41% and pEC_50_ = 7.91, *E*_max_ = 43%, respectively) as well as in the mini-G_i_ assay (pEC_50_ = 6.97, *E*_max_ = 51% and pEC_50_ = 7.40, *E*_max_ = 50%, respectively). Interestingly, while (*S*)-1 displayed robust G_i_ and β-arrestin recruitment (pEC_50_ = 7.44, *E*_max_ = 86% and pEC_50_ = 7.12, *E*_max_ = 72%, respectively), (*R*)-1 failed to elicit a response in both assays (mini-G_i_ and β-arrestin) and did not measurably recruit either effector protein. An observable trend for this set of essays is that only very efficacious agonists elicited a response, suggesting that β-arrestin and mini-G_i_ assays are well-suited for comparative assessment for the profiling of efficacious ligands because of their unamplified and associative nature. Conversely, more sensitive assays such as G_i_-CASE (lower receptor reserve)^66^ and HTRF cAMP (downstream amplification) may be better suited for the identification of protean ligands.

**Table 3:** Functional characterization: mini-G_i_ Protein^a^ and β-Arrestin recruitment^b^ at CB_2_R^c^

Ligand	C(2′)-R	Mini-G_i_ assay		β-arrestin assay	
		pEC_50_	*E*_max_/%	pEC_50_	*E*_max_/%
(*R*)-1	CF_3_	—	—	—	—
(*S*)-1	CF_3_	7.12 (±0.17)	72 (±5)	7.44 (±0.10)	86 (±4)
(*S*/*R*)-6a^d^	*i*-Pr	—	—	—	—
(*S*/*R*)-6b^d^	*n*-Pr	—	—	—	—
(*S*)-6c^e^	Et	6.97 (±0.07)	51 (±5)	7.48 (±0.20)	41 (±5)
(*S*/*R*)-6c^d^	Et	7.40 (±0.17)	50 (±2)	7.91 (±0.08)	43 (±3)
HU-308	—	6.93 (±0.11)	79 (±9)	7.07 (±0.10)	94 (±13)
CP-55,940	—	8.37 (±0.06)	100 (±1)	8.14 (±0.16)	100 (±2)

a Potency (pEC_50_) and *E*_max_ data were obtained in a mini-G_i_ BRET-based assay using hCB_2_R-HEK293 T-REx cells.

b Potency (pEC_50_) and *E*_max_ data were obtained in a β-arrestin BRET-based assay using hCB_2_R-HEK293 T-REx cells.

c Functional data were normalized to agonist CP-55,940 response (100%) and basal level (0%). Data shown as a mean (±SEM), *N* = 6–8.

d C(2′) Diastereomeric mixture.

e C(2′) Configuration assigned by analogy.

Data from the compounds that were able to elicit a response in the mini-G_i_ and β-arrestin assays was furthermore used to assess whether the ligands showed biased signaling behavior according to the method developed by Bouvier and co-workers.^80^ Indeed, (*S*)-1, (*S*)-6c, (*S*/*R*)-6c displayed bias towards β-arrestin signaling, mirroring the behavior of HU-308 (see SI Table S1 and Fig. S3). This suggests that C(2′) substitution has a limited impact on biased signaling and that the scaffold's preference for β-arrestin recruitment is governed by other structural determinants which are beyond the scope of this work.

#### Strategic modifications to HU-308 scaffold fine-tune signaling profile

**Fig. 4:**
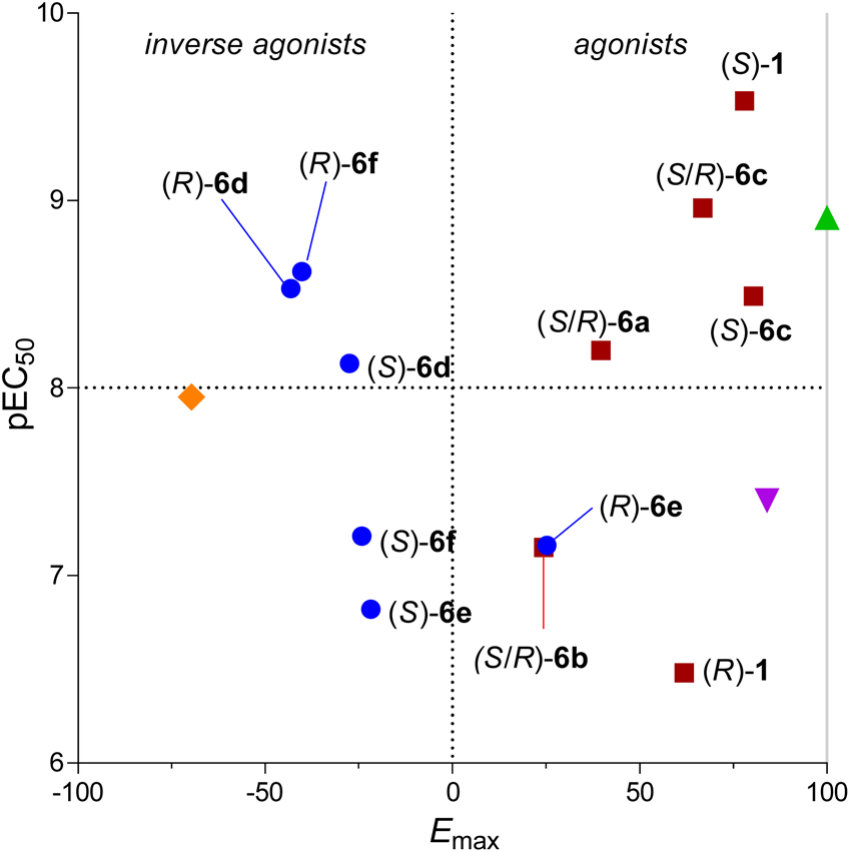
Correlation diagram of ligand potency *vs.* efficacy as assessed in the G_i_-CASE assay. (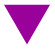 = HU-308; 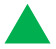 = CP,55 940; 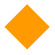 = SR-144,528 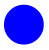 = aryl substituent; 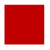 = alkyl substituent). Data were normalized to agonist CP-55,940 response (100%) and basal level (0%).

In general, our results across different functional assays correlate. Exceptions include ligands showing protean behavior in terms of functionality: partial agonists that can also display neutral antagonist or moderate inverse agonist character (*i.e.* compounds (*R*)-1, (*R*)-6e, (*S*/*R*)-6a, and (*S*/*R*)-6b). We attribute this phenomenon to different basal activities and protein constructs across assays. As exemplified in the G_i_-CASE assay, a generally identifiable trend for our HU-308 derivatives is that aryl groups at the C(2′) position induce a functionality switch from agonism to inverse agonism (Fig. 4). Based on our working model, we suggest that inactivation of the receptor occurs by steric interaction-driven reorientation of the toggle switch into the conformation that leads to stabilization of the inactive state of CB_2_R. Fluorinated phenyls are tolerated, albeit at the cost of potency. The only exception to this trend of decreased potency is *meta*-F-substituted compound (*R*)-6f, which showed slightly increased potency (pEC_50_ = 8.62 *vs.* pEC_50_ = 8.47) compared to benchmark compound (*R*)-6d (Table 2). Alkyl substitution at the C(2′) position generally leads to partial agonists, with (*S*/*R*)-6a and (*S*/*R*)-6b displaying antagonist character in the mini-G_i_ and β-arrestin assays, presumably due to their low efficacy and the non-amplified character of these assays. Compound (*S*/*R*)-6a displayed inverse agonist properties in the cAMP assay, illustrating how low potency ligands have the potential to elicit a range of context-dependent functional outcomes.

Of the compounds with C(2′) aliphatic substitution, (*S*)-1 is an especially interesting case, as the introduction of a CF_3_ residue leads to a significant increase in potency as well as efficacy compared to the parent compound HU-308 in the G_i_-CASE assay. In contrast, diastereomer (*R*)-1 displays a stark drop in affinity and potency at CB_2_R and did not recruit effector proteins in the mini-G_i_ and β-arrestin assays.

Of note, although G_i_-CASE, HTRF cAMP, and mini-G_i_ assays all probe G protein-coupled signaling, they can yield markedly different outcomes, particularly for ligands that do not act as full agonists. Evaluating ligands across multiple functional assays is important, particularly for G protein-coupled pathways of signaling, since results can vary depending on where in the signaling cascade the measurement is taken.^67^ Context-dependency influenced by basal signaling tone as well as assay conditions and sensitivity can lead to functional profiles that may not be recapitulated across other assays targeting the same pathway. For instance, (*R*)-1 did not significantly recruit mini-G_i_ but did act as a partial agonist in the G_i_-CASE (*E*_max_ = 62%) and HTRF cAMP (*E*_max_ = 90%) assays. Our results are consistent with previous observations that show how narrow functional profiling may lead to premature identification of seemingly clear-cut functional profiles, highlighting the importance of early, thorough profiling across multiple and comparable assay platforms.^51^

### Molecular dynamics investigation of (*R*)-1 and (*S*)-1

**Fig. 5:**
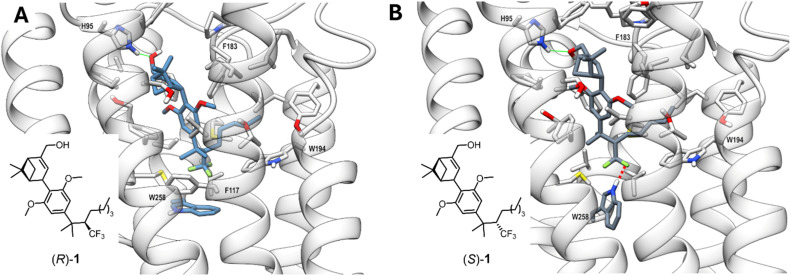
Representative frames from MD simulations of CB_2_R in complex with CF_3_-bearing epimers (*R*)-1 and (*S*)-1. (*R*)-1 is colored in steel blue (A), (*S*)-1 in slate gray (B). A stick representation is used for heavy atoms of the ligand and for protein sidechains within 5 Å of the ligand. Protein backbone atoms are represented as ribbons colored in light gray. Hydrogen, nitrogen, oxygen, and sulfur atoms are colored white, blue, red, and yellow, respectively. A green wire representation is adopted for peripheral H-bonds and a red dash representation for the key interaction in (B).

Given the distinct pharmacological profile of compounds (*R*)-1 and (*S*)-1, as discussed above, molecular dynamics (MD) simulations were employed. We selected the X-ray structure of CB_2_R in its inactive conformation (PDB: 5ZTY) as the starting point for the MD simulations in order to investigate ligand-induced transition toward the active state.^81,82^ Starting from the inactive state rather than the active state is an approach to explore potential differences in receptor activation triggered by the two compounds (*R*)-1 and (*S*)-1. Within the orthosteric binding site, both epimers are predicted to exhibit the previously reported, canonical HU-308 L-shaped conformation^40,49,50^ which is stable over the simulated time of 1 µs (Fig. 5 and see SI). The hydroxyl group on the pinene core of both compounds (Fig. 5A for (*R*)-1, Fig. 5B for (*S*)-1) is predicted to form a hydrogen-bond with His95^2.65^.

The structure of the CB_2_R/(*R*)-1 simulated complex reflects an inactive state of the receptor. Conversely, in the simulation, the CF_3_ group of (*S*)-1 engages the indole of Trp258^6.48^ in a close contact interaction that promotes a reorientation of the Trp258^6.48^ sidechain. This interaction is reflected by a shorter distance between the fluorine atoms of (*S*)-1 and the NE1 atom of Trp258^6.48^ in the CB_2_R/(*S*)-1 complex (≤3.5 Å in 50.07% of the MD frames) compared to the CB_2_R/(*R*)-1 complex over the simulation period (see SI Fig. S5). Furthermore, only in the CB_2_R/(*S*)-1 simulated complex is the ionic lock (not shown in Fig. 5) between Arg131^3.50^ and Asp240^6.30^ disrupted, indicating a conformational shift towards the activated form of CB_2_R (see SI Fig. S6).^40^ Finally, the difference in free energy of binding (ΔΔ*G*) between the two epimers, obtained by performing molecular mechanics/Poisson–Boltzmann (Generalized Born) surface area (MM/PB(GB)SA), shows that (*S*)-1 in complex is more stable by 3.55 kcal mol^−1^ (MM/GBSA) and 3.53 kcal mol^−1^ (MM/PBSA) than (*R*)-1 (see SI), providing insight for the marked difference in affinity between the two compounds. Overall, the MD simulations suggest that (*S*)-1 may demonstrate both higher receptor affinity and an agonist-like profile, providing a compelling rationale for its experimentally observed greater propensity for inducing CB_2_R activation and a greater affinity for the receptor compared to (*R*)-1.

## Conclusions

We have demonstrated that peripheral structural changes to an established agonist scaffold enable access to the entire efficacy spectrum at CB_2_R. In line with our initial hypothesis that varying substituent size at the C(2′) position of HU-308-derived ligands would modulate the CB_2_R toggle switch and thereby affect functional outcome, targeting the modulation of Trp258^6.48^ enables graded functional outcomes. Our design considerations for reutilizing the canonical CB_2_R ligand HU-308 were guided by a qualitative docking model that allowed for assessment of interaction with the toggle switch. Synthesis of a panel of designed ligands was achieved by a unified approach that required a modified HAT-reduction for access to CF_3_-bearing derivatives. The ligands' subsequent pharmacological profiling revealed a wide range of accessible functionalities, from full agonists to partial agonists and neutral antagonists to inverse agonists. Notably, (*S*)-1 showed high potency and β-arrestin bias, whereas its (*R*)-epimer exhibited reduced potency and failed to recruit effector proteins in both mini-G_i_ and β-arrestin assays. This exemplifies the marked impact that minor modifications, such as the introduction of a CF_3_ group, can have on receptor binding affinity and functional outcomes. These findings were further substantiated by molecular dynamics simulations, which provided mechanistic insights into the proposed effect of a close-contact interaction with the toggle switch residue and its influence on the increased affinity and activity of (*S*)-1 compared to (*R*)-1 and HU-308.

Our study, exemplified by precision pharmacology at CB_2_R, demonstrates how mechanistic understanding of class A GPCR activation facilitates rational design of fine-tunable ligands based on a single chemical scaffold and its binding modes in the orthosteric pocket. Derivatives with smaller aliphatic sidechains generally promote partial agonist activity that can switch into antagonist territory in a context-dependent manner. In contrast, sterically more demanding aromatic sidechains tend to induce inverse agonism or neutral antagonism, with those derivatives revealing a tight size tolerance for the cleft harboring the CB_2_R single-residue toggle switch.

More broadly, this work underscores the potential of scaffold reutilization on the basis of rational ligand design to achieve functional outcomes at designated positions on the efficacy spectrum. The approach presented herein is possible because of diversity-oriented synthesis strategies that enable modification at C(2′) of the HU-308 scaffold. Traditional focused library synthesis leads to variety of structures aimed typically at optimizing for one outcome. The present approach leverages targeted structural diversification to access diversity across a range of functionality.

Reutilizing known GPCR ligand scaffolds in this manner may be useful for early-stage discovery by leveraging pre-existing pharmacological and synthetic knowledge. Designing class A GPCR ligands that target toggle switches based on a single scaffold may provide a practical strategy to rapidly produce families of ligands with minimal modifications that display distinct functional profiles.

## Author contributions

E. M. C., D. B. V., D. A. S., U. G. conceived and oversaw the project. M. K. and R. L. Z. G. conceived the project, conceived chemical experiments and synthetic routes to tool compounds and derivatives, synthesized compounds, and analyzed data of chemical synthesis as well as biological data. U. G. further provided project administration and logistics. Y. H. and T. P. P. performed selected biological assays (G_i_-CASE). Y. H. performed data analysis and comparative data analysis on selected biological assays (G_i_-CASE, HTRF cAMP). R. M. V. and P. A. performed the molecular dynamics (MD) simulations. T. H. conceived oversaw the performed HTRF cAMP assays. X. L. and Z. Z. performed HTRF cAMP assays and provided data analysis thereof. D. A. S. conceived and performed biological assays and provided data analysis thereof as well as resulting calculations (Tb-FRET, G_i_-CASE, m-G_i_, β-arrestin). W. G. conceived and oversaw the docking studies. K. A. and W. G. performed the docking studies. R. B., P. T., S. B., and C. B. conducted experiments and analytics pertaining to the attempted asymmetric hydrogenation. K. J. P., B. K., T. E. N. S., L. R., and U. G.-B. performed the synthesis of selected compounds. R. L. Z. G., M. K., and E. M. C. wrote the manuscript. All authors participated in discussion and preparation of the manuscript.

## Conflicts of interest

The authors declare the following competing financial interest: M. K., B. K., W. G., U. G., and E. M. C. have filed a patent on CB_2_R selective modulators and fluorescent probes.

## Supplementary Material

SC-OLF-D6SC00062B-s001

SC-OLF-D6SC00062B-s002

## Data Availability

Experimental data associated with this work are available in the supplementary information (SI). Supplementary information is available. See DOI: https://doi.org/10.1039/d6sc00062b. CCDC 2450722 contains the supplementary crystallographic data for this paper.^83^
