# Supplementary material for: Single-position ligand modifications tune CB2R activity by targeting the toggle switch
Source: Chem Sci. 2026 Mar 18;17(18):8976–88. doi: 10.1039/d6sc00062b (PMC12998566; doi:10.1039/d6sc00062b)
Supplement: SC-017-D6SC00062B-s001 [file SC-017-D6SC00062B-s001.pdf]

Supplementary Information (SI) for Chemical Science.
This journal is © The Royal Society of Chemistry 2026

Single-Position Ligand Modifications Tune CB_2R Activity by Targeting the Toggle Switch

Rudolf L. Z. Ganzoni,[‡a] Miroslav Kosar,[‡a] Yongqi Han,[b] Rosa Maria Vitale,[c] Pietro Amodeo,[c] Xiaoting Li,[d] Zhonghua Zha,[d] Kacper J. Patej,[a] Bilal Kicin,[a] Taddäus E. N. Strunden,[a] Lisa Reichert,[a] Uxía Gómez-Bouzó,[a] Themiya P. Perera,[b] Kenneth Atz,[e] Wolfgang Guba,[e] Christian Bartelmus,[e] Raphael Bigler,[f] Paolo Tosatti,[f] Stephan Bachmann,[f] Tian Hua,[d] David A. Sykes,*[b] Dmitry B. Veprintsev,*[b] Uwe Grether,*[e] and Erick M. Carreira*[a]

[a]Laboratorium für Organische Chemie, Eidgenössische Technische Hochschule Zürich, Vladimir-Prelog-Weg 3, 8093 Zürich, Switzerland

[b]Faculty of Medicine & Health Sciences, University of Nottingham, Nottingham NG7 2UH, UK; Centre of Membrane Proteins and Receptors (COMPARE), University of Birmingham and University of Nottingham, Midlands, UK

[c]Institute of Biomolecular Chemistry, National Research Council, Via Campi Flegrei 34, 80078 Pozzuoli, Italy

[d]iHuman Institute, ShanghaiTech University, Shanghai 201210, China

[e]Roche Pharma Research & Early Development, Roche Innovation Center Basel, F. Hoffmann-La Roche Ltd., 4070 Basel, Switzerland

[f]Department of Process Chemistry and Catalysis, F. Hoffmann-La Roche Ltd, Basel 4070, Switzerland

*E-mail: erickm.carreira@org.chem.ethz.ch

*E-mail: uwe.grether@roche.com

*E-mail: dmitry.veprintsev@nottingham.ac.uk

*E-mail: david.sykes@nottingham.ac.uk

SUPPORTING INFORMATION

Table of Contents

TR-FRET CB_1 and CB_2R Binding Assays 4

Cell culture 4

Terbium labeling of SNAP-tagged CB_1 and CB_2R HEK293-TR cells 4

Fluorescent ligand binding assays 4

In vitro functional assays 5

Homogeneous Time Resolved Fluorescence (HTRF) assays 5

mG and β-arrestin recruitment assays 6

G_iCASE assay 6

Signal detection and data analysis 7

Supplementary figures for in vitro assays 9

Molecular Modeling and Molecular Dynamics Simulations 13

Molecular modeling 13

Molecular dynamics: computational methods 13

General Synthetic Methods 18

Procedure 18

Chemicals 18

Chromatography 18

Nuclear Magnetic Resonance Spectroscopy 18

High-Resolution Mass Spectrometry 18

Infrared Spectroscopy 18

Optical Rotation 18

X-ray Diffraction 19

Semi-preparative SFC 19

Synthesis 20

X-Ray Crystallographic Studies for the Stereochemical Assignment of (+)-(R)-4/(–)-(S)-4 26
Derivatization studies for the stereochemical assignment of (*S*)-9 and (*R*)-9........62
Asymmetric Hydrogenation Study 64
NMR Spectra 96
SFC Traces 205
Crystallographic Data 217
References 218

TR-FRET CB_1 and CB_2R Binding Assays

General

Human embryonic kidney 293TR cells were obtained from ThermoFisher Scientific. Unless otherwise stated, culture media, chemicals and ligands were purchased from Sigma-Aldrich. SR-144,528, and HU-308 were obtained from Tocris Bioscience (Bristol, United Kingdom). All ligands were dissolved in 100% DMSO (Sigma-Aldrich, 276855) and stored as aliquots at −20°C until required.

Cell culture

SNAP-tagged human CB_1 and CB_2 HEK293-TR cells were maintained in a humidified environment at 37 °C and 5% CO_2 in Dulbecco's modified Eagle's medium (DMEM) with 10% fetal bovine serum (FBS) containing blasticidin (5 μg/mL; Invitrogen) and Zeocin (20 μg/mL; Invitrogen). For receptor-inducible expression, cells were seeded into t175 cm^2 flasks, grown to 70% confluence and DMEM containing 1 μg/mL tetracycline added. 48h later cells were labelled with SNAP-Lumi4-Tb (CisBio) and membranes prepared as described in detail below.

Terbium labeling of SNAP-tagged CB_1 and CB_2R HEK293-TR cells

Cell culture medium was removed from the t175 cm^2 flasks containing confluent adherent CB_1 and CB_2R HEK293-TR cells. Cells were washed 1× in PBS (GIBCO Carlsbad, CA) followed by 1× Tag-lite labeling medium (LABMED, CisBio) to remove the excess cell culture media, then ten millilitre of LABMED containing 100 nM of SNAP-Lumi4-Tb was added to the flask and incubated for 1 h at 37 °C under 5% CO_2. Cells were washed 1× in PBS (GIBCO Carlsbad, CA) to remove the excess of SNAP-Lumi4-Tb then detached using 5 mL of GIBCO enzyme-free Hank's-based cell dissociation buffer (GIBCO, Carlsbad, CA) and collected in a vial containing 5 mL of DMEM (Sigma-Aldrich) supplemented with 10% fetal calf serum. Cells were pelleted by centrifugation (5 min at 1500 rpm) and the pellets were frozen to −80 °C. To prepare membranes, homogenization steps were conducted at 4 °C (to avoid receptor degradation). Following homogenization in ice cold buffer 10 mM HEPES containing 10 mM EDTA pH 7.4, an initial centrifugation was performed at 1200 g for 3 min to remove the nuclear fraction. Subsequent centrifugation steps were then performed as described by Borrega-Roman et al. [1]

Fluorescent ligand binding assays

All fluorescent ligand binding experiments were conducted in white 384-well Optiplate plates, in assay binding buffer, consisting of Hanks Balanced Salt Solution (HBSS), 5 mM HEPES, 0.5% BSA, 0.02% pluronic F-127 pH 7.4 and 100 μM GppNHp at room temperature.

Saturation binding assays to determine D77 equilibrium affinity

To determine equilibrium affinity values, at least six different concentrations of fluorescent ligand were used. Increasing concentrations of fluorescent ligand were incubated with human CB_2R HEK293-TR cell membranes (1 μg per well) in assay binding buffer (final assay volume, 50 μL). Nonspecific binding

was determined as the amount of HTRF signal detected in the presence of rimonabant (1 μM) or SR-144,528 (1 μM) in the case of human CB_1R and CB_2R, respectively, and was subtracted from total binding, to calculate specific binding, meaning that t = 0 was always equal to zero. The degree of fluorescent ligand bound to the receptor was assessed by TR-FRET detection. Saturation analysis was performed at equilibrium, by simultaneously fitting total and non-specific (NSB) binding data (Eq. 1) allowing the determination of fluorescent ligand binding affinity.

Competition binding TR-FRET assays

To determine the affinity of CB_1R and CB_2R-specific ligands, we used a competition binding assay. This approach involves the simultaneous addition of both fluorescent ligand and competitor to the CB_1 and CB_2R membrane preparations. Binding experiments were conducted in white 384-well Optiplate plates, in assay binding buffer, Hanks Balanced Salt Solution (HBSS), 5mM HEPES, 0.5% BSA, 0.02% pluronic F-127 pH 7.4, and 100 μM GppNHp. HEK293 cell membranes containing the human CB_1R and CB_2R (1 μg per well) were added to wells containing increasing concentrations of unlabelled ligands, and a fixed concentration of the fluorescent ligand D77 (700 nM), a concentration approximately 2-3x its K_d in 50 μL of assay buffer in a 384-well plate incubated at room temperature with orbital mixing. The degree of fluorescent ligand bound to the receptor was assessed at multiple time points by HTRF detection.

The equilibrium affinity of D77 plus those of unlabeled compounds were determined following an incubation period of 3h. Nonspecific binding was determined as the amount of HTRF signal detected in the presence of (rimonabant, 1 μM) or (SR-144,528, 1 μM) in the case of human CB_1R and CB_2R respectively, and was subtracted from total binding, to calculate specific binding. Data were normalized to the specific binding of the tracer D77 in the absence of test compounds, which was set as 100% (maximum binding). IC_{50} and K_i values obtained from competition binding assays were determined by fitting the data to a four-parameter logistic Hill equation (Eq. 2) and applying the Cheng and Prusoff correction.

In vitro functional assays

Homogeneous Time Resolved Fluorescence (HTRF) assays

The HTRF cAMP assay was performed as previously described.[2] Briefly, CHO cells stably overexpressing the CB2 receptor were cultured in Ham's F12 medium supplemented with 10% fetal bovine serum, 100 U/mL penicillin, 100 μg/mL streptomycin, and 400 μg/mL Geneticin Selective Antibiotic (G418 Sulfate). For compound testing, cells were harvested using Cell Dissociation Buffer and resuspended in F-12K medium at a density of 0.4×10^6 cells/mL. Subsequently, 5 μL of cell suspension (2000 cells/well) was dispensed into 384-well low-volume plates. The cells were then stimulated with test compounds diluted in Stimulation Buffer (2.5 μL/well) for 15 min at room

temperature, followed by the addition of 2.5 μL of 25 μM forskolin. After an additional 15 min incubation, the reactions were terminated by adding 5 μL/well of cAMP-d2 conjugate working solution, followed by 5 μL/well of anti-cAMP cryptate working solution. Following a 1 h incubation at room temperature, time-resolved fluorescence resonance energy transfer was measured using a PerkinElmer EnVision plate reader (excitation: 620 nm; emission: 665 nm). Data were analyzed by plotting the HTRF ratio ((Signal 665 nm/Signal 620 nm) × 104) against compound concentrations using Prism 8.1 (GraphPad Software).

Mini-G_i and β-arrestin recruitment assays

HEK293TR SNAP-CB_2R-nLuc cells expressing either a fluorescently labelled (Venus) mGi or β-arrestin protein were maintained in a humidified environment at 37 °C and 5% CO_2 in Dulbecco's modified Eagle's medium (DMEM) with 10% fetal bovine serum (FBS) containing blasticidin (5 μg/mL; Invitrogen), Zeocin (20 μg/mL; Invitrogen) and G418 (0.2mg/mL; Invitrogen) and used to assess either compound stimulated mGi or β-arrestin recruitment to the human CB_2R. Cultured cells were harvested upon reaching 70% confluency and plated at a seeding density of 50,000 cells per well. The cells were grown for 48h until they reached confluency and then stimulated with 1 μg/mL tetracycline for a further 48h to induce CB_2R expression. Cell culture media was then aspirated from the wells and the cells were washed with assay buffer, 100 μL/well HBSS containing 0.5% BSA and 5 mM HEPES. Following the wash, assay buffer containing 10 μM furimazine, 99 μL/well was added to the wells. The plate was then incubated at 37°C for 15 minutes to allow the nanoLuc substrate furimazine to enter the cells. Three BRET cycles of 1-minute intervals were performed to assess basal BRET levels, following which 1 μL of each compound, diluted in DMSO was added to the assay plate, which was read at 1-minute intervals for 30 minutes.

Compounds included serial dilutions of the synthetic CB_2R agonists CP-55,950, HU-308 and the novel HU-308-based agonists. DMSO (1% final) served as the vehicle control and 1 μM CP-55,950 was the positive control to which all responses were normalised.

G_iCASE assay

The development of the membrane-based Gi-CASE biosensor assay for profiling compounds at cannabinoid receptors has been described previously in the publication by Scott-Dennis et al.[3] The assay buffer used to profile compounds in the CB_2R G_i-CASE system consisted of HBSS containing 0.02% pluronic F127, 0.5% BSA and 5 mM HEPES. The reference compounds CP-55,950, HU-308, SR-144,528 and the test compounds were initially diluted in DMSO in a 96-well polypropylene plate. CB_2R membranes containing the Gi-CASE biosensor were thawed and added into assay buffer containing 50 μM furimazine. Membranes (49.5 μL) were added to a white 384 well Optiplate™ (PerkinElmer) at a final concentration of 5 μg/well. After 5 cycles (each of 1 min duration) 0.5 μL of reference and test compound serial dilutions were then added to the plate, the total assay volume was 50 μL. The assay plates were read using a BMG Pherastar FSX reader at 28 °C.[4] The duration of data collection on the

PHERAstar FSX using the BRET1 plus module (535-30LP/475-30BP), was 60 minutes at 1-minute intervals (60 cycles).

Signal detection and data analysis

For fluorescent ligand-binding assays signal detection was performed on a Pherastar FSX (BMG Labtech, Offenburg, Germany). The terbium donor was always excited with four laser flashes at a wavelength of 337 nm. TR-FRET signals were collected at both 520 nm (acceptor) and 620 nm (donor surrogate), when using the green NBD fluorescent ligand D77. TR-FRET ratios were obtained by dividing the acceptor signal by the donor signal and multiplying this value by 10,000.

Steady state CB_1R and CB_2R saturation binding data were analysed using GraphPad Prism 10.0 by non-linear regression according to a one-site equation by globally fitting total and NSB. Individual estimates for the fluorescent ligand dissociation constant (K_d) were calculated using the following equations where L is the fluorescent ligand concentration:

Total binding = Specific+NSB=(Bmax*[L])/(Kd+[L])+slope*[L]+Background

NSB=slope*[L]+Background (Eq. 1)

Fitting the total and NSB data sets globally (simultaneously), sharing the value of slope, provides one best-fit value for both the K_d and the B_{max}.

Steady state CB_1R and CB_2R competition displacement binding data were fitted to sigmoidal (variable slope) curves using a four-parameter logistic equation:

$$Y = Bottom + \frac{(Top - Bottom)}{1 + 10^{(logIC50 - X)*Hillslope}} \quad \text{(Eq. 2)}$$

Where, Bottom and Top are the plateaus of the agonist and inverse agonist curves. $LogIC_{50}$ is the concentration of competitor that gives a half-maximal effect, and the Hillslope is the unitless slope factor or Hillslope. IC_{50} values obtained from the inhibition curves were converted to K_i values using the method of Cheng and Prusoff.[5]

For *in-vitro* CB_2R functional assays signal detection was performed on a Pherastar FSX (BMG Labtech, Offenburg, Germany). The raw data from all experiments were converted to the ratio of BRET 1 (535-30LP/475-30BP). The data were then transferred to GraphPad PRISM 10. Characterisation of agonist CB_2R responses was achieved by selecting the concentration-response data from membranes at fixed time points which produced maximal responses to the ligands under test.

The selected data was normalized and expressed as the change in the BRET signal relative to the addition of vehicle. The graphs were plotted from the normalized data using sigmoidal dose-response (variable slope) or four-parameter logistic equation:

$$Y = Bottom + \frac{(Top - Bottom)}{1 + 10^{(logEC50 - X) - Hillslope}} \quad \text{(Eq 3)}$$

Where, Bottom and Top are the plateaus of the agonist and inverse agonist curves. $LogEC_{50}$ is the concentration of agonist/inverse agonist that gives a half-maximal effect and the HillSlope is the unitless slope factor or Hill slope.

To determine the relative effectiveness of the compounds to activate the different signaling pathways, the difference between the $\log(E_{max}/EC_{50})$ values was calculated. Analysis was performed as described previously[6, 7] to determine $\Delta\log(E_{max}/EC_{50})$ allowing the calculation of bias factors:

$$\Delta log\left(\frac{Emax}{EC50}\right) = \log(\frac{EmaxL1}{EC_{50}L1}) - \log(\frac{EmaxL2}{EC_{50}L2}) \qquad \text{(Eq.4)}$$

Where ligand 2 ($L2$) is the reference compound and ligand 1 ($L1$) is the test compound. $\Delta\log(E_{max}/EC_{50})$ values were determined using CP-55,940 as the reference agonist.

Using the estimates of agonist activity $\Delta\log(E_{max}/EC_{50})$ for test and reference agonist, pathway log bias was calculated as follows:

$$\Delta\Delta log\left(\frac{Emax}{EC50}\right) = \Delta log\left(\frac{Emax}{EC50}\right)P1 - \Delta log(\frac{Emax}{EC50})P2 \qquad \text{(Eq.5)}$$

Where pathway 1 ($P1$) is mG_s dependent recruitment and pathway 2 ($P2$) is β-arrestin2 recruitment. Bias factors were calculated by taking the antilog of the $\Delta\Delta\log(E_{max}/EC_{50})$.

Statistical analysis of the obtained data was performed using a two-tailed Welch t-test using Prism 10.4 (GraphPad Software). The data is tabulated below (Table S1).

Table S1. Results of statistical analysis of calculated bias factors

Ligand	*C(2')-R*	*Log Bias Factor ± SEM (BF)*	*N*	*p-value*
(*S*)-**1**	CF_3	0.62 ± 0.21 (4.2)	7	0.032*
(*S*)-**6c**	Et	0.65 ± 0.28 (4.5)	8	0.064+
(*S/R*)-**6c**	Et	0.68 ± 0.20(4.7)	6	0.020*
HU-308	–	0.45± 0.15 (2.8)	7	0.050*
CP55940	-	0 ± 0.14 (1)	8	n/a

*Significance at the 5% level; +significance at the 10% level.

Note: (*S*)-**6c** did not cross the significance threshold for significance at the 5% level, however, as p ((S)-**6c**) < 0.1 we attribute a trend towards bias for this compound.

Supplementary figures for in vitro assays

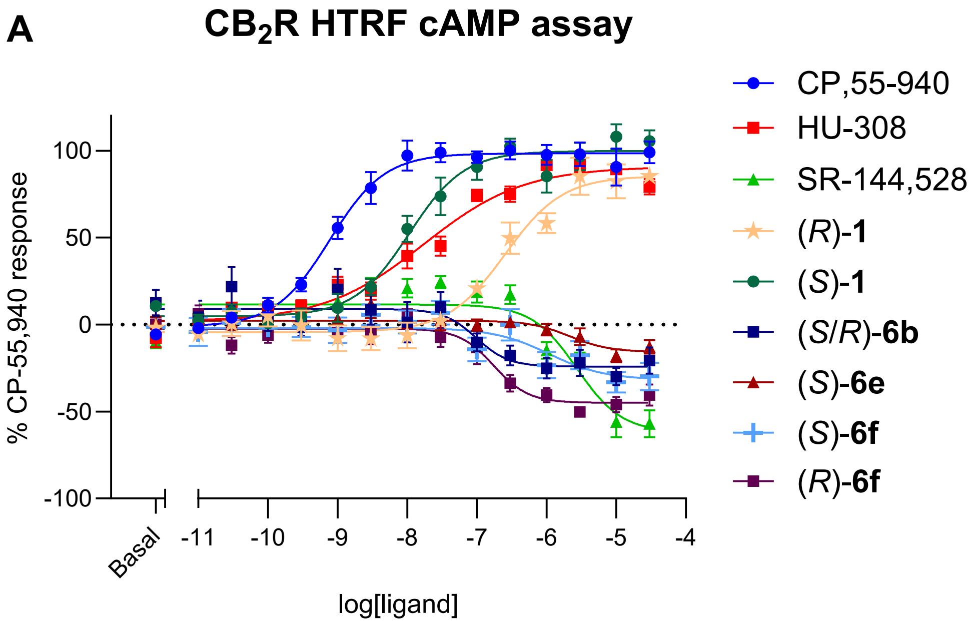

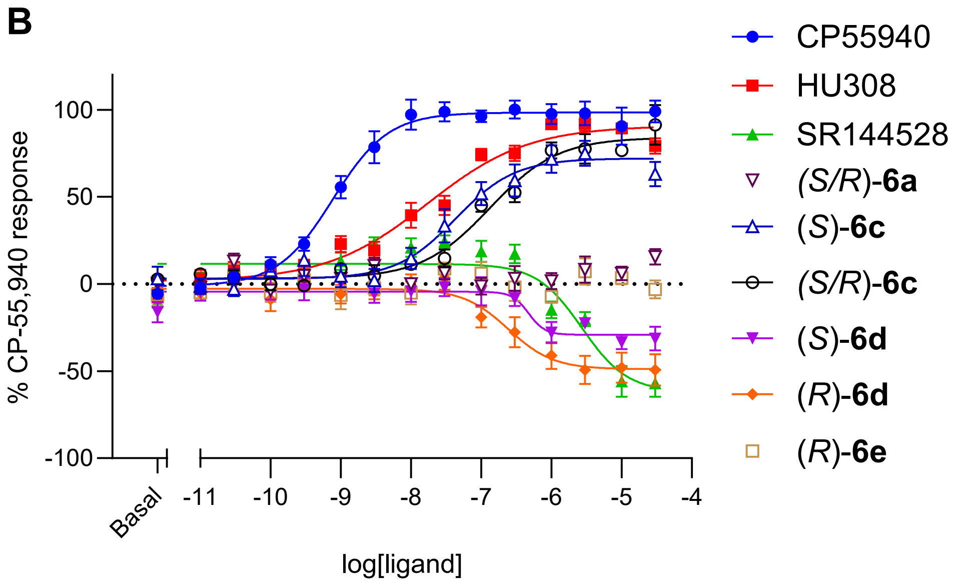

Figure S1. Visualized data from HTRF cAMP assay using hCB$_2$R-CHO cells. Comparable spectum of response to G_i-CASE assay: (A) corresponds to Figure S2 A and (B) corresponds to Figure S2 B. Data were normalized to agonist CP-55,940 response (100%) and basal level (0%). Data shown as a mean, $N = 3$–4 (±SD).

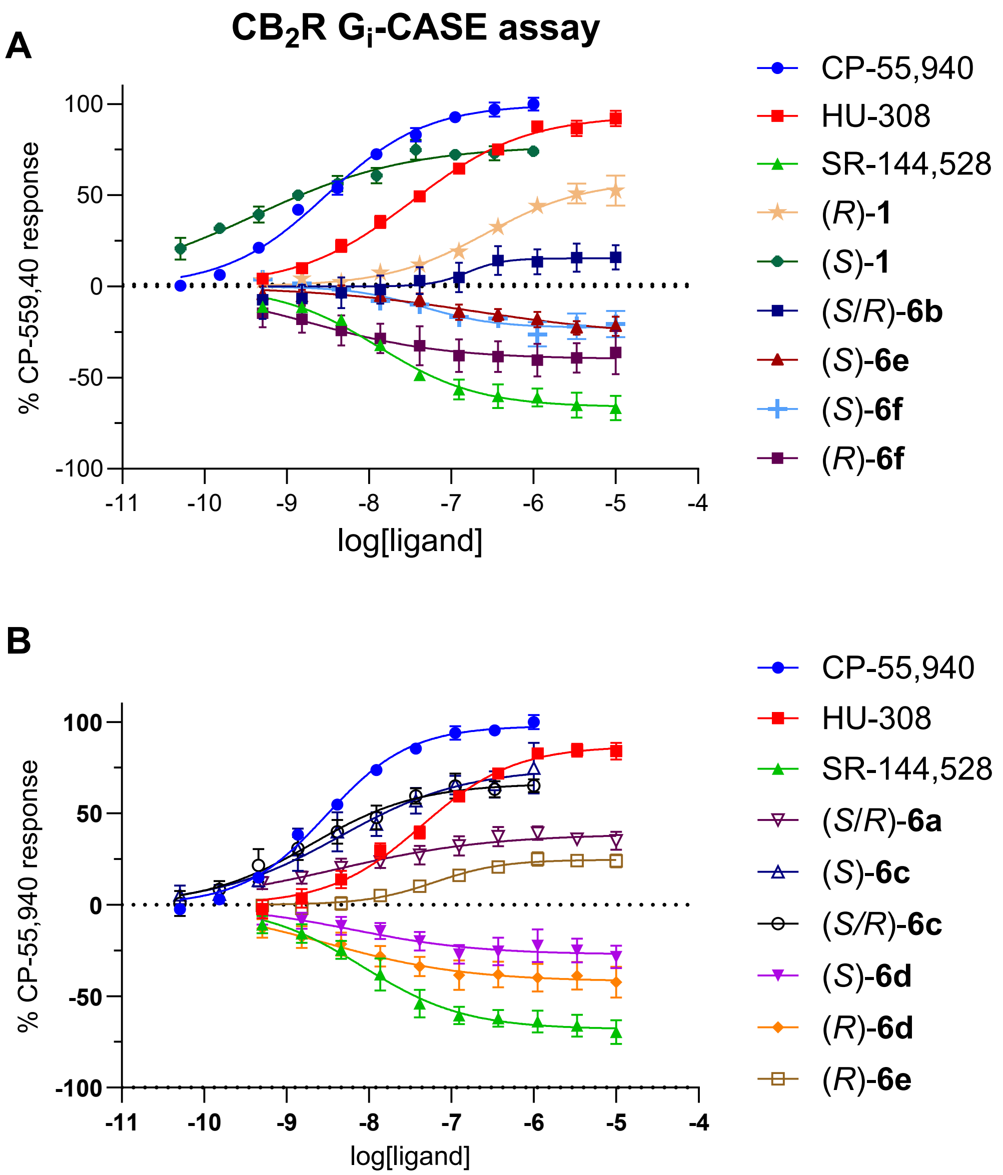

Figure S2. Visualized data from G_i-CASE assay using membrane preparations from hCB_2R-HEK293 T-REx cells. Comparable spectum of response to HTRF cAMP assay: (A) corresponds to Figure S1 A and (B) corresponds to Figure S1 B. Data were normalized to agonist CP-55,940 response (100%) and basal level (0%). Data shown as a mean, $N = 3–4$(±SD).

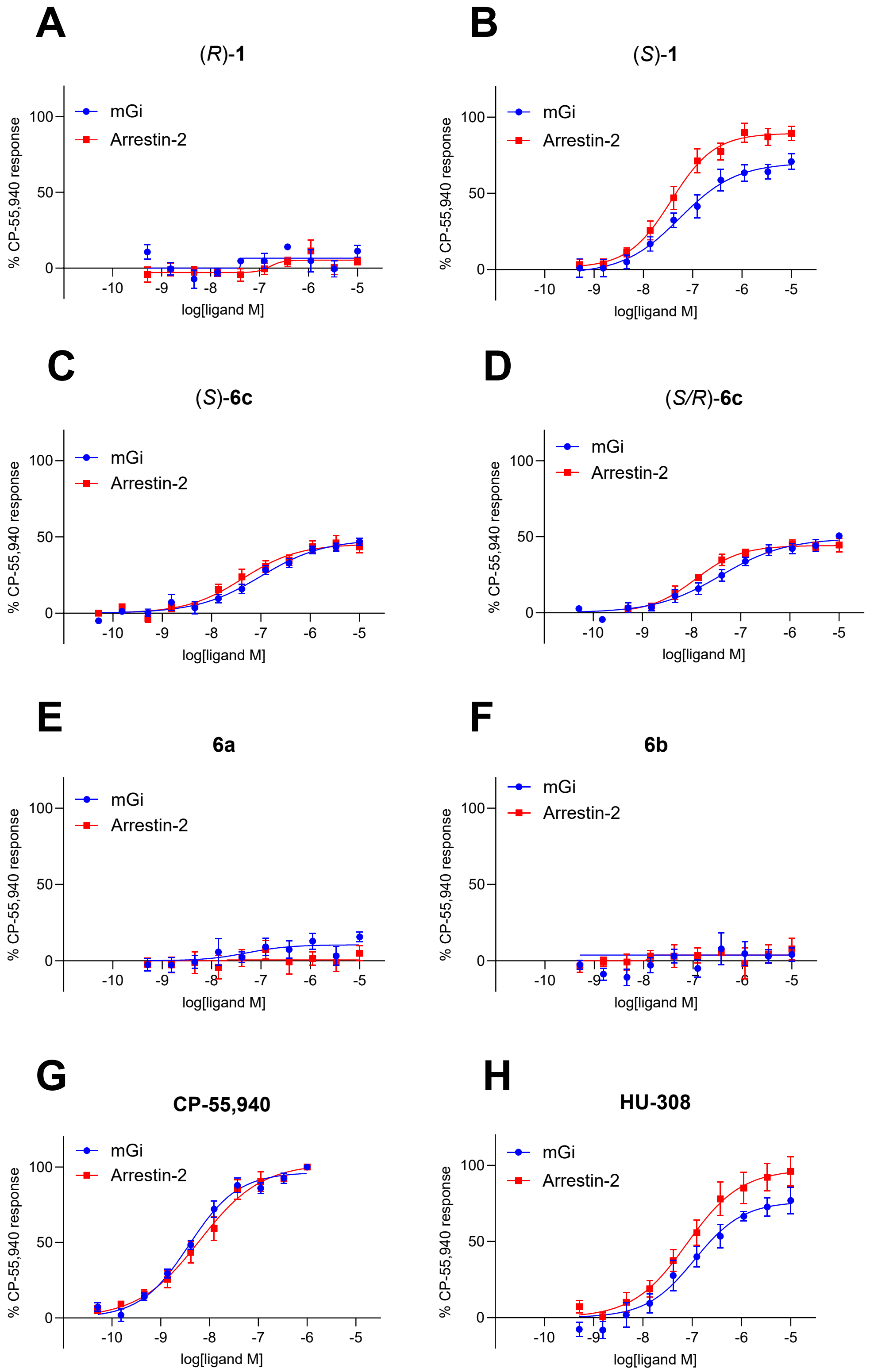

Figure S3-1. Visualized data were obtained in a mini-G$_i$ BRET and β-arrestin BRET assay using hCB$_2$R-HEK293 T-REx cells. Data were normalized to agonist CP-55,940 response (100%) and basal level (0%). Data shown as a mean, N = 6–8 (±SD).

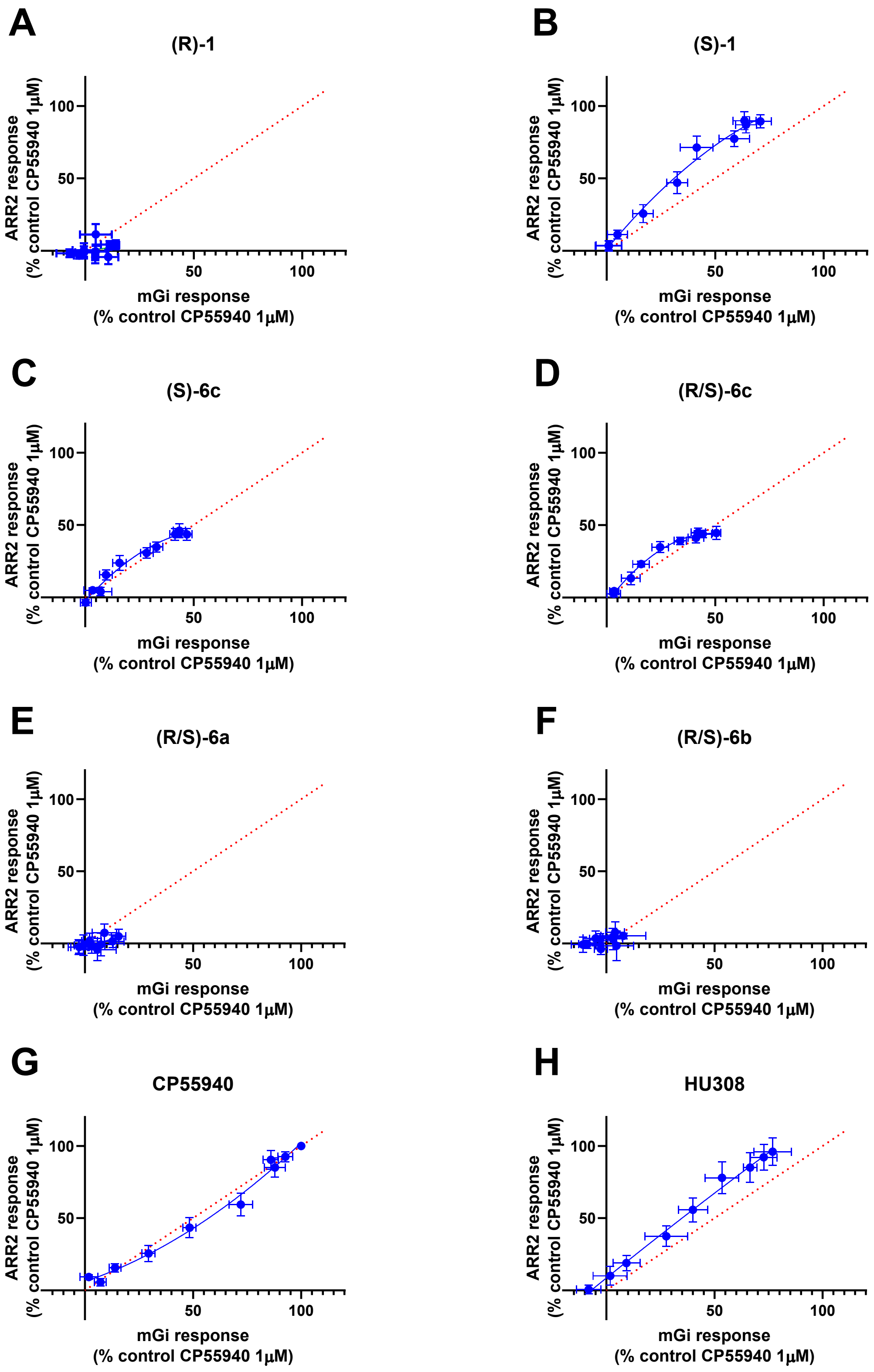

Figure S3-2. Qualitative bias plots for BRET-based CB_2R mG_i and β-arrestin2 recruitment assay responses at equimolar concentrations. CB_2R mGi (x-axis) and β-arrestin2 responses (y-axis) for the CB_2R agonists **(A)** (R)-1, **(B)** (S)-1, **(C)** (S)-6c, **(D)**, (R/S)-6c, **(E)** (R/S)-6a, **(F)** (R/S)-6b, **(G)** CP55940, and **(H)** HU308 plotted at equimolar concentrations. The BRET ratios obtained were normalized using the pathway balanced ligand CP55940 as the reference agonist (100% response). Data shown are the mean ± SEM from 3 or more experiments. A centered second-order polynomial fit of these response values in the respective assays is shown.

Molecular Modeling and Molecular Dynamics Simulations

Molecular docking

Protein Preparation. The crystal structures of the human CB_2 receptor were obtained from the Protein Data Bank. For docking of agonists, the active-state structure in complex with HU-308 (PDB ID: 8GUS) [8] was used, whereas the inactive-state structure in complex with AM10257 (PDB ID: 5ZTY) [9] was used for docking of inverse agonists and antagonists. Protein preparation was performed in MOE[10] v.2019.0102 (Chemical Computing Group, Montreal, Canada) using the QuickPrep module with the following settings: Preserve Sequence and Neutralize enabled, Use Protonate 3D for Protonation = True, Allow ASN/GLN/HIS "Flips" in Protonate3D = True, and Delete Water Molecules Farther than 4.5 Å from Ligand or Receptor = True. The receptor was tethered (Strength = 10, Buffer = 0.25) and atoms farther than 8 Å from the ligand were fixed. Hydrogens close to the ligand were not fixed. Energy minimization was performed to a root mean square (RMS) gradient of 0.1 kcal/mol/Å, with Retain QuickPrep Minimization Restraints enabled.

Ligand Docking. All ligands were docked using static docking, i.e., without modeling induced fit effects. Docking was carried out using GOLD[11] v.5.2.2 integrated into MOE v.2019.0102 with the following parameters: Efficiency = default, Score efficiency = 100, Early Termination (Number = 3, RMS = 1.5), GOLD scoring, and 30 poses per compound. No receptor flexibility was allowed during the docking procedure.

Pose Minimization and Selection. The top 10 docking poses of each compound were energy-minimized within the rigid binding site using MOE: We selected the top-ranked pose per ligand by GOLDScore and report the corresponding Gold.PLP.PLP value in parentheses. For the inactive CB_2R structure (PDB 5ZTY), the best poses gave: p-F-phenyl 121.01 (−82.35), n-propyl 140.06 (−102.55), and phenyl 119.24 (−79.38). For the active CB_2R structure (PDB 8GUS), the best poses were: ethyl 143.05 (−106.71), CF_3 133.42 (−110.71), and HU-308 140.61 (−102.75). These poses correspond to Fig. 2A–F and show no obvious steric clashes.

We emphasize that docking scores are most useful within a ligand to compare alternative poses and diagnose unfavorable contacts/clashes; they should not be used to compare different ligands to each other or to infer relative binding affinities across chemotypes or receptor states

Molecular dynamics: computational methods

Ligands were built with UCSF Chimera 1.17[12] followed by initial energy minimization (EM) at the molecular mechanics level, using AM1-BC charges. The molecules were then fully optimized using the GAMESS program[13] at the Hartree–Fock level with the STO-3G basis set and subjected to HF/6-

31G*/STO-3G single-point calculations to derive the partial atomic charges using the RESP procedure.[14, 15] The complexes were completed by addition of all hydrogen atoms, and they underwent energy minimization (EM) and then molecular dynamics (MD) simulations with Amber20 pmemd.cuda module,[16] using the 14SB version of the AMBER force field for the protein, gaff2 for the ligand and lipid 14 force field parameter for the lipid. The inactivating mutations present in the PDB entry 5ZTY were back-mutated to wild type residues before MD simulations. The energy-minimized complexes were embedded in a pre-equilibrated palmitoyl-oleoyl-phosphatidyl-choline (POPC) lipid bilayer and solvated in an aqueous medium using the charmmgui web-interface (http://www.charmm-gui.org). Potassium and chloride ions were added to ensure electric neutrality and 0.15 M ionic strength. The systems underwent EM and MD simulations following a previously-published.[17] Production runs were carried out at least for 1 μs. Python scripts ante-MMPBSA.py and MMPBSA.py were used to compute MM/PB(GB)SA, selecting the GBn model (igb=5).[18] 1000 frames were taken evenly from the last 500 ns of MD trajectories. A LCPO algorithm[19] was used to estimate solvent accessible area. The Cpptraj module and the UCSF Chimera 1.17 program were used to perform MD analysis and to draw the figures, respectively.

Molecular Dynamics simulations on (*S*)-1 and (*R*)-1 epimers

To rationalize at molecular level the experimentally observed differences at CB_2R in terms of both binding affinity and functional profile (agonist vs antagonist) between (*S*)-**1** and (*R*)-**1** comparative study based on molecular dynamics (MD) in membrane environment was carried out, using the respective docking complexes as starting point. The X-ray structure of CB_2R in the inactive form (PDB:5ZTY) was selected for the study. The rmsd (root mean square deviation) plots of the ligands after best fit of protein backbone showed that both binding poses were stable over 1 μs (Figure S1).

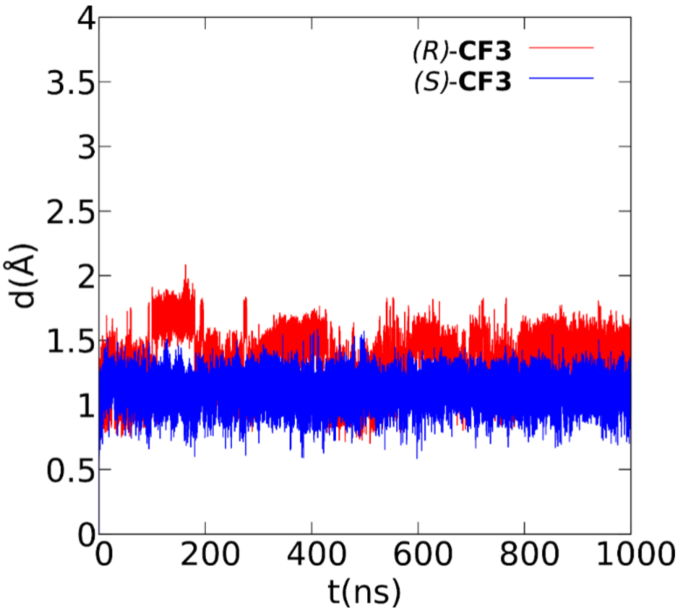

Figure S4. Ligand atom root-mean-square deviation (rmsd) plot calculated for CB_2R with the ligand complexes, after best fit of the protein backbone atoms (except the flexible N-terminus and ICL3 loop) over 1 μs of MD. The rmsd plot was smoothed with a five-point window running average.

Both the epimers adopt the canonical L-shaped conformation in the orthosteric binding site, with the terminal end of the dimethyl-heptyl chain hosted in a cleft formed by aromatic and polar residues such as Phe183^{ECL2}, Tyr190$^{5.39}$, Trp194$^{5.43}$ and Thr114$^{3.33}$. The resorcinol moiety is sandwiched between Phe183^{ECL2} and Phe87$^{2.57}$ while the bicyclo heptene ring is surrounded by aromatic residues (Phe183^{ECL2}, Phe91$^{2.61}$ and Phe94$^{2.64}$). Both compounds are involved in an H-bond between the hydroxyl group on the bicyclic moiety with His95$^{2.65}$ (occurrence 29% and 32% for (*S*)-**1** and (*R*)-**1**, respectively). However, looking at the pattern of interactions engaged by the CF_3 group within the binding site, a clear difference emerges between the two ligands. Only the (*S*)-epimer was able to form a close contact interaction with the indole NE1 atom of the Trp258$^{6.48}$ sidechain, which in turn promotes a reorientation of the Trp258$^{6.48}$ sidechain, as shown in Figure 1 in the article. This interaction is also mirrored by the shorter distance between the fluorine atoms and the Trp258$^{6.48}$NE1 atom in the CB_2R/(*S*)-**1** complex in comparison to the CB_2R/(*R*)-**1** one over the simulated period (Figure S2). The average F-NE1 minimum distance for the (*S*)-epimer over the whole MD trajectory is 4.28 ± 0.0019 Å. The distance is <=3.5 Å in 50.07% of MD frames. For the (*R*)-epimer the distance is 5.62 ±0.0017 Å with 0.98% of MD frames showing a distance of <=3.5 Å.

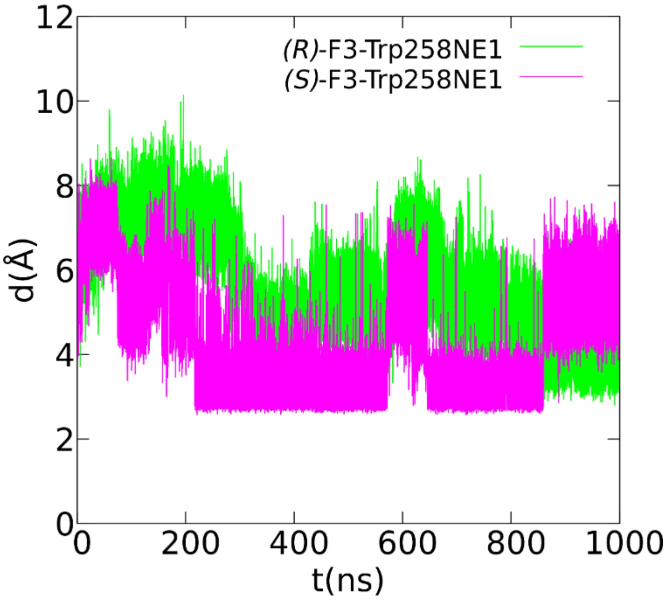

Figure S5. Minimum distance plot between the ligand fluorine atoms (Fx, x=1-3) of the (R)- and the (S)-epimers and the Trp238 NE1 atom calculated over 1 μs of MD. The plot was smoothed with a five-point window running average.

Only in the CB_2R/(*S*)-**1** complex a breakage of the ionic lock between Arg131$^{3.50}$ and Asp240$^{6.30}$ is observed, suggestive of a conformational switch toward the CB_2R activated form (see Figure S3).

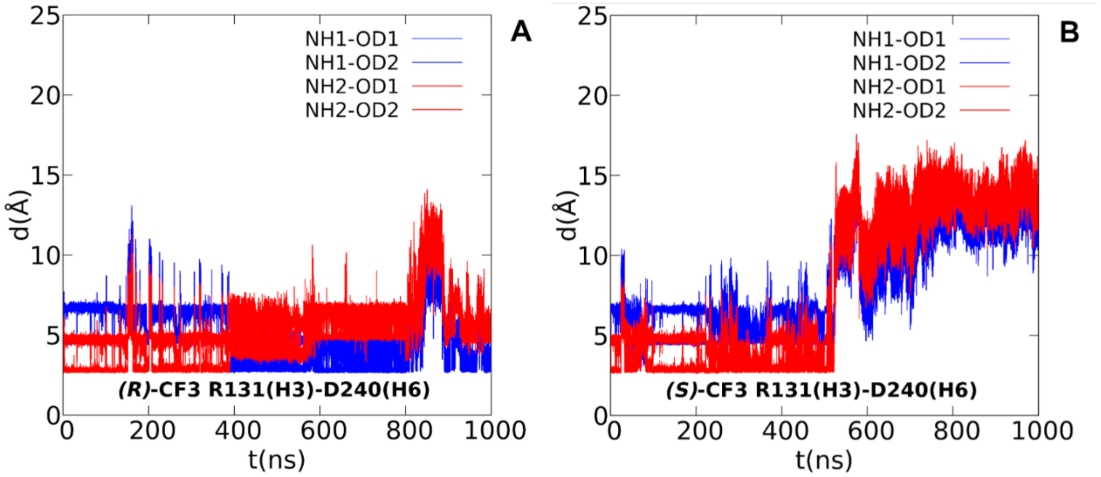

Figure S6. Distance plot between the guanidine nitrogen atoms of R131$^{3.50}$ and the carboxylate oxygen atoms of Asp240$^{6.30}$ in the CB_2R/(*R*)-**1** (A) and CB_2R/(*S*)-**1** (B) complexes over 1 μs of MD simulation. Distance plot was smoothed with a five-point window running average.

The difference in free energy of binding (ΔΔG) between the two epimers, obtained performing molecular mechanics/Poisson-Boltzmann (Generalized Born) surface area (MM/PB(GB)SA) calculations, shows that the (*S*)-epimer is more stable by 3.55 kcal mol^{-1} (MM/GBSA) and 3.53 kcal mol^{-1} (MM/PBSA) than the (*R*)-epimer (−63.00 ± 0.46 kcal mol^{-1} versus -59.45 ± 0.52 (MM/GBSA) and −7.81 ± 0.68 kcal mol^{-1} versus -4.28 ± 0.64 kcal mol^{-1} (MM/PBSA), respectively).

To further assess the propensity of the S-epimer to engage in interaction with Trp258$^{6.48}$, additional MD simulations on CB_2R complexes were carried out using a different starting docking pose, with the bicyclic moiety rotated by ~180° and the hydroxyl group oriented toward Ser90$^{2.60}$ instead of His95$^{2.65}$. Also in this case the occurrence of a close contact interaction was observed in the CB_2R/(*S*)-**1** complex alone (Figure S4), with the relative arrangement of the resorcinol and the alkyl chain conserved inrespect to the ligand poses already described for both the epimers.

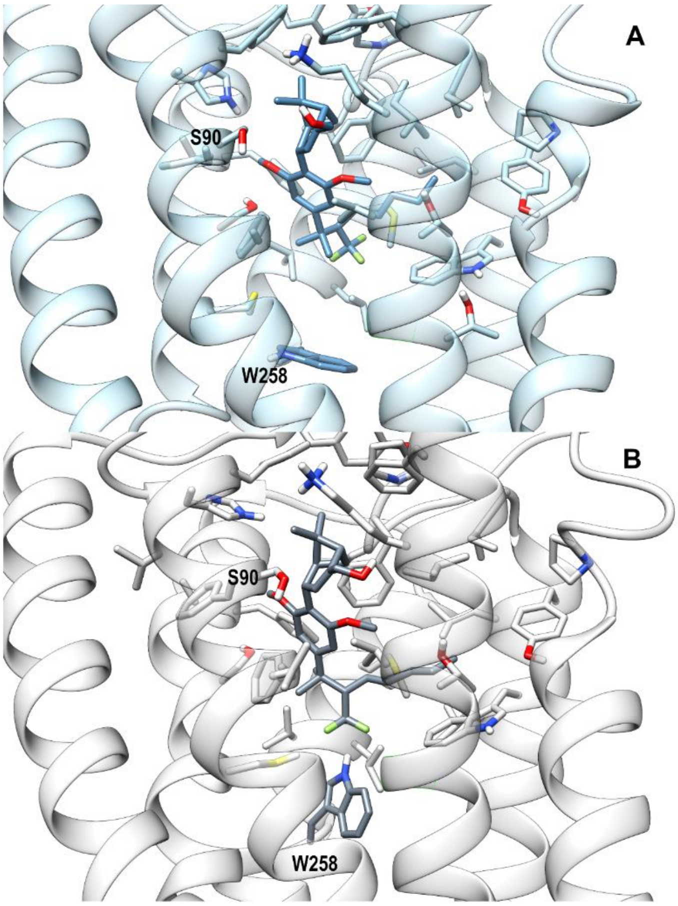

Figure S7. Representative frames from MD of CB2R in complex with (*S*)-**1** and (*R*)-**1** starting from the pose with the hydroxyl group pointing toward Ser90. (*R*)-**1** (panel A) is colored in steel blue, (*S*)-**1** in slate gray (panel B). A stick representation is used for heavy atoms of the ligand and for protein sidechains within 5 Å of the ligand. Protein backbone atoms are represented as ribbons colored in light blue (A) gray (B) according to the sidechains, using half-transparency. Hydrogen, nitrogen, oxygen, and sulfur atoms are painted white, blue, red, and yellow, respectively. A green wire representation is adopted for H-bonds.

General Synthetic Methods

Procedure

Unless otherwise noted, all reactions were carried out under nitrogen atmosphere.

Chemicals

All chemicals and solvents were purchased from commercial suppliers and were used without further purification. DMF, DMSO, THF, Et_2O, MeCN, toluene, benzene and CH_2Cl_2 were dried using 4 Å molecular sieves or using an LC Technology Solutions solvent purification system (SP-1) under an atmosphere of dry nitrogen. Deuterated solvents were purchased from Cambridge Isotopic Laboratories. Pyridine and *i*-Pr_2NEt were distilled from KOH under an atmosphere of dry nitrogen. Et_3N was distilled from CaH_2 under an atmosphere of dry nitrogen.

Chromatography

Analytical thin-layer chromatography (TLC) was performed on Merck silica gel 60 F_{254} TLC glass plates. Purification of reaction products was carried out by flash column chromatography (FCC) using Sigma Aldrich silica 230-400 mesh particle size, 60 Å under 0.3–0.5 bar overpressure or Büchi Pure Chromatography System C-805 Flash with FlashPure silica cartridges.

Nuclear Magnetic Resonance Spectroscopy

NMR spectra were acquired on Bruker AVIII HD 600 MHz, 500 MHz and 400 MHz spectrometers operating at the denoted spectrometer frequency given in MHz for the specified nucleus. ^{1}H NMR spectra are reported with the solvent resonance as the reference ($CDCl_3$ at 7.26 ppm). Peaks are reported as (s = singlet, bs = broad singlet, d = doublet, bt = broad triplet, t = triplet, q = quartet, m = multiplet or unresolved, coupling constant(s) in Hz, integral). ^{13}C NMR spectra were recorded with ^{1}H-decoupling and are reported in ppm with the solvent resonance as the reference ($CDCl_3$ at 77.16 ppm). Service measurements were performed by the NMR service team of the Laboratorium für Organische Chemie at ETH Zürich.

High-Resolution Mass Spectrometry

High-resolution mass spectrometric data were obtained at ETH Zürich mass spectrometry service on Bruker Daltonics maXis ESI-QTOF, Thermo Q Exactive EI-Trace 1310 Analyser or a Bruker Daltonics maXiS II ESI-QTOF spectrometers and are reported as (*m/z*).

Infrared Spectroscopy

Infrared (IR) spectra were measured neat on a Perkin-Elmer UATR Two FT-IR Spectrometer and the band maxima are reported in wavenumbers (cm^{-1}).

Optical Rotation

Optical rotations ($[\alpha]_D^T$) were determined using a Jasco P-2000 Polarimeter (10 cm, 1.5 mL cell).

X-ray Diffraction

Single crystalline samples were measured on a Rigaku Oxford Diffraction XtaLAB Synergy-R kappa diffractometer equipped with a Rigaku HyPix Arc150 HPAD detector and using microfocus rotating anode Cu-Kα radiation with mirror optics (λ = 1.54178 Å).
All measurements were carried out at 100K using an Oxford Cryosystems Cryostream 1000+ sample cryostat. Data were integrated using CrysAlisPro and corrected for absorption effects using a combination of empirical (ABSPACK) and numerical corrections.[20] The structures were solved using SHELXT[21] and refined by full-matrix least-squares analysis (SHELXL),[22, 23] using the program package OLEX2.[24] All non-hydrogen atoms were refined anisotropically and hydrogen atoms were constrained to ideal geometries and refined with fixed isotropic displacement parameters (in terms of a riding model). CCDC2450722 contains the supplementary crystallographic data for this paper, including structure factors and refinement instructions. These data can be obtained free of charge from The Cambridge Crystallographic Data Centre, 12 Union Road, Cambridge CB2 1EZ, UK (fax: +44(1223)-336-033; e-mail: deposit@ccdc.cam.ac.uk), or via https://www.ccdc.cam.ac.uk/structures.

The X-ray diffraction was measured and analyzed by Dr. Nils Trapp and Mr. Michael Solar.

Semi-preparative SFC

The separations were performed using a Prep-SFC-100 semi preparative system with columns (20 × 250 mm, 5 µm) supplied by Daicel, Chiral Technology and 90 mL/min flow of solvent A (CO_2) and co-solvent B, specified by one of the methods below:

Method 1. Column: Chiralpak IC, B = 25% *i*-PrOH
Method 2. Column: Chiralcel IK, B = 15% MeOH
Method 3. Column: Chiralcel IK, B = 16% MeOH
Method 4. Column: Chiralpak IK, B = 18% *i*-PrOH
Method 5. Column: Chiralcel OD-H, B = 20% (EtOH:*n*-heptane, 1:1)
Method 6. Column: Chiralpak AD-H, B = 10% (MeOH:EtOH:*i*-PrOH, 1:1:1)

Synthesis

Synthesis of SI-1

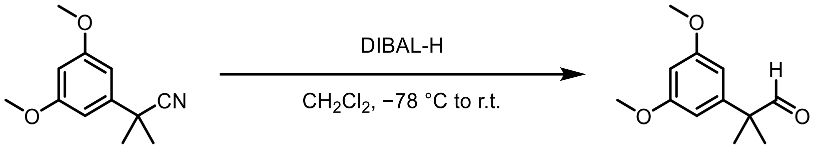

2 **SI-1**

To a solution of **2** (5.00 g, 24.4 mmol, 1.00 equiv) in anhydrous CH_2Cl_2 (85.0 mL) at −78 °C under argon atmosphere was added dropwise DIBAL-H (1.00 M solution in CH_2Cl_2, 60.9 mL, 60.9 mmol, 2.50 equiv). The reaction mixture was stirred at −78 °C for 1 h and subsequently quenched by dropwise addition of Rochelle salt (as a 10% solution in water). The resulting mixture was allowed to warm to ambient temperature, stirred vigorously for 40 min, and then diluted with EtOAc. The phases were separated, and the aqueous phase was extracted with EtOAc. The combined organic layers were washed with brine, dried over $MgSO_4$, and concentrated under reduced pressure. Purification by filtration through a silica plug, eluting with 10 % EtOAc in *n*-hexane afforded the product (4.89 g, 23.5 mmol, 96% yield) as a colorless oil.

^{1}H NMR (400 MHz, $CDCl_3$) δ = 9.46 (s, 1H), 6.41 – 6.40 (m, 2H), 6.39 – 6.38 (m, 1H), 3.79 (s, 6H), 1.43 (s, 6H) ppm.

Data in accordance with that reported in the literature.[25]

Synthesis of SI-2

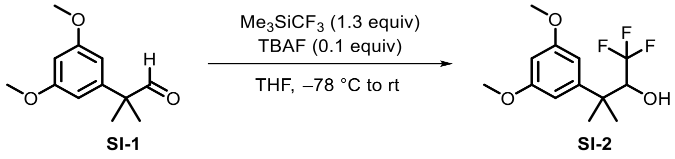

To a solution of **SI-1** (2.00 g, 9.60 mmol, 1.0 equiv) in anhydrous THF (16.0 mL) at –78 °C was added Me_3SiCF_3 (1.85 mL, 12.5 mmol, 1.3 equiv) followed by a solution of TBAF (1.0 M in THF, 960 µL, 960 µmol, 0.1 equiv). The mixture was allowed to warm up to rt and stirred overnight. Additional Me_3SiCF_3 (710 µL, 4.80 mmol, 0.5 equiv) and a solution of TBAF (1.0 M in THF, 960 µL, 960 µmol, 0.1 equiv) were added dropwise and stirred for 1 h to bring the reaction to completion. The mixture was quenched with water (25 mL), extracted with Et_2O (3 × 25 mL) and the organic extracts concentrated under reduced pressure. The unpurified mixture was dissolved in THF (10 mL) and aq. 1 M HCl (10 mL) and refluxed for 1h. The mixture was cooled down to rt extracted with Et_2O (3 × 25 mL), combined organic extracts dried with $MgSO_4$, filtered and concentrated under reduced pressure. The material was purified by flash column chromatography (SiO_2; using 0 – 30% Et_2O in hexanes) to yield the product (2.50 g, 8.98 mmol, 94%). as a colorless liquid.

^{1}H NMR (400 MHz, $CDCl_3$) δ 6.55 (d, J = 2.2 Hz, 2H), 6.37 (t, J = 2.2 Hz, 1H), 4.06 (td, J = 7.5, 5.6 Hz, 1H), 3.80 (s, 6H), 2.28 (d, J = 5.6 Hz, 1H), 1.49 – 1.42 (m, 6H).

^{13}C NMR (101 MHz, $CDCl_3$) δ 160.9, 148.0, 125.4 (q, J = 284.4 Hz), 105.4, 98.0, 77.1 (q, J = 28.1 Hz), 55.4, 41.2, 24.8 (q, J = 2.4 Hz), 23.7 (q, J = 2.0 Hz).

^{19}F NMR (377 MHz, $CDCl_3$) δ -71.2.

IR (neat, ν_{max}/cm^{-1}): 3472, 2942, 2841, 1597, 1458, 1425, 1270, 1156.

HRMS (ESI): *m/z* = 279.1204 $[M+H]^+$ (calc. for $C_{13}H_{18}F_3O_3$ *m/z* = 279.1203)

Synthesis of 3

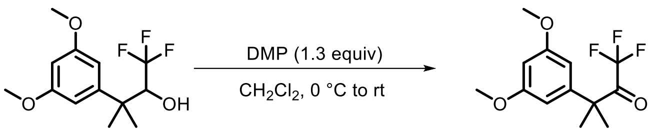

To a solution of **SI-2** (2.70 g, 9.70 mmol, 1.0 equiv) in anhydrous CH_2Cl_2 (29.0 mL) at 0 °C was added DMP (5.35 g, 12.6 mmol, 1.3 equiv) in one portion. The mixture was allowed to warm up to rt and stirred for 2 h. The mixture was concentrated under reduced pressure and the residue triturated with pentane (50 mL). The white solid was filtered off and the filter cake was washed with pentane (2 × 50 mL). The organic washings were concentrated under reduced pressure and the residue was purified by flash column chromatography (SiO_2; using 0 – 15% Et_2O in hexanes) to afford the product (2.10 g, 7.60 mmol, 78%) as a colorless liquid.

^{1}H NMR (400 MHz, $CDCl_3$) δ 6.39 (t, J = 2.2 Hz, 1H), 6.34 (d, J = 2.2 Hz, 2H), 3.78 (s, 6H), 1.58 (q, J = 0.8 Hz, 6H).

^{13}C NMR (101 MHz, $CDCl_3$) δ 193.8 (q, J = 31.2 Hz), 161.4, 142.7, 116.2 (q, J = 295.1 Hz), 104.3, 99.1, 55.4, 50.2, 24.7 (q, J = 1.4 Hz).

^{19}F NMR (377 MHz, $CDCl_3$) δ -71.0.

IR (neat, ν_{max}/cm^{-1}): 2941, 2841, 1745, 1596, 1458, 1426, 1371, 1341, 1299, 1261, 1203.

HRMS (ESI): m/z = 277.1047 $[M+H]^+$ (calc. for $C_{13}H_{16}F_3O_3$ m/z = 277.1046).

Synthesis of (*E*)-SI-3 & (*Z*)-SI-3

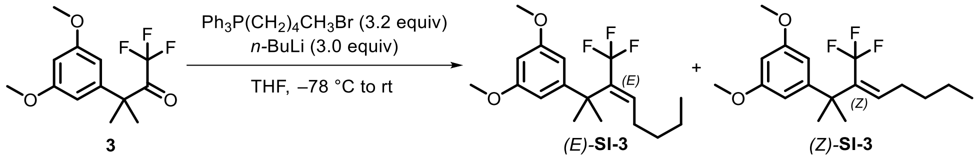

To a suspension of pentyltriphenylphosphonium bromide (1.92 g, 4.63 mmol, 3.2 equiv) at –78 °C in dry THF (40 mL)) was added dropwise *n*-BuLi (1.6 M in hexane, 2.71 mL, 4.34 mmol, 3.0 equiv). The solution was stirred for 10 min at –78 °C, allowed to warm up to ambient temperature, and stirred for additional 30 min. Subsequently, **3** (400 mg, 1.45 mmol, 1.0 equiv) was dissolved in dry THF (1 mL) and added dropwise to the mixture at 0 °C. The mixture was stirred overnight at rt, quenched with aq. sat. NH_4Cl solution (25 mL), diluted with Et_2O (25 mL) and the phases were separated. The aqueous phase was extracted with Et_2O (2 × 25 mL). The combined organic extracts were dried over $MgSO_4$, filtered and concentrated under reduced pressure. The residue was purified by flash column chromatography (SiO_2; using 0 – 10% Et_2O in hexanes) to yield separable (*E*)-**SI-3** (210 mg, 636 µmol, 44%) and (*Z*)-**SI-3** (145 mg, 438 µmol, 30%) isomers, respectively, as light yellow liquids.
Note: For the purposes of synthetic ease, the (*E*)- and (*Z*)-isomers do not need to be separated before subjection to the subsequent hydrogenation step.

(*E*)-**3.55**
^{1}H NMR (500 MHz, $CDCl_3$) δ 6.48 (d, J = 2.2 Hz, 2H), 6.30 (t, J = 2.2 Hz, 1H), 6.14 (tq, J = 7.6, 2.0 Hz, 1H), 3.78 (s, 6H), 1.64 – 1.57 (m, 2H), 1.51 (q, J = 1.2 Hz, 6H), 1.13 – 0.98 (m, 4H), 0.70 (t, J = 7.1 Hz, 3H).

^{13}C NMR (126 MHz, $CDCl_3$) δ 160.8, 153.5, 138.18 (q, J = 7.7 Hz), 135.30 (q, J = 24.4 Hz), 125.17 (q, J = 274.4 Hz), 104.5, 97.2, 55.4, 41.8, 30.5, 29.91 (q, J = 1.9 Hz), 28.3, 22.4, 13.9.

^{19}F NMR (471 MHz, $CDCl_3$) δ -60.9.

IR (neat, ν_{max}/cm^{-1}): 2958, 2931, 2874, 2838, 1649, 1596, 1457, 1423, 1290.

HRMS (ESI): *m/z* = 331.1882 $[M+H]^+$ (calc. for $C_{18}H_{26}F_3O_2$ *m/z* = 331.1879).

(*Z*)-**3.55**
^{1}H NMR (500 MHz, $CDCl_3$) δ 6.43 (d, J = 2.2 Hz, 2H), 6.32 (t, J = 2.2 Hz, 1H), 6.04 (t, J = 7.7 Hz, 1H), 3.78 (s, 6H), 2.41 – 2.33 (m, 2H), 1.53 – 1.48 (m, 2H), 1.47 (s, 6H), 1.45 – 1.36 (m, 2H), 0.96 (t, J = 7.2 Hz, 3H).

^{13}C NMR (126 MHz, $CDCl_3$) δ 160.8, 150.7, 137.4 (q, J = 25.2 Hz), 137.0 (q, J = 3.5 Hz), 124.9 (q, J = 279.4 Hz), 104.1, 97.4, 55.3, 43.1, 31.8, 29.6, 28.5, 22.5, 14.0.

^{19}F NMR (471 MHz, $CDCl_3$) δ -53.9.

IR (neat, ν_{max}/cm^{-1}): 2959, 2933, 2874, 2838, 1652, 1596, 1457, 1424, 1204.

HRMS (ESI): *m/z* = 331.1881 $[M+H]^+$ (calc. for $C_{18}H_{26}F_3O_2$ *m/z* = 331.1879).

Synthesis of SI-4

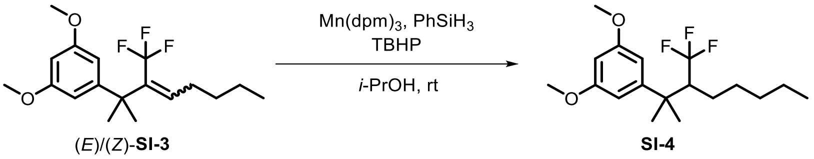

To a solution of **SI-3** (1.20 g, 3.63 mmol, 1.0 equiv) in *i*-PrOH (70 mL) was added phenylsilane (1.22 g, 1.39 mL, 11.3 mmol, 3.1 equiv) and *t*-BuOOH (2.64 mL, 14.5 mmol, 5.5 M in water, 4.0 equiv), and the solution was degassed by bubbling Ar under vigorous stirring for 15 minutes. Then, $Mn(dpm)_3$ (659 mg, 1.09 mmol, 0.300 equiv) was added in one portion, and the dark reaction mixture was degassed by bubbling Ar for another 5 min. The reaction mixture turned pale brown after 5 min, light yellow after 20 min. The mixture was stirred for 20 h and return of a dark brown color of the solution indicated reductant consumption. Sat. aq $NaHCO_3$ and Na_2SO_5 (50:50 v/v%) were added, and the heterogeneous brown mixture was diluted with CH_2Cl_2. The phases were separated and the aqueous layer was extracted with CH_2Cl_2 (3 × 50 mL). The combined organic layers were washed with brine, dried over Na_2SO_4, filtered, and concentrated under reduced pressure. Filtration over a plug of silica (10% Et_2O in hexane) afforded a brown oil, which displayed incomplete reaction (as judged by NMR) and was re-subjected to the exact same conditions for a second cycle of hydrogenation. The residue was then purified by flash column chromatography (SiO_2, dry load on celite, 1 to 1.5% Et_2O in hexane), affording the product (1.00 g, 3.01 mmol, 83% yield) as a colorless oil.

^{1}H NMR (500 MHz, $CDCl_3$) δ 6.49 (d, J = 2.2 Hz, 2H), 6.34 (t, J = 2.2 Hz, 1H), 3.80 (s, 6H), 2.37 (qdd, J = 10.3, 7.7, 2.7 Hz, 1H), 1.54 – 1.43 (m, 1H), 1.41 (d, J = 2.0 Hz, 3H), 1.35 (s, 3H), 1.29 – 1.20 (m, 1H), 1.18 – 0.97 (m, 6H), 0.78 (t, J = 7.2 Hz, 3H).

^{13}C NMR (126 MHz, $CDCl_3$) δ 160.7, 151.5, 129.07 (q, J = 283.3 Hz), 105.0, 97.4, 55.4, 52.50 (q, J = 22.1 Hz), 40.4, 31.7, 29.4, 28.33 (q, J = 2.5 Hz), 26.26 (q, J = 2.4 Hz), 23.80 (q, J = 1.8 Hz), 22.3, 14.0.

^{19}F NMR (471 MHz, $CDCl_3$) δ -62.1.

IR (neat, ν_{max}/cm^{-1}): 2957, 2873, 1597, 1458, 1206, 1156.

HRMS (ESI): *m/z* = 333.2033 $[M+H]^+$ (calc. for $C_{18}H_{28}F_3O_2$ *m/z* = 333.2036).

Synthesis of (+)-(R)-4/(A)-4 & (–)-(S)-4/(B)-4

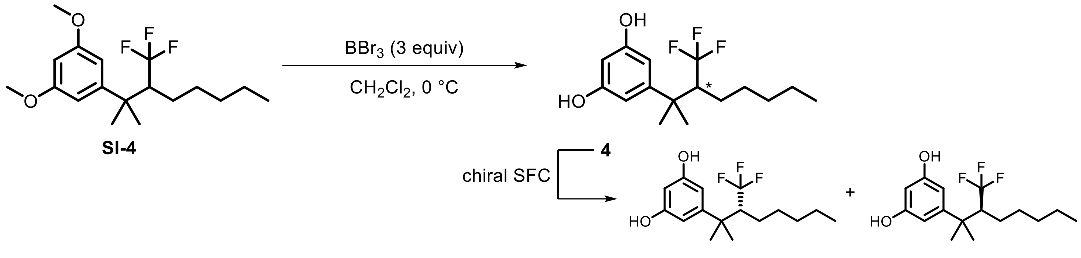

(*R*)-**4**, %ee > 99% =(A)-**4**

(*S*)-**4**, %ee = 98% =(B)-**4**

To a solution of **SI-4** (100 mg, 301 µmol, 1.0 equiv) in anhydrous CH_2Cl_2 (3.0 mL) at 0 °C was added BBr_3 (86 µL, 903 µmol, 3.0 equiv) dropwise and the solution was stirred at 0 °C for 2 h. The mixture was carefully quenched with aq. sat. $NaHCO_3$ (5 mL) and diluted with Et_2O (10 mL). The layers were separated and the aqueous phase was extracted with Et_2O (2 × 10 mL). Combined organic extracts were dried over $MgSO_4$ and concentrated under reduced pressure. Purification by flash column chromatography (SiO_2; using 0 – 30% EtOAc in hexanes) afforded the product as a light red oil (91 mg, 299 µmol, 99%).

The racemate **4** was resolved by chiral SFC Method 4 to afford enantiomers (+)-(*R*)-**4** (38 mg, 138 µmol, 42%, %ee > 99%) and (–)-(*S*)-**4** (115 mg, 38%, %ee = 98%). Please refer to the following section for the stereochemical assignment. Prefixes (A)- and (B)- denote the elution sequence on the chiral column and were used for identification before the assignment of configuration (Scheme S1 and Scheme S2).

^{1}H NMR (400 MHz, $CDCl_3$) δ 6.42 (d, J = 2.2 Hz, 2H), 6.23 (t, J = 2.2 Hz, 1H), 5.51 (bs, 2H, phenol OHs), 2.30 (qdd, J = 10.2, 7.5, 2.6 Hz, 1H), 1.51 – 0.96 (m, 8H), 1.34 (q, J = 1.9 Hz, 3H), 1.28 (s, 3H), 0.79 (t, J = 7.1 Hz, 3H).

^{13}C NMR (101 MHz, $CDCl_3$) δ 156.4, 152.6, 128.93 (q, J = 283.4 Hz), 106.3, 100.8, 52.52 (q, J = 22.1 Hz), 40.1, 31.7, 29.4, 27.6, 26.20 (q, J = 2.4 Hz), 24.3, 22.3, 14.0.

^{19}F NMR (376 MHz, $CDCl_3$) δ -62.1.

IR (neat, ν_{max}/cm^{-1}): 3349, 2957, 2928, 2872, 1598, 1459, 1256, 1148.

HRMS (ESI): *m/z* = 305.1721 $[M+H]^+$ (calc. for $C_{16}H_{24}F_3O_2$ *m/z* = 305.1723).

(*R*)-4 $[\alpha]^{25}_{D}$ = +17.570 ± 0.110 (c = 1.0, $CHCl_3$).

(*S*)-4 $[\alpha]^{25}_{D}$ = –16.414 ± 0.153 (c = 1.0, $CHCl_3$).

X-Ray Crystallographic Studies for the Stereochemical Assignment of (+)-(R)-4/(–)-(S)-4

Derivatizable Sidechain Synthesis:

Scheme S1. Synthesis of (A)-SI-8/(B)-SI-8 from intermediates **3** and **SI-5**.[a]

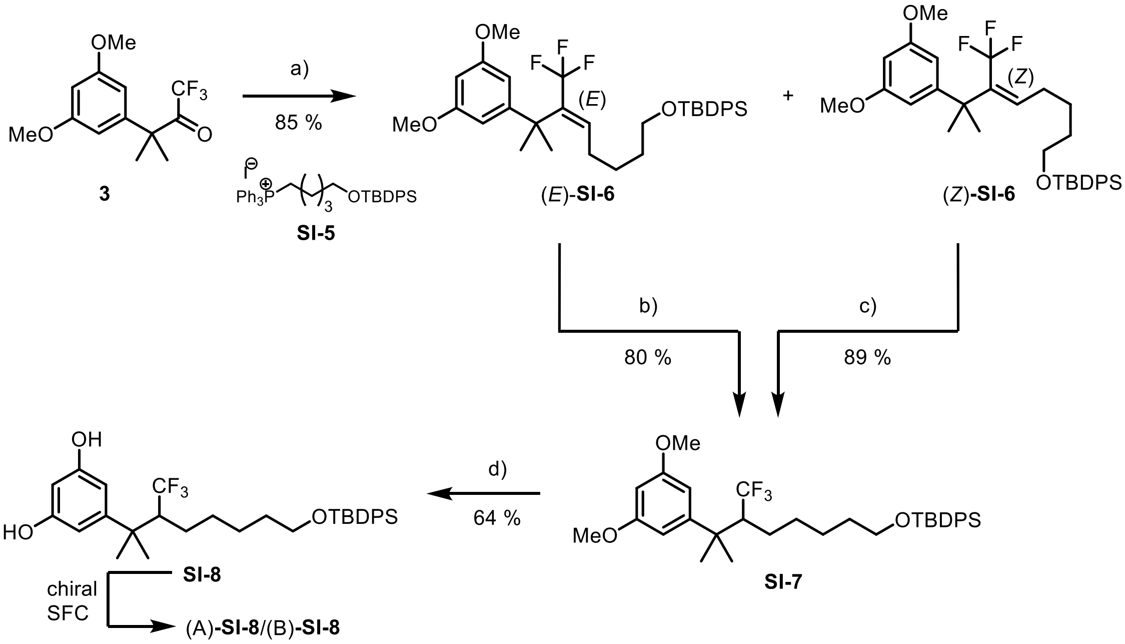

[a] Reagents and conditions: (a) **3**, *n*-BuLi, −78 °C to r.t. to 0 °C to r.t., 85 %; (b) Mn(dpm)$_3$, $PhSiH_3$, TBHP, ^{i}PrOH, r.t., 24 h, 80 % after 3 cycles; (c) Mn(dpm)$_3$, $PhSiH_3$, TBHP, ^{i}PrOH, r.t., 24 h, 89 %; (d) BCl_3, TBAI, CH_2Cl_2, 0 °C, 1 h, 64 %.

Synthesis of Derivative (B)-**SI-9** Suitable for Crystallization/X-Ray Crystallographic Analysis & Defunctionalization of (A)-**SI-8** for Optical Rotation Comparison with Enantiomerically Enriched (A)-4. As before, prefixes (A)- and (B)- denote elution sequence and were used as identifiers before assignment:

Scheme S2. Synthesis of (B)-**SI-9** for crystallization and compound (A)-**SI-10** for comparison to authentic material.[a]

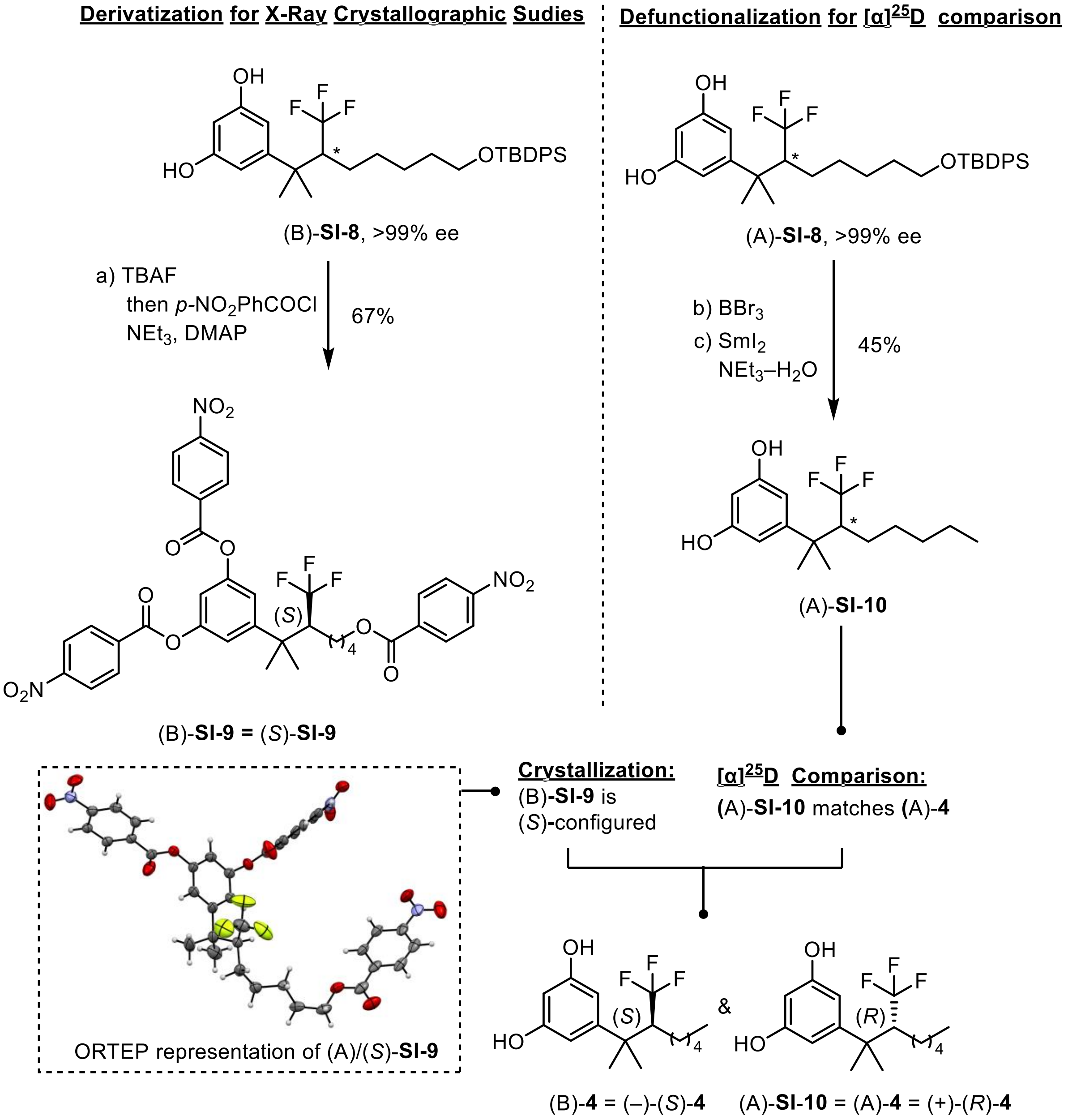

[a] Reagents and conditions: (a) TBAF, THF, rt, then *p*-$NO_2PhCOCl$, NEt_3, DMAP, CH_2Cl_2, rt, (b) BBr_3, CH_2Cl_2, 0°C to rt, 67% (c) SmI_2, H_2O–NEt_3, rt, 45%, steps.

Synthesis of SI-11

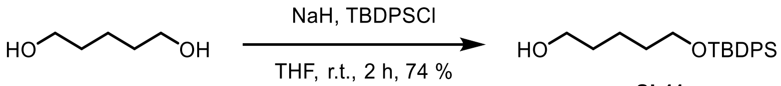

To a suspension of sodium hydride (60% in mineral oil, 1.15 g, 28.8 mmol, 1.0 equiv) in anhydrous THF (60.0 mL) was added pentane-1,5-diol (3.00 g, 3.02 mL, 28.8 mmol, 1.0 equiv) and the mixture was allowed to stir at room temperature for 1 h. Then, TBDPSCl (7.92 g, 7.49 mL, 28.8 mmol, 1.0 equiv) was added under vigorous stirring, and the reaction mixture was further stirred for 1 h. The reaction mixture was quenched by addition of saturated aqueous $NaHCO_3$ solution, the phases were separated, and the aqueous phase was extracted with EtOAc. The combined organic layers were washed with brine, dried over Na_2SO_4, and concentrated under reduced pressure. Purification by flash column chromatography (SiO_2, 30 % EtOAc in *n*-hexane) afforded the product (7.40 g, 21.6 mmol, 75 %) as a colorless oil.

^{1}H NMR (400 MHz, $CDCl_3$) δ = 7.69 – 7.65 (m, 4H), 7.45 – 7.36 (m, 6H), 3.68 (t, J = 6.4 Hz, 2H), 3.65 – 3.59 (m, 2H), 1.66 – 1.49 (m, 4H), 1.49 – 1.38 (m, 2H), 1.27 (s, 1H), 1.06 (s, 9H) ppm.

Data in accordance with that reported in the literature:[26]

Synthesis of SI-12

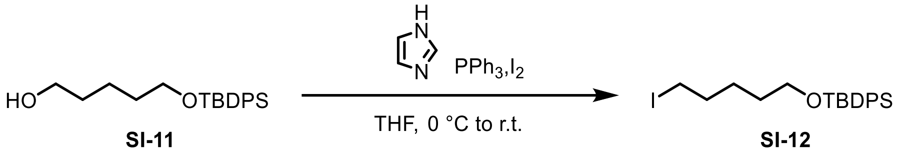

To a solution of alcohol **SI-11** (4.00 g, 11.7 mmol, 1.0 equiv), triphenylphosphine (4.59 g, 17.5 mmol, 1.5 equiv), and imidazole (1.19 g, 0.969 mL, 17.5 mmol, 1.5 equiv) in anhydrous THF (78.0 mL) at 0 °C was added molecular iodine (4.45 g, 17.5 mmol, 1.5 equiv) in one portion. The reaction mixture was allowed to warm to ambient temperature and stirred for 1 h. Then, the reaction mixture was diluted with Et_2O, washed with aqueous 10 % $Na_2S_2O_3$ solution, brine, dried over Na_2SO_4, and concentrated under reduced pressure. Purification by trituration in *n*-hexane afforded the product (5.25 g, 11.6 mmol, 99 %) as a pale-yellow oil.

^{1}H NMR (400 MHz, CDCl3) δ = 7.69 – 7.64 (m, 4H), 7.45 – 7.35 (m, 6H), 3.66 (t, J = 6.2 Hz, 2H), 3.16 (t, J = 7.0 Hz, 2H), 1.81 (p, J = 7.1 Hz, 2H), 1.61 – 1.54 (m, 2H), 1.52 – 1.44 (m, 2H), 1.05 (s, 9H) ppm.

^{13}C NMR (101 MHz, $CDCl_3$) δ = 135.7, 134.1, 129.7, 127.8, 63.7, 33.4, 32.4, 31.6, 27.0, 19.4, 7.2 ppm.

IR (neat, ν_{max}/cm^{-1}) 3070, 2931, 2857, 1472, 1428, 1390, 1361, 1213, 1172, 1111, 998, 823, 740, 701, 614, 541, 505, 493.

ESI-HRMS m/z calcd for $C_{21}H_{29}INaOSi$ $[M+Na]^+$ 475.0925; found 475.0925.

Synthesis of SI-5

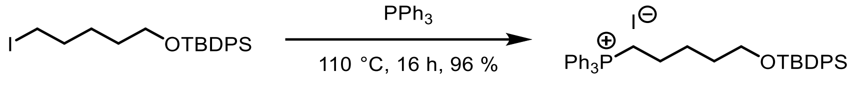

Triphenylphosphine (3.35 g, 12.8 mmol, 1.1 equiv) was added to neat **SI-12** (5.25 g, 11.6 mmol, 1.0 equiv), and the reaction mixture was stirred at 110 °C for 1 h. Then, the reaction mixture was allowed to cool to ambient temperature, at which point it developed a gum-like consistency. Et_2O was added, and the semi-solid mixture was ultrasonicated for 10 min, facilitating solidification. Subsequently, the mixture was filtered, the residue was washed with Et_2O, and dried under reduced pressure to give the product (7.95 g, 11.1 mmol, 96 %) as a colorless solid.

^{1}H NMR (400 MHz, $CDCl_3$) δ = 7.85 – 7.75 (m, 9H), 7.71 – 7.65 (m, 6H), 7.61 – 7.57 (m, 4H), 7.41 – 7.30 (m, 6H), 3.76 – 3.67 (m, 2H), 3.61 (t, J = 6.3 Hz, 2H), 1.78 – 1.64 (m, 2H), 1.62 – 1.52 (m, 4H), 0.98 (s, 9H) ppm.

^{13}C NMR (101 MHz, $CDCl_3$) δ = 135.6, 135.2, 135.2, 133.9, 133.8, 130.7, 130.6, 129.7, 127.8, 118.8, 118.0, 63.5, 32.0, 27.1, 27.0, 23.6, 23.1, 22.6, 19.3 ppm.

^{31}P NMR (162 MHz, $CDCl_3$) δ = 24.4 ppm.

IR (neat, ν_{max}/cm^{-1}) 2930, 2857, 1587, 1472, 1438, 1111, 996, 824, 742, 723, 705, 689, 614, 532, 498.

ESI-HRMS m/z calcd for $C_{39}H_{44}OPSi$ $[M]^+$ 587.2894; found 587.2888.

Synthesis of (*E*)-SI-6/(*Z*)-SI-6

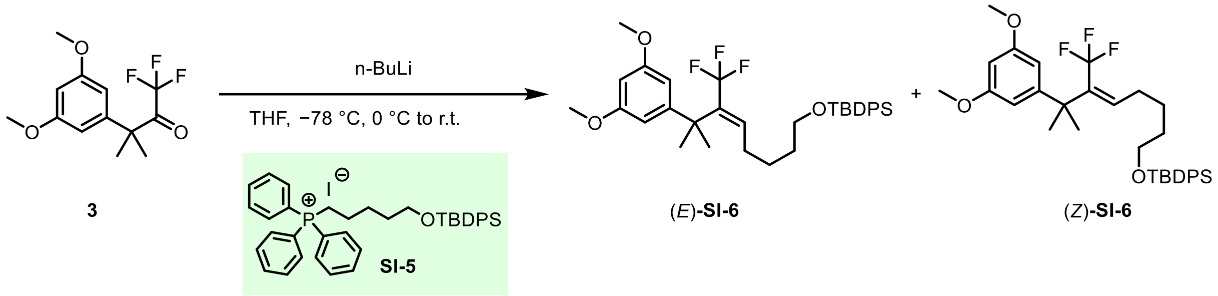

To a suspension of **3** (3.10 g, 4.34 mmol, 1.2 equiv) in anhydrous THF (36.0 mL) at −78 °C was added dropwise butyllithium (.60 mL, 4.16 mmol, 1.6 M in *n*-hexane2, 1.15 equiv). The solution was stirred for 10 min at −78 °C, allowed to warm to ambient temperature, and stirred for additional 30 min. Then, the reaction mixture was cooled to 0 °C, and a solution of ketone **3** (1.00 g, 3.62 mmol, 1.0 equiv) in anhydrous THF (2.00 mL) was added dropwise. The reaction mixture was allowed to reach ambient temperature and stirred for further 2 h. Subsequently, the reaction mixture was quenched with sat. aq. NH_4Cl solution, diluted with Et_2O, the phases were separated, and the aqueous phase was extracted with Et_2O. Combined organic extracts were dried over Na_2SO_4 and concentrated under reduced pressure. Purification by flash column chromatography (SiO_2, eluting with 1–2 % Et_2O in *n*-hexane) afforded the two diastereomers as colourless oils and a mixed fraction. The mixed fraction was further purified by flash column chromatography (SiO_2, eluting with 1 % Et_2O and 10 % CH_2Cl_2 in *n*-hexane), affording the products **(*E*)-SI-6** (1.10 g, 1.93 mmol, 53 %) and **(*Z*)-SI-6** (655 mg, 1.15 mmol, 32 %), after merging fractions.

<u>(*E*)-**SI-6**</u>

^{1}H NMR (400 MHz, $CDCl_3$) δ = 7.66 – 7.58 (m, 4H), 7.46 – 7.32 (m, 6H), 6.45 (d, *J* = 2.3 Hz, 2H), 6.26 (t, *J* = 2.2 Hz, 1H), 6.12 (td, *J* = 7.5, 2.0 Hz, 1H), 3.73 (s, 6H), 3.48 (t, *J* = 6.2 Hz, 2H), 1.63 – 1.57 (m, 2H), 1.50 (bs, 6H), 1.30 – 1.13 (m, 4H), 1.02 (s, 9H) ppm.

^{13}C NMR (101 MHz, $CDCl_3$) δ = 160.8, 153.4, 138.0, 135.7, 134.1, 129.7, 127.7, 104.5, 97.2, 63.6, 55.4, 41.8, 32.2, 29.9, 28.3, 27.0, 24.7, 19.3 ppm.

^{19}F NMR (377 MHz, $CDCl_3$) δ = −60.9 ppm.

IR (neat, ν_{max}/cm^{-1}) 3072, 2934, 2859, 1648, 1597, 1459, 1427, 1390, 1300, 1206, 1193, 1169, 1156, 1107, 1067, 938, 824, 741, 702, 689, 614, 505.

ESI-HRMS m/z calcd for $C_{34}H_{43}F_3NaO_3Si$ $[M+Na]^+$ 607.2826; found 607.2818.

(Z)-**SI-6**

^{1}H NMR (400 MHz, $CDCl_3$) δ = 7.70 – 7.65 (m, 4H), 7.45 – 7.36 (m, 6H), 6.41 (d, *J* = 2.2 Hz, 2H), 6.30 (t, *J* = 2.2 Hz, 1H), 6.00 (t, *J* = 7.7 Hz, 1H), 3.76 (s, 6H), 3.70 (t, *J* = 6.0 Hz, 2H), 2.40 – 2.29 (m, 2H), 1.67 – 1.56 (m, 4H), 1.45 (s, 6H), 1.06 (s, 9H) ppm.

^{13}C NMR (101 MHz, $CDCl_3$) δ = 160.8, 150.6, 136.7, 135.7, 134.2, 129.7, 127.8, 126.3, 104.1, 97.4, 63.7, 55.3, 43.1, 32.3, 29.6, 28.5, 27.0, 25.9, 19.4 ppm.

^{19}F NMR (377 MHz, $CDCl_3$) δ = −53.9 ppm.

IR (neat, ν_{max}/cm^{-1}) 3072, 2999, 2934, 2859, 1652, 1596, 1459, 1427, 1389, 1377, 1362, 1339, 1311, 1291, 1251, 1203, 1156, 1110, 1067, 1006, 938, 824, 741, 702, 614, 505.

ESI-HRMS m/z calcd for $C_{34}H_{43}F_3NaO_3Si$ $[M+Na]^+$ 607.2826; found 607.2830.

Synthesis of SI-7

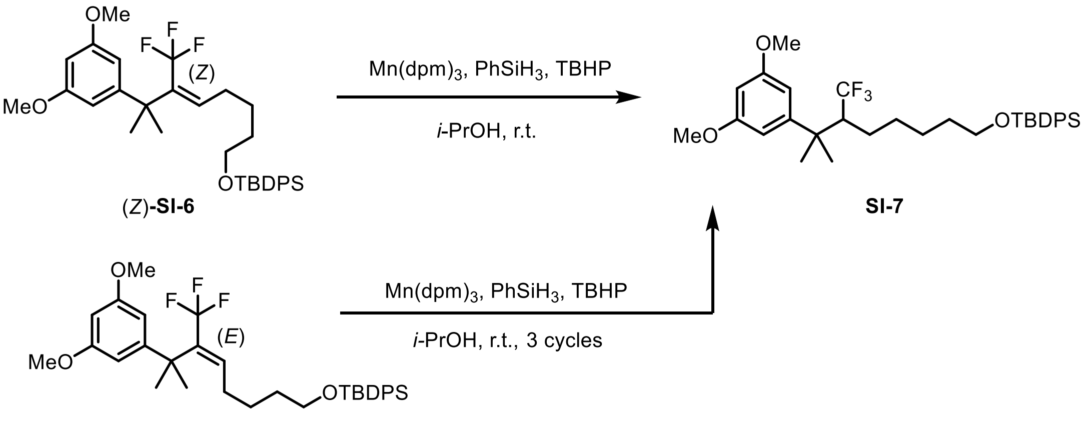

To a solution of (*Z*)-**SI-6** (630 mg, 1.08 mmol, 1.0 equiv) was added phenyl silane (361 mg, 412 µL, 3.34 mmol, 3.1 equiv) and 2-hydroperoxy-2-methylpropane (388 mg, 784 µL, 4.31 mmol, 5.5 M, 4.0 equiv), and the solution was degassed by bubbling Ar under vigorous stirring for 15 minutes. Then, $Mn(dpm)_3$ (195 mg, 323 µmol, 0.3 equiv) was added in one portion, and the dark reaction mixture was degassed by bubbling Ar for another 5 min. The solution turned pale brown after 5 min, light yellow after 35 min. The mixture was stirred for 24 h and return of a dark brown color of the solution indicated reaction completion. Saturated aqueous $NaHCO_3$ solution and Na_2SO_5 were added, and the heterogeneous brown mixture was diluted with CH_2Cl_2. The phases were separated, and the aqueous layer was extracted with CH_2Cl_2. The combined organic layers were washed with brine, dried over Na_2SO_4, filtered and concentrated under reduced pressure. Purification by flash column chromatography (SiO_2, dry load on celite, eluting with 2.5% EtOAc in hexane) afforded the product (563 mg, 959 µmol, 89% yield) as a colorless oil.

(*E*)-**SI-6** (20.0 mg, 34.2 µmol, 1.00 equiv) was treated analogously, however, the reaction conditions proceeded sluggishly and the material was re-subjected to the hydrogenation conditions 3 times for full conversion. Final purification afforded the product (16.0 mg, 27.3 µmol, 80% yield)

Note: For ease of preparation, only the reaction employing (*Z*)-**SI-6** was scaled up to preparatively useful quantities.

^{1}H NMR (400 MHz, $CDCl_3$) δ = 7.67 – 7.60 (m, 4H), 7.46 – 7.33 (m, 6H), 6.50 – 6.44 (m, 2H), 6.31 (t, *J* = 2.2 Hz, 1H), 3.77 (s, 6H), 3.54 (t, *J* = 6.5 Hz, 2H), 2.35 (ddd, *J* = 12.9, 6.5, 2.6 Hz, 1H), 1.39 (q, *J* = 2.4, 2.0 Hz, 4H), 1.37 (s, 2H), 1.33 (s, 3H), 1.22 – 1.09 (m, 1H), 1.03 (d, *J* = 0.8 Hz, 9H) ppm.

^{13}C NMR (101 MHz, $CDCl_3$) δ = 160.7, 151.5, 135.7, 134.3, 130.4, 129.6, 127.7, 105.0, 97.3, 77.2, 63.9, 55.4, 52.6, 52.4, 40.4, 32.2, 29.6, 28.2, 27.0, 26.3, 25.7, 24.0, 19.3 ppm.

^{19}F NMR (377 MHz, $CDCl_3$) δ = –62.0 ppm.

IR (neat, ν_{max}/cm^{-1}) 2933, 1596, 1457, 1427, 1205, 1156, 1105, 1068, 1053, 824, 740, 701, 614, 504, 489.

ESI-HRMS m/z calcd for $C_{34}H_{45}F_3NaO_3Si$ $[M+Na]^+$ 609.2982; found 609.297

Synthesis of SI-8

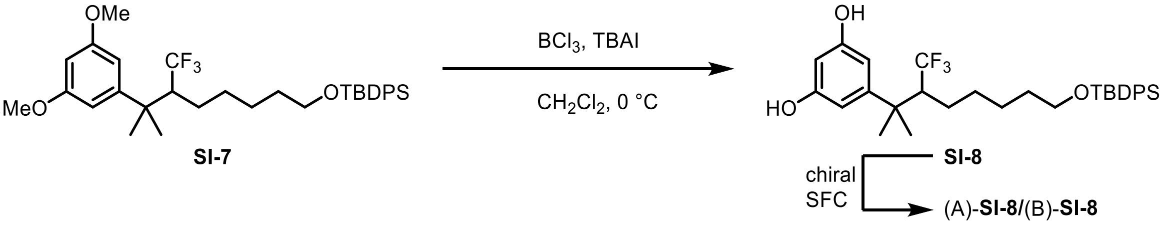

The starting material and TBAI were dried by azeotropic distillation two times with benzene. To a solution of **SI-7** (271 mg, 462 µmol, 1.00 equiv) and tetrabutylammonium iodide (426 mg, 1.15 mmol, 2.50 equiv) in CH_2Cl_2 (14.9 mL) at 0°C was dropwise added trichloro borane (1.00 M in CH_2Cl_2, 162 mg, 1.39 mL, 1.39 mmol, 3.00 equiv). The solution adopted a faint yellow color, and the color deepened over the course of the reaction. The reaction mixture was stirred for 1 h at 0 °C until full consumption of the starting material was observed. Saturated aqueous $NaHCO_3$ solution was carefully added to the reaction mixture, the phases were separated, and the aqueous layer was extracted with CH_2Cl_2. The combined organic layers were washed with brine, dried over Na_2SO_4, filtered, and concentrated under reduced pressure. Purification by flash column chromatography (SiO_2, eluting with 20 %–25% EtOAc in *n*-hexane) afforded the product (164 mg, 294 µmol, 64% yield) as a transparent liquid.

Combined material from two batches of the racemate (335 mg, 600 µmol) was resolved by chiral SFC Method 6 to afford enantiomers (A)-**SI-8** (107 mg, 191 µmol, 32%, %ee > 99%) and (B)-**SI-8** (115 mg, 206 µmol, 34%, %ee > 99%

^{1}H NMR (400 MHz, $CDCl_3$) δ = 7.69 – 7.62 (m, 4H), 7.46 – 7.33 (m, 6H), 6.35 (d, *J* = 2.2 Hz, 2H), 6.11 (t, *J* = 2.2 Hz, 1H), 4.65 (s, 2H), 3.55 (t, *J* = 6.5 Hz, 2H), 2.29 (tt, *J* = 10.2, 7.5 Hz, 1H), 1.59 (s, 1H), 1.51 – 1.40 (m, 1H), 1.37 (dd, *J* = 4.4, 2.3 Hz, 4H), 1.30 (s, 3H), 1.15 (h, *J* = 6.8 Hz, 2H), 1.04 (s, 12H) ppm.
^{13}C NMR (101 MHz, $CDCl_3$) δ = 156.6, 152.3, 135.8, 135.7, 134.3, 134.3, 129.7, 127.8, 106.1, 100.8, 77.2, 63.9, 52.5, 52.3, 52.1, 40.1, 32.1, 29.3, 28.1, 27.0, 26.1, 25.5, 23.9, 19.4 ppm.
^{19}F NMR (377 MHz, $CDCl_3$) δ = −62.1 ppm.
IR (neat, ν_{max}/cm^{-1}) 3341, 2934, 2860, 1601, 1472, 1429, 1330, 1251, 1147, 1111, 998, 823, 740, 702, 505.
ESI-HRMS m/z calcd for $C_{32}H_{41}F_3NaO_3S$ $[M+Na]^+$ 581.2669; found 581.2666

(A)-4 $[\alpha]^{25}_D$ = +8.257 ± 0.229 (c = 1.0, $CHCl_3$).

(B)-4 $[\alpha]^{25}_D$ = –8.556 ± 0.114 (c = 1.0, $CHCl_3$).

Synthesis of (B)/(S)-SI-9

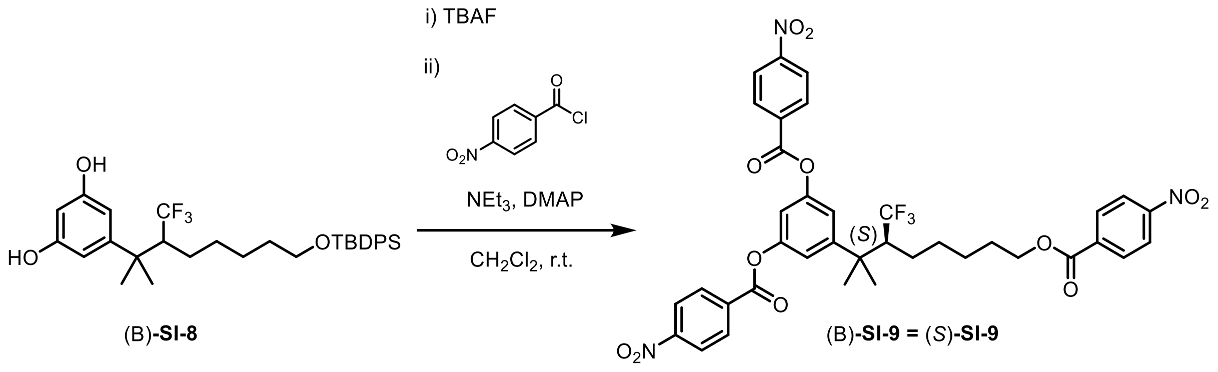

To a solution of (B)-**SI-8** (57.6 mg, 103 µmol, 1.00 equiv) in THF (1.0 mL) was added TBAF (309 µL, 309 µmol, 1 M in THF, 3.0 equiv) and the reaction was allowed to stir for 5 h. Then, sat. aq. NH_4Cl was added and the mixture was diluted with EtOAc. The phases were separated and the aqueous phase was extracted with EtOAc (3 x 5 mL). The combined organic layers were dried over Na_2SO_4, filtered, and concentrated under reduced pressure. The obtained brown residue was employed immediately in the subsequent reaction (quant. yield assumed).

To a solution of unpurified deprotection product, N,N-diethylethanamine (73.0 mg, 101 µL, 721 µmol, 7.0 equiv) and N,N-dimethylpyridin-4-amine (2.52 mg, 20.6 µmol, 0.2 equiv) in dry CH_2Cl_2 (1.51 mL) was added in one portion solid 4-nitrobenzoyl chloride (95.6 mg, 515 µmol, 5.0 equiv). The yellow reaction mixture was allowed to stir for 14 h before being loaded directly onto a silica column. Purification by flash column chromatography (SiO_2, 15 – 20% EtOAc in *n*-hexane) afforded the product (49.0 mg, 63.8 µmol, 62% yield) as an off-white solid.

Crystallization by slow evaporation (1 week) from 20% CH_2Cl_2 in MeOH afforded a mother liquor containing needle crystals suitable for X-Ray crystallographic studies (see appended data). Deposition number: CCDC2450722

^{1}H NMR (400 MHz, $CDCl_3$) δ 8.37 (s, 8H), 8.26 – 8.22 (m, 2H), 8.18 – 8.14 (m, 2H), 7.19 (d, J = 2.1 Hz, 2H), 7.10 (t, J = 2.0 Hz, 1H), 4.29 (t, J = 6.7 Hz, 2H), 2.47 – 2.33 (m, 1H), 1.68 (p, J = 7.0 Hz, 2H), 1.63 – 1.57 (m, 1H), 1.50 (bs, 3H), 1.44 (s, 3H), 1.36 (d, J = 7.2 Hz, 1H), 1.34 – 1.27 (m, 5H).

^{13}C NMR (101 MHz, $CDCl_3$) δ 164.83, 163.10, 152.17, 151.23, 151.01, 150.61, 135.94, 134.58, 131.49, 130.77, 128.64 (d, J = 283.4 Hz), 123.98, 123.63, 117.57, 113.49, 65.91, 52.75 (d, J = 22.7 Hz), 40.63, 29.85, 28.25, 27.92, 26.32, 25.86, 23.97.

^{19}F NMR (377 MHz, $CDCl_3$) δ –62.22.

IR (neat, ν_{max}/cm^{-1}) 3112, 2924, 2854, 1746, 1723, 1608, 1528, 1466, 1433, 1410, 1349, 1320, 1257, 1128, 1104, 1078, 1014, 962, 872, 856, 839, 779, 715.

ESI-HRMS m/z calcd for $C_{37}H_{32}F_3N_3NaO_{12}$ $[M+Na]^+$ 790.1830; found 790.1835

$[\alpha]^{25}_D$ = –2.964 ± 0.392 (c = 0.89, $CHCl_3$).

Independent Synthesis of (+)-(*R*)-4/(A)-4/(A)-SI-10

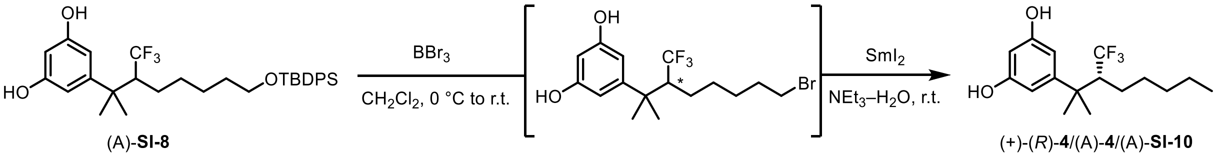

To a solution of (A)-**SI-8** (22.3 mg, 39.9 µmol, 1.0 equiv) in CH_2Cl_2 (2.70 mL) at 0 °C was dropwise added BBr_3 (132 µL, 132 µmol, 1 M in CH_2Cl_2, 3.3 equiv), and the resulting light orange solution was allowed to stir at ambient temperature for 1 h. Then, sat. aq. $NaHCO_3$ was added, the phases were separated and the aqueous phase was extracted with CH_2Cl_2. The combined organic layers were dried over Na_2SO_4, filtered and concentrated under reduced pressure. Filtration over a plug of silica (30% EtOAc in *n*-hexane) afforded the unpurified alkyl bromide as a pink gum and was employed immediately in the subsequent reaction (quant. yield assumed).

Note: Due to observed instability of the compound upon prolonged exposure to silica and/or air, the mateiral was directly employed in the next step.

To a solution of the unpurified alkyl bromide in dry THF (0.2 ml) was added NEt_3 (156 µL, 1.12 mmol, 28.0 equiv) and water (25.2 mg, 25.2 µL, 1.40 mmol, 35.0 equiv) and the mixture was thoroughly degassed via freeze-pump-thaw procedure (3x), and back-filled with argon, before a solution of SmI_2 (2.79 mL, 279 µmol, 0.100M, 7.00 equiv) was added dropwise. The ink-blue solution was allowed to stir for 12 h before diluting the reaction mixture with 1M aq. HCl and EtOAc. The phases were separated and the aqueous phase was extracted with EtOAc. The combined organic layers were washed with brine, dried over Na_2SO_4, filtered, and concentrated under reduced pressure. Purification by flash column chromatography (SiO_2, 20 – 25% EtOAc in hexane) afforded the product (6.40 mg, 21.0 µmol, 53% yield) as a colorless gum.

Note: The product (A)-**SI-10** ^{1}H NMR spectrum (shifts and coupling constants) as well as its $[\alpha]^{25}_D$ (value and sign) matched the data of seperately accessed/authentic (A)-**4**, hence allwoing for the unambiguous stereochemical assignement of (A)-**4** as (+)-(*R*)-**4**. Slight deviations are attributed to concentration effects, as data on (A)-**SI-10** was obtained with less material.

^{1}H NMR (400 MHz, $CDCl_3$) δ = 6.39 (d, J = 2.2 Hz, 2H), 6.20 (t, J = 2.2 Hz, 1H), 4.73 (bs, 2H, phenol OHs), 2.38 – 2.25 (m, 1H), 1.51 – 0.97 (m, 8H) 1.37 (q, J = 2.0 Hz, 3H), 1.31 (s, 3H), , 0.79 (t, J = 7.1 Hz, 3H).

$[\alpha]^{25}_D$ = +11.864 ± 0.429 (c = 0.58, $CHCl_3$).

<u>Comparison to authentic (A)-**4**:</u>

^{1}H NMR (400 MHz, $CDCl_3$) δ = 6.42 (d, J = 2.2 Hz, 2H), 6.23 (t, J = 2.2 Hz, 1H), 5.51 (bs, 2H, phenol OHs), 2.30 (qdd, J = 10.2, 7.5, 2.6 Hz, 1H), 1.51 – 0.96 (m, 8H), 1.34 (q, J = 1.9 Hz, 3H), 1.28 (s, 3H), 0.79 (t, J = 7.1 Hz, 3H).

(*R*)-4 $[\alpha]^{25}_D$ = +17.570 ± 0.110 (c = 1.0, $CHCl_3$).

Synthesis of (*R*)-SI-13

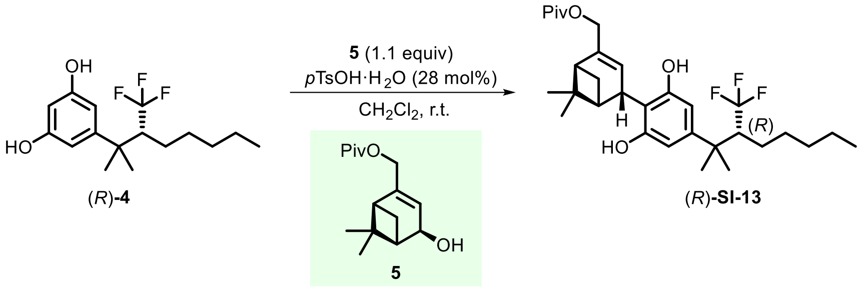

To a solution of (+)-(*R*)-**4** (17.9 mg, 58.8 µmol, 1.0 equiv) and **5** (16.3 mg, 64.7 µmol, 1.1 equiv) in CH_2Cl_2 (2.3 mL) was added *p*TsOH·H_2O (3.1 mg. 16.4 µmol, 0.28 equiv) and the solution was stirred for 30 min. The reaction was stopped by addition on sat. aq. $NaHCO_3$ (4 mL) and diluted with Et_2O (5 mL). The layers were separated and the aqueous phase was extracted with Et_2O (3 × 5 mL). Combined organic extracts were dried over $MgSO_4$, filtered and concentrated under reduced pressure. Purification by flash column chromatography (SiO_2; using 0 – 15% EtOAc in hexanes) afforded the product as a colourless foam (23.3 mg, 74%).

Note: Allylic alcohol **5** was prepared according to our previously published procedure.[27]

^{1}H NMR (500 MHz, $CDCl_3$) δ 6.34 (s, 2H), 5.99 (dt, J = 3.0, 1.5 Hz, 1H), 5.68 (bs, 2H), 4.64 (ddd, J = 13.5, 2.1, 1.4 Hz, 1H), 4.51 (ddd, J = 13.6, 2.4, 1.6 Hz, 1H), 4.04 – 3.97 (m, 1H), 2.36 (dt, J = 9.7, 5.6 Hz, 1H), 2.33 – 2.22 (m, 3H), 1.47 (d, J = 9.7 Hz, 1H), 1.45 – 1.38 (m, 1H), 1.35 (s, 3H), 1.34 (s, 3H), 1.32 – 1.20 (m, 2H), 1.28 (s, 3H), 1.23 (s, 9H), 1.14 – 1.00 (m, 5H), 0.98 (s, 3H), 0.76 (t, J = 7.1 Hz, 3H).

^{13}C NMR (126 MHz, $CDCl_3$) δ 178.7, 155.2, 149.8, 149.3, 129.1 (q, J = 283.3 Hz), 120.1, 112.5, 106.7, 66.6, 52.4 (q, J = 22.1 Hz), 47.5, 44.2, 41.1, 39.8, 39.1, 37.9, 31.6, 29.4, 28.1, 28.1, 27.4, 26.1, 26.0, 23.6, 22.2, 20.9, 14.0.

^{19}F NMR (471 MHz, $CDCl_3$) δ -62.2.

IR (neat, ν_{max}/cm^{-1}): 3445, 2942, 2872, 1707, 1626, 1578, 1481, 1254, 1149.

HRMS (ESI): *m/z* = 561.3147 $[M+Na]^+$ (calc. for $C_{31}H_{45}F_3NaO_4$ *m/z* = 561.3162).

$[\alpha]^{25}_D$ = +62.961 ± 0.119 (c = 1.0, $CHCl_3$).

Synthesis of (*S*)-SI-13

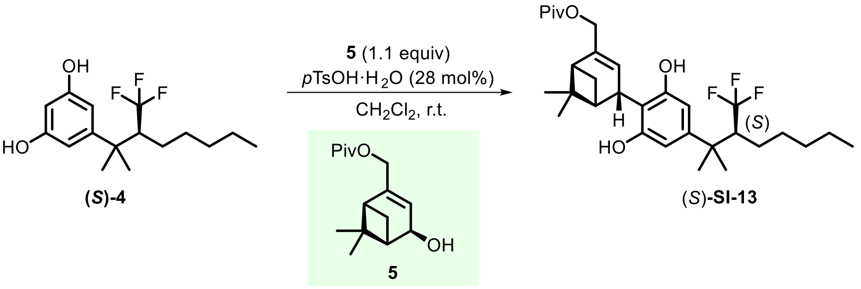

To a solution of (–)-(*S*)-**4** (16.6 mg, 54.5 µmol, 1.0 equiv) and **5** (15.1 mg, 60.0 µmol, 1.1 equiv) in CH_2Cl_2 (2.3 mL) was added *p*TsOH·H_2O (2.9 mg. 15.2 µmol, 0.28 equiv) and the solution was stirred for 30 min. The reaction was stopped by addition on sat. aq. $NaHCO_3$ (4 mL) and diluted with Et_2O (5 mL). The layers were separated and the aqueous phase was extracted with Et_2O (3 × 5 mL). Combined organic extracts were dried over $MgSO_4$, filtered and concentrated under reduced pressure. Purification by flash column chromatography (SiO_2; using 0 – 15% EtOAc in hexanes) afforded the product as a faint yellow foam (23.0 mg, 78%).

Note: Allylic alcohol **5** was prepared according to our previously published procedure.[27]

^{1}H NMR (500 MHz, $CDCl_3$) δ 6.34 (s, 2H), 6.00 (dt, J = 3.1, 1.6 Hz, 1H), 5.76 (bs, 2H), 4.64 (ddd, J = 13.5, 2.0, 1.4 Hz, 1H), 4.51 (ddd, J = 13.5, 2.3, 1.5 Hz, 1H), 4.02 – 3.98 (m, 1H), 2.36 (dt, J = 9.7, 5.6 Hz, 1H), 2.33 – 2.22 (m, 3H), 1.46 (d, J = 9.7 Hz, 1H), 1.45 – 1.38 (m, 1H), 1.35 (s, 3H), 1.34 (s, 3H), 1.33 – 1.19 (m, 2H), 1.28 (s, 3H), 1.23 (s, 9H), 1.16 – 0.96 (m, 5H), 0.98 (s, 3H), 0.76 (t, J = 7.1 Hz, 3H).

^{13}C NMR (126 MHz, $CDCl_3$) δ 178.7, 155.2, 149.8, 149.3, 129.1 (q, J = 283.5 Hz), 120.1, 112.4, 106.7, 66.6, 52.5 (q, J = 22.2 Hz), 47.5, 44.2, 41.1, 39.8, 39.1, 37.9, 31.6, 29.4, 28.1, 28.1, 27.4, 26.1, 26.0, 23.6, 22.2, 20.9, 14.0.

^{19}F NMR (471 MHz, $CDCl_3$) δ -62.2.

IR (neat, ν_{max}/cm^{-1}): 3446, 2942, 2872, 1707, 1626, 1578, 1481, 1254, 1149.

HRMS (ESI): *m/z* = 561.3154 $[M+Na]^+$ (calc. for $C_{31}H_{45}F_3NaO_4$ *m/z* = 561.3162).

$[\alpha]^{25}_D$ = +38.128 ± 0.330 (c = 1.0, $CHCl_3$).

Synthesis of (*R*)-SI-14

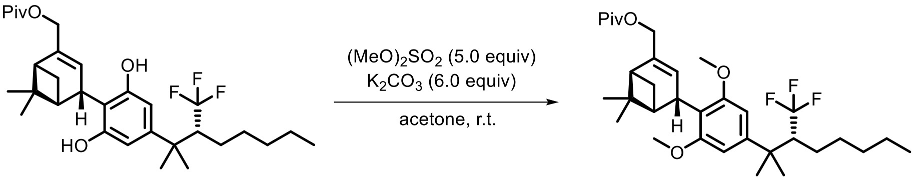

(*R*)-**SI-13** (*R*)-**SI-14**

To a solution of (*R*)-**SI-13** (23.3 mg, 43.2 µmol, 1.0 equiv) in acetone (0.5 mL) was added $(MeO)_2SO_2$ (20.5 µL. 0.216 mmol, 5.0 equiv) and K_2CO_3 (35.9 mg. 0.260 mmol, 6.0 equiv) and the light purple suspension was stirred overnight at rt. The reaction mixture was filtered and concentrated under reduced pressure. Purification by flash column chromatography (SiO_2; using 0 – 10% Et_2O in hexanes) afforded the product as a light red wax (23.1 mg, 94%).

^{1}H NMR (500 MHz, $CDCl_3$) δ 6.47 (s, 2H), 5.76 (dt, J = 2.9, 1.5 Hz, 1H), 4.61 – 4.54 (m, 1H), 4.51 (ddd, J = 12.2, 1.7, 1.2 Hz, 1H), 4.04 – 3.97 (m, 1H), 3.74 (s, 6H), 2.31 (dddd, J = 20.6, 10.3, 7.4, 2.5 Hz, 1H), 2.20 – 2.10 (m, 2H), 2.02 (tt, J = 5.8, 1.9 Hz, 1H), 1.73 – 1.66 (m, 1H), 1.50 – 1.41 (m, 1H), 1.43 (s, 3H), 1.36 (s, 3H), 1.34 – 1.18 (m, 2H), 1.29 (s, 3H), 1.21 (s, 9H), 1.12 – 0.88 (m, 5H), 0.97 (s, 3H), 0.74 (t, J = 7.1 Hz, 3H).

^{13}C NMR (126 MHz, $CDCl_3$) δ 178.6, 158.6, 148.1, 137.5, 129.1 (q, J = 283.3 Hz), 126.1, 118.7, 103.1, 67.6, 56.0, 52.7 (q, J = 22.1 Hz), 47.6, 44.0, 41.1, 40.5, 39.0, 37.7, 31.5, 30.5, 29.4, 28.8, 27.6, 27.4, 26.4, 26.2, 22.2, 21.2, 14.0.

^{19}F NMR (471 MHz, $CDCl_3$) δ -62.1.

IR (neat, ν_{max}/cm^{-1}): 2957, 2932, 2870, 1727, 1605, 1575, 1461, 1412, 1150.

HRMS (ESI): m/z = 589.3470 $[M+Na]^+$ (calc. for $C_{33}H_{49}F_3NaO_4$ m/z = 589.3475).

$[\alpha]^{25}_D$ = +73.052 ± 0.280 (c = 1.0, $CHCl_3$).

Synthesis of (*S*)-SI-14

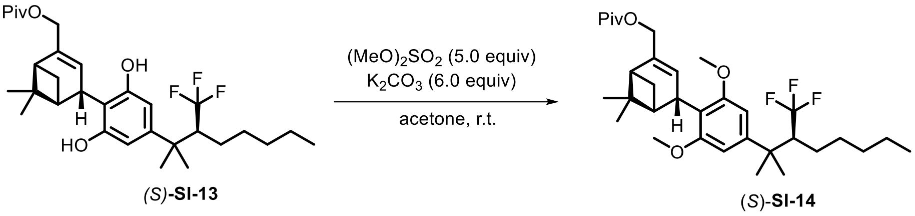

To a solution of (*S*)-**SI-13** (23.0 mg, 42.7 µmol, 1.0 equiv) in acetone (0.5 mL) was added $(MeO)_2SO_2$ (20.2 µL. 0.214 mmol, 5.0 equiv) and K_2CO_3 (35.4 mg. 0.256 mmol, 6.0 equiv) and the light purple suspension was stirred overnight at rt. The reaction mixture was filtered and concentrated under reduced pressure. Purification by flash column chromatography (SiO_2; using 0 – 10% Et_2O in hexanes) afforded the product as a light red wax (23.5 mg, 97%).

^{1}H NMR (500 MHz, $CDCl_3$) δ 6.47 (s, 2H), 5.88 – 5.64 (m, 1H), 4.61 – 4.55 (m, 1H), 4.51 (ddd, J = 12.2, 1.7, 1.3 Hz, 1H), 4.02 – 3.95 (m, 1H), 3.74 (s, 6H), 2.31 (dddd, J = 20.4, 10.1, 7.2, 2.5 Hz, 1H), 2.19 – 2.11 (m, 2H), 2.06 – 1.96 (m, 1H), 1.73 – 1.64 (m, 1H), 1.49 – 1.41 (m, 1H), 1.42 (s, 3H), 1.36 (s, 3H), 1.35 – 1.19 (m, 2H), 1.29 (s, 3H), 1.21 (s, 9H), 1.12 – 0.86 (m, 5H), 0.97 (s, 3H), 0.74 (t, J = 7.1 Hz, 3H).

^{13}C NMR (126 MHz, $CDCl_3$) δ 178.6, 158.6, 148.1, 137.5, 129.1 (q, J = 283.3 Hz), 126.2, 118.7, 103.1, 67.6, 56.0, 52.7 (q, J = 22.1 Hz), 47.5, 43.9, 41.0, 40.5, 39.0, 37.7, 30.5, 29.4, 28.8, 27.6, 27.4, 26.4, 26.2, 23.3, 22.1, 21.2, 14.0.

^{19}F NMR (471 MHz, $CDCl_3$) δ -62.1.

IR (neat, ν_{max}/cm^{-1}): 2956, 2932, 2870, 1727, 1605, 1575, 1461, 1412, 1149.

HRMS (ESI): m/z = 589.3460 $[M+Na]^+$ (calc. for $C_{33}H_{49}F_3NaO_4$ m/z = 589.3475).

$[\alpha]^{25}_D$ = +58.259 ± 0.119 (c = 1.0, $CHCl_3$).

Synthesis of (*R*)-1

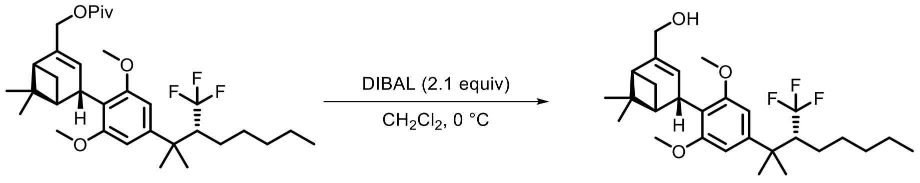

(*R*)-**SI-14** (*R*)-**1**

Solution of (*R*)-**SI-14** (26.1 mg, 46.0 µmol, 1.0 equiv) in CH_2Cl_2 (0.4 mL) was cooled to 0 °C and DIBAL (1.0 M in hexanes, 97 µL. 96.7 µmol, 2.1 equiv) was added dropwise. The mixture was stirred at 0 °C for 15 min. and subsequently quenched with sat. aq. Rochelle's salt (4 mL) and diluted with Et_2O (5 mL). The mixture was vigorously stirred at rt until the phases cleanly separated. The phases were separated and the aqueous phase extracted with Et_2O (2 × 5 mL). Combined organic fractions were dried over $MgSO_4$, filtered and concentrated under reduced pressure. Purification by flash column chromatography (SiO_2; using 5 – 20% EtOAc in hexanes) afforded the product as a clear oil (18.3 mg, 82%).

^{1}H NMR (500 MHz, $CDCl_3$) δ 6.47 (s, 2H), 5.69 (dt, J = 2.8, 1.4 Hz, 1H), 4.07 (ddd, J = 1.9, 1.3, 0.6 Hz, 2H), 4.03 – 3.98 (m, 1H), 3.75 (s, 6H), 2.31 (qdd, J = 10.3, 7.4, 2.6 Hz, 1H), 2.25 – 2.16 (m, 2H), 2.04 (tt, J = 5.8, 1.9 Hz, 1H), 1.69 (d, J = 8.3 Hz, 1H), 1.50 – 1.41 (m, 1H), 1.43 (s, 3H), 1.36 (s, 3H), 1.34 – 1.16 (m, 2H), 1.31 (s, 3H), 1.12 – 0.89 (m, 5H), 0.97 (s, 3H), 0.74 (t, J = 7.1 Hz, 3H).

^{13}C NMR (126 MHz, $CDCl_3$) δ 158.6, 148.1, 142.2, 129.1 (q, J = 283.4 Hz), 123.6, 118.8, 103.2, 66.8, 56.1, 52.7 (q, J = 22.2 Hz), 47.6, 44.0, 41.0, 40.5, 37.6, 31.6, 29.4, 28.8, 28.0, 26.4, 26.3, 23.3, 22.2, 21.2, 14.0.

^{19}F NMR (471 MHz, $CDCl_3$) δ -62.1.

IR (neat, ν_{max}/cm^{-1}): 3373, 2929, 2866, 1605, 1574, 1460, 1412, 1239, 1119.

HRMS (ESI): m/z = 505.2903 $[M+Na]^+$ (calc. for $C_{28}H_{41}F_3NaO_3$ m/z = 505.2900).

$[\alpha]^{25}_D$ = +97.242 ± 0.182 (c = 1.0, $CHCl_3$).

Synthesis of (*S*)-1

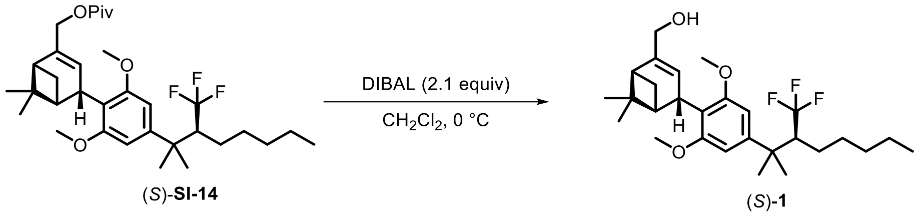

Solution of (S)-**SI-14** (24.0 mg, 42.3 µmol, 1.0 equiv) in CH_2Cl_2 (0.4 mL) was cooled to 0 °C and DIBAL (1.0 M in hexanes, 89 µL. 88.9 µmol, 2.1 equiv) was added dropwise. The mixture was stirred at 0 °C for 15 min. and subsequently quenched with sat. aq. Rochelle's salt (4 mL) and diluted with Et_2O (5 mL). The mixture was vigorously stirred at rt until the phases cleanly separated. The phases were separated and the aqueous phase extracted with Et_2O (2 × 5 mL). Combined organic fractions were dried over $MgSO_4$, filtered and concentrated under reduced pressure. Purification by flash column chromatography (SiO_2; using 5 – 20% EtOAc in hexanes) afforded the product as a clear oil (16.6 mg, 81%).

^{1}H NMR (400 MHz, $CDCl_3$) δ 6.47 (s, 2H), 5.69 (dt, J = 2.8, 1.4 Hz, 1H), 4.11 – 4.04 (m, 2H), 4.04 – 3.96 (m, 1H), 3.75 (s, 6H), 2.32 (qdd, J = 10.2, 7.4, 2.5 Hz, 1H), 2.25 – 2.16 (m, 2H), 2.04 (tt, J = 5.9, 1.9 Hz, 1H), 1.68 (d, J = 8.2 Hz, 1H), 1.51 – 1.45 (m, 1H), 1.43 (s, 3H), 1.36 (s, 3H), 1.34 – 1.16 (m, 2H), 1.31 (s, 3H), 1.12 – 0.87 (m, 5H), 0.97 (s, 3H), 0.74 (t, J = 7.0 Hz, 3H).

^{13}C NMR (101 MHz, $CDCl_3$) δ 158.7, 148.1, 142.2, 129.1 (d, J = 283.3 Hz), 123.6, 118.8, 103.2, 66.8, 56.0, 52.7 (q, J = 22.0 Hz), 47.6, 44.0, 41.0, 40.5, 37.6, 31.6, 29.4, 28.8, 28.0, 26.4, 26.2, 23.3, 22.2, 21.2, 14.0.

^{19}F NMR (376 MHz, $CDCl_3$) δ -62.1.

IR (neat, ν_{max}/cm^{-1}): 3371, 2929, 2866, 1605, 1574, 1459, 1412, 1239, 1119.

HRMS (ESI): m/z = 505.2902 $[M+Na]^+$ (calc. for $C_{28}H_{41}F_3NaO_3$ m/z = 505.2900).

$[\alpha]^{25}_D$ = +78.198 ± 0.132 (c = 1.0, $CHCl_3$).

Synthesis of 7

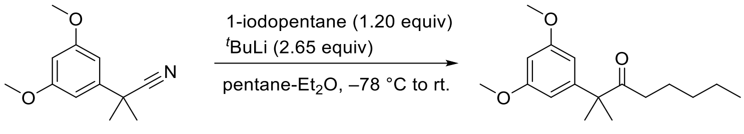

A solution of 1-iodopentane (3.82 mL, 29.2 mmol, 1.20 equiv) in 3:2 pentane-Et_2O (300 mL) was cooled to –78 °C under argon atmosphere and *t*-BuLi (1.7 M in pentane, 38.0 mL, 64.6 mmol, 2.65 equiv) was added dropwise. The solution was stirred at –78 °C for 5 min following addition, and then the mixture was allowed to reach rt. and was left standing for 1 h. The reaction mixture was cooled back to –78 °C, and a solution of **2** (5.00 g, 24.4 mmol, 1.00 equiv) in pentane (10 mL) was added in one portion. The mixture was stirred for 5 min. at –78 °C, allowed to warm up to rt. and stirred for 1.5 h. The reaction was quenched by addition of aq. HCl (0.5 M, 50 mL). The organic solvents were removed under reduced pressure*,* THF (150 mL) and aq. HCl (0.5 M, 100 mL) were added and the mixture stirred vigorously overnight at rt. The mixture was diluted with EtOAc (150 mL), layers were separated, and the aqueous phase was extracted with EtOAc (2 × 150 mL). Combined organic extracts were washed with 5% aq. $Na_2S_2O_3$ solution (150 mL), followed by brine, dried over $MgSO_4$ and concentrated under reduced pressure. Purification by flash column chromatography (SiO_2; using 0 – 15% Et_2O in hexanes) afforded the product as a light-yellow liquid (6.10 g, 90%).

^{1}H NMR (400 MHz, $CDCl_3$) δ 6.36 (d, J = 2.2 Hz, 2H), 6.33 (t, J = 2.2 Hz, 1H), 3.75 (s, 6H), 2.20 (t, J = 7.4 Hz, 2H), 1.50 – 1.43 (m, 2H), 1.42 (s, 6H), 1.23 – 1.02 (m, 4H), 0.79 (t, J = 7.2 Hz, 3H).

^{13}C NMR (101 MHz, $CDCl_3$) δ 213.0, 161.1, 146.7, 104.6, 98.3, 55.3, 52.4, 37.2, 31.4, 25.1, 24.1, 22.5, 13.9.

IR (neat, ν_{max}/cm^{-1}): 2956, 2932, 2872, 1708, 1594, 1456, 1423, 1205, 1155.

HRMS (ESI): *m/z* = 279.1955 $[M+H]^+$ (calc. for $C_{17}H_{27}O_3$ *m/z* = 279.1955).

Synthesis of 8a

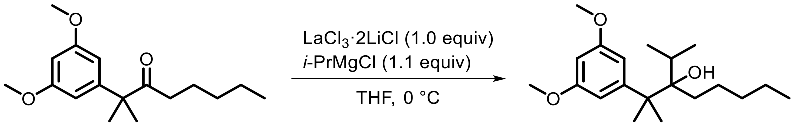

7 8a

To a solution of $LaCl_3$·2LiCl (0.6 M in THF, 6.00 mL, 3.59 mmol, 1.0 equiv) was added **7** (1.00 g, 3.59 mmol, 1.0 equiv) in dry THF (3.3 mL) and the resulting mixture was stirred at rt. for 1 h. The reaction mixture was cooled to 0 ºC and a solution of *i*-PrMgCl (1.4 M in THF, 2.82 mL, 3.95 mmol, 1.1 equiv) was added dropwise and the mixture was allowed to stir at the same temperature for 2.5 h. The mixture was quenched with sat. NH_4Cl solution (10 mL) and H_2O (10 mL) and diluted with Et_2O (10 mL). The layers were separated and the aqueous phase was extracted with Et_2O (3 × 10 mL). Combined organic extracts were dried with $MgSO_4$, filtered and concentrated under reduced pressure. The residue was purified by flash column chromatography (SiO_2; 0 – 15% EtOAc in hexanes) to afford the product as a colourless oil (820 mg, 71%).

^{1}H NMR (400 MHz, $CDCl_3$) δ 6.65 (d, J = 2.3 Hz, 2H), 6.34 (t, J = 2.2 Hz, 1H), 3.78 (s, 6H), 2.07 (hept, *J* = 7.0 Hz, 1H), 1.62 (ddd, J = 13.9, 11.2, 4.9 Hz, 1H), 1.50 – 1.38 (m, 1H), 1.43 (s, 1H), 1.41 (s, 3H), 1.40 (s, 3H), 1.35 – 1.12 (m, 6H), 0.90 – 0.79 (m, 9H).

^{13}C NMR (101 MHz, $CDCl_3$) δ 160.2, 150.5, 107.2, 97.4, 78.4, 55.3, 47.9, 33.7, 33.4, 33.2, 26.1, 25.3, 24.7, 22.8, 20.5, 20.2, 14.2.

IR (neat, ν_{max}/cm^{-1}): 3573, 2955, 1594, 1457, 1421, 1204, 1155, 1052.

HRMS (ESI): *m/z* = 345.2398 $[M+Na]^+$ (calc. for $C_{20}H_{34}NaO_3$ *m/z* = 345.2400).

Synthesis of 8b

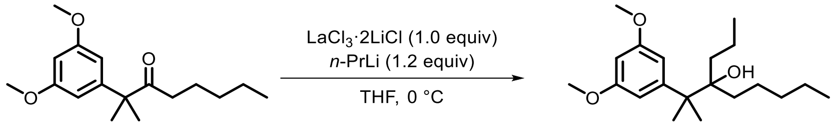

Preparation of *n*-PrLi:

To a solution of 1-iodopropane (414 µL, 4.25 mmol, 1.20 equiv) in dry 3:2 pentane-Et_2O (42 mL) at –78 °C was added *t*-BuLi (1.7 M in pentane, 5.52 mL, 9.38 mmol, 2.65 equiv) dropwise. The solution was stirred at –78 °C for 5 min following addition, and then the mixture was allowed to reach rt and was left standing for 1 h.

To a solution of $LaCl_3$·2LiCl (0.6 M in THF, 5.90 mL, 3.54 mmol, 1.00 equiv) was added **7** (985 mg, 3.54 mmol, 1.00 equiv) in dry THF (3 mL) and the resulting mixture was stirred at rt for 1 h. The reaction mixture was cooled to 0 ºC and the previously prepared solution of *n*-PrLi was added dropwise. The mixture was stirred for 30 min. at 0 °C and then was quenched with sat. NH_4Cl solution (15 mL) and H_2O (15 mL). The mixture was diluted with Et_2O (15 mL), the phases were separated and the aqueous phase was extracted with Et_2O (3 × 15 mL). Combined organic extracts were dried with $MgSO_4$, filtered and concentrated under reduced pressure. The residue was purified by flash column chromatography (SiO_2; 0 – 15% EtOAc in hexanes) to afford the product as a colourless oil (1.13 g, 99%).

^{1}H NMR (400 MHz, $CDCl_3$) δ 6.62 (d, J = 2.2 Hz, 2H), 6.35 (t, J = 2.2 Hz, 1H), 3.79 (s, 6H), 1.56 – 1.40 (m, 4H), 1.37 (s, 6H), 1.31 – 1.24 (m, 8H), 1.24 (s, 1H), 0.85 (t, J = 7.1 Hz, 3H), 0.84 (t, J = 7.2 Hz, 3H).

^{13}C NMR (101 MHz, $CDCl_3$) δ 160.2, 149.8, 107.1, 97.5, 77.1, 55.4, 46.8, 38.1, 35.6, 33.1, 24.9, 24.6, 22.8, 18.2, 15.2, 14.2.

IR (neat, ν_{max}/cm^{-1}): 3572, 2957, 2871, 1595, 1457, 1421, 1205, 1156, 1054.

HRMS (ESI): *m/z* = 245.2405 $[M+Na]^+$ (calc. for $C_{20}H_{34}NaO_3$ *m/z* = 345.2400).

Synthesis of 8c

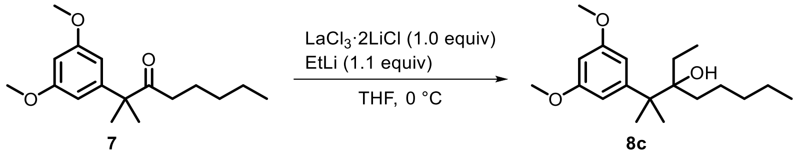

To a solution of $LaCl_3{\cdot}2LiCl$ (0.6 M in THF, 5.57 mL, 3.34 mmol, 1.0 equiv) was added **7** (930 mg, 3.34 mmol, 1.0 equiv) in dry THF (3.0 mL) and the resulting mixture was stirred at rt for 1 h. The reaction mixture was cooled to 0 ºC and a solution of EtLi (0.3 M in benzene:cyclohexane, 12.2 mL, 3.68 mmol, 1.1 equiv) was added dropwise and the mixture was allowed to stir at the same temperature for 1.5 h. The mixture was quenched with sat. NH_4Cl solution (10 mL) and H_2O (10 mL) and diluted with Et_2O (10 mL). The layers were separated and the aqueous phase was extracted with Et_2O (3 × 10 mL). The combined organic extracts were dried with $MgSO_4$, filtered and concentrated under reduced pressure. The residue was purified by flash column chromatography (SiO_2; 0 – 15% EtOAc in hexanes) to afford the product as a colourless oil (932 mg, 99%).

^{1}H NMR (400 MHz, $CDCl_3$) δ 6.62 (d, J = 2.3 Hz, 2H), 6.34 (t, J = 2.2 Hz, 1H), 3.76 (s, 6H), 1.60 – 1.41 (m, 4H), 1.38 (s, 6H), 1.30 (s, 1H), 1.29 – 1.15 (m, 6H), 0.85 (t, J = 6.8 Hz, 3H), 0.81 (t, J = 7.4 Hz, 3H).

^{13}C NMR (101 MHz, $CDCl_3$) δ 160.1, 149.7, 107.0, 97.3, 77.0, 55.1, 46.7, 34.9, 33.0, 27.8, 24.8, 24.8, 24.5, 22.6, 14.1, 9.2.

IR (neat, ν_{max}/cm^{-1}): 3568, 2954, 2872, 1594, 1456, 1421, 1204, 1154, 1021.

HRMS (ESI): m/z = 331.2244 $[M+Na]^+$ (calc. for $C_{19}H_{32}NaO_3$ m/z = 331.2244).

Synthesis of 8d

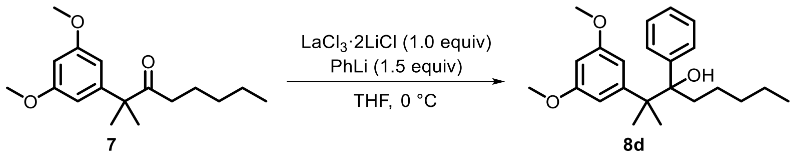

To a solution of $LaCl_3 \cdot 2LiCl$ (0.6 M in THF, 6.29 mL, 3.77 mmol, 1.0 equiv) was added **7** (1.05 g, 3.77 mmol, 1.0 equiv) in dry THF (3.0 mL) and the resulting mixture was stirred at rt. for 1 h. The reaction mixture was cooled to 0 ºC and PhLi (1.8 M in dibutyl ether, 3.14 mL, 5.66 mmol, 1.5 equiv) was added dropwise. The mixture was stirred for 30 min. at 0 °C and then was quenched with aq. sat. NH_4Cl solution (15 mL) and H_2O (15 mL). The aqueous phase was diluted with Et_2O (20 mL), the phases were separated and the aqueous phase extracted with Et_2O (3 × 30 mL). Combined organic extracts were dried with $MgSO_4$, filtered and concentrated under reduced pressure. The residue was purified by flash column chromatography (SiO_2; 0 – 15% EtOAc in hexanes) to afford the product as a light yellow oil (1.33 g, 99%).

^{1}H NMR (400 MHz, $CDCl_3$) δ 7.30 – 7.20 (m, 5H), 6.46 (d, J = 2.3 Hz, 2H), 6.37 (t, J = 2.2 Hz, 1H), 3.75 (s, 6H), 2.21 (ddd, J = 14.0, 11.6, 4.2 Hz, 1H), 1.81 (s, 1H), 1.60 (ddd, J = 13.0, 11.6, 3.6 Hz, 1H), 1.39 (s, 3H), 1.27 (s, 3H), 1.24 – 1.09 (m, 4H), 0.94 – 0.84 (m, 2H), 0.83 – 0.77 (m, 3H).

^{13}C NMR (101 MHz, $CDCl_3$) δ 160.0, 148.4, 142.5, 128.0, 127.0, 126.4, 107.5, 98.0, 80.5, 55.3, 46.7, 35.7, 32.6, 25.1, 24.8, 23.8, 22.8, 14.2.

IR (neat, ν_{max}/cm^{-1}): 3552, 2954, 2870, 1595, 1457, 1205, 1155.

HRMS (ESI): *m/z* = 379.2241 $[M+Na]^+$ (calc. for $C_{23}H_{32}NaO_3$ *m/z* = 379.2244).

Synthesis of 8e

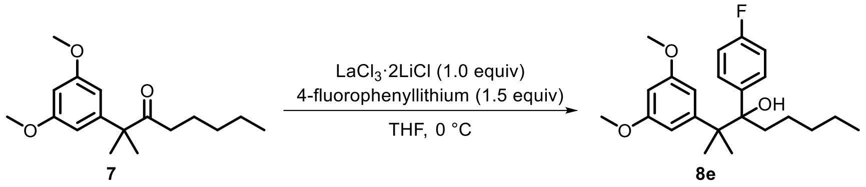

Preparation of 4-fluorophenyllithium:

To a solution of 4-fluoroiodobenzene (853 µL, 7.40 mmol, 2.0 equiv) in dry Et_2O (8.0 mL) was added at –78 ºC *n*-BuLi (1.6 M in hexane, 3.47 mL, 5.55 mmol, 1.5 equiv) dropwise and the solution was stirred at –78 °C for 30 min.

To a solution of $LaCl_3$·2LiCl (0.6 M in THF, 6.17 mL, 3.70 mmol, 1.0 equiv) was added **7** (1.03 g, 3.70 mmol, 1.0 equiv) in dry THF (3.0 mL) and the resulting mixture was stirred at rt. for 1 h. The reaction mixture was cooled to 0 ºC and the previously prepared solution of 4-fluorophenyllithium was added dropwise. The mixture was stirred for 30 min. at 0 °C and then was quenched with aq. sat. NH_4Cl solution (15 mL) and H_2O (15 mL). The aqueous phase was diluted with Et_2O (15 mL), the phases were separated and the aqueous phase extracted with Et_2O (3 × 15 mL). Combined organic extracts were dried with $MgSO_4$, filtered and concentrated under reduced pressure. The residue was purified by flash column chromatography (SiO_2; 0 – 15% EtOAc in hexanes) to afford the product as a colourless oil (1.36 g, 98%).

^{1}H NMR (400 MHz, $CDCl_3$) δ 7.19 (bs, 2H), 6.94 (t, *J* = 8.8 Hz, 2H), 6.44 (d, J = 2.0 Hz, 2H), 6.36 (t, *J* = 2.2 Hz, 1H), 3.75 (s, 6H), 2.21 – 2.09 (m, 1H), 1.79 (s, 1H), 1.58 (ddd, *J* = 15.1, 11.7, 3.5 Hz, 1H), 1.35 (s, 3H), 1.24 (s, 3H), 1.21 – 1.04 (m, 4H), 0.92 – 0.74 (m, 2H), 0.79 (t, *J* = 6.9 Hz, 3H).

^{13}C NMR (101 MHz, $CDCl_3$) δ 161.64 (d, J = 244.7 Hz), 160.1, 148.2, 138.14 (d, J = 3.1 Hz), 129.54 (d, J = 7.8 Hz), 113.69 (d, J = 20.9 Hz), 107.5, 97.9, 80.3, 55.3, 46.7, 35.8, 32.5, 24.9, 24.8, 23.8, 22.7, 14.1.

^{19}F NMR (377 MHz, $CDCl_3$) δ -117.8.

IR (neat, ν_{max}/cm^{-1}): 3559, 2955, 2872, 1595, 1508, 1457, 1421, 1205, 1156, 1054.

HRMS (ESI): *m/z* = 397.2156 $[M+Na]^+$ (calc. for $C_{23}H_{31}FNaO_3$ *m/z* = 397.2149).

Synthesis of 8f

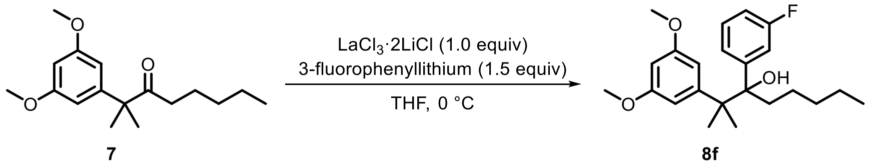

Preparation of 3-fluorophenyllithium:

To a solution of 3-fluoroiodobenzene (861 µL, 7.33 mmol, 2.0 equiv) in dry Et_2O (8.0 mL) was added at –78 ºC *n*-BuLi (1.6 M in hexane, 3.43 mL, 5.50 mmol, 1.5 equiv) dropwise and the solution was stirred at –78 ºC for 30 min.

To a solution of $LaCl_3 \cdot 2LiCl$ (0.6 M in THF, 6.11 mL, 3.66 mmol, 1.0 equiv) was added **7** (1.02 g, 3.66 mmol, 1.0 equiv) in dry THF (3.0 mL) and the resulting mixture was stirred at rt for 1 h. The reaction mixture was cooled to 0 ºC and the previously prepared solution of 3-fluorophenyllithium was added dropwise. The mixture was stirred for 30 min. at 0 °C and then quenched with aq. sat. NH_4Cl solution (15 mL) and H_2O (15 mL). The mixture was diluted with Et_2O (15 mL), the phases were separated and the aqueous phase was extracted with Et_2O (3 × 15 mL). Combined organic extracts were dried with $MgSO_4$, filtered and concentrated under reduced pressure. The residue was purified by flash column chromatography (SiO_2; 0 – 15% EtOAc in hexanes) to afford the product as a colourless oil (1.26 g, 99%).

^{1}H NMR (400 MHz, $CDCl_3$) δ 7.29 – 7.20 (m, 1H), 7.11 – 6.99 (m, 2H), 6.98 – 6.91 (m, 1H), 6.51 (d, J = 2.3 Hz, 2H), 6.41 (t, J = 2.2 Hz, 1H), 3.78 (s, 6H), 2.27 – 2.14 (m, 1H), 1.88 (s, 1H), 1.70 – 1.56 (m, 1H), 1.41 (s, 3H), 1.29 (s, 3H), 1.20 (d, J = 4.2 Hz, 4H), 0.94 – 0.77 (m, 2H), 0.82 (t, J = 6.8 Hz, 3H).

^{13}C NMR (101 MHz, $CDCl_3$) δ 162.33 (d, J = 243.6 Hz), 160.1, 148.0, 145.58 (d, J = 6.6 Hz), 128.15 (d, J = 8.0 Hz), 123.54 (d, J = 2.7 Hz), 115.22 (d, J = 22.4 Hz), 113.11 (d, J = 21.1 Hz), 107.5, 98.0, 80.3, 55.3, 46.6, 35.8, 32.5, 25.0, 24.7, 23.7, 22.7, 14.1.

^{19}F NMR (377 MHz, $CDCl_3$) δ -114.5.

IR (neat, ν_{max}/cm^{-1}): 3554, 2955, 2871, 1594, 1457, 1422, 1205, 1156, 1059.

HRMS (ESI): *m/z* = 397.2144 $[M+Na]^+$ (calc. for $C_{23}H_{31}FNaO_3$ *m/z* = 397.2149).

Synthesis of SI-15a

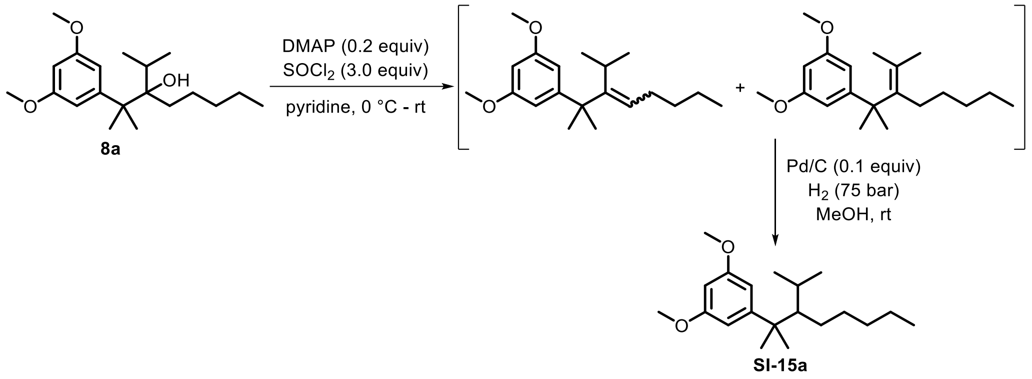

To a solution of **8a** (550 mg, 1.71 mmol, 1.0 equiv) in dry pyridine (6.0 mL) was added DMAP (41.7 mg, 341 µmol, 0.2 equiv). The mixture was cooled to 0 ºC, $SOCl_2$ (373 µL, 5.12 mmol, 3.0 equiv) was added dropwise and the solution was allowed to warm up to rt. and stirred for 30 min. The reaction was quenched by addition of aq. 1 M HCl (30 mL) and diluted with EtOAc (50 mL). The phases were separated and the aqueous phase was extracted with EtOAc (50 mL). Combined organic phases were washed with 0.5 M HCl (2 × 20 mL), dried over $MgSO_4$, filtered and concentrated under reduced pressure. The unpurified material was dissolved in dry MeOH (3.0 mL), Pd (10 wt% on C, 182 mg, 171 µmol, 0.1 equiv) was added and the resulting mixture was stirred in an autoclave at rt for 48 h under 75 bar H_2 pressure. Then the reaction mixture was filtered through Celite, evaporated under reduced pressure, and the residue was purified by flash column chromatography (SiO_2; 0 – 10% EtOAc in hexanes) to afford the product as a colourless oil (425 mg, 81%).

^{1}H NMR (400 MHz, $CDCl_3$) δ 6.52 (d, J = 2.2 Hz, 2H), 6.31 (t, J = 2.2 Hz, 1H), 3.80 (s, 6H), 1.76 – 1.63 (m, 1H), 1.56 (ddd, J = 7.2, 3.9, 1.5 Hz, 1H), 1.34 – 1.21 (m, 8H), 1.25 (s, 3H), 1.21 (s, 3H), 0.90 – 0.80 (m, 9H).

^{13}C NMR (101 MHz, $CDCl_3$) δ 160.4, 154.0, 105.1, 96.6, 55.3, 53.5, 43.1, 32.7, 31.4, 27.8, 27.8, 26.3, 26.3, 24.9, 22.7, 18.6, 14.2.

IR (neat, ν_{max}/cm^{-1}): 2955, 2872, 1595, 1456, 1421, 1205, 1155, 1055.

GC-MS (EI): *m/z* = 306.2557 $[M]^+$ (calc. for $C_{20}H_{34}O_2$ *m/z* = 306.2553).

Synthesis of SI-15b

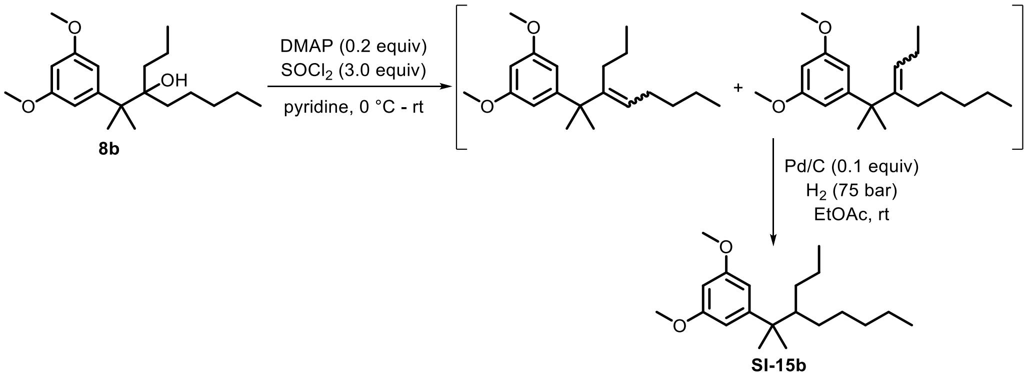

To a solution of **8b** (840 mg, 2.61 mmol, 1.0 equiv) in dry pyridine (6.5 mL) was added DMAP (63.6 mg, 521 µmol, 0.2 equiv). The mixture was cooled to 0 ºC, $SOCl_2$ (570 µL, 7.81 mmol, 3.0 equiv) was added dropwise and the solution was allowed to warm up to rt. and stirred for 30 min. The reaction was quenched by addition of aq. 1 M HCl (30 mL) and diluted with EtOAc (50 mL). The phases were separated and the aqueous phase was extracted with EtOAc (50 mL). Combined organic phases were washed with 0.5 M HCl (2 × 20 mL), dried over $MgSO_4$, filtered and concentrated under reduced pressure. The unpurified material was dissolved in dry EtOAc (7.0 mL), Pd (10 wt% on C, 278 mg, 261 µmol, 0.1 equiv) was added and the resulting mixture was stirred in an autoclave at rt for 24 h under 75 bar hydrogen pressure. Then the reaction mixture was filtered through Celite and the residue was purified by flash column chromatography (SiO_2; 0 – 10% EtOAc in hexanes) to afford the product as a colourless oil (790 mg, 99%).

^{1}H NMR (400 MHz, $CDCl_3$) δ 6.56 (d, J = 2.3 Hz, 2H), 6.34 (t, J = 2.2 Hz, 1H), 3.81 (s, 6H), 1.51 (tt, J = 7.4, 2.6 Hz, 1H), 1.47 – 1.18 (m, 10H), 1.25 (s, 6H), 1.14 – 1.03 (m, 2H), 0.96 – 0.78 (m, 6H).

^{13}C NMR (101 MHz, $CDCl_3$) δ 160.4, 153.5, 105.2, 96.6, 55.1, 48.7, 41.9, 34.5, 32.5, 32.0, 29.8, 25.6, 25.4, 23.1, 22.6, 14.7, 14.1.

IR (neat, ν_{max}/cm^{-1}): 2956, 2931, 2871, 1595, 1456, 1421, 1205, 1155, 1055.

GC-MS (EI): *m/z* =306.2552 $[M]^+$ (calc. for $C_{20}H_{34}O_2$ *m/z* = 306.2553).

Synthesis of SI-15c

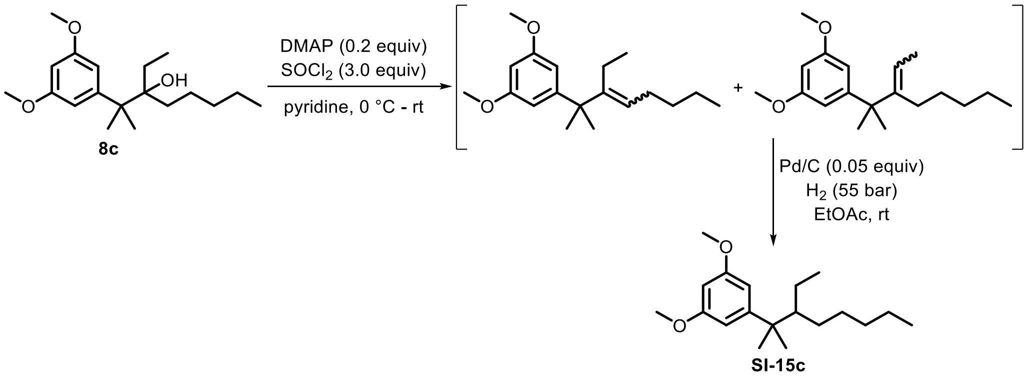

To a solution of **8c** (932 mg, 3.02 mmol, 1.0 equiv) in dry pyridine (12.0 mL) was added DMAP (73.8 mg, 604 µmol, 0.2 equiv). The mixture was cooled to 0 ºC, $SOCl_2$ (661 µL, 9.07 mmol, 3.0 equiv) was added dropwise and the solution was allowed to warm up to rt. and stirred for 30 min. The reaction was quenched by addition of aq. 1 M HCl (30 mL) and diluted with EtOAc (50 mL). The phases were separated and the aqueous phase was extracted with EtOAc (50 mL). Combined organic phases were washed with 0.5 M HCl (2 × 20 mL), dried over $MgSO_4$, filtered and concentrated under reduced pressure. The unpurified material was dissolved in dry EtOAc (6.0 mL), Pd (10 wt% on C, 161 mg, 151 µmol, 0.05 equiv) was added and the resulting mixture was stirred in an autoclave at rt for 16 h under 55 bar H_2 pressure. Then the reaction mixture was filtered through Celite, evaporated under reduced pressure, and the residue was purified by flash column chromatography (SiO_2; 0 – 10% EtOAc in hexanes) to afford the product as a colourless oil (830 mg, 94%).

^{1}H NMR (400 MHz, $CDCl_3$) δ 6.54 (d, J = 2.2 Hz, 2H), 6.32 (t, J = 2.2 Hz, 1H), 3.81 (s, 6H), 1.47 – 1.00 (m, 11H), 1.23 (s, 6H), 0.86 (t, J = 7.2, 3H), 0.86 (t, J = 6.2 Hz, 3H).

^{13}C NMR (101 MHz, $CDCl_3$) δ 160.4, 153.7, 105.2, 96.6, 55.3, 50.9, 42.1, 32.5, 31.5, 30.0, 25.9, 25.3, 24.6, 22.7, 14.6, 14.2.

IR (neat, ν_{max}/cm^{-1}): 2957, 2932, 2872, 1595, 1457, 1421, 1205, 1155, 1055.

HRMS (ESI): *m/z* = 315.2294 $[M+Na]^+$ (calc. for $C_{19}H_{32}NaO_2$ *m/z* = 315.2295).

Synthesis of 9a

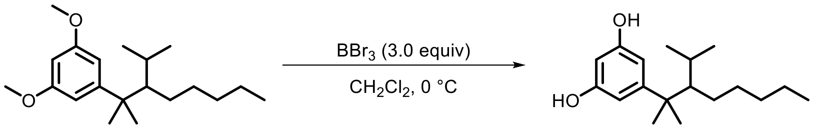

To a solution of **SI-15a** (230 mg, 751 µmol, 1.0 equiv) in anhydrous CH_2Cl_2 (7.5 mL) at 0 °C was added BBr_3 (1.0 M in CH_2Cl_2, 2.25 mL, 2.25 mmol, 3.0 equiv) dropwise and the green solution was stirred at 0 °C for 5.5 h. The mixture was diluted with CH_2Cl_2 (15 mL) and carefully quenched with aq. sat. $NaHCO_3$ (15 mL). The layers were separated and the aqueous phase was extracted with CH_2Cl_2 (2 × 20 mL). Combined organic extracts were dried over $MgSO_4$ and concentrated under reduced pressure. Purification by flash column chromatography (SiO_2; using 0 – 40% EtOAc in hexanes) afforded the product as a golden oil (220 mg, 96%).

^{1}H NMR (400 MHz, $CDCl_3$) δ 6.45 (d, J = 2.2 Hz, 2H), 6.20 (t, J = 2.2 Hz, 1H), 5.78 (bs, 2H), 1.65 (heptd, J = 7.0, 1.3 Hz, 1H), 1.48 (ddd, J = 7.2, 4.0, 1.5 Hz, 1H), 1.33 – 1.09 (m, 8H), 1.16 (s, 3H), 1.13 (s, 3H), 0.86 (t, J = 7.0 Hz, 3H), 0.83 – 0.75 (m, 6H).

^{13}C NMR (101 MHz, $CDCl_3$) δ 156.0, 155.2, 106.5, 100.1, 53.4, 40.8, 32.7, 31.4, 27.8, 27.8, 26.3, 26.2, 24.7, 22.7, 18.6, 14.2.

IR (neat, ν_{max}/cm^{-1}): 3330, 2958, 2872, 1597, 1469, 1325, 1152, 991.

HRMS (ESI): *m/z* = 279.2319 $[M+H]^+$ (calc. for $C_{18}H_{31}O_2$ *m/z* = 279.2319).

Synthesis of 9b

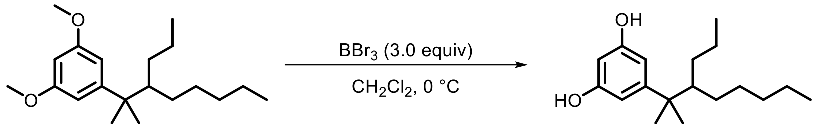

To a solution of **SI-15b** (890 mg, 2.90 mmol, 1.0 equiv) in anhydrous CH_2Cl_2 (30 mL) at 0 °C was added BBr_3 (827 µL, 8.71 mmol, 3.0 equiv) dropwise and the green solution was stirred at 0 °C for 4 h. The mixture was diluted with CH_2Cl_2 (30 mL) and carefully quenched with aq. sat. $NaHCO_3$ (30 mL). The layers were separated and the aqueous phase was extracted with CH_2Cl_2 (2 × 50 mL). Combined organic extracts were dried over $MgSO_4$ and concentrated under reduced pressure. Purification by flash column chromatography (SiO_2; using 0 – 40% EtOAc in hexanes) afforded the product as a golden oil (800 mg, 99%).

^{1}H NMR (400 MHz, $CDCl_3$) δ 6.59 (bs, 2H), 6.49 (d, J = 2.2 Hz, 2H), 6.23 (t, J = 2.1 Hz, 1H), 1.43 – 1.35 (m, 1H), 1.34 – 1.16 (m, 10H), 1.12 (d, J = 2.0 Hz, 6H), 1.04 – 0.92 (m, 2H), 0.84 (t, J = 7.1 Hz, 3H), 0.78 (t, J = 6.9 Hz, 3H).

^{13}C NMR (101 MHz, $CDCl_3$) δ 155.8, 155.0, 106.8, 100.2, 48.6, 41.8, 34.4, 32.5, 31.9, 29.7, 25.6, 25.2, 23.1, 22.7, 14.7, 14.2.

IR (neat, ν_{max}/cm^{-1}): 3324, 2957, 2930, 2871, 1596, 1466, 1325, 1151, 992.

HRMS (ESI): m/z = 279.2318 [M+H]$^+$ (calc. for $C_{18}H_{31}O_2$ m/z = 279.2319).

Synthesis of 9c

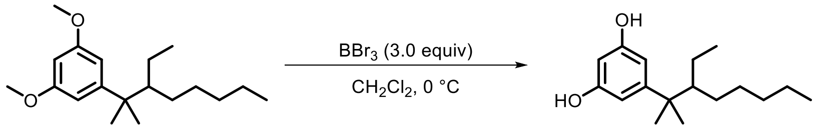

SI-15c 9c

To a solution of **SI-15c** (890 mg, 3.04 mmol, 1.0 equiv) in anhydrous CH_2Cl_2 (30 mL) at 0 °C was added BBr_3 (866 µL, 9.13 mmol, 3.0 equiv) dropwise and the green solution was stirred at 0 °C for 3 h. The mixture was diluted with CH_2Cl_2 (30 mL) and carefully quenched with aq. sat. $NaHCO_3$ (30 mL). The layers were separated and the aqueous phase was extracted with CH_2Cl_2 (2 × 50 mL). Combined organic extracts were dried over $MgSO_4$ and concentrated under reduced pressure. Purification by flash column chromatography (SiO_2; using 0 – 40% EtOAc in hexanes) afforded the product as a golden oil (777 mg, 97%).

^{1}H NMR (400 MHz, $CDCl_3$) δ 6.43 (d, J = 2.2 Hz, 2H), 6.19 (t, J = 2.2 Hz, 1H), 5.38 (bs, 2H), 1.37 – 1.10 (m, 9H), 1.15 (s, 3H), 1.14 (s, 3H), 1.07 – 0.95 (m, 2H), 0.84 (t, J = 7.1 Hz, 3H), 0.80 (t, J = 7.3 Hz, 3H).

^{13}C NMR (101 MHz, $CDCl_3$) δ 156.1, 154.8, 106.5, 100.0, 50.8, 41.9, 32.6, 31.4, 30.0, 25.9, 25.1, 24.6, 22.7, 14.5, 14.2.

IR (neat, ν_{max}/cm^{-1}): 3322, 2959, 2931, 2873, 1596, 1465, 1325, 1150, 991.

HRMS (ESI): *m/z* = 265.2164 [M+H]$^+$ (calc. for $C_{17}H_{29}O_2$ *m/z* = 264.2162).

Synthesis of SI-16d

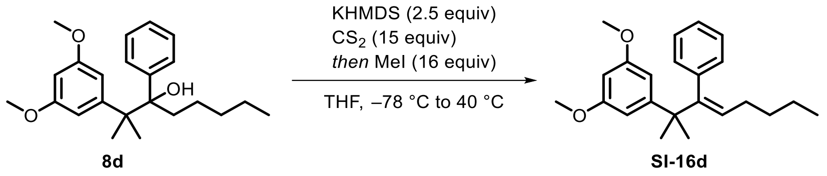

To a solution of **8d** (2.65 g, 7.43 mmol, 1.0 equiv) in anhydrous THF (48 mL) at –78 °C was added KHMDS (1.0 M in THF, 18.6 mL, 18.6 mmol, 2.5 equiv) and CS_2 (6.71 mL, 112 mmol, 15 equiv). The yellow solution was stirred at –78 °C for 10 min, the cooling bath was removed and the mixture stirred for another 30 min. MeI (7.40 mL, 119 mmol, 16 equiv) was added and the mixture was first stirred at ambient temperature for 1 h, and then at 40 °C overnight. The mixture was diluted with Et_2O (50 mL) and aq. sat. $NaHCO_3$ (50 mL) was added. The layers were separated and the aqueous phase was extracted with Et_2O (2 × 50 mL). Combined organic extracts were washed with brine, dried over $MgSO_4$ and concentrated under reduced pressure. Purification by flash column chromatography (SiO_2; using 0.5% Et_2O in pentanes) afforded the product as an amber orange liquid (2.35 g, 93%).

^{1}H NMR (400 MHz, $CDCl_3$) δ 7.19 – 7.11 (m, 3H), 6.75 – 6.64 (m, 2H), 6.55 (d, J = 2.3 Hz, 2H), 6.35 (t, J = 2.3 Hz, 1H), 5.71 (t, J = 7.2 Hz, 1H), 3.79 (s, 6H), 1.78 (q, J = 7.1 Hz, 2H), 1.40 (s, 6H), 1.36 – 1.18 (m, 4H), 0.83 (t, J = 7.2 Hz, 3H).

^{13}C NMR (101 MHz, $CDCl_3$) δ 160.4, 151.2, 148.5, 140.5, 129.7, 127.4, 126.5, 126.1, 105.7, 97.4, 55.3, 44.2, 32.3, 29.1, 29.0, 22.4, 14.1.

IR (neat, ν_{max}/cm^{-1}): 2956, 2872, 1595, 1456, 1423, 1204, 1153.

HRMS (ESI): *m/z* = 339.2315 $[M+H]^+$ (calc. for $C_{23}H_{31}O_2$ *m/z* = 339.2319).

Synthesis of SI-16e

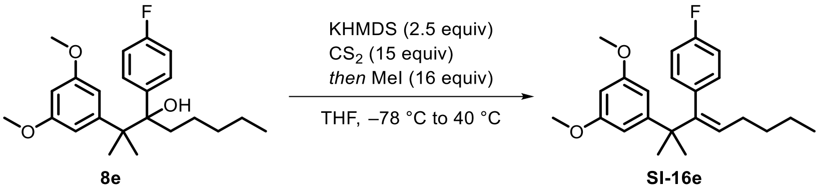

To a solution of **8e** (1.32 g, 3.53 mmol, 1.0 equiv) in anhydrous THF (22 mL) at –78 °C was added KHMDS (0.5 M in toluene, 17.6 mL, 8.81 mmol, 2.5 equiv) and CS_2 (3.20 mL, 52.9 mmol, 15 equiv). The yellow solution was stirred at –78 °C for 10 min, the cooling bath was removed and the mixture stirred for another 30 min. MeI (3.51 mL, 56.4 mmol, 16 equiv) was added and the mixture was first stirred at ambient temperature for 1 h, and then at 40 °C overnight. The mixture was diluted with Et_2O (25 mL) and aq. sat. $NaHCO_3$ (25 mL) was added. The layers were separated and the aqueous phase was extracted with Et_2O (2 × 25 mL). Combined organic extracts were washed with brine, dried over $MgSO_4$ and concentrated under reduced pressure. Purification by flash column chromatography (SiO_2; 0 – 1.5% Et_2O in hexanes) afforded the product as an amber orange liquid (1.25 g, 99%).

^{1}H NMR (400 MHz, $CDCl_3$) δ 6.87 – 6.77 (m, 2H), 6.63 – 6.57 (m, 2H), 6.49 (d, J = 2.3 Hz, 2H), 6.32 (t, J = 2.3 Hz, 1H), 5.72 (t, J = 7.3 Hz, 1H), 3.77 (s, 6H), 1.79 – 1.68 (m, 2H), 1.36 (s, 6H), 1.31 – 1.19 (m, 4H), 0.80 (t, J = 7.1 Hz, 3H).

^{13}C NMR (101 MHz, $CDCl_3$) δ 161.5 (d, J = 244.1 Hz), 160.5, 150.8, 147.5, 136.2 (d, J = 3.4 Hz), 131.2 (d, J = 7.6 Hz), 126.9, 114.3 (d, J = 20.9 Hz), 105.7, 97.4, 55.4, 44.2, 32.2, 29.0, 28.9, 22.4, 14.1.

^{19}F NMR (377 MHz, $CDCl_3$) δ -117.2.

IR (neat, ν_{max}/cm^{-1}): 2958, 2933, 2867, 1600, 1508, 1458, 1422, 1205, 1155, 1059.

HRMS (ESI): *m/z* = 379.2043 $[M+Na]^+$ (calc. for $C_{23}H_{29}FNaO_2$ *m/z* = 379.2044).

Synthesis of SI-16f

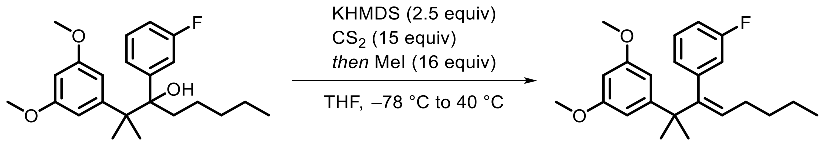

To a solution of **8f** (1.40 g, 3.74 mmol, 1.0 equiv) in anhydrous THF (24 mL) at –78 °C was added KHMDS (0.5 M in toluene, 18.7 mL, 9.35 mmol, 2.5 equiv) and CS_2 (3.39 mL, 56.1 mmol, 15 equiv). The yellow solution was stirred at –78 °C for 10 min, the cooling bath was removed and the mixture stirred for another 30 min. MeI (3.72 mL, 59.8 mmol, 16 equiv) was added and the mixture was first stirred at ambient temperature for 1 h, and then at 40 °C overnight. The mixture was diluted with Et_2O (25 mL) and aq. sat. $NaHCO_3$ (25 mL) was added. The layers were separated and the aqueous phase was extracted with Et_2O (2 × 25 mL). Combined organic extracts were washed with brine, dried over $MgSO_4$ and concentrated under reduced pressure. Purification by flash column chromatography (SiO_2; 0 – 1.2% Et_2O in hexanes) afforded the product as an amber orange liquid (1.09 g, 82%).

^{1}H NMR (400 MHz, $CDCl_3$) δ 7.10 (ddd, J = 8.4, 7.6, 6.1 Hz, 1H), 6.89 – 6.82 (m, 1H), 6.52 (d, J = 2.3 Hz, 2H), 6.50 – 6.46 (m, 1H), 6.41 (ddd, J = 10.2, 2.7, 1.5 Hz, 1H), 6.35 (t, J = 2.2 Hz, 1H), 5.73 (t, J = 7.3 Hz, 1H), 3.79 (s, 6H), 1.82 – 1.73 (m, 2H), 1.39 (s, 6H), 1.37 – 1.20 (m, 4H), 0.83 (t, J = 7.2 Hz, 3H).

^{13}C NMR (101 MHz, $CDCl_3$) δ 162.2 (d, *J* = 244.9 Hz), 160.5, 150.7, 147.4 (d, *J* = 1.6 Hz), 142.8 (d, J = 7.5 Hz), 128.7 (d, J = 8.4 Hz), 127.0, 125.57 (d, J = 2.8 Hz), 116.6 (d, J = 20.8 Hz), 113.0 (d, J = 20.9 Hz), 105.6, 97.5, 55.3, 44.1, 32.2, 29.0, 29.0, 22.4, 14.0.

^{19}F NMR (377 MHz, $CDCl_3$) δ -114.6.

IR (neat, ν_{max}/cm^{-1}): 2957, 2933, 2873, 1598, 1458, 1423, 1205, 1155, 1058.

HRMS (ESI): *m/z* = 379.2041 $[M+Na]^+$ (calc. for $C_{23}H_{29}FNaO_2$ *m/z* = 379.2044).

Synthesis of SI-17d

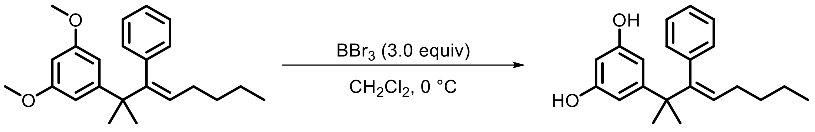

SI-16d SI-17d

To a solution of **SI-16d** (1.70 g, 5.02 mmol, 1.0 equiv) in anhydrous CH_2Cl_2 (48 mL) at 0 °C was added BBr_3 (1.43 mL, 15.1 mmol, 3.0 equiv) dropwise and the green solution was stirred at 0 °C for 3 h. The mixture was diluted with CH_2Cl_2 (100 mL) and carefully quenched with aq. sat. $NaHCO_3$ (50 mL). The layers were separated and the aqueous phase was extracted with CH_2Cl_2 (2 × 100 mL). Combined organic extracts were dried over $MgSO_4$ and concentrated under reduced pressure. Purification by flash column chromatography (SiO_2; using 0 – 30% EtOAc in hexanes) afforded the product as a golden oil (1.54 g, 99%).

^{1}H NMR (400 MHz, $CDCl_3$) δ 7.18 – 7.09 (m, 3H), 6.73 – 6.63 (m, 2H), 6.40 (d, J = 2.3 Hz, 2H), 6.19 (t, J = 2.2 Hz, 1H), 5.66 (t, J = 7.3 Hz, 1H), 4.87 (s, 2H), 1.72 (q, J = 7.2 Hz, 2H), 1.33 (s, 6H), 1.32 – 1.16 (m, 4H), 0.81 (t, J = 7.1 Hz, 3H).

^{13}C NMR (101 MHz, $CDCl_3$) δ 156.2, 152.1, 148.2, 140.5, 129.8, 127.4, 126.6, 126.2, 107.2, 100.4, 43.9, 32.3, 29.0, 28.9, 22.5, 14.1.

IR (neat, ν_{max}/cm^{-1}): 3337, 3054, 2960, 2928, 2872, 1598, 1492, 1465, 1439, 1322, 1149.

HRMS (ESI): *m/z* = 311.2013 $[M+H]^+$ (calc. for $C_{21}H_{27}O_2$ *m/z* = 311.2006).

Synthesis of SI-17e

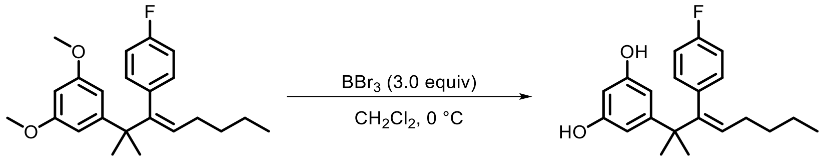

To a solution of **SI-16e** (1.25 g, 3.51 mmol, 1.0 equiv) in anhydrous CH_2Cl_2 (30 mL) at 0 °C was added BBr_3 (1.0 M in CH_2Cl_2, 10.5 mL, 10.5 mmol, 3.0 equiv) dropwise and the green solution was stirred at 0 °C for 5.5 h. The mixture was diluted with CH_2Cl_2 (25 mL) and carefully quenched with aq. sat. $NaHCO_3$ (25 mL). The layers were separated and the aqueous phase was extracted with CH_2Cl_2 (2 × 30 mL). Combined organic extracts were dried over $MgSO_4$ and concentrated under reduced pressure. Purification by flash column chromatography (SiO_2; using 0 – 50% EtOAc in hexanes) afforded the product as a golden oil (1.14 g, 99%).

^{1}H NMR (400 MHz, $CDCl_3$) δ 6.86 – 6.78 (m, 2H), 6.64 – 6.57 (m, 2H), 6.38 (d, J = 2.2 Hz, 2H), 6.20 (t, J = 2.2 Hz, 1H), 5.68 (t, J = 7.2 Hz, 1H), 5.19 (bs, 2H), 1.75 – 1.65 (m, 2H), 1.32 (s, 6H), 1.29 – 1.17 (m, 4H), 0.81 (t, J = 7.2 Hz, 3H).

^{13}C NMR (101 MHz, $CDCl_3$) δ 161.5 (d, J = 244.2 Hz), 156.3, 151.8, 147.2, 136.1 (d, J = 3.5 Hz), 131.2 (d, J = 7.6 Hz), 127.0, 114.3 (d, J = 20.8 Hz), 107.1, 100.5, 43.9, 32.2, 29.0, 28.8, 22.5, 14.1.

^{19}F NMR (377 MHz, $CDCl_3$) δ -117.0.

IR (neat, ν_{max}/cm^{-1}): 3311, 2961, 2929, 2872, 1599, 1507, 1466, 1324, 1220, 1153, 993, 835.

HRMS (ESI): *m/z* = 351.1729 $[M+Na]^+$ (calc. for $C_{21}H_{25}FNaO_2$ *m/z* = 351.1731).

Synthesis of SI-17f

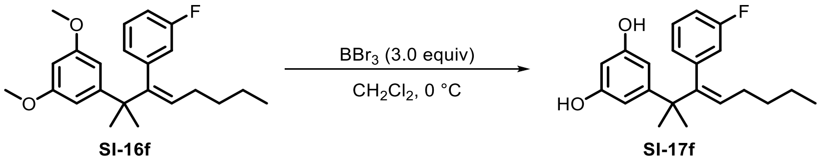

To a solution of **SI-16f** (1.01 g, 2.83 mmol, 1.0 equiv) in anhydrous CH_2Cl_2 (25 mL) at 0 °C was added BBr_3 (1.0 M in CH_2Cl_2, 8.50 mL, 8.50 mmol, 3.0 equiv) dropwise and the green solution was stirred at 0 °C for 5.5 h. The mixture was diluted with CH_2Cl_2 (25 mL) and carefully quenched with aq. sat. $NaHCO_3$ (25 mL). The layers were separated and the aqueous phase was extracted with CH_2Cl_2 (2 × 30 mL). Combined organic extracts were dried over $MgSO_4$ and concentrated under reduced pressure. Purification by flash column chromatography (SiO_2; using 0 – 50% EtOAc in hexanes) afforded the product as a golden oil (920 mg, 99%).

^{1}H NMR (400 MHz, $CDCl_3$) δ 7.08 (dd, J = 8.0, 6.1 Hz, 1H), 6.83 (ddd, J = 8.5, 2.6, 1.0 Hz, 1H), 6.46 (dd, J = 7.6, 1.2 Hz, 1H), 6.40 (d, J = 2.1 Hz, 2H), 6.39 (dd, J = 2.7, 1.7 Hz, 1H), 6.20 (t, J = 2.2 Hz, 1H), 5.67 (t, J = 7.3 Hz, 1H), 5.27 (bs, 2H), 1.75 – 1.68 (m, 2H), 1.31 (s, 6H), 1.29 – 1.17 (m, 4H), 0.81 (t, J = 7.1 Hz, 3H).

^{13}C NMR (101 MHz, $CDCl_3$) δ 162.2 (d, J = 244.7 Hz), 156.2, 151.8, 147.1 (d, *J* = 1.7 Hz), 142.7 (d, *J* = 7.5 Hz), 128.8 (d, *J* = 8.4 Hz), 127.1, 125.6 (d, *J* = 2.7 Hz), 116.6 (d, *J* = 20.8 Hz), 113.1 (d, *J* = 20.9 Hz), 107.1, 100.6, 43.8, 32.1, 29.0, 28.8, 22.4, 14.1.

^{19}F NMR (377 MHz, $CDCl_3$) δ -114.3.

IR (neat, ν_{max}/cm^{-1}): 3341, 2961, 2930, 2872, 1599, 1580, 1466, 1434, 1423, 1324, 1151, 993.

HRMS (ESI): *m/z* = 351.1732 $[M+Na]^+$ (calc. for $C_{21}H_{25}FNaO_2$ *m/z* = 351.1731).

Synthesis of (–)-(*S*)-9d/(A)-9d and (+)-(*R*)-9d/(B)-9d

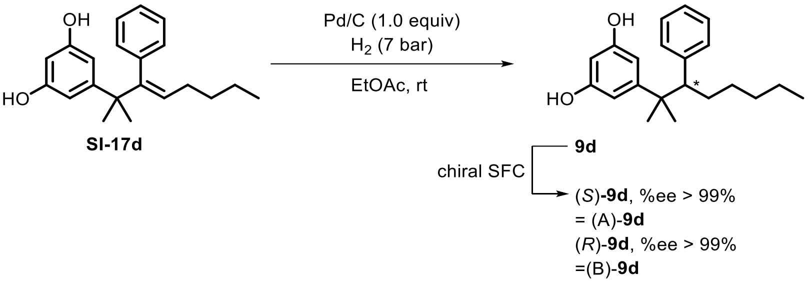

To a solution of **SI-17d** (1.00 g, 3.22 mmol, 1.0 equiv) in EtOAc (27 mL) was added Pd (10 wt% on C, 3.43 g, 3.22 mmol, 1.0 equiv) and the mixture was stirred overnight in an autoclave under 7 bar H_2 pressure. The suspension was filtered over a pad of Celite and concentrated under reduced pressure to afford the product as a pink foam (960 mg, 95% yield).

The racemate was resolved by chiral SFC Method 1 to afford (–)-(*S*)-**9d** (430 mg, 43%, %ee >99%) and (+)-(*R*)-**9d** (420 mg, 42%, %ee >99%).

Note: vide infra for stereochemical assignment

^{1}H NMR (400 MHz, $CDCl_3$) δ 7.28 – 7.14 (m, 3H), 7.11 – 7.03 (m, 2H), 6.39 (d, J = 2.2 Hz, 2H), 6.20 (t, J = 2.2 Hz, 1H), 4.69 (bs, 2H), 2.73 (dd, J = 12.1, 2.9 Hz, 1H), 1.61 (dddd, J = 13.3, 12.1, 9.7, 4.7 Hz, 1H), 1.38 (dddd, J = 13.1, 9.6, 6.5, 2.9 Hz, 1H), 1.22 (s, 3H), 1.18 – 1.01 (m, 4H), 1.07 (s, 3H), 1.00 – 0.79 (m, 2H), 0.74 (t, J = 6.6 Hz, 3H).

^{13}C NMR (101 MHz, $CDCl_3$) δ 156.2, 153.3, 141.8, 130.2, 127.5, 126.2, 106.8, 100.2, 57.1, 41.6, 31.9, 29.4, 29.1, 28.1, 23.5, 22.6, 14.2.

IR (neat, ν_{max}/cm^{-1}): 3340, 2956, 2931, 2870, 1599, 1496, 1467, 1328, 1151, 992, 704.

GC-MS (EI): *m/z* = 312.2084 $[M]^+$ (calc. for $C_{21}H_{28}O_2$ *m/z* = 312.2084).

(–)-(*S*)-9d $[\alpha]^{25}_D$ = –38.557 ± 0.179 (c = 1.0, $CHCl_3$).

(+)-(*R*)-9d $[\alpha]^{25}_D$ = +38.058 ± 0.691 (c = 1.0, $CHCl_3$).

Derivatization studies for the stereochemical assignment of (*S*)-9 and (*R*)-9

Synthesis of (*S*)-SI-19/(A)-9/(*S*)-9

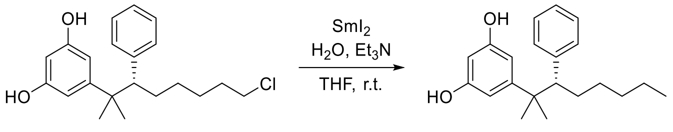

(*S*)-**SI-18** (*S*)-**SI-19**

Note: The stereochemistry of (*S*)-**SI-18** was unambiguously assigned by X-Ray crystallographic analysis of a derivative in our previous work.[2] Herein, we defunctionalized (*S*)-**SI-18** to (S)-**SI-19** to compare optical rotations with authentic (A)-**9d** and thereby assign the C(2') configuration as (*S*) in (–)-(*S*)-**9** (see Note).

To a solution of (*S*)-**SI-18** (11.4 mg, 32.9 µmol, 1.00 equiv) in dry THF (0.2 ml) was added dry triethylamine (128 µL, 920 µmol, 28.0 equiv) and water (20.7 µL, 1.15 mmol, 35.0 equiv). The mixture was thoroughly degassed via freeze-pump-thaw procedure (3x) and back-filled with argon before a solution of SmI_2 (2.30 mL, 230 µmol, 0.1 M in THF, 7.00 equiv) was added dropwise. The ink-blue solution was allowed to stir for 12 h before diluting the reaction mixture with 1M aq HCl and EtOAc. The phases were separated, and the aqueous phase was extracted with EtOAc (3x). The combined organic layers were washed with brine, dried over Na2SO4, filtered, and concentrated under reduced pressure. Purification by flash column chromatography (SiO_2, 20 – 30% EtOAc in *n*-hexane) afforded **XX** (6.00 mg, 19.2 µmol, 58% yield) as a colorless gum.

Note: The ^{1}H NMR spectra and the $[\alpha]^{25}_D$ of (*S*)-**SI-19** matched (A)-**9d**, therefore allowing for the assignment of (*S*)-SI-**19**/(A)-**9d** as (*S*)-**9d**.

^{1}H NMR (400 MHz, $CDCl_3$) δ 7.26 – 7.16 (m, 3H), 7.09 – 7.05 (m, 2H), 6.39 (d, J = 2.2 Hz, 1H), 6.19 (t, J = 2.2 Hz, 1H), 4.64 (bs, 2H), 2.72 (dd, J = 12.2, 2.9 Hz, 1H), 1.65 – 1.59 (m, 1H), 1.37 (dddd, J = 13.2, 9.7, 6.4, 2.9 Hz, 1H), 1.22 (s, 3H), 1.17 – 1.01 (m, 4H), 1.07 (s, 3H), 0.99 – 0.79 (m, 2H), 0.74 (t, J = 6.8 Hz, 3zH).

$[\alpha]^{25}_D$ = –39.250 ± 1.204 ($CHCl_3$, c=0.2

Asymmetric Hydrogenation Study

Scheme S3. Workflow for asymmetric hydrogenation and ee determination

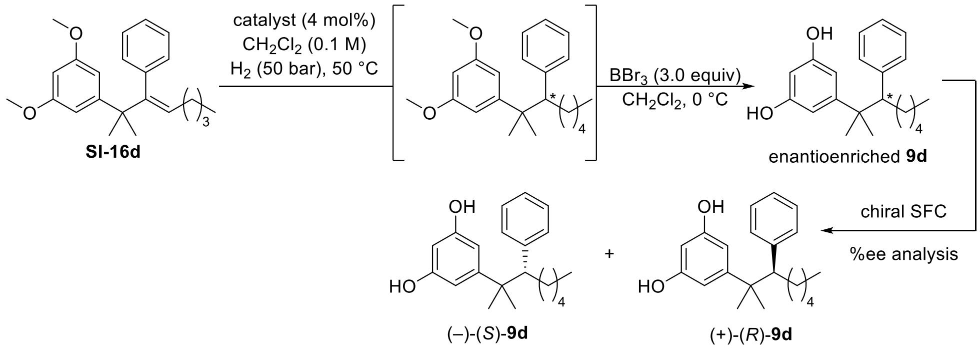

General Procedure:

Catalyst (2.4 µmol, 4 mol%) was weighed into a 5 mL glass vial equipped with a stirring bar and **SI-16d** (20.0 mg. 59.1 µmol) in CH_2Cl_2 (667 uL) was added. The vial was placed in a 350 mL parallel autoclave and pressurized with argon (6 bar). The autoclave was connected to a H_2 line, flushed three times with H_2 (5 × 20 bar) and sealed under H_2 (50 bar). The autoclave was placed in a pre-heated metal block and stirred at 50 °C for 16 h. The pressure was released, and the solvent was removed under reduced pressure. The unpurified product was analyzed by LCMS. If reduction product was observed, the unpurified residue was treated with BBr_3 in analogy to the synthesis of **SI-17e** and analyzed by chiral SFC Method 1.

Note: See SFC chromatogramms for Table S2 entries 1 and 11

Table S2. Screening of $[Ir(L_2)(cod)]BAr^F{}_4$ complexes.

Entry	*Catalyst*	*Conversion/ %*	*ee/ %*
1	[Ir((*S*)-DTB-MeOBIPHEP)(cod)]$BAr^F{}_4$	78	80
2	[Ir((*S,S*)-Cy-UBAPHOX)(cod)]$BAr^F{}_4$	<1	N.A.
3	[Ir((Trifer SL-F131-2)(cod)]$BAr^F{}_4$	<1	N.A.
4	[Ir((*S,S*)-Ph-Bn-SIPHOX)(cod)]$BAr^F{}_4$	<1	N.A.
5	[Ir((*S*)-$Ph_2PCH_2CMe_2$-ox-*t*-Bu)(cod)]$BAr^F{}_4$	<1	N.A.
6	[Ir((*S*)-*t*-Bu_2PCH_2-ox-Ph)(cod)]$BAr^F{}_4$	25	25
7	[Ir((*S*)-Cy_2PCH_2-4-ox-Ph)(cod)]$BAr^F{}_4$	75	11
8	(*S*)-Burgess' catalyst	<1	N.A.
9	[Ir((*R*)-IPr-DHCCP-Ph)(cod)]$BAr^F{}_4$	<1	N.A.
10	[Ir((*R*)-Ph_2PO-THQ-Ph)(cod)]$BAr^F{}_4$	<1	N.A.
11‡	[Ir((*S*)-Cy_2PCH_2-ox-DTB)(cod)]$BAr^F{}_4$	97	32
12	[Ir((*S*)-*t*-Bu_2PO-DHCPP-Ph)(cod)]$BAr^F{}_4$	<1	N.A.
13	[Ir((*R*)-$^oTol_2POCMe_2$-ox-*t*-Bu)(cod)]$BAr^F{}_4$	50	–8

‡The catalyst and substrate were analyzed by the quadrant model outlined by Andersson and Church[28] and suggests that the catalyst yields the (*S*)-enantiomer of **9a** preferentially. Combined with the chiral SFC analysis, the enriched product thus has the (*S*)- configuration at C(2').

Synthesis of (–)-(*S*)-9e & (+)-(*R*)-9e

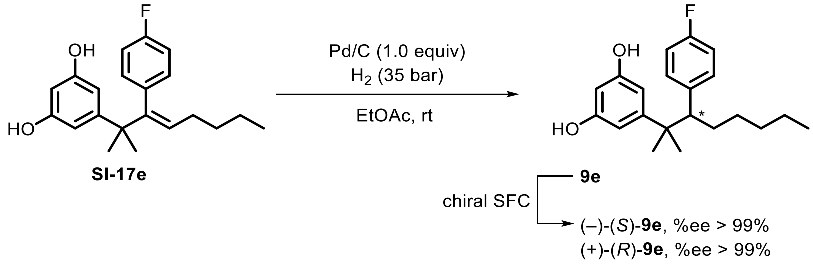

To a solution of **SI-17e** (800 mg, 2.44 mmol, 1.0 equiv) in EtOAc (20 mL) was added Pd (10 wt% on C, 2.59 g, 2.44 mmol, 1.0 equiv) and the mixture was stirred for 48 h at rt in an autoclave under 35 bar H_2 pressure. The suspension was filtered over a pad of Celite and concentrated under reduced pressure. Purification by flash column chromatography (SiO_2; using 20 – 30% EtOAc in hexanes) afforded the product as a golden foam (550 mg, 68% yield). The racemate was resolved by chiral SFC Method 2. to afford (–)-(*S*)-**9e** (170 mg, 21%, %ee >99%) and (+)-(*R*)-**9e** (160 mg, 20%, %ee >99%).

Note: Based on the close structural similarity to (*S*)-**9d** and (*R*)-**9d**, the compounds (–)-**9e** and (+)-**9e** are assigned by analogy based on optical rotation and SFC retention (the (–)-isomer elutes first under compareable conditions, see SFC methods 1 and 2) time as (–)-(*S*)-**9e** and (+)-(*R*)-**9e**.

^{1}H NMR (400 MHz, $CDCl_3$) δ 7.02 – 6.95 (m, 2H), 6.95 – 6.88 (m, 2H), 6.36 (d, J = 2.2 Hz, 2H), 6.21 (t, J = 2.2 Hz, 1H), 5.03 (bs, 2H), 2.71 (dd, J = 12.1, 3.0 Hz, 1H), 1.62 – 1.50 (m, 1H), 1.47 – 1.37 (m, 1H), 1.20 (s, 3H), 1.15 – 1.01 (m, 4H), 1.07 (s, 3H), 0.96 – 0.83 (m, 2H), 0.75 (t, J = 6.7 Hz, 3H).

^{13}C NMR (101 MHz, $CDCl_3$) δ 161.5 (d, J = 243.7 Hz), 156.2, 153.0, 137.4 (d, J = 3.2 Hz), 131.3 (d, J = 7.6 Hz), 114.3 (d, J = 20.8 Hz), 106.8, 100.3, 56.4, 41.6, 31.8, 29.5, 28.6, 28.0, 23.8, 22.6, 14.1.

^{19}F NMR (377 MHz, $CDCl_3$) δ -117.4.

IR (neat, ν_{max}/cm^{-1}): 3345, 2961, 2932, 2863, 1600, 1509, 1225, 1157, 993, 842.

HRMS (ESI): 353.1889 $[M+Na]^+$ (calc. for $C_{21}H_{27}FNaO_2$ *m/z* = 353.1887).

(–)-(*S*)-9e $[\alpha]^{25}_D$ = –40.068 ± 0.134 (c = 1.0, $CHCl_3$).

(+)-(*R*)-9e $[\alpha]^{25}_D$ = +40.982 ± 0.175 (c = 1.0, $CHCl_3$).

Synthesis of (–)-(*S*)-9f & (+)-(*R*)-9f

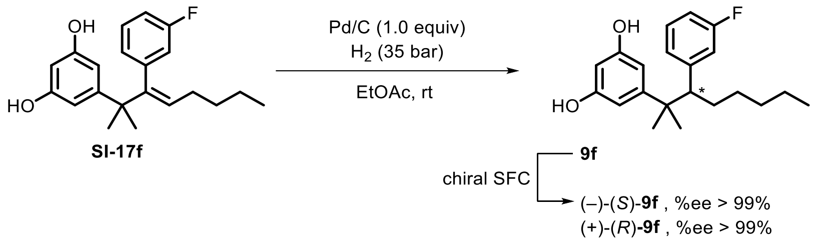

To a solution of **SI-17f** (850 mg, 2.59 mmol, 1.0 equiv) in EtOAc (25 mL) was added Pd (10 wt% on C, 2.75 g, 2.59 mmol, 1.0 equiv) and the mixture was stirred for 48 h at rt in an autoclave under 35 bar H_2 pressure. The suspension was filtered over a pad of Celite and concentrated under reduced pressure. Purification by flash column chromatography (SiO_2; using 20 – 30% EtOAc in hexanes) afforded the product as a golden foam (560 mg, 65% yield). The racemate was resolved by chiral SFC Method 3. to afford (–)-(*S*)-**9f** (200 mg, 23%, %ee >99%) and (+)-(*R*)-**9f** (190 mg, 22%, %ee >99%).

Note: Based on the close structural similarity to (*S*)-**9d** and (*R*)-**9d**, the compounds (–)-**9f** and (+)-**9f** are assigned by analogy based on optical rotation and SFC retention (the (–)-isomer elutes first under compareable conditions, see SFC methods 2 and 4) time as (–)-(*S*)-**9f** and (+)-(*R*)-**9f**.

^{1}H NMR (400 MHz, $CDCl_3$) δ 7.21 – 7.11 (m, 1H), 6.92 – 6.74 (m, 3H), 6.43 (d, J = 2.2 Hz, 2H), 6.26 (t, J = 2.1 Hz, 1H), 6.08 (bs, 2H), 2.73 (dd, J = 12.1, 2.8 Hz, 1H), 1.61 – 1.49 (m, 1H), 1.48 – 1.34 (m, 1H), 1.21 (s, 3H), 1.15 – 1.00 (m, 4H), 1.05 (s, 3H), 0.95 – 0.84 (m, 2H), 0.74 (t, J = 6.9 Hz, 3H).

^{13}C NMR (101 MHz, $CDCl_3$) δ 162.5 (d, J = 243.9 Hz), 156.1, 153.1, 144.7 (d, J = 6.7 Hz), 128.8 (d, J = 8.3 Hz), 126.0 (d, J = 1.2 Hz), 116.6 (d, J = 20.8 Hz), 113.0 (d, J = 21.0 Hz), 106.9, 100.5, 56.9, 41.5, 31.8, 29.4, 28.7, 28.0, 23.6, 22.5, 14.1.

^{19}F NMR (377 MHz, $CDCl_3$) δ -114.4.

IR (neat, ν_{max}/cm^{-1}): 3342, 2960, 2933, 2862, 1595, 1486, 1466, 1445, 1326, 1150, 992, 704.

HRMS (ESI): 353.1882 $[M+Na]^+$ (calc. for $C_{21}H_{27}FNaO_2$ *m/z* = 353.1887).

(–)-(*S*)-9f $[\alpha]^{25}_{D}$ = –40.385 ± 0.408 (c = 1.0, $CHCl_3$).

(+)-(*R*)-9f $[\alpha]^{25}_{D}$ = +42.357 ± 0.102 (c = 1.0, $CHCl_3$).

Synthesis of (*S*/*R*)-SI-20a

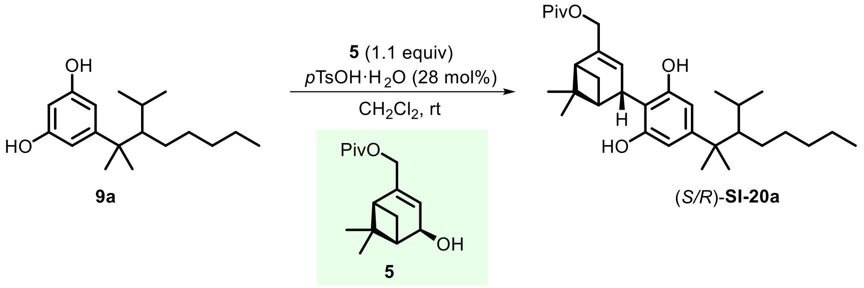

To a solution of **9a** (50.1 mg, 180 µmol, 1.0 equiv) and **5** (49.9 mg, 198 µmol, 1.1 equiv) in CH_2Cl_2 (7.0 mL) was added *p*TsOH·H_2O (9.6 mg, 50.4 µmol, 0.28 equiv), and the mixture was stirred for 1 h at rt. The reaction was quenched by addition of sat. aq. $NaHCO_3$ (3 mL) and diluted with Et_2O (3 mL). The phases were separated, and the aqueous layer was extracted with Et_2O (3 × 3 mL). Combined organic extracts were dried over $MgSO_4$, filtered and concentrated under reduced pressure. Purification by flash column chromatography (SiO_2; 2 – 8% EtOAc in hexanes) afforded the product as a colourless foam (58.6 mg, 64%).

Note: Allylic alcohol **5** was prepared according to our previously published procedure.[27]

^{1}H NMR (400 MHz, $CDCl_3$) δ 6.35 (s, 2H), 6.02 (m, 1H), 5.90 – 5.47 (bs, 2H), 4.68 – 4.59 (m, 1H), 4.55 – 4.47 (m, 1H), 4.05 – 3.99 (m, 1H), 2.42 – 2.33 (m, 1H), 2.32 – 2.27 (m, 2H), 1.71 (dhept, *J* = 7.0, 1.7 Hz, 1H), 1.49 (d, *J* = 1.6 Hz, 1H), 1.48 – 1.43 (m, 1H), 1.34 (s, 3H), 1.30 – 1.05 (m, 8H), 1.23 (s, 9H), 1.18 (s, 3H), 1.14 (s, 3H), 0.98 (s, 3H), 0.85 (t, *J* = 5.2 Hz, 3H), 0.84 (d, *J* =7.2 Hz, 3H), 0.78 (dd, *J* = 6.9, 1.2 Hz, 3H).

^{13}C NMR (101 MHz, $CDCl_3$) δ 178.7, 154.9, 151.9, 149.4, 120.6, 111.5, 106.9, 66.6, 53.4, 47.5, 44.3, 42.4, 41.0, 39.1, 37.9, 32.6, 31.4, 28.2, 28.1, 27.7, 27.4, 27.4, 26.2, 26.0, 25.0, 22.7, 20.9, 18.6, 14.2.

IR (neat, ν_{max}/cm^{-1}): 3457, 2956, 2871, 1707, 1625, 1575, 1480, 1429, 1366, 1283, 1233, 1162, 1027, 837.

HRMS (ESI): *m/z* = 535.3749 $[M+Na]^+$ (calc. for $C_{33}H_{52}NaO_4$ *m/z* = 535.3758).

$[\alpha]^{25}_D$ = +63.340 ± 0.159 (c = 1.0, $CHCl_3$).

Synthesis of (*S*/*R*)-10a

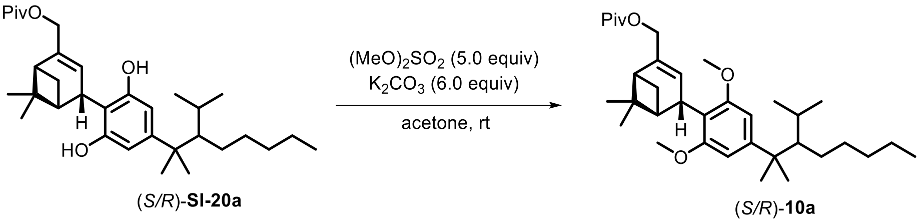

To a suspension of (*S*/*R*)-**SI-20a** (48.1 mg, 93.8 µmol, 1.0 equiv) and K_2CO_3 (77.8 mg, 563 µmol, 6.0 equiv) in acetone (1.0 mL) was added $(MeO_2)SO_2$ (45 µL, 469 µmol, 5.0 equiv). The resulting purple suspension was stirred for 20 h at rt. The reaction was quenched by addition of sat. aq. $NaHCO_3$ (3 mL). The phases were separated, and the aqueous layer was extracted with Et_2O (3 × 3 mL). Combined organic extracts were dried over $MgSO_4$, filtered and concentrated under reduced pressure. Purification by flash column chromatography (SiO_2; 0 – 15% Et_2O in hexanes) afforded the product as a pale pink oil (38.2 mg, 75%).

^{1}H NMR (400 MHz, $CDCl_3$) δ 6.49 (m, 2H), 5.82 – 5.75 (m, 1H), 4.61 – 4.55 (m, 1H), 4.54 – 4.47 (m, 1H), 4.02 – 3.98 (m, 1H), 3.74 (s, 6H), 2.21 – 2.12 (m, 2H), 2.08 – 2.01 (m, 1H), 1.77 – 1.65 (m, 2H), 1.55 – 1.48 (m, 1H), 1.36 – 1.05 (m, 8H), 1.30 (s, 3H), 1.25 (s, 3H), 1.21 (s, 9H), 1.20 (s, 3H), 0.98 (s, 3H), 0.86 (dd, *J* = 7.1, 1.1 Hz, 3H), 0.85 (t, *J* = 7.2 Hz, 3H), 0.80 (d, *J* = 6.9 Hz, 3H).

^{13}C NMR (101 MHz, $CDCl_3$) δ 178.6, 158.4, 150.7, 137.3, 126.6, 117.7, 103.3, 67.7, 55.9, 53.7, 47.5, 44.0, 43.1, 41.0, 39.0, 37.7, 32.7, 31.4, 27.8, 27.7, 27.6, 27.5, 26.5, 26.3, 25.2, 25.1, 22.7, 21.2, 18.6, 14.2.

IR (neat, ν_{max}/cm^{-1}): 2946, 2870, 1728, 1605, 1572, 1461, 1410, 1365, 1280, 1239, 1151, 1122, 832.

HRMS (ESI): *m/z* = 563.4057 $[M+Na]^+$ (calc. for $C_{35}H_{56}NaO_4$ *m/z* = 563.4071).

$[\alpha]^{25}_D$ = +89.637 ± 0.233 (c = 1.0, $CHCl_3$).

Synthesis of (*S*/*R*)-6a

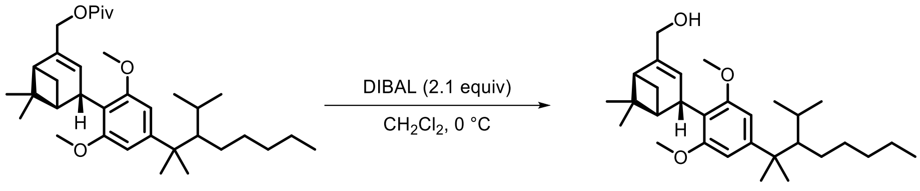

A solution of (*S*/*R*)-**10a** (28.2 mg, 52.1 μmol, 1.0 equiv) in CH_2Cl_2 (0.5 mL) was cooled to 0 °C and DIBAL (1.0 M in hexanes, 110 μL, 110 μmol, 2.1 equiv) was added dropwise. The mixture was stirred at 0 °C for 20 min, subsequently diluted with Et_2O (2 mL) and quenched with sat. aq. Rochelle's salt (1.5 mL). The mixture was stirred vigorously at rt. until the phases cleanly separated. The layers were separated, and the aqueous phase was extracted with Et_2O (4 × 3 mL). Combined organic extracts were washed with brine (5 mL), dried over $MgSO_4$, filtered and concentrated under reduced pressure. Purification by flash column chromatography (SiO_2; 0 – 20% EtOAc in hexanes) afforded the product as a pale pink oil (18.8 mg, 79%).

^{1}H NMR (400 MHz, $CDCl_3$) δ 6.50 (s, 2H), 5.74 – 5.69 (m, 1H), 4.09 – 4.05 (m, 2H), 4.03 – 3.98 (m, 1H), 3.74 (s, 6H), 2.25 – 2.16 (m, 2H), 2.09 – 2.04 (m, 1H), 1.75 – 1.66 (m, 2H), 1.55 – 1.49 (m, 1H), 1.35 – 1.05 (m, 8H), 1.31 (s, 3H), 1.25 (s, 3H), 1.21 (s, 3H), 0.97 (s, 3H), 0.86 (d, *J* = 7.1 Hz, 3H), 0.84 (t, *J* = 7.1 Hz, 3H), 0.80 (dd, *J* = 6.9, 1.1 Hz, 3H).

^{13}C NMR (101 MHz, $CDCl_3$) δ 158.4, 150.7, 142.0, 123.9, 117.8, 103.4, 66.8, 56.0, 53.7, 47.6, 43.9, 43.1, 41.0, 37.6, 32.7, 31.4, 28.0, 27.8, 27.7, 26.4, 26.3, 26.3, 25.2, 22.7, 21.2, 18.6, 14.2.

IR (neat, ν_{max}/cm^{-1}): 3363, 2953, 2930, 2869, 1604, 1572, 1464, 1409, 1238, 1121, 832.

HRMS (ESI): *m/z* = 479.3495 $[M+Na]^+$ (calc. For $C_{30}H_{48}NaO_3$ *m/z* = 479.3496).

$[\alpha]^{25}_D$ = +88.948 ± 0.459 (c = 1.0, $CHCl_3$).

Synthesis of (*S*/*R*)-SI-20b

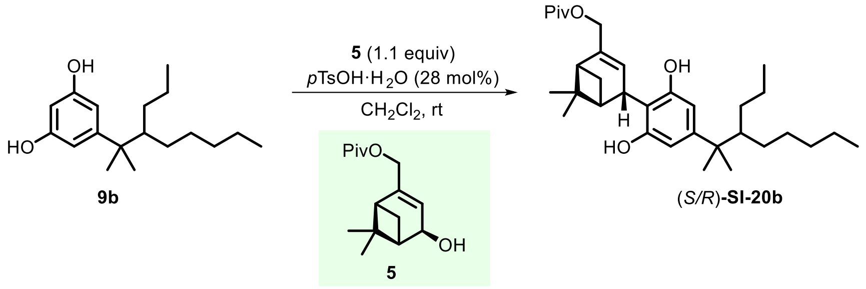

9b (*S*/*R*)-SI-20b

To a solution of (*S*/*R*)-**9b** (50.6 mg, 182 μmol, 1.0 equiv) and **5** (60.0 mg, 236 μmol, 1.3 equiv) in CH_2Cl_2 (7.0 mL), was added *p*TsOH·H_2O (9.7 mg, 50.8 μmol, 0.28 equiv) and the mixture was stirred for 30 min at rt. The reaction was quenched by addition of sat. aq. $NaHCO_3$ (3 mL). The phases were separated, and the aqueous layer was extracted with Et_2O (3 × 5 mL). Combined organic extracts were dried over $MgSO_4$, filtered and concentrated under reduced pressure. Purification by flash column chromatography (SiO_2; 0 – 10% EtOAc in hexanes) afforded the product as a white foam (82.2 mg, 88%).

Note: Allylic alcohol **5** was prepared according to our previously published procedure.[27]

^{1}H NMR (400 MHz, $CDCl_3$) δ 6.35 (s, 2H), 6.04 – 5.99 (m, 1H), 5.90 – 5.51 (bs, 2H), 4.67 – 4.59 (m, 1H), 4.55 – 4.47 (m, 1H), 4.04 – 3.99 (m, 1H), 2.40 – 2.33 (m, 1H), 2.32 – 2.27 (m, 2H), 1.49 (d, *J* = 9.7 Hz, 1H), 1.35 – 0.91 (m, 12H), 1.34 (s, 3H), 1.23 (s, 9H), 1.14 (s, 3H), 1.13 (s, 3H), 0.98 (s, 3H), 0.84 – 0.80 (m, 1H), 0.82 (t, *J* = 7.0 Hz, 3H), 0.80 (t, *J* = 7.0 Hz, 3H).

^{13}C NMR (101 MHz, $CDCl_3$) δ 178.7, 154.9, 151.5, 149.4, 120.6, 111.5, 107.0, 66.6, 48.7, 47.5, 44.3, 41.4, 41.1, 39.1, 37.9, 34.4, 32.5, 31.9, 29.8, 28.1, 27.4, 26.0, 25.7, 25.1, 23.1, 22.7, 20.9, 14.8, 14.2.

IR (neat, ν_{max}/cm^{-1}): 3458, 2956, 2929, 2870, 1730, 1708, 1625, 1574, 1479, 1462, 1429, 1366, 1282, 1232, 1159, 1026, 837, 757.

HRMS (ESI): *m/z* = 535.375 [M+Na]$^+$ (calc. for $C_{33}H_{52}NaO_4$ *m/z* = 535.3758).

$[\alpha]^{25}_D$ = +53.675 ± 0.246 (c = 1.0, $CHCl_3$).

Synthesis of (*S*/*R*)-10b

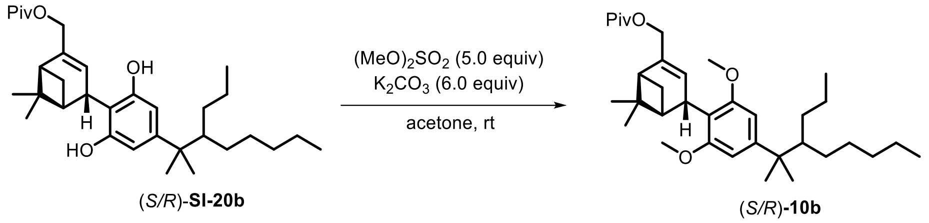

To a suspension of (*S*/*R*)-**SI-20b** (82.2 mg, 160 μmol, 1.0 equiv) and K_2CO_3 (133 mg, 962 μmol, 6.0 equiv) in acetone (1.6 mL) was added $(MeO)_2SO_2$ (76 μL, 80.2 μmol, 5.0 equiv) and the mixture was stirred for 19 h at rt. The suspension was filtered and concentrated under reduced pressure. Purification by flash column chromatography (SiO_2; 0 – 15% Et_2O in hexanes) afforded the product as a pale pink oil (86.0 mg, 99%).

^{1}H NMR (400 MHz, $CDCl_3$) δ 6.49 (s, 2H), 5.81 – 5.76 (m, 1H), 4.62 – 4.55 (m, 1H), 4.54 – 4.48 (m, 1H), 4.03 – 3.98 (m, 1H), 3.74 (s, 6H), 2.20 – 2.13 (m, 2H), 2.08 – 2.01 (m, 1H), 1.75 – 1.68 (m, 1H), 1.46 – 0.97 (m, 12H), 1.30 (s, 3H), 1.22 (s, 12H), 1.21 (s, 3H), 0.98 (s, 3H), 0.85 – 0.80 (m, 1H), 0.82 (t, J = 7.1 Hz, 3H), 0.82 (t, J = 7.0 Hz, 3H).

^{13}C NMR (101 MHz, $CDCl_3$) δ 178.7, 158.4, 150.4, 137.3, 126.6, 117.7, 103.5, 67.7, 56.0, 48.9, 47.6, 44.0, 42.0, 41.0, 39.0, 37.7, 34.6, 32.5, 32.1, 29.9, 27.7, 27.5, 26.5, 26.0, 25.4, 23.1, 22.7, 21.2, 14.8, 14.2.

IR (neat, ν_{max}/cm^{-1}): 2955, 2928, 2868, 1728, 1605, 1572, 1461, 1410, 1280, 1239, 1151, 1123.

HRMS (ESI): m/z = 563.4056 $[M+Na]^+$ (calc. for $C_{35}H_{56}NaO_4$ m/z = 563.4071).

$[\alpha]^{25}_D$ = +65.594 ± 0.383 (c = 1.0, $CHCl_3$).

Synthesis of (*S*/*R*)-6b

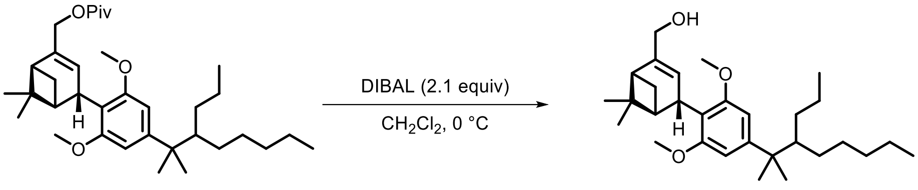

A solution of (*S*/*R*)-**10b** (86.0 mg, 159 μmol, 1.0 equiv) in CH_2Cl_2 (1.6 mL) was cooled to 0 °C and DIBAL (1.0 M in hexanes, 334 μL, 334 μmol, 2.1 equiv) was added dropwise. The mixture was stirred at 0 °C for 20 min, subsequently diluted with CH_2Cl_2 (2 mL) and quenched with sat. aq. Rochelle's salt (5 mL). The mixture was stirred vigorously at rt until the phases cleanly separated. The layers were separated, and the aqueous phase was extracted with Et_2O (5 × 3 mL). Combined organic extracts were dried over $MgSO_4$, filtered and concentrated under reduced pressure. Purification by flash column chromatography (SiO_2; 10 – 20% EtOAc in hexanes) afforded the product (61.6 mg, 85%) as a pale pink oil.

^{1}H NMR (400 MHz, $CDCl_3$) δ 6.50 (s, 2H), 5.73 – 5.69 (m, 1H), 4.08 (s, 2H), 4.03 – 3.98 (m, 1H), 3.74 (s, 6H), 2.26 – 2.17 (m, 2H), 2.09 – 2.03 (m, 1H), 1.71 (d, *J* = 7.9 Hz, 1H), 1.47 – 1.39 (m, 1H), 1.38 – 0.99 (m, 11 H), 1.31 (s, 3H), 1.22 (s, 3H), 1.21 (s, 3H), 0.97 (s, 3H), 0.87 – 0.83 (m, 1H), 0.83 (t, *J* = 7.0 Hz, 3H), 0.82 (t, *J* = 7.1 Hz, 3H).

^{13}C NMR (101 MHz, $CDCl_3$) δ 158.4, 150.3, 142.0, 123.9, 117.8, 103.5, 66.8, 56.0, 48.8, 47.6, 43.9, 42.0, 41.0, 37.6, 34.6, 32.5, 32.0, 29.9, 28.0, 26.4, 25.9, 25.4, 23.1, 22.6, 21.2, 14.8, 14.2.

IR (neat, ν_{max}/cm^{-1}): 3368, 2954, 2930, 2868, 1605, 1571, 1463, 1409, 1238, 1121, 831.

HRMS (ESI): *m/z* = 495.3234 $[M+K]^+$ (calc. for $C_{30}H_{48}KO_3$ *m/z* = 495.3235).

$[\alpha]^{25}_D$ = +92.576 ± 0.103 (c = 1.0, $CDCl_3$).

Synthesis of (*S*/*R*)-SI-20c

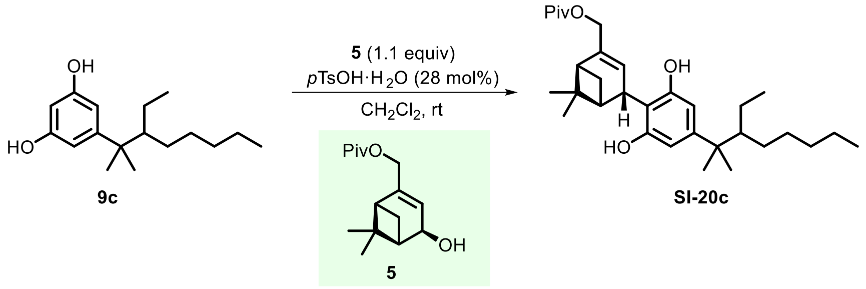

To a solution of **9c** (52.0 mg, 197 µmol, 1.0 equiv) and **5** (54.6 mg, 216 µmol, 1.1 equiv) in CH_2Cl_2 (6.7 mL) was added *p*TsOH·H_2O (10.5 mg. 55.0 µmol, 0.28 equiv) and the solution was stirred for 30 min. The reaction was stopped by addition on sat. aq. $NaHCO_3$ (5 mL) and diluted with Et_2O (10 mL). The layers were separated and the aqueous phase was extracted with Et_2O (3 × 10 mL). Combined organic extracts were dried over $MgSO_4$, filtered and concentrated under reduced pressure. Purification by flash column chromatography (SiO_2; using 0 – 20% EtOAc in hexanes) afforded the product as a colourless foam (72.0 mg, 73%).

Note: Allylic alcohol **5** was prepared according to our previously published procedure.[27]

^{1}H NMR (400 MHz, $CDCl_3$) δ 6.35 (s, 2H), 6.01 (dt, J = 3.1, 1.7 Hz, 1H), 5.63 (bs, 2H), 4.63 (ddd, J = 13.5, 2.0, 1.2 Hz, 1H), 4.51 (ddd, J = 13.5, 2.4, 1.6 Hz, 1H), 4.07 – 3.94 (m, 1H), 2.40 – 2.33 (m, 1H), 2.32 – 2.27 (m, 2H), 1.49 (d, J = 9.6 Hz, 1H), 1.42 – 0.79 (m, 11H), 1.34 (s, 3H), 1.23 (s, 9H), 1.15 (s, 3H), 1.14 (s, 3H), 0.98 (s, 3H), 0.83 (t, J = 6.8 Hz, 3H), 0.82 (t, J = 7.3 Hz, 3H).

^{13}C NMR (101 MHz, $CDCl_3$) δ 178.6, 154.9, 151.6, 149.5, 120.5, 111.5, 107.0, 66.6, 50.8, 47.5, 44.3, 41.5, 41.1, 39.1, 37.9, 32.5, 31.3, 30.0, 28.2, 27.4, 26.0, 25.7, 25.2, 24.6, 22.7, 20.9, 14.5, 14.2.

IR (neat, ν_{max}/cm^{-1}): 3453, 2958, 2931, 2872, 1730, 1708, 1625, 1575, 1480, 1157.

HRMS (ESI): *m/z* = 521.3596 $[M+Na]^+$ (calc. for $C_{32}H_{50}NaO_4$ *m/z* = 521.3601).

$[\alpha]^{25}_D$ = +53.699 ± 0.155 (c = 1.0, $CHCl_3$).

Synthesis of (*S*/*R*)-10c

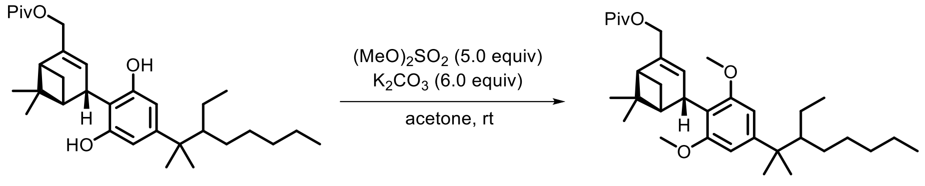

To a solution of (*S*/*R*)-**SI-20c** (43.0 mg, 86.2 μmol, 1.0 equiv) in acetone (0.9 mL) was added $(MeO)_2SO_2$ (40.9 μL. 431 μmol, 5.0 equiv) and K_2CO_3 (71.5 mg. 517 μmol, 6.0 equiv) and the light purple suspension was stirred overnight at rt. The reaction mixture was filtered and concentrated under reduced pressure. Purification by flash column chromatography (SiO_2; using 0 – 5% Et_2O in hexanes) afforded the product as a light red wax (40.0 mg, 88%).

^{1}H NMR (500 MHz, $CDCl_3$) δ 6.49 (s, 2H), 5.78 (dt, J = 2.8, 1.4 Hz, 1H), 4.60 – 4.56 (m, 1H), 4.52 – 4.49 (m, 1H), 4.02 – 3.98 (m, 1H), 3.73 (s, 6H), 2.22 – 2.11 (m, 2H), 2.11 – 1.99 (m, 1H), 1.77 – 1.66 (m, 1H), 1.44 – 1.00 (m, 11H), 1.29 (s, 3H), 1.22 (s, 3H), 1.21 (s, 9H), 1.21 (s, 3H), 0.97 (s, 3H), 0.84 (t, J = 7.3 Hz, 3H), 0.83 (t, J = 7.1 Hz, 3H).

^{13}C NMR (126 MHz, $CDCl_3$) δ 178.6, 158.4, 150.4, 137.3, 126.6, 117.7, 103.5, 67.7, 56.0, 51.1, 47.5, 44.0, 42.1, 41.0, 39.0, 37.7, 32.5, 31.5, 30.0, 27.7, 27.5, 26.5, 25.7, 25.7, 24.7, 22.7, 21.2, 14.6, 14.2.

IR (neat, ν_{max}/cm^{-1}): 2957, 2930, 2871, 1728, 1605, 1573, 1461, 1410, 1151.

HRMS (ESI): *m/z* = 549.3917 $[M+Na]^+$ (calc. for $C_{34}H_{54}NaO_4$ *m/z* = 549.3914).

$[\alpha]^{25}_D$ = +67.051 ± 0.163 (c = 1.0, $CHCl_3$).

Synthesis of (*S/R*)-6c

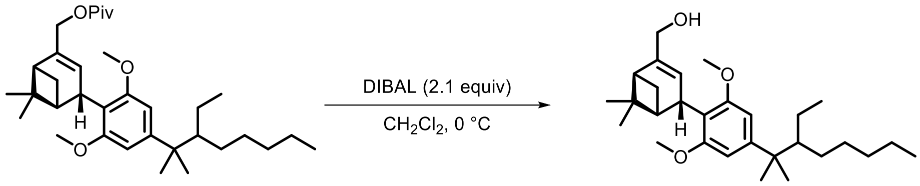

(*S/R*)-**10c** (*S/R*)-**6c**

A solution of (*S/R*)-**10c** (20.0 mg, 37.9 µmol, 1.0 equiv) in CH_2Cl_2 (0.3 mL) was cooled to 0 °C and DIBAL (1.0 M in hexanes, 80 µL. 79.7 µmol, 2.1 equiv) was added dropwise. The mixture was stirred at 0 °C for 15 min. and subsequently quenched with sat. aq. Rochelle's salt (4 mL) and diluted with Et_2O (5 mL). The mixture was vigorously stirred at rt. until the phases cleanly separated. The phases were separated and the aqueous phase extracted with Et_2O (3 × 5 mL). Combined organic fractions were dried over $MgSO_4$, filtered and concentrated under reduced pressure. Purification by flash column chromatography (SiO_2; using 5 – 20% EtOAc in hexanes) afforded the product as a clear wax (15.0 mg, 89%).

^{1}H NMR (500 MHz, $CDCl_3$) δ 6.50 (s, 2H), 5.71 (dt, J = 2.8, 1.4 Hz, 1H), 4.08 – 4.06 (m, 2H), 4.01 – 3.99 (m, 1H), 3.74 (s, 6H), 2.25 – 2.17 (m, 2H), 2.06 (tt, J = 5.8, 1.8 Hz, 1H), 1.71 (d, J = 8.1 Hz, 1H), 1.45 – 0.98 (m, 11H), 1.31 (s, 3H), 1.22 (s, 3H), 1.21 (s, 3H), 0.97 (s, 3H), 0.84 (t, J = 7.4 Hz, 3H), 0.83 (t, J = 7.1 Hz, 3H).

^{13}C NMR (126 MHz, $CDCl_3$) δ 158.4, 150.4, 142.0, 124.0, 117.7, 103.5, 66.8, 56.0, 51.1, 47.6, 44.0, 42.1, 41.0, 37.6, 32.5, 31.5, 30.0, 28.0, 26.4, 25.7, 25.6, 24.7, 22.7, 21.2, 14.6, 14.2.

IR (neat, ν_{max}/cm^{-1}): 3375, 2956, 2929, 2868, 1605, 1572, 1463, 1410, 1239, 1122.

HRMS (ESI): *m/z* = 460.3778 $[M+NH_4]^+$ (calc. for $C_{29}H_{50}NO_3$ *m/z* = 460.3785).

$[\alpha]^{25}_D$ = +88.454 ± 0.171 (c = 1.0, $CHCl_3$).

Synthesis of (*S*)-6c

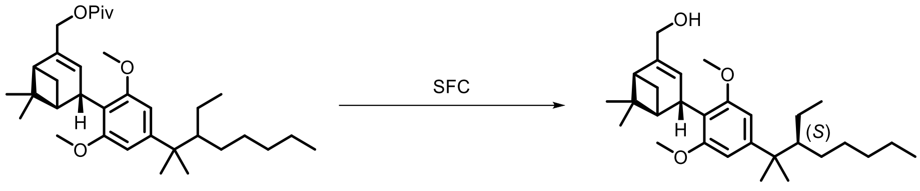

The C(2') diastereomeric mixture of (*S/R*)-**6c** was purified by chiral SFC Method 5 to yield the diastereomer (*S*)-**6c** (4.5 mg, 27%, ee > 99%).

Note: Based on the structural similarity to (*R*)-**1** ($[\alpha]^{25}_{D}$ = +97.242 ± 0.182) and (*S*)-**1** ($[\alpha]^{25}_{D}$ = +58.259 ± 0.119), and taking into account the optical rotation of the C(2') diastereomeric mixture **6c** ($[\alpha]^{25}_{D}$ = +88.454 ± 0.171), the compound is tentatively assigned as (*S*)-**6c**. ^{1}H and ^{13}C NMR spectra are homogenous and indistiguishable from the C(2') epimeric mixture.

$[\alpha]^{25}_{D}$ = +54.405 ± 0.207 (c = 0.45, $CHCl_3$).

Synthesis of (*S*)-SI-20d

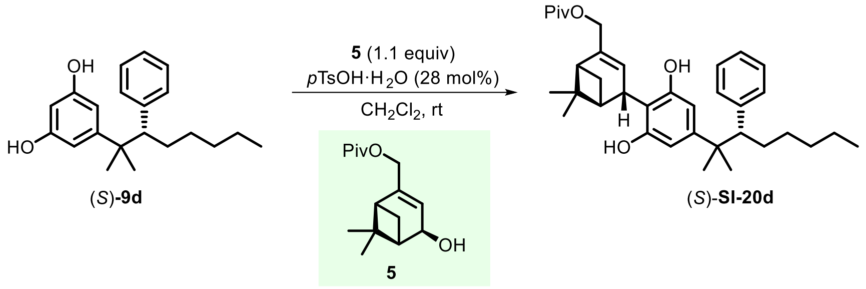

To a solution of resorcinol (*S*)-**9d** (17.6 mg, 56.3 µmol, 1.0 equiv) in anhydrous CH_2Cl_2 (1.4 mL) was added *p*TsOH·H_2O (3.0 mg, 15.7 µmol, 0.28 equiv) and a solution of **5** (15.6 mg, 61.9 µmol, 1.1 equiv) in anhydrous CH_2Cl_2 (0.7 mL). The reaction was stirred at ambient temperature for 30 min. The reaction was quenched by addition of sat. aq. $NaHCO_3$ (2 mL). The phases were separated, and the aqueous layer was extracted with CH_2Cl_2 (3 × 15 mL) and EtOAc (3 × 15 mL). The combined organic extracts were dried over $MgSO_4$ and concentrated under reduced pressure. Purification by flash column chromatography (SiO_2, 5 – 10 % EtOAc in hexanes) afforded the product (23.5 mg, 76 %) as a light-yellow foam.

Note: Allylic alcohol **5** was prepared according to our previously published procedure.[27]

^{1}H NMR (400 MHz, $CDCl_3$) δ 7.25 – 7.14 (m, 3H), 7.12 – 7.05 (m, 2H), 6.36 (s, 2H), 6.04 (dt, J = 3.1, 1.5 Hz, 1H), 5.69 (s, 2H), 4.65 (dt, J = 13.3, 1.6 Hz, 1H), 4.53 (dt, J = 13.5, 1.9 Hz, 1H), 4.03 (s, 1H), 2.72 (dd, J = 12.1, 2.9 Hz, 1H), 2.43 – 2.35 (m, 1H), 2.35 – 2.28 (m, 2H), 1.68 – 1.54 (m, 1H), 1.51 (d, J = 9.6 Hz, 1H), 1.41 – 1.33 (m, 1H), 1.36 (s, 3H), 1.24 (s, 9H), 1.19 (s, 3H), 1.13 – 1.00 (m, 4H), 1.02 (s, 3H), 1.00 (s, 3H), 0.98 – 0.82 (m, 2H), 0.74 (t, J = 6.5 Hz, 3H).

^{13}C NMR (101 MHz, $CDCl_3$) δ 178.7, 154.9, 150.4, 149.5, 142.0, 130.2, 127.5, 126.1, 120.5, 111.8, 107.3, 66.6, 56.9, 47.5, 44.3, 41.2, 41.1, 39.1, 37.9, 31.8, 29.4, 29.3, 28.2, 28.0, 27.4, 26.0, 22.8, 22.5, 20.9, 14.2.

IR (neat, ν_{max}/cm^{-1}): 3455, 2957, 2932, 2871, 1728, 1707, 1625, 1575, 1479, 1465, 1452, 1429, 1367, 1283, 1230, 1163, 1040, 1029, 748, 704.

HRMS (ESI): *m/z* = 569.3603 $[M+Na]^+$ (calc. for $C_{36}H_{50}NaO_4$ *m/z* = 569.3601).

$[\alpha]^{25}_D$ = +19.921 ± 0.144 (c = 1.0, $CHCl_3$).

Synthesis of (*R*)-SI-20d

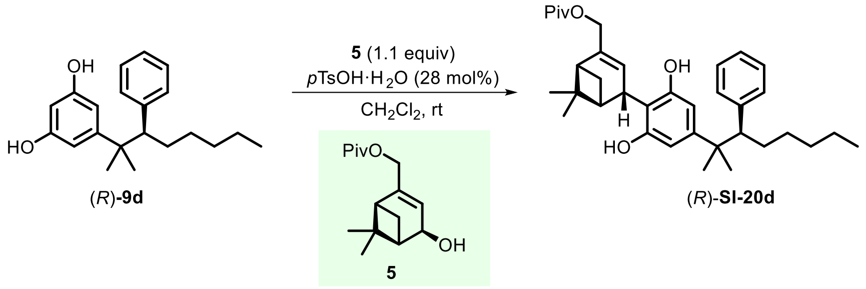

To a solution of resorcinol (*R*)-**9d** (18.3 mg, 58.5 μmol, 1.0 equiv) in anhydrous CH_2Cl_2 (1.7 mL) was added *p*TsOH·H_2O (3.1 mg, 16.4 μmol, 0.28 equiv) and a solution of **5** (16.3 mg, 64.4 μmol, 1.1 equiv) in anhydrous CH_2Cl_2 (0.5 mL). The reaction was stirred at ambient temperature for 30 min. The reaction was quenched by addition of sat. aq. $NaHCO_3$ (2 mL). The phases were separated, and the aqueous layer was extracted with CH_2Cl_2 (3 × 15 mL) and EtOAc (3 × 15 mL). The combined organic extracts were dried over $MgSO_4$ and concentrated under reduced pressure. Purification by flash column chromatography (SiO_2, 5 – 10% EtOAc in hexanes) afforded the product (24.3 mg, 76%) as a light-yellow foam.

Note: Allylic alcohol **5** was prepared according to our previously published procedure.[27]

^{1}H NMR (400 MHz, $CDCl_3$) δ 7.25 – 7.14 (m, 3H), 7.10 – 6.98 (m, 2H), 6.36 (s, 2H), 6.04 (dt, J = 3.3, 1.6 Hz, 1H), 5.68 (bs, 2H), 4.65 (dt, J = 13.3, 1.7 Hz, 1H), 4.52 (dt, J = 13.5, 2.0 Hz, 1H), 4.08 – 3.97 (m, 1H), 2.71 (dd, J = 12.1, 2.9 Hz, 1H), 2.44 – 2.35 (m, 1H), 2.34 – 2.28 (m, 2H), 1.69 – 1.56 (m, 1H), 1.52 (d, J = 9.7 Hz, 1H), 1.44 – 1.33 (m, 1H), 1.36 (s, 3H), 1.24 (s, 9H), 1.19 (s, 3H), 1.16 – 1.00 (m, 4H), 1.03 (s, 3H), 0.99 (s, 3H), 0.96 – 0.82 (m, 2H), 0.74 (t, J = 6.5 Hz, 3H).

^{13}C NMR (101 MHz, $CDCl_3$) δ 178.7, 154.9, 150.3, 149.6, 142.0, 130.2, 127.5, 126.1, 120.5, 111.8, 107.3, 66.6, 57.0, 47.5, 44.3, 41.2, 41.1, 39.1, 37.9, 31.9, 29.4, 29.4, 28.2, 28.0, 27.4, 26.0, 22.9, 22.5, 20.9, 14.2.

IR (neat, ν_{max}/cm^{-1}): 3459, 2957, 2932, 2871, 1729, 1707, 1625, 1575, 1480, 1465, 1452, 1429, 1367, 1326, 1283, 1230, 1163, 1029, 759, 704.

HRMS (ESI): *m/z* = 569.3592 $[M+Na]^+$ (calc. for $C_{36}H_{50}NaO_4$ *m/z* = 569.3601).

$[\alpha]^{25}_D$ = +82.985 ± 0.109 (c = 1.0, $CHCl_3$).

Synthesis of (*S*)-10d

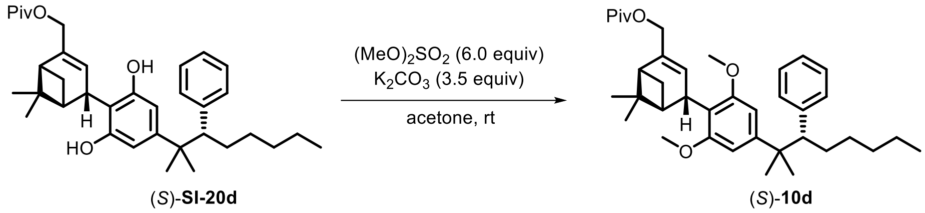

To a solution of (*S*)-**SI-20d** (23.5 mg, 42.9 µmol, 1.0 equiv) in acetone (0.5 mL) was added $(MeO)_2SO_2$ (12.2 µL. 129 µmol, 3.0 equiv) and K_2CO_3 (20.8 mg. 0.150 mmol, 3.5 equiv) and the light red suspension was stirred overnight at rt. Subsequently, $(MeO)_2SO_2$ (12.2 µL. 0.129 mmol, 3.0 equiv) was added and the solution stirred for 2h at rt. The reaction mixture was diluted with Et_2O (5 mL) and water (5 mL). The layers were separated and the aqueous layer extracted with Et_2O (2 × 5 mL). Combined organic fractions were dried over $MgSO_4$, filtered and concentrated under reduced pressure. Purification by flash column chromatography (SiO_2; using 0 to 5% Et_2O in hexanes) afforded the product as a light red wax (21.8 g, 88%).

^{1}H NMR (500 MHz, $CDCl_3$) δ 7.25 – 7.15 (m, 3H), 7.07 – 6.98 (m, 2H), 6.42 (s, 2H), 5.79 (dt, J = 2.7, 1.4 Hz, 1H), 4.65 – 4.55 (m, 1H), 4.55 – 4.46 (m, 1H), 4.05 – 3.97 (m, 1H), 3.69 (s, 6H), 2.72 (dd, J = 12.1, 2.9 Hz, 1H), 2.21 – 2.13 (m, 2H), 2.09 – 2.02 (m, 1H), 1.73 (d, J = 7.8 Hz, 1H), 1.61 (dddd, J = 13.5, 11.9, 9.5, 4.6 Hz, 1H), 1.50 – 1.40 (m, 1H), 1.31 (s, 3H), 1.27 (s, 3H), 1.22 (s, 9H), 1.14 (s, 3H), 1.13 – 1.02 (m, 4H), 0.98 (s, 3H), 0.97 – 0.84 (m, 2H), 0.75 (t, J = 7.0 Hz, 3H).

^{13}C NMR (126 MHz, $CDCl_3$) δ 178.6, 158.3, 148.7, 142.0, 137.4, 130.2, 127.4, 126.5, 126.1, 118.0, 104.0, 67.6, 57.3, 56.0, 47.6, 44.0, 41.8, 41.0, 39.0, 37.7, 31.9, 29.3, 28.8, 28.1, 27.7, 27.5, 26.5, 24.1, 22.6, 21.2, 14.2.

IR (neat, ν_{max}/cm^{-1}): 2932, 2870, 1727, 1604, 1573, 1453, 1411, 1151, 1122.

HRMS (ESI): *m/z* = 597.3915 $[M+Na]^+$ (calc. for $C_{38}H_{54}NaO_4$ *m/z* = 597.3914).

$[\alpha]^{25}_D$ = +38.804 ± 0.225 (c = 1.0, $CHCl_3$).

Synthesis of (*R*)-10d

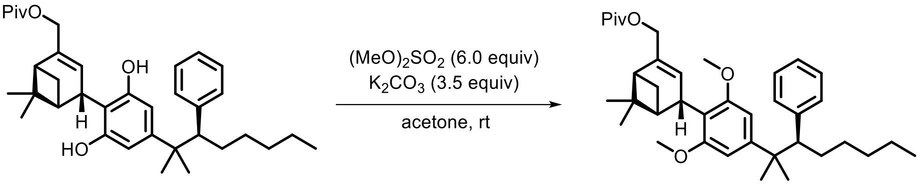

(*R*)-**SI-20d** (*R*)-**10d**

To a solution of (*R*)-**SI-20** (24.3 mg, 44.4 µmol, 1.0 equiv) in acetone (0.5 mL) was added $(MeO)_2SO_2$ (12.6 µL. 0.133 mmol, 3.0 equiv) and K_2CO_3 (21.5 mg. 0.155 mmol, 3.5 equiv) and the light red suspension was stirred overnight at rt. Subsequently, $(MeO)_2SO_2$ (12.6 µL, 0.133 mmol, 3.0 equiv) was added and the solution stirred for 2 h at rt. The reaction mixture was diluted with Et_2O (5 mL) and water (5 mL). The layers were separated and the aqueous layer extracted with Et_2O (2 × 5 mL). Combined organic fractions were dried over $MgSO_4$, filtered and concentrated under reduced pressure. Purification by flash column chromatography (SiO_2; using 0 – 5% Et_2O in hexanes) afforded the product as a light red wax (23.4 g, 92%).

^{1}H NMR (400 MHz, $CDCl_3$) δ 7.25 – 7.15 (m, 3H), 7.06 – 6.99 (m, 2H), 6.42 (s, 2H), 5.80 (dt, J = 2.8, 1.4 Hz, 1H), 4.61 – 4.56 (m, 1H), 4.54 – 4.49 (m, 1H), 4.03 – 3.98 (m, 1H), 3.69 (s, 6H), 2.72 (dd, J = 12.0, 2.9 Hz, 1H), 2.23 – 2.13 (m, 2H), 2.06 (ddt, J = 5.4, 3.6, 1.8 Hz, 1H), 1.75 – 1.54 (m, 2H), 1.51 – 1.39 (m, 1H), 1.31 (s, 3H), 1.27 (s, 3H), 1.22 (s, 9H), 1.15 (s, 3H), 1.12 – 1.03 (m, 4H), 0.99 (s, 3H), 0.96 – 0.83 (m, 2H), 0.75 (t, J = 6.9 Hz, 3H).

^{13}C NMR (101 MHz, $CDCl_3$) δ 178.6, 158.2, 148.7, 142.0, 137.3, 130.2, 127.4, 126.6, 126.1, 118.0, 103.9, 67.7, 57.3, 55.9, 47.6, 44.0, 41.8, 41.1, 39.1, 37.7, 31.9, 29.4, 28.9, 28.1, 27.7, 27.5, 26.5, 24.1, 22.6, 21.2, 14.2.

IR (neat, ν_{max}/cm^{-1}): 2931, 2869, 1727, 1604, 1573, 1452, 1411, 1151, 1122.

HRMS (ESI): m/z = 597.3911 $[M+Na]^+$ (calc. for $C_{38}H_{54}NaO_4$ m/z = 597.3914).

$[\alpha]^{25}_D$ = +86.208 ± 0.243 (c = 1.0, $CHCl_3$).

Synthesis of (*S*)-6d

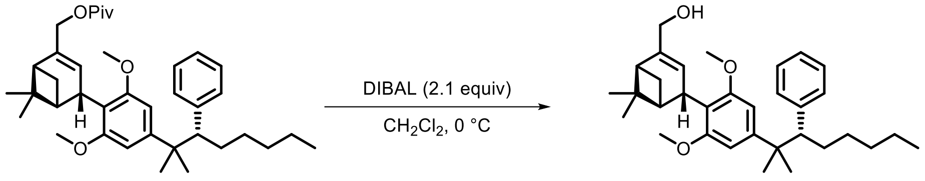

(*S*)-**10d** (*S*)-**6d**

Solution of (*S*)-**10d** (22.0 mg, 38.2 µmol, 1.0 equiv) in CH_2Cl_2 (0.4 mL) was cooled to 0 °C and DIBAL (1.0 M in hexanes, 81 µL. 80.7 µmol, 2.1 equiv) was added dropwise. The mixture was stirred at 0 °C for 15 min. and subsequently quenched with sat. aq. Rochelle's salt (4 mL) and diluted with Et_2O (5 mL). The mixture was vigorously stirred at rt. until the phases cleanly separated. The phases were separated and the aqueous phase extracted with Et_2O (2 × 5 mL). Combined organic fractions were dried over $MgSO_4$, filtered and concentrated under reduced pressure. Purification by flash column chromatography (SiO_2; using 10 – 15% EtOAc in hexanes) afforded the product as a white foam (16.0 mg, 85%).

^{1}H NMR (400 MHz, $CDCl_3$) δ 7.25 – 7.14 (m, 3H), 7.08 – 6.96 (m, 2H), 6.43 (s, 2H), 5.76 – 5.68 (m, 1H), 4.08 (s, 2H), 4.05 – 3.98 (m, 1H), 3.70 (s, 6H), 2.73 (dd, J = 12.0, 2.9 Hz, 1H), 2.28 – 2.18 (m, 2H), 2.11 – 2.00 (m, 1H), 1.78 – 1.69 (m, 1H), 1.68 – 1.54 (m, 1H), 1.52 – 1.39 (m, 1H), 1.32 (s, 3H), 1.27 (s, 3H), 1.15 (s, 3H), 1.13 – 1.02 (m, 4H), 0.98 (s, 3H), 0.96 – 0.81 (m, 2H), 0.75 (t, J = 6.8 Hz, 3H).

^{13}C NMR (101 MHz, $CDCl_3$) δ 158.3, 148.7, 142.1, 142.0, 130.2, 127.4, 126.1, 123.9, 118.1, 104.0, 66.8, 57.3, 56.0, 47.6, 44.0, 41.8, 41.0, 37.6, 31.9, 29.4, 28.9, 28.1, 28.0, 26.4, 24.1, 22.6, 21.2, 14.2.

IR (neat, ν_{max}/cm^{-1}): 3398, 2931, 2866, 1604, 1573, 1410, 1122.

HRMS (ESI): m/z = 513.3335 $[M+Na]^+$ (calc. for $C_{33}H_{46}NaO_3$ m/z = 513.3339).

$[\alpha]^{25}_D$ = +57.735 ± 0.424 (c = 1.0, $CHCl_3$).

Synthesis of (*R*)-6d

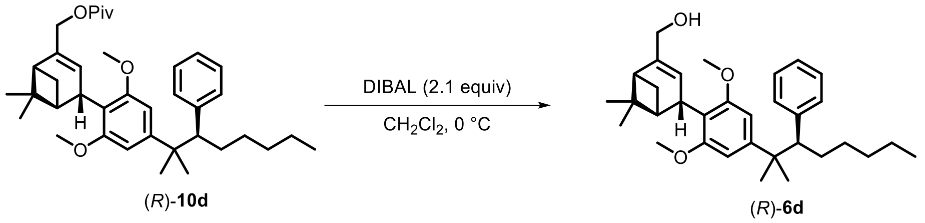

Solution of (*R*)-**10d** (23.4 mg, 40.7 µmol, 1.0 equiv) in CH_2Cl_2 (0.4 mL) was cooled to 0 °C and DIBAL (1.0 M in hexanes, 86 µL. 85.4 µmol, 2.1 equiv) was added dropwise. The mixture was stirred at 0 °C for 15 min. and subsequently quenched with sat. aq. Rochelle's salt (4 mL) and diluted with Et_2O (5 mL). The mixture was vigorously stirred at rt. until the phases cleanly separated. The phases were separated and the aqueous phase extracted with Et_2O (2 × 5 mL). Combined organic fractions were dried over $MgSO_4$, filtered and concentrated under reduced pressure. Purification by flash column chromatography (SiO_2; using 10 – 15% EtOAc in hexanes) afforded the product as a white foam (17.3 mg, 87%).

^{1}H NMR (400 MHz, $CDCl_3$) δ 7.26 – 7.13 (m, 3H), 7.06 – 6.99 (m, 2H), 6.43 (s, 2H), 5.79 – 5.68 (m, 1H), 4.08 (s, 2H), 4.03 – 4.00 (m, 1H), 3.70 (s, 6H), 2.73 (dd, J = 12.1, 2.9 Hz, 1H), 2.28 – 2.18 (m, 2H), 2.10 – 2.05 (m, 1H), 1.76 – 1.67 (m, 1H), 1.67 – 1.54 (m, 1H), 1.50 – 1.39 (m, 1H), 1.32 (s, 3H), 1.27 (s, 3H), 1.15 (s, 3H), 1.13 – 1.03 (m, 4H), 0.98 (s, 3H), 0.96 – 0.84 (m, 2H), 0.75 (t, J = 6.7 Hz, 3H).

^{13}C NMR (101 MHz, $CDCl_3$) δ 158.3, 148.7, 142.0, 142.0, 130.2, 127.4, 126.1, 123.9, 118.0, 104.0, 66.8, 57.3, 56.0, 47.6, 44.0, 41.8, 41.0, 37.6, 31.9, 29.4, 28.9, 28.1, 28.0, 26.4, 24.1, 22.6, 21.2, 14.2.

IR (neat, ν_{max}/cm^{-1}): 3369, 2931, 2867, 1604, 1573, 1410, 1122.

HRMS (ESI): m/z = 513.3335 $[M+Na]^+$ (calc. for $C_{33}H_{46}NaO_3$ m/z = 513.3339).

$[\alpha]^{25}_D$ = +123.317 ± 0.334 (c = 1.0, $CHCl_3$).

Synthesis of (*S*)-SI-20e

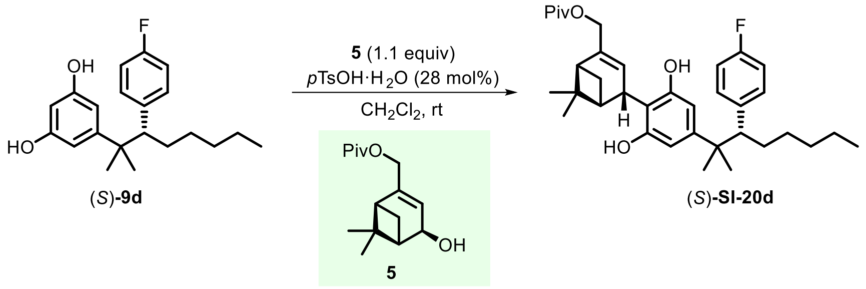

To a solution of (*S*)-**9d** (24.0 mg, 72.6 µmol, 1.0 equiv) and **5** (20.2 mg, 79.8 µmol, 1.1 equiv) in CH_2Cl_2 (3.0 mL) was added pTsOH·H_2O (3.8 mg. 20.3 µmol, 0.28 equiv) and the solution was stirred for 30 min. The reaction was stopped by addition of sat. aq. $NaHCO_3$ (3 mL) and diluted with Et_2O (5 mL). The layers were separated and the aqueous phase was extracted with Et_2O (3 × 5 mL). Combined organic extracts were dried over $MgSO_4$, filtered and concentrated under reduced pressureo. Purification by flash column chromatography (SiO_2; using 0 – 10% EtOAc in hexanes) afforded the product as a white foam (32.3 mg, 79%).

Note: Allylic alcohol **5** was prepared according to our previously published procedure.[27]

^{1}H NMR (400 MHz, $CDCl_3$) δ 7.04 – 6.94 (m, 2H), 6.94 – 6.87 (m, 2H), 6.31 (s, 2H), 6.03 (dt, J = 3.1, 1.6 Hz, 1H), 5.69 (bs, 2H), 4.65 (dt, J = 13.4, 1.5 Hz, 1H), 4.52 (ddd, J = 13.5, 2.4, 1.6 Hz, 1H), 4.05 – 4.00 (m, 1H), 2.69 (dd, J = 12.0, 3.0 Hz, 1H), 2.39 (dt, J = 9.6, 5.6 Hz, 1H), 2.35 – 2.24 (m, 2H), 1.59 – 1.48 (m, 1H), 1.50 (d, J = 9.7 Hz, 1H), 1.47 – 1.38 (m, 1H), 1.35 (s, 3H), 1.24 (s, 9H), 1.17 (s, 3H), 1.15 – 1.05 (m, 4H), 1.03 (s, 3H), 0.99 (s, 3H), 0.94 – 0.83 (m, 2H), 0.75 (t, J = 6.7 Hz, 3H).

^{13}C NMR (101 MHz, $CDCl_3$) δ 178.7, 161.5 (d, J = 243.6 Hz), 154.9, 149.8, 149.6, 137.6 (d, J = 3.2 Hz), 131.3 (d, J = 7.5 Hz), 120.4, 114.2 (d, J = 20.8 Hz), 111.9, 107.3, 66.6, 56.3, 47.5, 44.3, 41.2, 41.1, 39.1, 37.9, 31.8, 29.5, 28.9, 28.2, 28.0, 27.4, 26.0, 23.4, 22.6, 20.9, 14.1.

^{19}F NMR (377 MHz, $CDCl_3$) δ -117.6.

IR (neat, ν_{max}/cm^{-1}): 3453, 2956, 2928, 2871, 2857, 1729, 1707, 1625, 1575, 1509, 1225, 1160.

HRMS (ESI): m/z = 587.3509 $[M+Na]^+$ (calc. for $C_{36}H_{49}FNaO_4$ m/z = 587.3507).

$[\alpha]^{25}_D$ = +28.542 ± 0.383 (c = 1.0, $CHCl_3$).

Synthesis of (*R*)-SI-20e

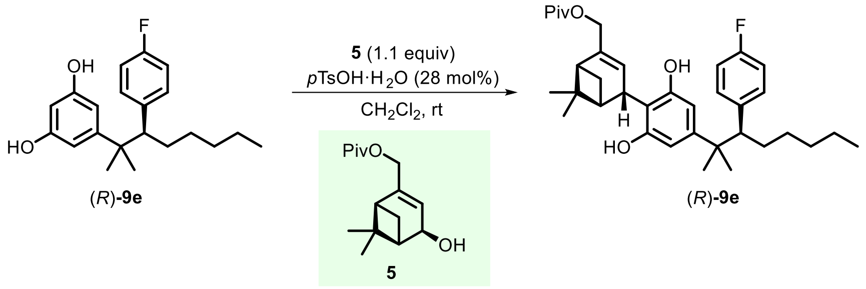

To a solution of (*R*)-**9e** (26.6 mg, 80.5 µmol, 1.0 equiv) and **5** (22.4 mg, 88.5 µmol, 1.1 equiv) in CH_2Cl_2 (3.3 mL) was added *p*TsOH·H_2O (4.3 mg. 22.5 µmol, 0.28 equiv) and the solution was stirred for 30 min. The reaction was stopped by addition of sat. aq. $NaHCO_3$ (3 mL) and diluted with Et_2O (5 mL). The layers were separated and the aqueous phase was extracted with Et_2O (3 × 5 mL). Combined organic extracts were dried over $MgSO_4$, filtered and concentrated under reduced pressure. Purification by flash column chromatography (SiO_2; using 0 – 10% EtOAc in hexanes) afforded the product as a white foam (38.5 mg, 85%).

Note: Allylic alcohol **5** was prepared according to our previously published procedure.[27]

^{1}H NMR (500 MHz, $CDCl_3$) δ 7.03 – 6.96 (m, 2H), 6.96 – 6.87 (m, 2H), 6.32 (s, 2H), 6.03 (dt, J = 3.1, 1.5 Hz, 1H), 5.70 (bs, 2H), 4.65 (ddd, J = 13.5, 2.0, 1.4 Hz, 1H), 4.52 (ddd, J = 13.5, 2.4, 1.6 Hz, 1H), 4.09 – 3.96 (m, 1H), 2.69 (dd, J = 12.1, 3.0 Hz, 1H), 2.39 (dt, J = 9.7, 5.6 Hz, 1H), 2.35 – 2.25 (m, 2H), 1.58 – 1.48 (m, 1H), 1.50 (d, J = 9.8 Hz, 1H), 1.45 – 1.38 (m, 1H), 1.35 (s, 3H), 1.24 (s, 9H), 1.17 (s, 3H), 1.14 – 1.05 (m, 4H), 1.02 (s, 3H), 0.99 (s, 3H), 0.95 – 0.81 (m, 2H), 0.74 (t, J = 6.6 Hz, 3H).

^{13}C NMR (126 MHz, $CDCl_3$) δ 178.7, 161.5 (d, J = 243.6 Hz), 154.9, 149.9, 149.6, 137.6 (d, J = 3.2 Hz), 131.3 (d, J = 7.5 Hz), 120.4, 114.3 (d, J = 20.8 Hz), 111.9, 107.3, 66.6, 56.2, 47.5, 44.3, 41.2, 41.1, 39.1, 37.9, 31.8, 29.4, 28.8, 28.2, 28.0, 27.4, 26.0, 23.2, 22.6, 20.9, 14.1.

^{19}F NMR (471 MHz, $CDCl_3$) δ -117.6.

IR (neat, ν_{max}/cm^{-1}): 3453, 2956, 2929, 2871, 1728, 1707, 1625, 1575, 1509, 1225, 1160.

HRMS (ESI): *m/z* = 587.3495 $[M+Na]^+$ (calc. for $C_{36}H_{49}FNaO_4$ *m/z* = 587.3507).

$[\alpha]^{25}_D$ = +77.406 ± 0.191 (c = 1.0, $CHCl_3$).

Synthesis of (*S*)-10e

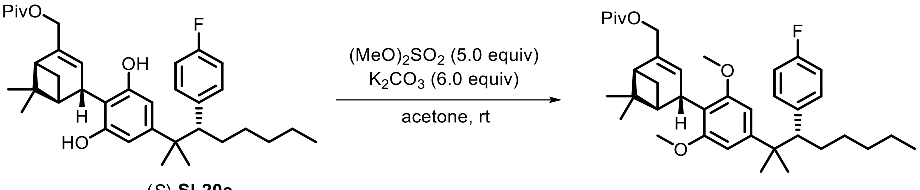

To a suspension of (*S*)-**SI-20e** (32.3 mg, 57.1 μmol, 1.0 equiv) and K_2CO_3 (47.4 mg. 343 μmol, 6.0 equiv) in acetone (1.0 mL) was added $(MeO)_2SO_2$ (27.1 μL. 286 μmol, 5.0 equiv) and the solution was stirred at rt overnight. The reaction mixture was diluted with Et_2O (5 mL), filtered and concentrated under reduced pressure. Purification by flash column chromatography (SiO_2; 0 – 5% EtOAc in hexanes) afforded the product as a white foam (28.9 mg, 85%).

^{1}H NMR (400 MHz, $CDCl_3$) δ 7.00 – 6.80 (m, 4H), 6.39 (s, 2H), 5.78 (dt, J = 2.8, 1.4 Hz, 1H), 4.62 – 4.55 (m, 1H), 4.54 – 4.48 (m, 1H), 4.01 (s, 1H), 3.69 (s, 6H), 2.71 (dd, J = 11.6, 3.4 Hz, 1H), 2.18 (dd, J = 7.2, 5.6 Hz, 2H), 2.05 (td, J = 6.0, 2.9 Hz, 1H), 1.72 (d, J = 7.4 Hz, 1H), 1.63 – 1.45 (m, 2H), 1.31 (s, 3H), 1.26 (s, 3H), 1.22 (s, 9H), 1.15 (s, 3H), 1.14 – 1.02 (m, 4H), 0.98 (s, 3H), 0.94 – 0.81 (m, 2H), 0.76 (t, J = 6.8 Hz, 3H).

^{13}C NMR (101 MHz, $CDCl_3$) δ 178.6, 161.5 (d, J = 243.7 Hz), 158.3, 148.3, 137.6 (d, J = 3.3 Hz), 137.4, 131.3 (d, J = 7.5 Hz), 126.4, 118.1, 114.2 (d, J = 20.8 Hz), 103.9, 67.6, 56.6, 55.9, 47.6, 44.0, 41.8, 41.1, 39.0, 37.7, 31.8, 29.5, 28.4, 28.0, 27.7, 27.5, 26.5, 24.4, 22.6, 21.2, 14.1.

^{19}F NMR (376 MHz, $CDCl_3$) δ -117.6.

IR (neat, ν_{max}/cm^{-1}): 2955, 2930, 2869, 1726, 1658, 1604, 1572, 1508, 1479, 1462, 1410, 1381, 1365, 1339, 1280, 1239, 1224, 1152, 11122, 1032, 1015, 957, 938, 907, 841, 770, 742, 702, 670, 546.

HRMS (ESI): *m/z* = 615.3808 [M+Na]$^+$ (calc. for $C_{38}H_{53}FNaO_4$ *m/z* = 615.3820).

[α]$^{25}_D$ = +50.994 ± 0.086 (c = 1.0, $CHCl_3$).

Synthesis of (*R*)-10e

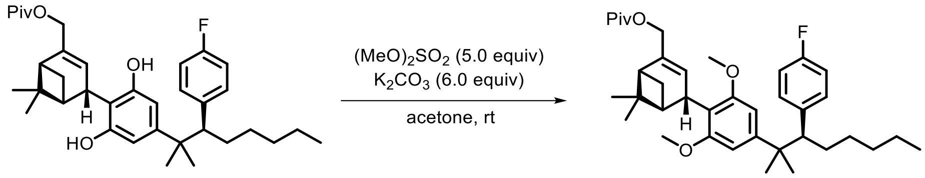

(*R*)-**SI-20e** (*R*)-**10e**

To a suspension of diol (*R*)-**SI-20e** (38.5 mg, 68.1 µmol, 1.0 equiv) and K_2CO_3 (56.5 mg. 409 µmol, 6.0 equiv) in acetone (1.0 mL) was added $(MeO)_2SO_2$ (32.3 µL. 341 µmol, 5.0 equiv) and the solution was stirred at rt overnight. The reaction mixture was diluted with Et_2O (5 mL), filtered and concentrated under reduced pressure. Purification by flash column chromatography (SiO_2; 0 – 5% EtOAc in hexanes) afforded the product as a white foam (35.5 mg, 88%).

^{1}H NMR (400 MHz, $CDCl_3$) δ 6.99 – 6.85 (m, 4H), 6.39 (s, 2H), 5.79 (dt, J = 2.9, 1.4 Hz, 1H), 4.62 – 4.56 (m, 1H), 4.55 – 4.48 (m, 1H), 4.01 (s, 1H), 3.69 (s, 6H), 2.71 (dd, J = 11.7, 3.4 Hz, 1H), 2.22 – 2.14 (m, 2H), 2.10 – 2.02 (m, 1H), 1.70 (d, J = 7.8 Hz, 1H), 1.63 – 1.45 (m, 2H), 1.31 (s, 3H), 1.26 (s, 3H), 1.22 (s, 9H), 1.15 (s, 3H), 1.14 – 1.02 (m, 4H), 0.99 (s, 3H), 0.94 – 0.81 (m, 2H), 0.76 (t, J = 6.8 Hz, 3H).

^{13}C NMR (101 MHz, $CDCl_3$) δ 178.6, 161.5 (d, J = 243.7 Hz), 158.3, 148.3, 137.6 (d, J = 3.2 Hz), 137.4, 131.3 (d, J = 7.3 Hz), 126.5, 118.1, 114.2 (d, J = 20.8 Hz), 103.9, 67.6, 56.6, 55.9, 47.6, 44.0, 41.8, 41.1, 39.0, 37.7, 31.8, 29.5, 28.4, 28.0, 27.7, 27.5, 26.5, 24.4, 22.6, 21.2, 14.1.

^{19}F NMR (376 MHz, $CDCl_3$) δ -117.6.

IR (neat, ν_{max}/cm^{-1}): 2931, 2869, 1726, 1658, 1604, 1572, 1508, 1479, 14623, 1410, 1397, 1381, 1364, 1339, 1280, 1239, 1224, 1151, 1121, 1042, 1031, 1015, 957, 938, 907, 841, 769, 757, 702, 562, 547, 525.

HRMS (ESI): *m/z* = 615.3818 $[M+Na]^+$ (calc. for $C_{38}H_{53}FNaO_4$ *m/z* = 615.3820).

$[\alpha]^{25}_D$ = +96.771 ± 0.079 (c = 1.0, $CHCl_3$).

Synthesis of (*S*)-6e

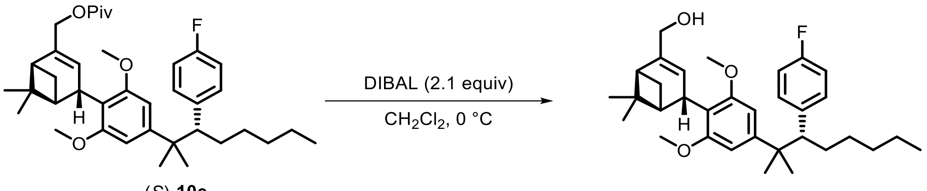

(*S*)-**10e** (*S*)-**6e**

To a solution of (*S*)-**10e** (28.9 mg, 48.7 µmol, 1.0 equiv) in CH_2Cl_2 (0.55 mL) was added DIBAL (102 µL, 102 µmol, 2.1 equiv) at 0 °C. After stirring the reaction for 15 minutes at 0 °C, sat. aq. NH_4Cl (3 mL) was added, the layers were separated and the aqueous layer was extracted with CH_2Cl_2 (3 × 5 mL). The combined organic extracts were dried over $MgSO_4$ and concentrated under reduced pressure. Purification by flash column chromatography (SiO_2; 5 – 20% EtOAc in hexanes) afforded the product as a colorless oil (21.3 mg, 86%).

^{1}H NMR (400 MHz, $CDCl_3$) δ 7.02 – 6.84 (m, 4H), 6.39 (s, 2H), 5.71 (dt, J = 3.0, 1.4 Hz, 1H), 4.08 (s, 2H), 4.01 (t, J = 2.3 Hz, 1H), 3.69 (s, 6H), 2.71 (dd, J = 11.5, 3.5 Hz, 1H), 2.27 – 2.17 (m, 2H), 2.10 – 2.03 (m, 1H), 1.72 (d, J = 6.9 Hz, 1H), 1.62 – 1.45 (m, 2H), 1.32 (s, 3H), 1.26 (s, 3H), 1.15 (s, 3H), 1.13 – 1.01 (m, 4H), 0.98 (s, 3H), 0.94 – 0.82 (m, 2H), 0.76 (t, J = 6.6 Hz, 3H).

^{13}C NMR (101 MHz, $CDCl_3$) δ 161.5 (d, J = 243.7 Hz), 158.3, 148.3, 142.1, 137.6 (d, J = 3.3 Hz), 131.3 (d, J = 7.5 Hz), 123.8, 118.2, 114.2 (d, J = 20.8 Hz), 104.0, 66.8, 56.6, 56.0, 47.6, 44.0, 41.8, 41.0, 37.6, 31.8, 29.5, 28.4, 28.0, 28.0, 26.4, 24.4, 22.6, 21.2, 14.1.

^{19}F NMR (376 MHz, $CDCl_3$) δ -117.6.

IR (neat, ν_{max}/cm^{-1}): 3378, 2928, 2863, 1655, 1604, 1572, 1508, 1461, 1410, 1380, 1364, 1340, 1301, 1238, 1224, 1184, 1160, 1120, 1049, 1014, 986, 908, 840, 743, 701, 669, 563, 547, 525.

HRMS (ESI): m/z = 531.3238 $[M+Na]^+$ (calc. for $C_{33}H_{45}FNaO_3$ m/z = 531.3245).

$[\alpha]^{25}_D$ = +43.736 ± 0.139 (c = 1.0, $CDCl_3$).

Synthesis of (*R*)-6e

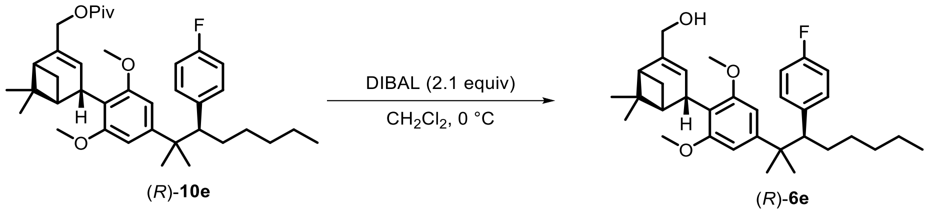

To a solution of (*R*)-**10e** (35.5 mg, 59.8 µmol, 1.0 equiv) in CH_2Cl_2 (0.55 mL) was added DIBAL (126 µL, 126 µmol, 2.1 equiv) at 0 °C. After stirring the reaction for 15 minutes at 0 °C, sat. aq. NH_4Cl (3 mL) was added, the layers were separated and the aqueous layer was extracted with CH_2Cl_2 (3 × 5 mL). The combined organic extracts were dried over $MgSO_4$ and concentrated under reduced pressure. Purification by flash column chromatography (SiO_2; 5 – 20% EtOAc in hexanes) afforded the product as a colorless oil (29.1 mg, 96%).

^{1}H NMR (400 MHz, $CDCl_3$) δ 6.98 – 6.83 (m, 4H), 6.39 (s, 2H), 5.72 (dt, J = 2.9, 1.4 Hz, 1H), 4.08 (s, 2H), 4.01 (s, 1H), 3.70 (s, 6H), 2.71 (dd, J = 11.7, 3.3 Hz, 1H), 2.26 – 2.18 (m, 2H), 2.11 – 2.05 (m, 1H), 1.70 (d, J = 7.6 Hz, 1H), 1.63 – 1.45 (m, 2H), 1.32 (s, 3H), 1.26 (s, 3H), 1.15 (s, 3H), 1.14 – 1.02 (m, 4H), 0.98 (s, 3H), 0.94 – 0.80 (m, 2H), 0.76 (t, J = 6.7 Hz, 3H).

^{13}C NMR (101 MHz, $CDCl_3$) δ 161.5 (d, J = 243.7 Hz), 158.3, 148.3, 142.1, 137.6 (d, J = 3.2 Hz), 131.3 (d, J = 7.5 Hz), 123.7, 118.2, 114.2 (d, J = 20.8 Hz), 103.9, 66.8, 56.6, 56.0, 47.6, 44.0, 41.8, 41.0, 37.6, 31.8, 29.5, 28.4, 28.0, 28.0, 26.4, 24.4, 22.6, 21.2, 14.1.

^{19}F NMR (376 MHz, $CDCl_3$) δ -117.6.

IR (neat, ν_{max}/cm^{-1}): 3379, 2929, 2864, 1655, 1604, 1572, 1508, 1462, 1410, 1381, 1364, 1340, 1301, 1238, 1224, 1184, 1160, 1120, 1049, 1014, 986, 908, 841, 742, 701, 670, 563, 525.

HRMS (ESI): m/z = 531.3248 $[M+Na]^+$ (calc. for $C_{33}H_{45}FNaO_3$ m/z = 531.3245).

$[\alpha]^{25}_{D}$ = +109.233 ± 0.100(c = 1.0, $CHCl_3$).

Synthesis of (*S*)-SI-20f

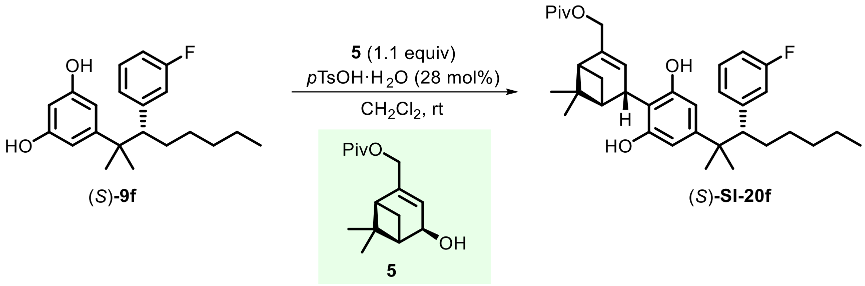

To a solution of resorcinol (*S*)-**9f** and *p*TsOH·H_2O (4.1 mg, 21.6 µmol, 0.28 equiv) in CH_2Cl_2 (3.0 mL) was added **5** (21.4 mg, 84.8 µmol, 1.1 equiv) and the solution was stirred at rt for 30 minutes. The reaction was stopped by addition of sat. aq. $NaHCO_3$ (3 mL), the layers were separated and the aqueous layer was extracted with Et_2O (3 × 5 mL). The combined organic extracts were dried over $MgSO_4$ and concentrated under reduced pressure. Purification by flash column chromatography (SiO_2; 5 – 15% EtOAc in hexanes) afforded the product as a colourless foam (33.6 mg, 77%).

Note: Allylic alcohol **5** was prepared according to our previously published procedure.[27]

^{1}H NMR (400 MHz, $CDCl_3$) δ 7.17 (td, *J* = 8.0, 6.2 Hz, 1H), 6.91 – 6.82 (m, 2H), 6.75 (dt, *J* = 10.4, 2.1 Hz, 1H), 6.33 (s, 2H), 6.03 (dt, *J* = 3.2, 1.6 Hz, 1H), 5.72 (bs, 2H), 4.65 (dt, J = 13.5, 1.7 Hz, 1H), 4.52 (dt, J = 13.5, 2.0 Hz, 1H), 4.06 – 4.01 (m, 1H), 2.71 (dd, *J* = 12.0, 2.9 Hz, 1H), 2.39 (dt, *J* = 9.7, 5.6 Hz, 1H), 2.35 – 2.28 (m, 2H), 1.62 – 1.53 (m, 1H), 1.51 (d, *J* = 9.7 Hz, 1H), 1.45 – 1.38 (m, 1H), 1.35 (s, 3H), 1.24 (s, 9H), 1.20 (s, 3H), 1.16 – 1.05 (m, 4H), 1.04 (s, 3H), 0.99 (s, 3H), 0.96 – 0.82 (m, 2H), 0.75 (t, J = 6.7 Hz, 3H).

^{13}C NMR (101 MHz, $CDCl_3$) δ 178.7, 162.5 (d, J = 244.0 Hz), 155.0, 149.8, 149.6, 145.0 (d, J = 6.7 Hz), 128.7 (d, J = 8.3 Hz), 126.0, 120.4, 116.6 (d, J = 20.8 Hz), 113.0 (d, J = 21.0 Hz), 112.0, 107.3, 66.6, 56.9, 47.5, 44.3, 41.2, 41.1, 39.1, 37.9, 31.8, 29.4, 28.9, 28.1, 28.0, 27.4, 26.0, 23.3, 22.5, 20.9, 14.1.

^{19}F NMR (376 MHz, $CDCl_3$) δ -114.7.

IR (neat, ν_{max}/cm^{-1}): 3451, 2956, 2931, 2870, 1728, 1707, 1624, 1613, 1586, 1577, 1510, 1480, 1464, 1445, 1429, 1398, 1384, 1366, 1328, 1283, 1230, 1159, 1111, 1073, 1040, 1027, 963, 935, 916, 876, 783., 758, 724, 700, 672, 587, 569, 545, 521.

HRMS (ESI): *m/z* = 587.3499 $[M+Na]^+$ (calc. for $C_{36}H_{49}FNaO_4$ *m/z* = 587.3507).

$[\alpha]^{25}_D$ = +14.014 ± 0.249 (c = 1.0, $CHCl_3$).

Synthesis of (*R*)-SI-20f

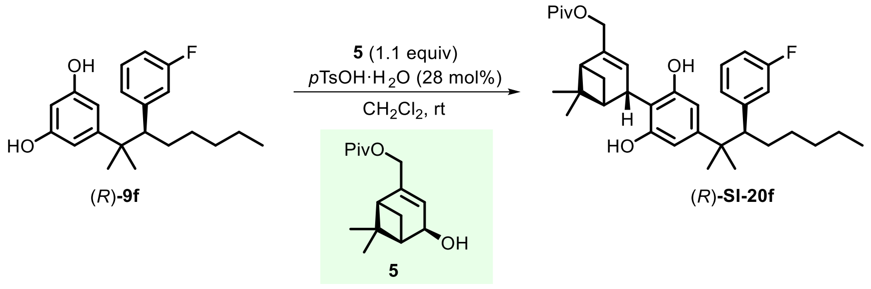

To a solution of resorcinol (*R*)-**9f** (25.5 mg, 77.1 µmol, 1.0 equiv) and *p*TsOH·H_2O (4.1 mg, 21.6 µmol, 0.28 equiv) in CH_2Cl_2 (3.0 mL) was added **5** (21.4 mg, 84.8 µmol, 1.1 equiv) and the solution was stirred at rt for 30 minutes. The reaction was stopped by addition of sat. aq. $NaHCO_3$ (3 mL), the layers were separated and the aqueous layer was extracted with Et_2O (3 × 5 mL). The combined organic extracts were dried over $MgSO_4$ and concentrated under reduced pressure. Purification by flash column chromatography (SiO_2; 5 – 15% EtOAc in hexanes) afforded the product as a colourless foam (31.6 mg, 73%).

Note: Allylic alcohol **5** was prepared according to our previously published procedure.[27]

^{1}H NMR (400 MHz, $CDCl_3$) δ 7.23 – 7.14 (m, 1H), 6.94 – 6.82 (m, 2H), 6.76 (ddd, *J* = 10.7, 2.6, 1.6 Hz, 1H), 6.33 (s, 2H), 6.03 (dt, J = 3.2, 1.6 Hz, 1H), 5.73 (bs, 2H), 4.68 – 4.61 (m, 1H), 4.52 (ddd, *J* = 13.5, 2.3, 1.6 Hz, 1H), 4.03 (s, 1H), 2.71 (dd, *J* = 12.0, 2.9 Hz, 1H), 2.43 – 2.35 (m, 1H), 2.33 – 2.29 (m, 2H), 1.61 – 1.52 (m, 1H), 1.50 (d, *J* = 9.7 Hz, 1H), 1.45 – 1.37 (m, 1H), 1.35 (s, 3H), 1.24 (s, 9H), 1.20 (s, 3H), 1.19 – 1.05 (m, 4H), 1.04 (s, 3H), 0.99 (s, 3H), 0.96 – 0.80 (m, 2H), 0.75 (t, *J* = 6.6 Hz, 3H).

^{13}C NMR (101 MHz, $CDCl_3$) δ 178.7, 162.5 (d, J = 244.1 Hz), 155.0, 149.9, 149.6, 145.0 (d, J = 6.7 Hz), 128.7 (d, J = 8.2 Hz), 126.0, 120.4, 116.6 (d, J = 20.7 Hz), 113.0 (d, J = 21.0 Hz), 112.0, 107.2, 66.6, 56.9, 47.5, 44.3, 41.2, 41.1, 39.1, 37.9, 31.8, 29.4, 28.9, 28.2, 28.0, 27.4, 26.0, 23.2, 22.5, 20.9, 14.1.

^{19}F NMR (376 MHz, $CDCl_3$) δ -114.7.

IR (neat, ν_{max}/cm^{-1}): 3449, 2956, 2930, 2870, 1726, 1707, 1625, 1613, 1586, 1577, 1480, 1464, 1445, 1428, 1398, 1384, 1366, 1337, 1326, 1283, 1229, 1157, 1111, 1073, 1040, 1028, 962, 935, 916, 876, 783, 756, 724, 700, 668, 587, 569, 545, 521.

HRMS (ESI): *m/z* = 587.3498 $[M+Na]^+$ (calc. for $C_{36}H_{49}FNaO_4$ *m/z* = 587.3507).

$[\alpha]^{25}_D$ = +69.607 ± 0.141 (c = 1.0, $CHCl_3$).

Synthesis of (*S*)-10f

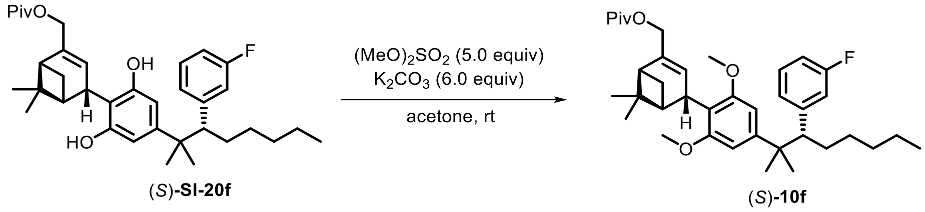

(*S*)-**SI-20f** (*S*)-**10f**

To a suspension of (*S*)-**SI-20f** (33.6 mg, 59.4 µmol, 1.0 equiv) and K_2CO_3 (49.3 mg. 357 µmol, 6.0 equiv) in acetone (0.7 mL) was added $(MeO)_2SO_2$ (28.2 µL, 298 µmol, 5.0 equiv) and the solution was stirred at rt overnight. The reaction mixture was diluted with Et_2O (5 mL), filtered and concentrated under reduced pressure. Purification by flash column chromatography (SiO_2; 0 – 5% EtOAc in hexanes) afforded the product as a white foam (28.3 mg, 80%).

^{1}H NMR (400 MHz, $CDCl_3$) δ 7.21 – 7.12 (m, 1H), 6.91 – 6.84 (m, 1H), 6.79 (d, J = 7.7 Hz, 1H), 6.75 – 6.66 (m, 1H), 6.40 (s, 2H), 5.78 (dt, J = 2.8, 1.4 Hz, 1H), 4.61 – 4.55 (m, 1H), 4.55 – 4.48 (m, 1H), 4.01 (s, 1H), 3.69 (s, 6H), 2.72 (dd, J = 11.8, 3.2 Hz, 1H), 2.23 – 2.14 (m, 2H), 2.08 – 2.02 (m, 1H), 1.72 (d, J = 7.6 Hz, 1H), 1.62 – 1.44 (m, 2H), 1.30 (s, 3H), 1.28 (s, 3H), 1.22 (s, 9H), 1.16 (s, 3H), 1.15 – 1.03 (m, 4H), 0.98 (s, 3H), 0.94 – 0.84 (m, 2H), 0.76 (t, J = 6.8 Hz, 3H).

^{13}C NMR (101 MHz, $CDCl_3$) δ 178.6, 162.5 (d, J = 244.1 Hz), 158.3, 148.3, 145.0 (d, J = 6.7 Hz), 137.4, 128.6 (d, J = 8.3 Hz), 126.4, 126.0, 118.2, 116.6 (d, J = 21.2 Hz), 112.9 (d, J = 21.0 Hz), 103.9, 67.6, 57.2, 56.0, 47.6, 44.0, 41.8, 41.1, 39.0, 37.7, 31.8, 29.4, 28.5, 28.0, 27.7, 27.5, 26.5, 24.3, 22.6, 21.2, 14.1.

^{19}F NMR (376 MHz, $CDCl_3$) δ -114.8.

IR (neat, ν_{max}/cm^{-1}): 2955, 2930, 2869, 1726, 1656, 1604, 1586, 1573, 1480, 1455, 1410, 1397, 1382, 1365, 1338, 1280, 1239, 1149, 1120, 1032, 957, 928, 904, 871, 852, 832, 782, 724, 699, 585, 520.

HRMS (ESI): *m/z* = 615.3806 $[M+Na]^+$ (calc. for $C_{38}H_{53}FNaO_4$ *m/z* = 615.3820).

$[\alpha]^{25}_D$ = +25.224 ± 0.173 (c = 1.0, $CHCl_3$).

Synthesis of (*R*)-10f

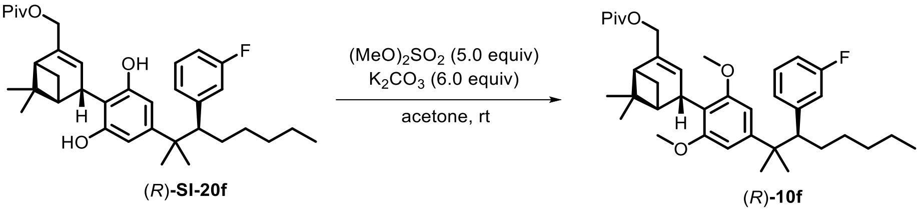

To a suspension of diol (*R*)-**SI-20f** (31.6 mg, 55.9 µmol, 1.0 equiv) and K_2CO_3 (46.4 mg. 336 µmol, 6.0 equiv) in acetone (0.7 mL) was added $(MeO)_2SO_2$ (26.5 µL, 280 µmol, 5.0 equiv) and the solution was stirred at rt overnight. The reaction mixture was diluted with Et_2O (5 mL), filtered and concentrated under reduced pressure. Purification by flash column chromatography (SiO_2; 0 – 5% EtOAc in hexanes) afforded the product as a white foam (30.2 mg, 91%).

^{1}H NMR (400 MHz, $CDCl_3$) δ 7.20 – 7.12 (m, 1H), 6.91 – 6.83 (m, 1H), 6.80 (d, J = 7.9 Hz, 1H), 6.71 (d, J = 10.5 Hz, 1H), 6.41 (s, 2H), 5.79 (s, 1H), 4.61 – 4.55 (m, 1H), 4.55 – 4.48 (m, 1H), 4.01 (s, 1H), 3.70 (s, 6H), 2.72 (dd, J = 11.7, 3.2 Hz, 1H), 2.23 – 2.13 (m, 2H), 2.09 – 2.01 (m, 1H), 1.70 (d, J = 7.5 Hz, 1H), 1.63 – 1.43 (m, 2H), 1.31 (s, 3H), 1.28 (s, 3H), 1.22 (s, 9H), 1.17 (s, 3H), 1.15 – 1.04 (m, 4H), 0.98 (s, 3H), 0.94 – 0.85 (m, 2H), 0.76 (t, J = 6.4 Hz, 3H).

^{13}C NMR (101 MHz, $CDCl_3$) δ 178.6, 162.5 (d, J = 244.1 Hz), 158.3, 148.2, 145.0 (d, J = 6.8 Hz), 137.4, 128.7 (d, J = 8.2 Hz), 126.4, 126.0, 118.2, 116.6 (d, J = 20.0 Hz), 112.9 (d, J = 21.0 Hz), 103.9, 67.6, 57.2, 55.9, 47.6, 44.0, 41.8, 41.1, 39.0, 37.7, 31.8, 29.4, 28.6, 28.0, 27.7, 27.5, 26.5, 24.3, 22.6, 21.2, 14.1.

^{19}F NMR (376 MHz, $CDCl_3$) δ -114.8.

IR (neat, ν_{max}/cm^{-1}): 2955, 2930, 2869, 1725, 1657, 1635, 1604, 1586, 1573, 1508, 1480, 1463, 1450, 1410, 1382, 1365, 1337, 1281, 1260, 1239, 1150, 1120, 1031, 957, 928, 904, 871, 832, 782, 756, 669, 654, 584, 521.

HRMS (ESI): m/z = 615.3804 $[M+Na]^+$ (calc. for $C_{38}H_{53}FNaO_4$ m/z = 615.3820).

$[\alpha]^{25}_D$ = +73.779 ± 0.081 (c = 1.0, $CHCl_3$).

Synthesis of (*S*)-6f

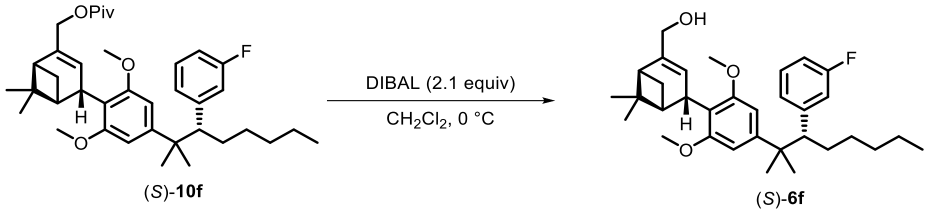

To a solution of (*S*)-**10f** (30.2 mg, 50.1 µmol, 1.0 equiv) in CH_2Cl_2 (0.55 mL) was added DIBAL (107 µL, 107 µmol, 2.1 equiv) at 0 °C. After stirring the reaction for 15 minutes at 0 °C, sat. aq. NH_4Cl (3 mL) was added, the layers were separated and the aqueous layer was extracted with CH_2Cl_2 (3 × 5 mL). The combined organic extracts were dried over $MgSO_4$ and concentrated under reduced pressure. Purification by flash column chromatography (SiO_2; 5 – 20% EtOAc in hexanes) afforded the product as a colourless oil (21.8 mg, 84%).

^{1}H NMR (400 MHz, $CDCl_3$) δ 7.22 – 7.10 (m, 1H), 6.93 – 6.83 (m, 1H), 6.79 (d, J = 7.7 Hz, 1H), 6.71 (d, J = 10.5 Hz, 1H), 6.41 (s, 2H), 5.71 (dt, J = 2.7, 1.4 Hz, 1H), 4.08 (s, 2H), 4.03 – 3.99 (m, 1H), 3.70 (s, 6H), 2.72 (dd, J = 11.7, 3.2 Hz, 1H), 2.26 – 2.19 (m, 2H), 2.09 – 2.05 (m, 1H), 1.72 (d, J = 7.9 Hz, 1H), 1.65 – 1.41 (m, 2H), 1.32 (s, 3H), 1.28 (s, 3H), 1.16 (s, 3H), 1.14 – 1.02 (m, 4H), 0.98 (s, 3H), 0.96 – 0.82 (m, 2H), 0.76 (t, J = 6.9 Hz, 3H).

^{13}C NMR (101 MHz, $CDCl_3$) δ 162.4 (d, J = 244.1 Hz), 158.2, 148.1, 144.8 (d, J = 6.7 Hz), 142.0, 128.5 (d, J = 8.2 Hz), 125.9, 123.6, 118.2, 116.5 (d, J = 21.1 Hz), 112.8 (d, J = 21.0 Hz), 103.8, 66.7, 57.1, 55.9, 47.5, 43.8, 41.6, 40.9, 37.5, 31.7, 29.3, 28.4, 27.8, 27.8, 26.3, 24.2, 22.4, 21.1, 14.0.

^{19}F NMR (376 MHz, $CDCl_3$) δ -114.8.

IR (neat, ν_{max}/cm^{-1}): 3381, 2928, 2862, 1656, 1605, 1586, 1572, 1487, 1463, 1449, 1410, 1380, 1364, 1340, 1302, 1238, 1184, 1160, 1119, 1049, 986, 927, 871, 832, 782, 724, 699, 671, 654, 578.

HRMS (ESI): m/z = 531.3245 $[M+Na]^+$ (calc. for $C_{33}H_{45}FNaO_3$ m/z = 531.3245).

$[\alpha]^{25}_D$ = +55.004 ± 0.309 (c = 1.0, $CHCl_3$).

Synthesis of (*R*)-6f

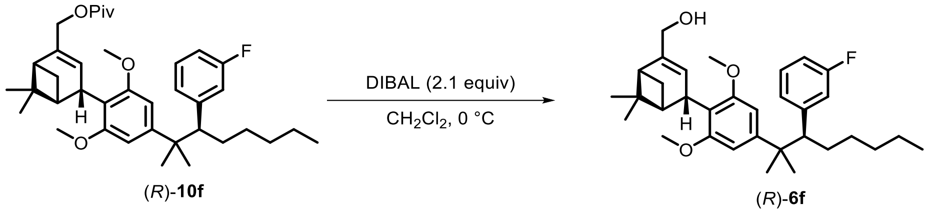

To a solution of (*R*)-**10f** (27.3 mg, 46.0 µmol, 1.0 equiv) in CH_2Cl_2 (0.55 mL) was added DIBAL (97.0 µL, 97.0 µmol, 2.1 equiv) at 0 °C. After stirring the reaction for 15 minutes at 0 °C, sat. aq. NH_4Cl (3 mL) was added, the layers were separated and the aqueous layer was extracted with CH_2Cl_2 (3 × 5 mL). The combined organic extracts were dried over $MgSO_4$ and concentrated under reduced pressure. Purification by flash column chromatography (SiO_2; 5 – 20% EtOAc in hexanes) afforded the product as a colourless oil (21.5 mg, 92%).

^{1}H NMR (400 MHz, $CDCl_3$) δ 7.19 – 7.13 (m, 1H), 6.91 – 6.83 (m, 1H), 6.79 (d, J = 7.7 Hz, 1H), 6.71 (d, J = 10.2 Hz, 1H), 6.41 (s, 2H), 5.72 (dt, J = 2.8, 1.4 Hz, 1H), 4.08 (d, J = 4.4 Hz, 2H), 4.03 – 3.96 (m, 1H), 3.70 (s, 6H), 2.72 (dd, J = 11.8, 3.2 Hz, 1H), 2.27 – 2.18 (m, 2H), 2.11 – 2.05 (m, 1H), 1.70 (d, J = 7.7 Hz, 1H), 1.61 – 1.40 (m, 2H), 1.32 (s, 3H), 1.27 (s, 3H), 1.16 (s, 3H), 1.15 – 1.02 (m, 4H), 0.98 (s, 3H), 0.96 – 0.81 (m, 2H), 0.76 (t, J = 6.5 Hz, 3H).

^{13}C NMR (101 MHz, $CDCl_3$) δ 162.5 (d, J = 244.1 Hz), 158.3, 148.2, 145.0 (d, J = 6.7 Hz), 142.1, 128.7 (d, J = 8.3 Hz), 126.0, 123.7, 118.3, 116.6 (d, J = 21.2 Hz), 112.9 (d, J = 21.0 Hz), 103.9, 66.8, 57.2, 56.0, 47.6, 44.0, 41.8, 41.0, 37.6, 31.8, 29.4, 28.6, 28.0, 28.0, 26.4, 24.3, 22.6, 21.2, 14.1.

^{19}F NMR (376 MHz, $CDCl_3$) δ -114.8.

IR (neat, ν_{max}/cm^{-1}): 3373, 2927, 2862, 1661, 1604, 1586, 1572, 1508, 1487, 1463, 1450, 1410, 1380, 1364, 1341, 1302, 1239, 1183, 1160, 1119, 1072, 1049, 987, 927, 871, 832, 782, 724, 699, 671, 578, 522.

HRMS (ESI): m/z = 531.3245 $[M+Na]^+$ (calc. for $C_{33}H_{45}FNaO_3$ m/z = 531.3245).

$[\alpha]^{25}_D$ = +107.650 ± 0.171 (c = 1.0, $CHCl_3$).

NMR Spectra

^{1}H NMR (400 MHz, $CDCl_3$) of **SI-3.**

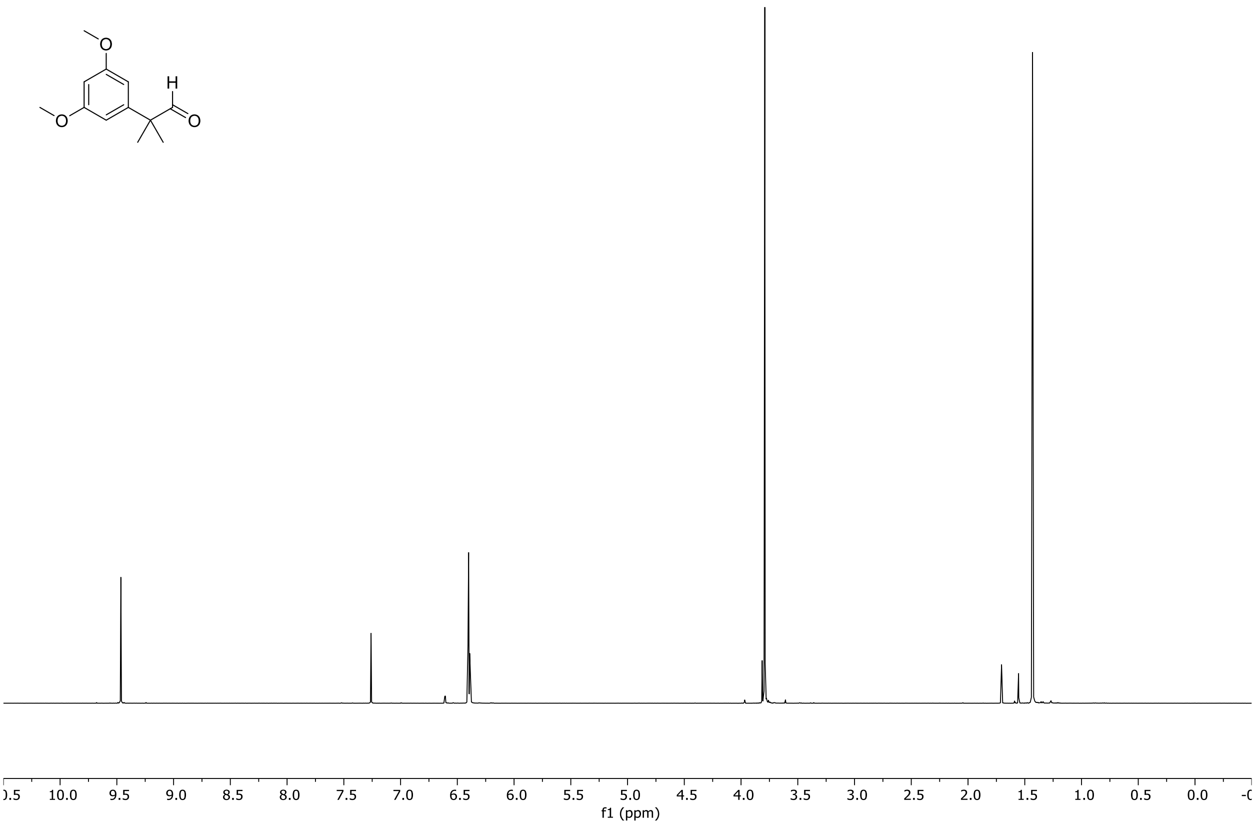

^{1}H NMR (400 MHz, $CDCl_3$) of **SI-2**

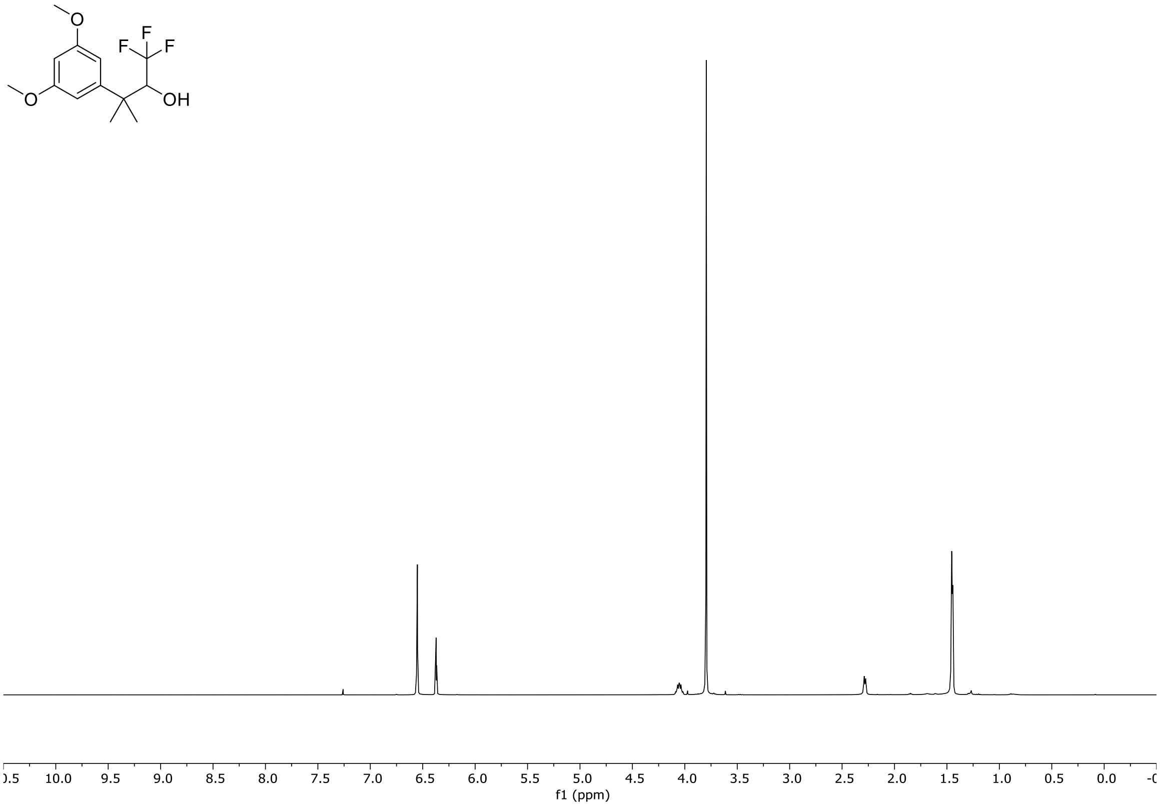

^{13}C NMR (101 MHz, $CDCl_3$) of **SI-2**

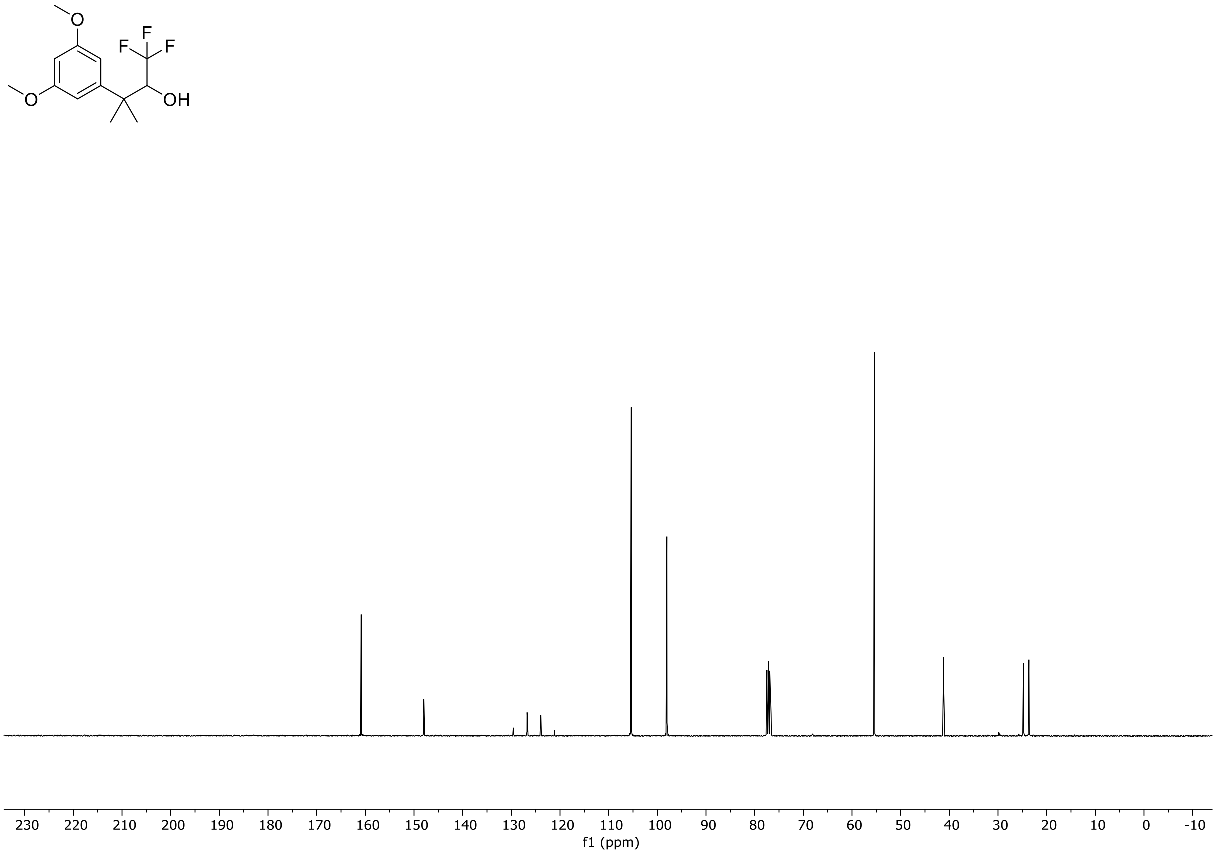

^{19}F NMR (377 MHz, $CDCl_3$) of **SI-2**

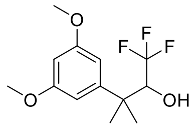

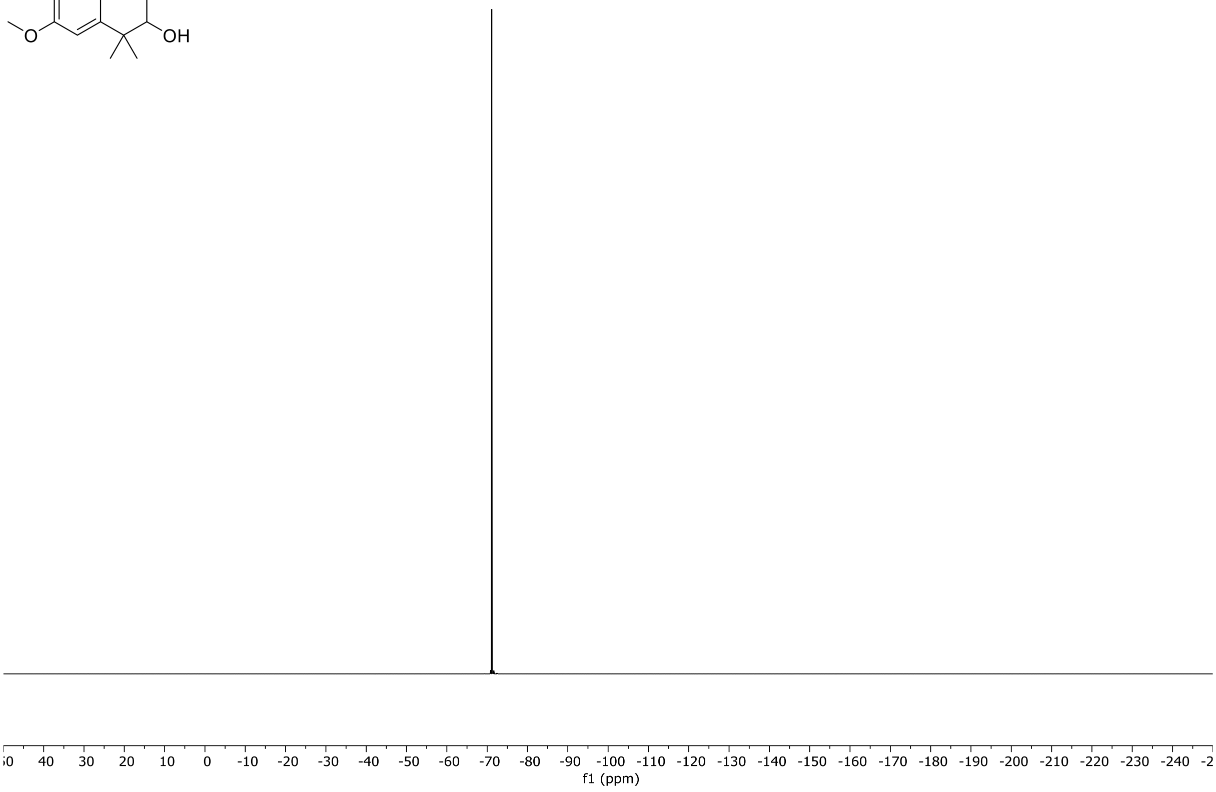

^{1}H NMR (400 MHz, $CDCl_3$) of **3**

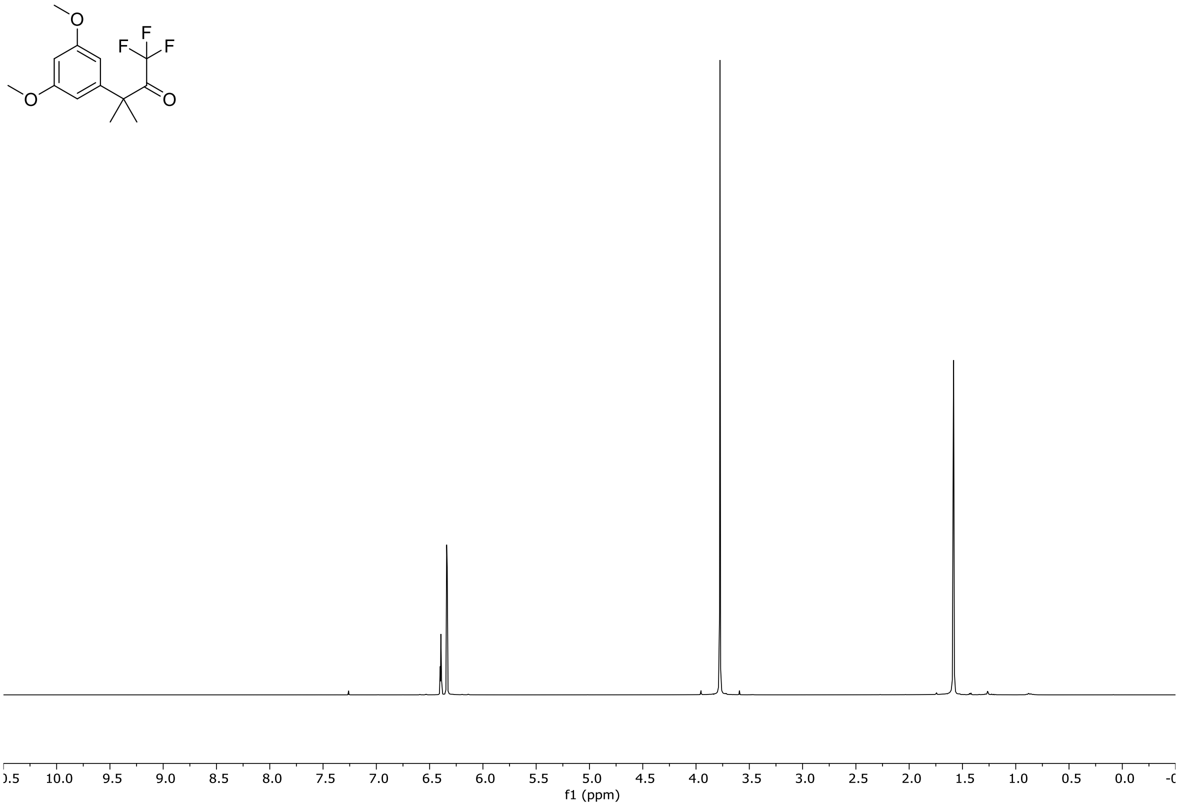

^{13}C NMR (101 MHz, $CDCl_3$) of **3**

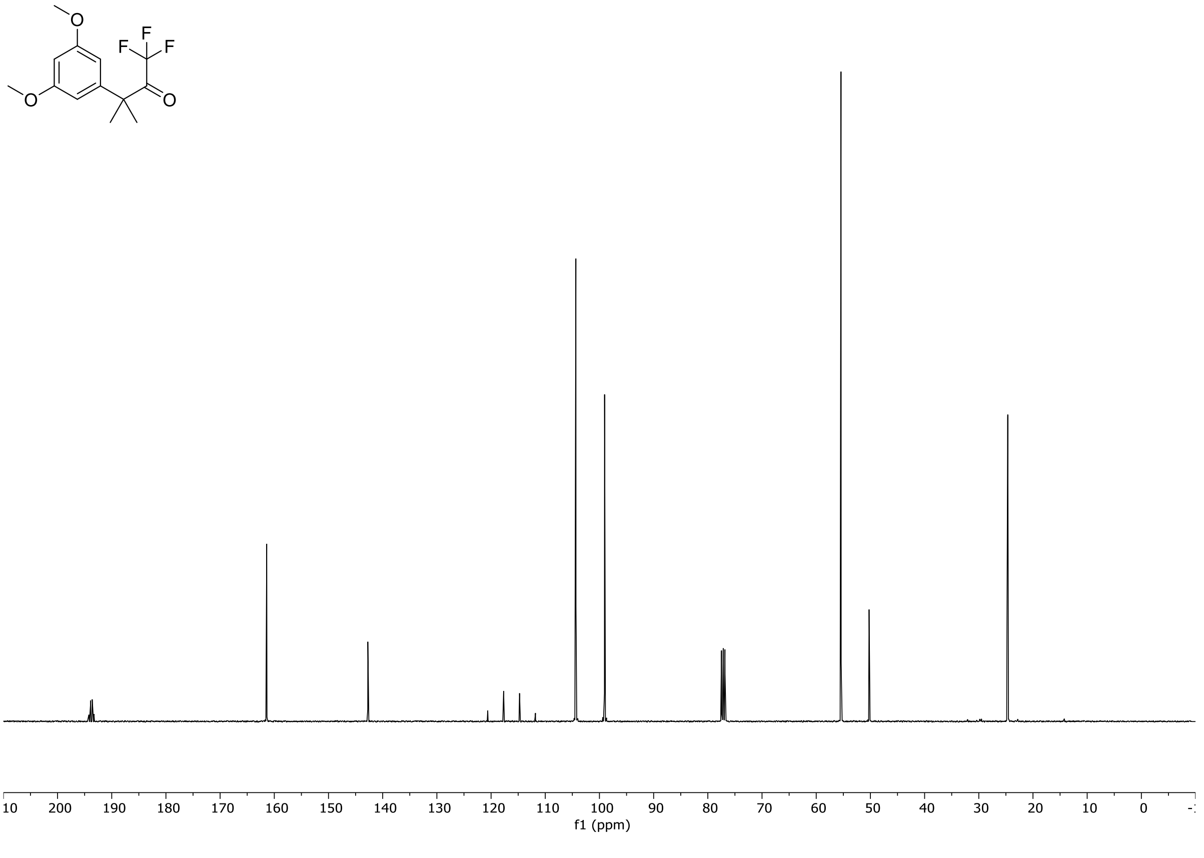

^{19}F NMR (377 MHz, $CDCl_3$) of **3**

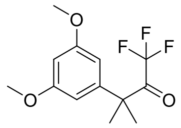

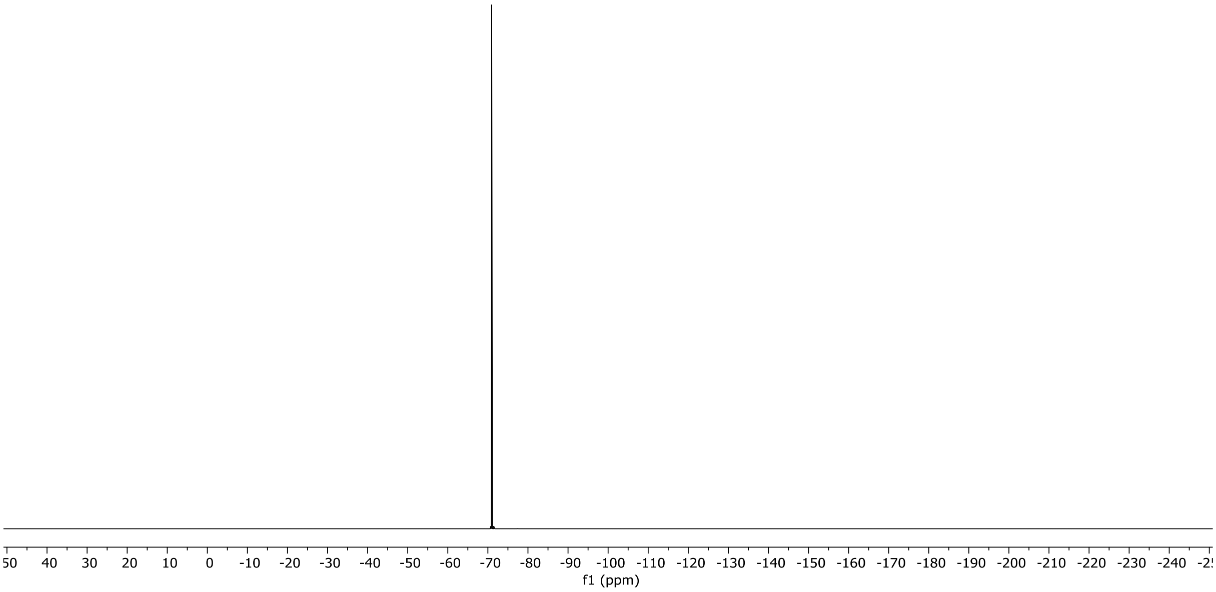

^{1}H NMR (500 MHz, $CDCl_3$) of ***(E)*-SI-3**

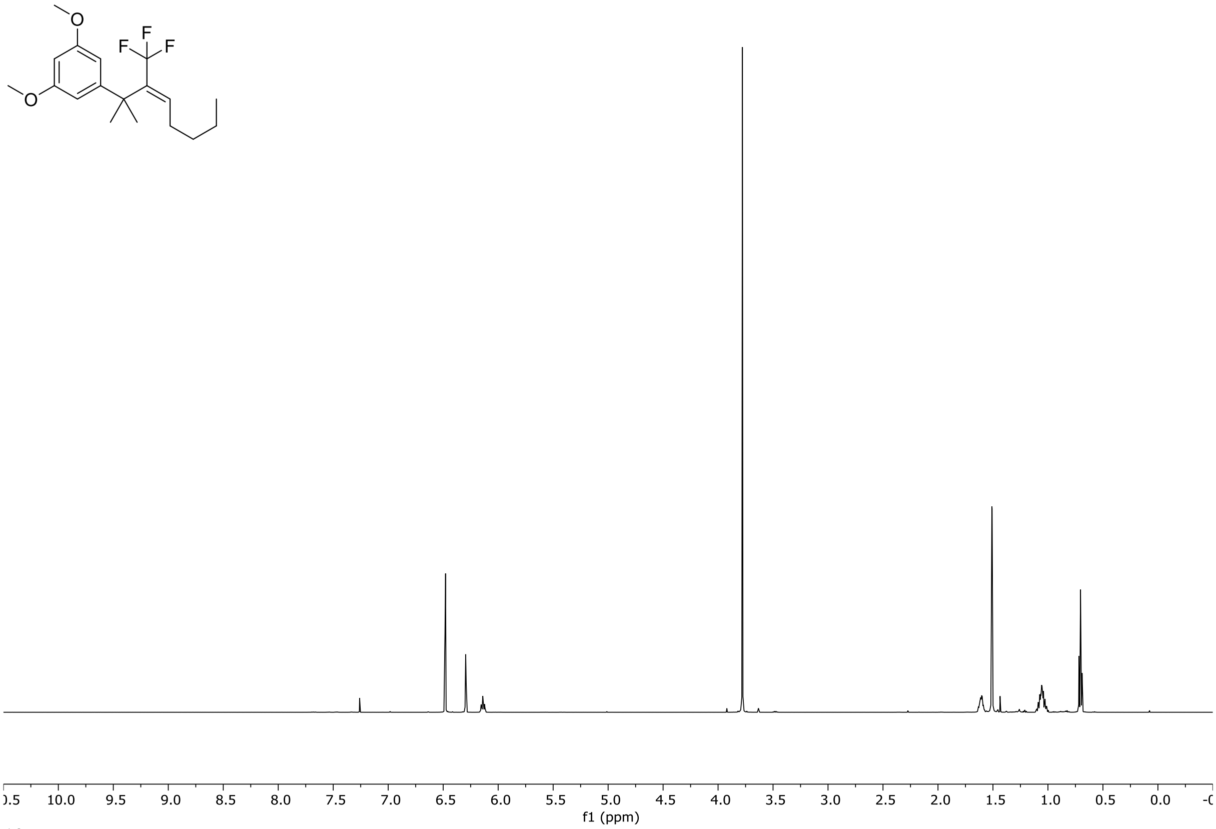

^{13}C NMR (126 MHz, $CDCl_3$) of ***(E)*-SI-3**

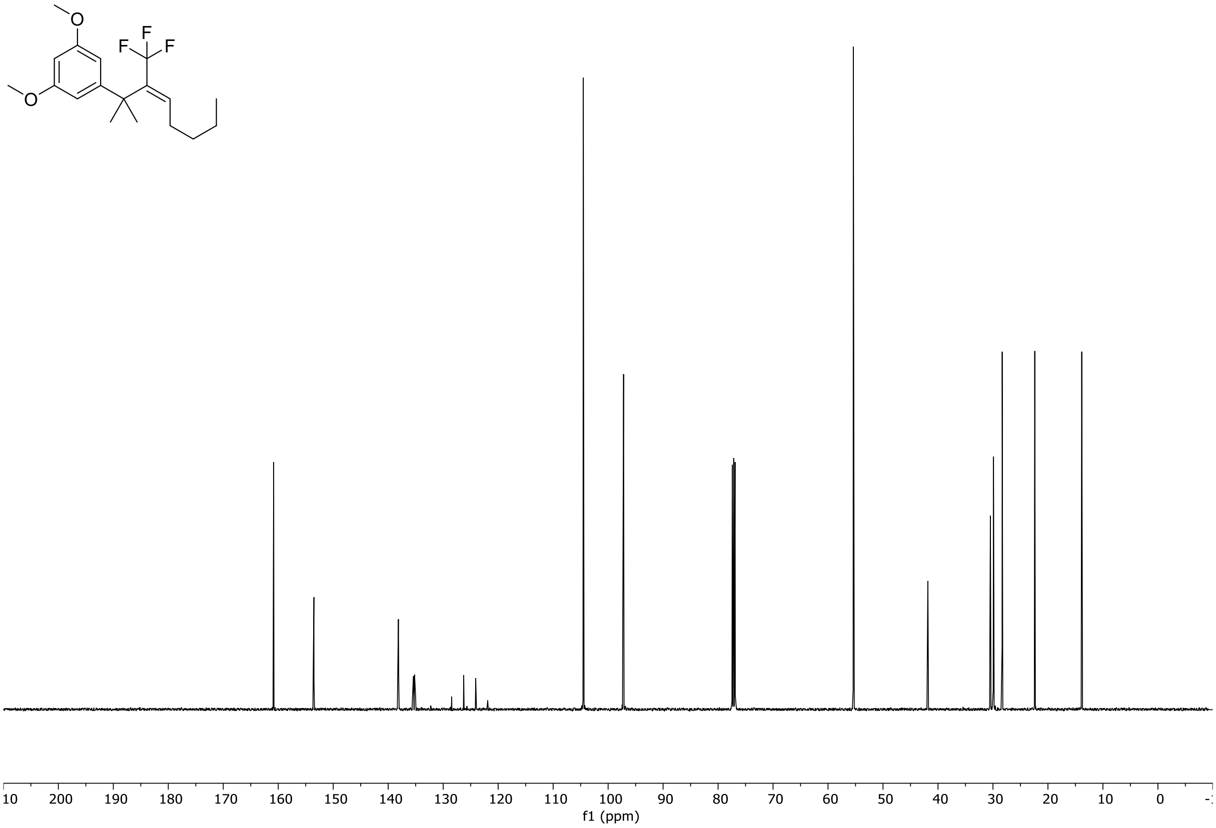

^{19}F NMR (471 MHz, $CDCl_3$) of ***(E)*-SI-3**

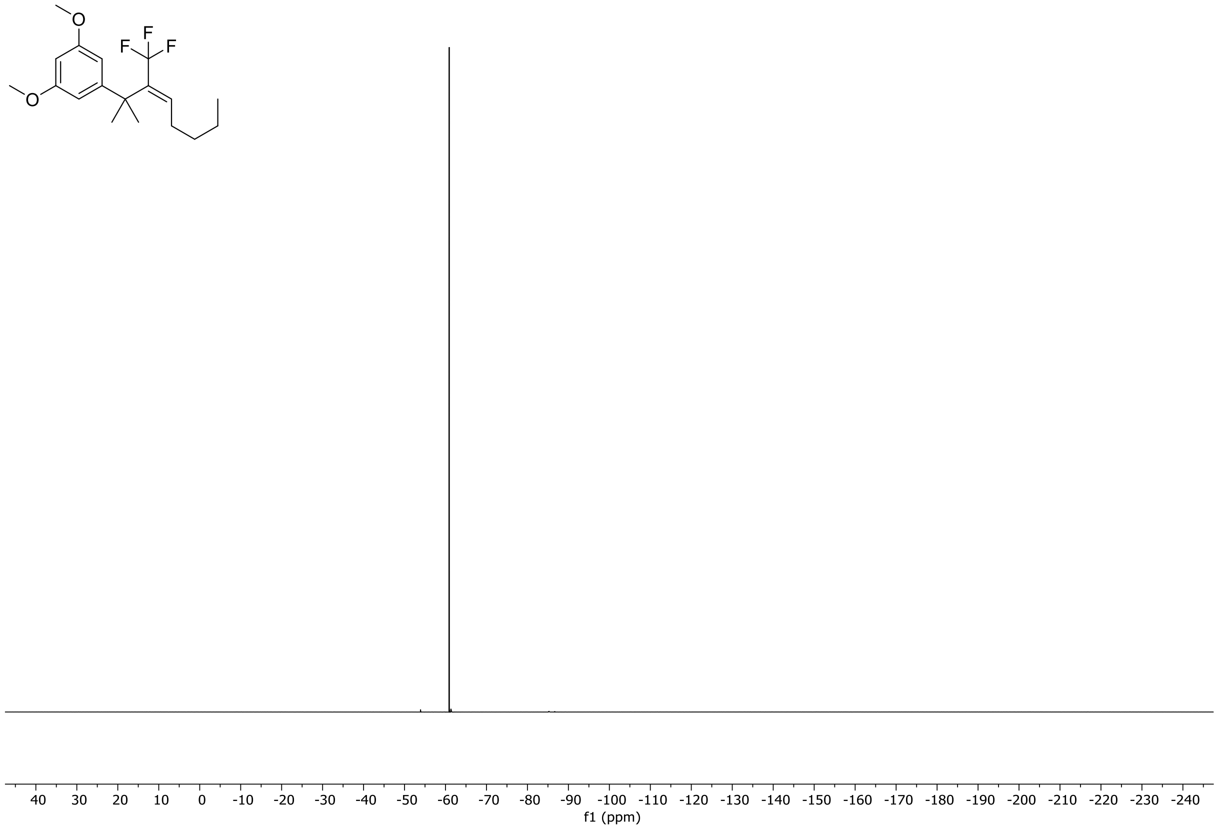

^{1}H NMR (500 MHz, $CDCl_3$) of ***(Z)*-SI-3**

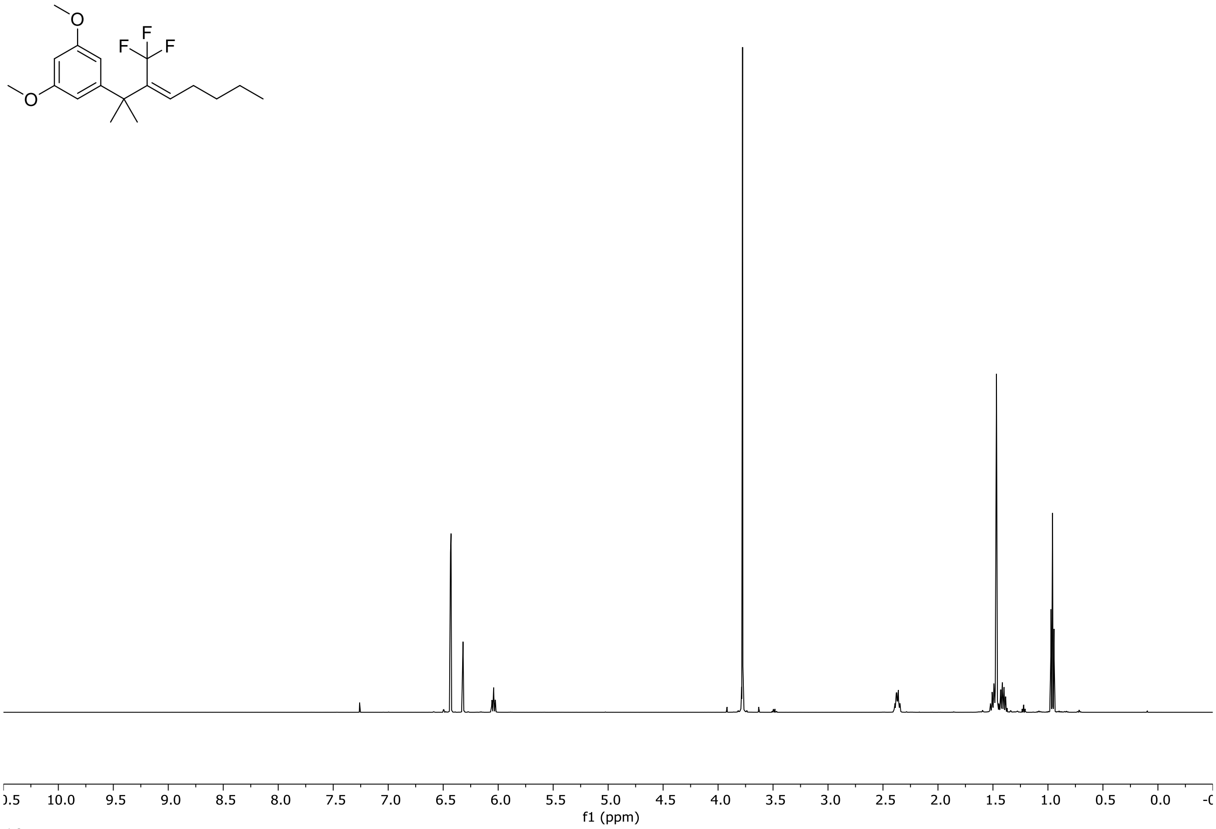

^{13}C NMR (126 MHz, $CDCl_3$) of ***(Z)*-SI-3**

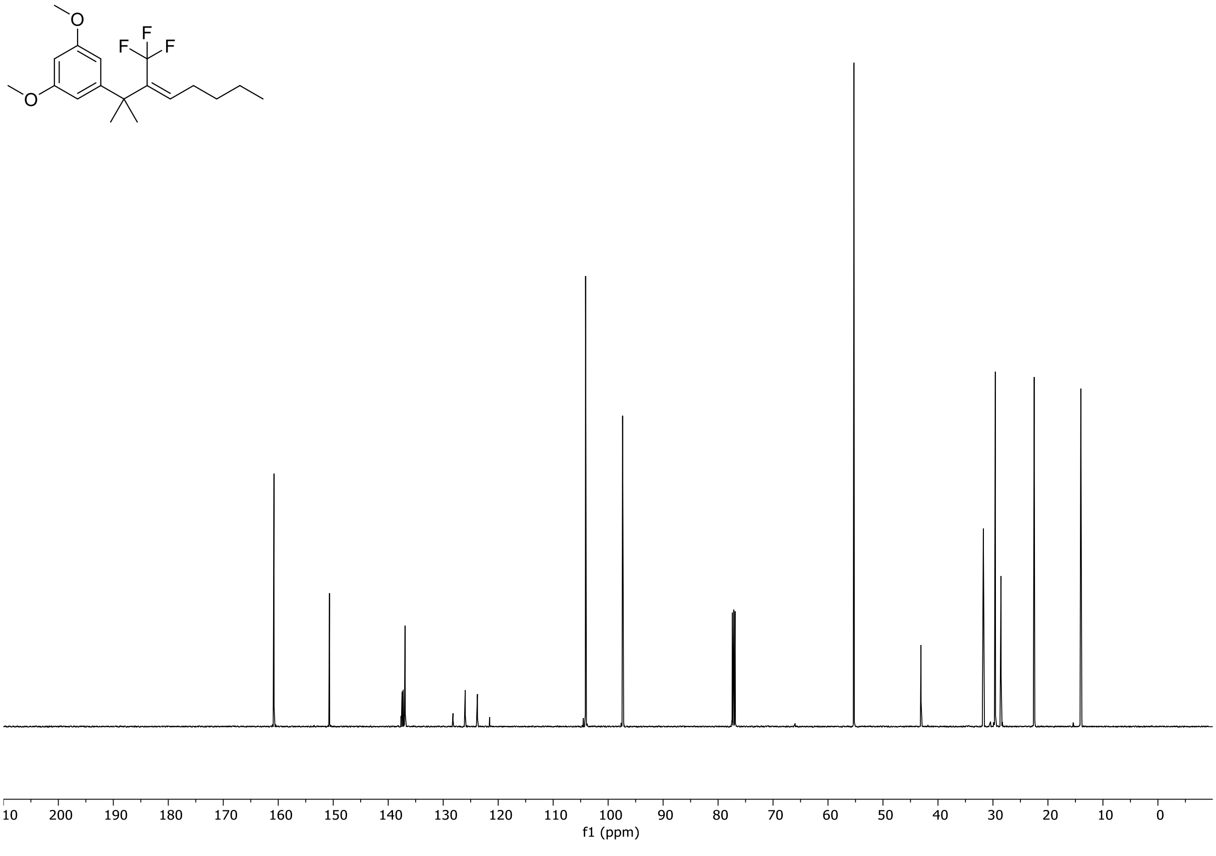

^{19}F NMR (471 MHz, $CDCl_3$) of ***(Z)*-SI-3**

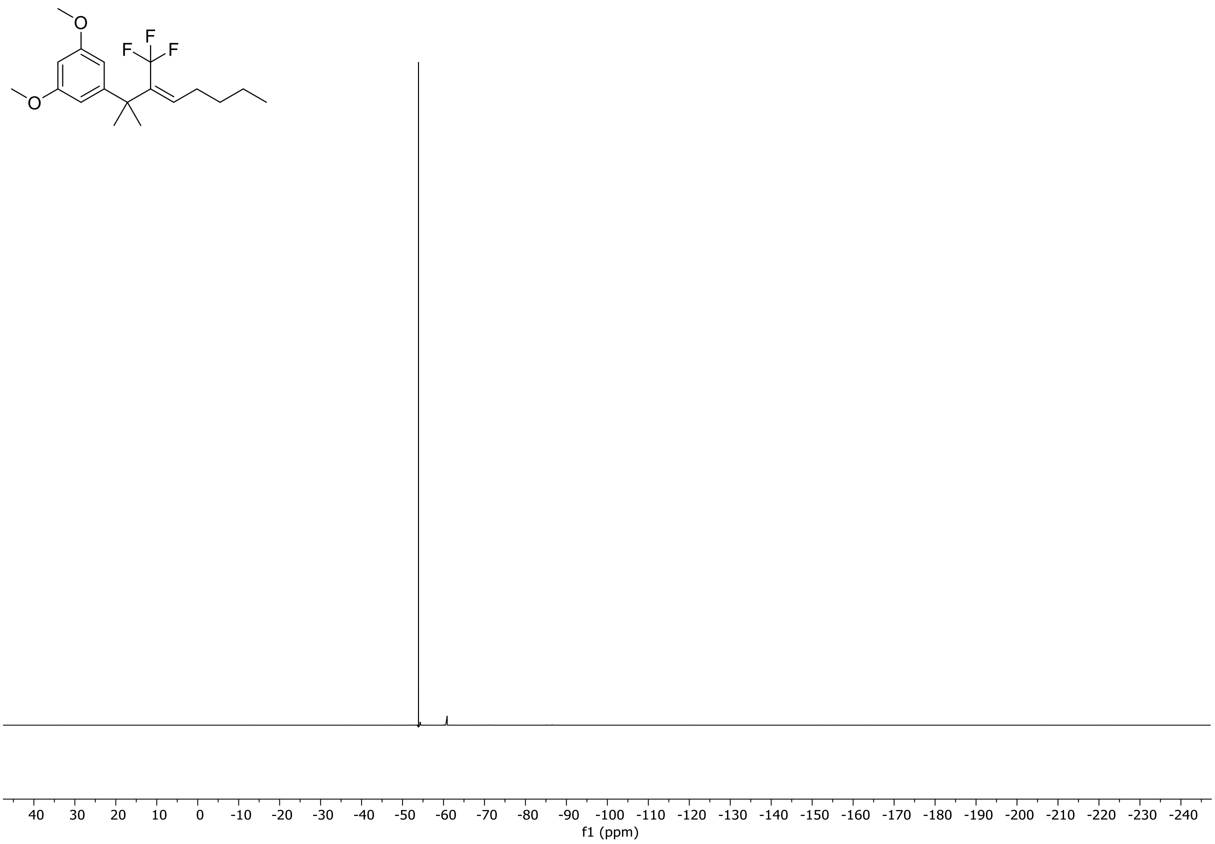

^{1}H NMR (500 MHz, $CDCl_3$) of (+)-(*R*)-**4** **&** (–)-(*S*)-**4**

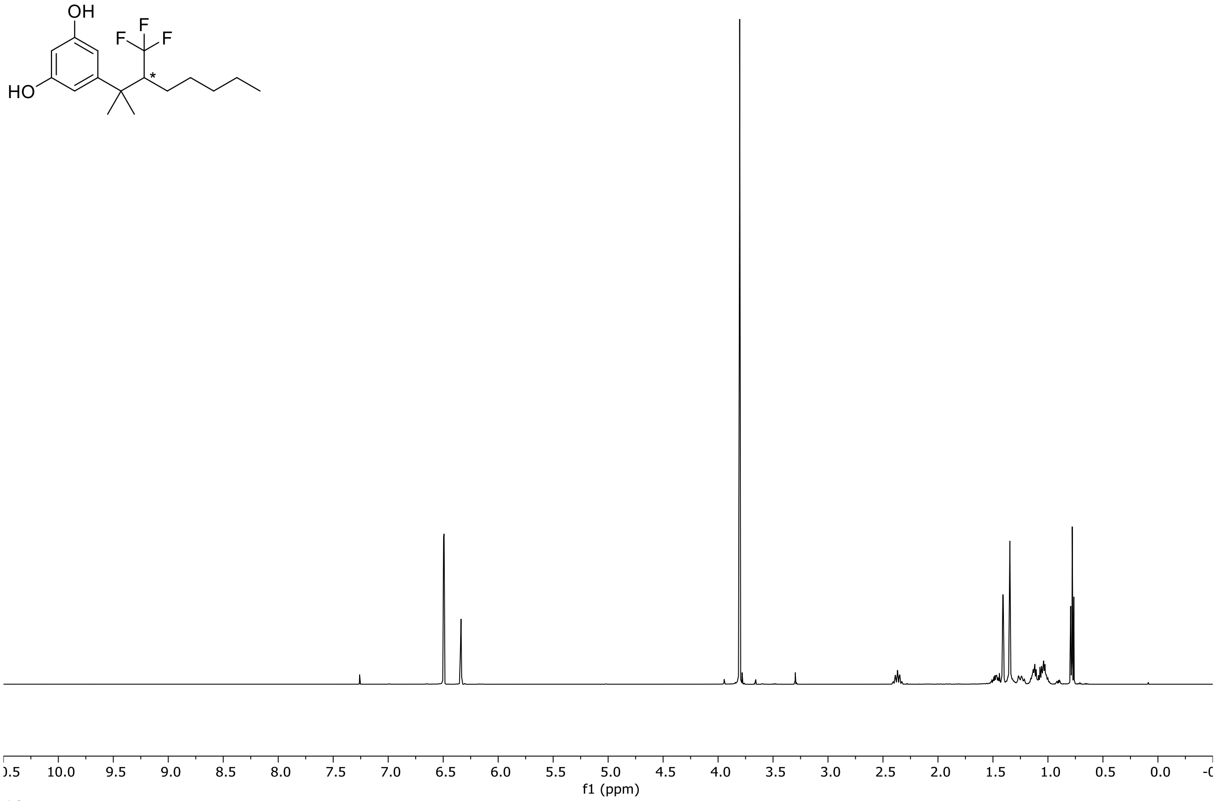

^{13}C NMR (126 MHz, $CDCl_3$) of (+)-(*R*)-**4** **&** (–)-(*S*)-**4**

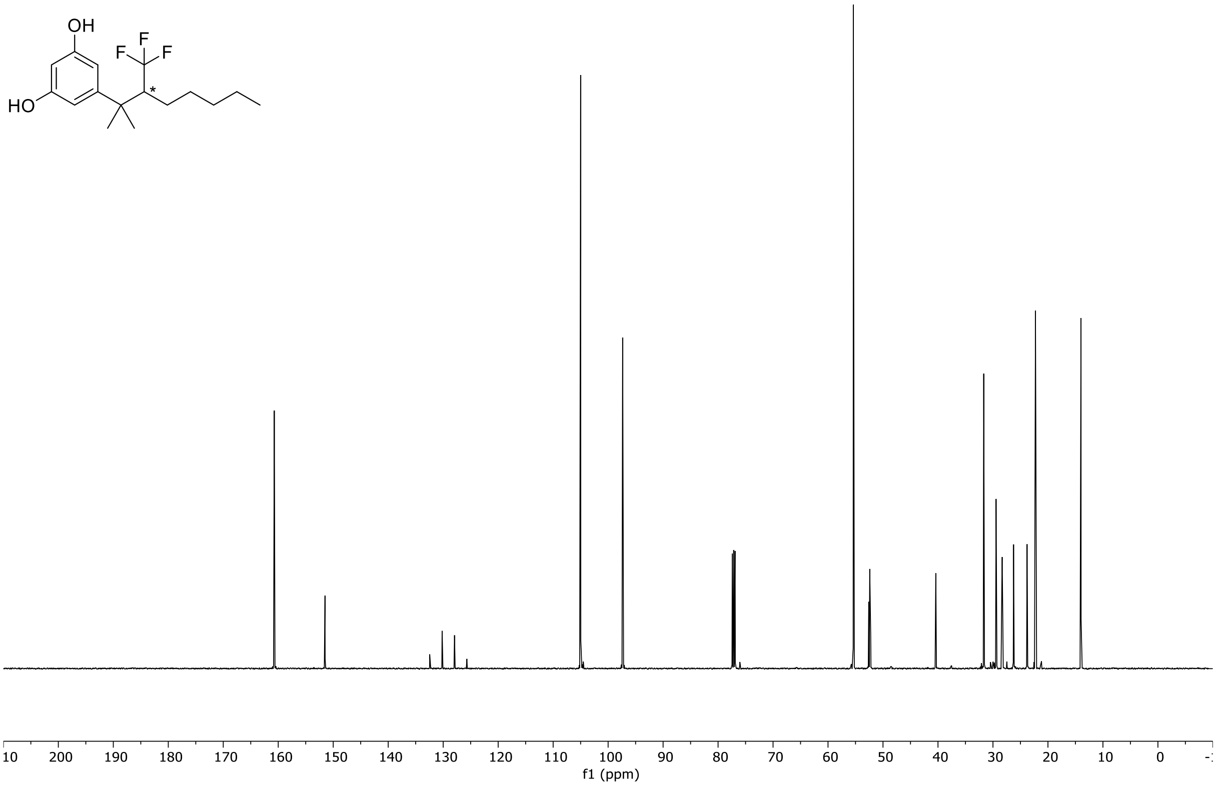

^{19}F NMR (471 MHz, $CDCl_3$) of (+)-(*R*)-**4** **&** (–)-(*S*)-**4**

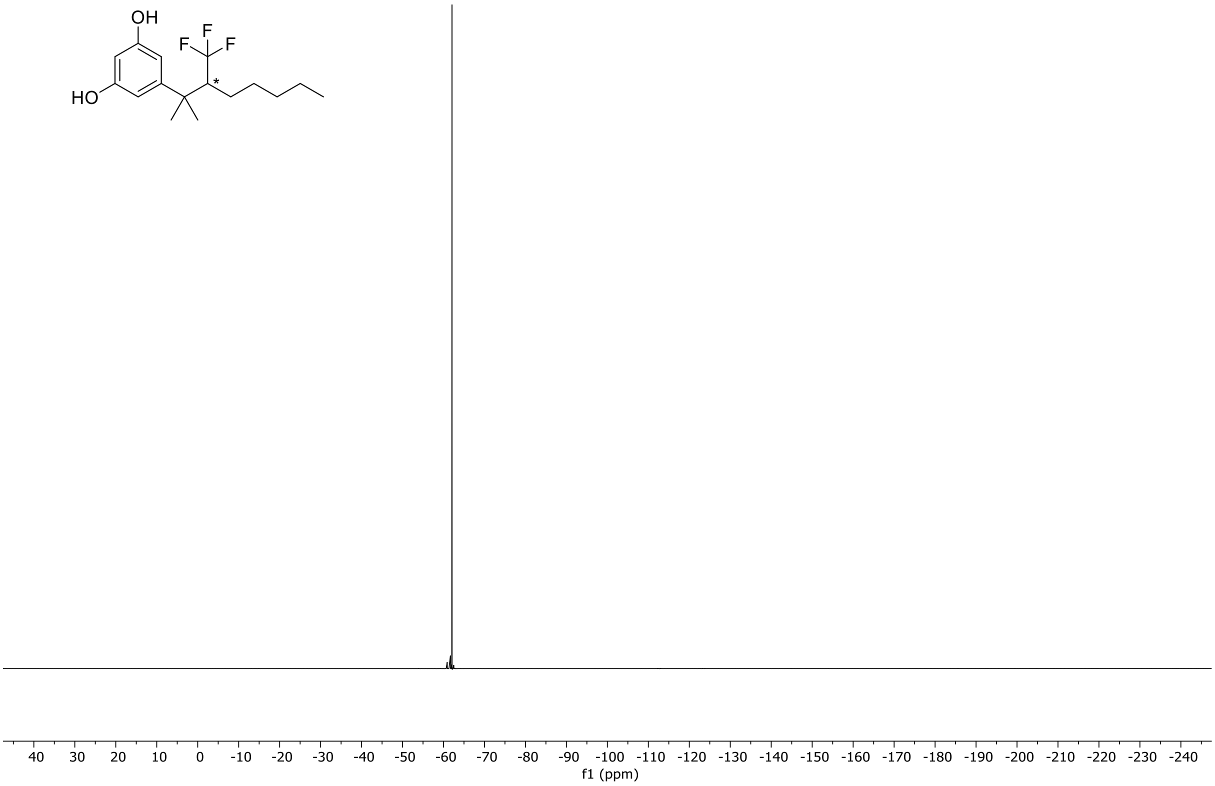

^{1}H NMR (400 MHz, $CDCl_3$) of **SI-11**

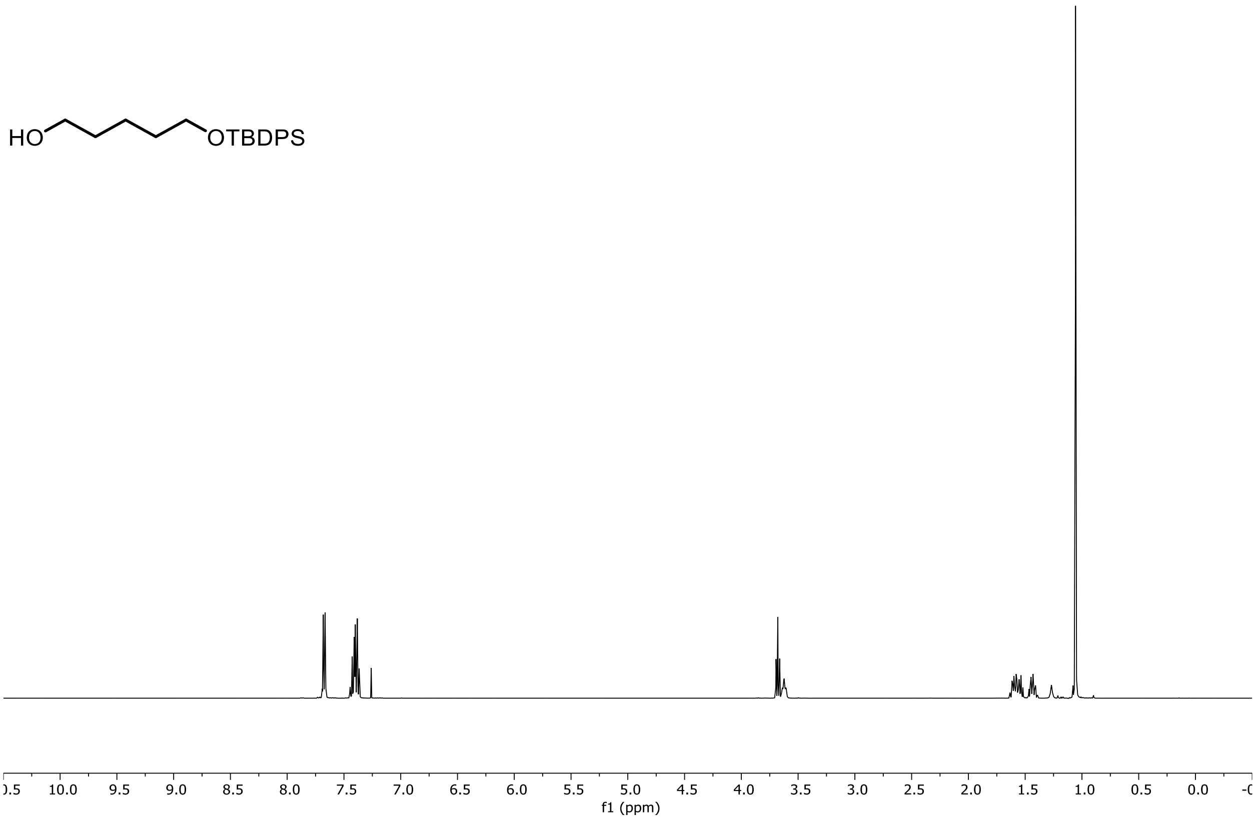

^{1}H NMR (400 MHz, $CDCl_3$) of **SI-12**

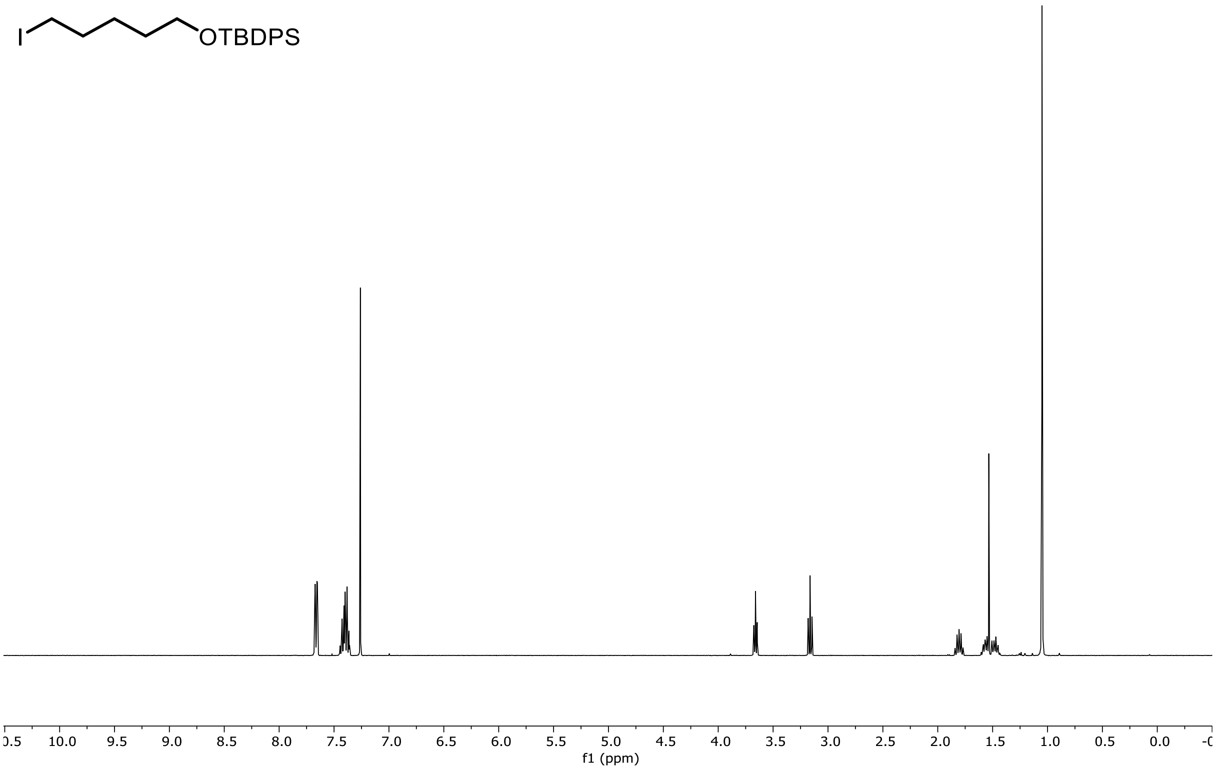

^{13}C NMR (101 MHz, $CDCl_3$) of **SI-12**

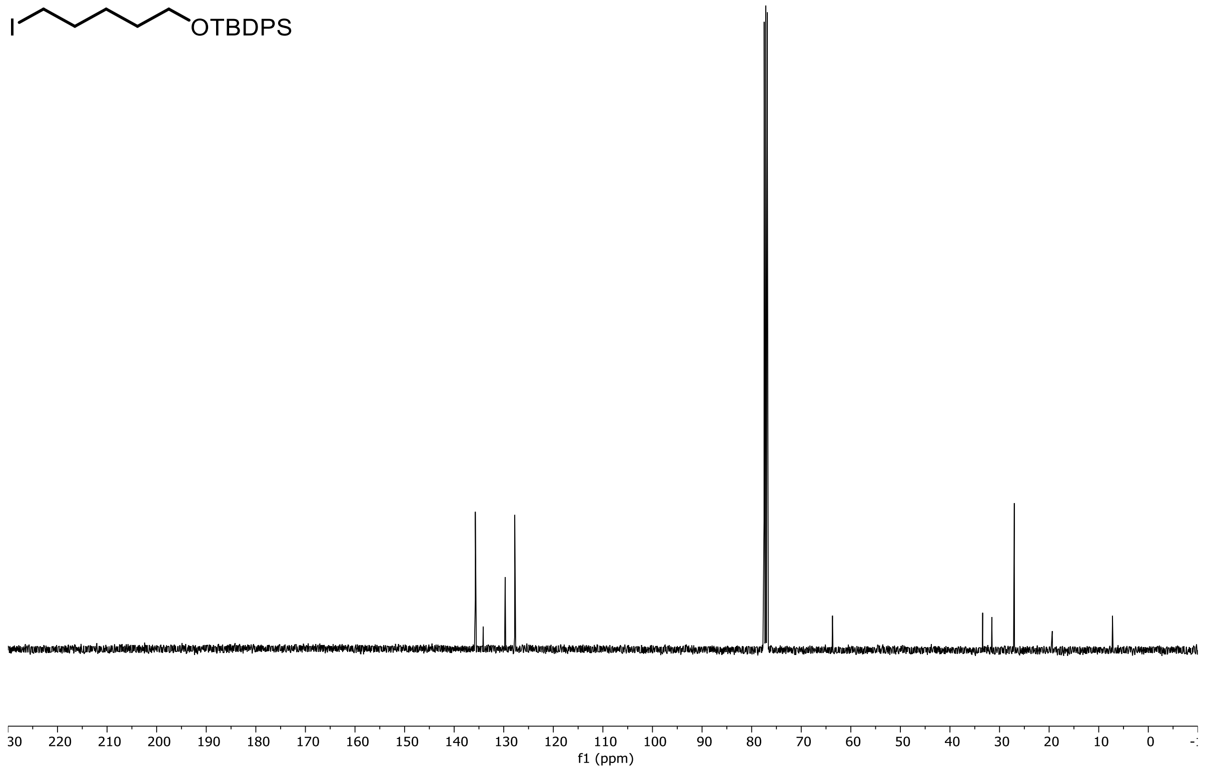

^{1}H NMR (400 MHz, $CDCl_3$) of **SI-5**

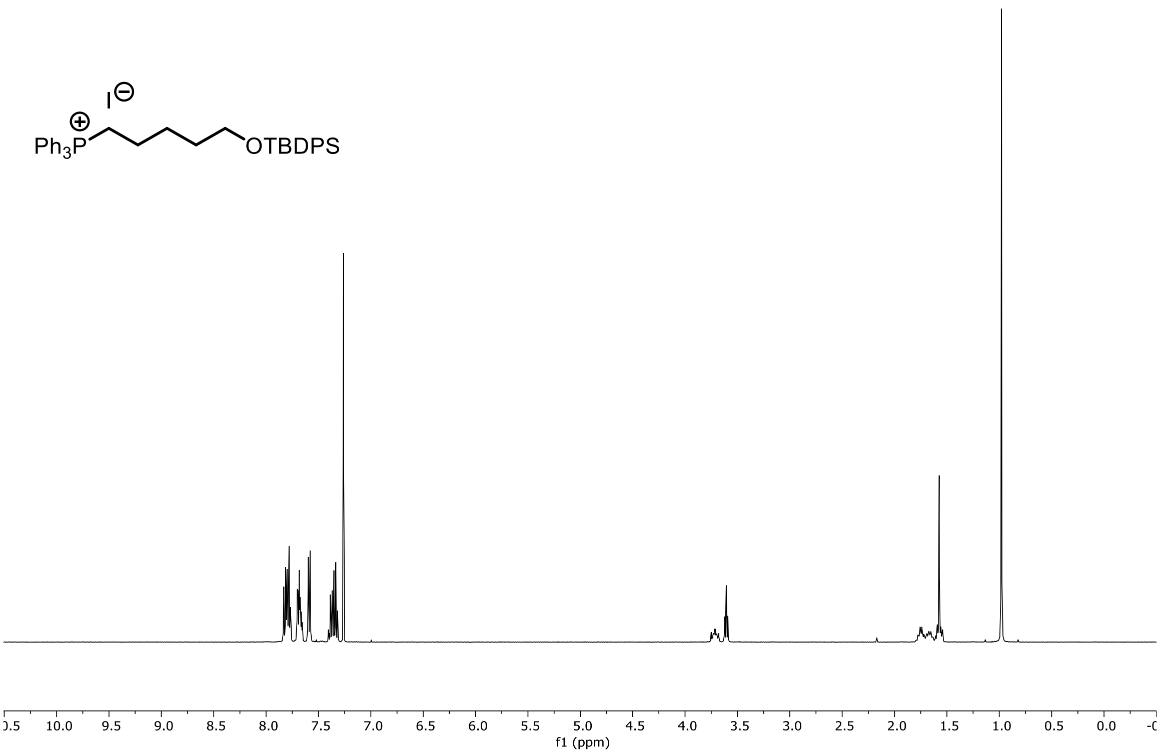

^{13}C NMR (101 MHz, $CDCl_3$) of **SI-5**

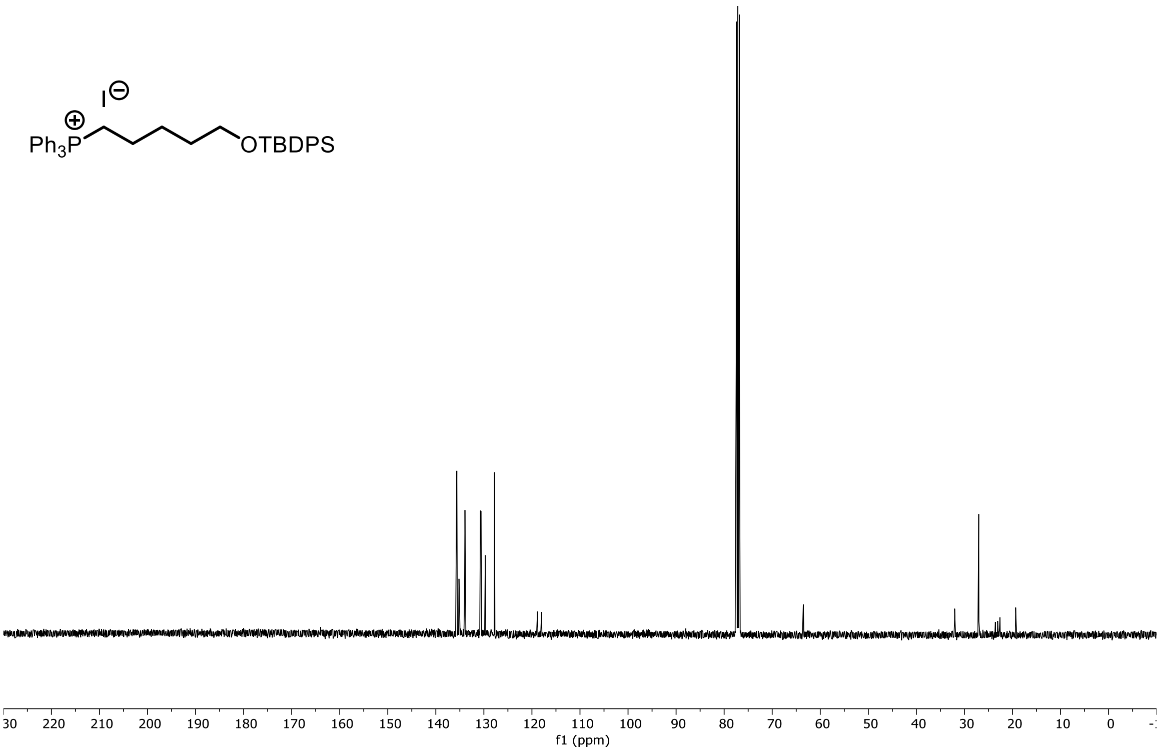

^{31}P NMR (162 MHz, $CDCl_3$) of **SI-5**

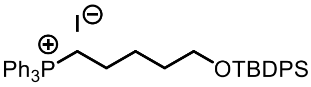

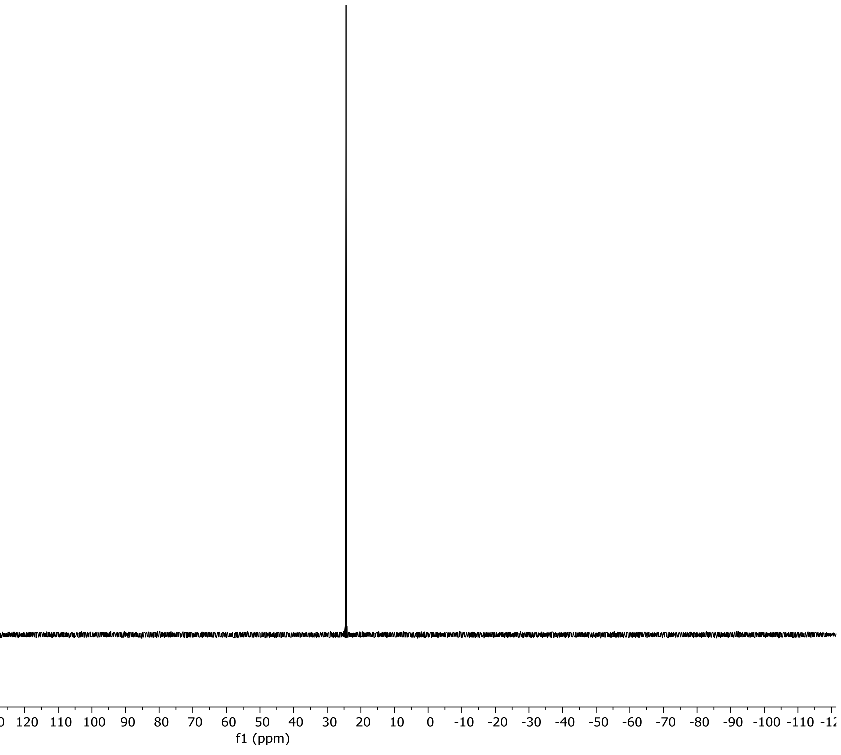

^{1}H NMR (400 MHz, $CDCl_3$) of (*E*)-**SI-6**

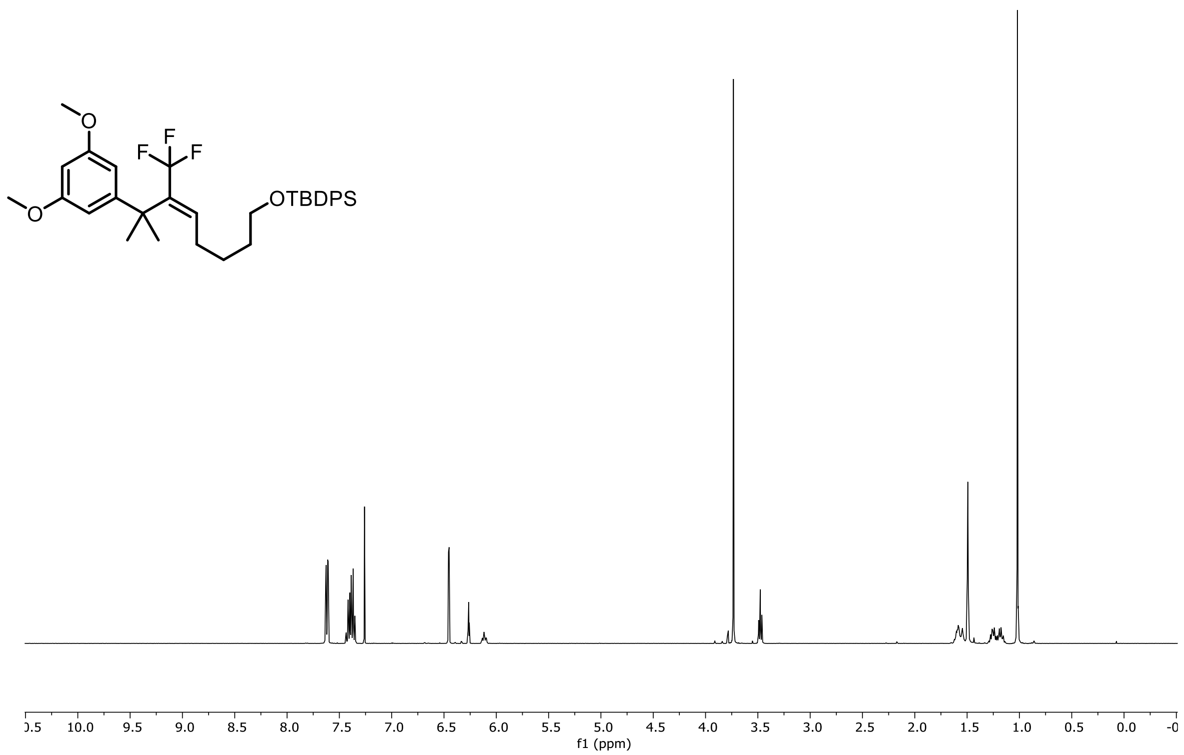

^{13}C NMR (101 MHz, $CDCl_3$) of (*E*)-**SI-6**

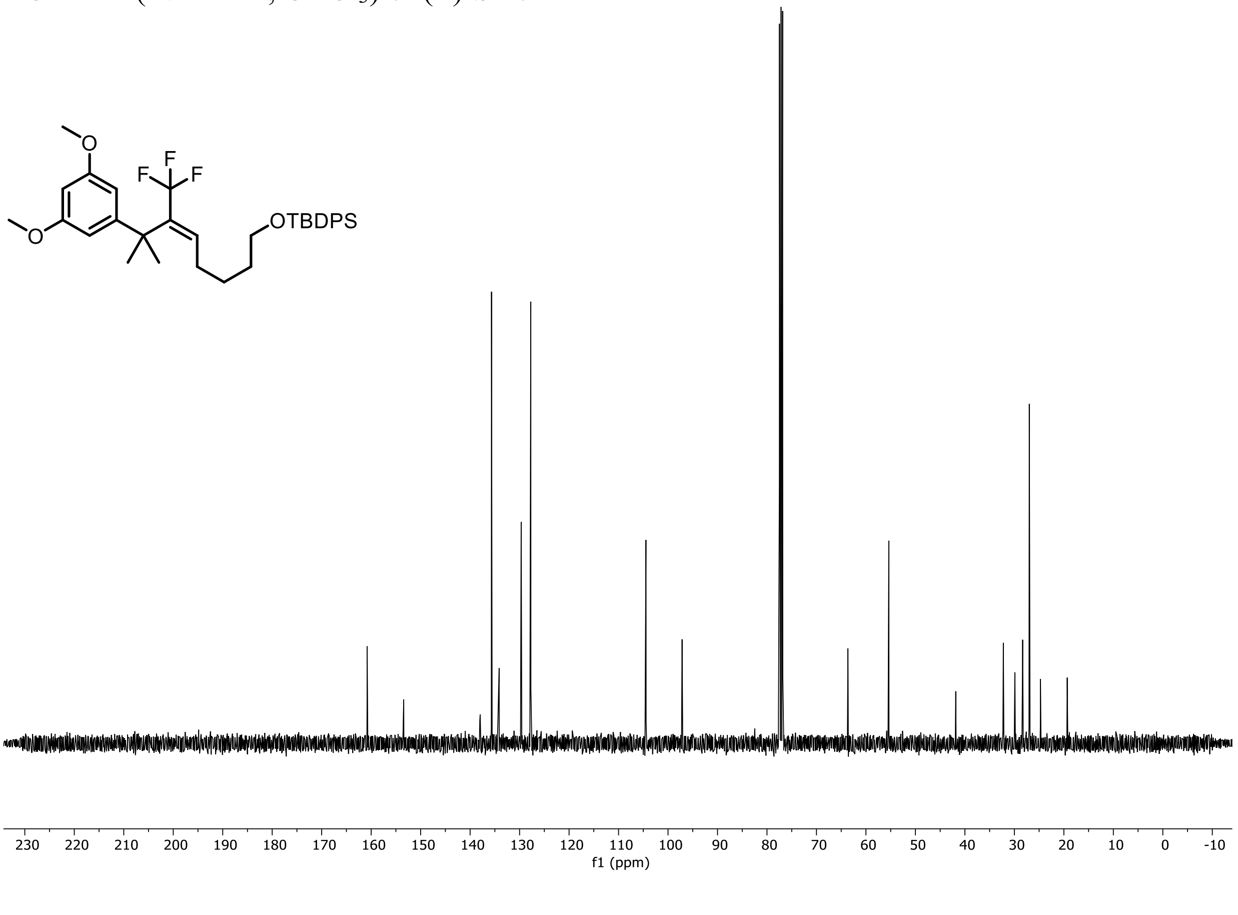

^{19}F NMR (377 MHz, $CDCl_3$) of *(E)*-**SI-6**

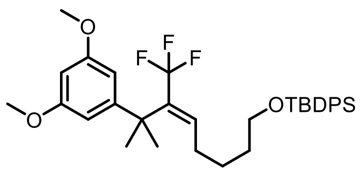

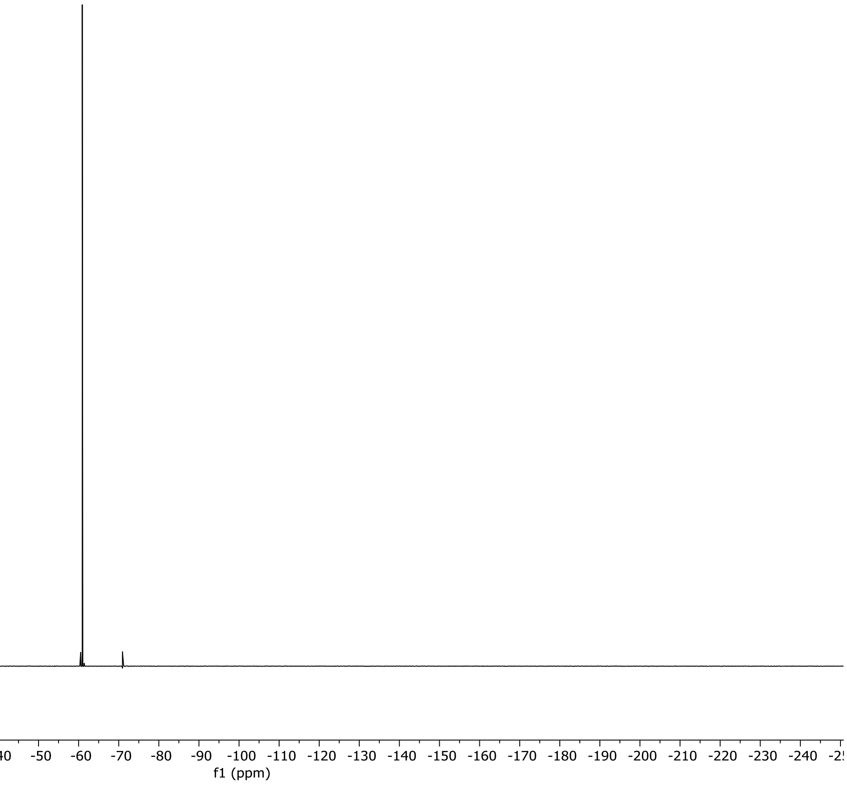

^{1}H NMR (400 MHz, $CDCl_3$) of (*Z*)-**SI-6**

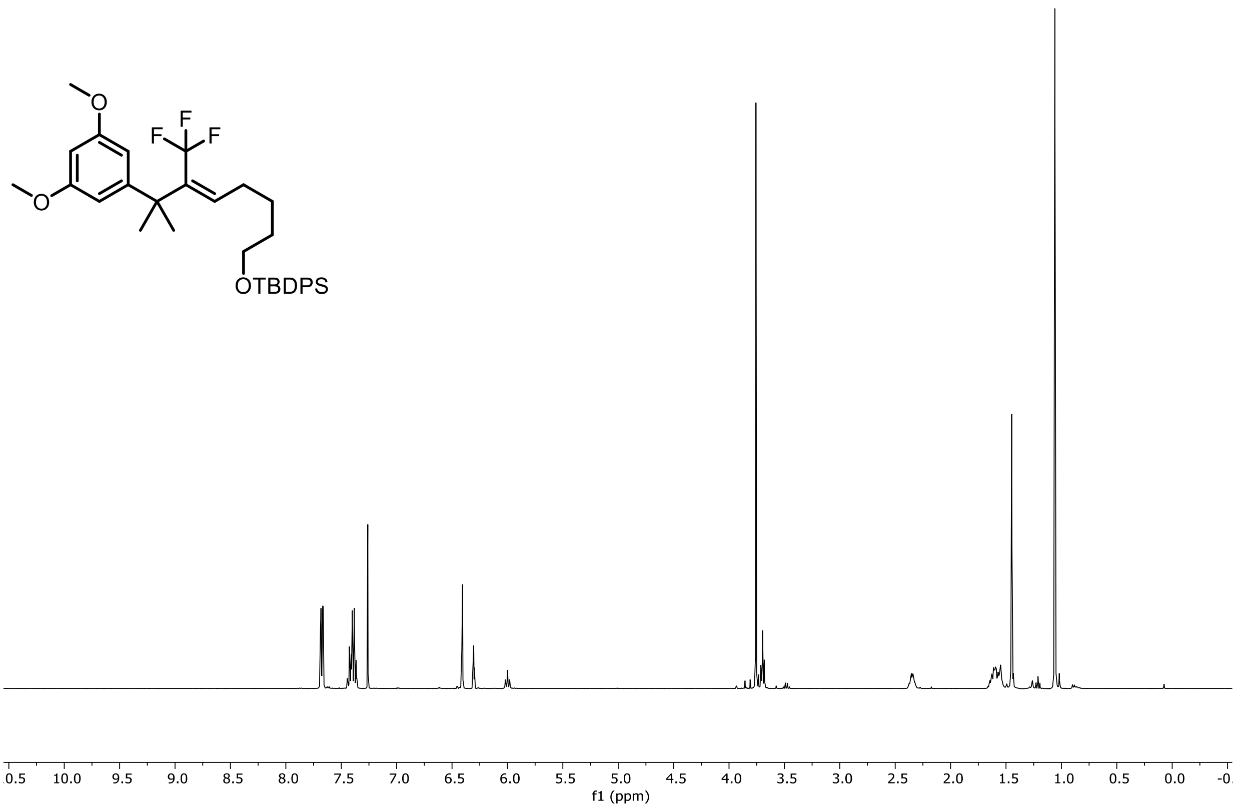

^{13}C NMR (101 MHz, $CDCl_3$) of (Z)-**SI-6**

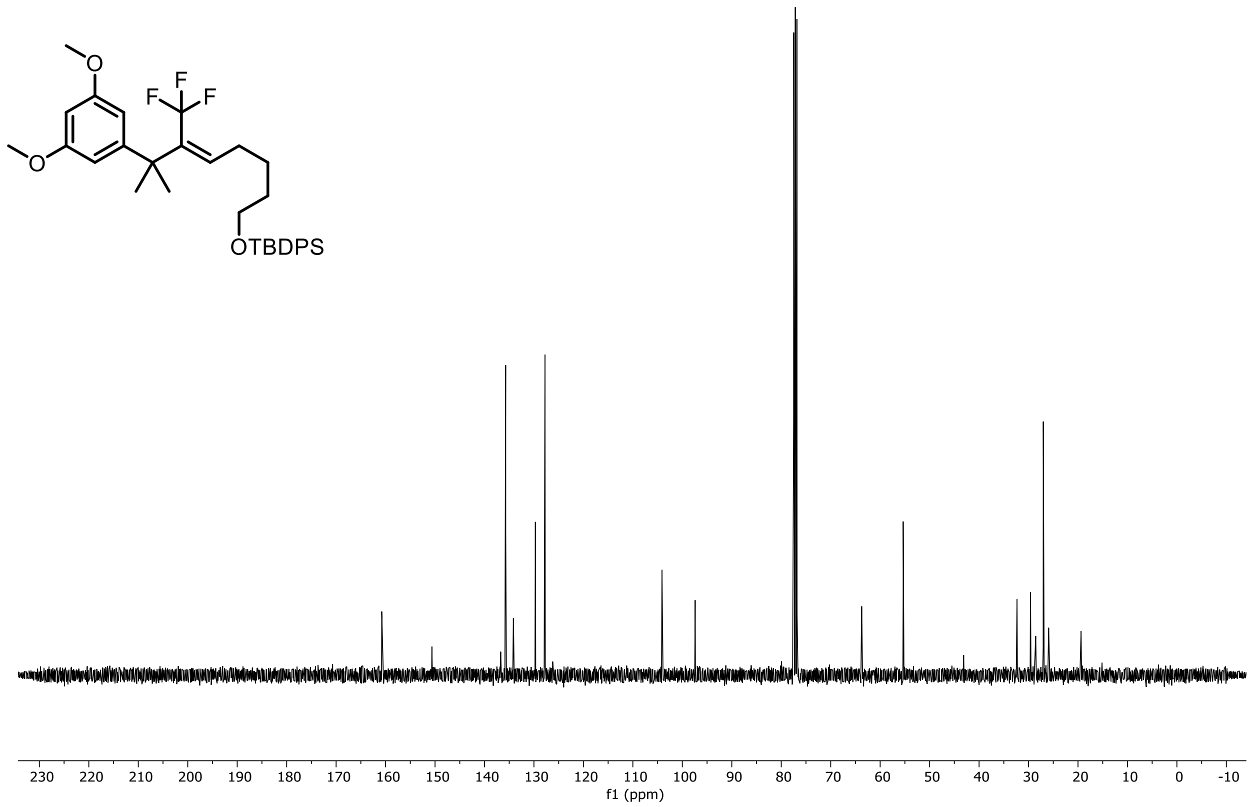

^{19}F NMR (377 MHz, $CDCl_3$) of (*Z*)-**SI-6**

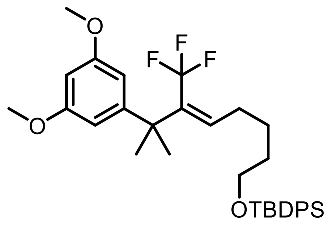

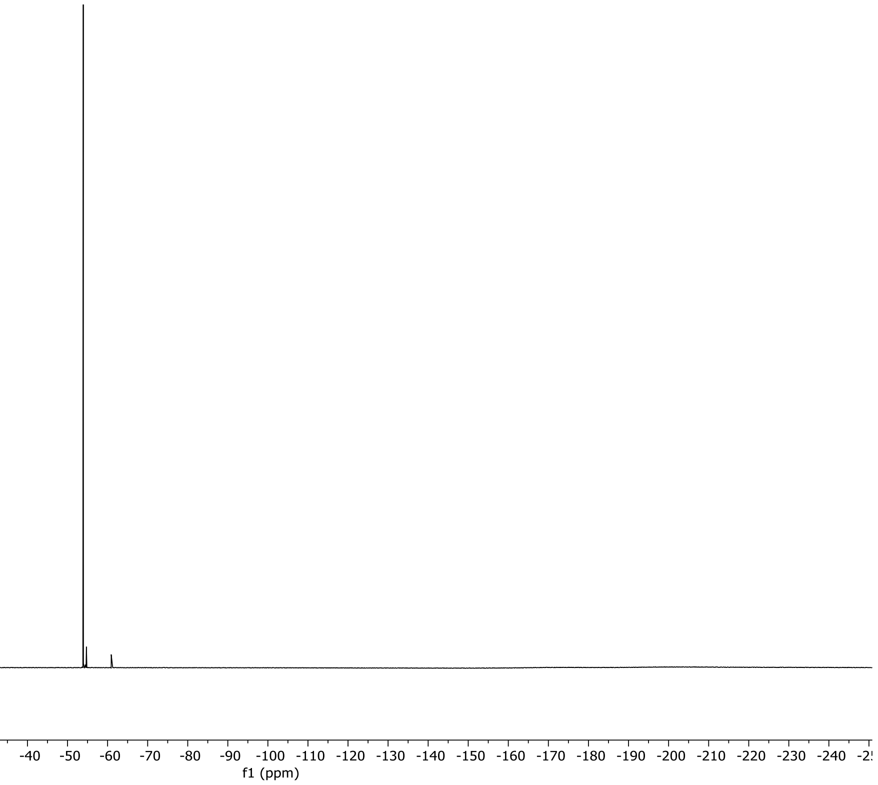

^{1}H NMR (400 MHz, $CDCl_3$) of **SI-7**

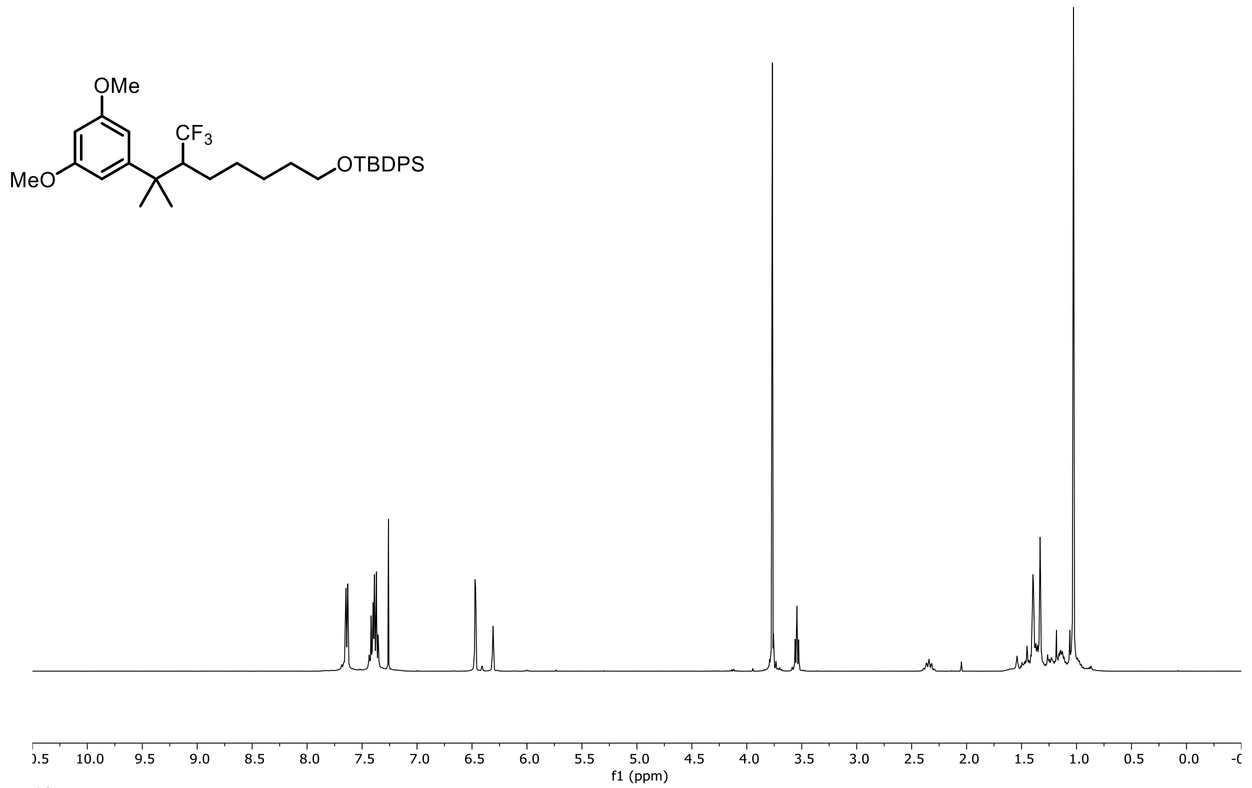

^{13}C NMR (101 MHz, $CDCl_3$) of **SI-7**

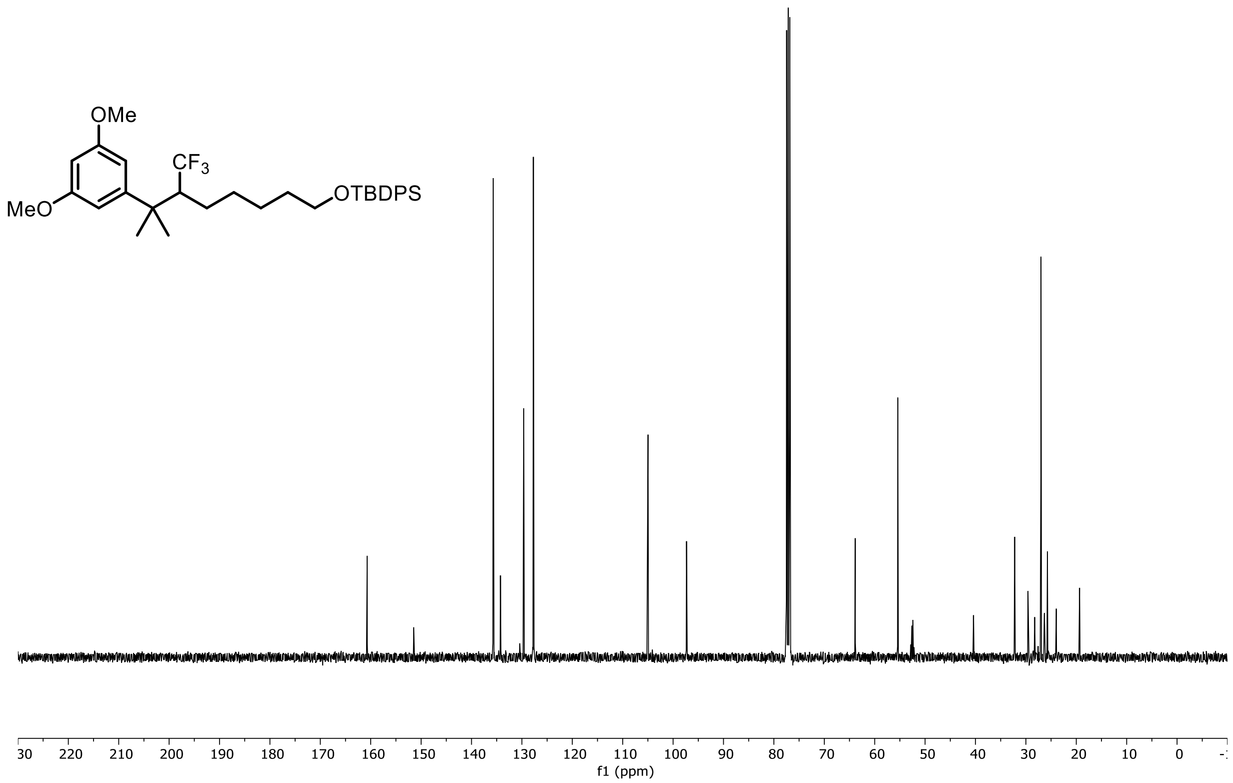

^{19}F NMR (377 MHz, $CDCl_3$) of **SI-7**

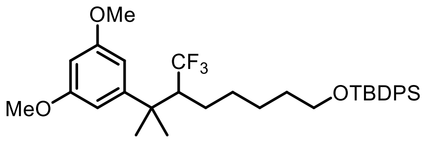

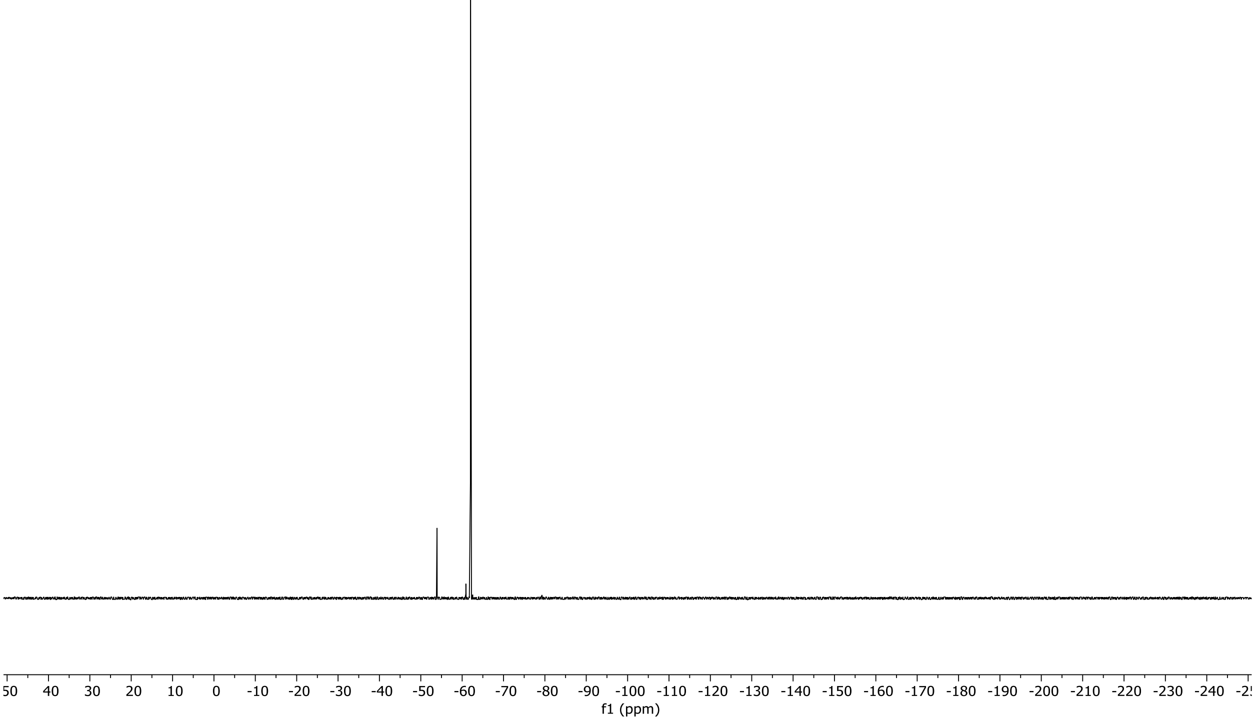

^{1}H NMR (400 MHz, $CDCl_3$) of (A)-**SI-8** & (B)-**SI-8**

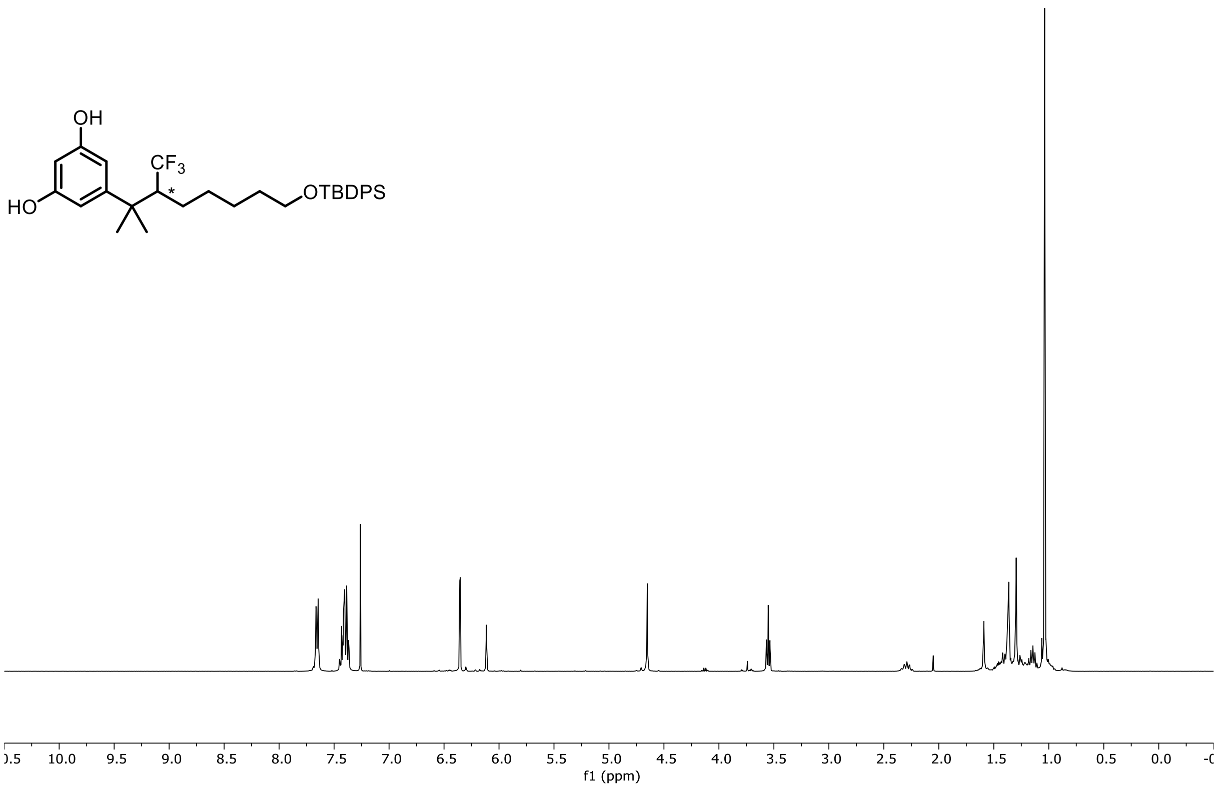

^{13}C NMR (101 MHz, $CDCl_3$) of (A)-**SI-8** & (B)-**SI-8**

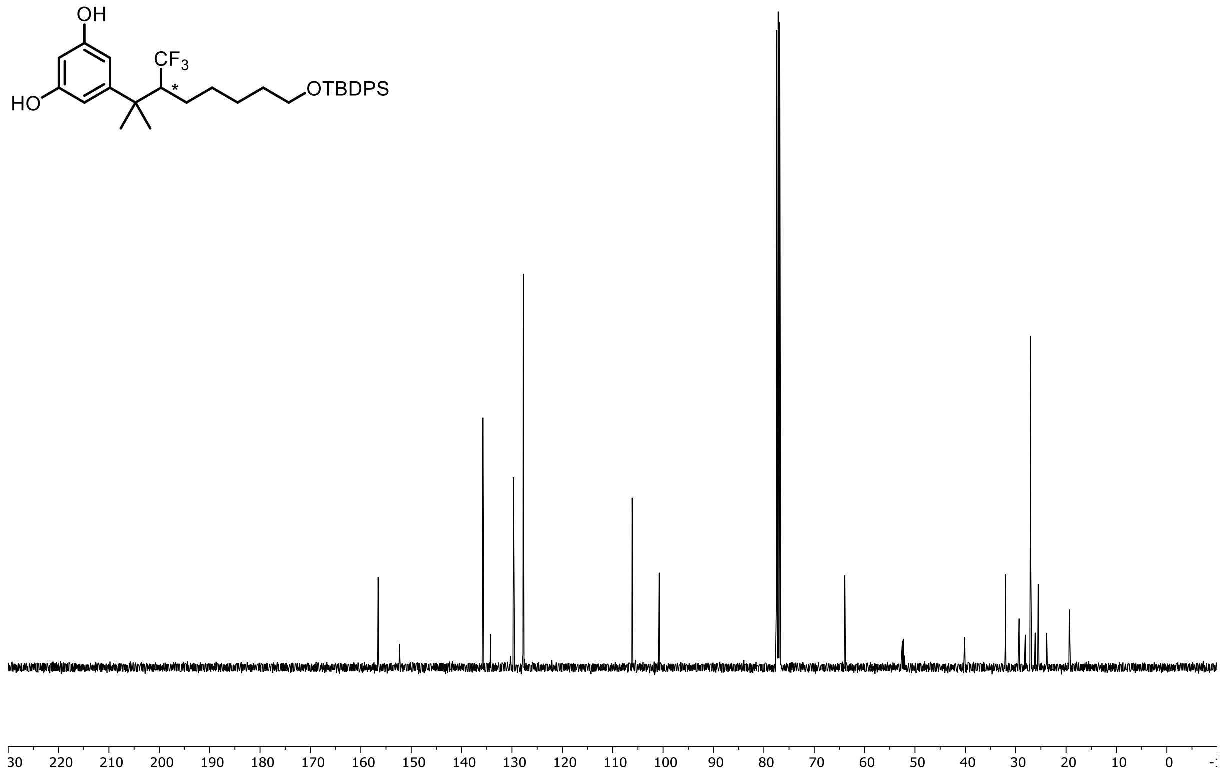

^{19}F NMR (377 MHz, $CDCl_3$) of (A)-**SI-8** & (B)-**SI-8**

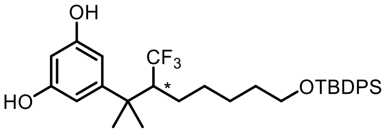

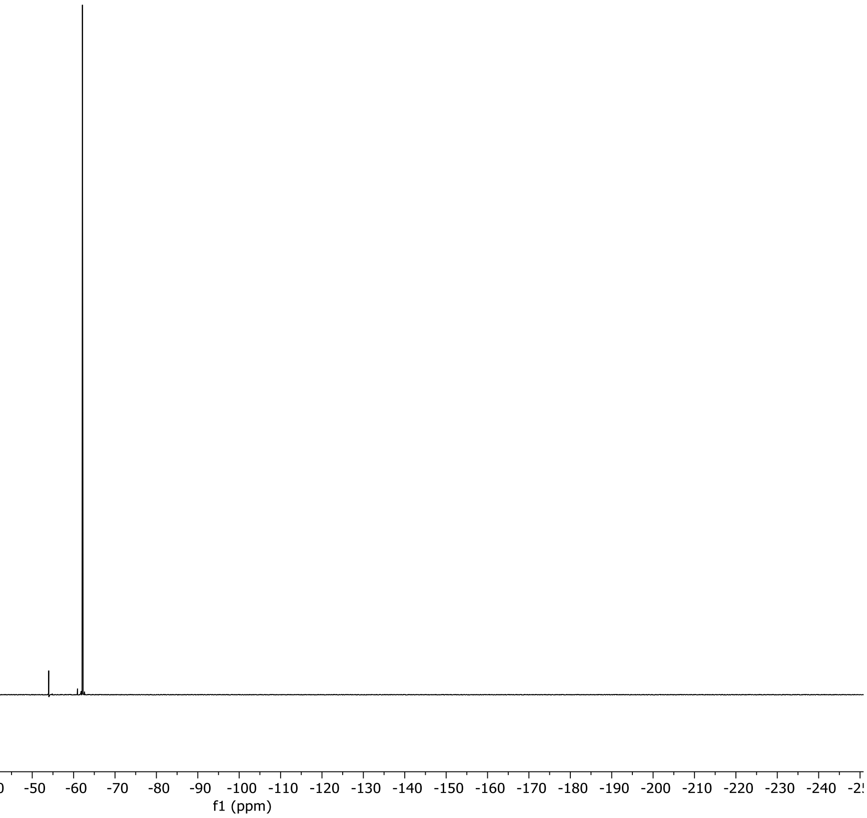

^{1}H NMR (400 MHz, $CDCl_3$) of (B)/(*S*)-**SI-9**

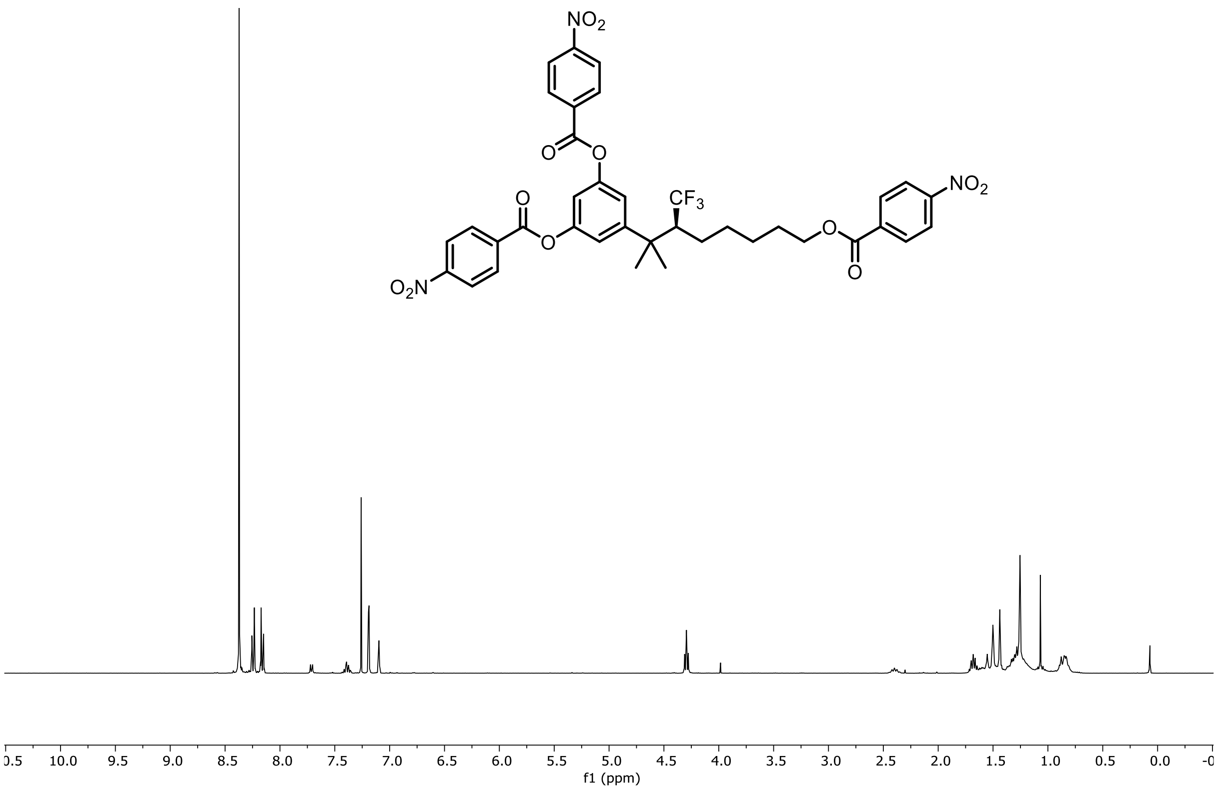

^{13}C NMR (101 MHz, $CDCl_3$) of (B)/(*S*)-**SI-9**

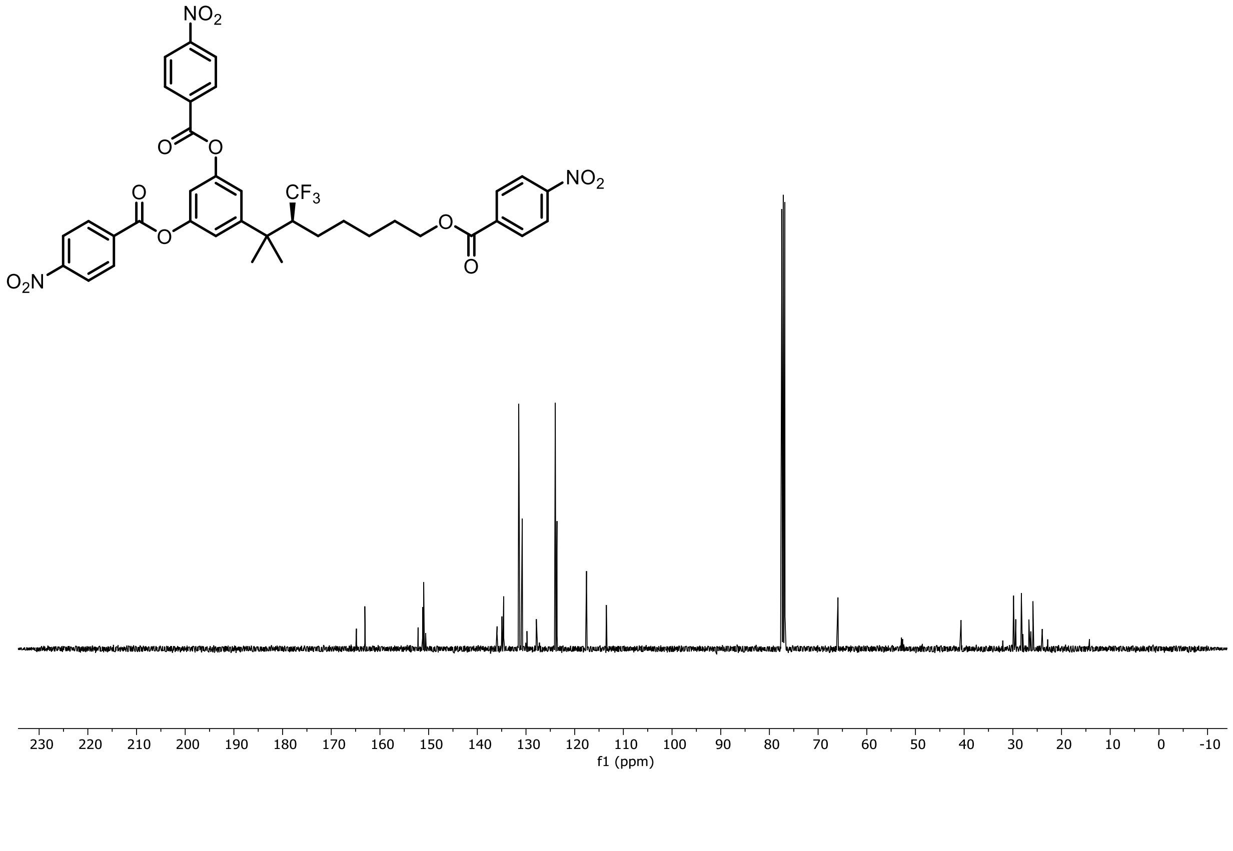

^{19}F NMR (377 MHz, $CDCl_3$) of (B)/(*S*)-**SI-9**

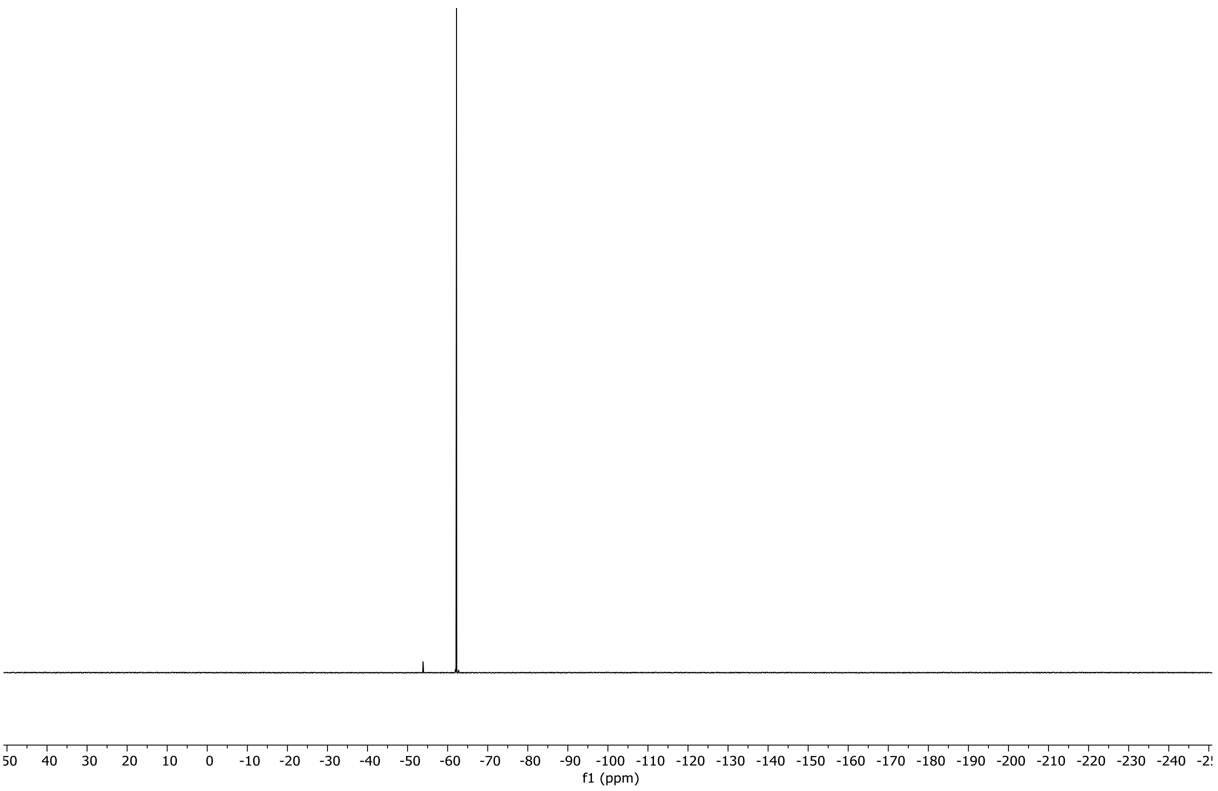

^{1}H NMR (400 MHz, $CDCl_3$) of (A)-**SI-10** for comparison to (A)-**4** = (+)-(*R*)-**4**

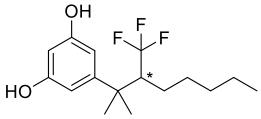

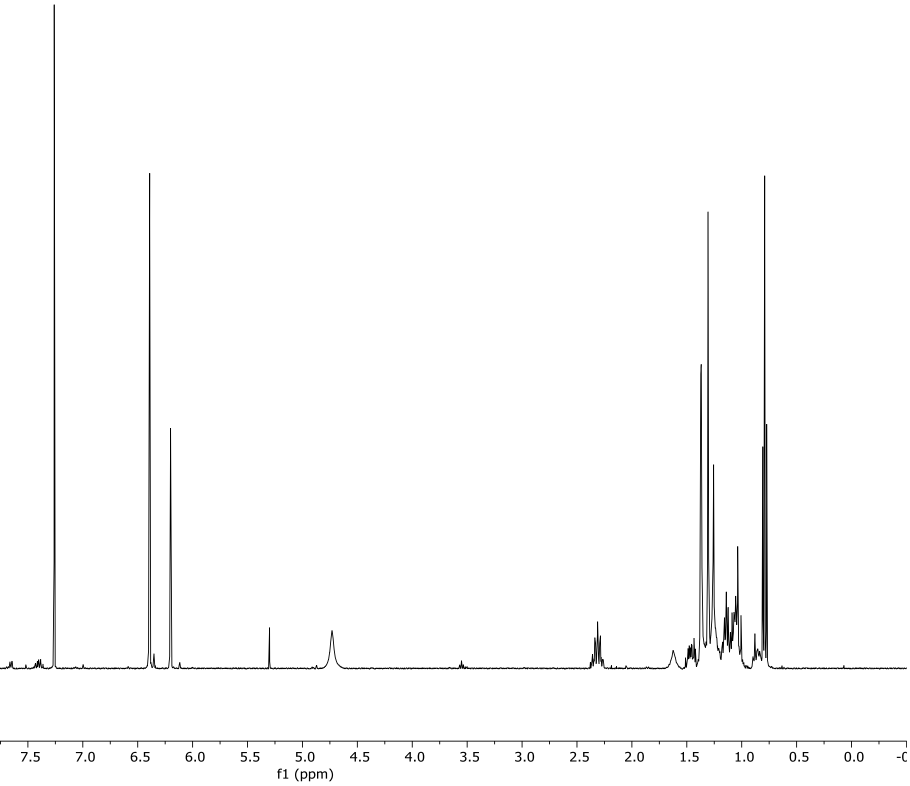

^{1}H NMR (500 MHz, $CDCl_3$) of (*R*)-**SI-13**

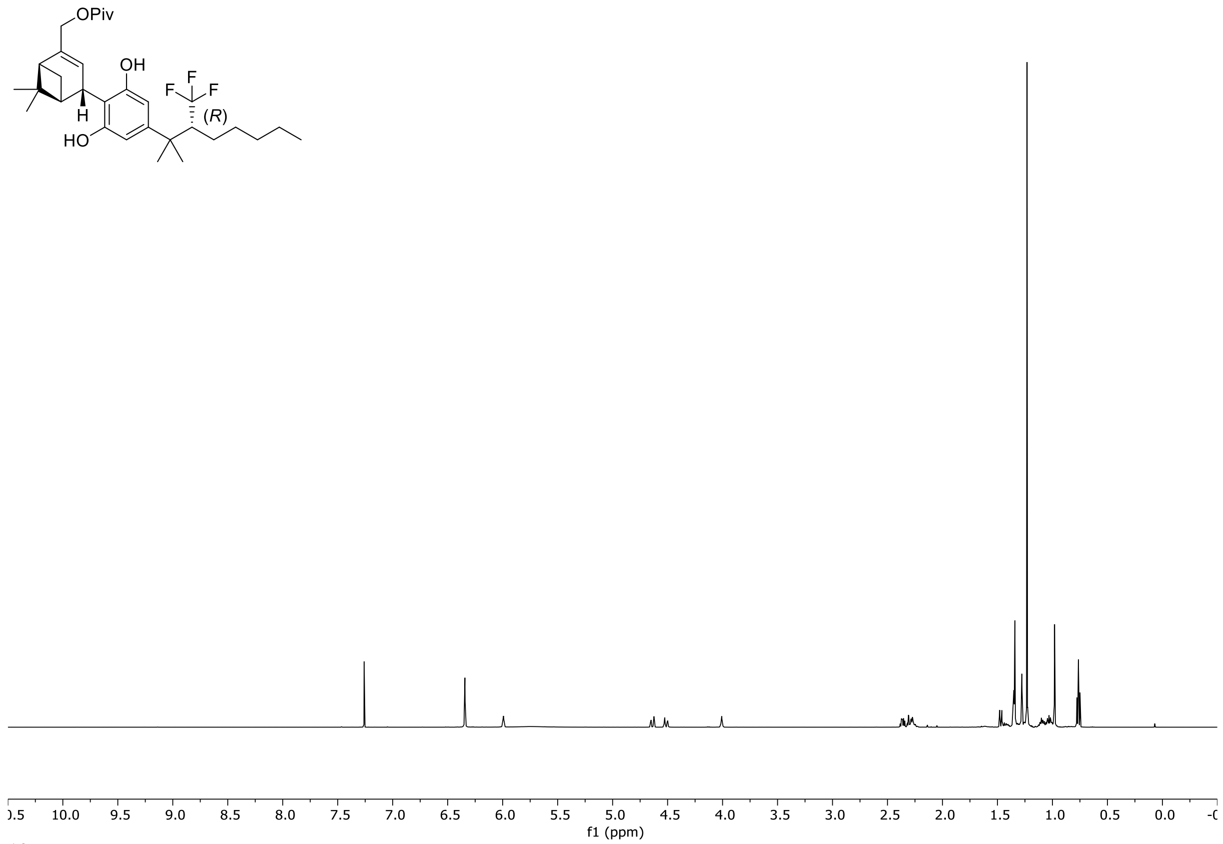

^{13}C NMR (126 MHz, $CDCl_3$) of (*R*)-**SI-13**

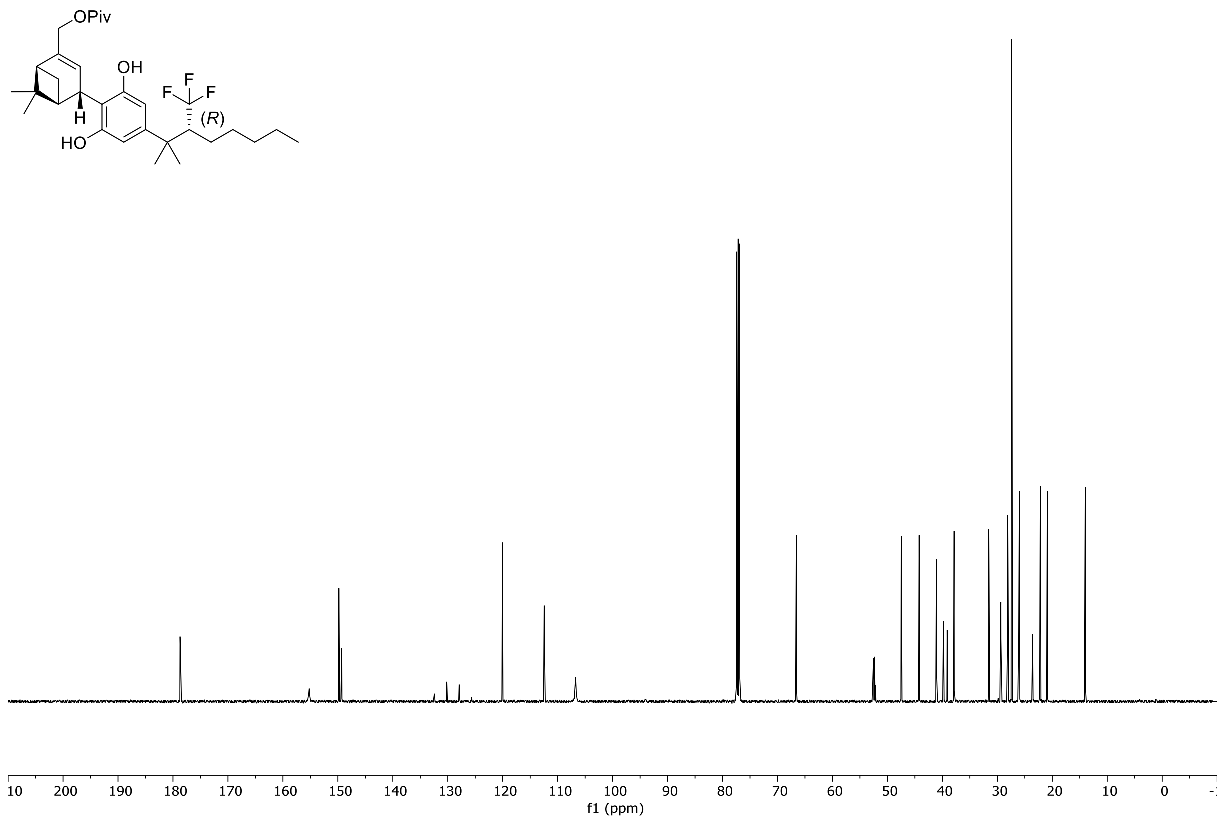

^{19}F NMR (471 MHz, $CDCl_3$) of (*R*)-**SI-13**

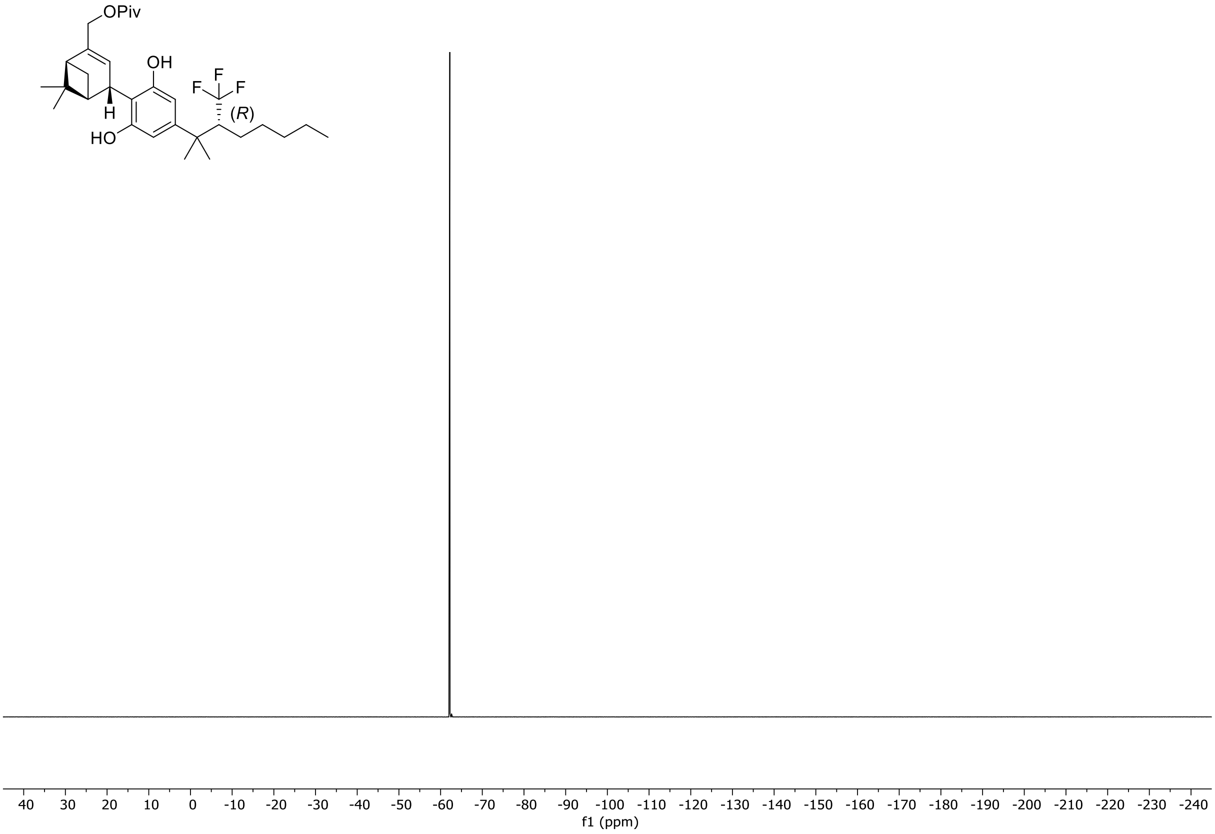

^{1}H NMR (500 MHz, $CDCl_3$) of (*S*)-**SI-13**

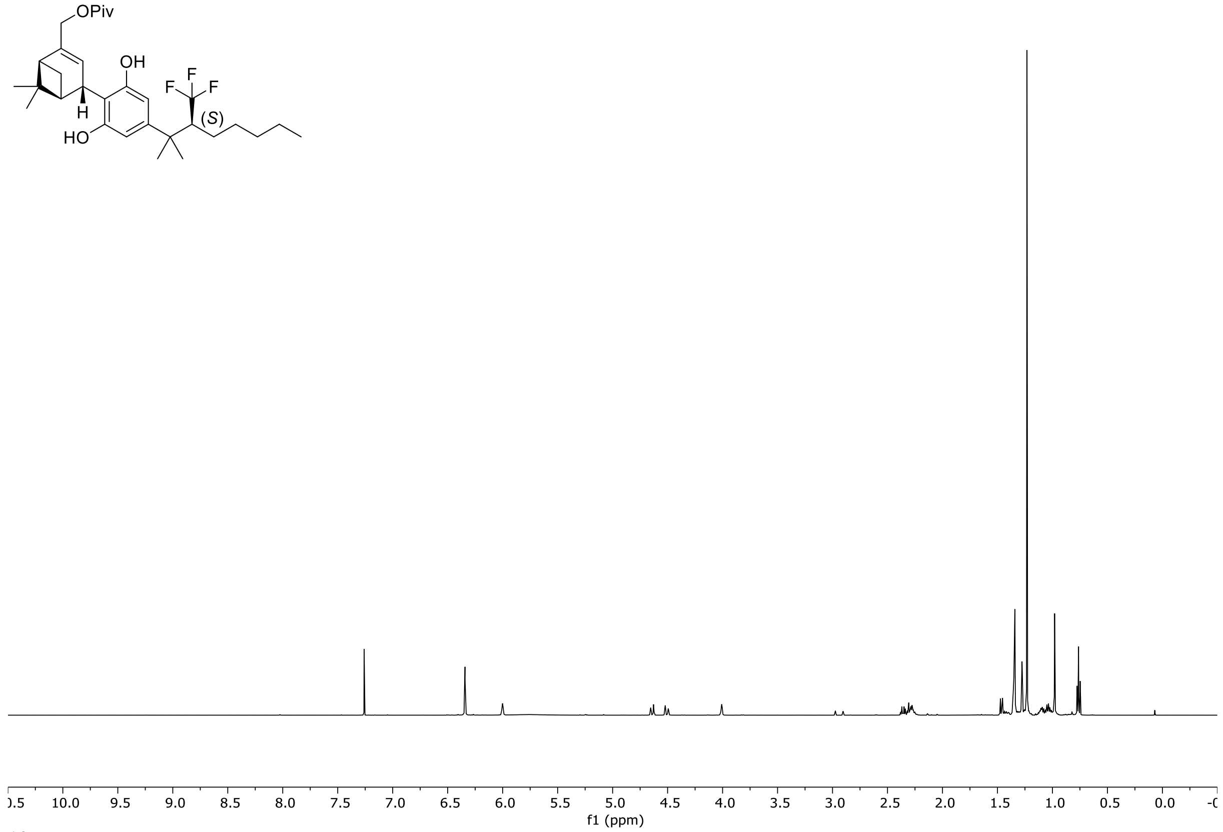

^{13}C NMR (126 MHz, $CDCl_3$) of (*S*)-**SI-13**

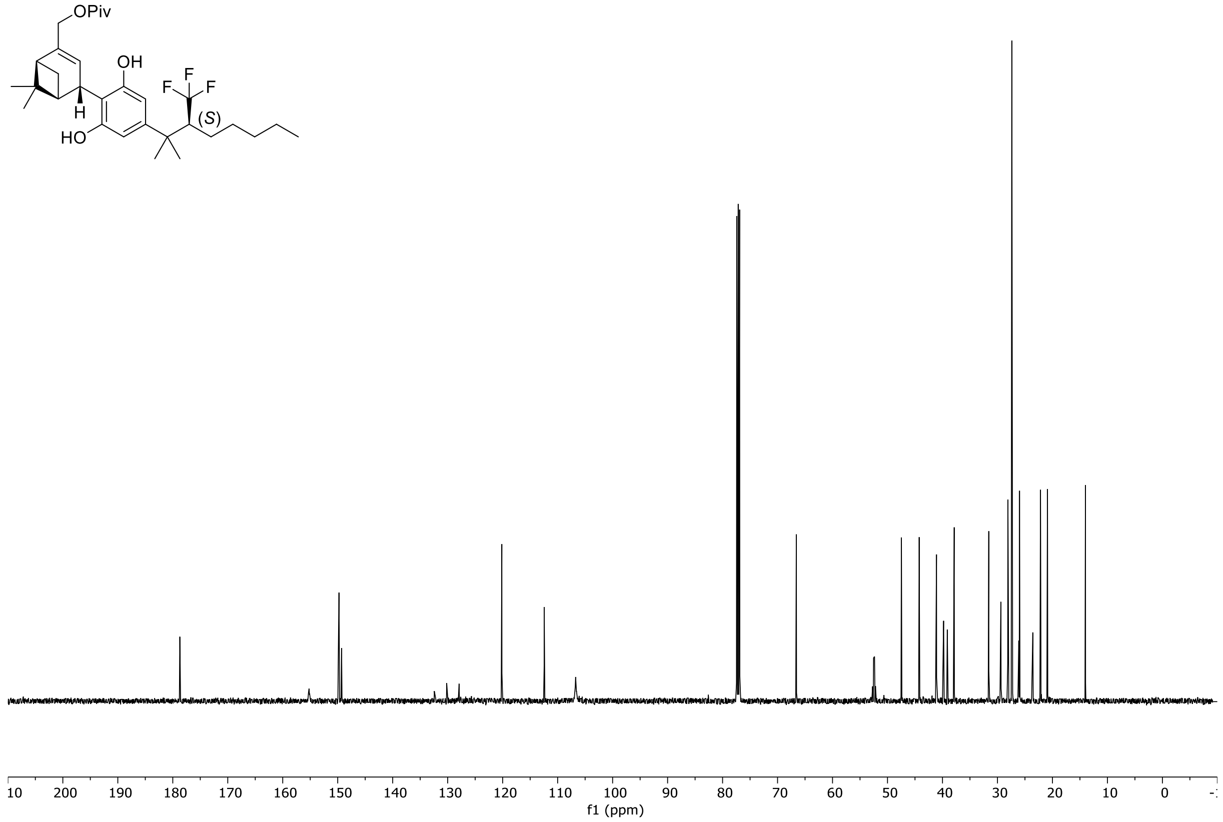

^{19}F NMR (471 MHz, $CDCl_3$) of (*S*)-**SI-13**

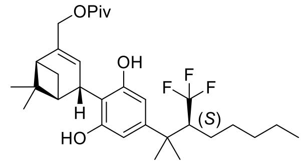

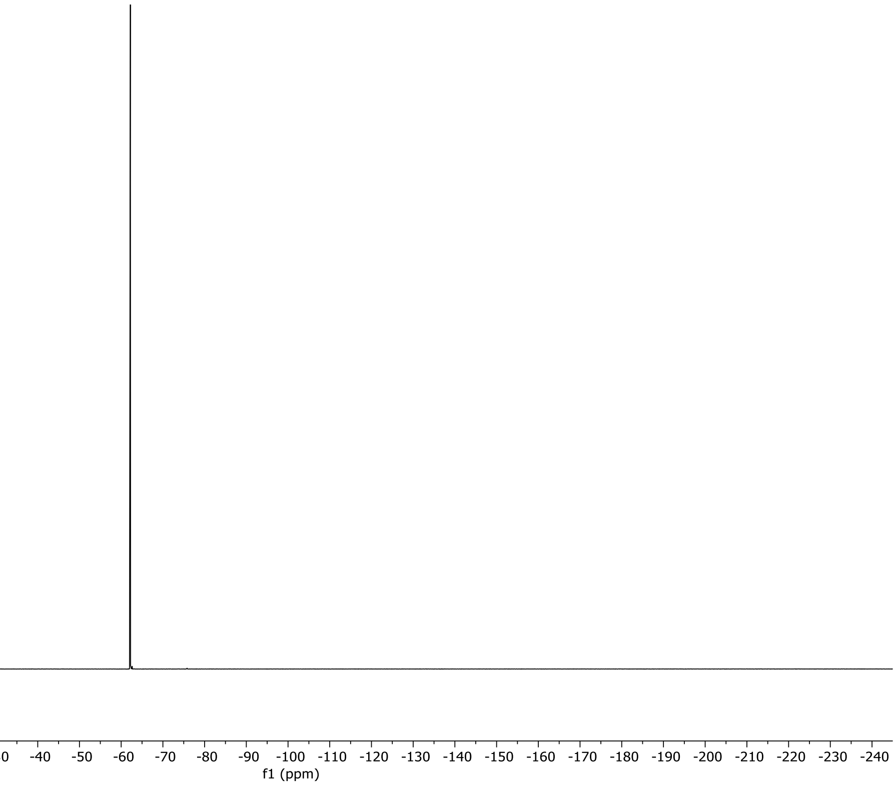

^{1}H NMR (500 MHz, $CDCl_3$) of (*R*)-**SI-14**

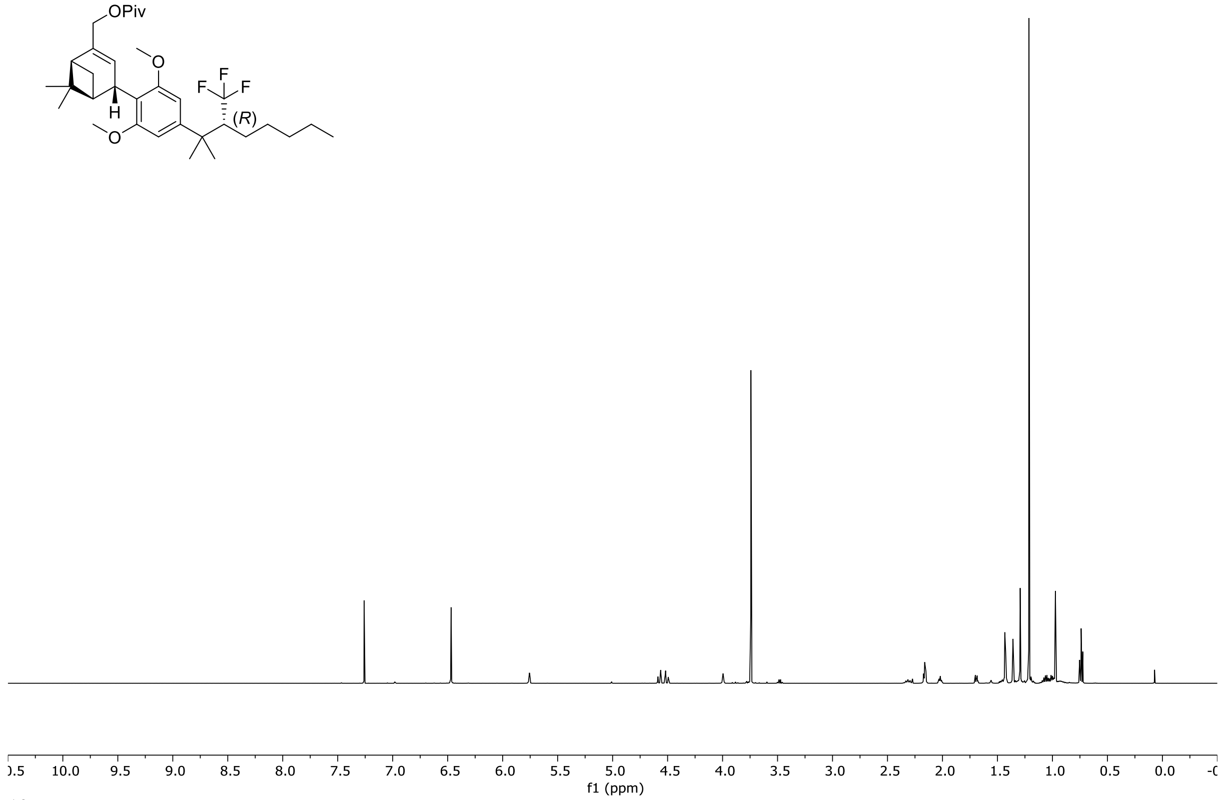

^{13}C NMR (126 MHz, $CDCl_3$) of (*R*)-**SI-14**

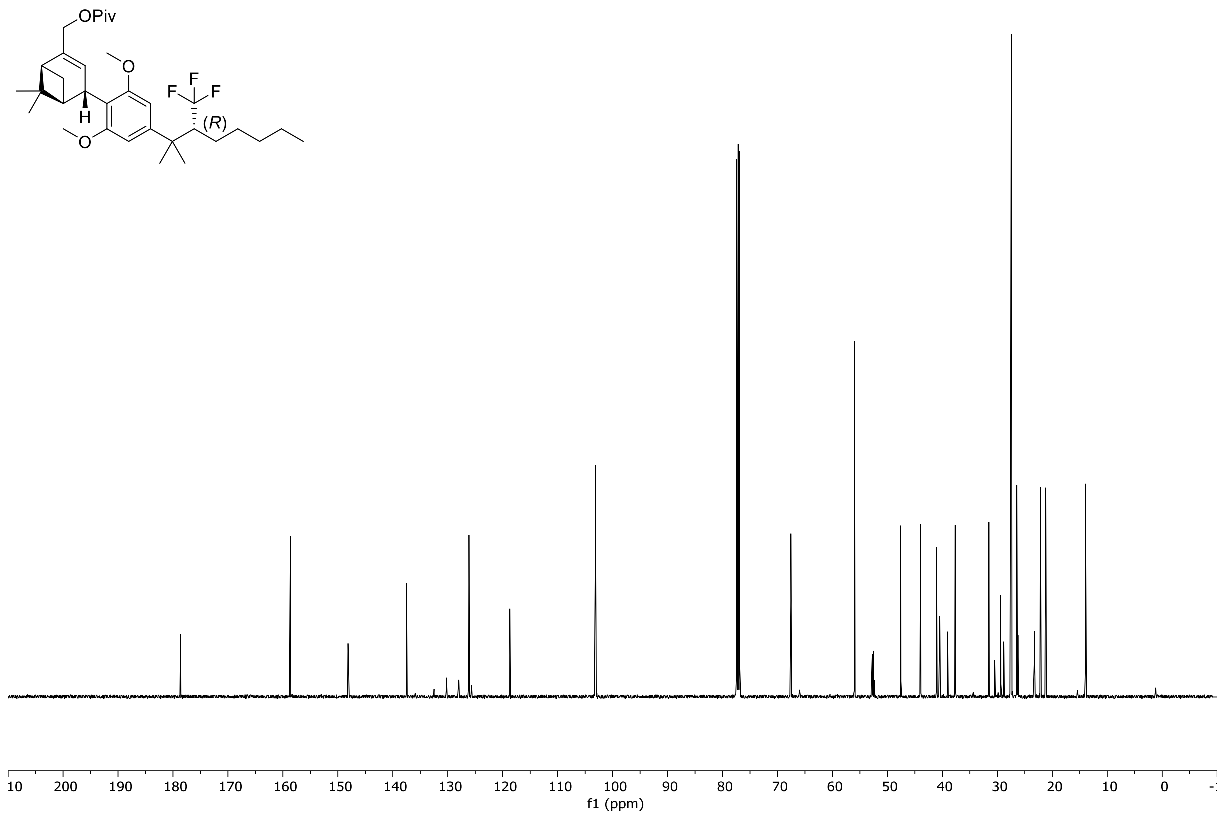

^{19}F NMR (471 MHz, $CDCl_3$) of (*R*)-**SI-14**

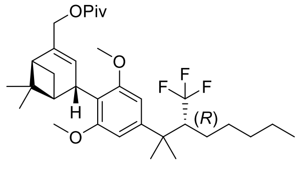

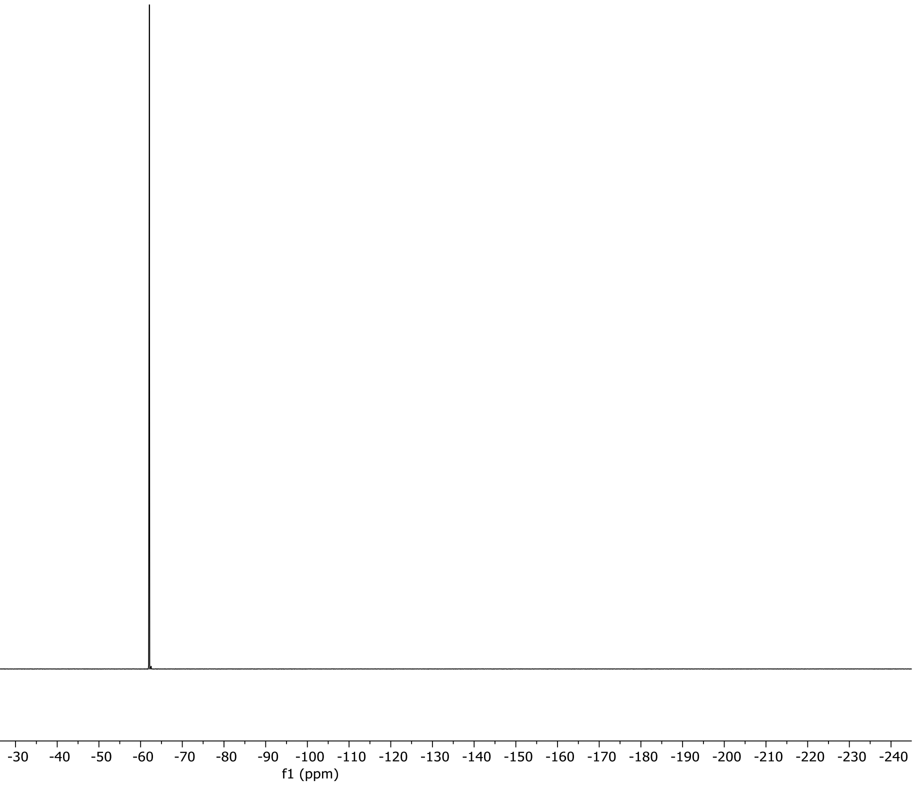

^{1}H NMR (500 MHz, $CDCl_3$) of (*S*)-**SI-14**

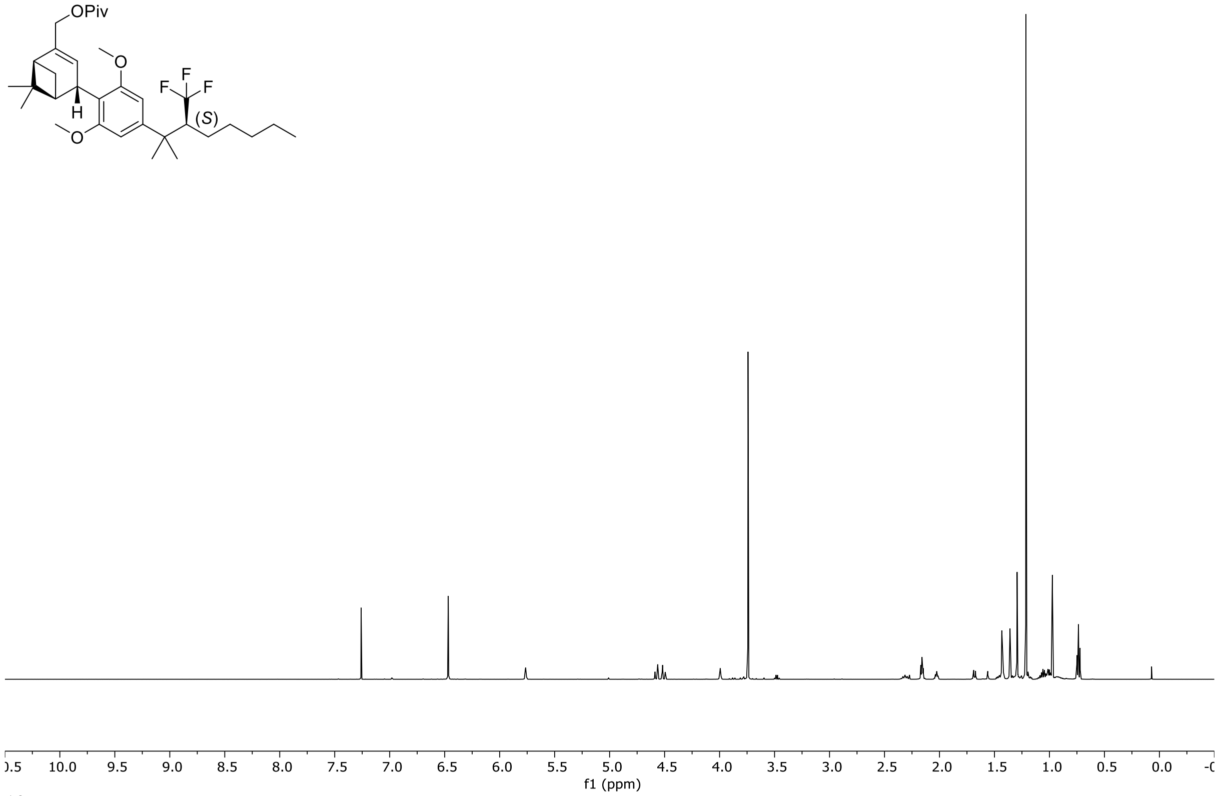

^{13}C NMR (126 MHz, $CDCl_3$) of (*S*)-**SI-14**

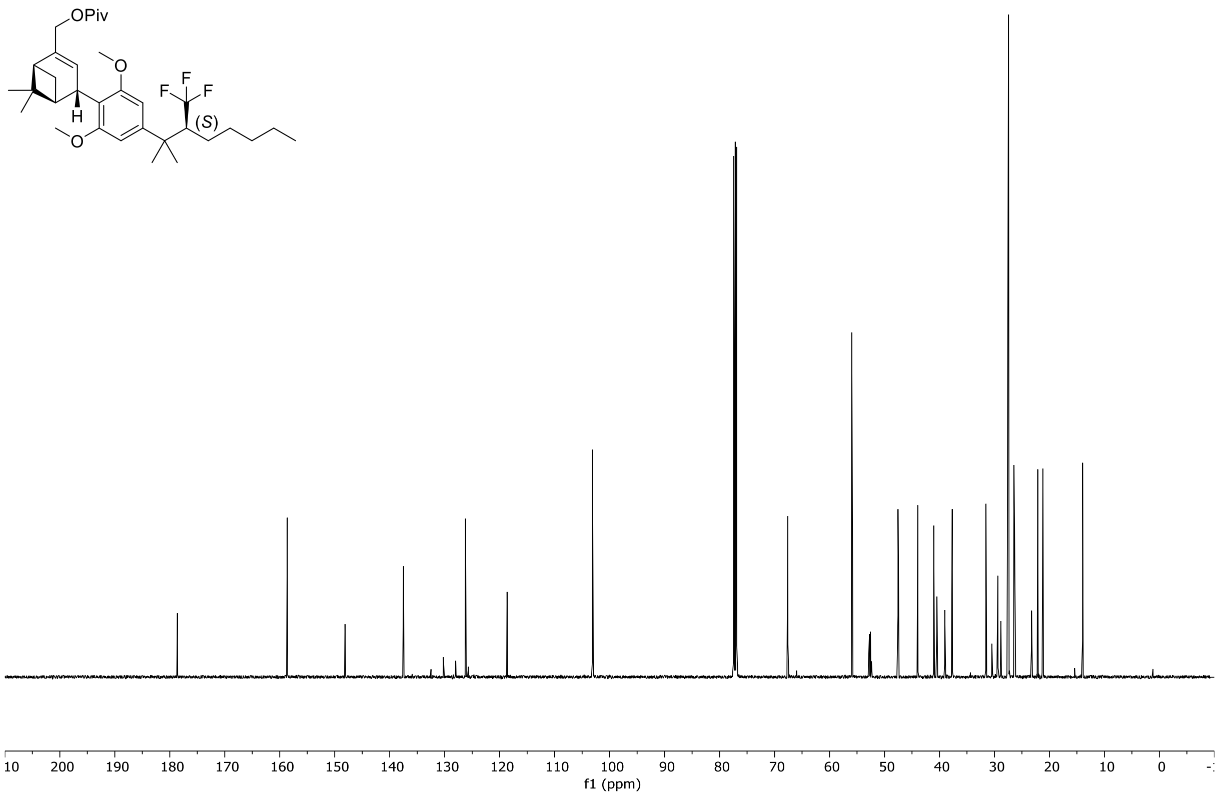

^{19}F NMR (471 MHz, $CDCl_3$) of (*S*)-**SI-14**

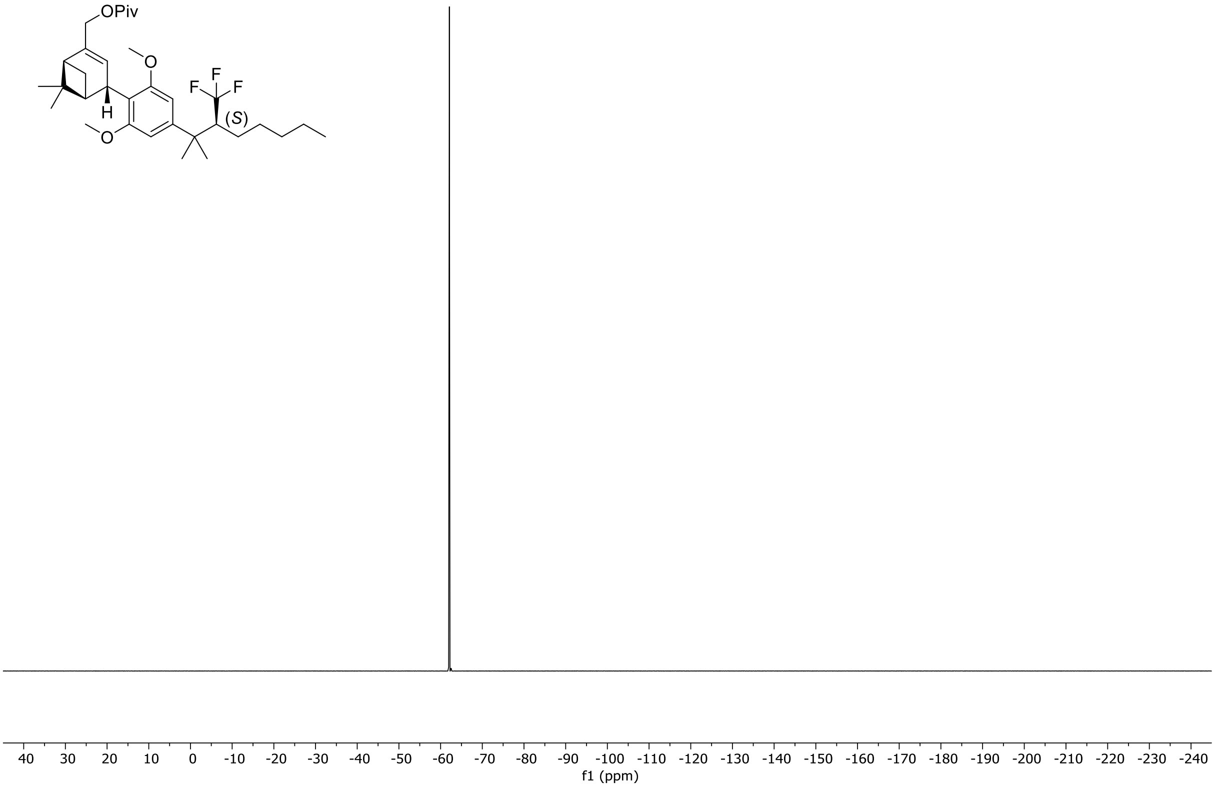

^{1}H NMR (500 MHz, $CDCl_3$) of (*R*)-**1**

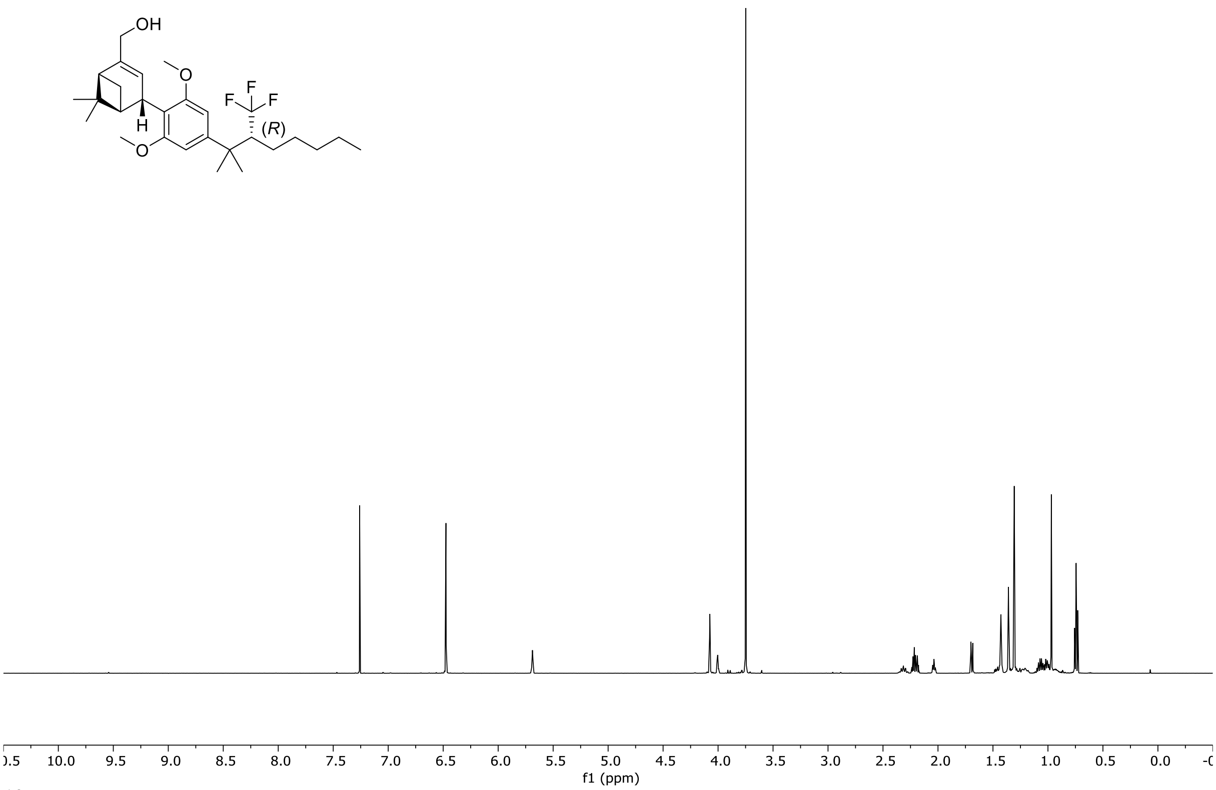

^{13}C NMR (126 MHz, $CDCl_3$) of (*R*)-**1**

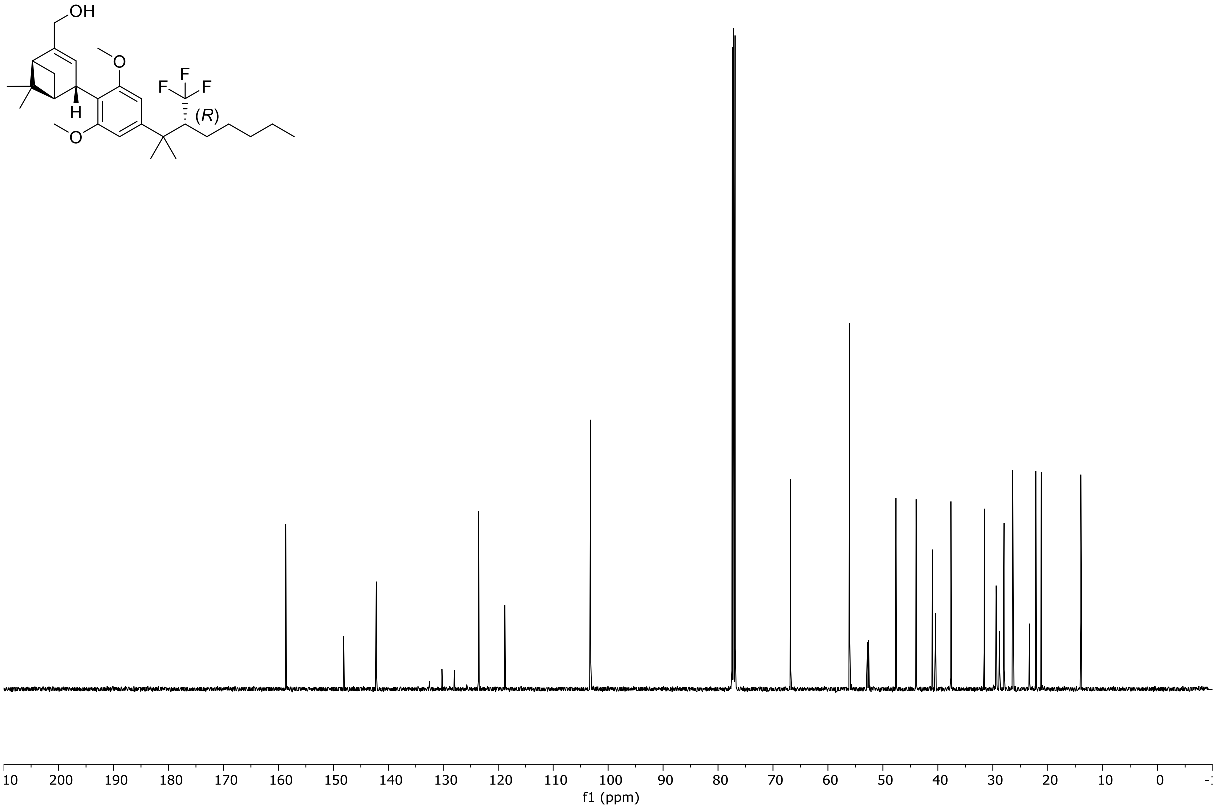

^{19}F NMR (471 MHz, $CDCl_3$) of (*R*)-**1**

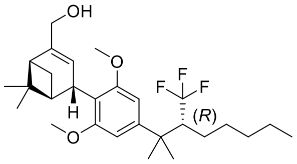

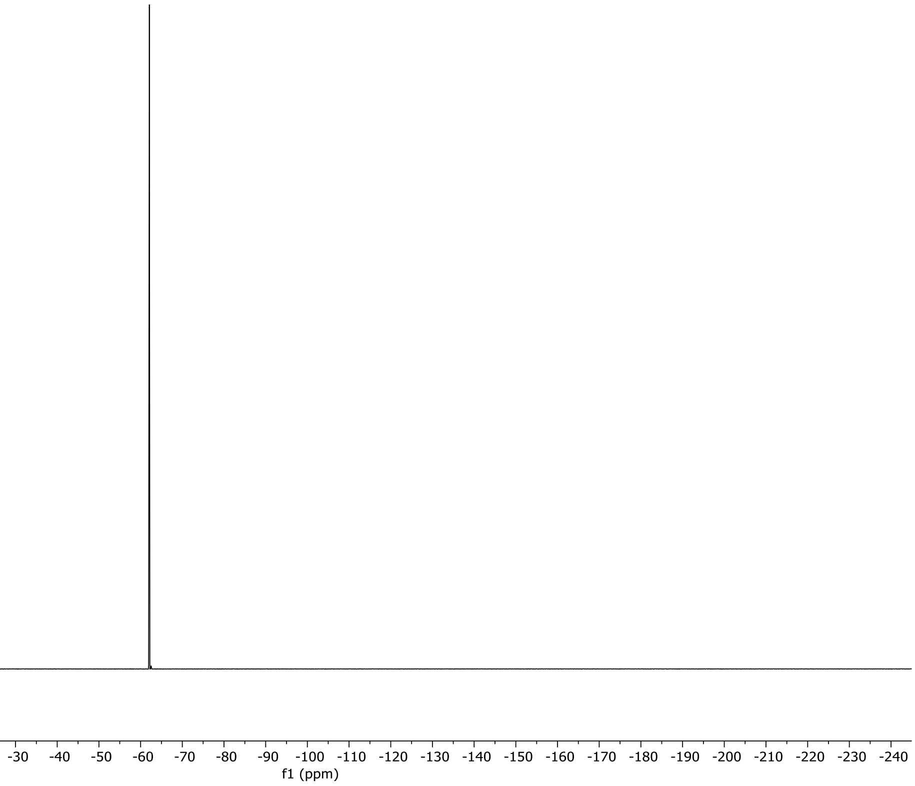

^{1}H NMR (400 MHz, $CDCl_3$) of (*S*)-**1**

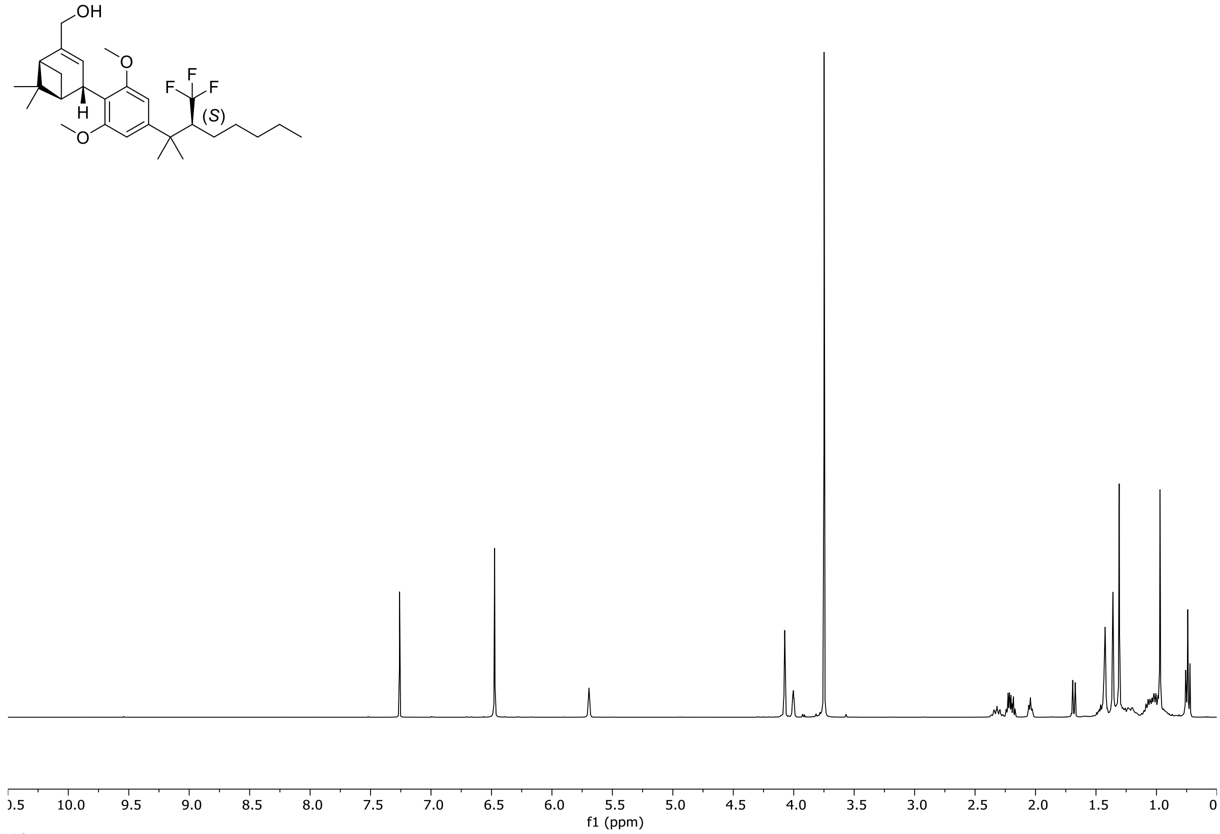

^{13}C NMR (101 MHz, $CDCl_3$) of (*S*)-**1**

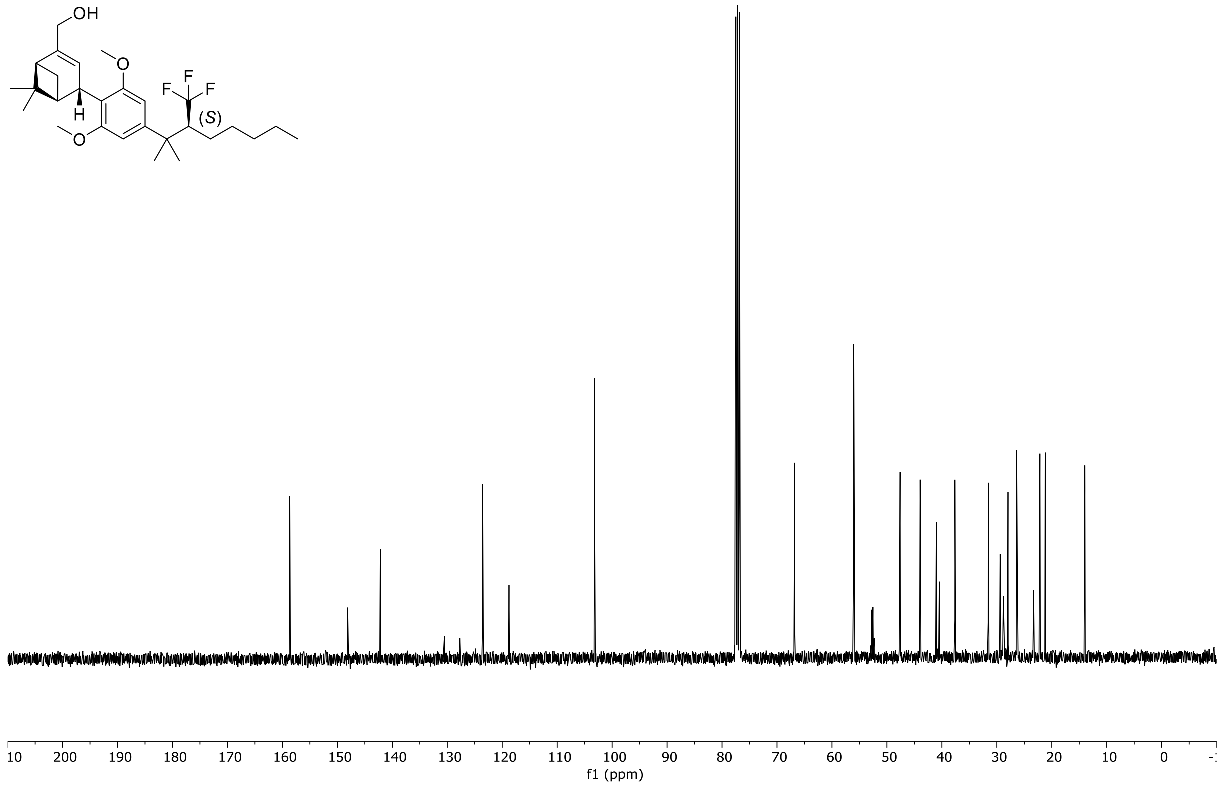

^{19}F NMR (376 MHz, $CDCl_3$) of (*S*)-**1**

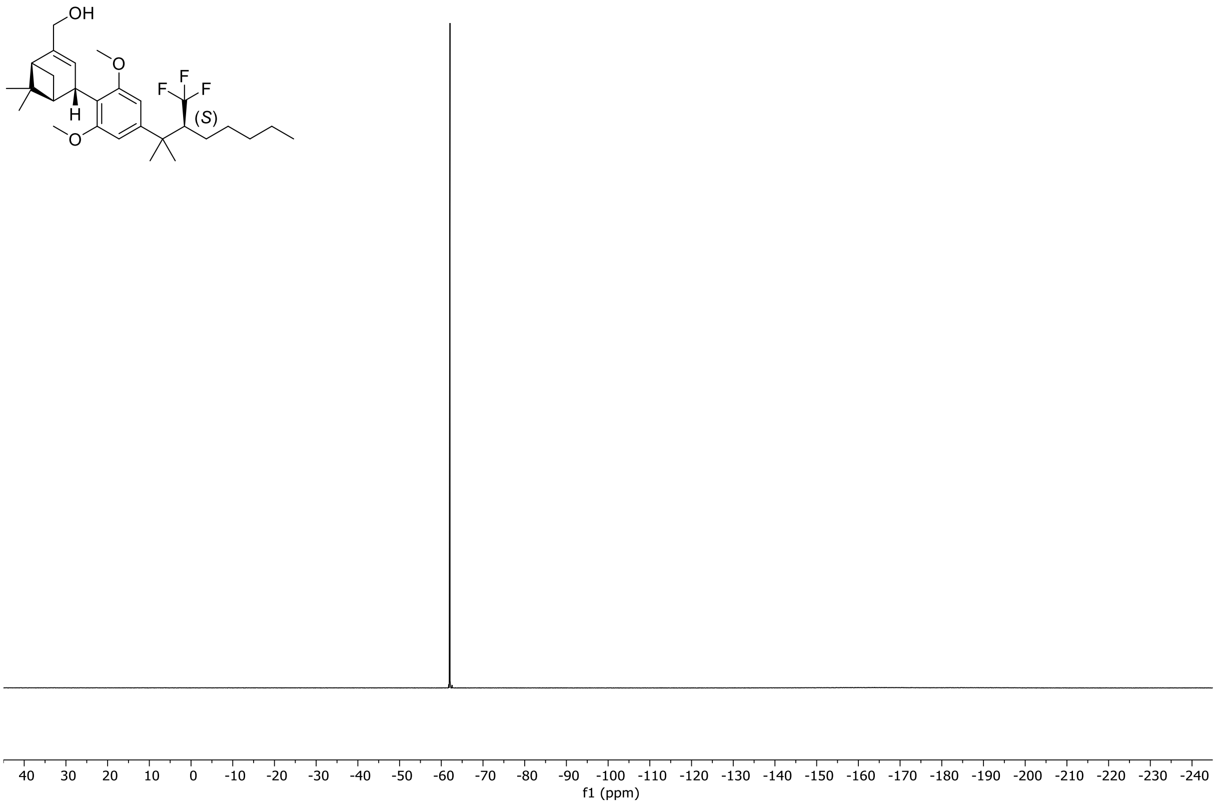

^{1}H NMR (400 MHz, $CDCl_3$) of **7**

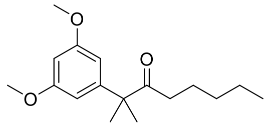

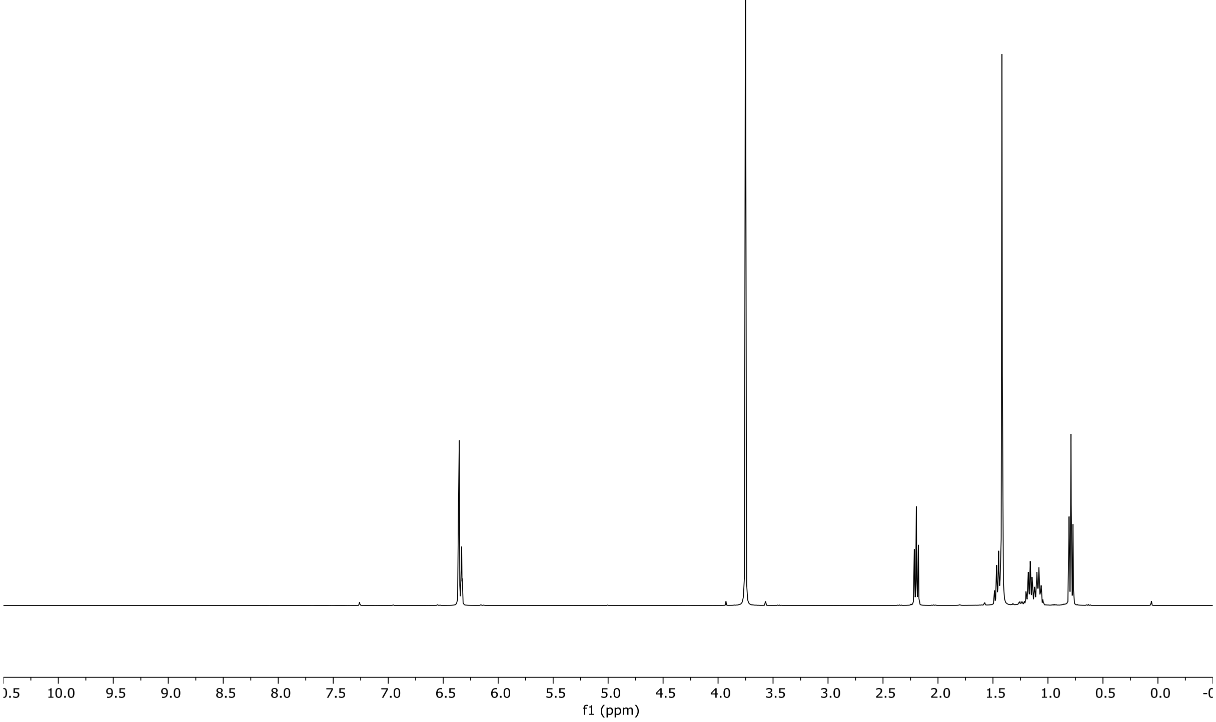

^{13}C NMR (101 MHz, $CDCl_3$) of **7**

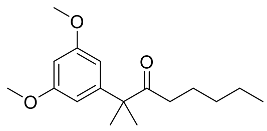

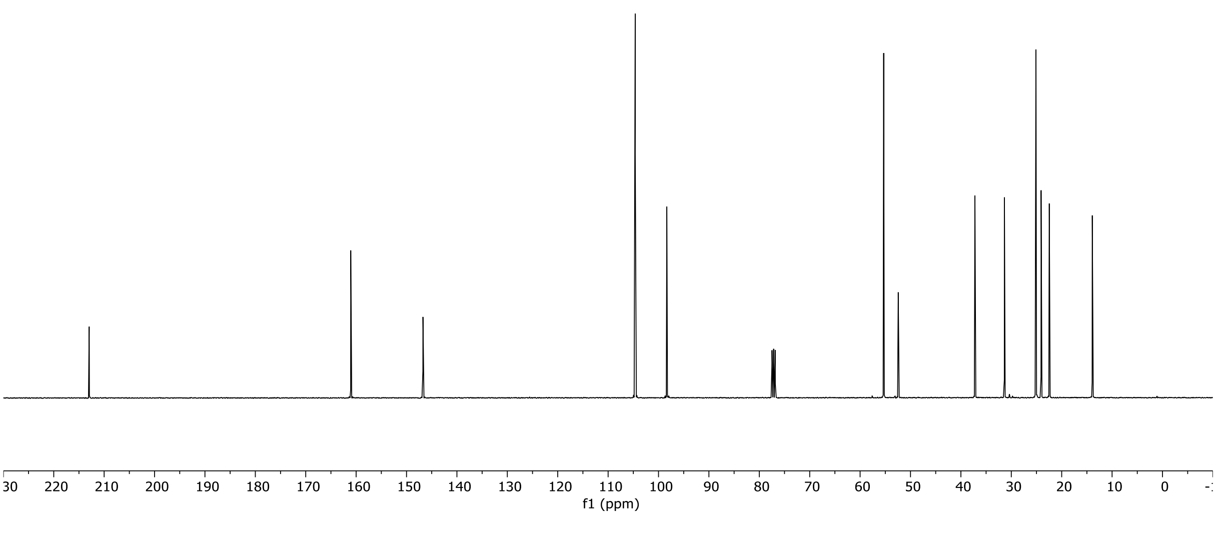

^{1}H NMR (400 MHz, $CDCl_3$) of **8a**

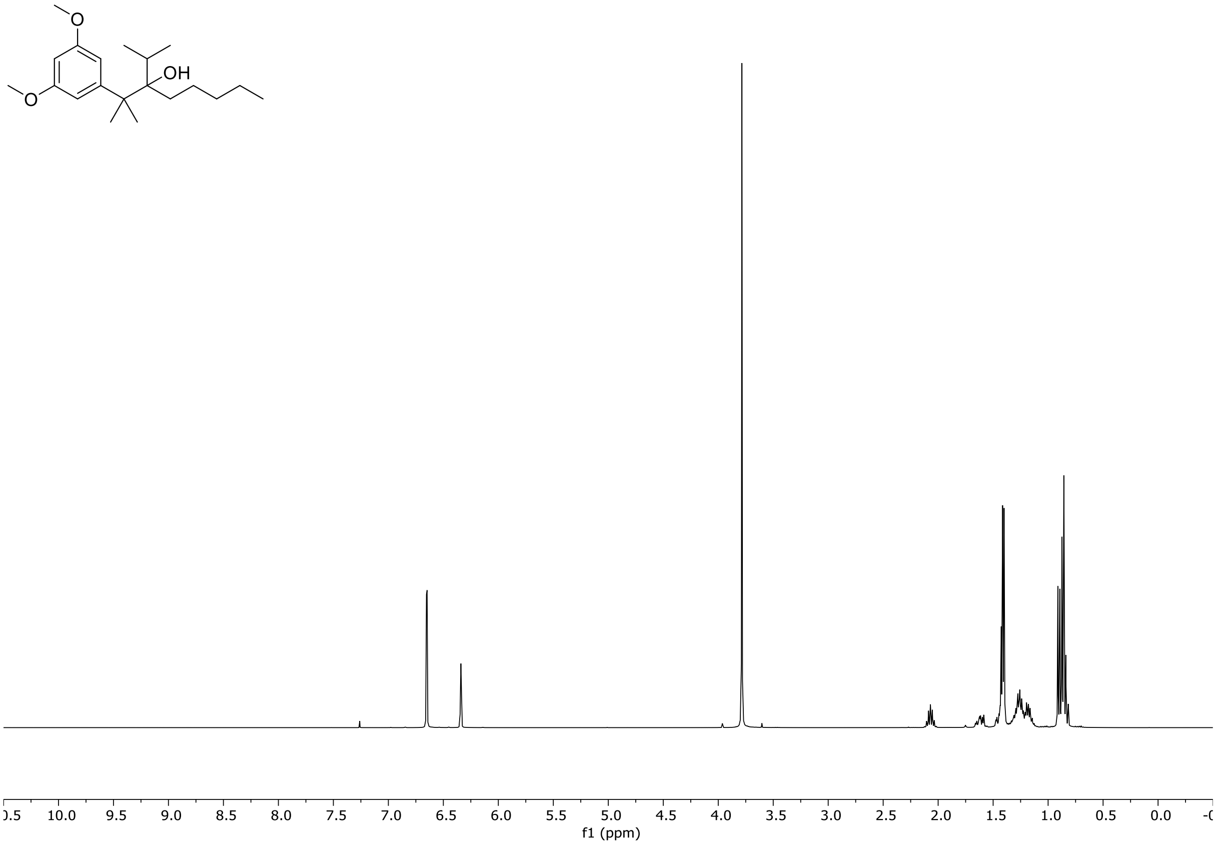

^{13}C NMR (101 MHz, $CDCl_3$) of **8a**

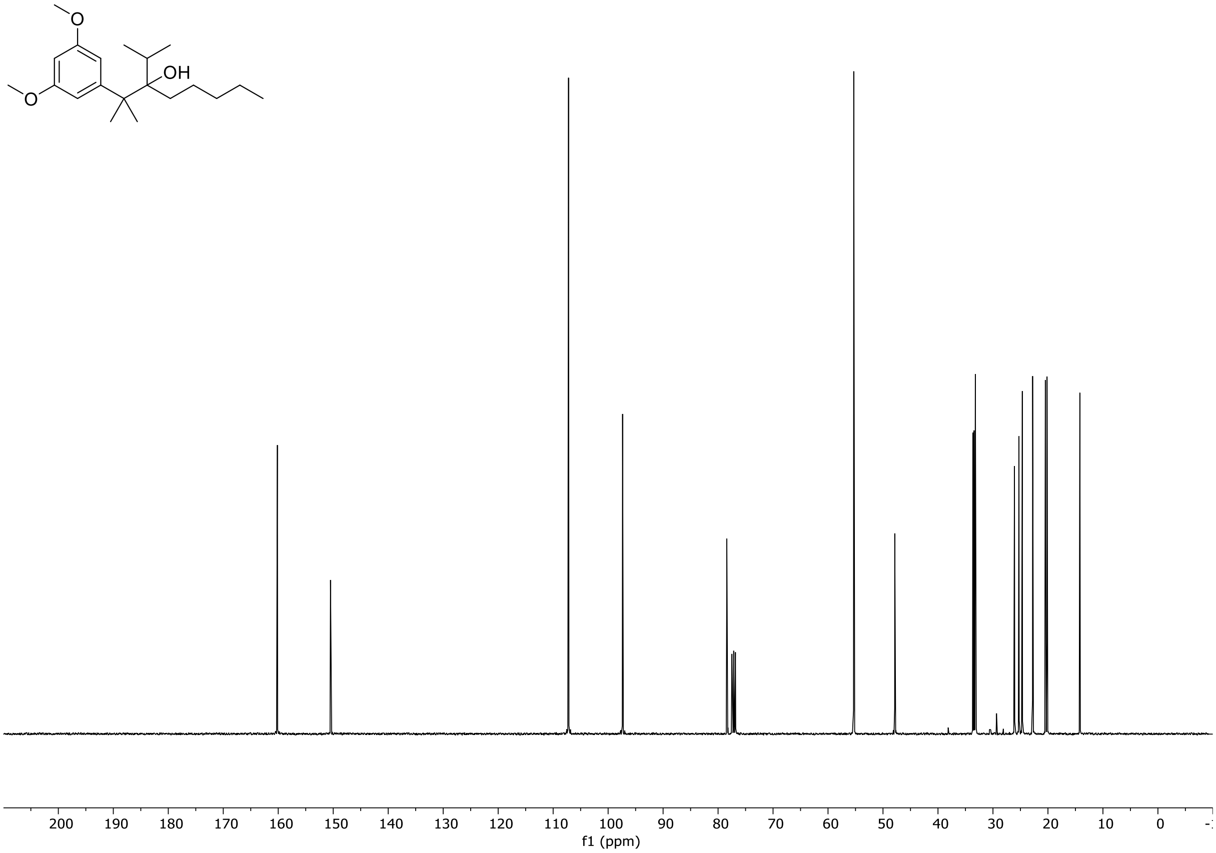

^{1}H NMR (400 MHz, $CDCl_3$) of **8b**

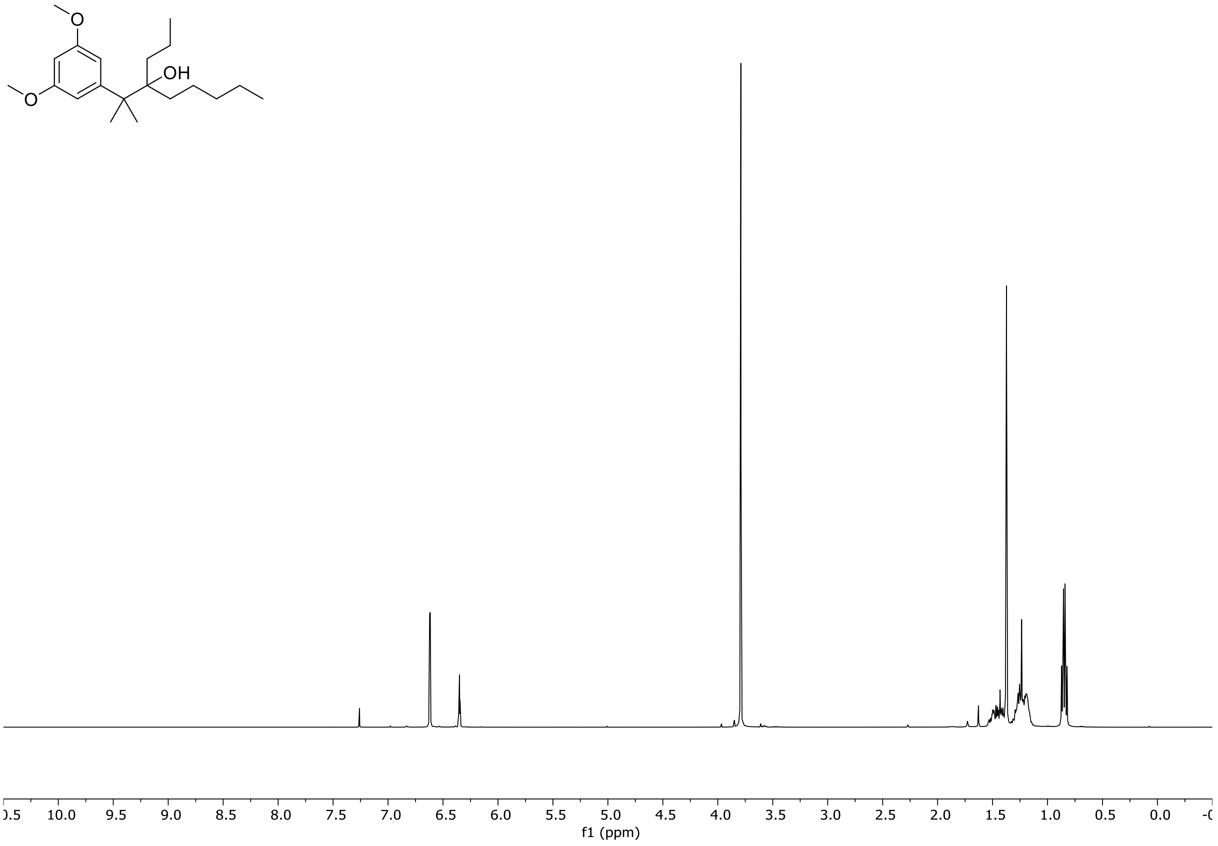

^{13}C NMR (101 MHz, $CDCl_3$) of **8b**

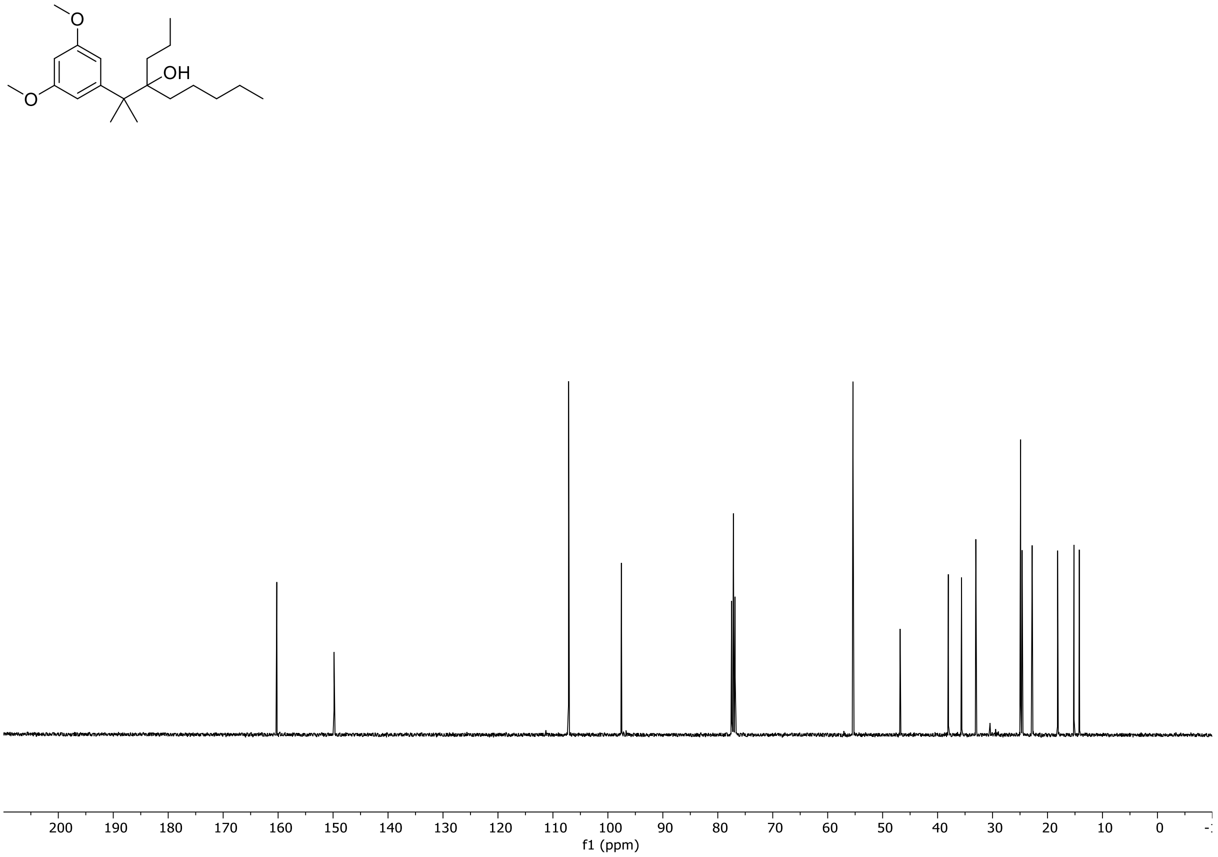

^{1}H NMR (400 MHz, $CDCl_3$) of **8c**

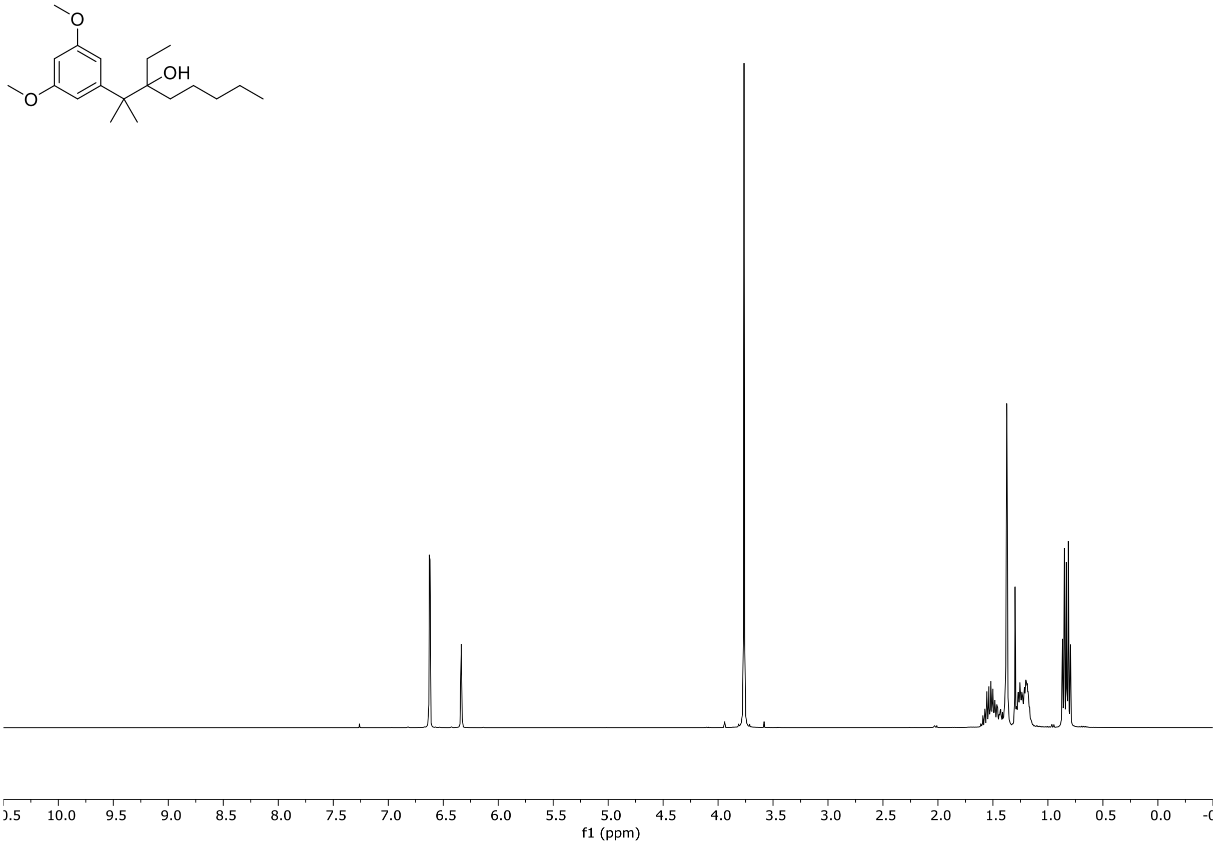

^{13}C NMR (101 MHz, $CDCl_3$) of **8c**

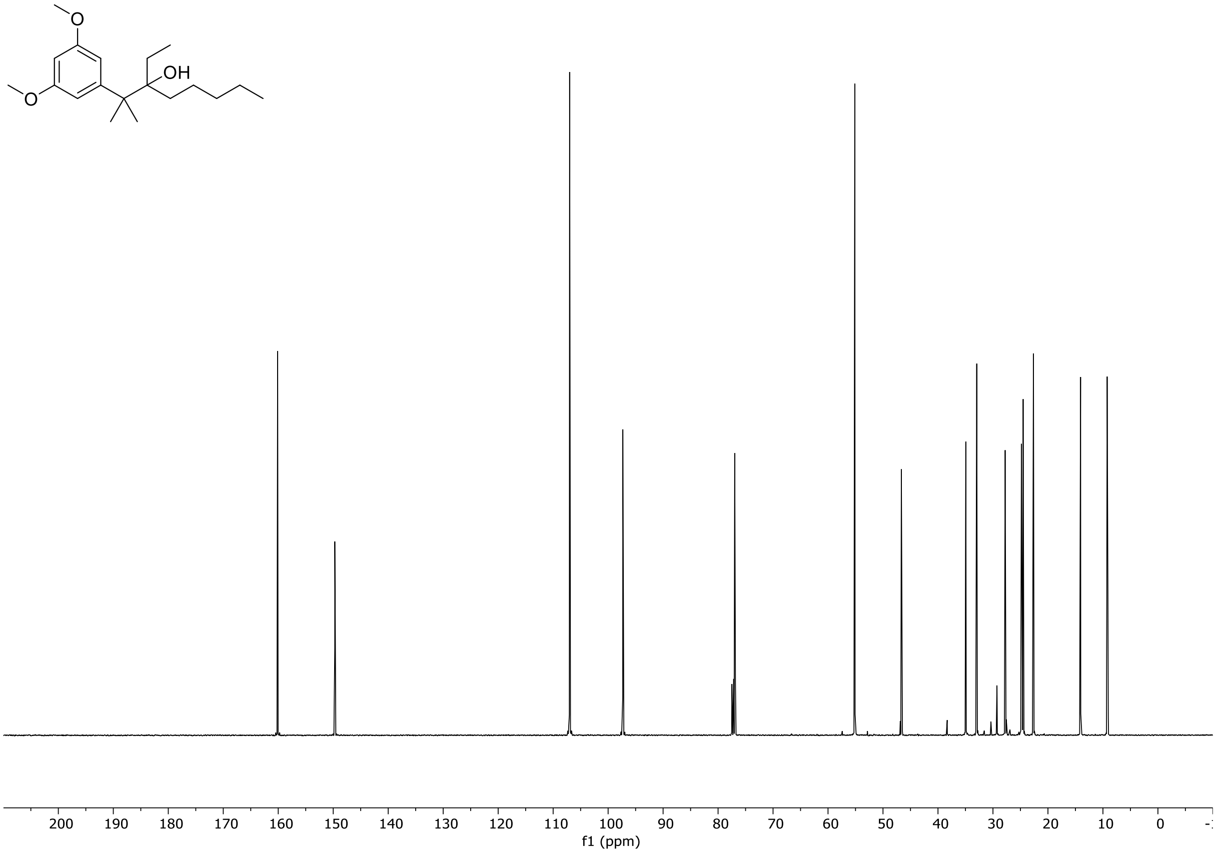

^{1}H NMR (400 MHz, $CDCl_3$) of **8d**

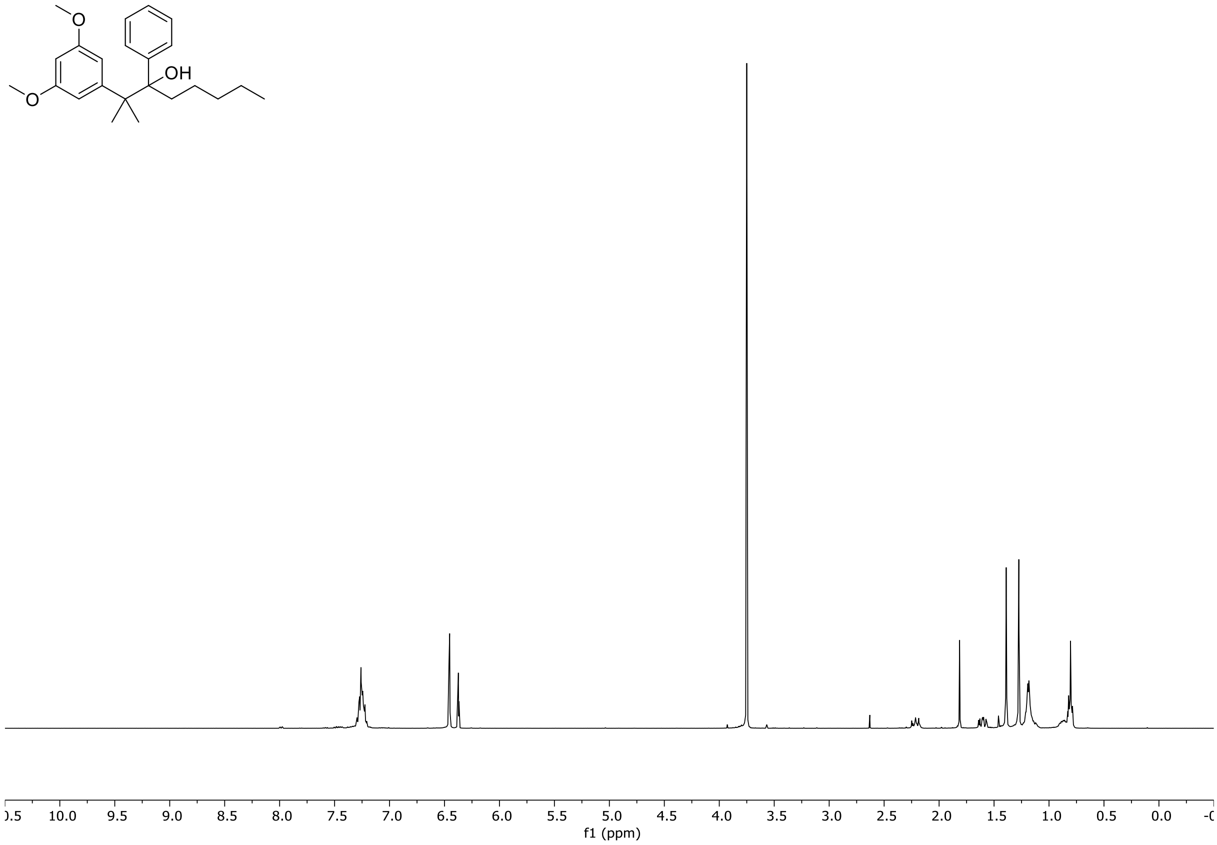

^{13}C NMR (101 MHz, $CDCl_3$) of **8d**

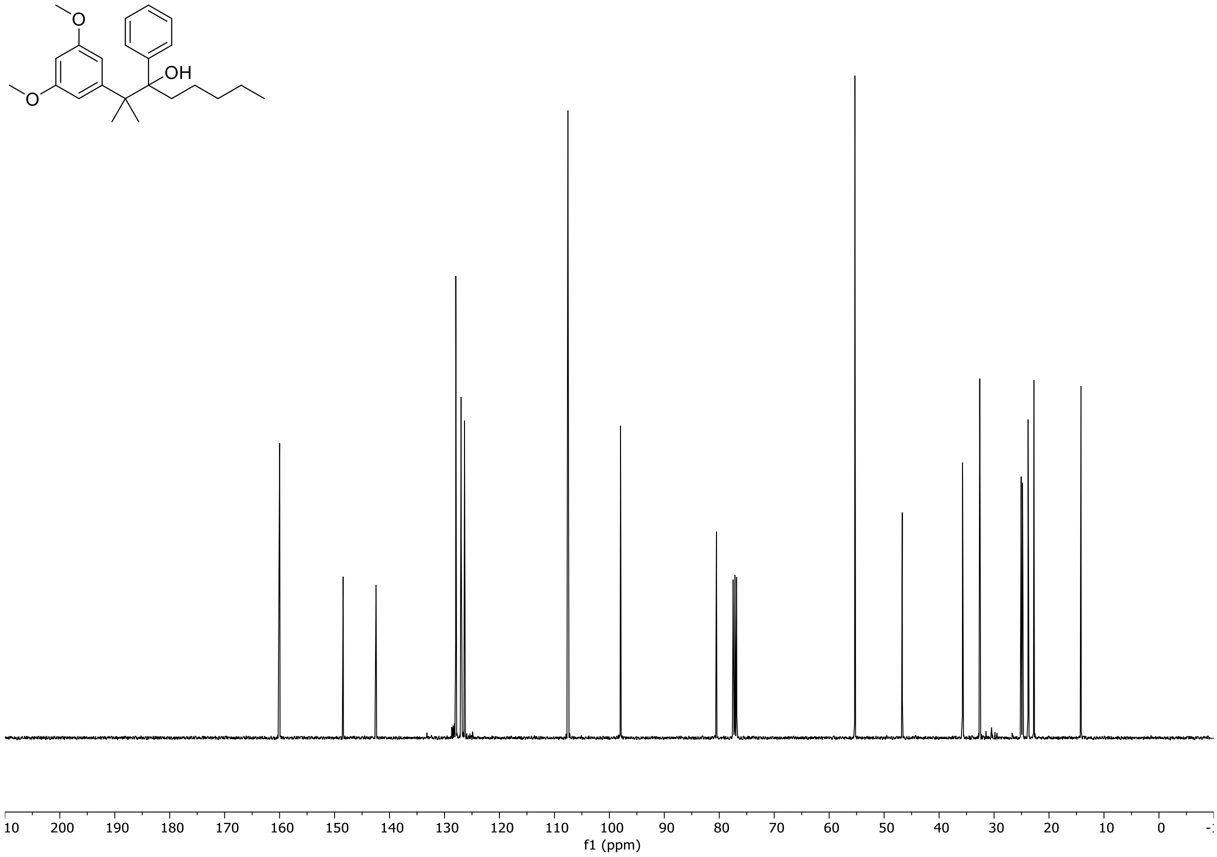

^{1}H NMR (400 MHz, CDCl$_3$) of **8e**

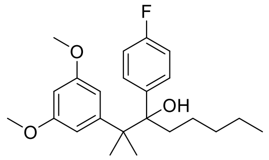

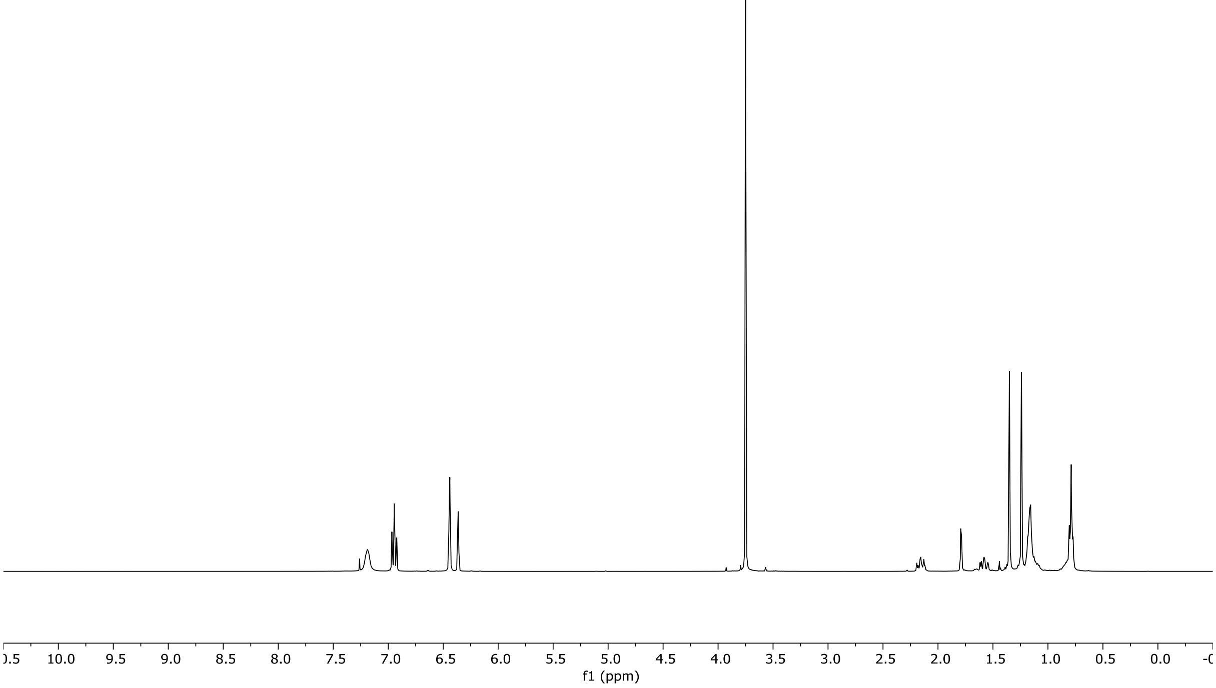

^{13}C NMR (101 MHz, CDCl$_3$) of **8e**

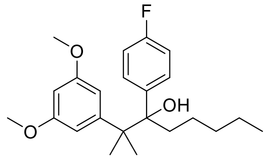

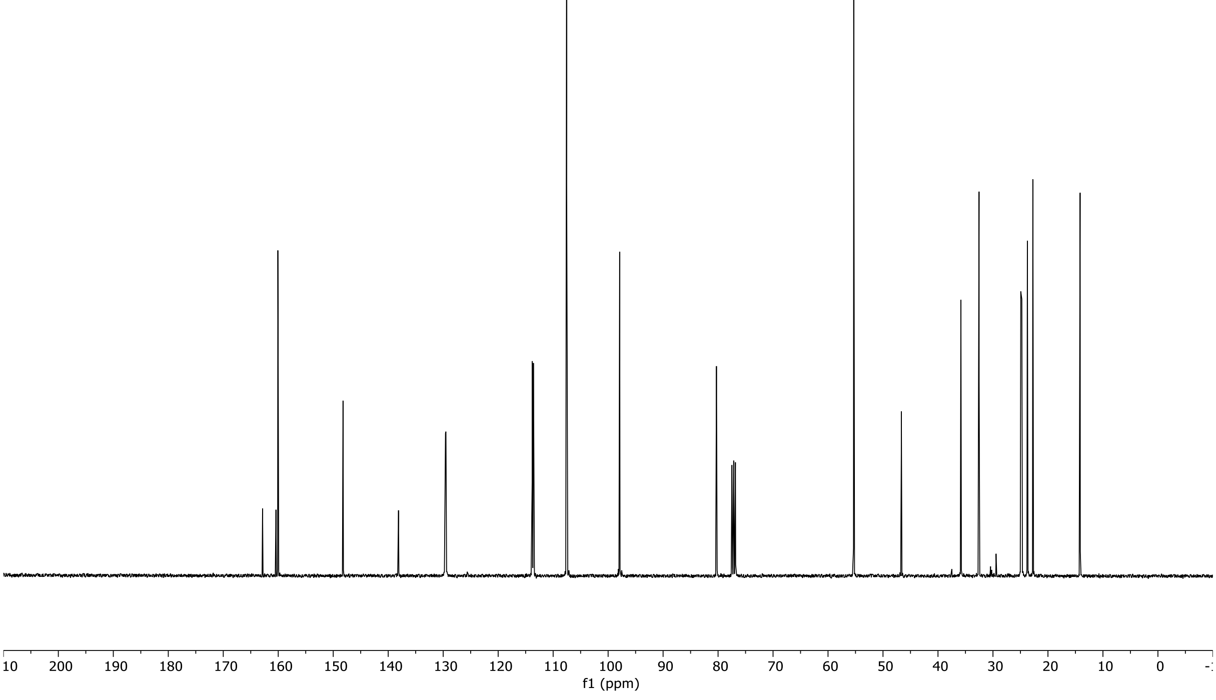

^{19}F NMR (377 MHz, $CDCl_3$) of **8e**

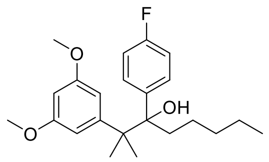

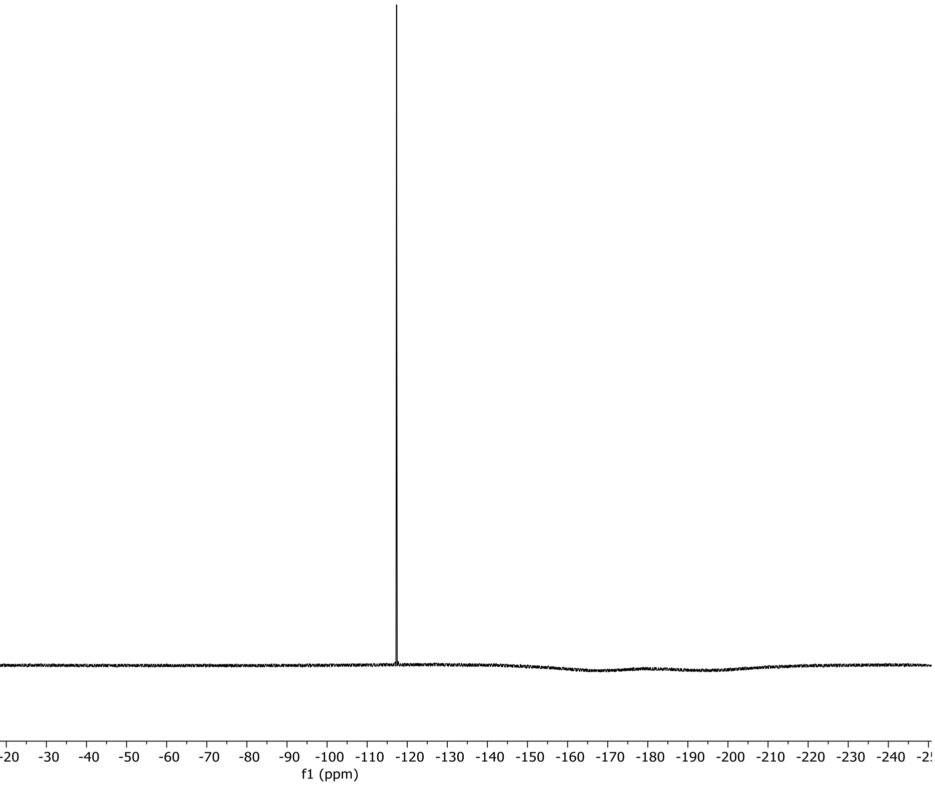

^{1}H NMR (400 MHz, $CDCl_3$) of **8f**

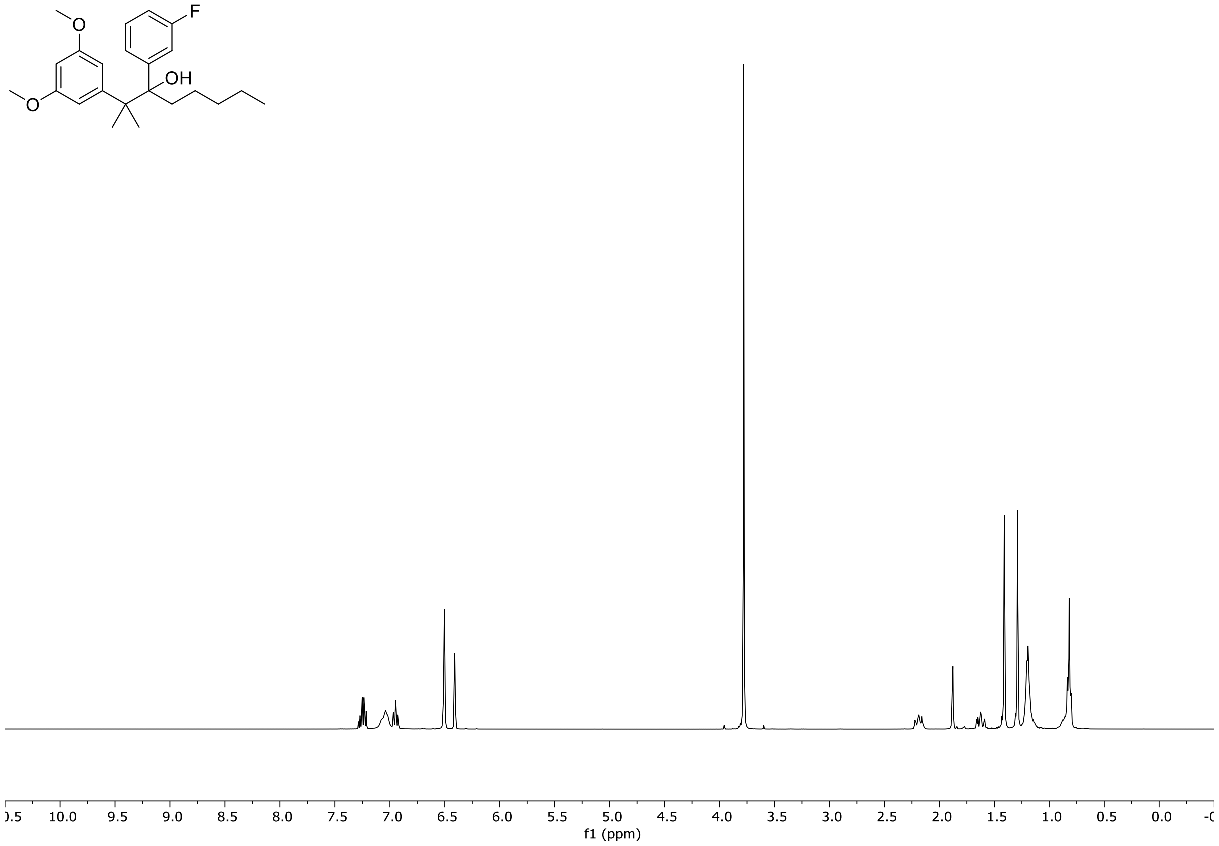

^{13}C NMR (101 MHz, $CDCl_3$) of **8f**

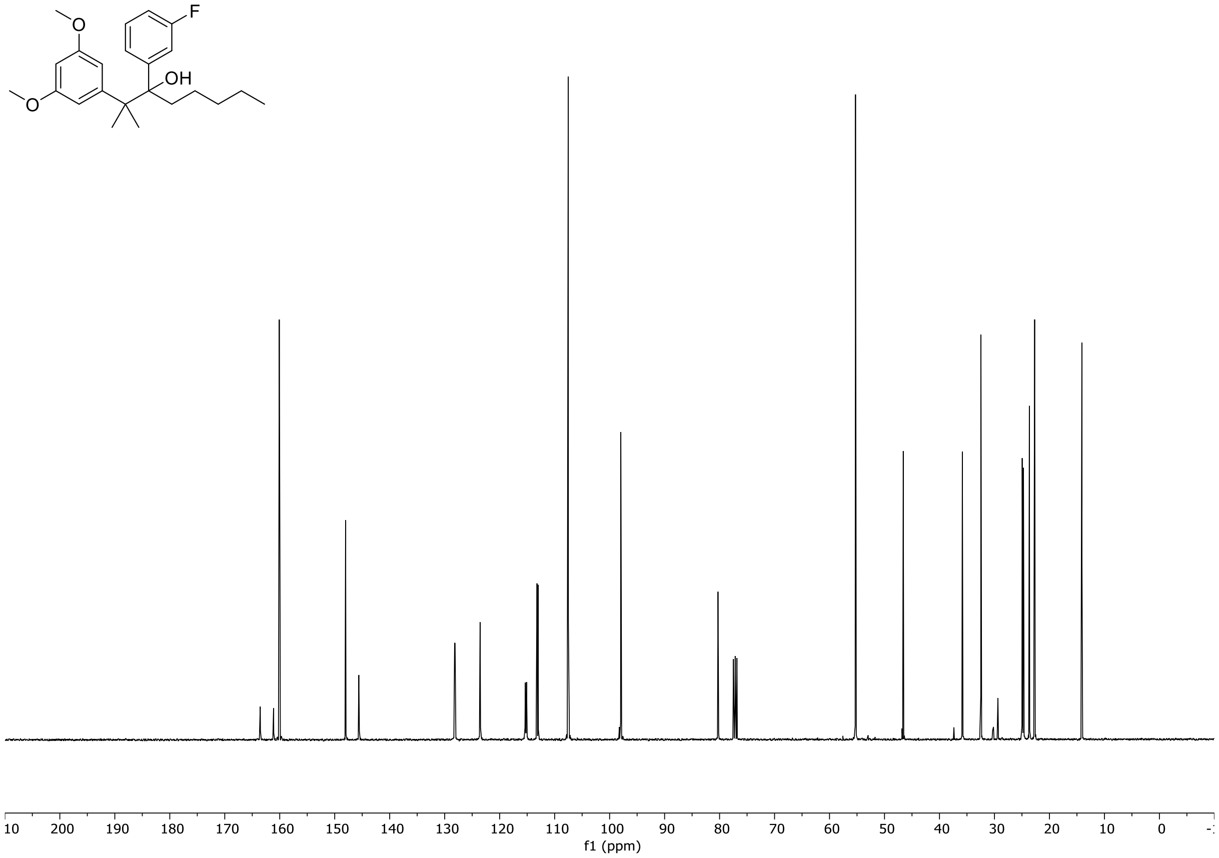

^{19}F NMR (377 MHz, $CDCl_3$) of **8f**

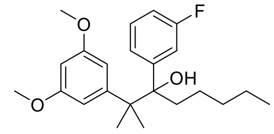

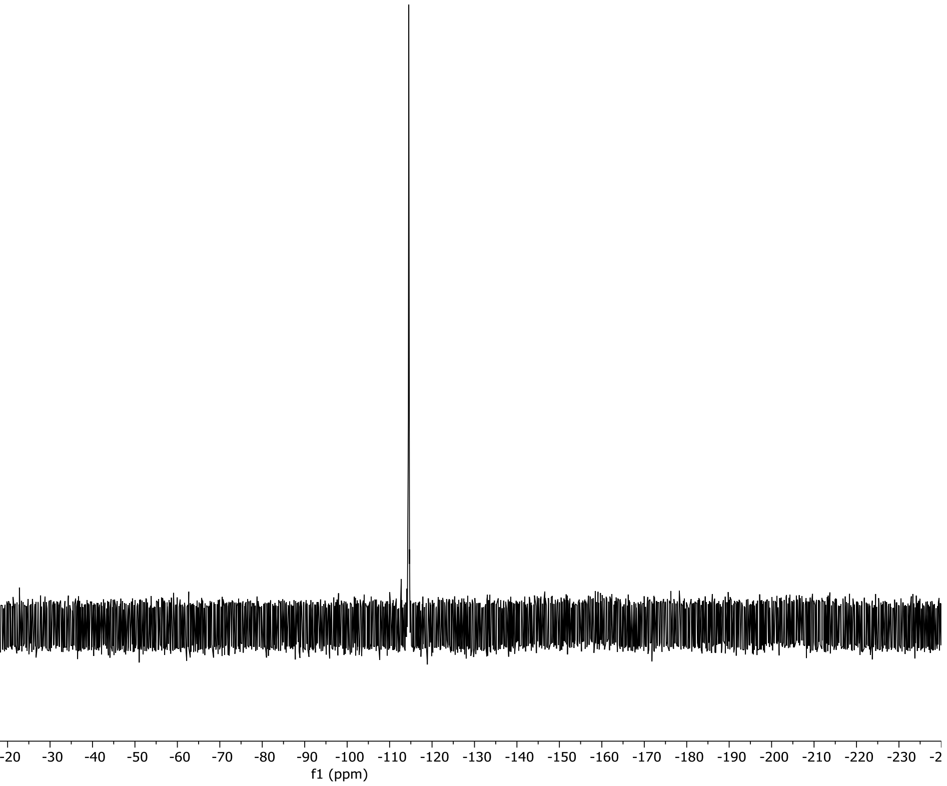

^1H NMR (400 MHz, $CDCl_3$) of **SI-15a**

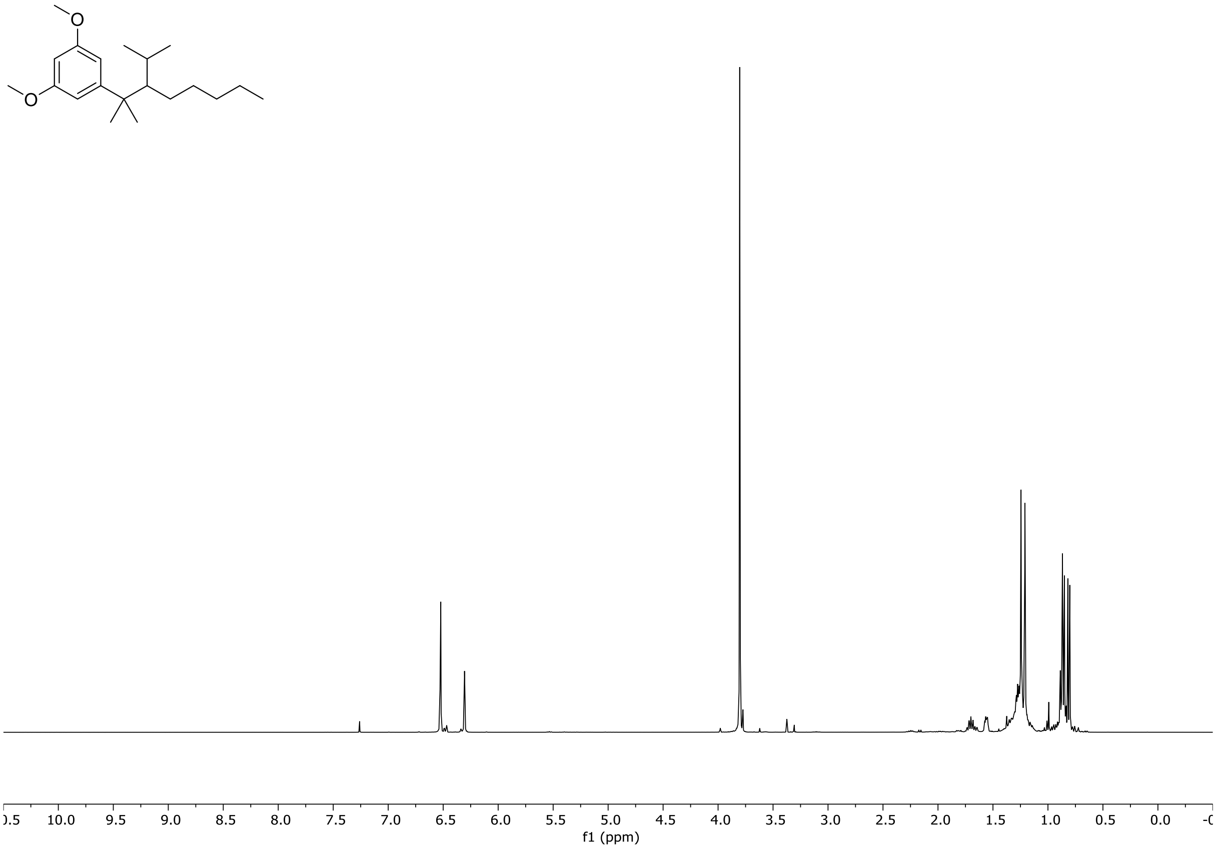

^{13}C NMR (101 MHz, $CDCl_3$) of **SI-15a**

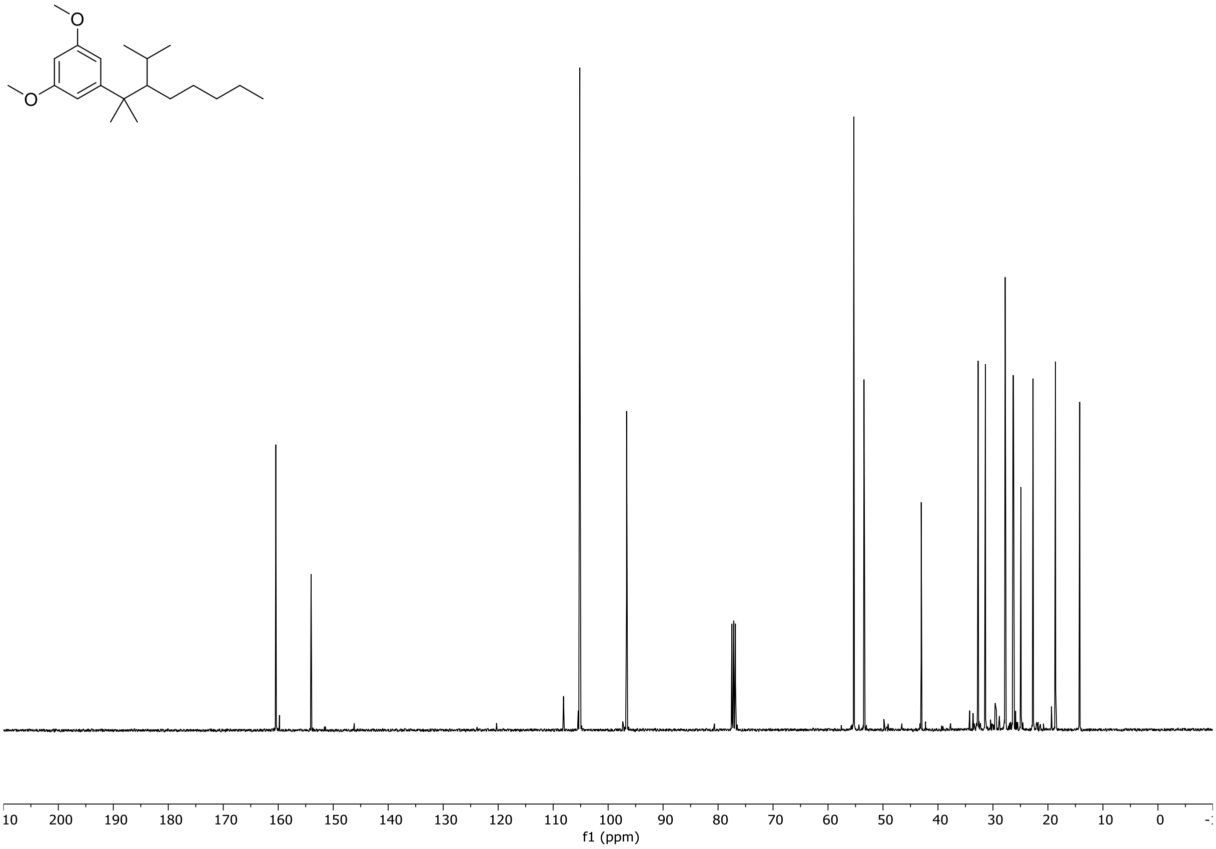

^{1}H NMR (400 MHz, $CDCl_3$) of **SI-15b**

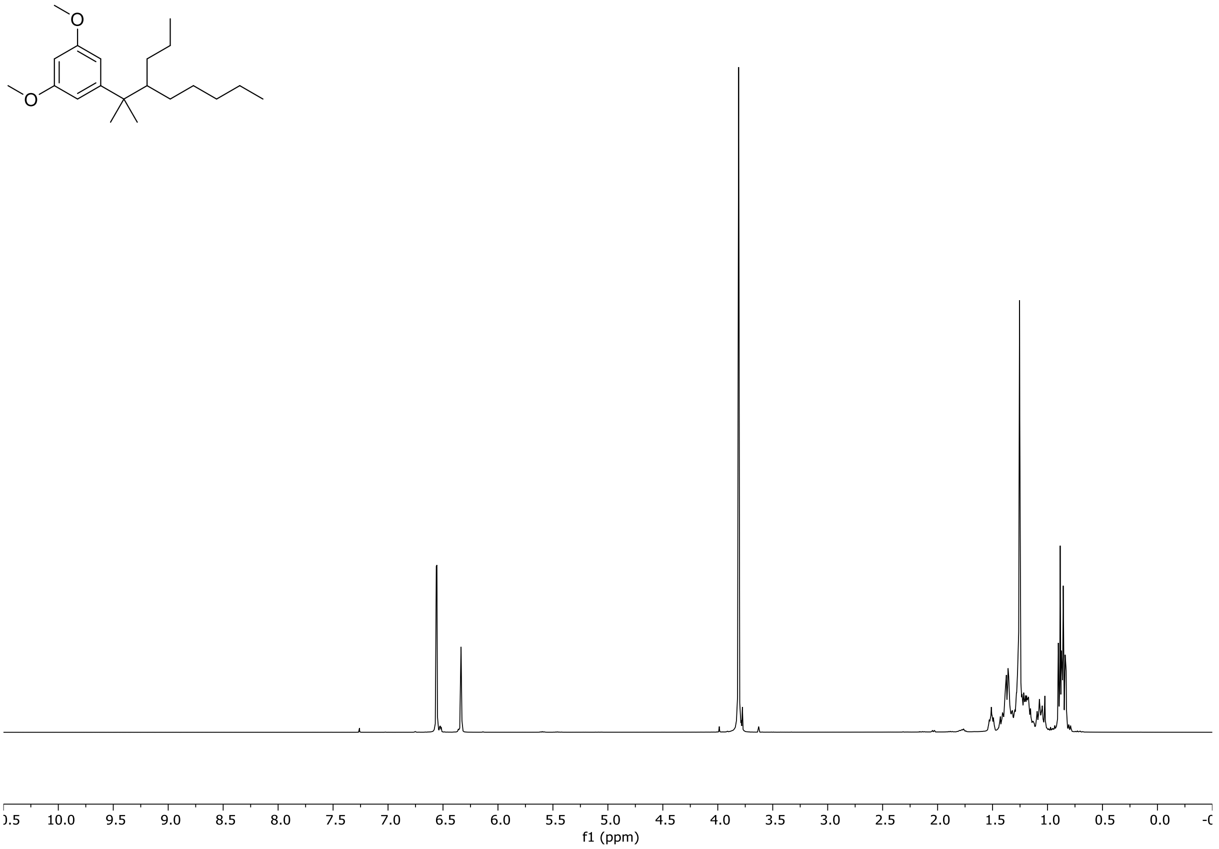

^{13}C NMR (101 MHz, $CDCl_3$) of **SI-15b**

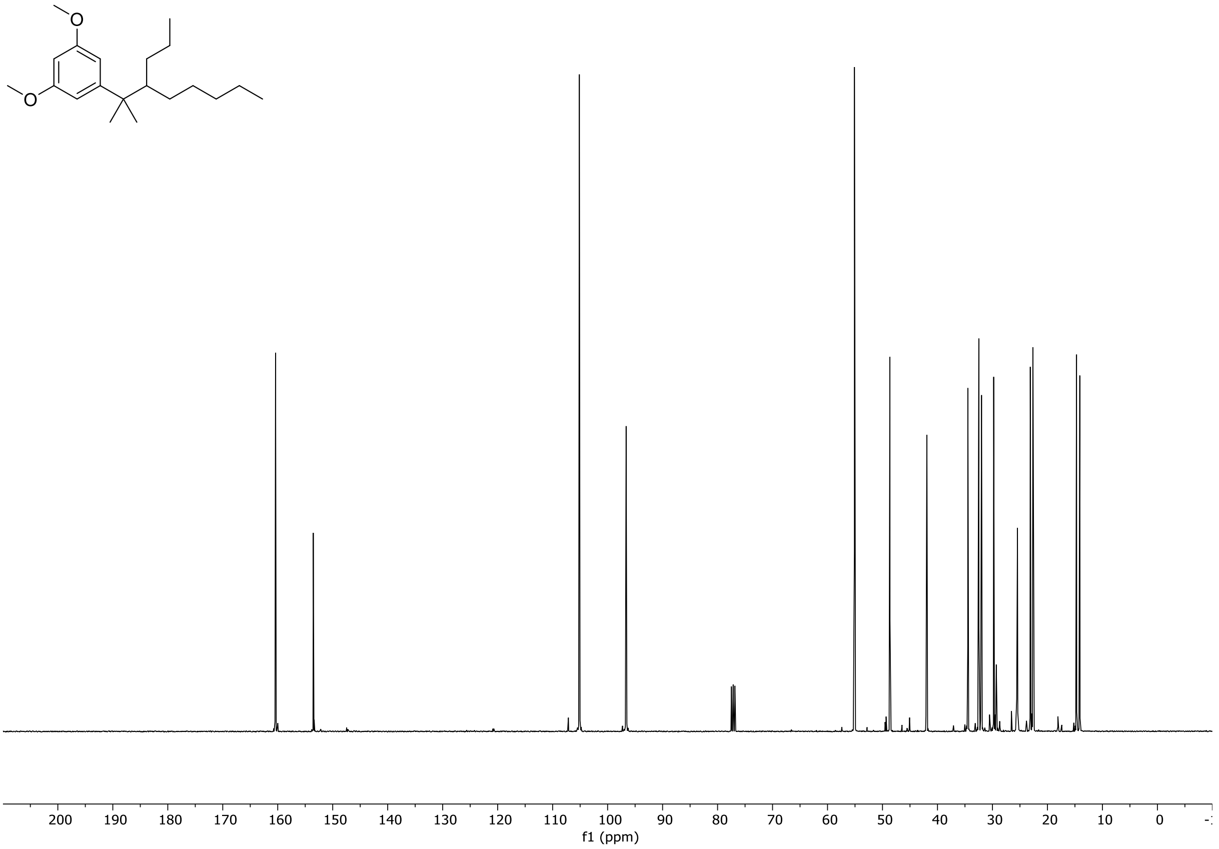

^{1}H NMR (400 MHz, $CDCl_3$) of **SI-15c**

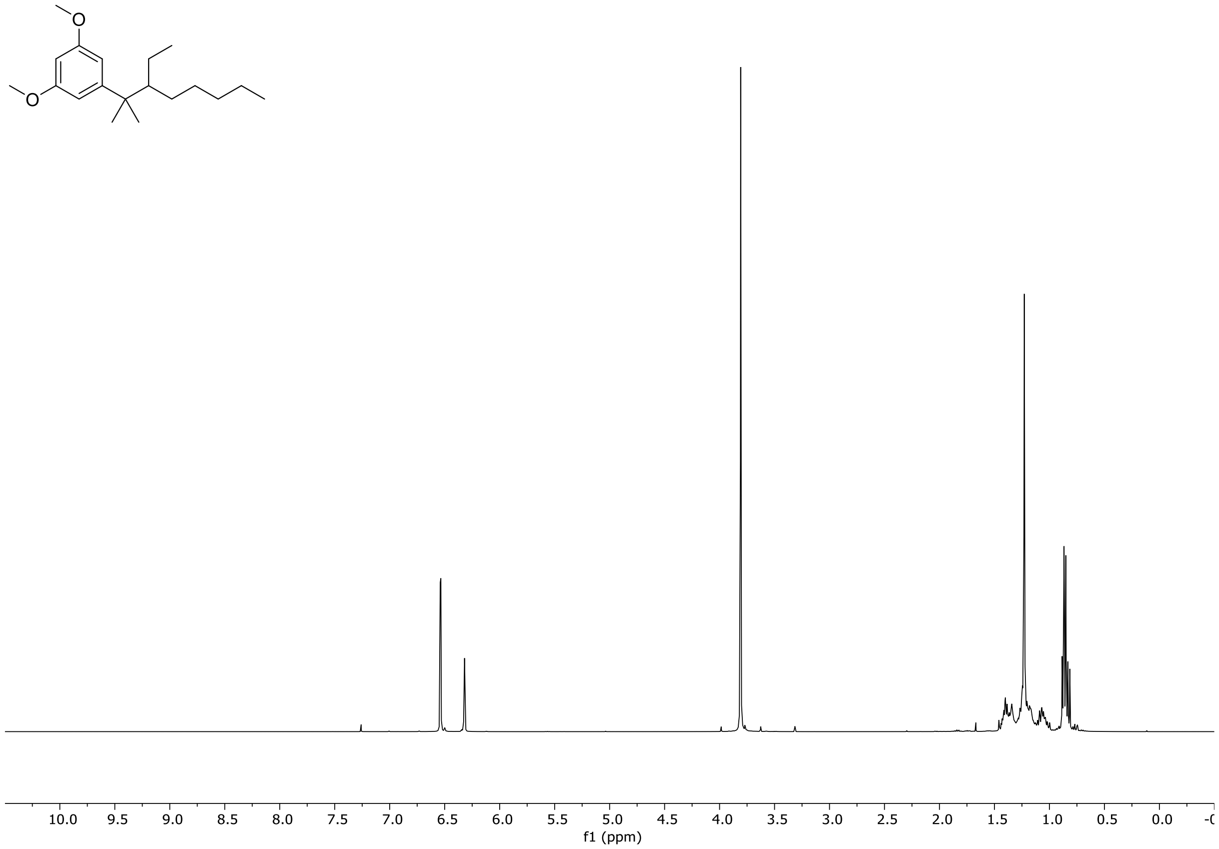

^{13}C NMR (101 MHz, $CDCl_3$) of **SI-15c**

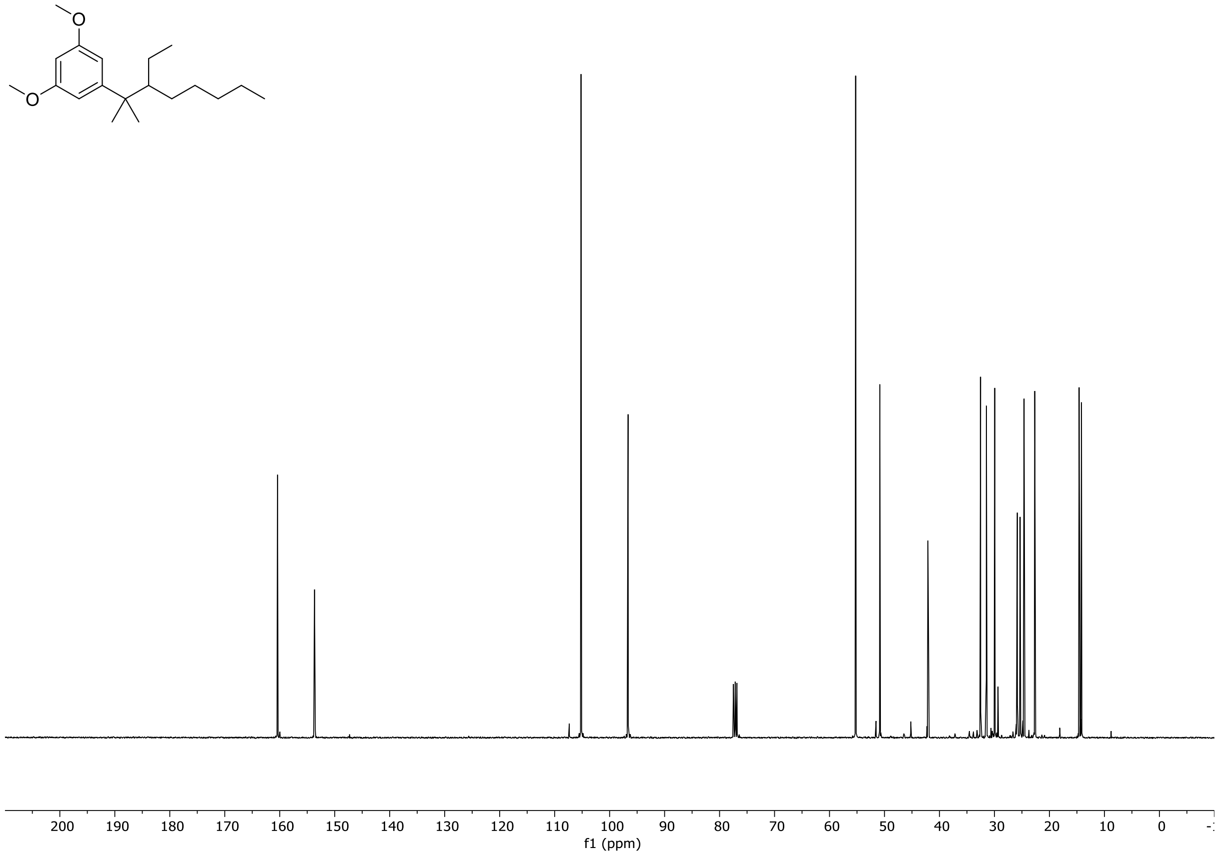

^{1}H NMR (400 MHz, $CDCl_3$) of **9a**

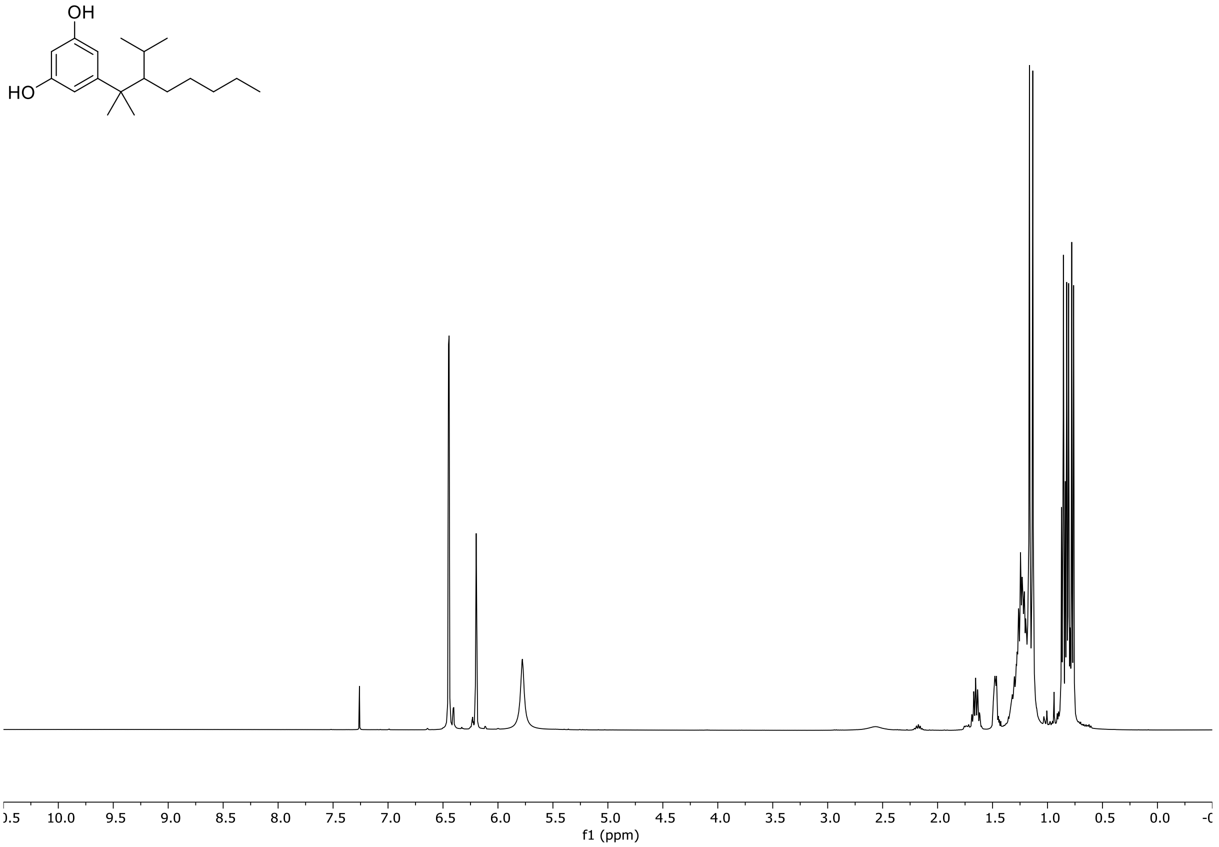

^{13}C NMR (101 MHz, $CDCl_3$) of **9a**

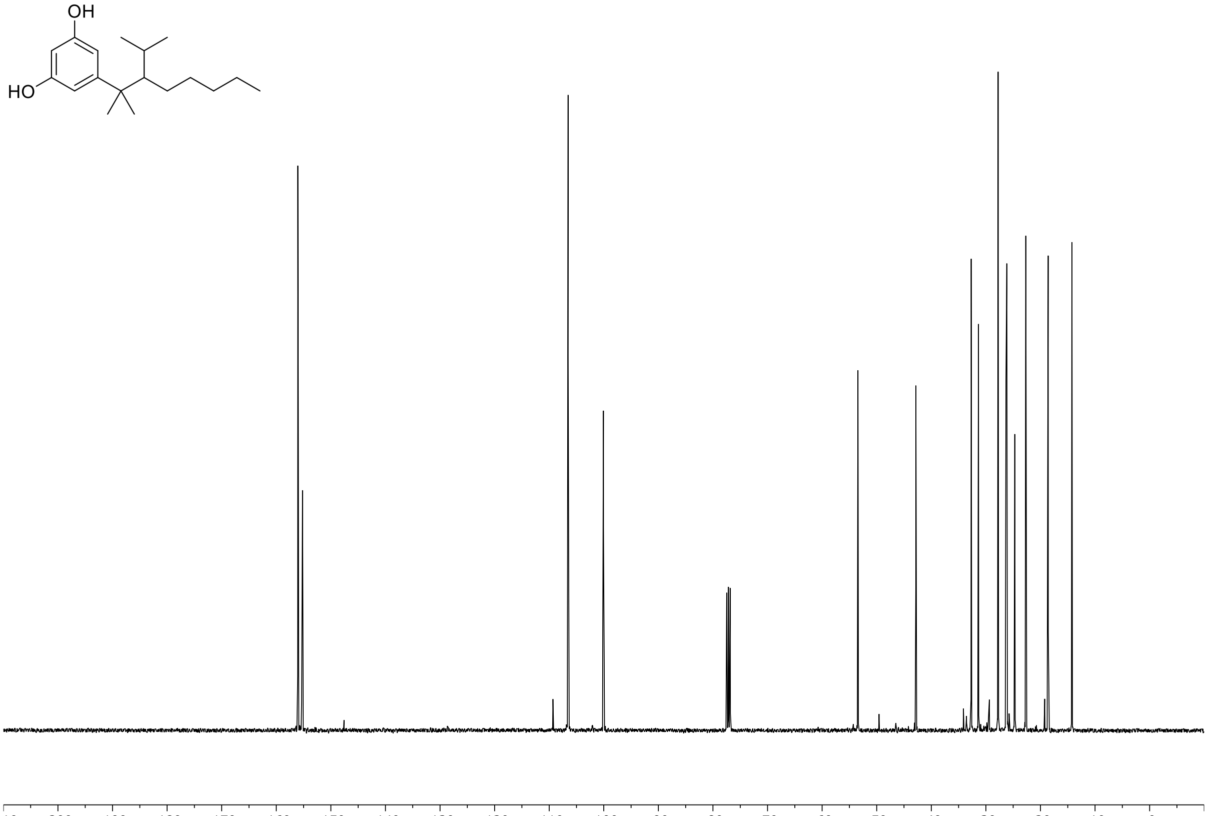

^{1}H NMR (400 MHz, $CDCl_3$) of **9b**

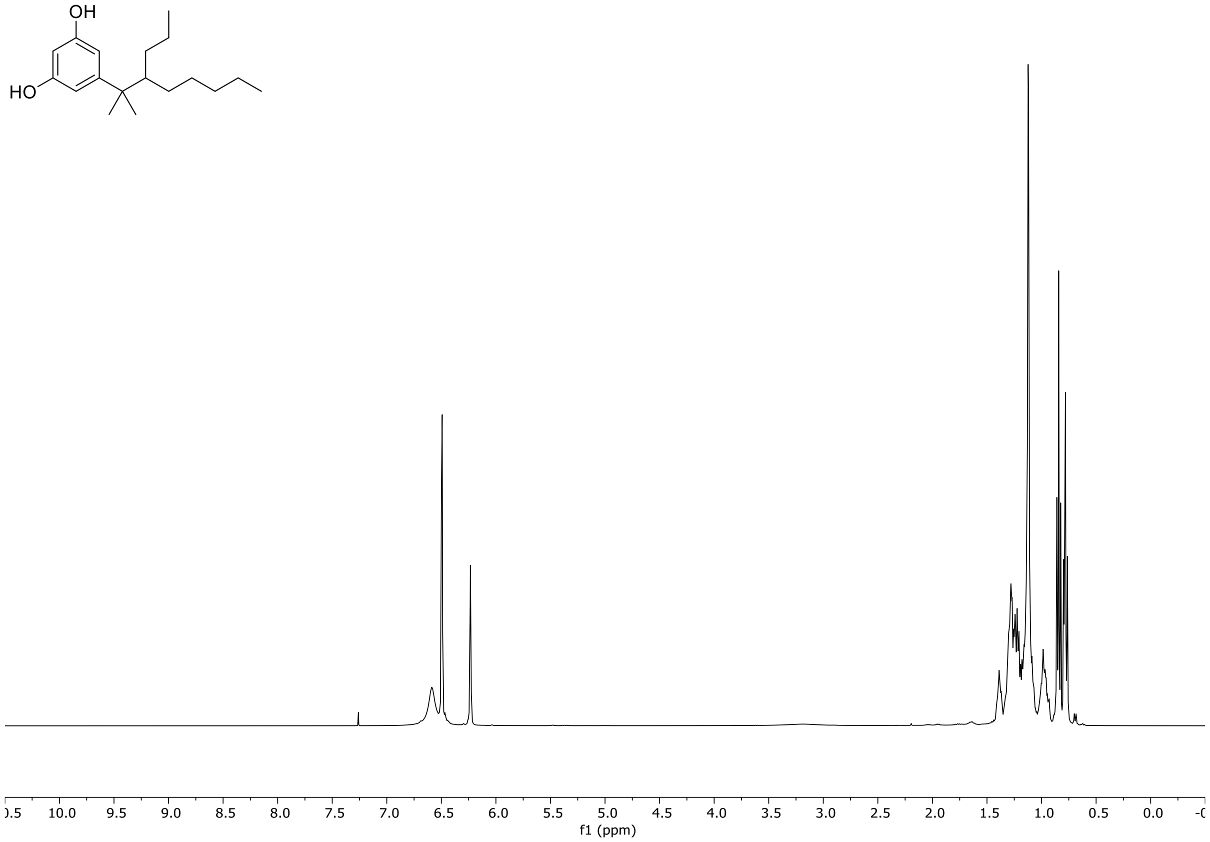

^{13}C NMR (101 MHz, $CDCl_3$) of **9b**

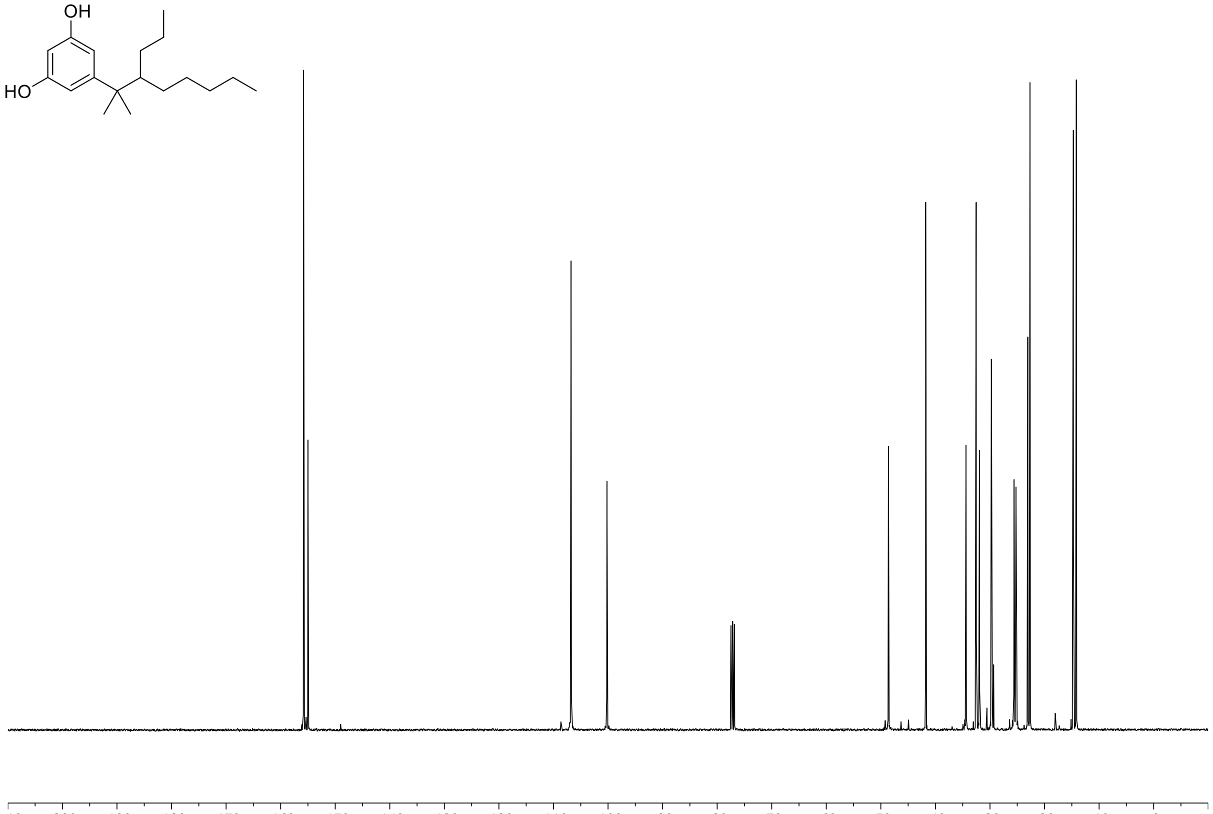

^{1}H NMR (400 MHz, $CDCl_3$) of **9c**

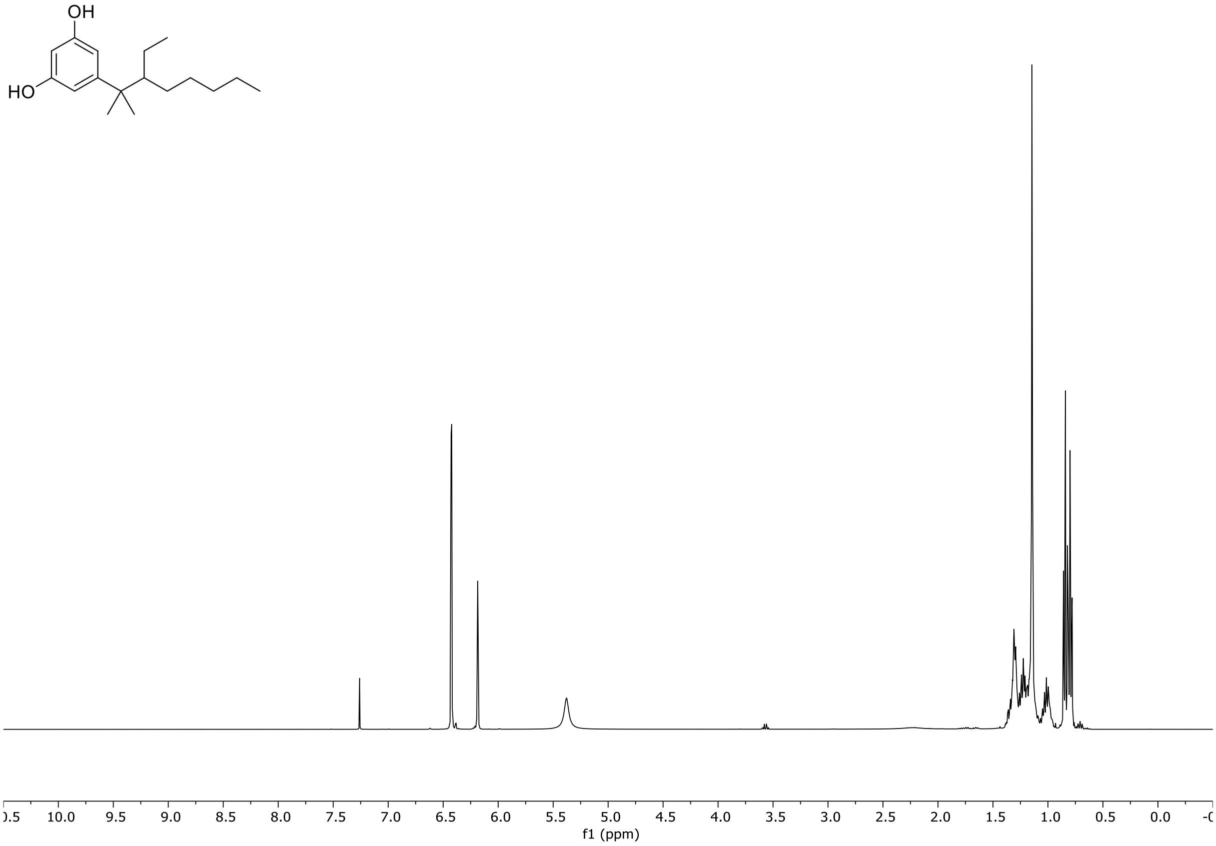

^{13}C NMR (101 MHz, $CDCl_3$) of **9c**

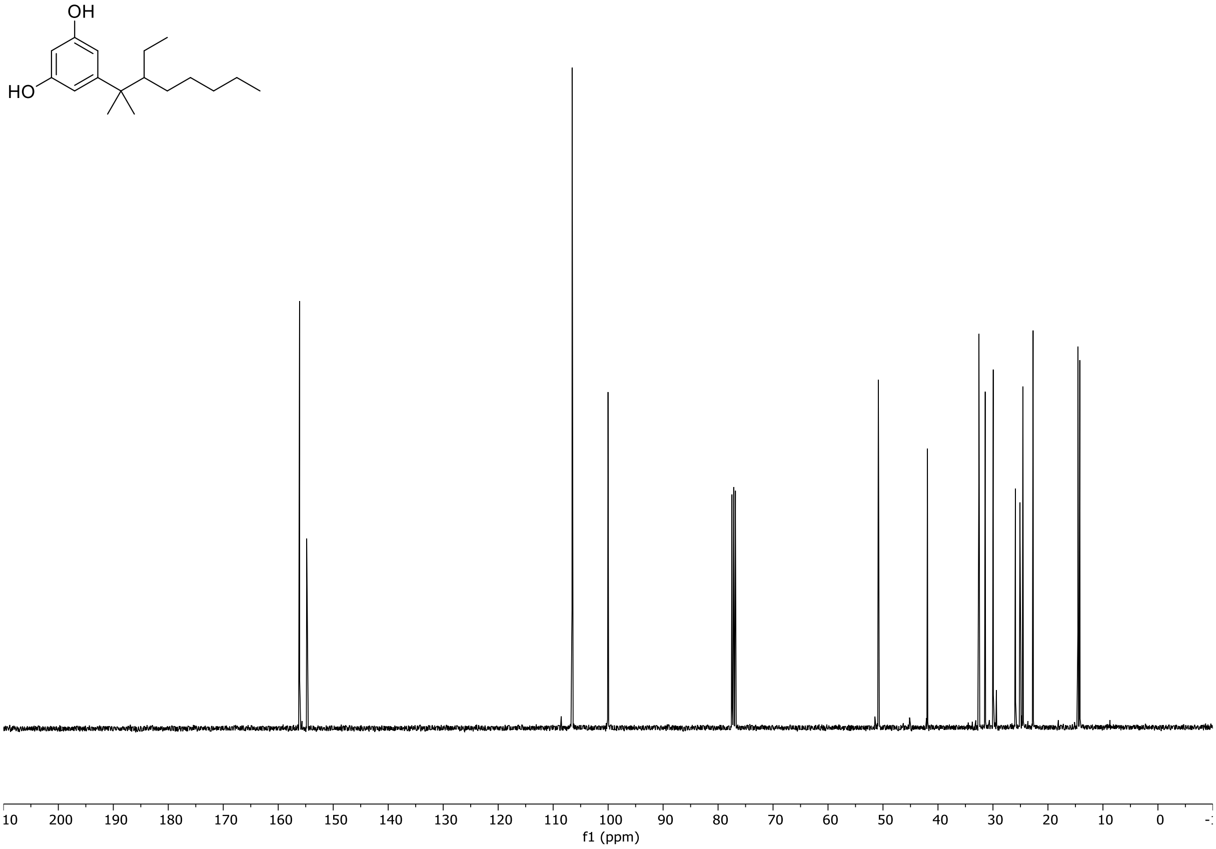

^{1}H NMR (400 MHz, $CDCl_3$) of **SI-16d**

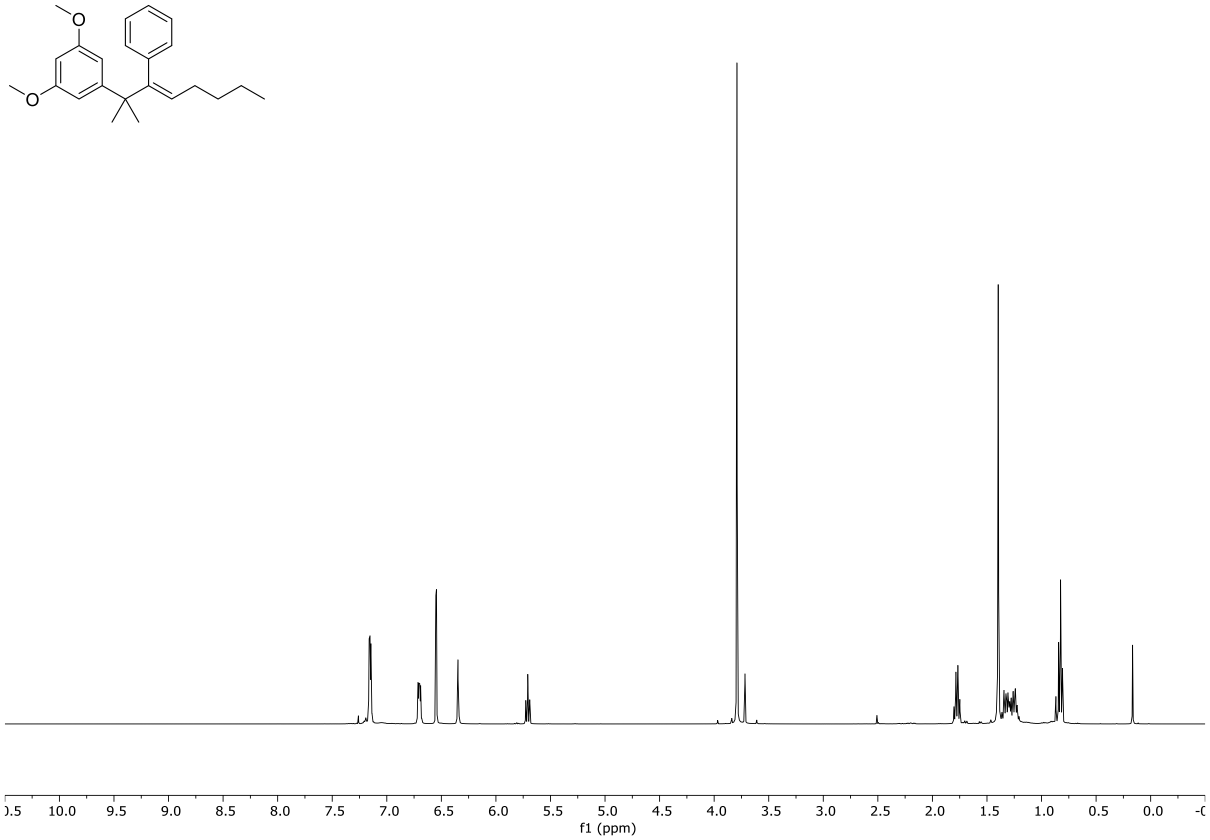

^{13}C NMR (101 MHz, $CDCl_3$) of **SI-16d**

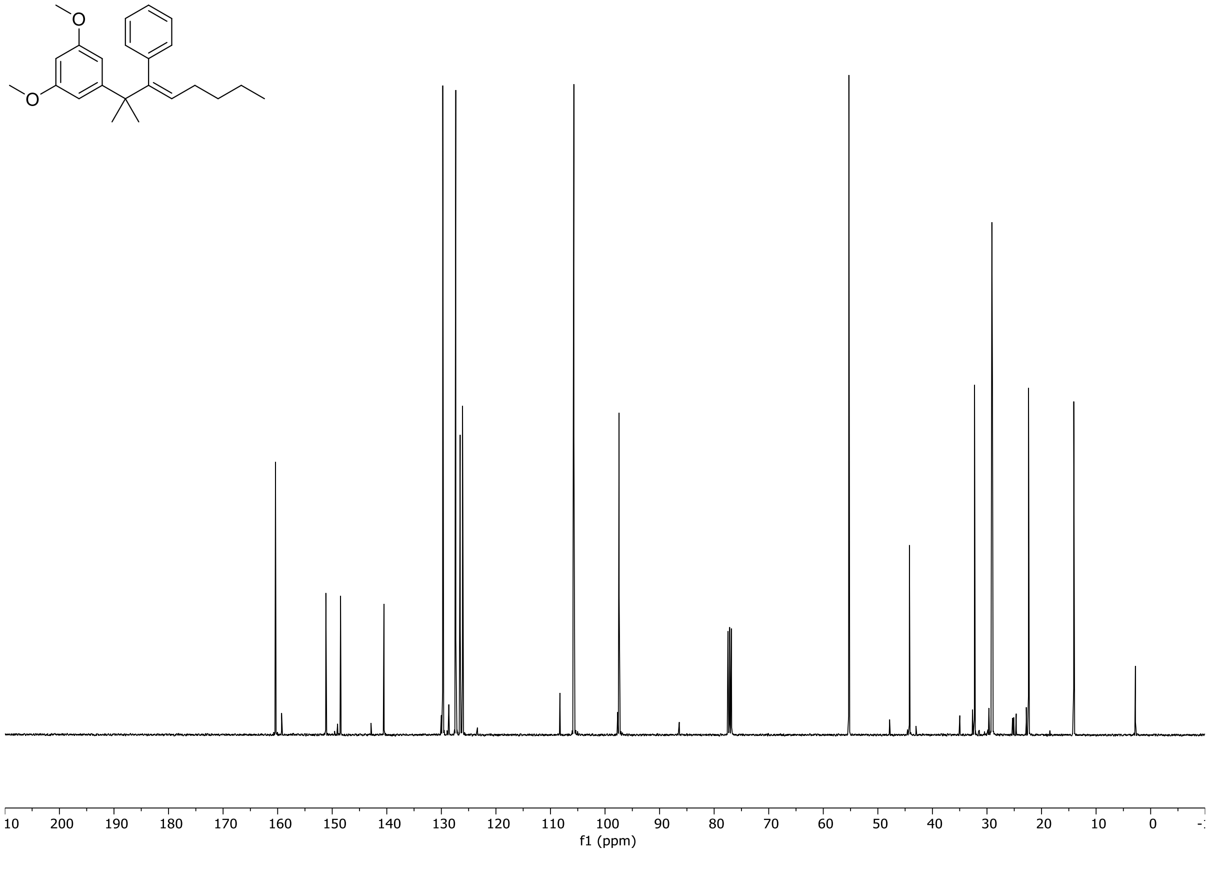

^{1}H NMR (400 MHz, $CDCl_3$) of **SI-16e**

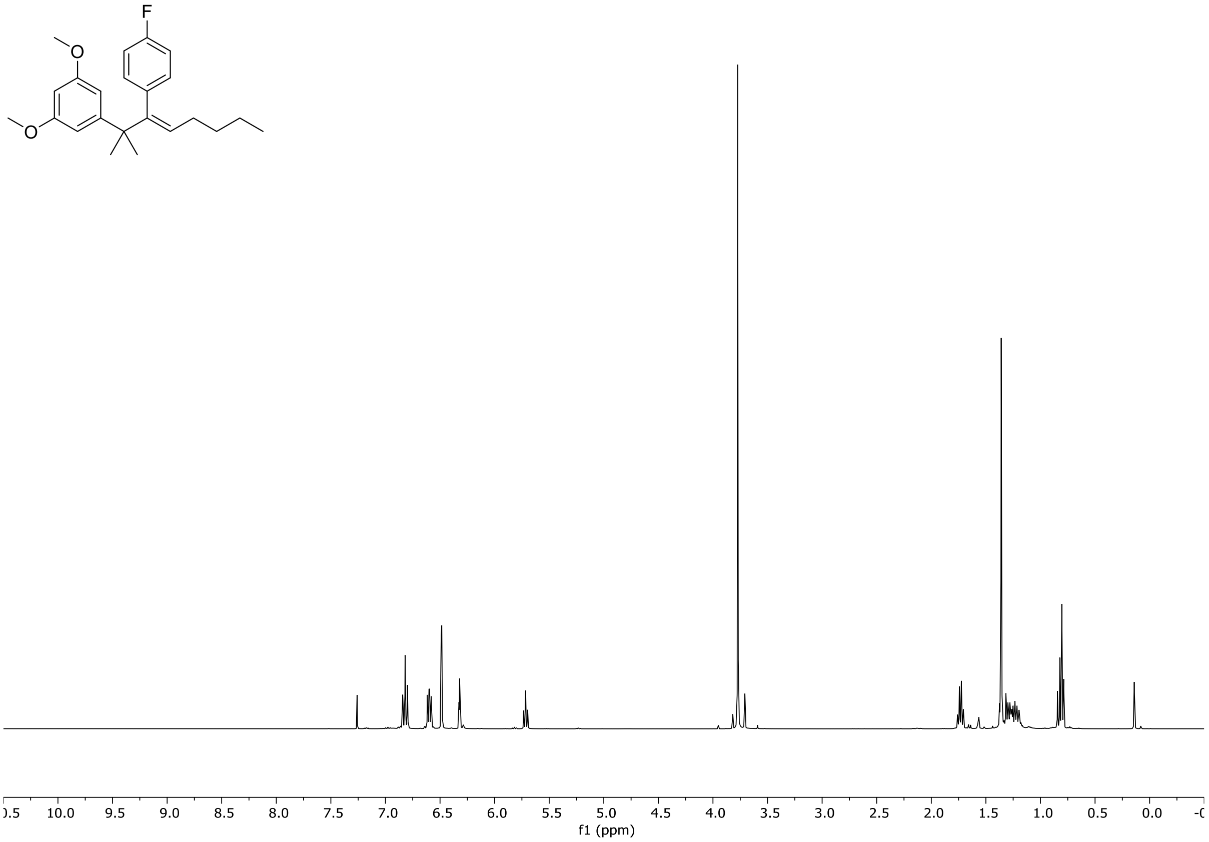

^{13}C NMR (101 MHz, $CDCl_3$) of **SI-16e**

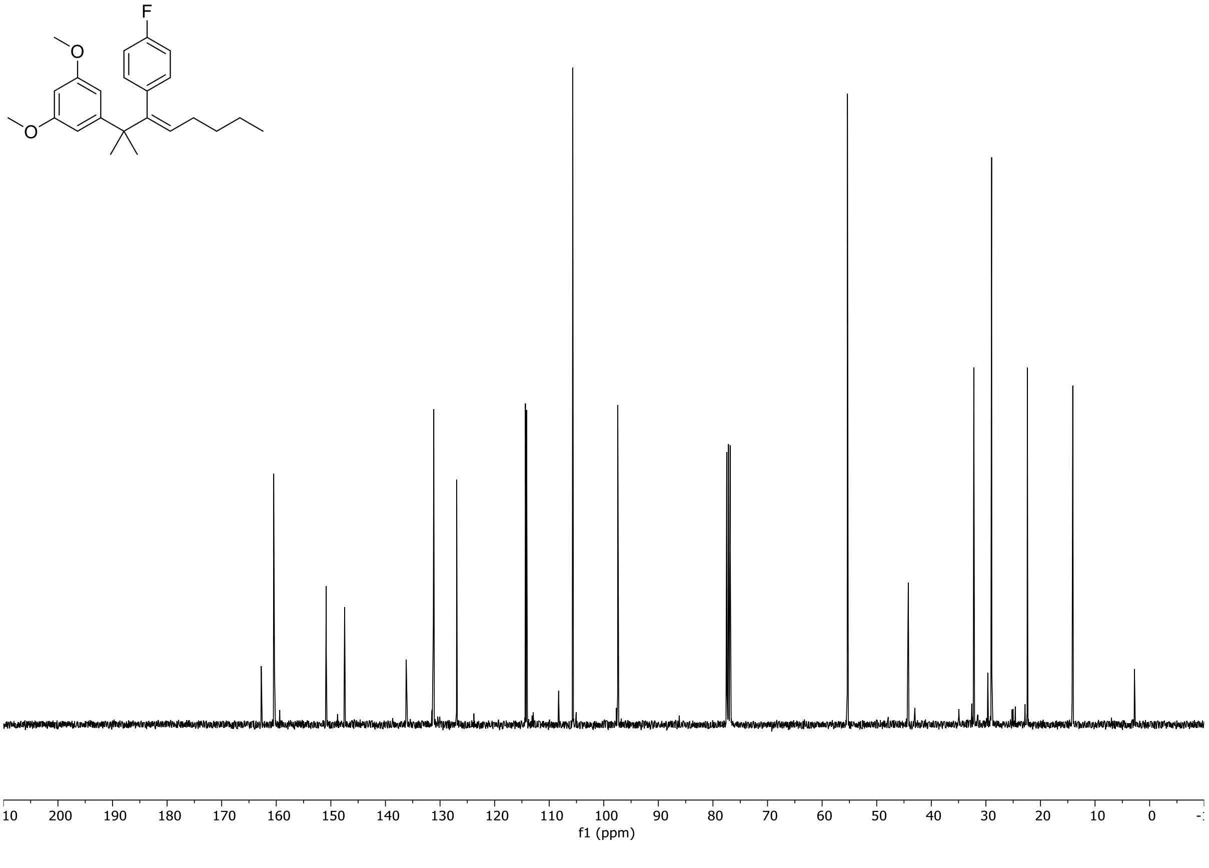

^{19}F NMR (377 MHz, $CDCl_3$) of **SI-16e**

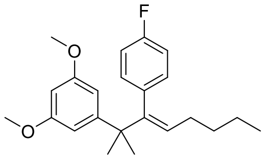

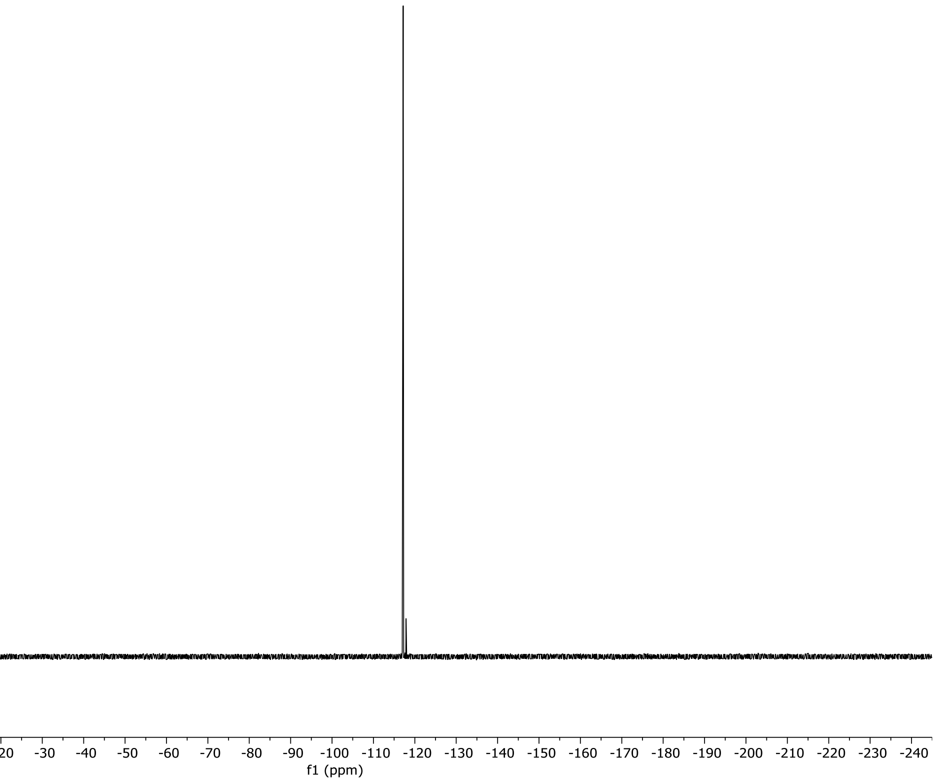

^{1}H NMR (400 MHz, $CDCl_3$) of **SI-16f**

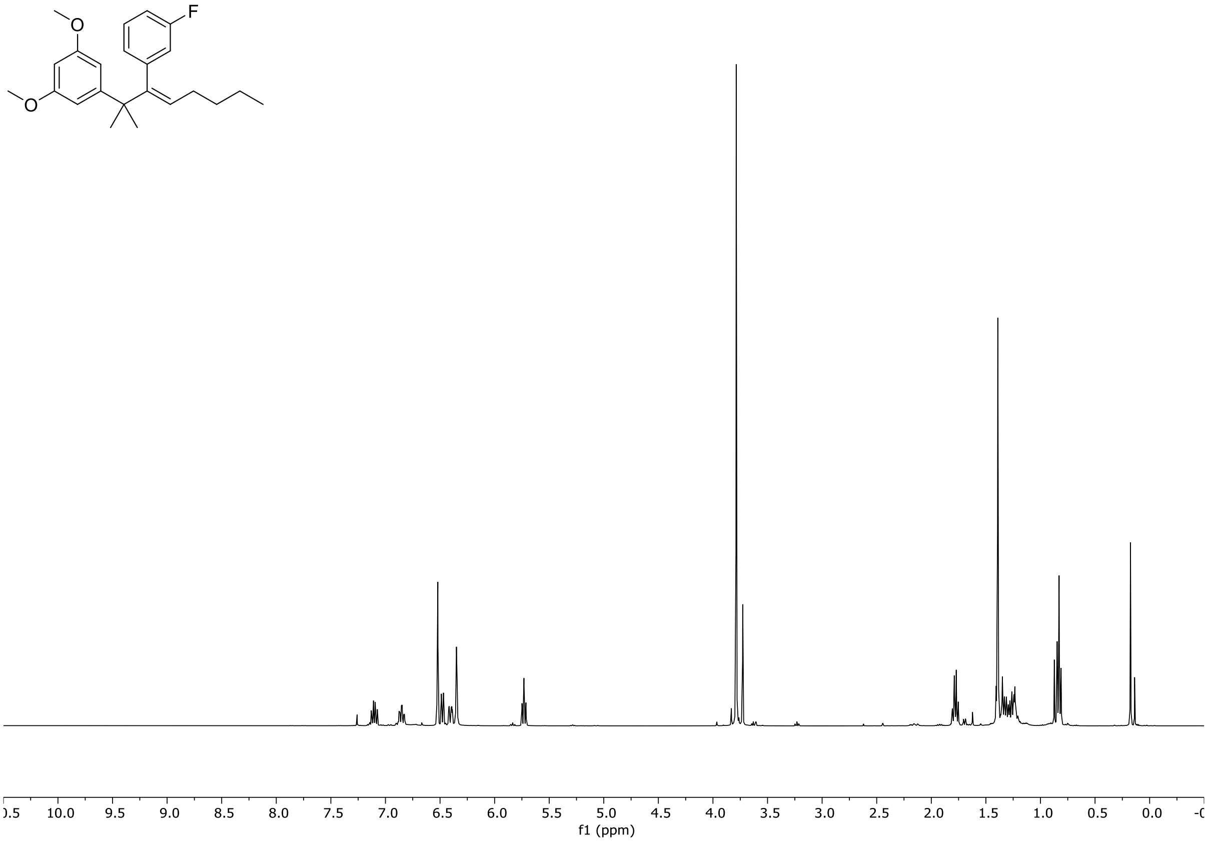

^{13}C NMR (101 MHz, $CDCl_3$) of **SI-16f**

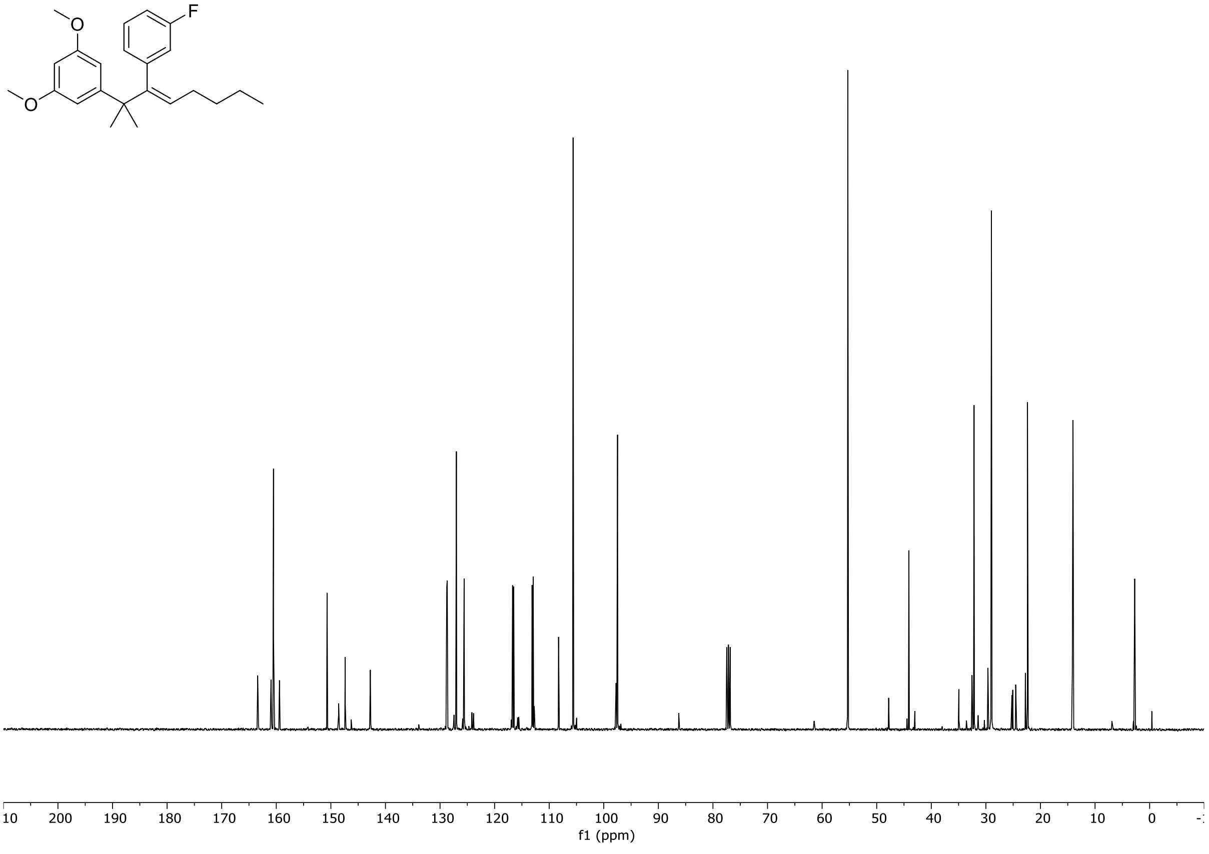

^{19}F NMR (377 MHz, $CDCl_3$) of **SI-16f**

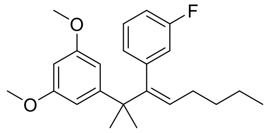

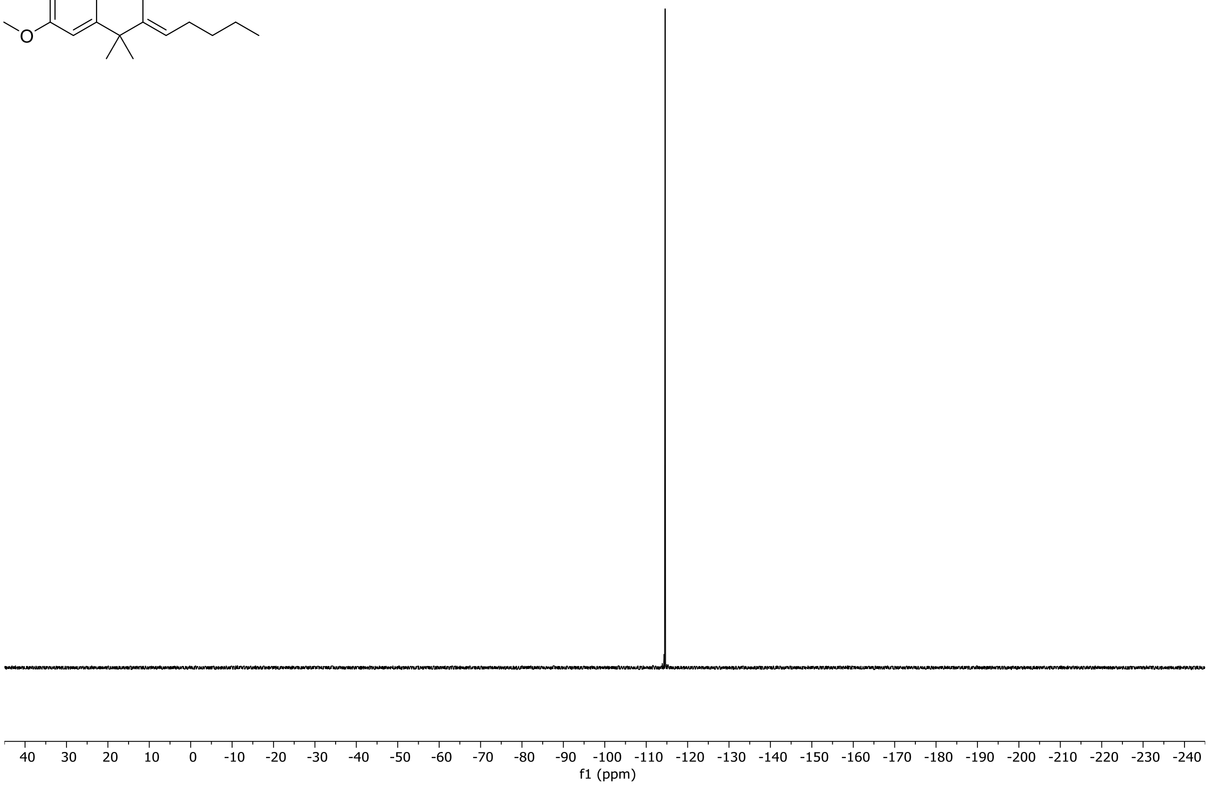

^{1}H NMR (400 MHz, $CDCl_3$) of **SI-17d**

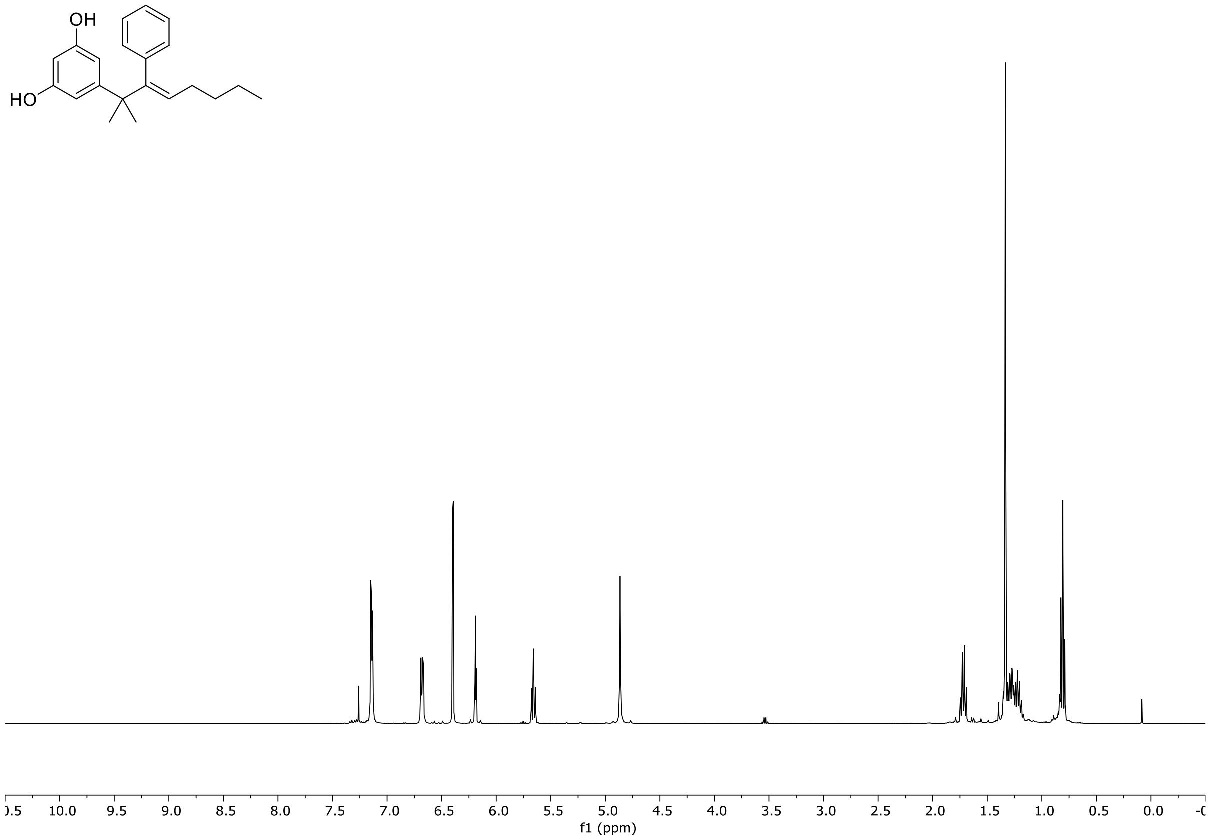

^{13}C NMR (101 MHz, $CDCl_3$) of **SI-17d**

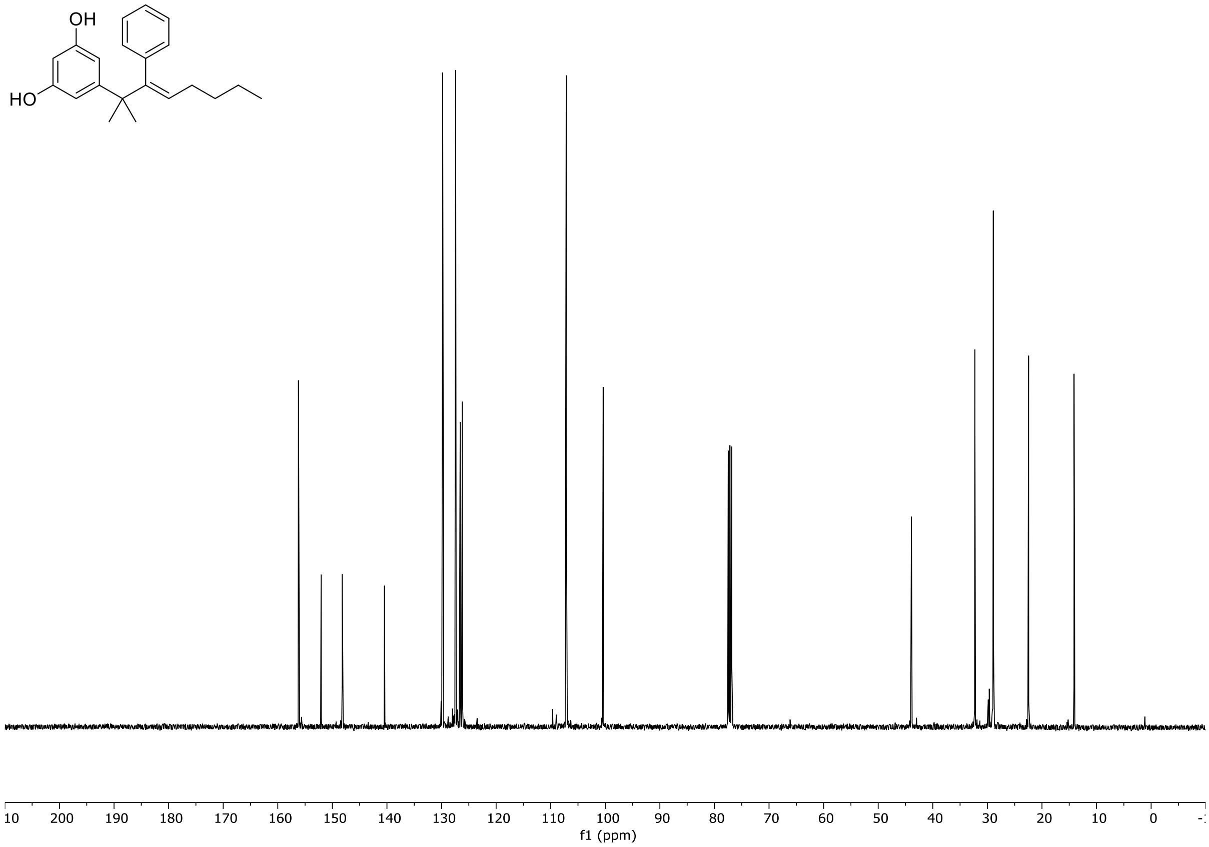

^{1}H NMR (400 MHz, $CDCl_3$) of **SI-17e**

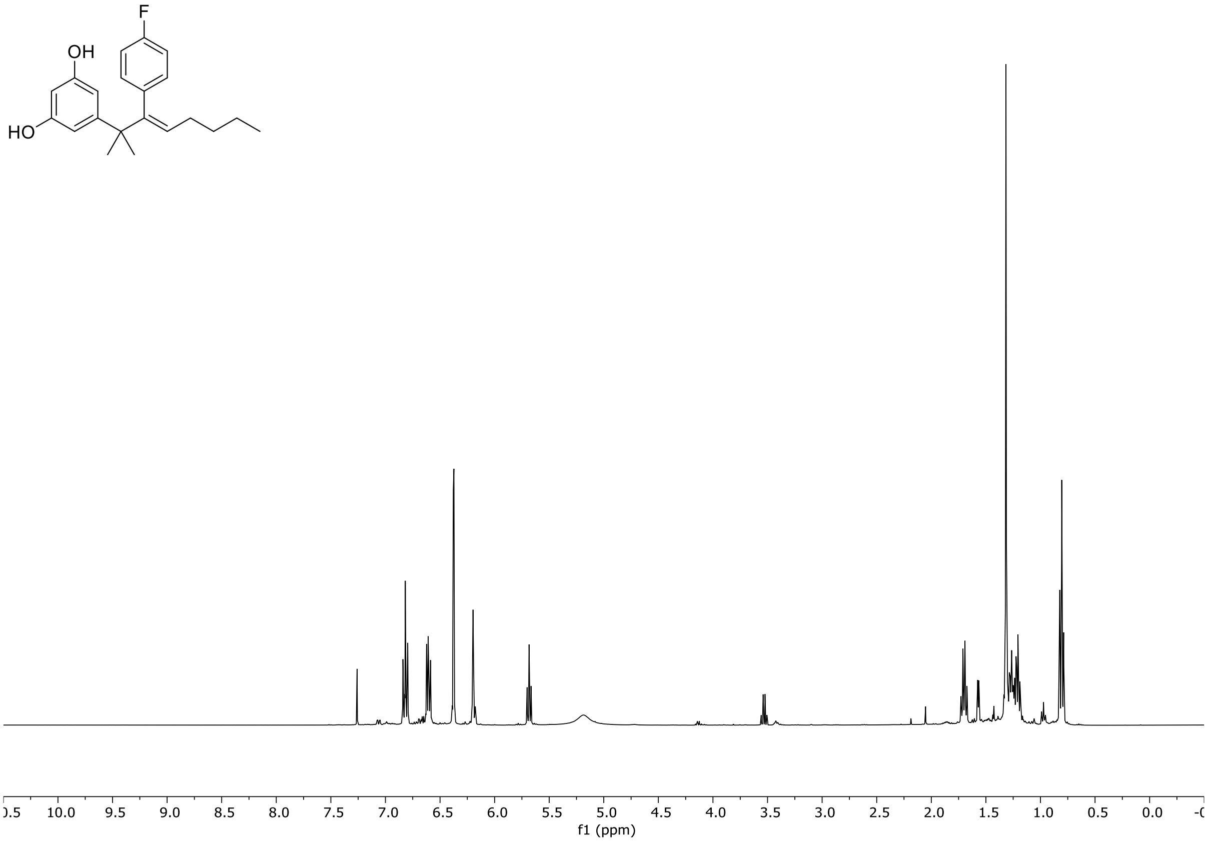

^{13}C NMR (101 MHz, $CDCl_3$) of **SI-17e**

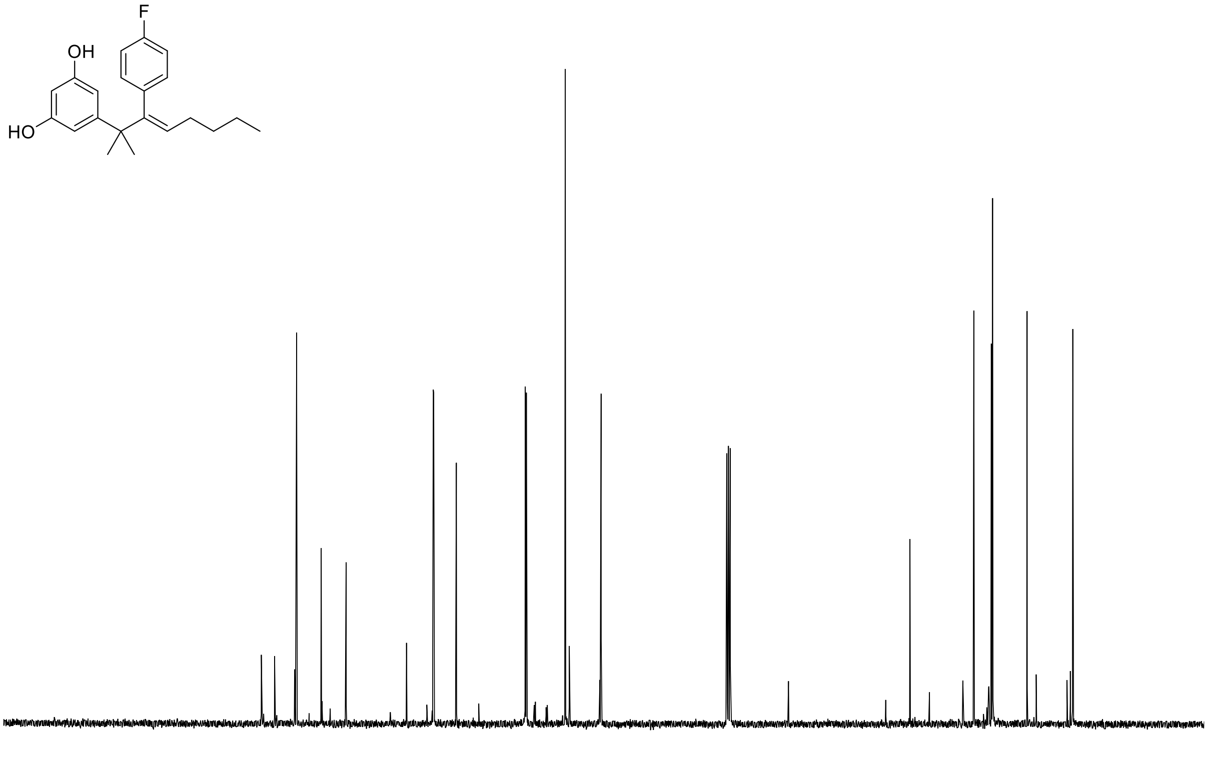

^{19}F NMR (377 MHz, $CDCl_3$) of **SI-17e**

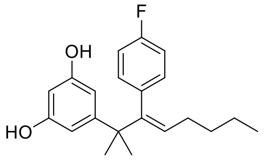

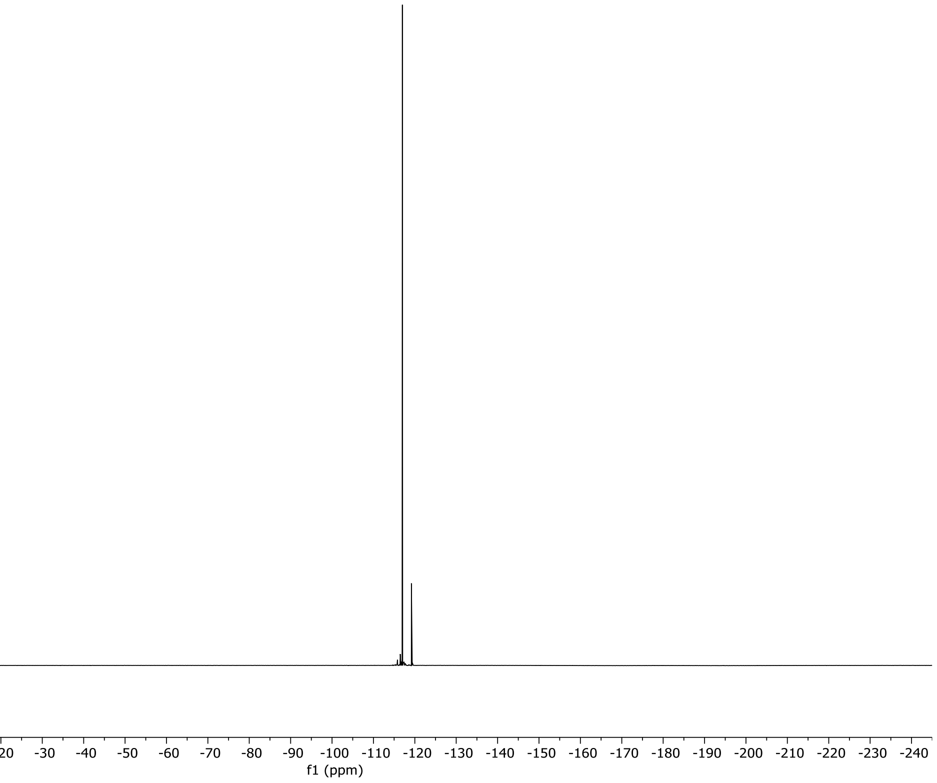

^{1}H NMR (400 MHz, CDCl$_3$) of **SI-17f**

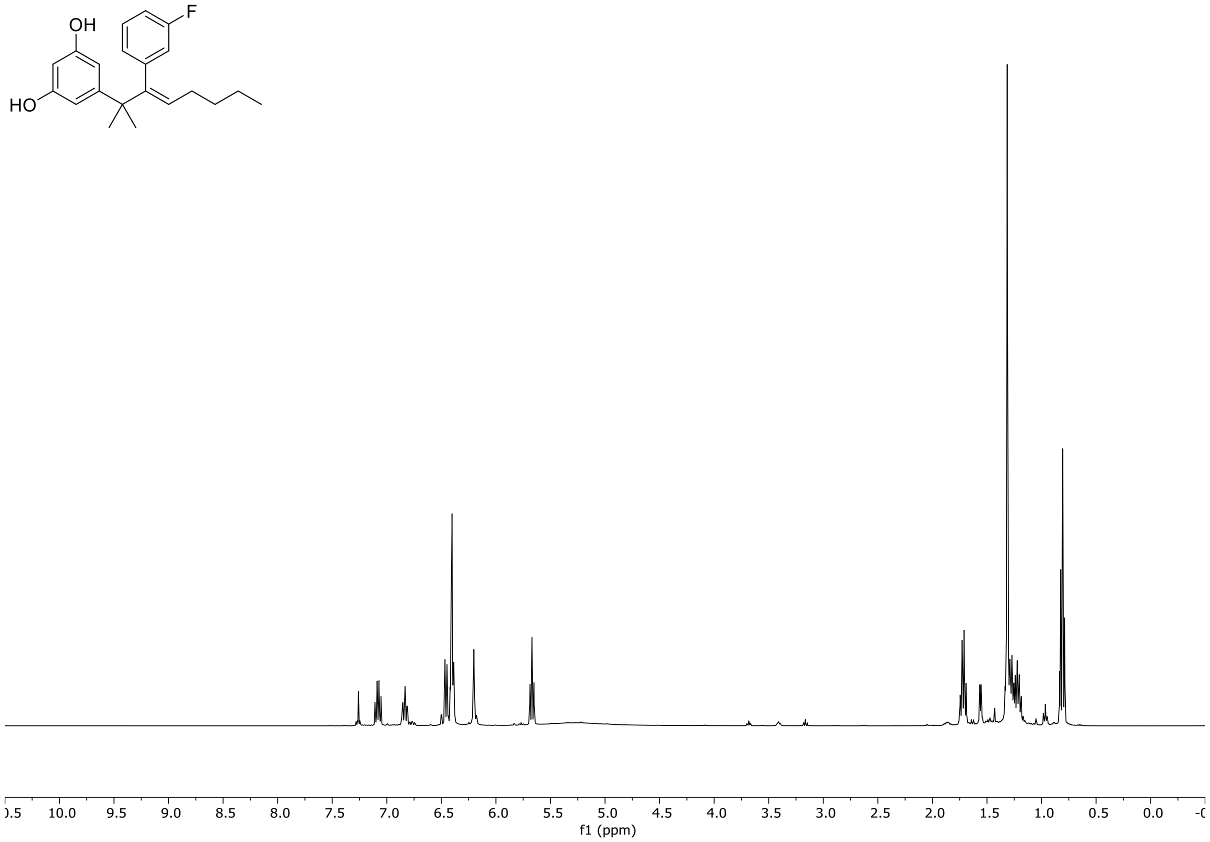

^{13}C NMR (101 MHz, CDCl$_3$) of **SI-17f**

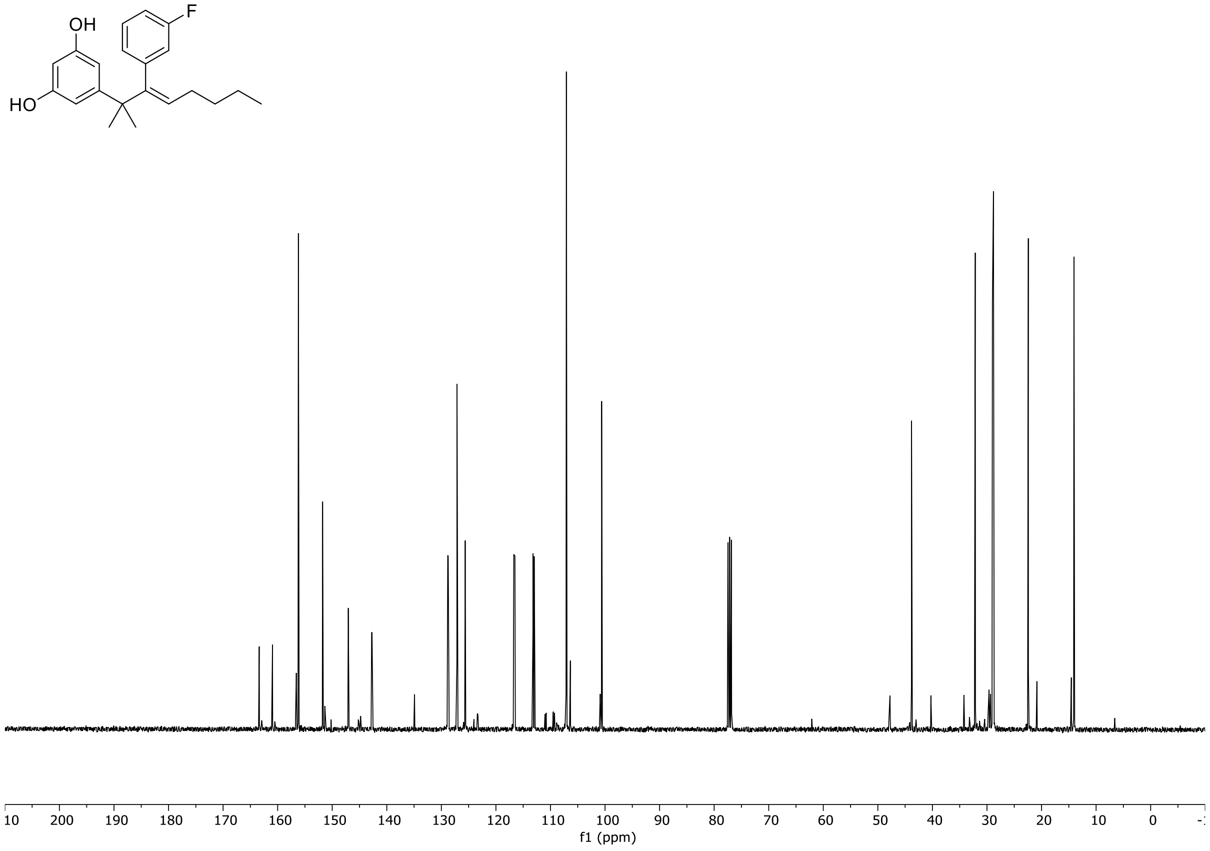

^{19}F NMR (377 MHz, $CDCl_3$) of **SI-17f**

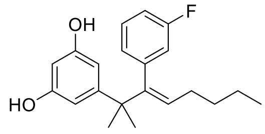

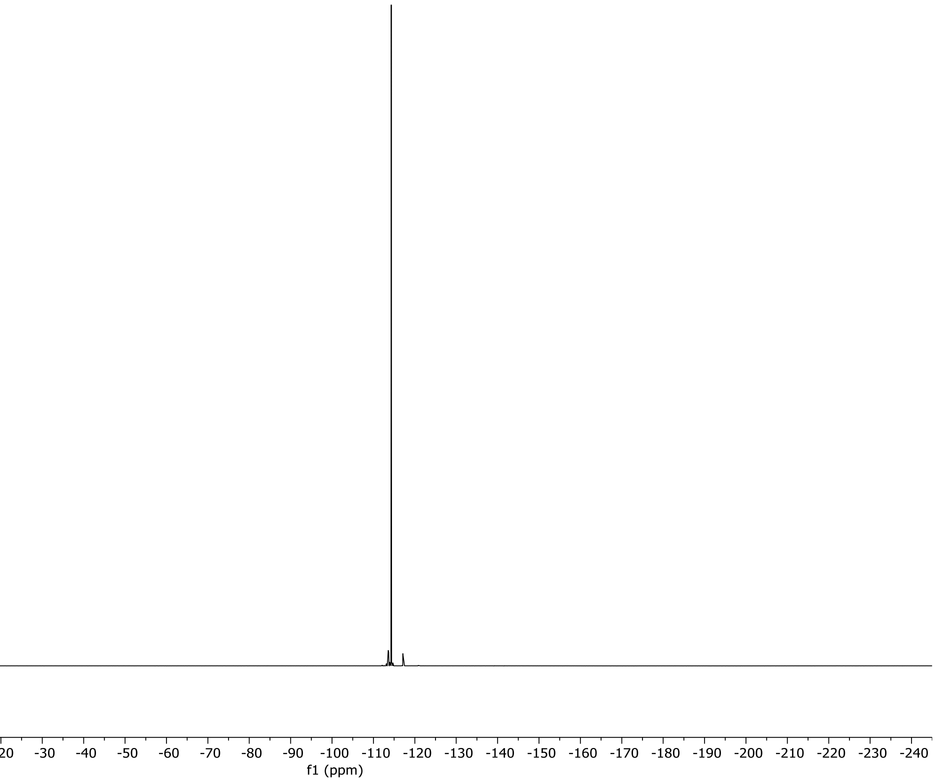

^{1}H NMR (400 MHz, CDCl$_3$) of (–)-(*S*)-**9d** & (+)-(*R*)-**9d**

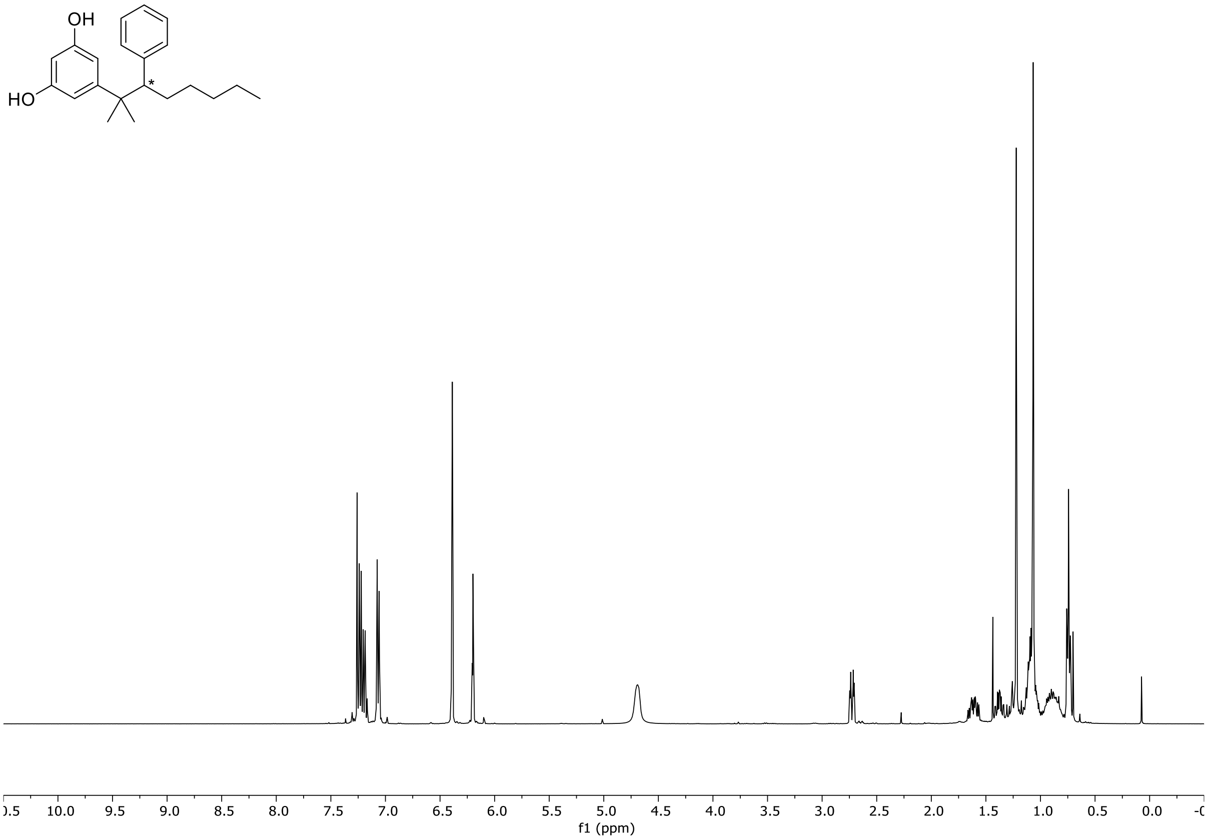

^{13}C NMR (101 MHz, CDCl$_3$) of (–)-(*S*)-**9d** & (+)-(*R*)-**9d**

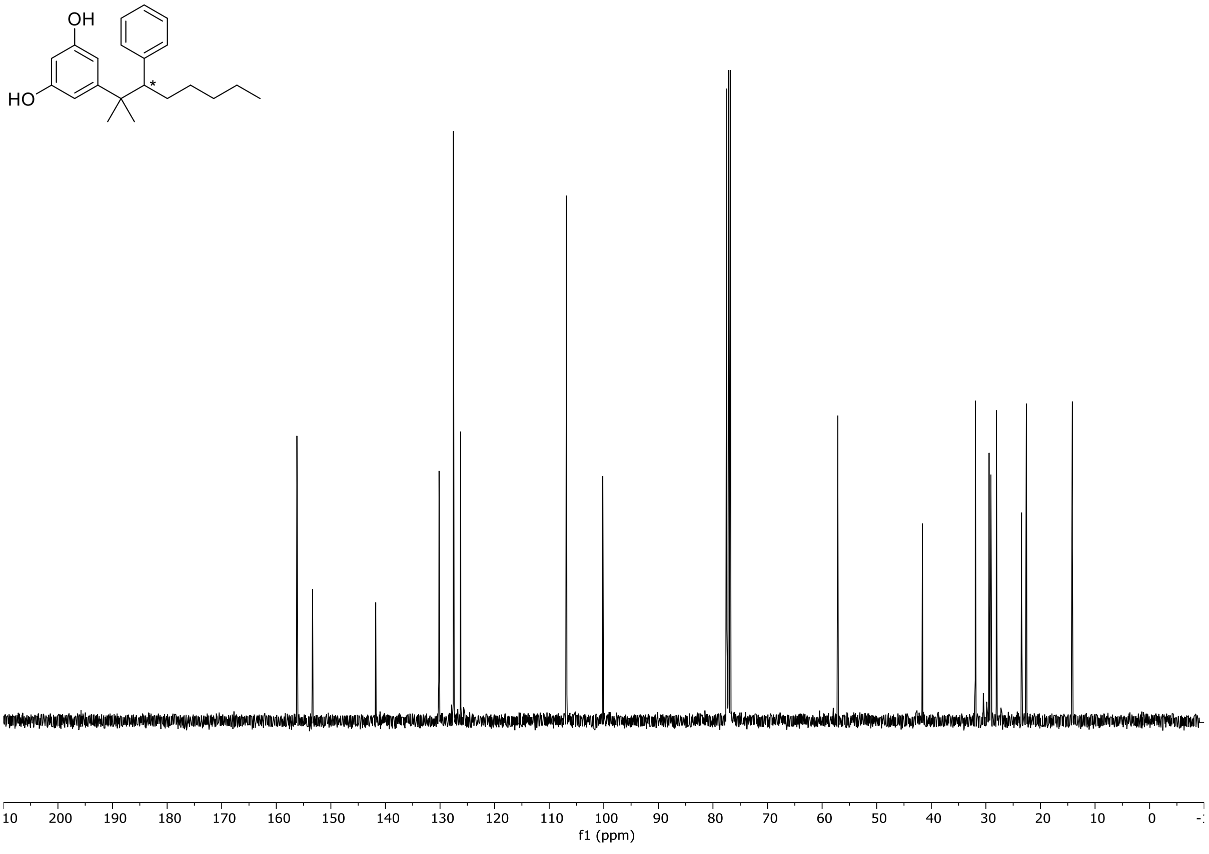

^{1}H NMR (500 MHz, $CDCl_3$) indepenent synthesis of (*S*)-**SI-18**/(A)-**9** = (–)-(*S*)-**19**

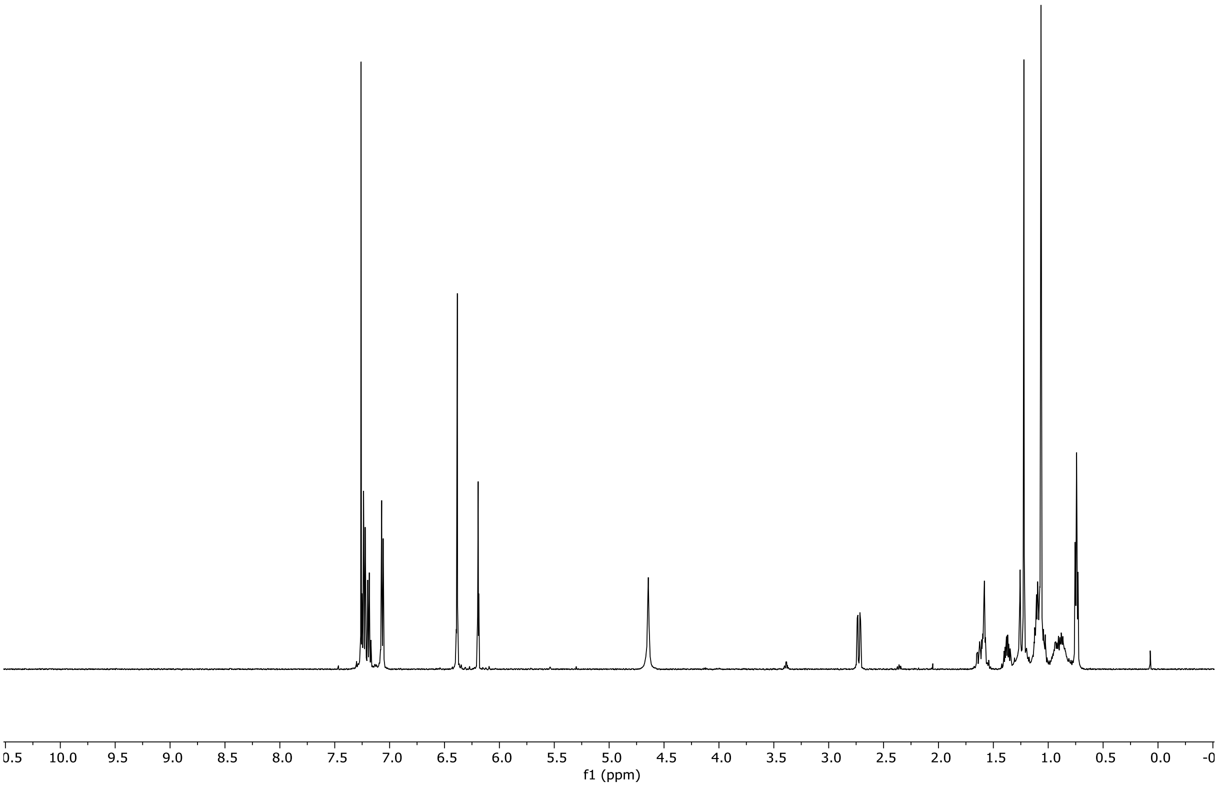

^{1}H NMR (400 MHz, $CDCl_3$) of (–)-(*S*)-**9e** & (+)-(*R*)-**9e**

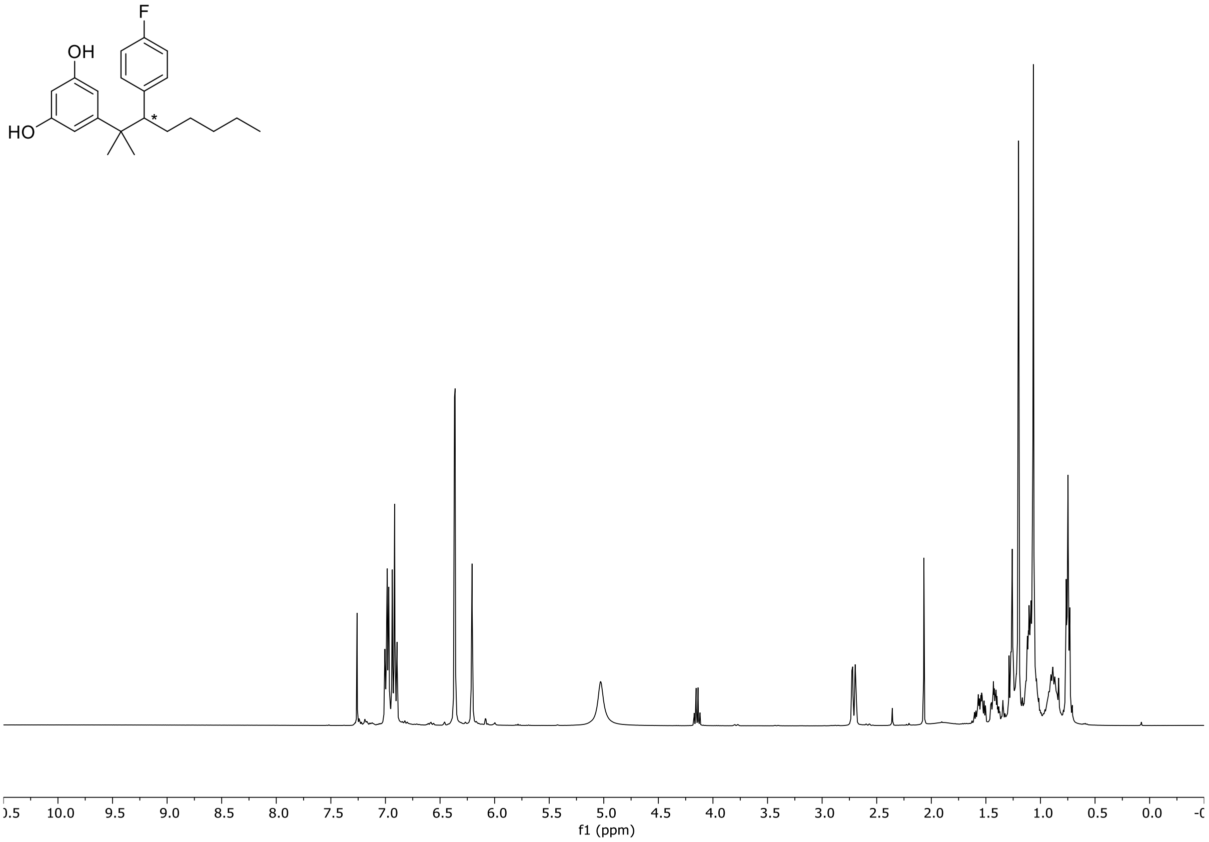

^{13}C NMR (101 MHz, $CDCl_3$) of (–)-(*S*)-**9e** & (+)-(*R*)-**9e**

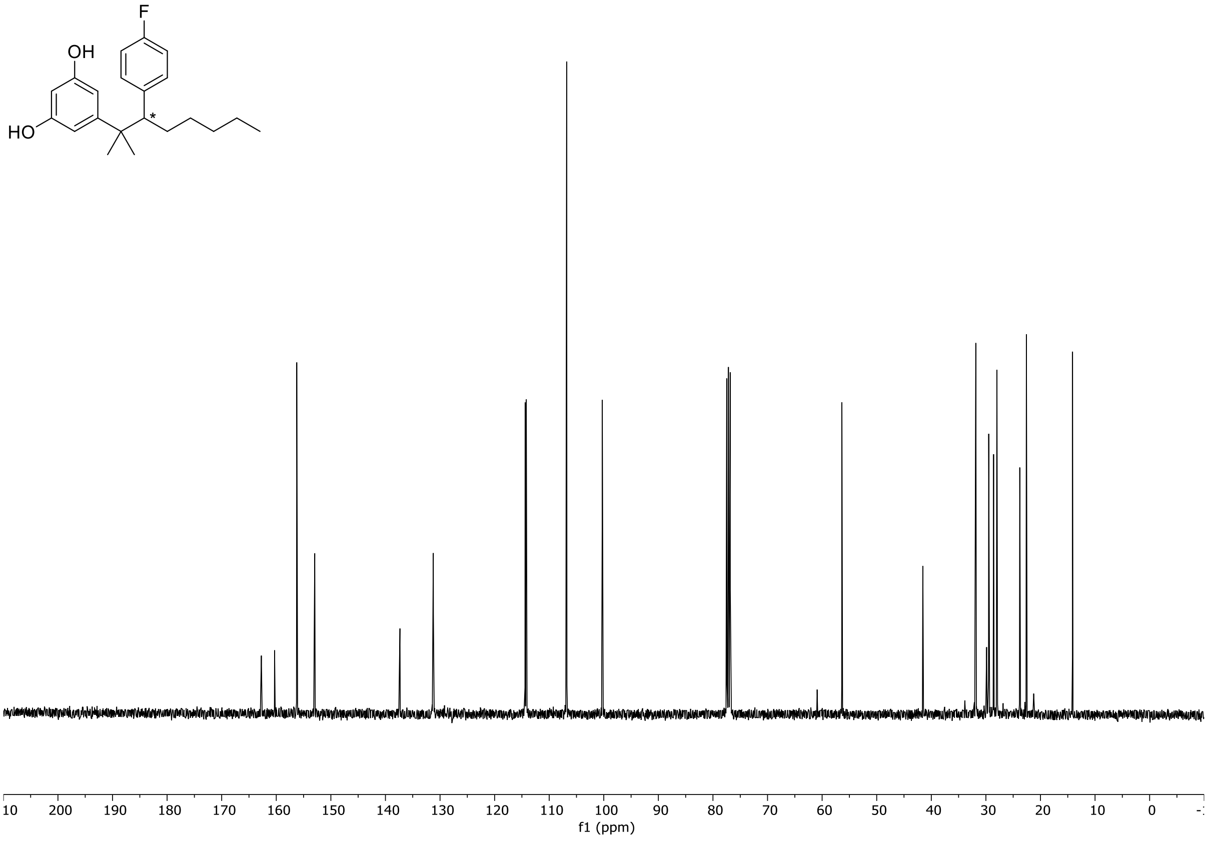

^{19}F NMR (377 MHz, $CDCl_3$) of (–)-(*S*)-**9e** & (+)-(*R*)-**9e**

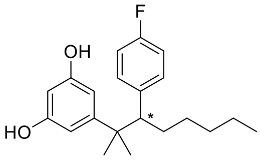

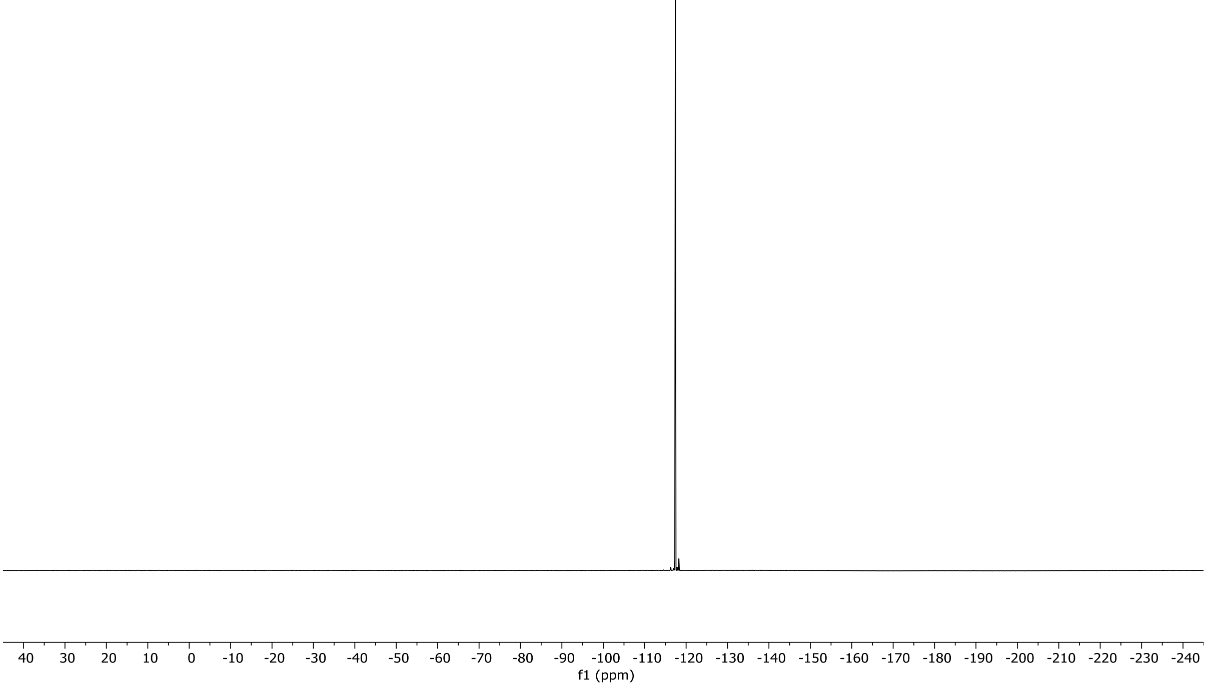

^{1}H NMR (400 MHz, $CDCl_3$) of (–)-(*S*)-**9f** & (+)-(*R*)-**9f**

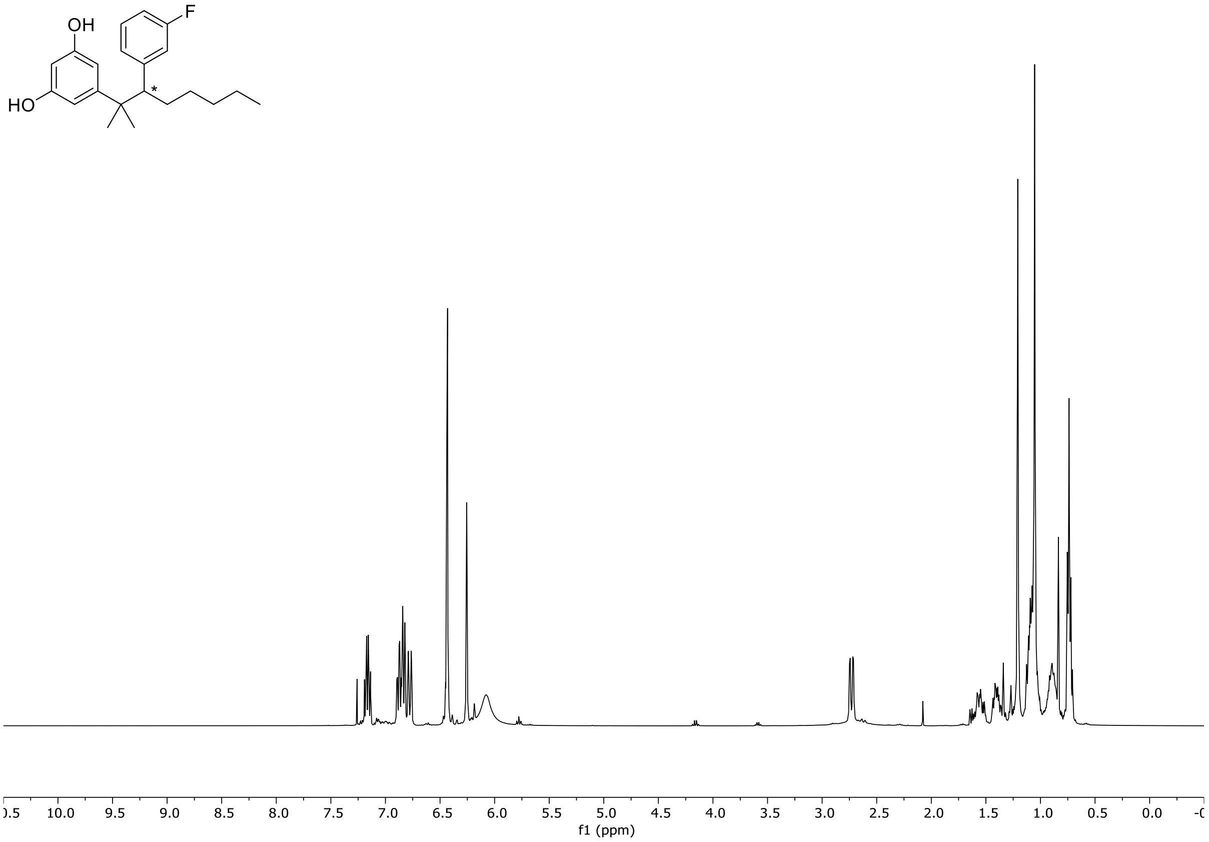

^{13}C NMR (101 MHz, $CDCl_3$) of (–)-(*S*)-**9f** & (+)-(*R*)-**9f**

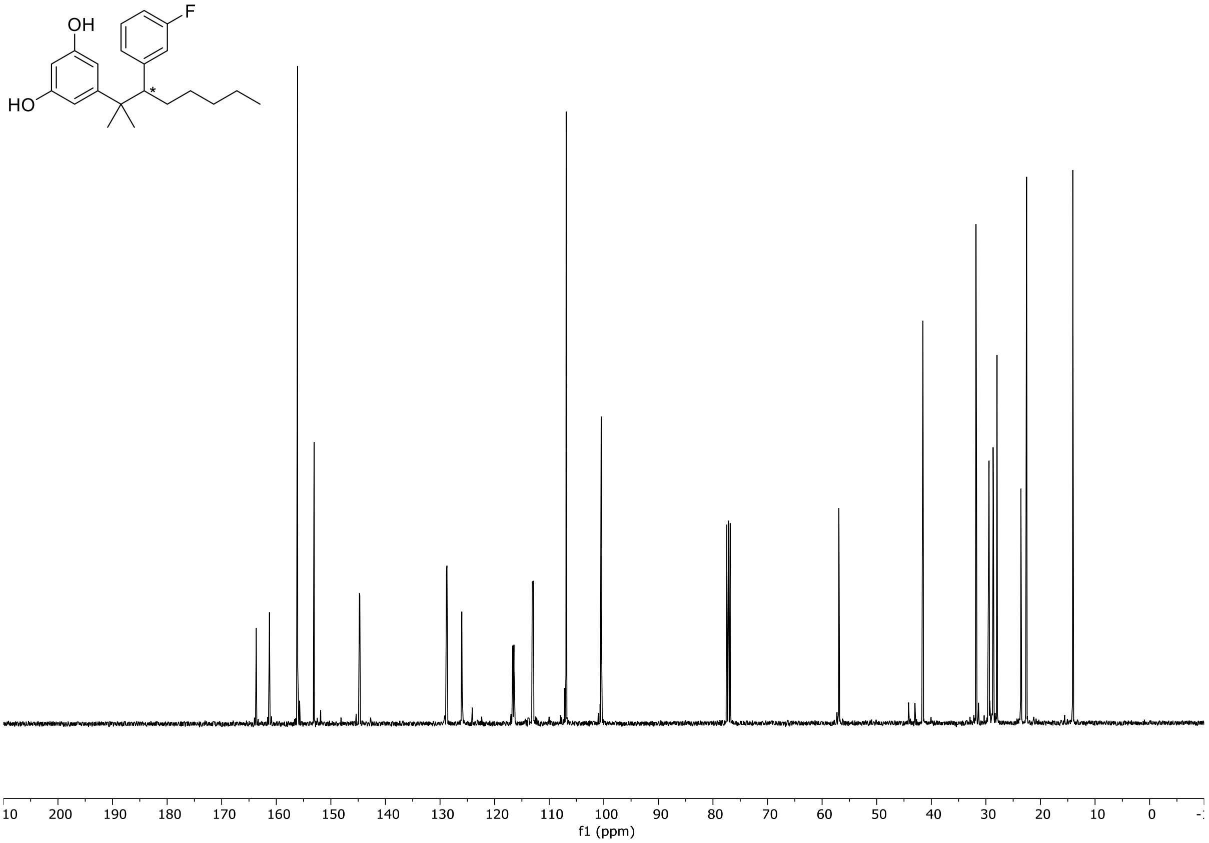

^{19}F NMR (377 MHz, $CDCl_3$) of (–)-(*S*)-**9f** & (+)-(*R*)-**9f**

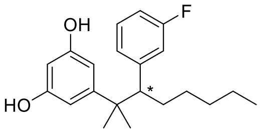

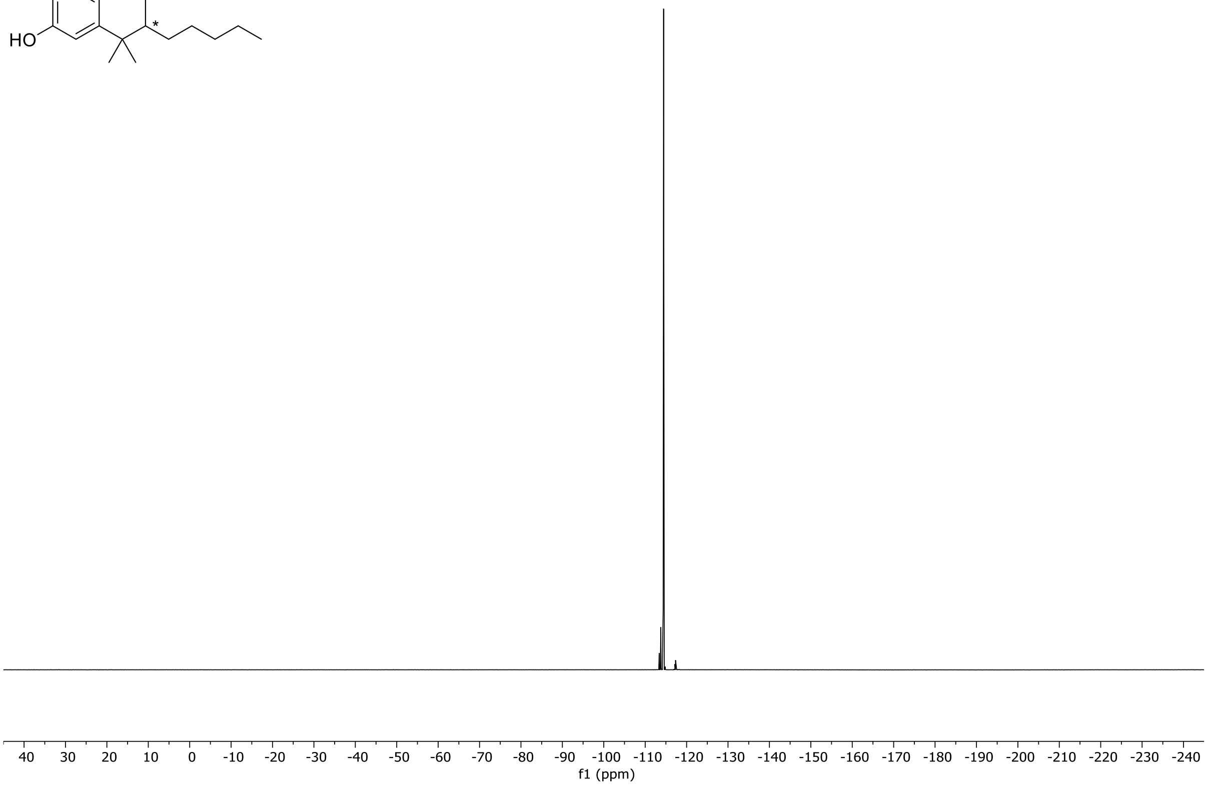

^{1}H NMR (400 MHz, $CDCl_3$) of (*S*/*R*)-**SI-20a**

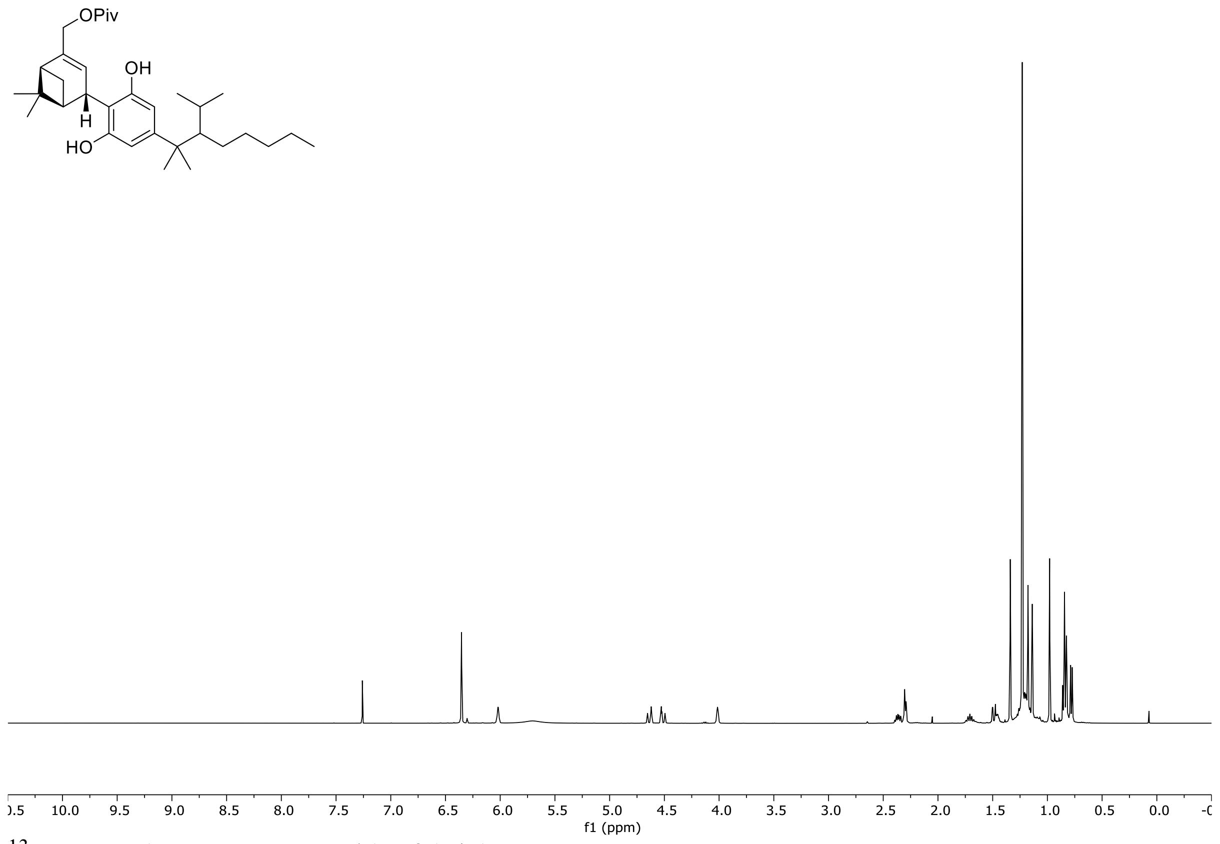

^{13}C NMR (101 MHz, $CDCl_3$) of (*S*/*R*)-**SI-20a**

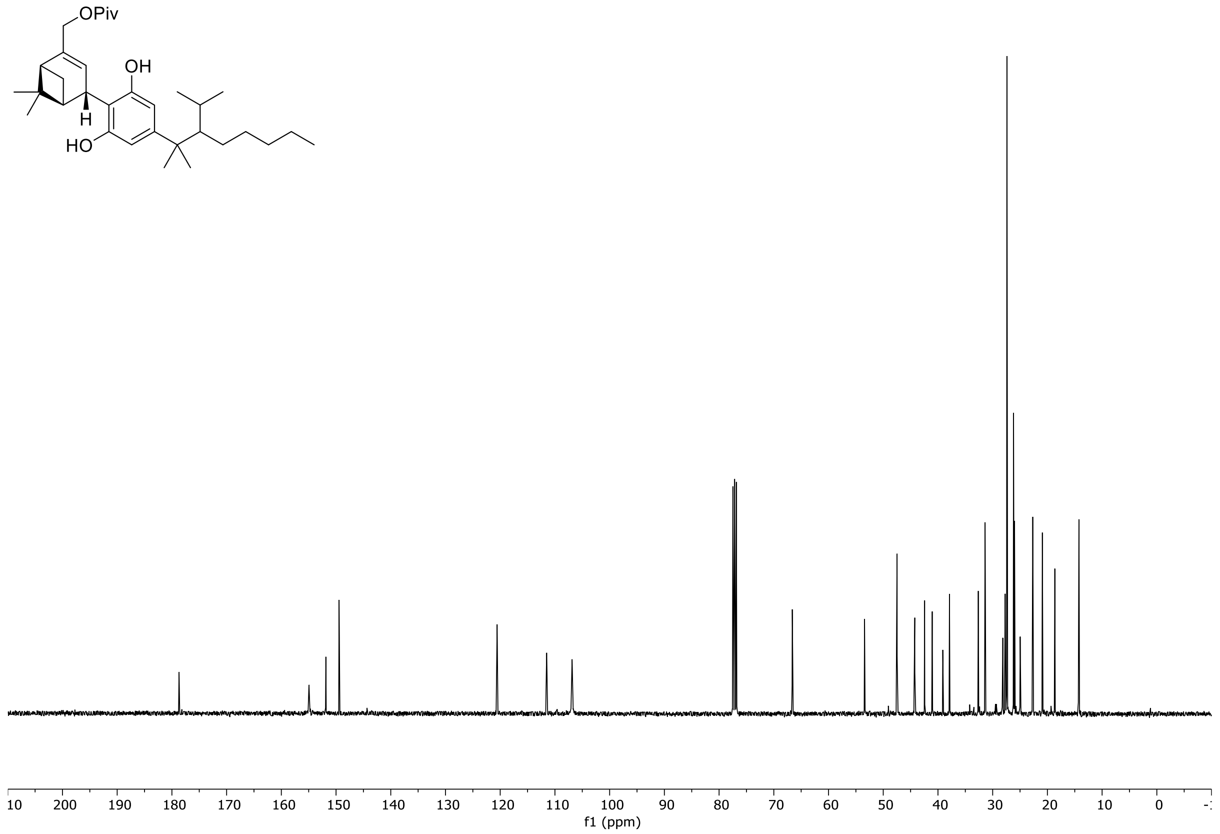

^{1}H NMR (400 MHz, $CDCl_3$) of (*S*/*R*)-**10a**

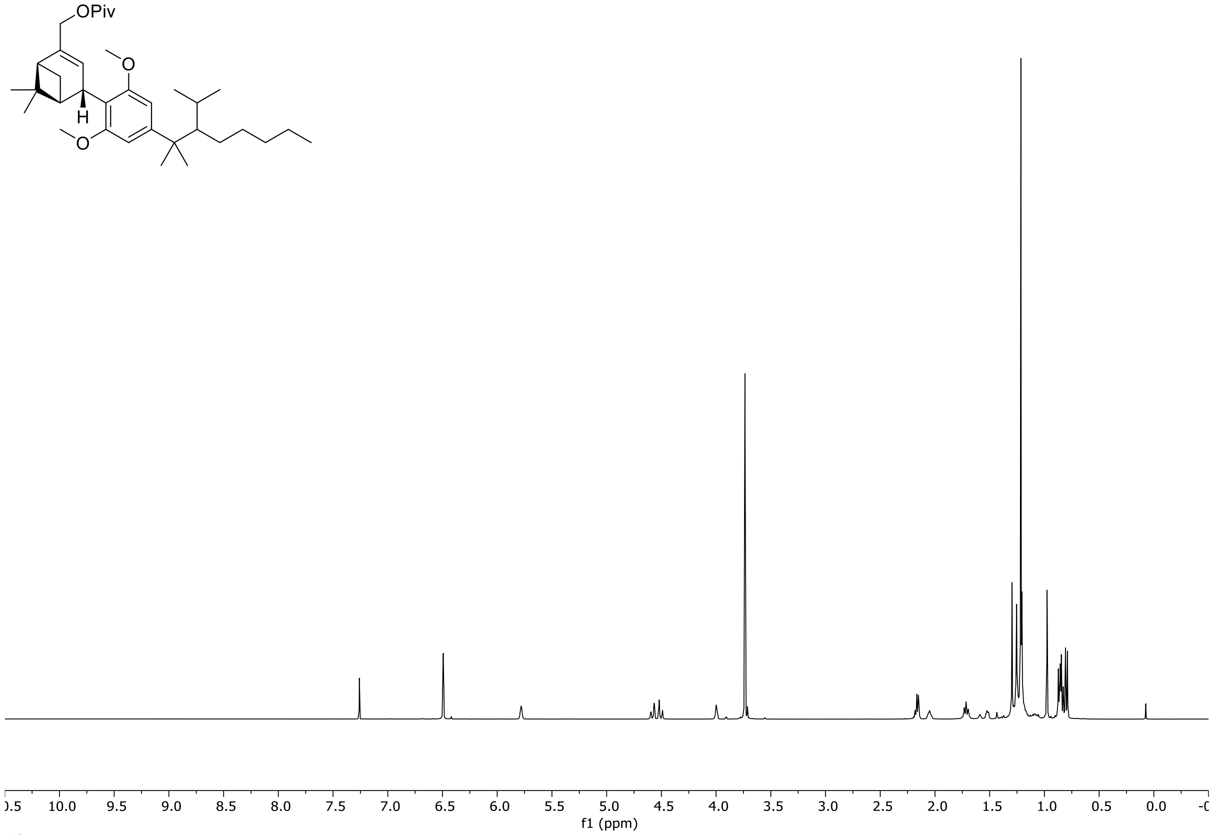

^{13}C NMR (101 MHz, $CDCl_3$) of (*S*/*R*)-**10a**

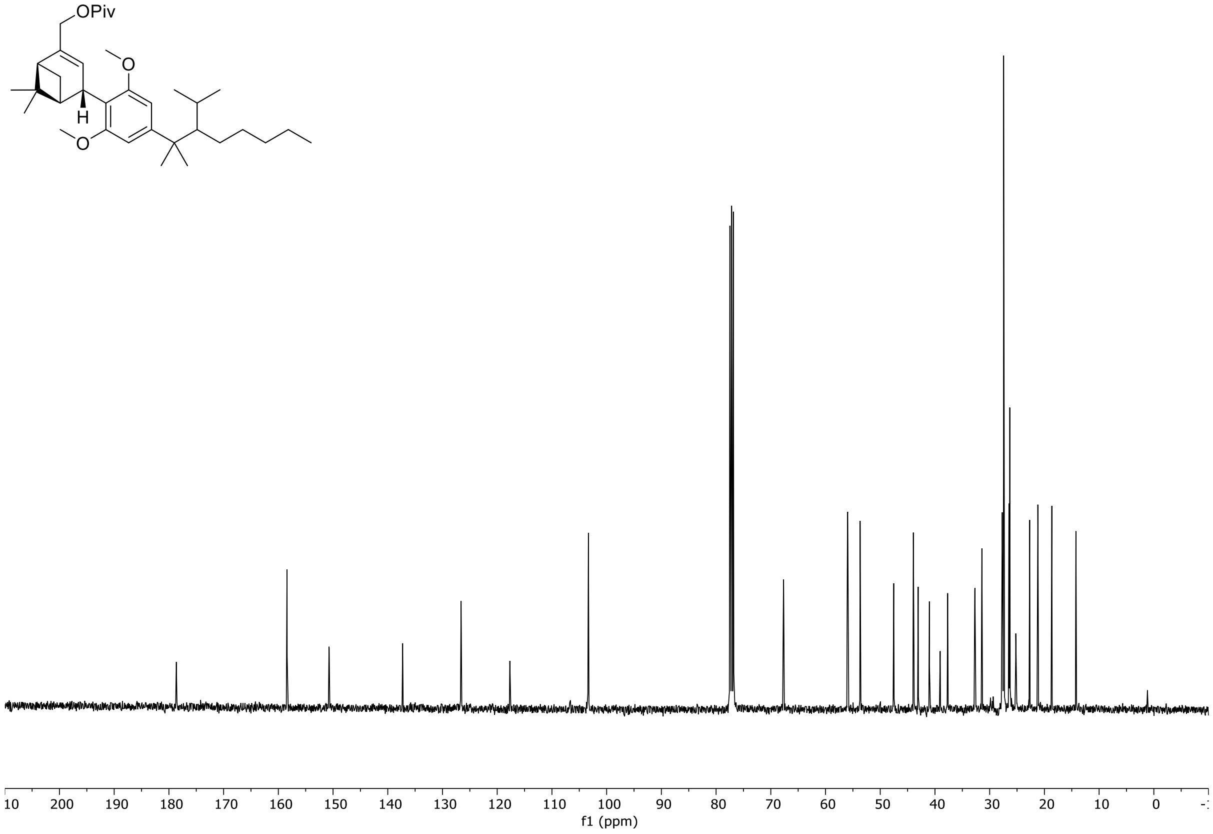

^{1}H NMR (400 MHz, $CDCl_3$) of (*S*/*R*)-**6a**

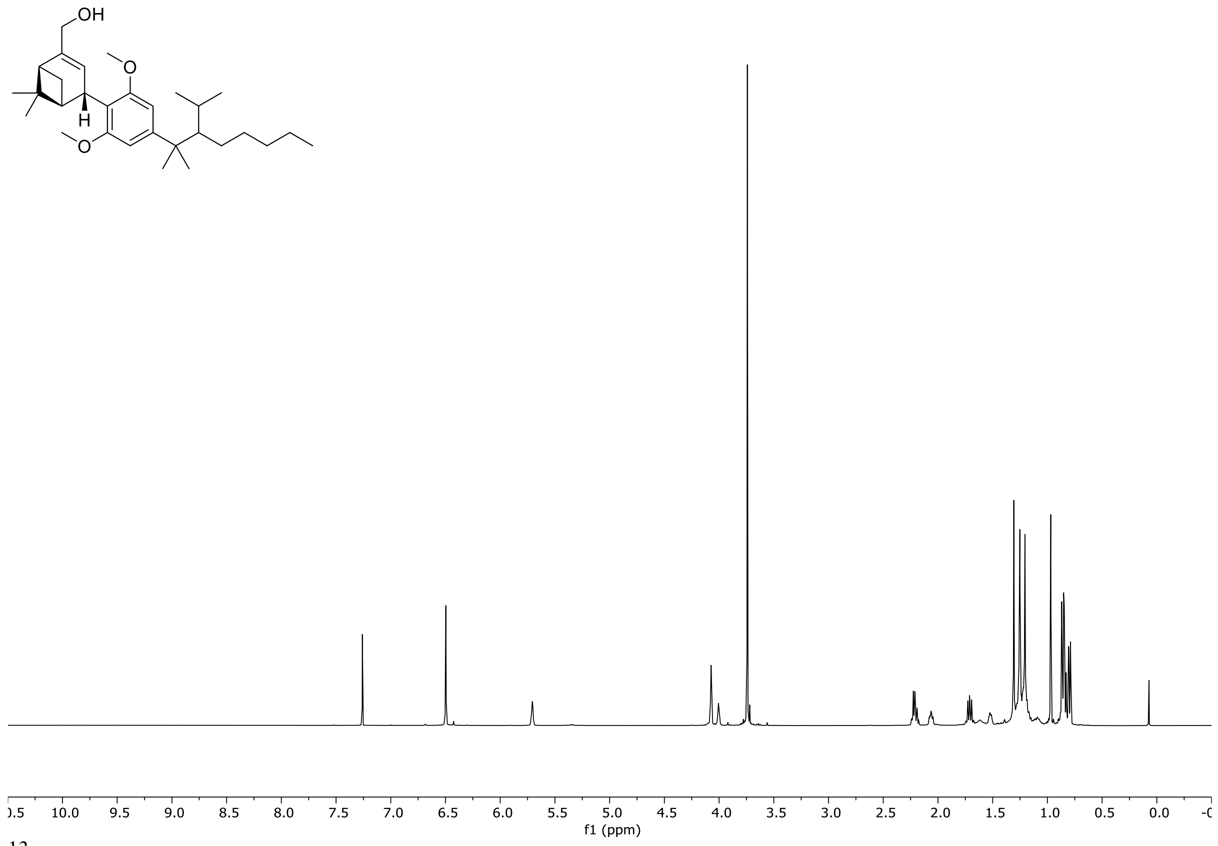

^{13}C NMR (101 MHz, $CDCl_3$) of (*S*/*R*)-**6a**

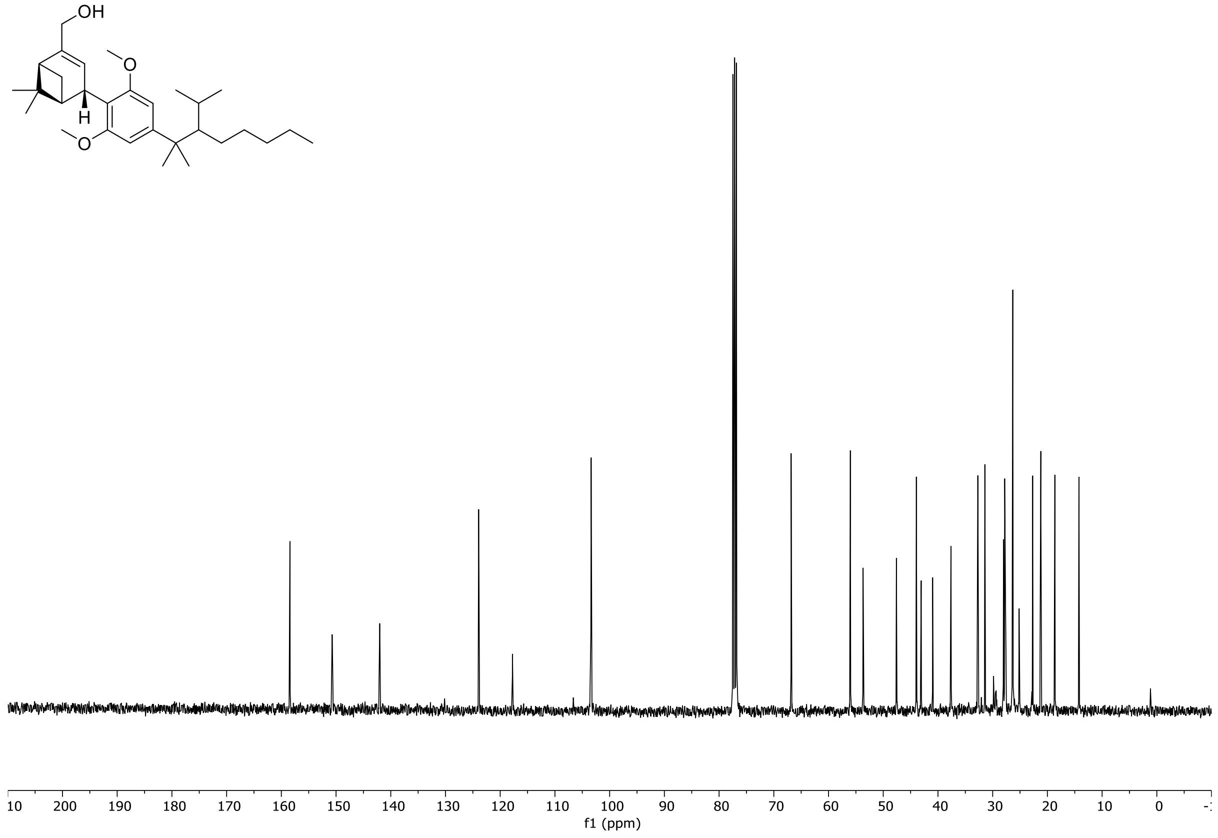

^{1}H NMR (400 MHz, $CDCl_3$) of (*S*/*R*)-**SI-20b**

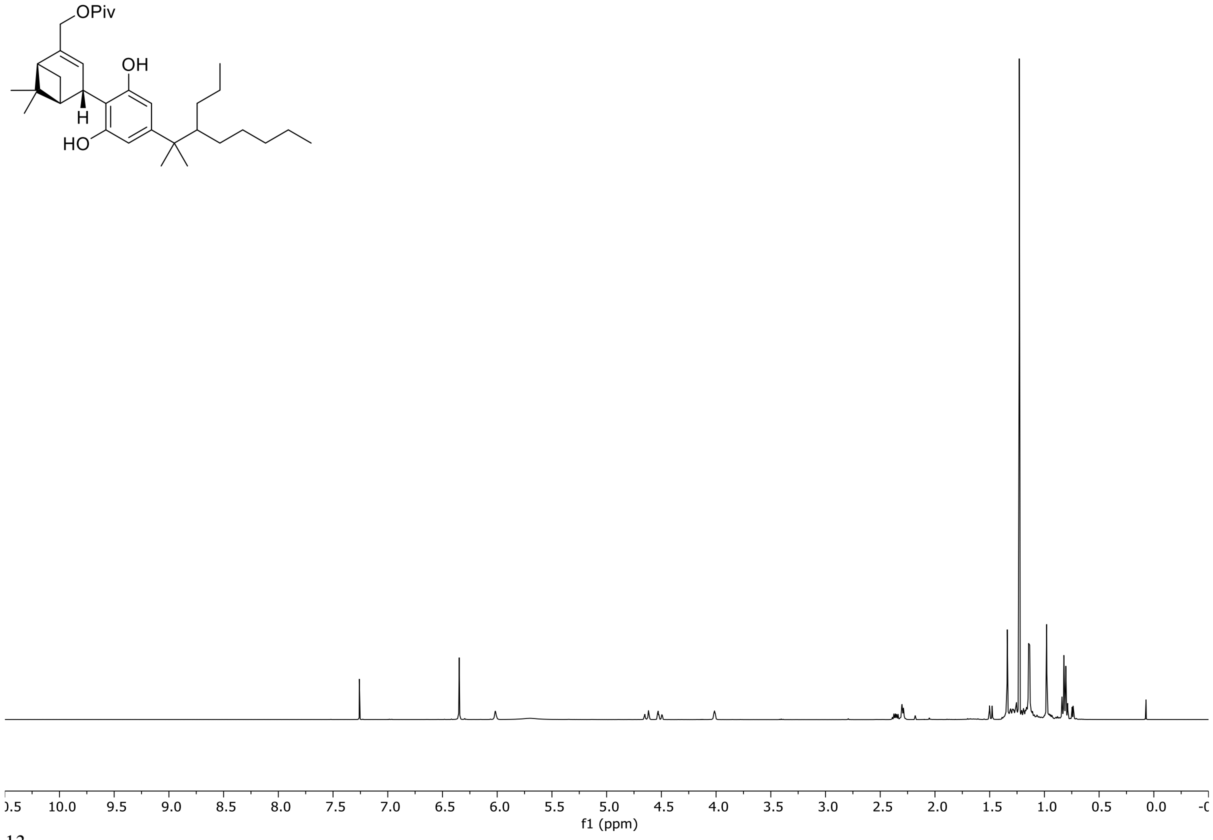

^{13}C NMR (101 MHz, $CDCl_3$) of (*S*/*R*)-**SI-20b**

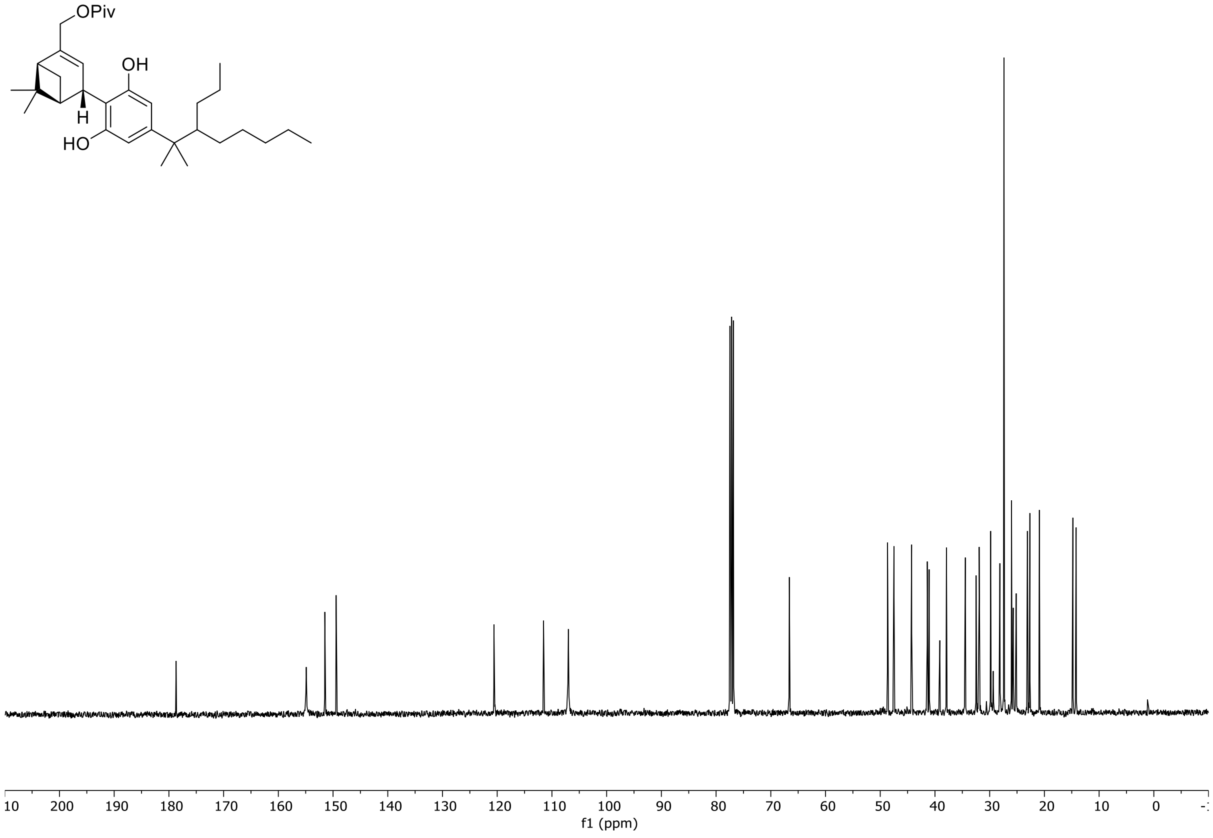

^{1}H NMR (400 MHz, $CDCl_3$) of (*S*/*R*)-**10b**

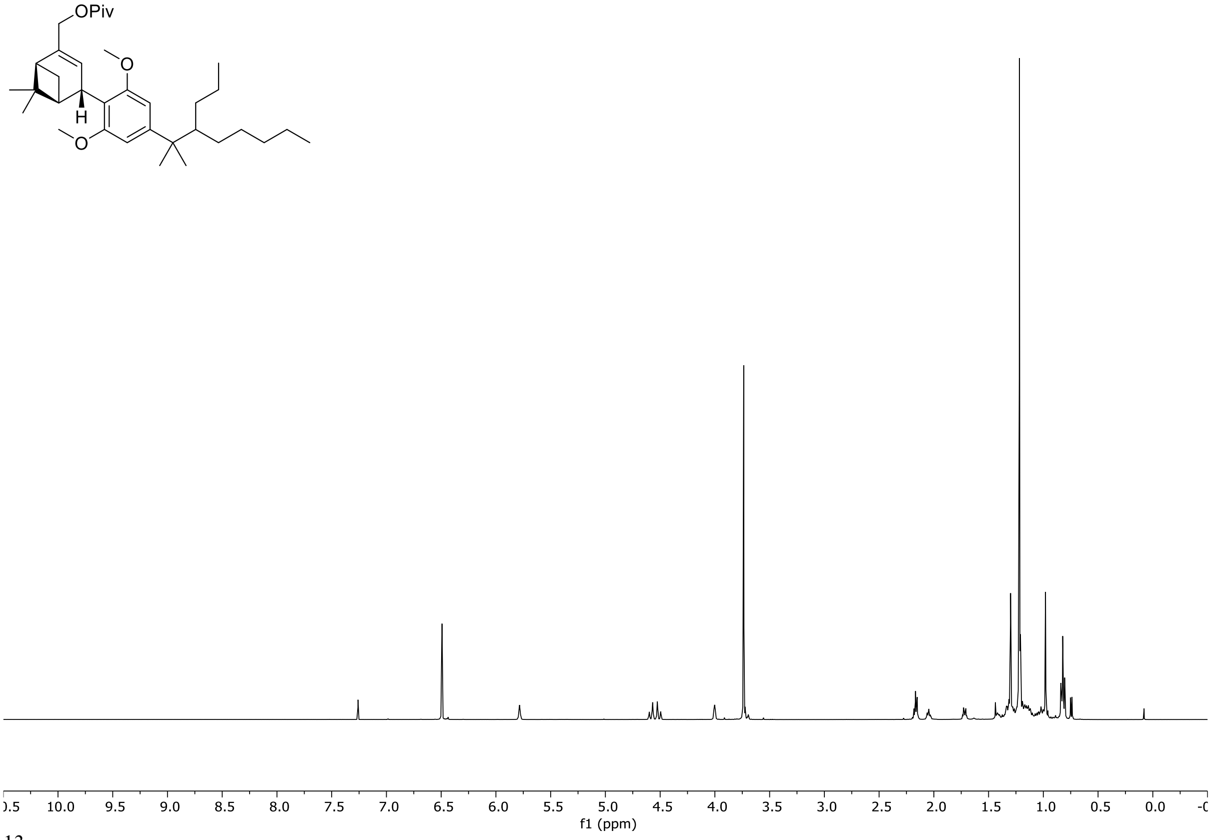

^{13}C NMR (101 MHz, $CDCl_3$) of (*S*/*R*)-**10b**

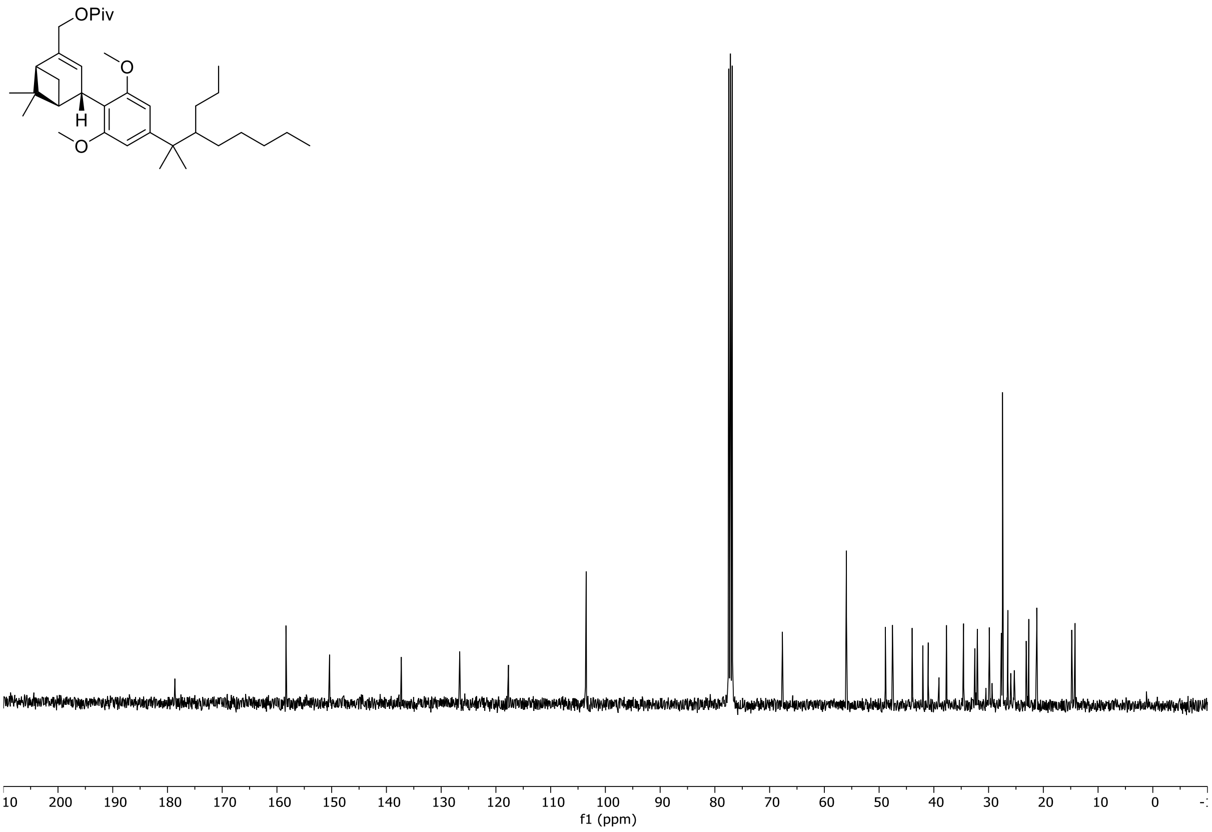

^{1}H NMR (400 MHz, $CDCl_3$) of (*S*/*R*)-**6b**

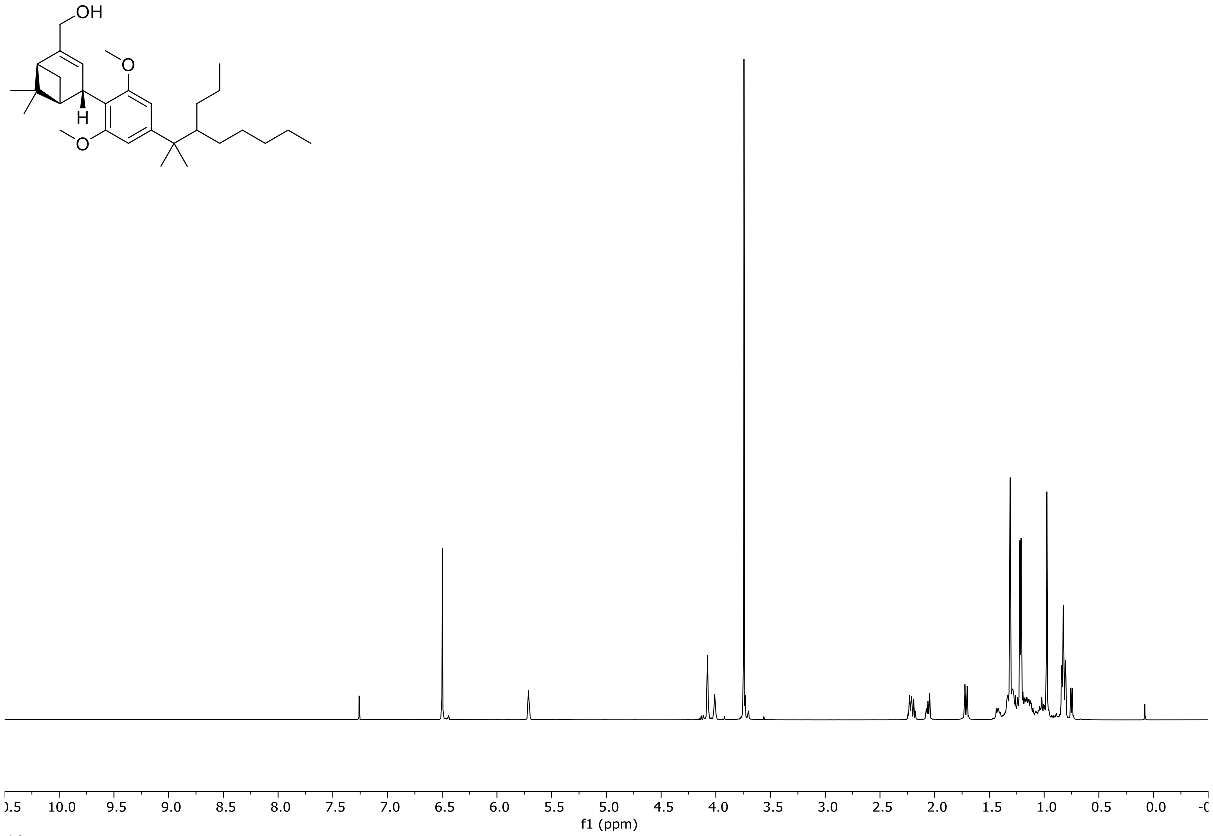

^{13}C NMR (101 MHz, $CDCl_3$) of (*S*/*R*)-**6b**

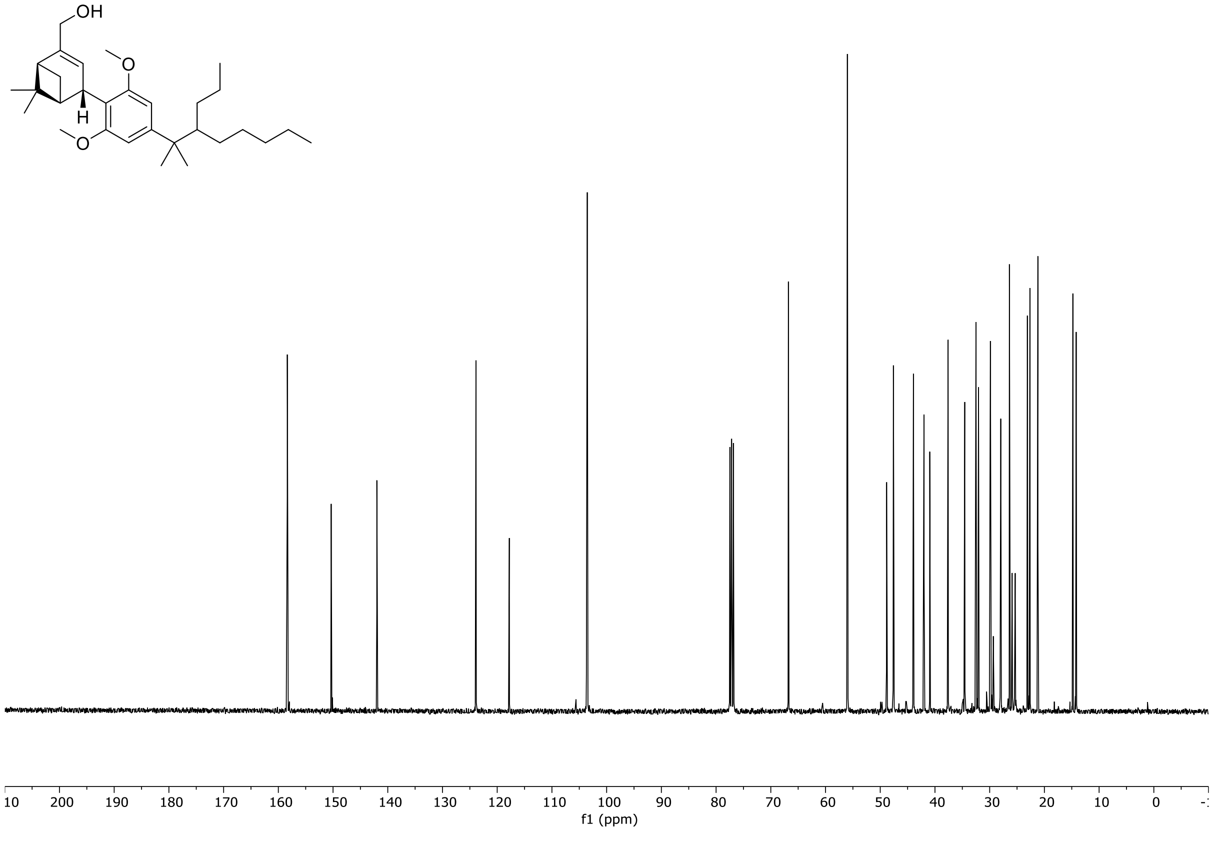

^{1}H NMR (400 MHz, $CDCl_3$) of (*S*/*R*)-**SI-20c**

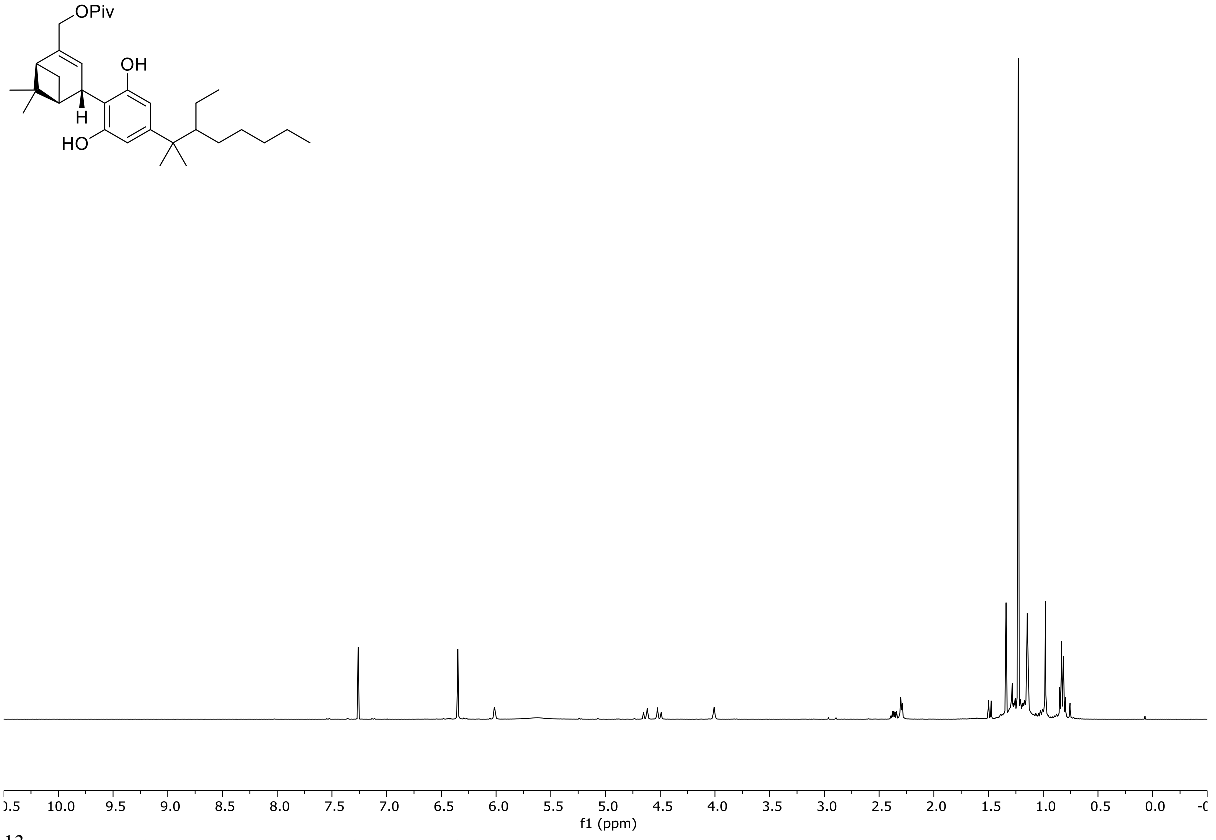

^{13}C NMR (101 MHz, $CDCl_3$) of (*S*/*R*)-**SI-20c**

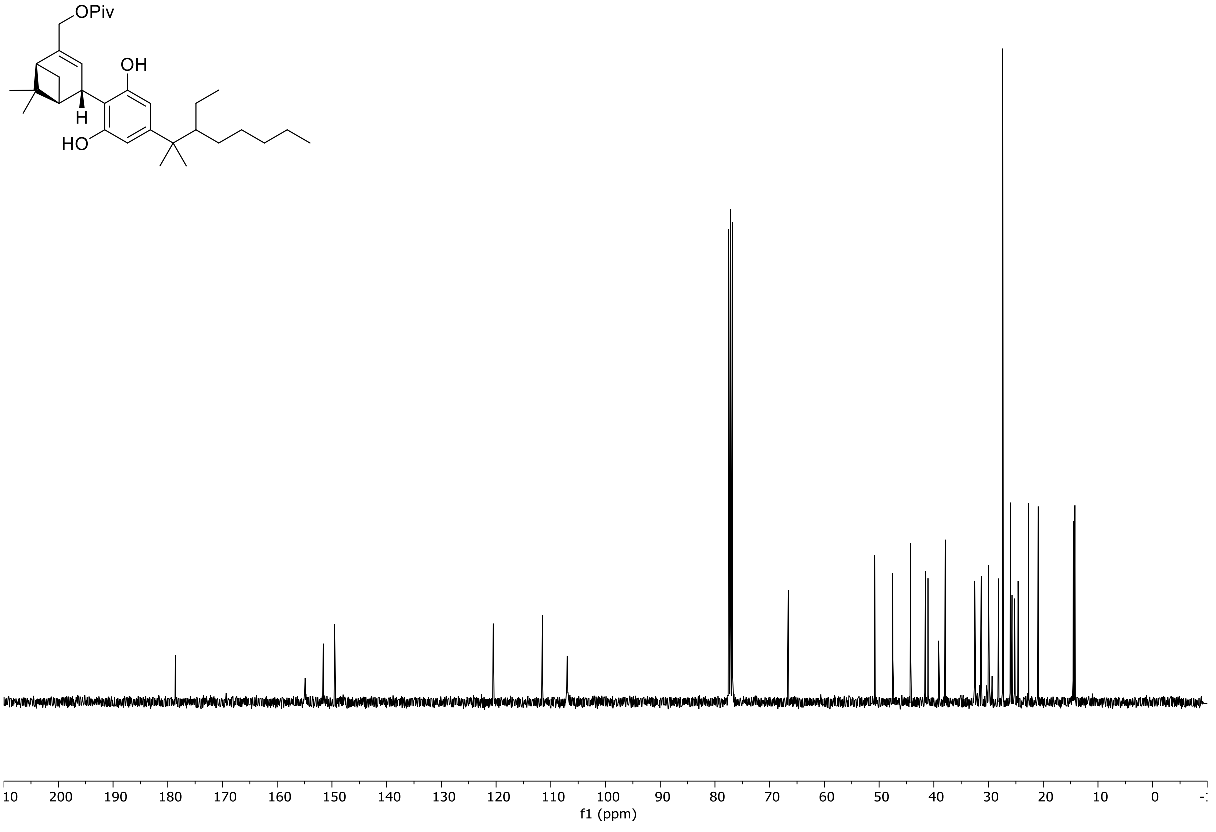

^{1}H NMR (400 MHz, $CDCl_3$) of (*S*/*R*)-**10c**

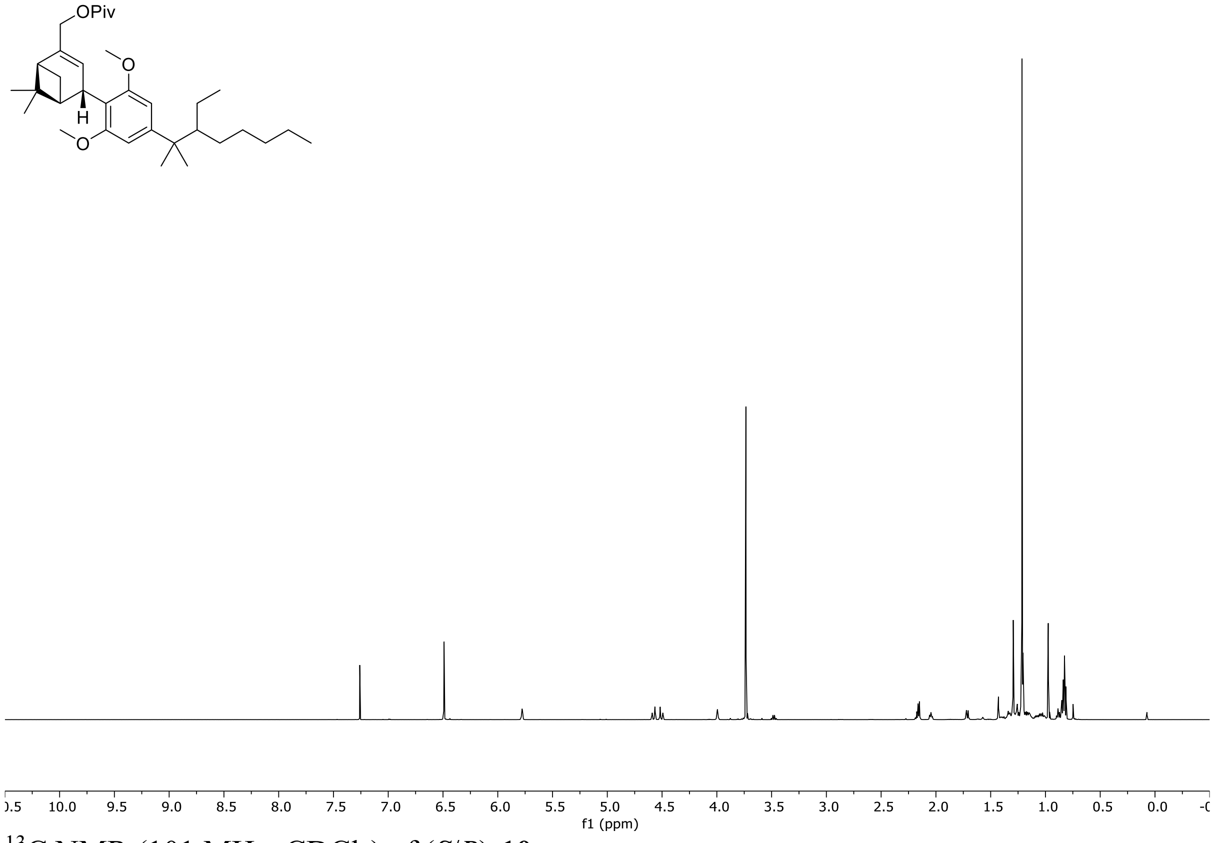

^{13}C NMR (101 MHz, $CDCl_3$) of (*S*/*R*)-**10c**

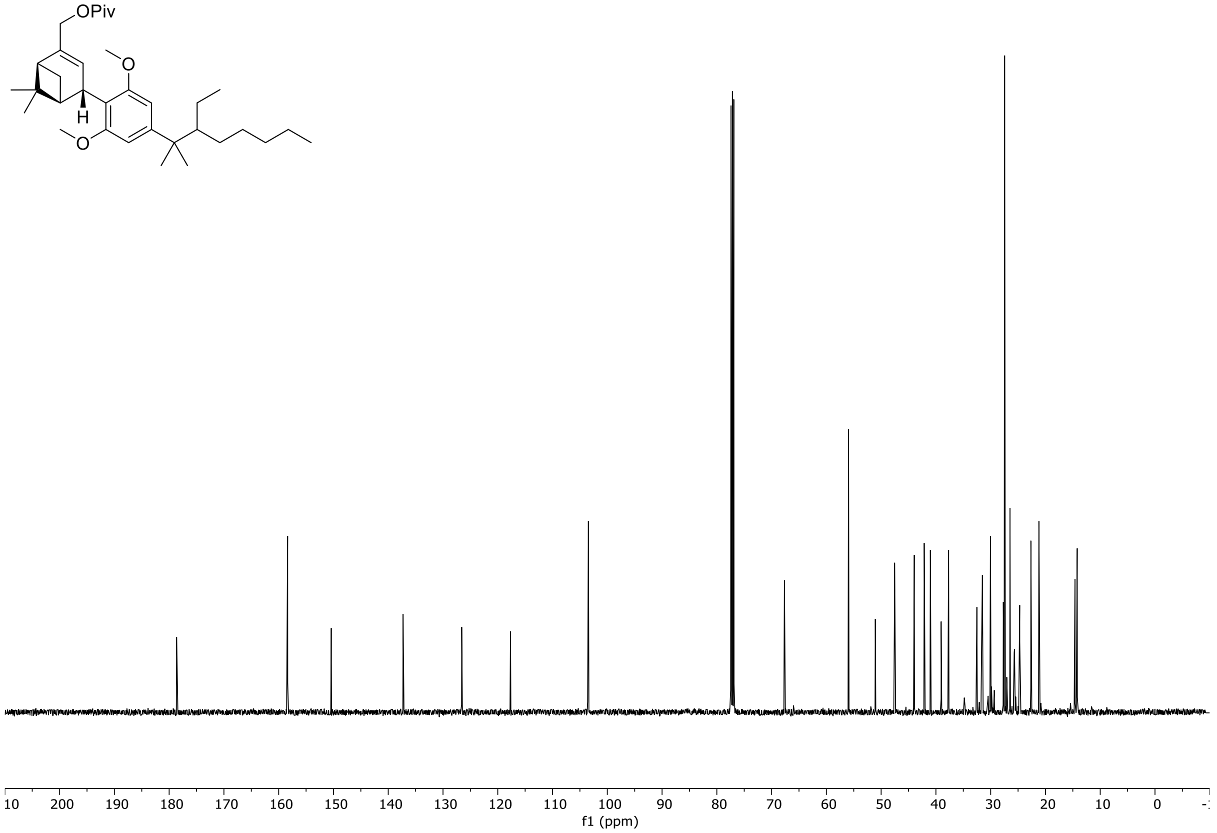

^{1}H NMR (400 MHz, $CDCl_3$) of (*S*/*R*)-**6c**

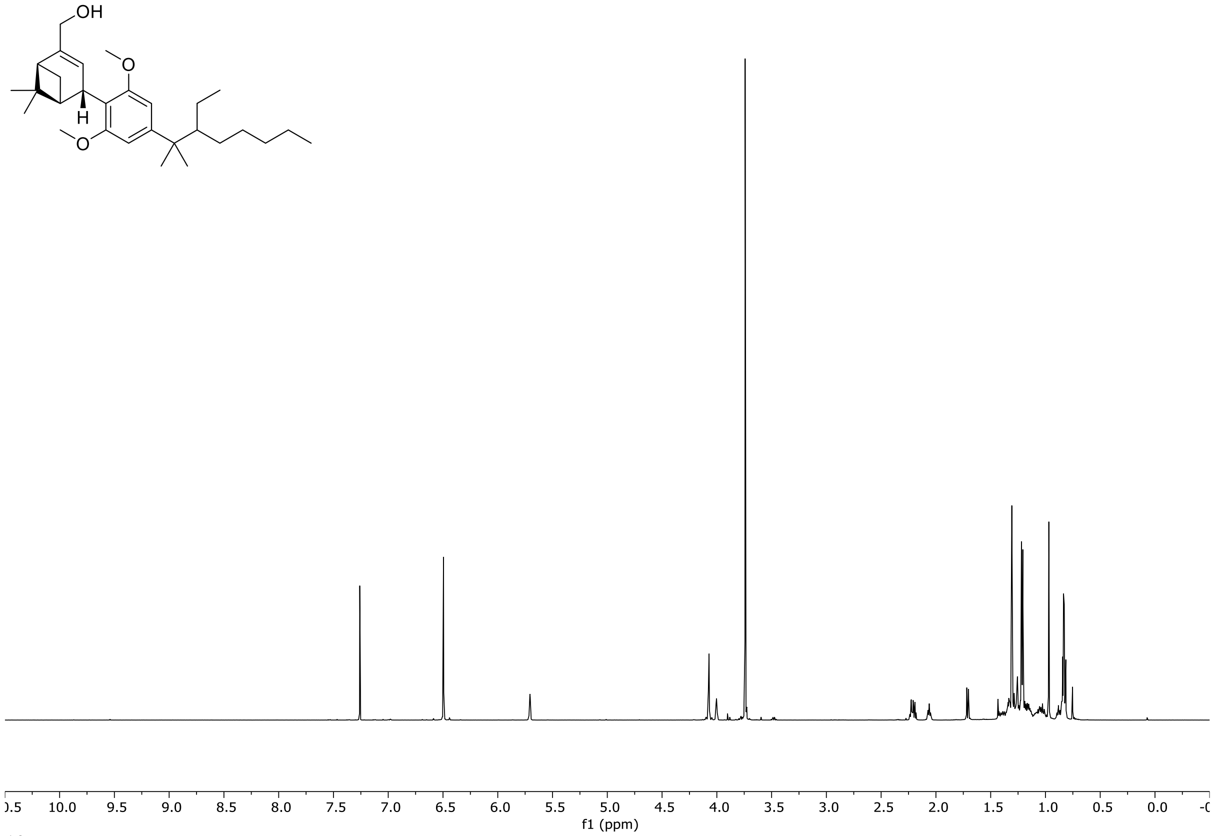

^{13}C NMR (101 MHz, $CDCl_3$) of (*S*/*R*)-**6c**

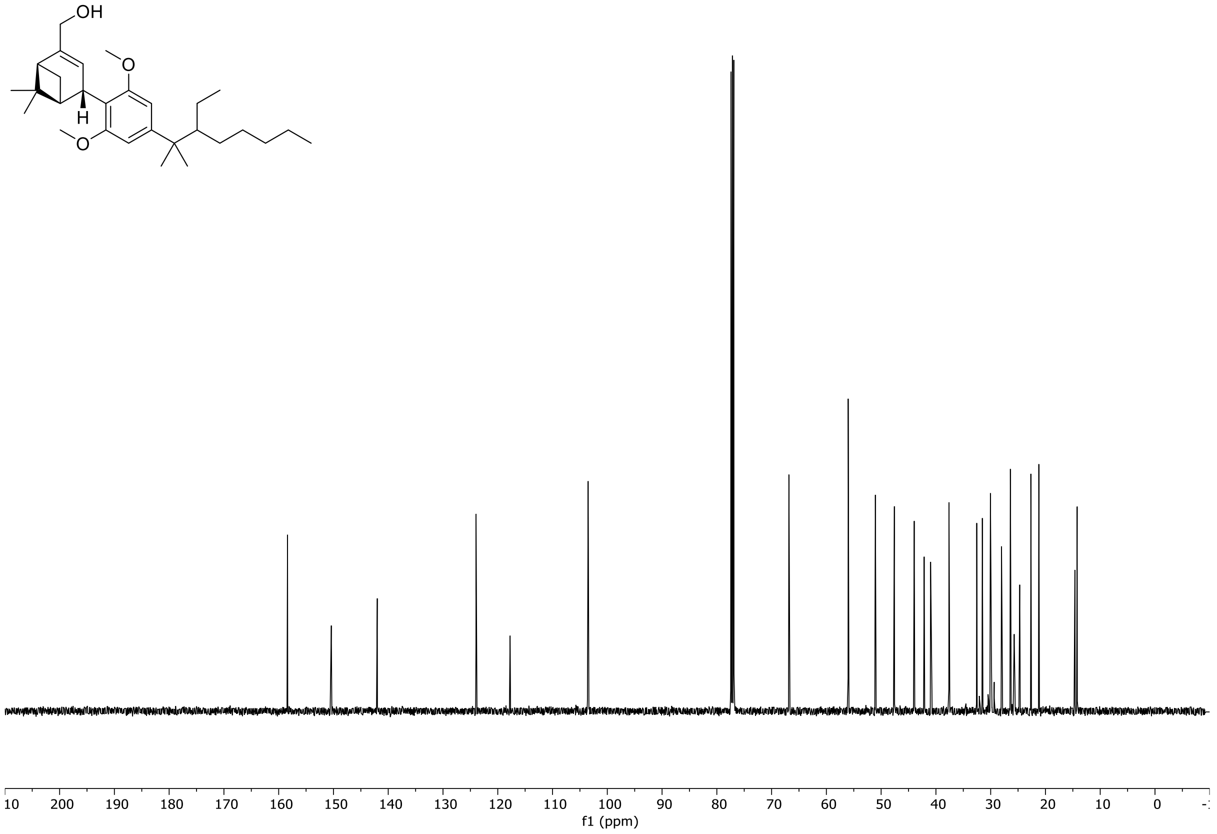

^{1}H NMR (400 MHz, $CDCl_3$) of (*S*)-**SI-20d**

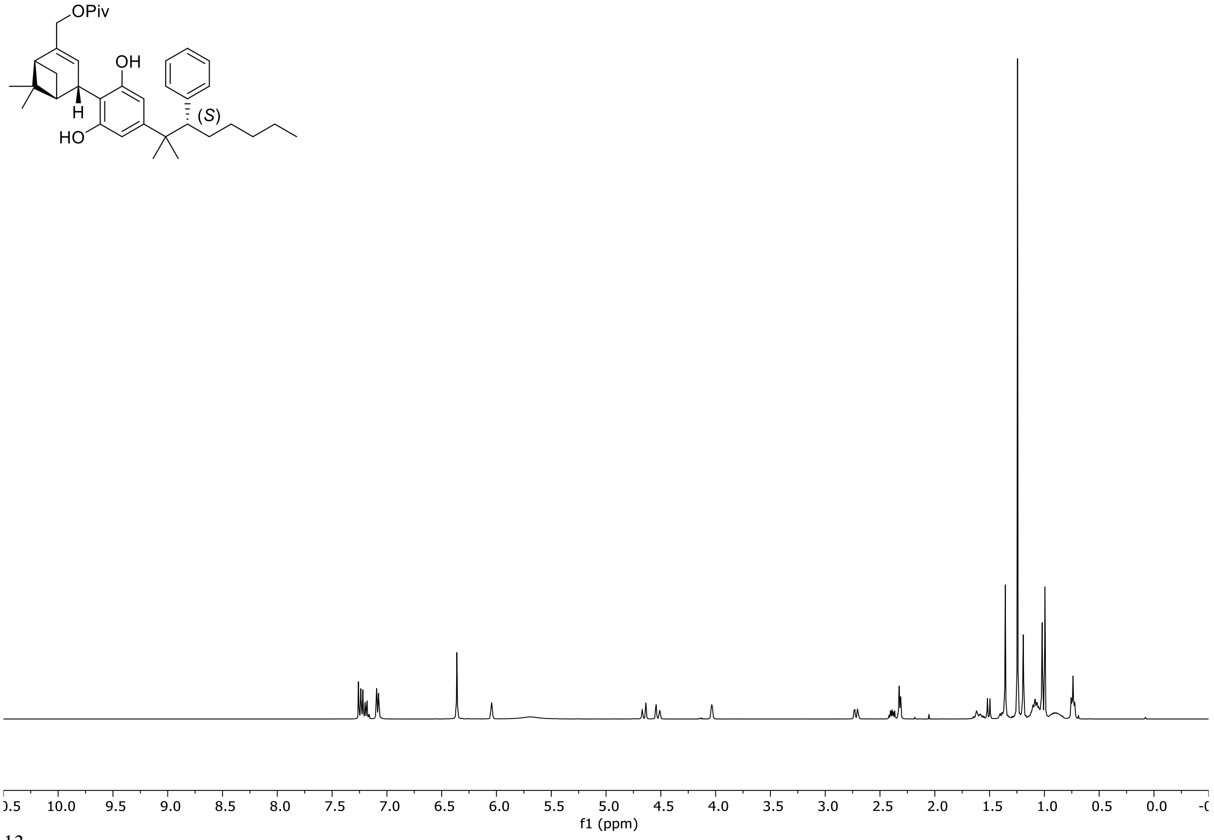

^{13}C NMR (101 MHz, $CDCl_3$) of (*S*)-**SI-20d**

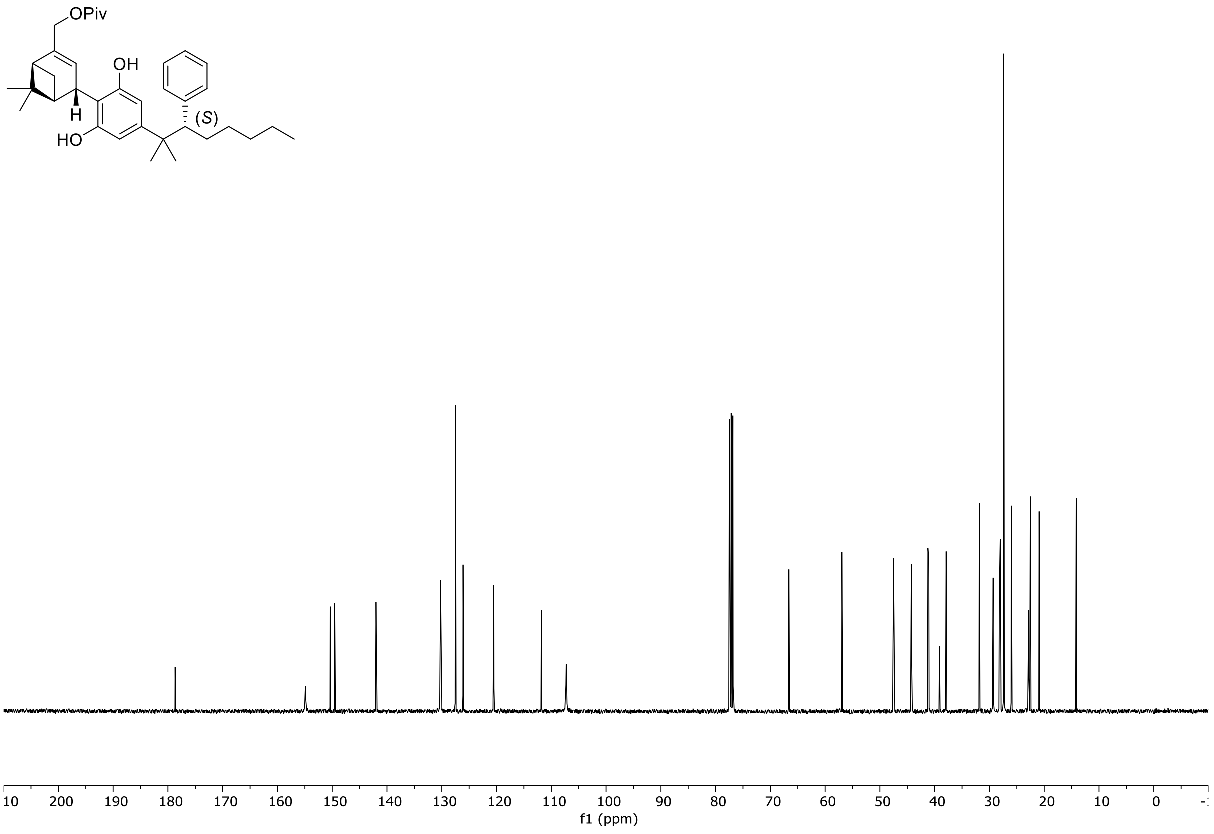

^{1}H NMR (400 MHz, $CDCl_3$) of (*R*)-**SI-20d**

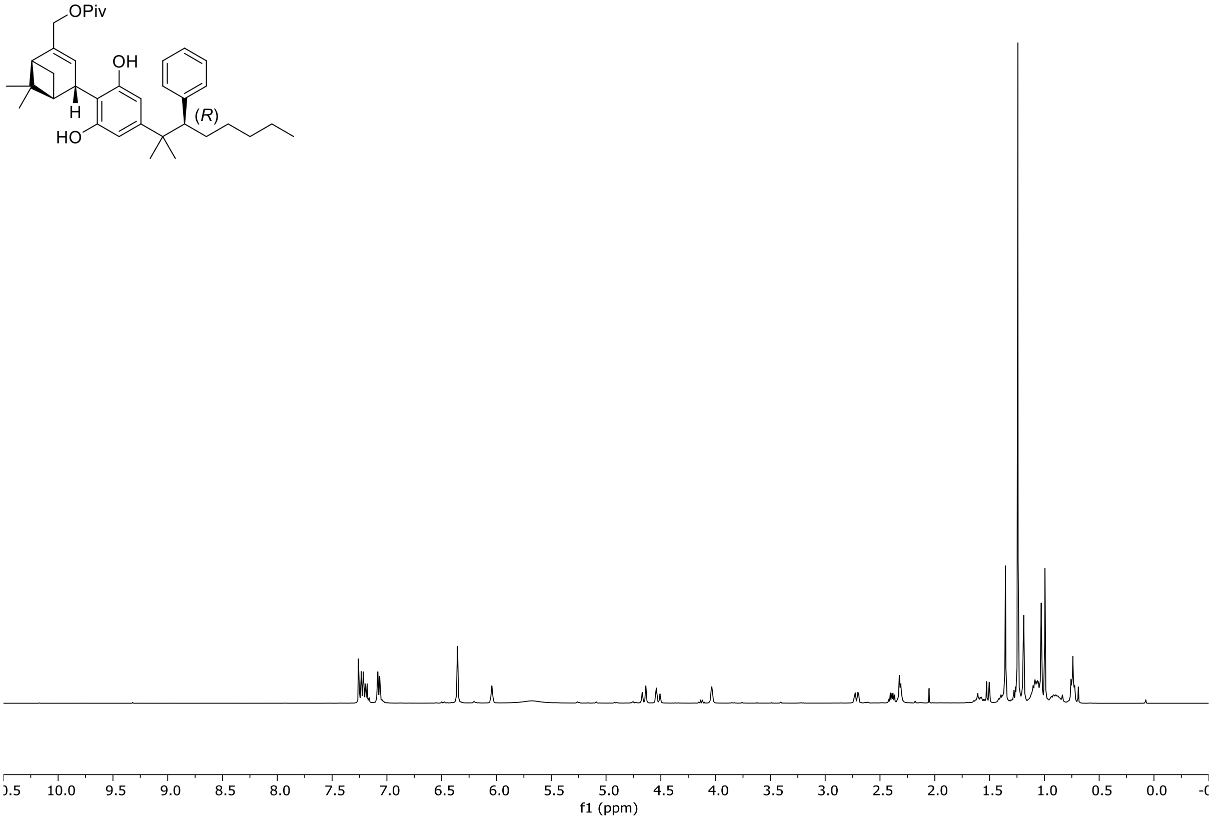

^{13}C NMR (101 MHz, $CDCl_3$) of (*R*)-**SI-20d**

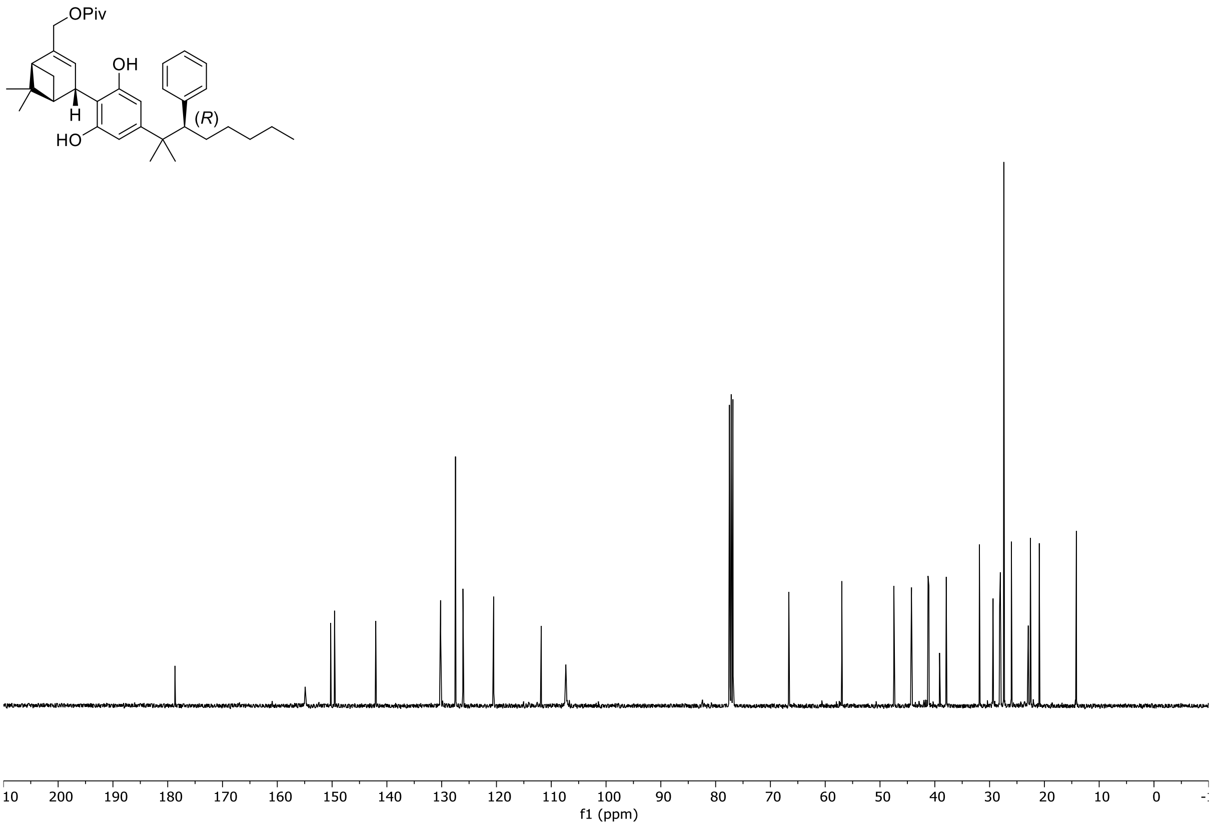

^{1}H NMR (500 MHz, $CDCl_3$) of (*S*)-**10d**

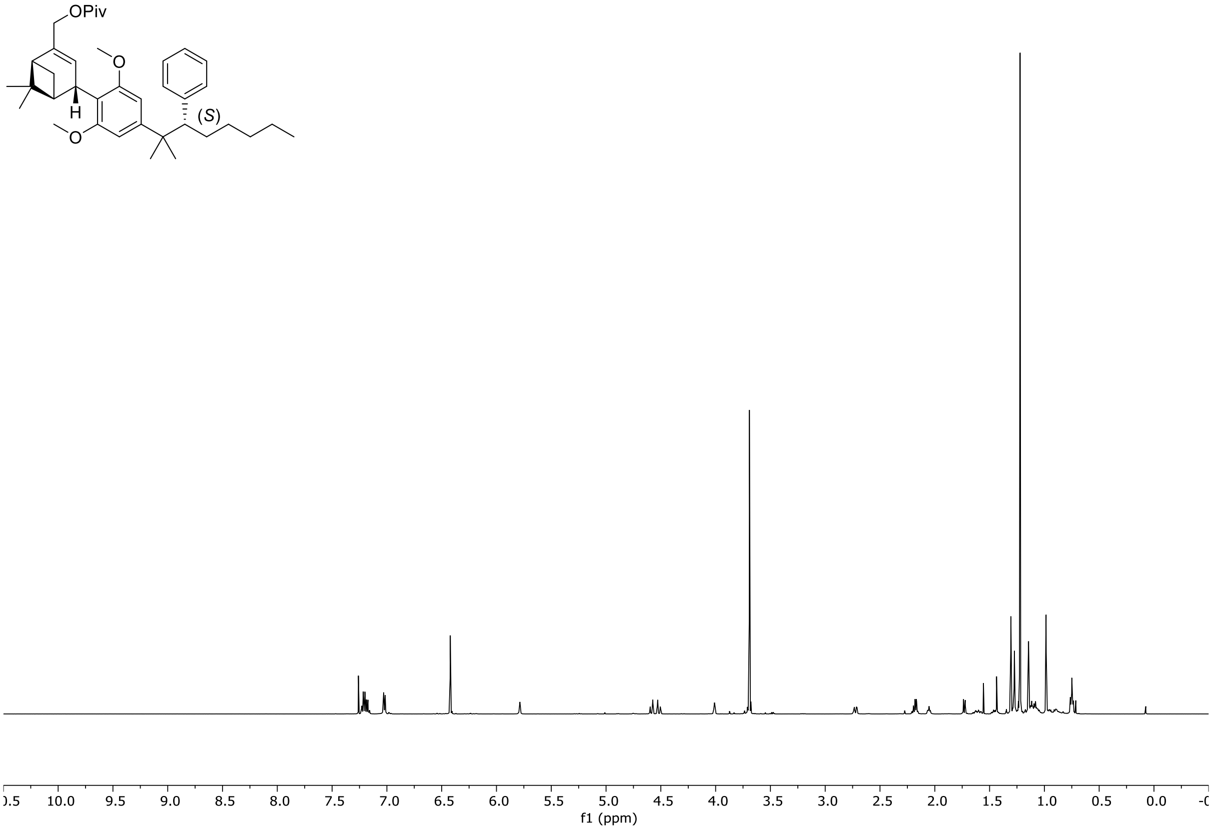

^{13}C NMR (126 MHz, $CDCl_3$) of (*S*)-**10d**

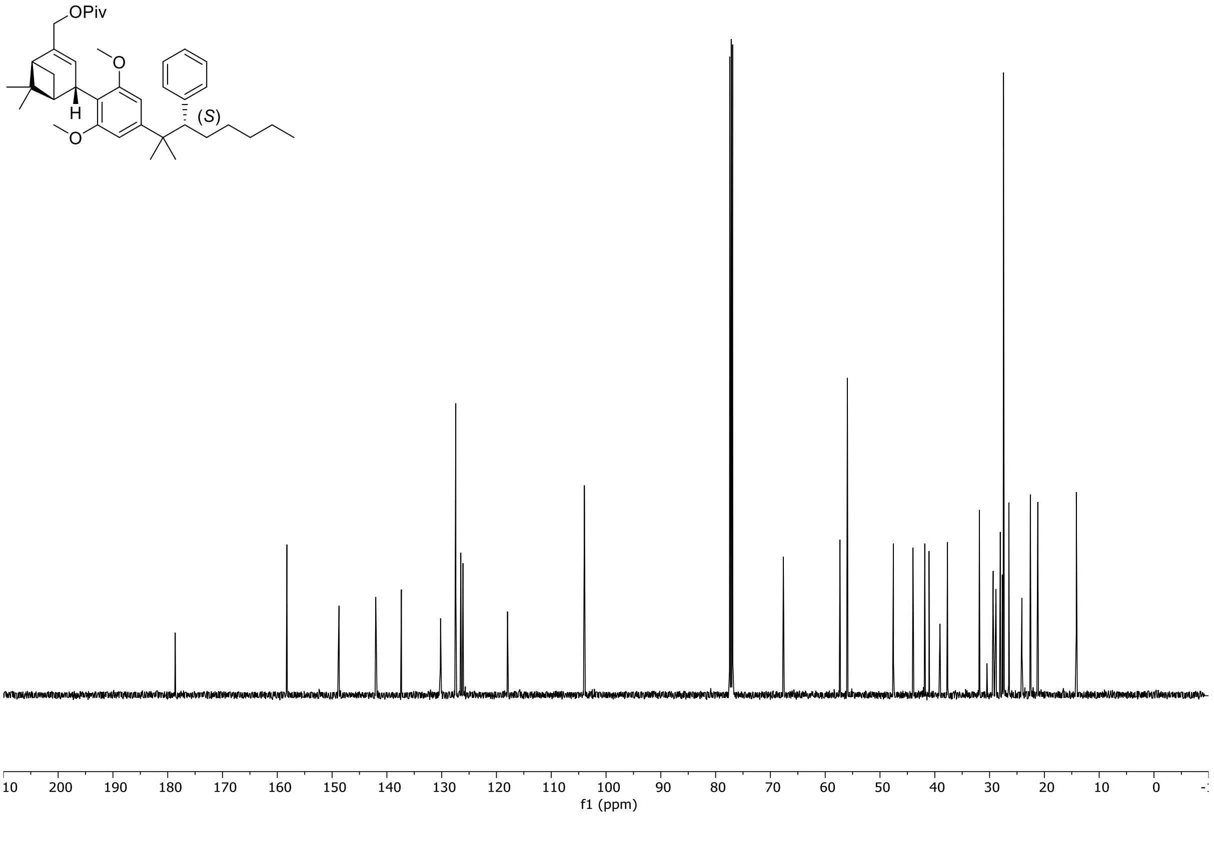

^{1}H NMR (400 MHz, $CDCl_3$) of (*R*)-**10d**

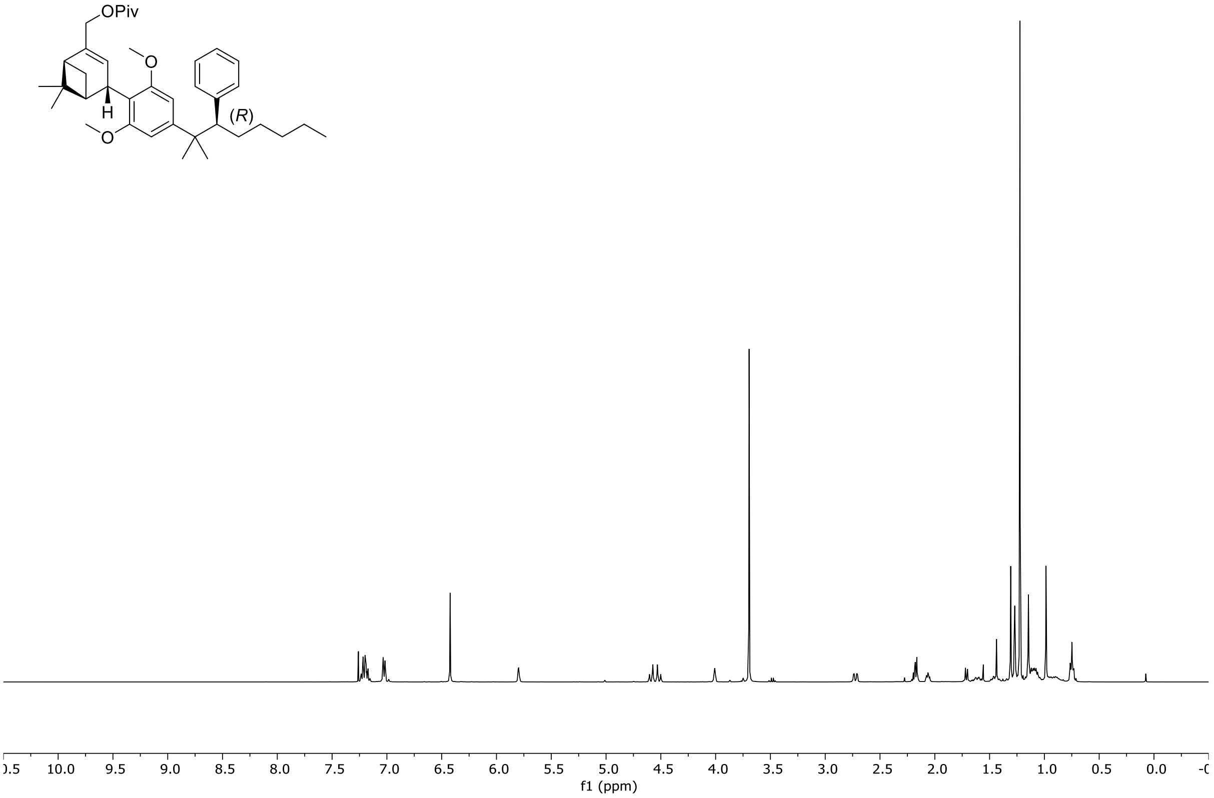

^{13}C NMR (101 MHz, $CDCl_3$) of (*R*)-**10d**

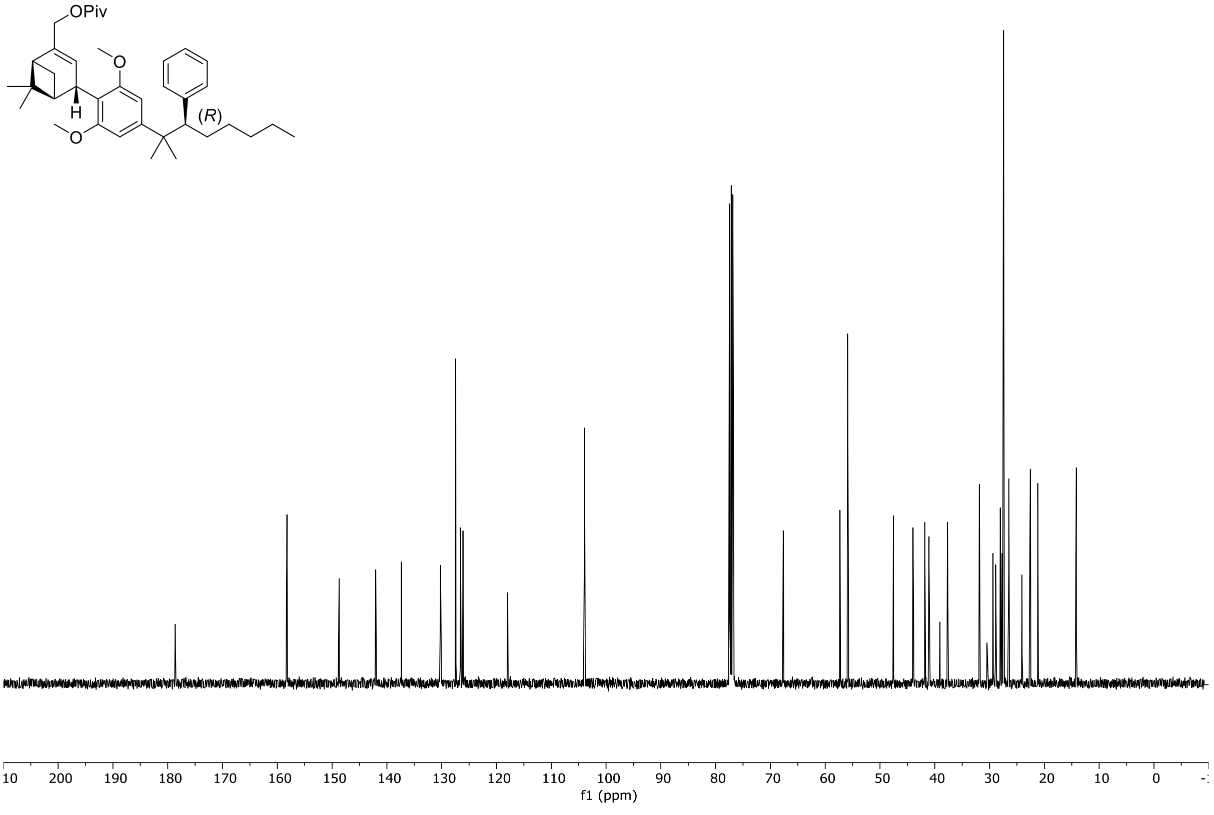

^{1}H NMR (400 MHz, $CDCl_3$) of (*S*)-**6d**

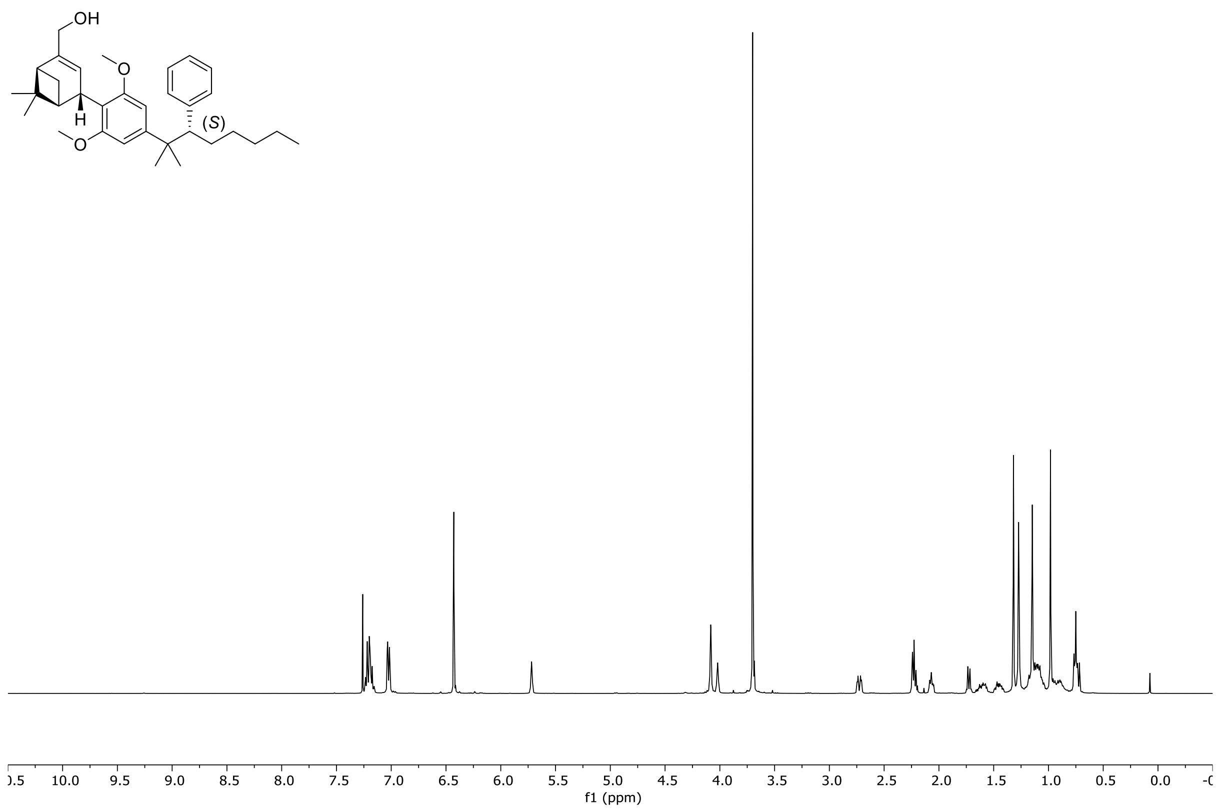

^{13}C NMR (101 MHz, $CDCl_3$) of (*S*)-**6d**

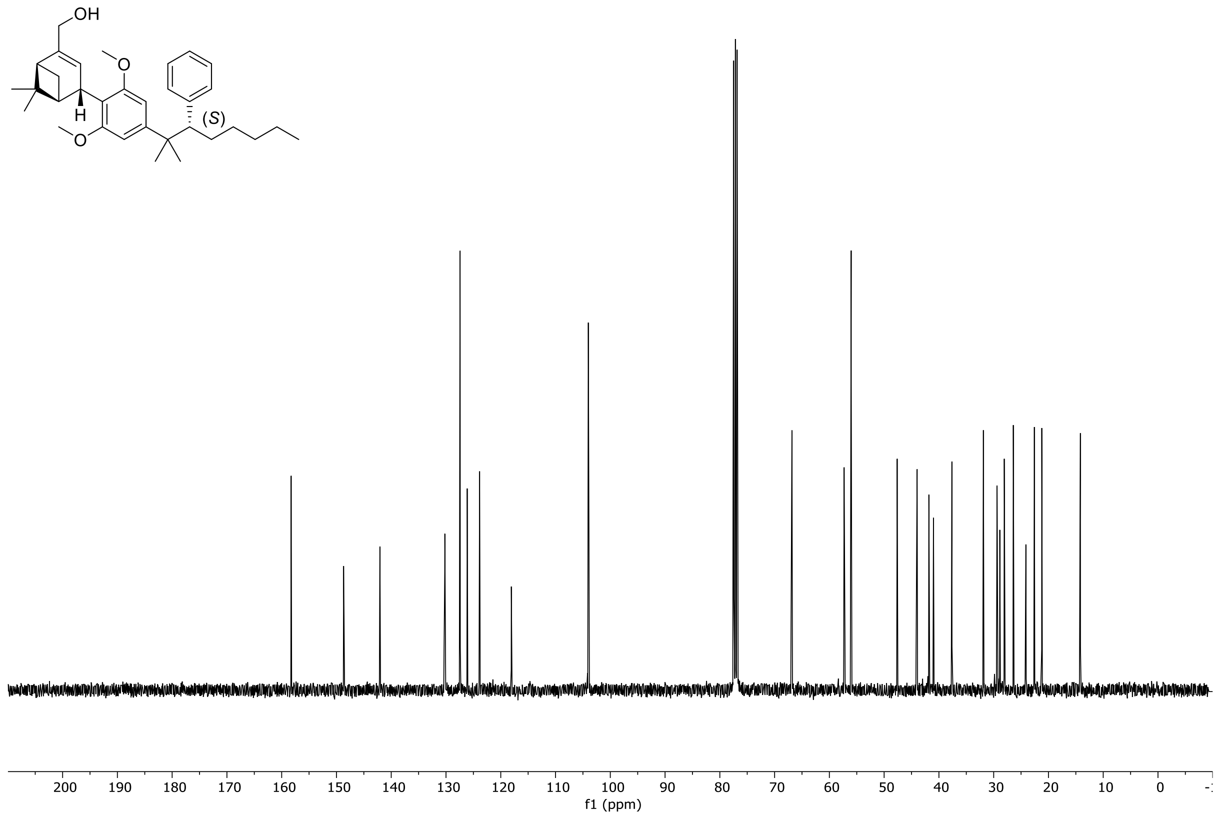

^{1}H NMR (400 MHz, $CDCl_3$) of (*R*)-**6d**

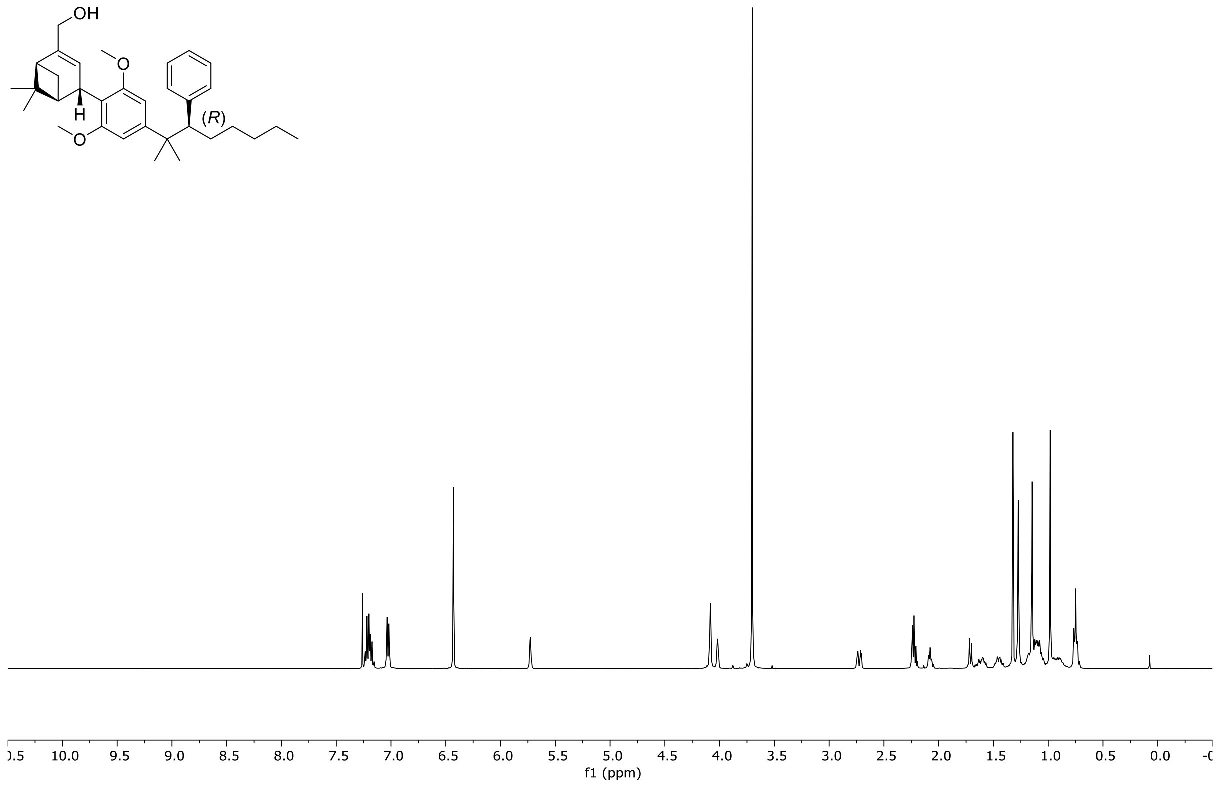

^{13}C NMR (101 MHz, $CDCl_3$) of (*R*)-**6d**

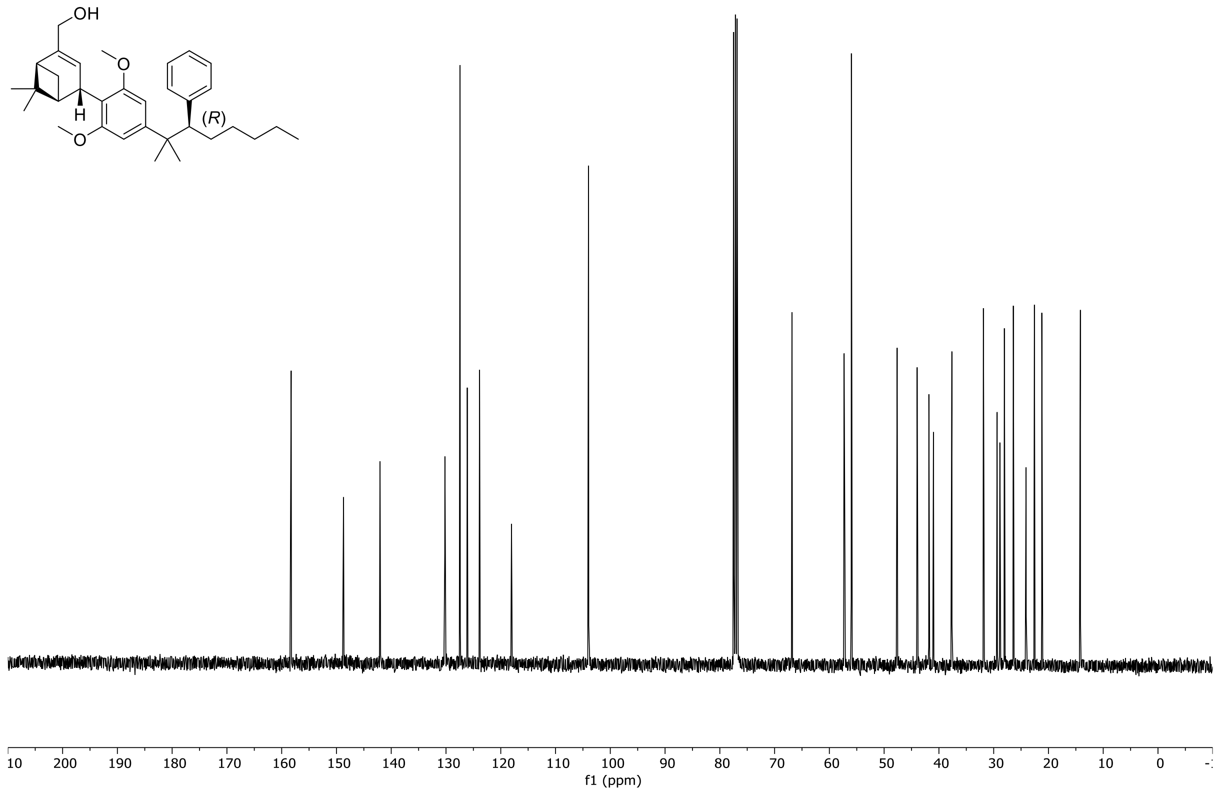

^{1}H NMR (400 MHz, $CDCl_3$) of (*S*)-**SI-20e**

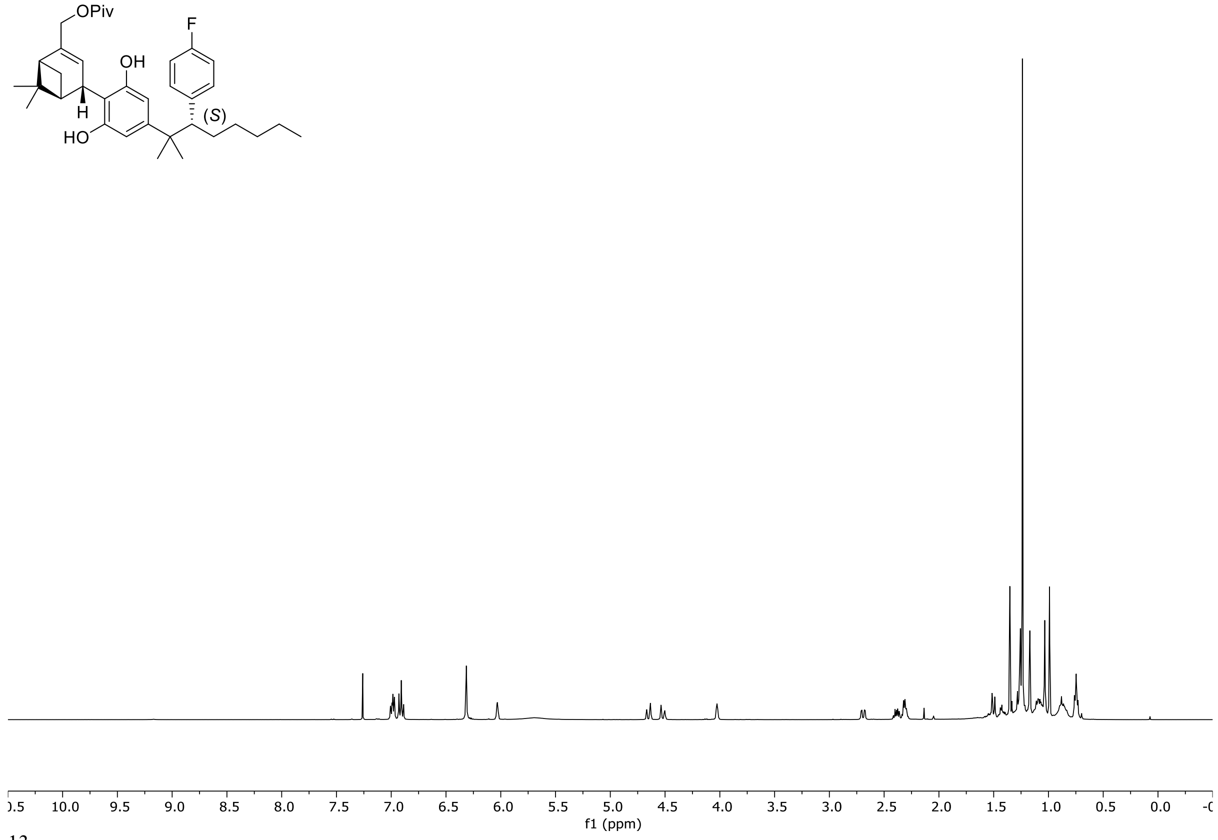

^{13}C NMR (101 MHz, $CDCl_3$) of (*S*)-**SI-20e**

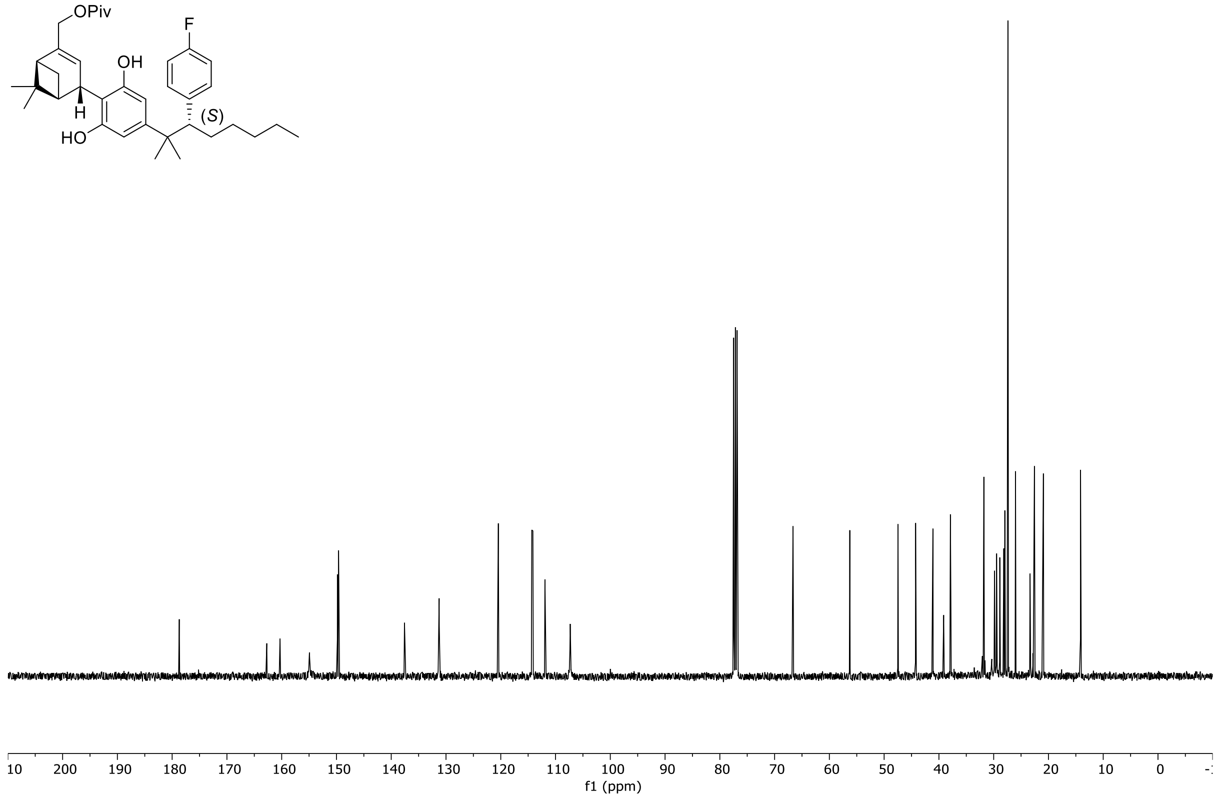

^{19}F NMR (377 MHz, $CDCl_3$) of (*S*)-**SI-20e**

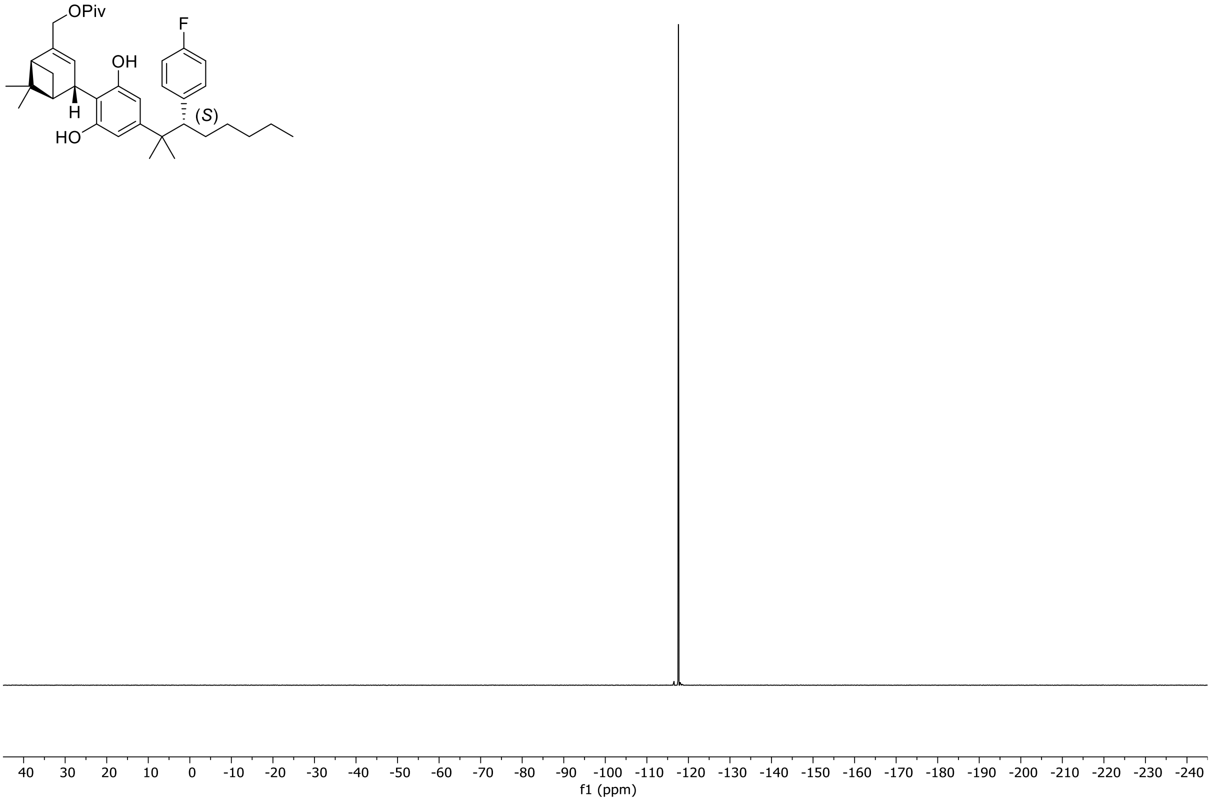

^{1}H NMR (500 MHz, $CDCl_3$) of (*R*)-**SI-20e**

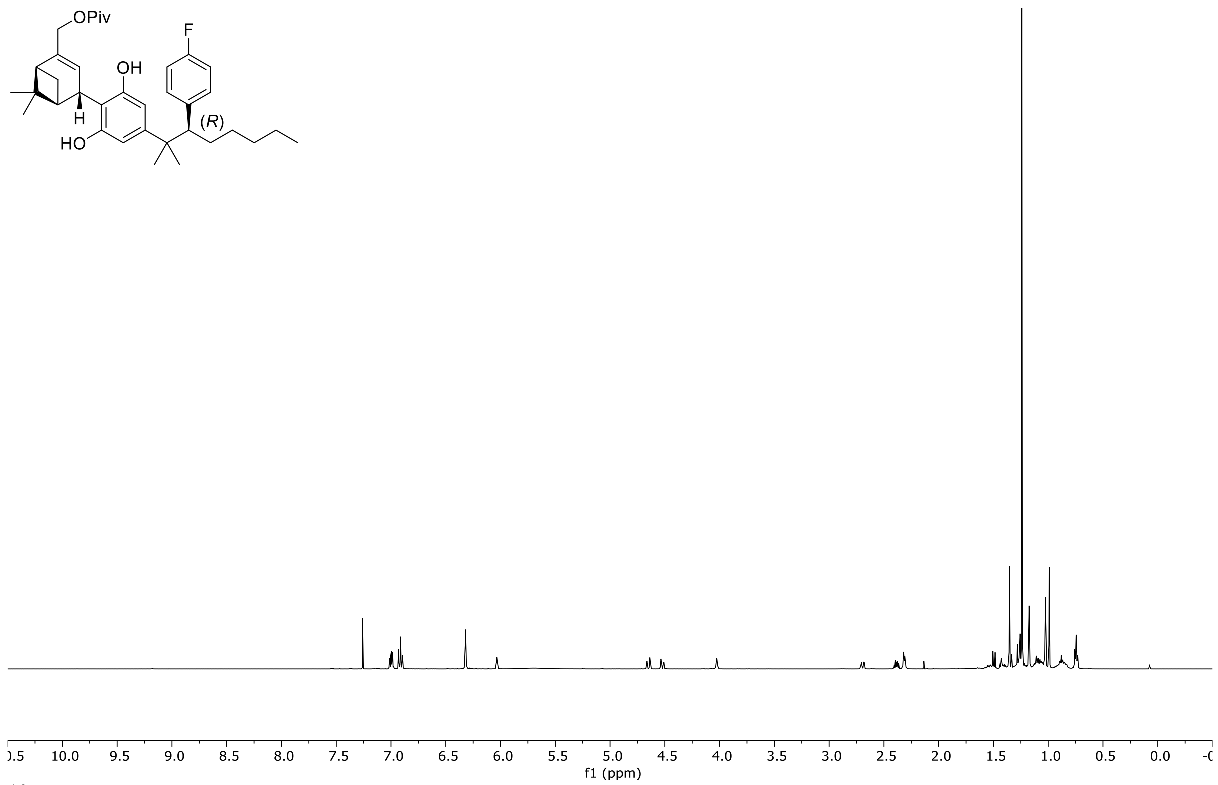

^{13}C NMR (126 MHz, $CDCl_3$) of (*R*)-**SI-20e**

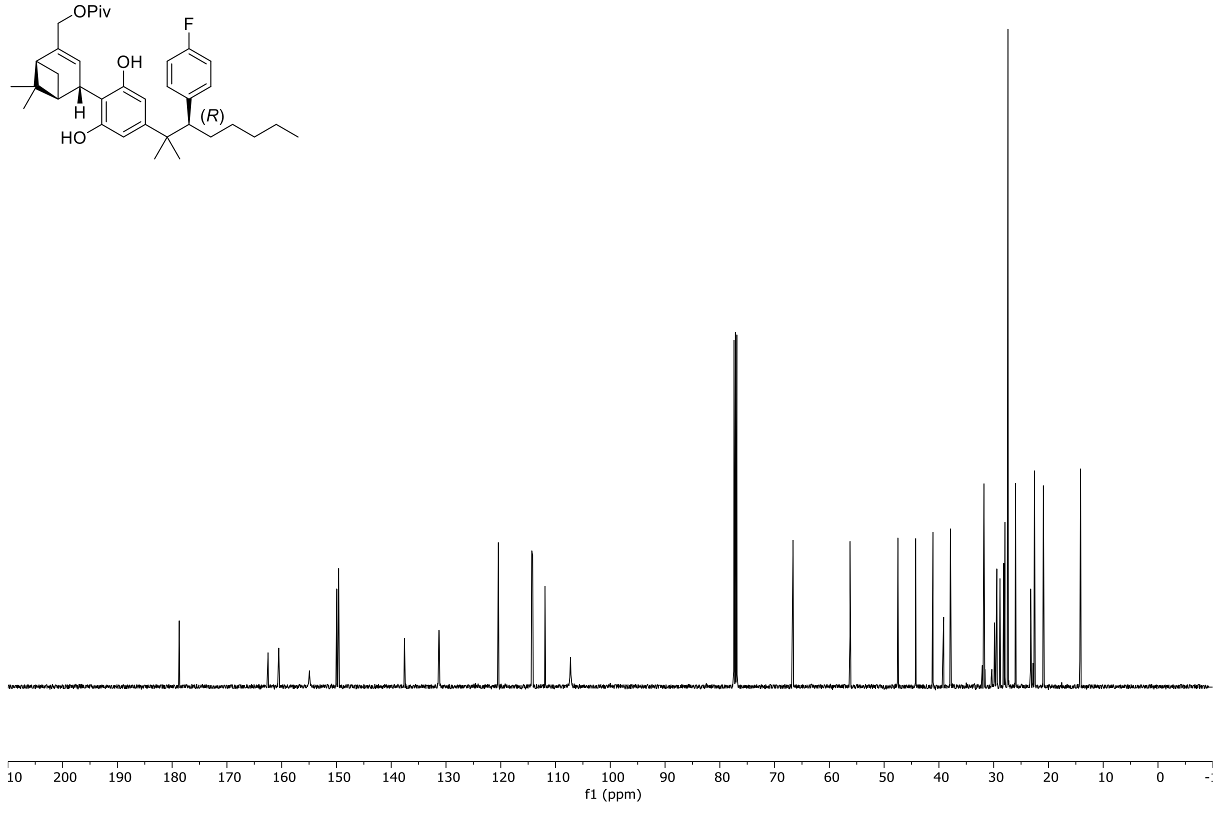

^{19}F NMR (471 MHz, $CDCl_3$) of (*R*)-**SI-20e**

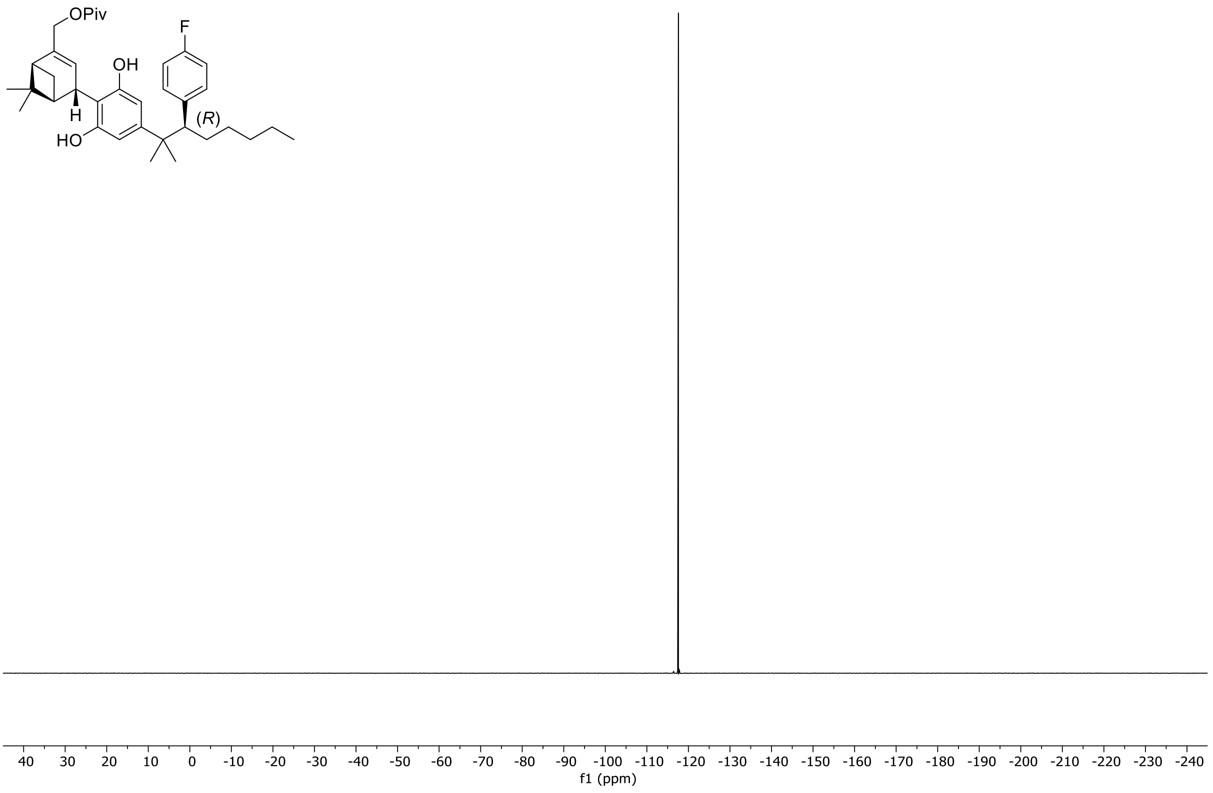

^{1}H NMR (400 MHz, $CDCl_3$) of (*S*)-**10e**

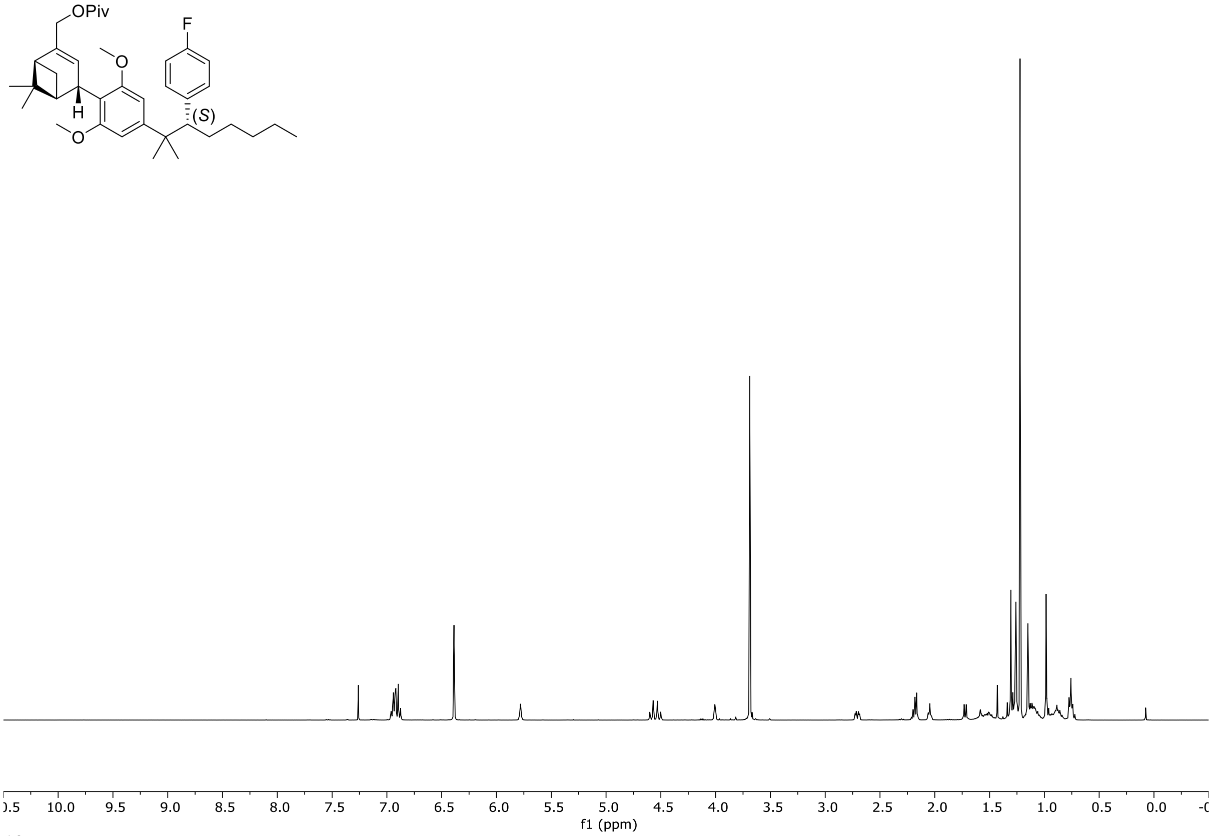

^{13}C NMR (101 MHz, $CDCl_3$) of (*S*)-**10e**

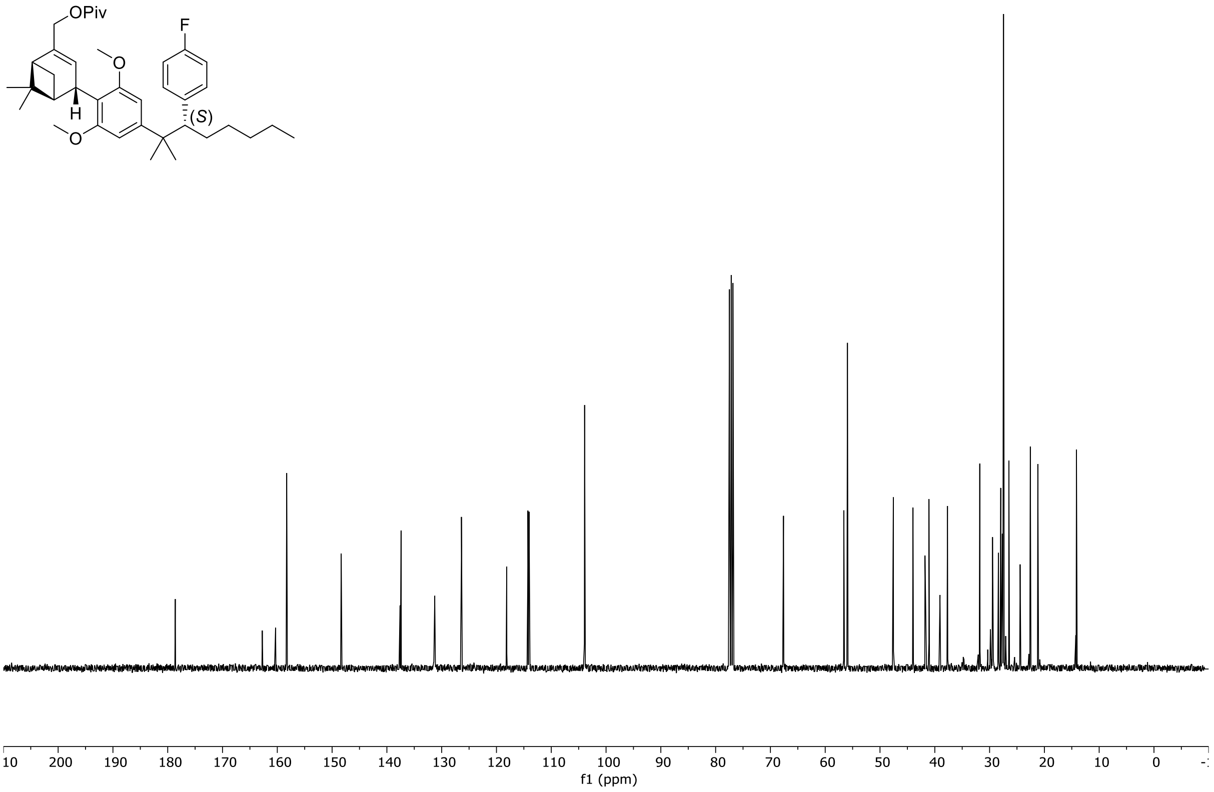

^{19}F NMR (376 MHz, $CDCl_3$) of (*S*)-**10e**

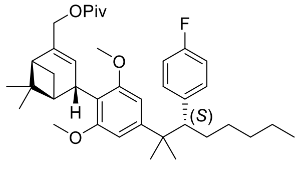

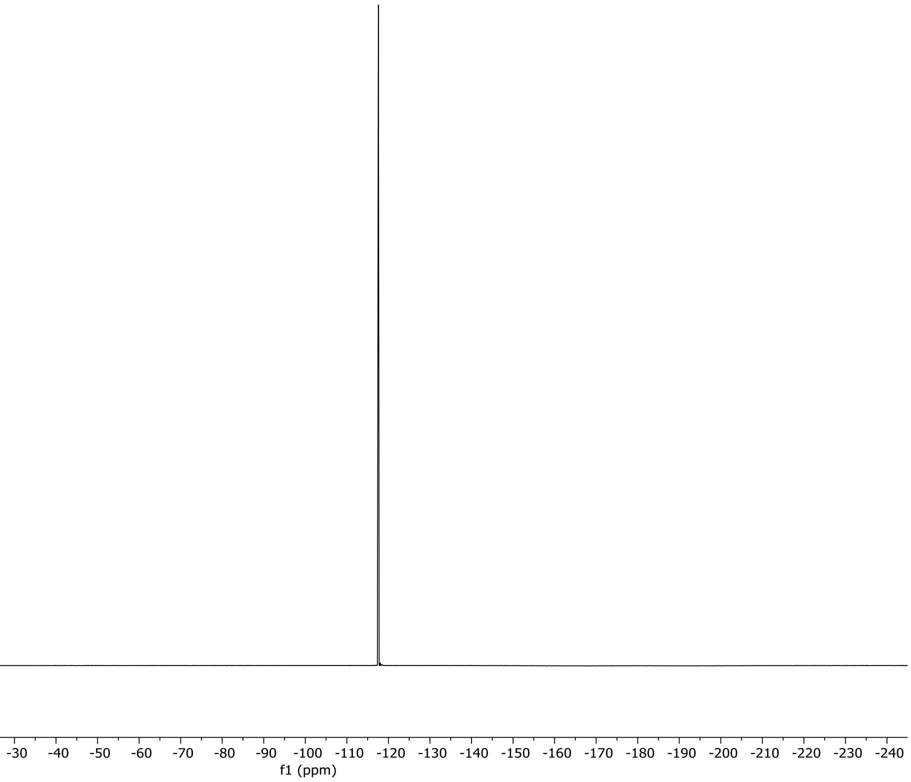

^{1}H NMR (400 MHz, $CDCl_3$) of (*R*)-**10e**

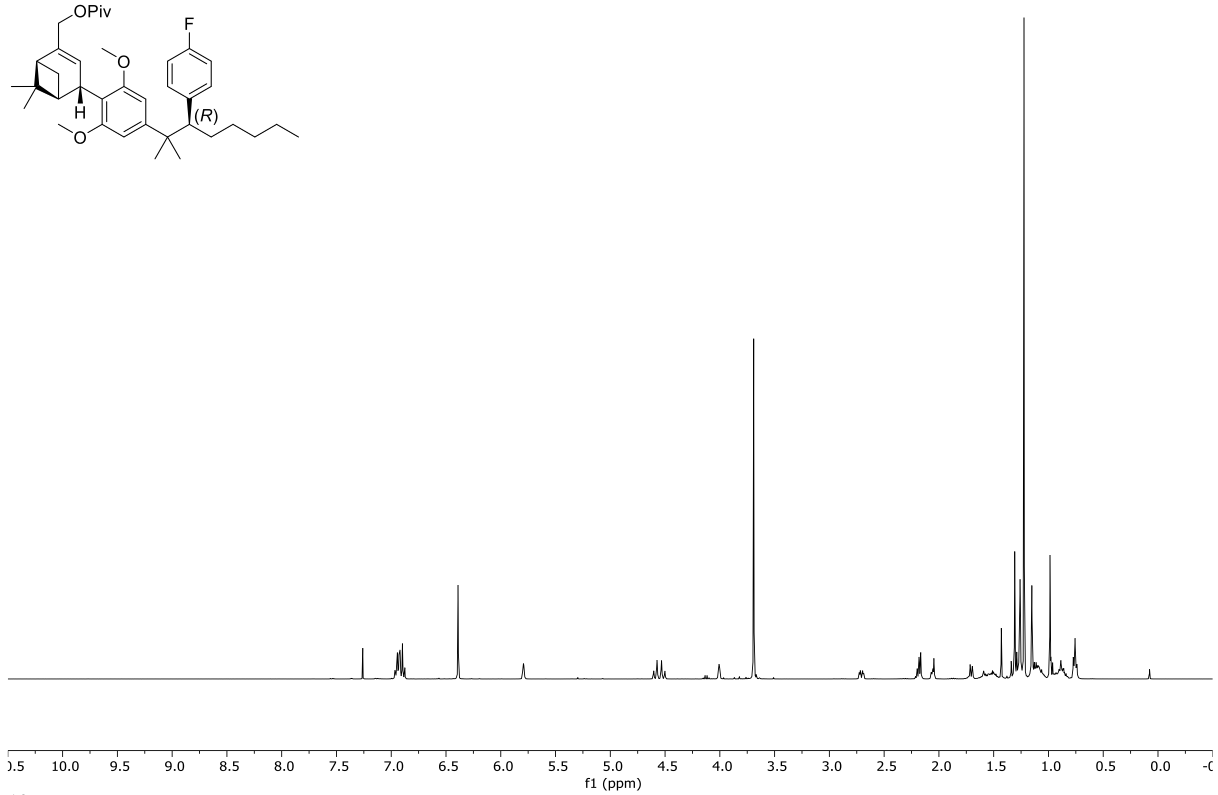

^{13}C NMR (101 MHz, $CDCl_3$) of (*R*)-**10e**

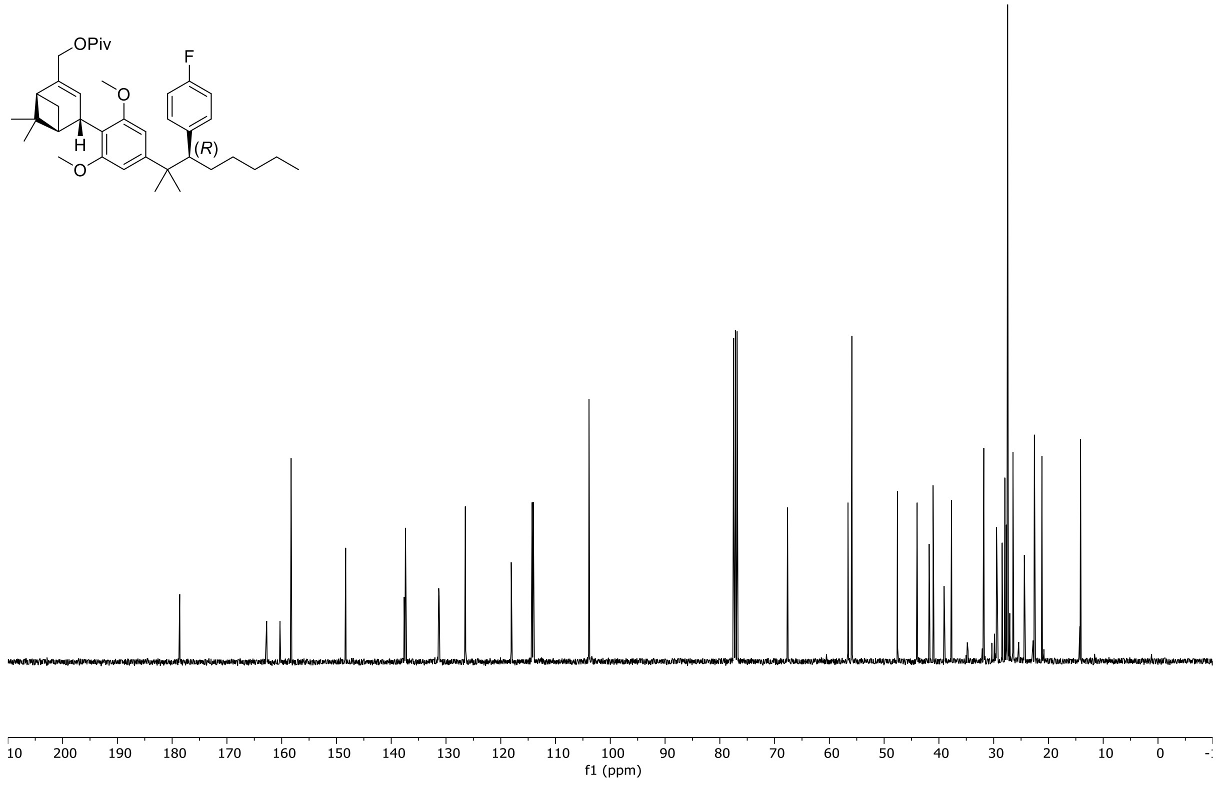

^{19}F NMR (376 MHz, CDCl$_3$) of (*R*)-**10e**

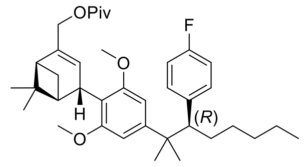

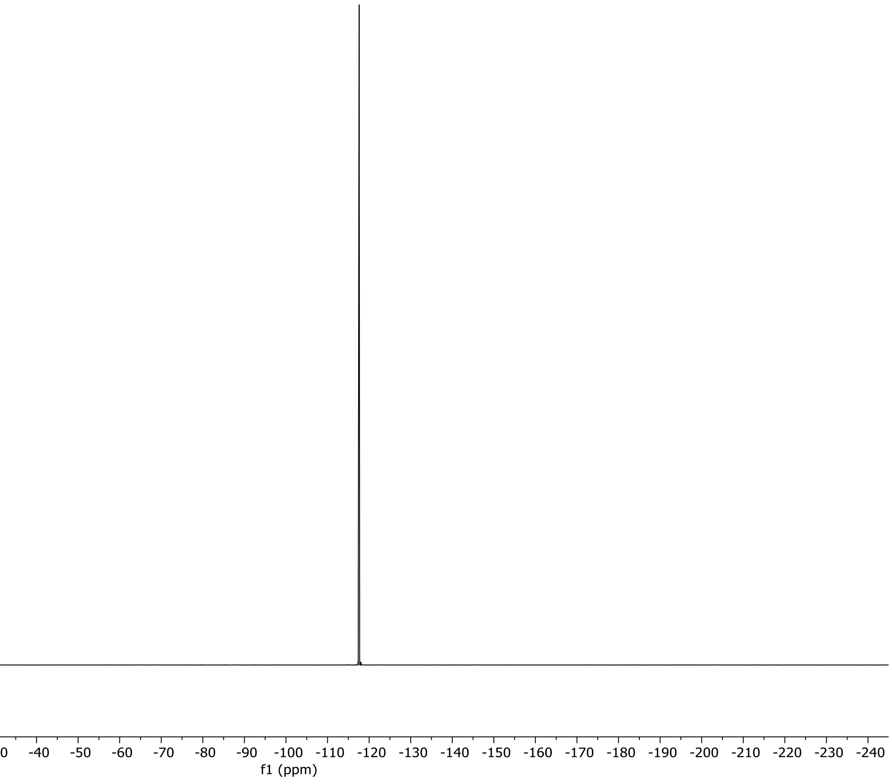

^{1}H NMR (400 MHz, $CDCl_3$) of (*S*)-**6e**

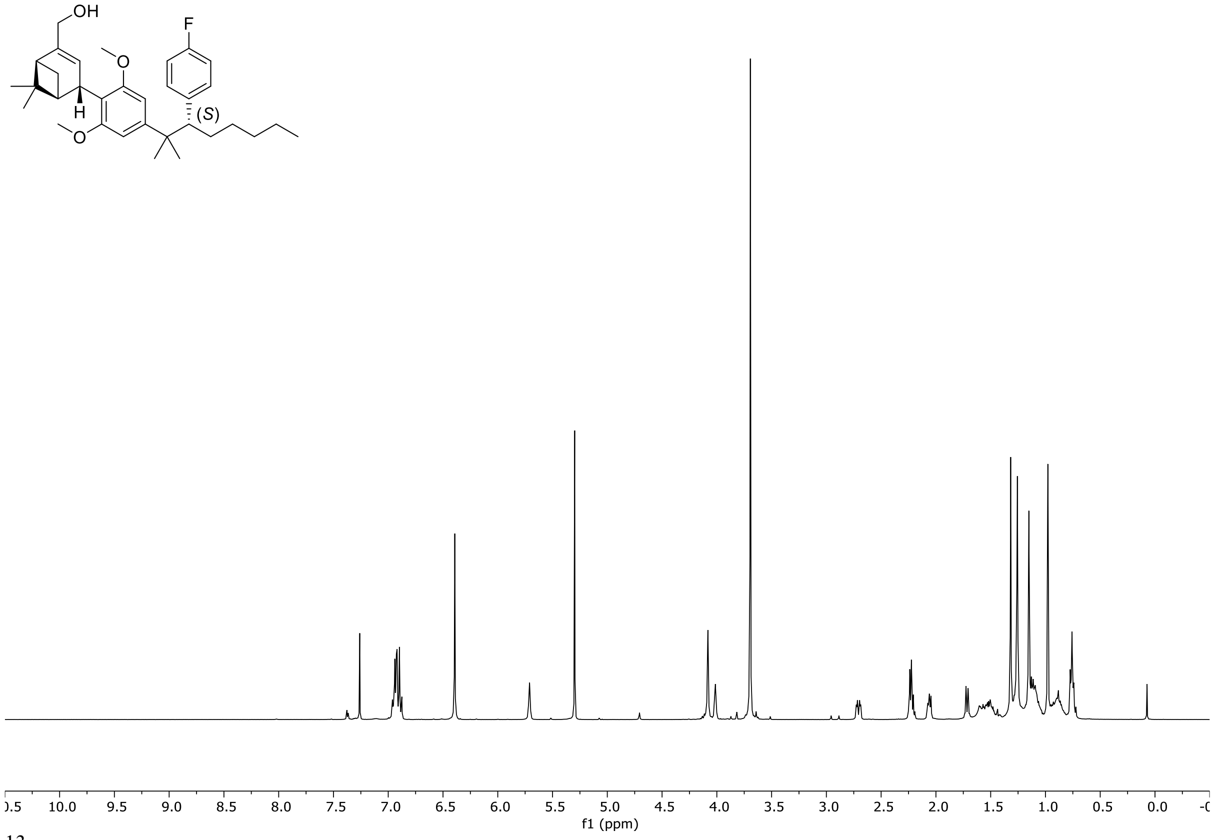

^{13}C NMR (101 MHz, $CDCl_3$) of (*S*)-**6e**

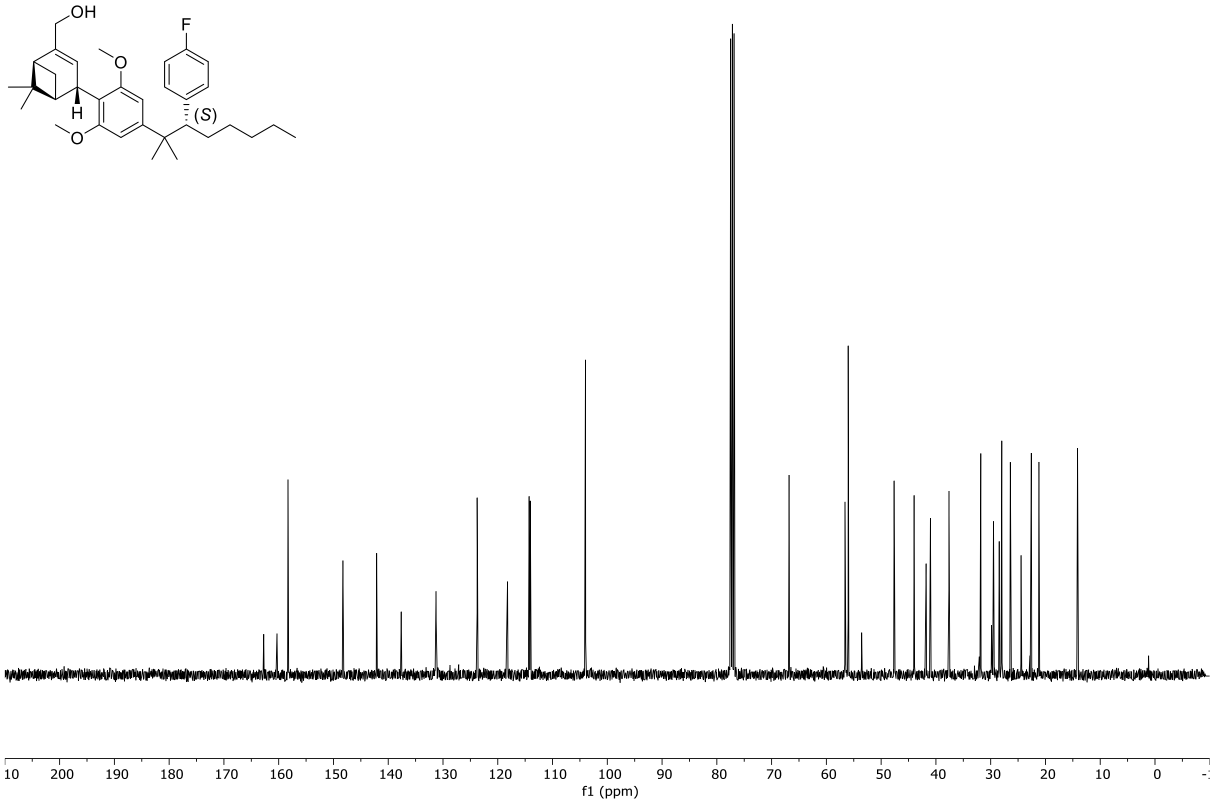

^{19}F NMR (376 MHz, CDCl$_3$) of (*S*)-**6e**

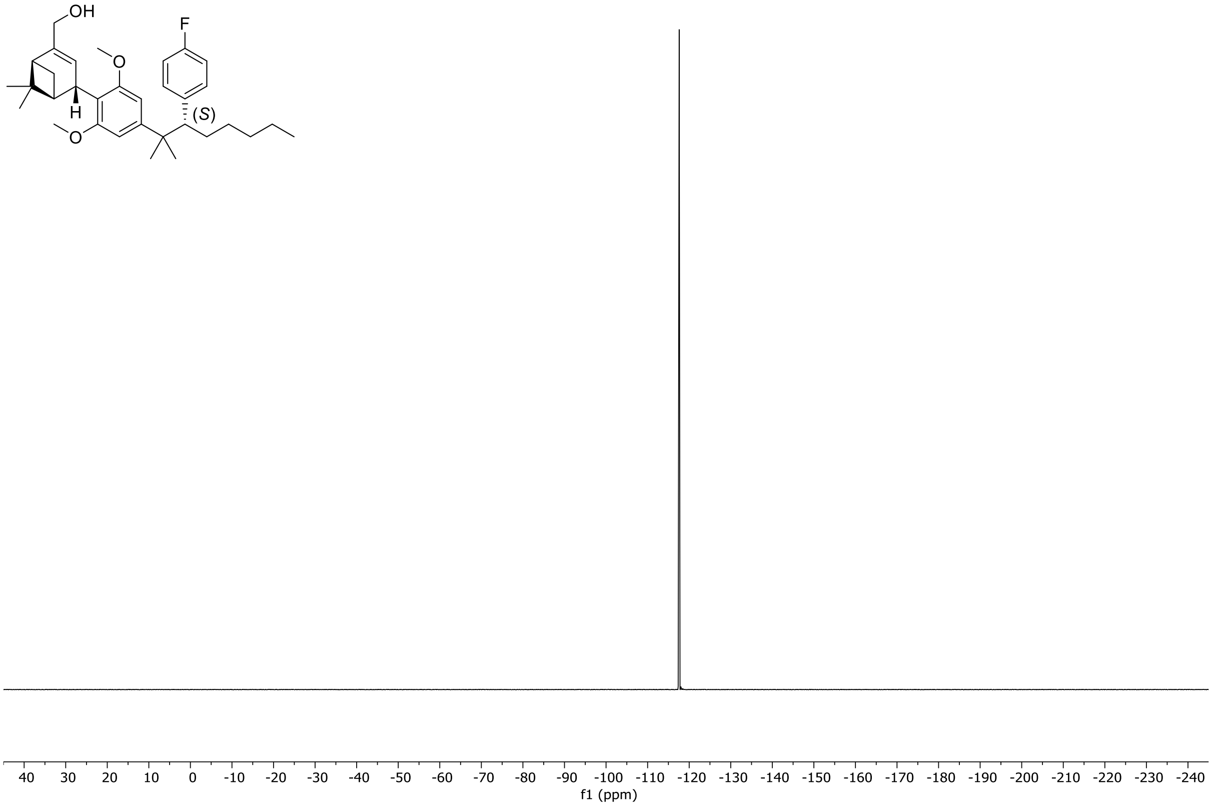

^{1}H NMR (400 MHz, $CDCl_3$) of (*R*)-**6e**

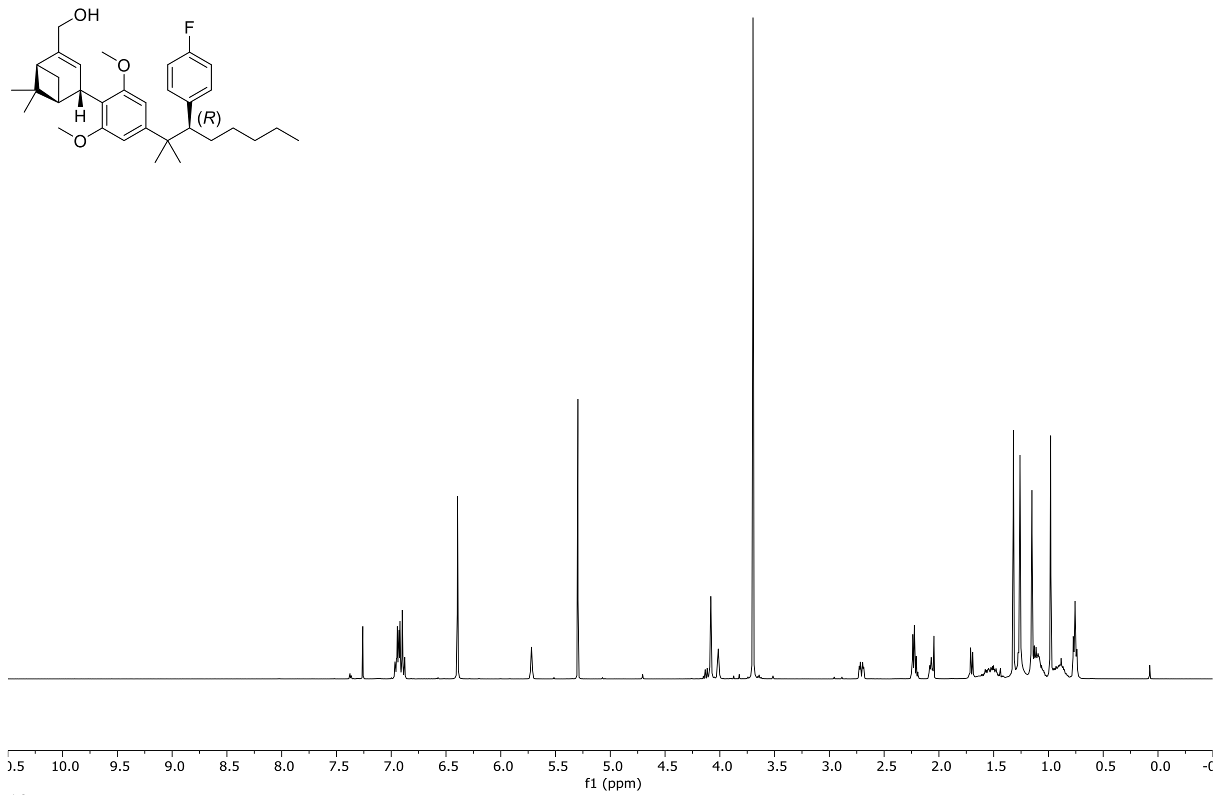

^{13}C NMR (101 MHz, $CDCl_3$) of (*R*)-**6e**

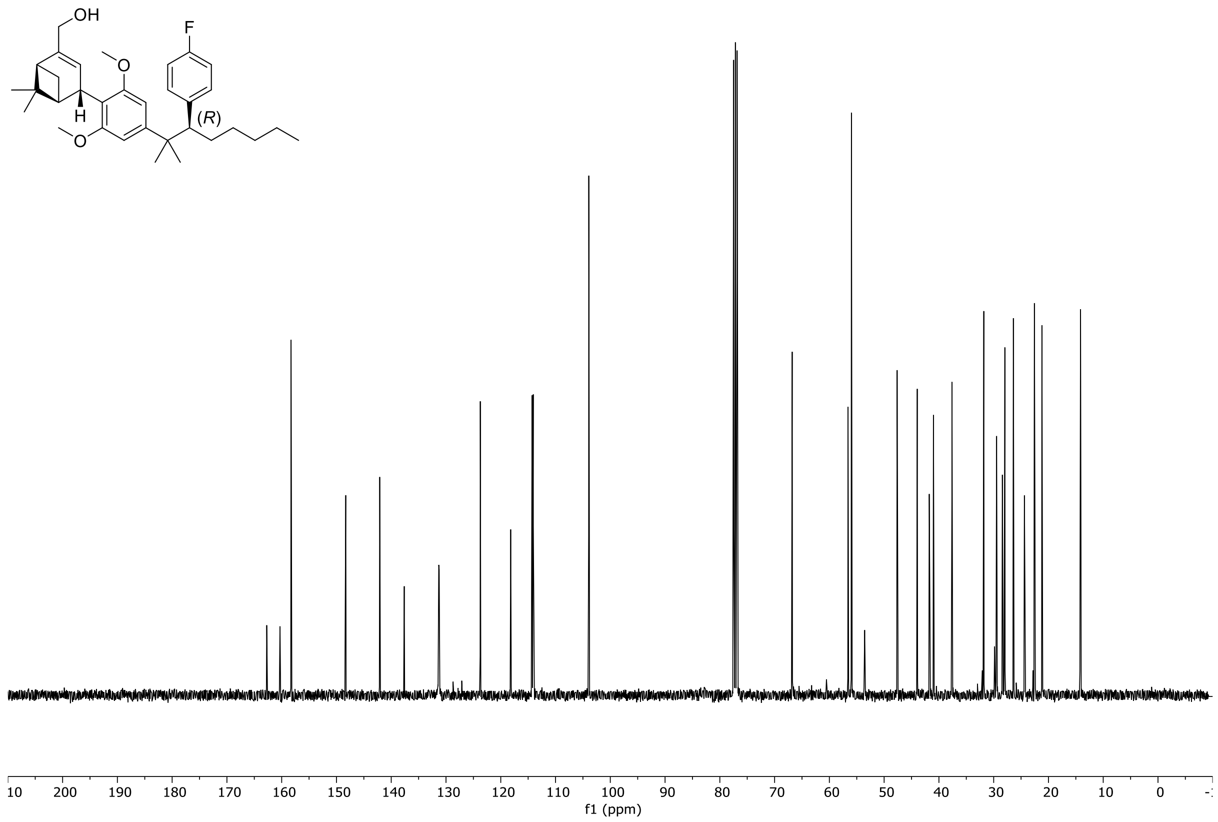

^{19}F NMR (376 MHz, $CDCl_3$) of (*R*)-**6e**

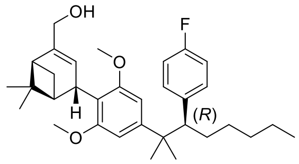

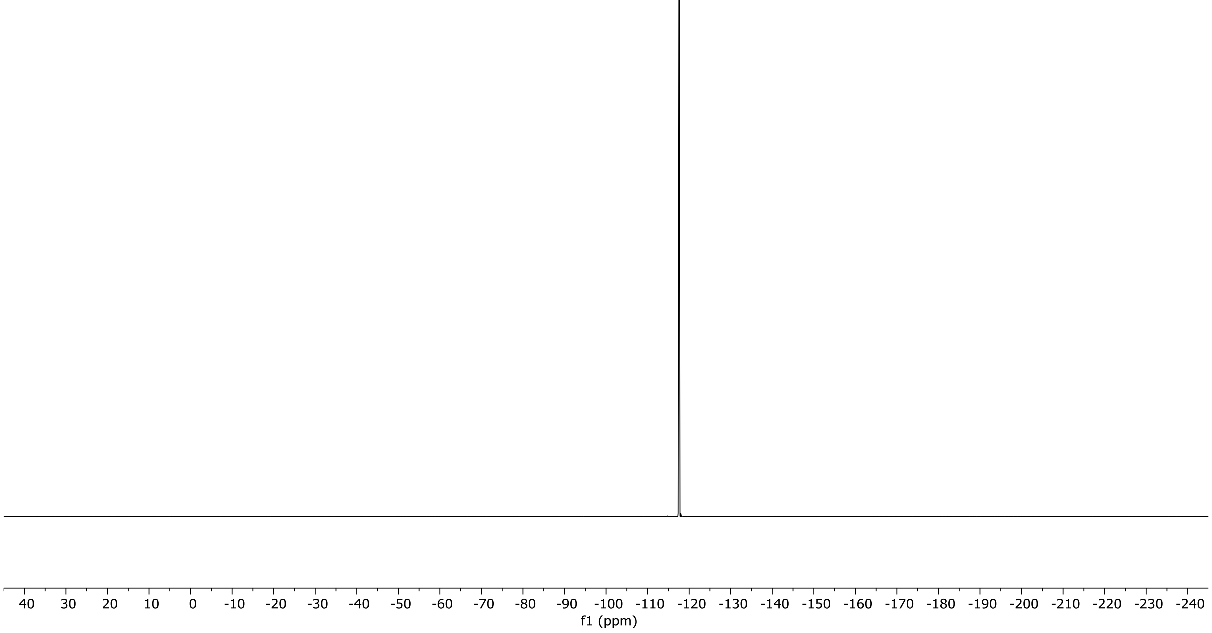

^{1}H NMR (400 MHz, $CDCl_3$) of (*S*)-**SI-20f**

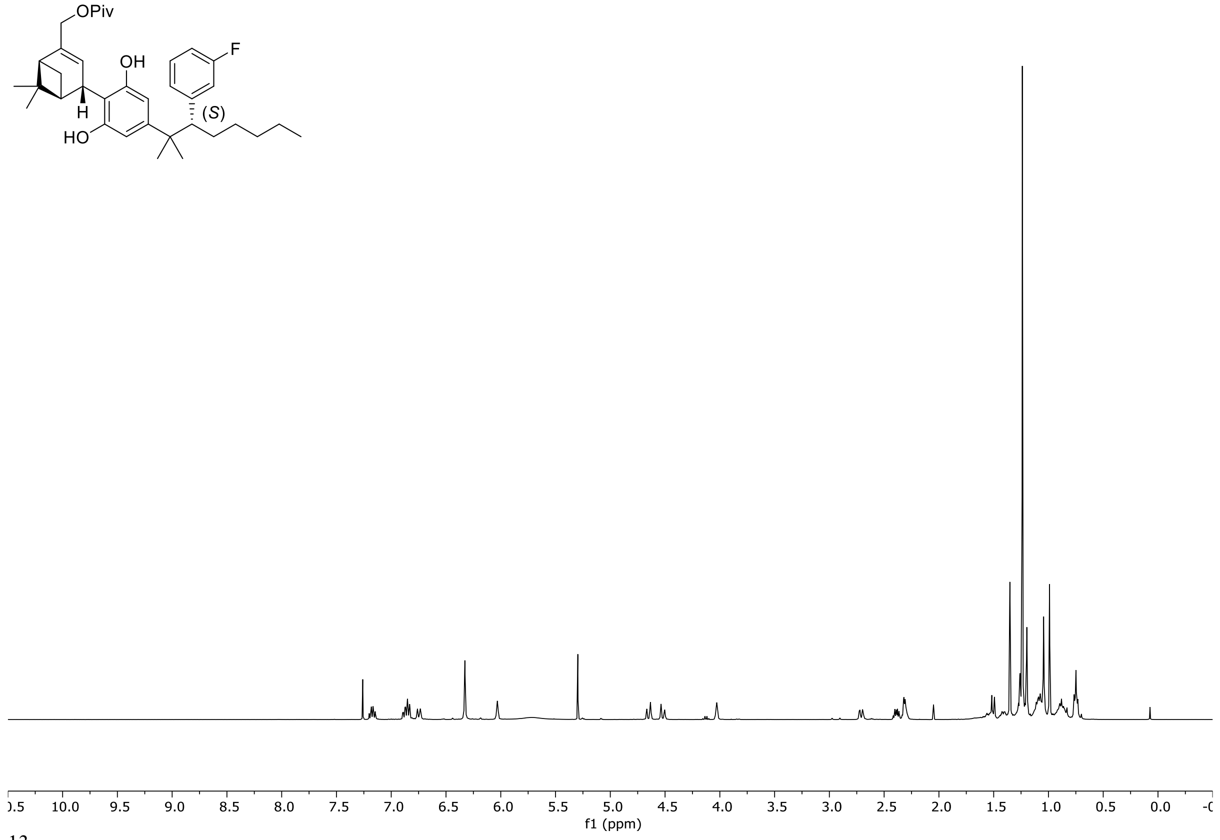

^{13}C NMR (101 MHz, $CDCl_3$) of (*S*)-**SI-20f**

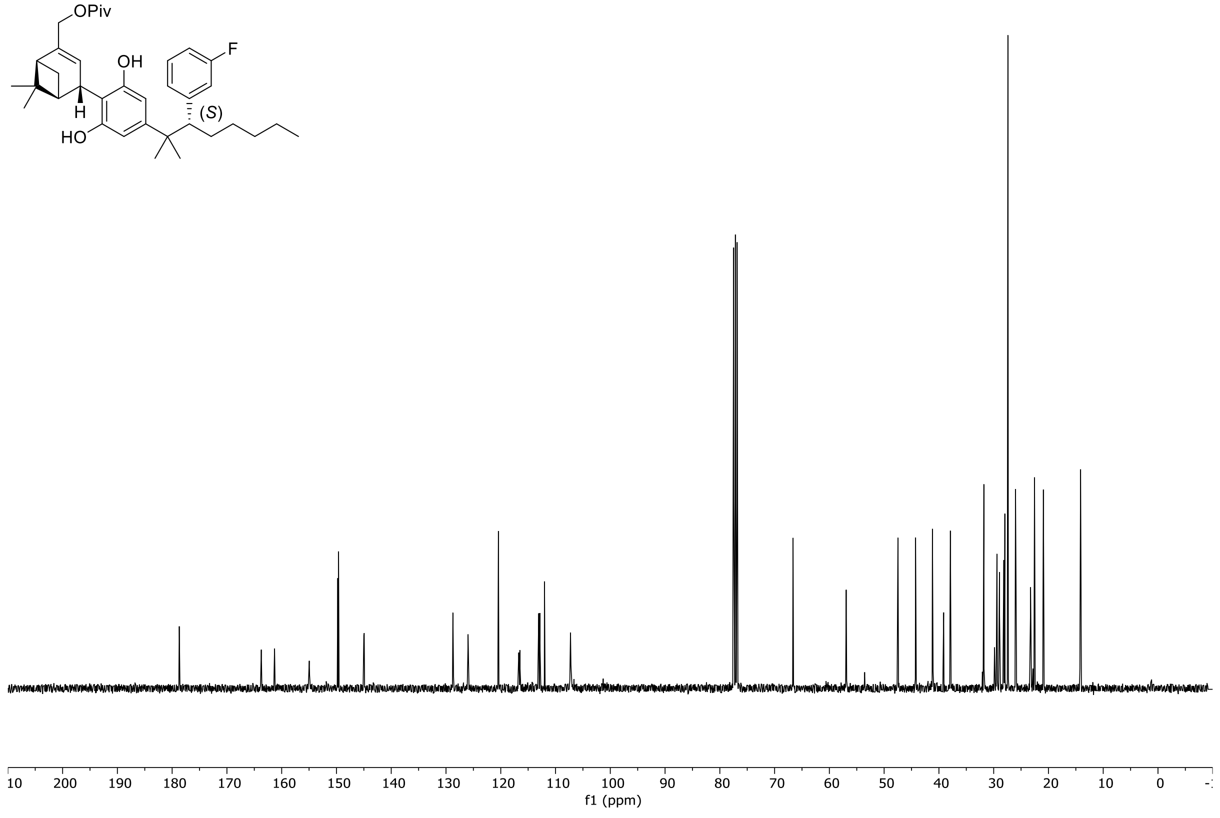

^{19}F NMR (377 MHz, $CDCl_3$) of (*S*)-**SI-20f**

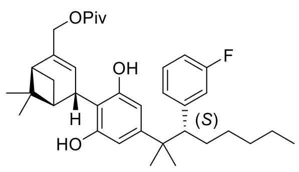

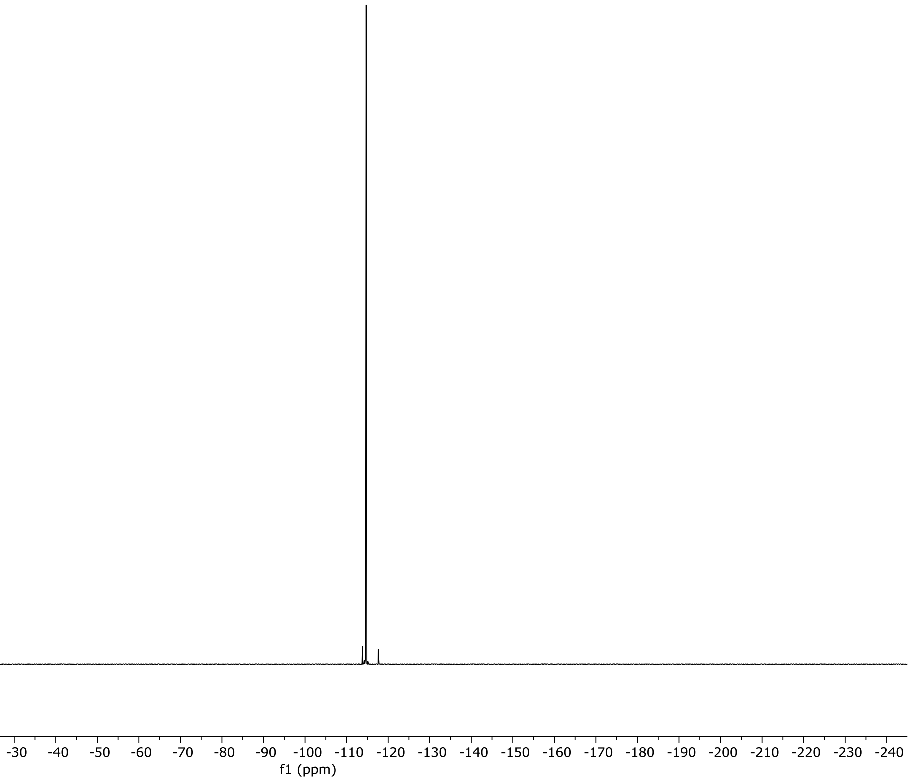

^{1}H NMR (400 MHz, $CDCl_3$) of (*R*)-**SI-20f**

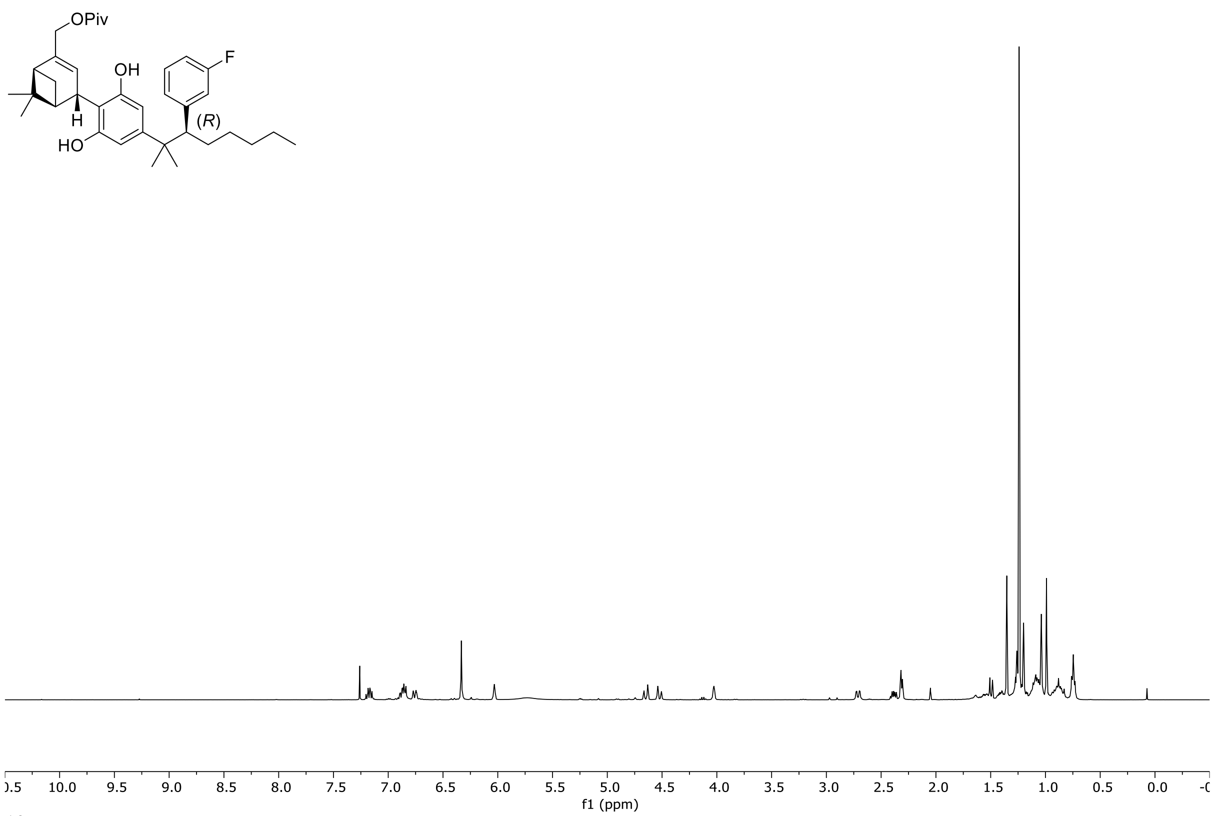

^{13}C NMR (101 MHz, $CDCl_3$) of (*R*)-**SI-20f**

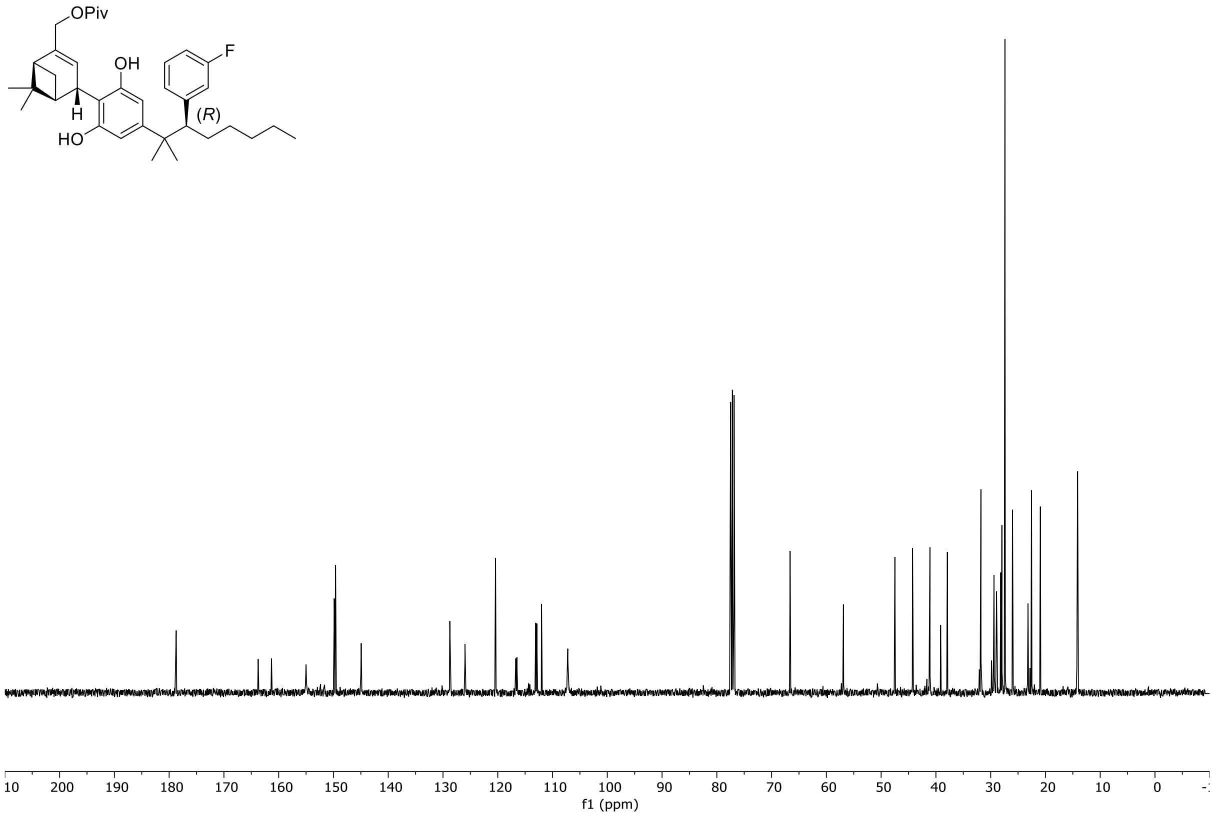

^{19}F NMR (377 MHz, $CDCl_3$) of (*R*)-**SI-20f**

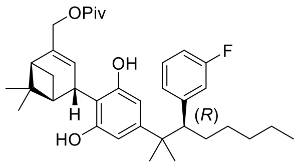

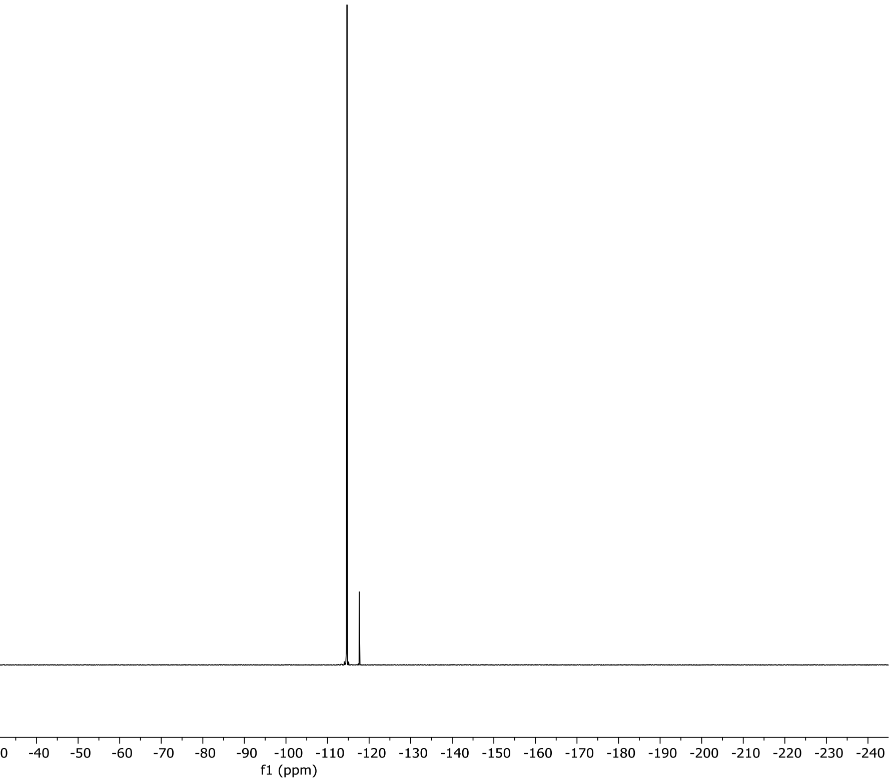

^{1}H NMR (400 MHz, $CDCl_3$) of (*S*)-**10f**

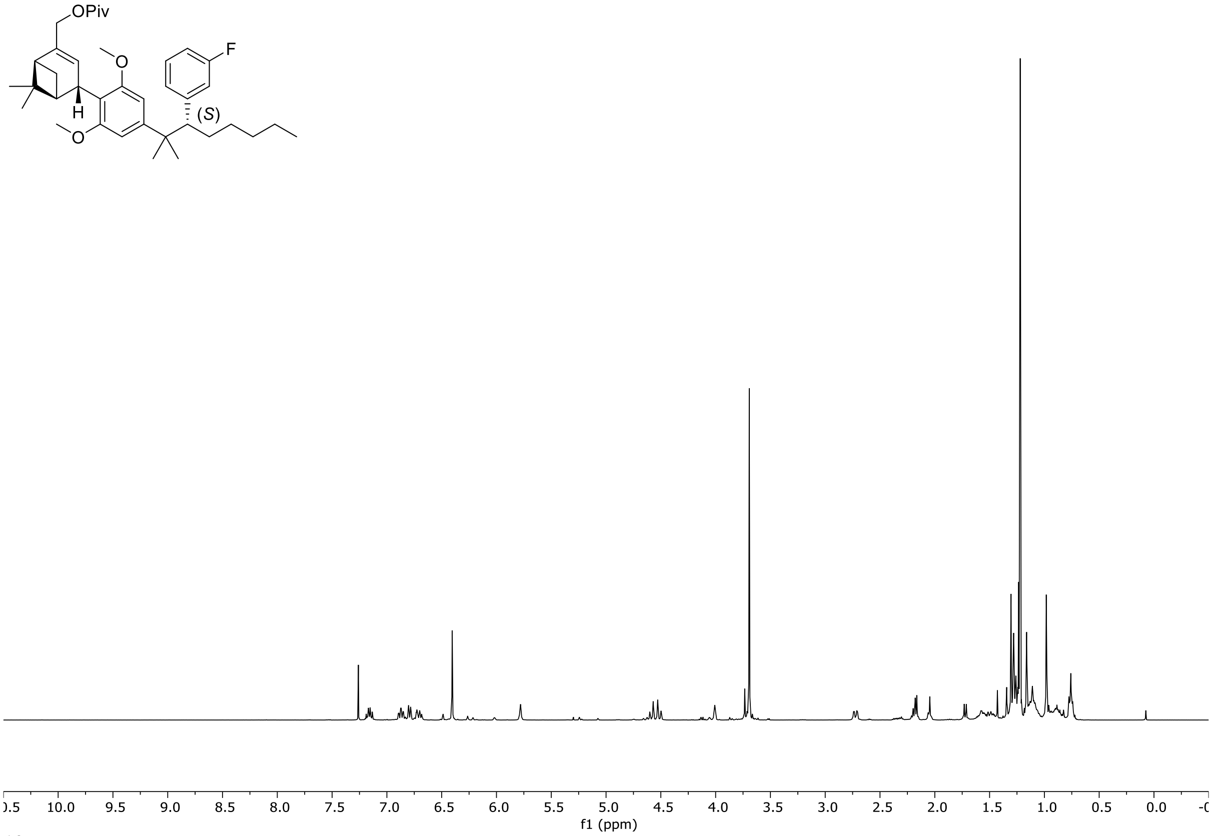

^{13}C NMR (101 MHz, $CDCl_3$) of (*S*)-**10f**

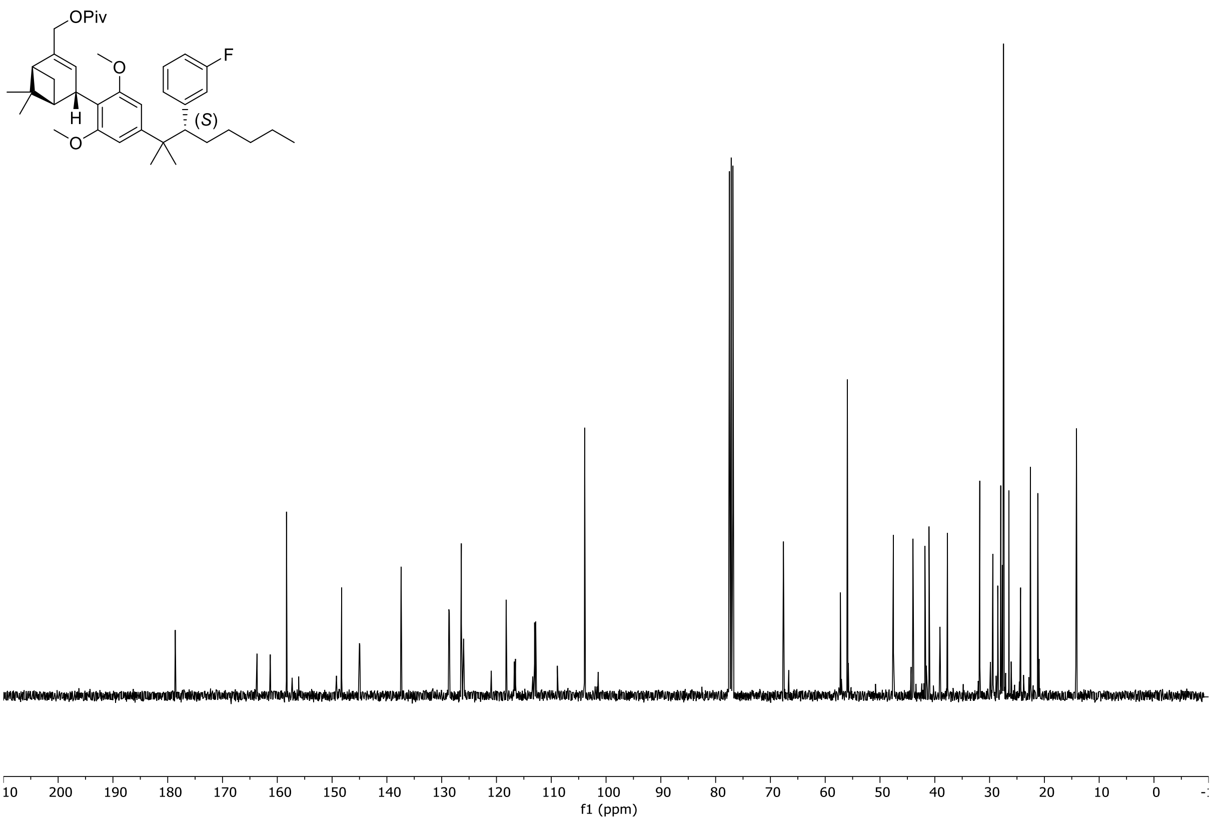

^{19}F NMR (376 MHz, $CDCl_3$) of (*S*)-**10f**

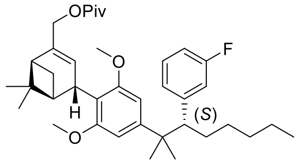

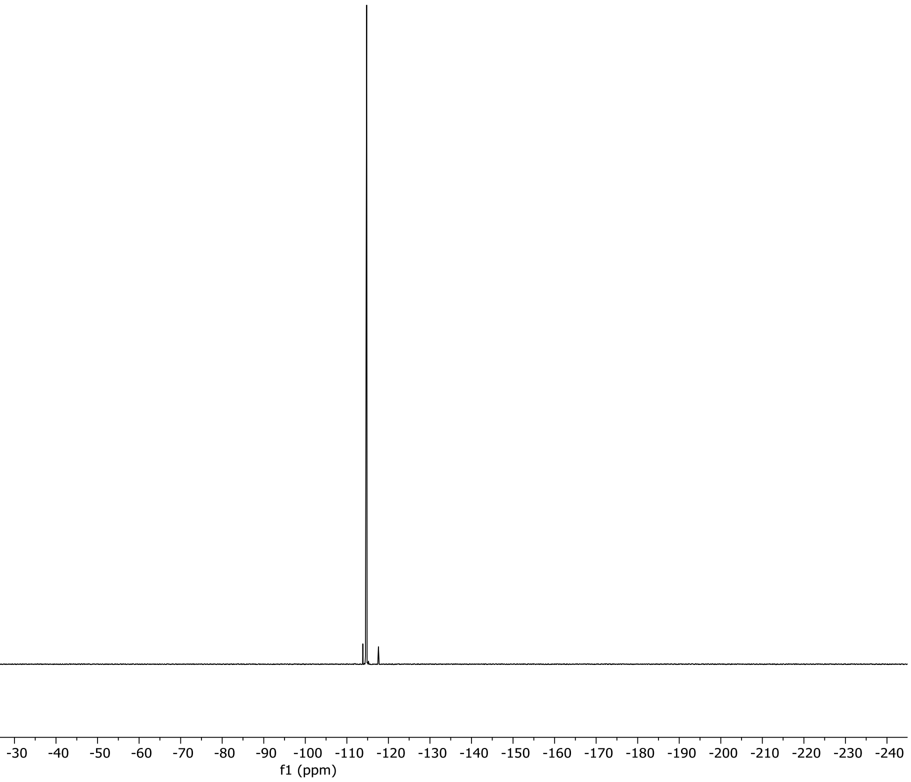

^{1}H NMR (400 MHz, $CDCl_3$) of (*R*)-**10f**

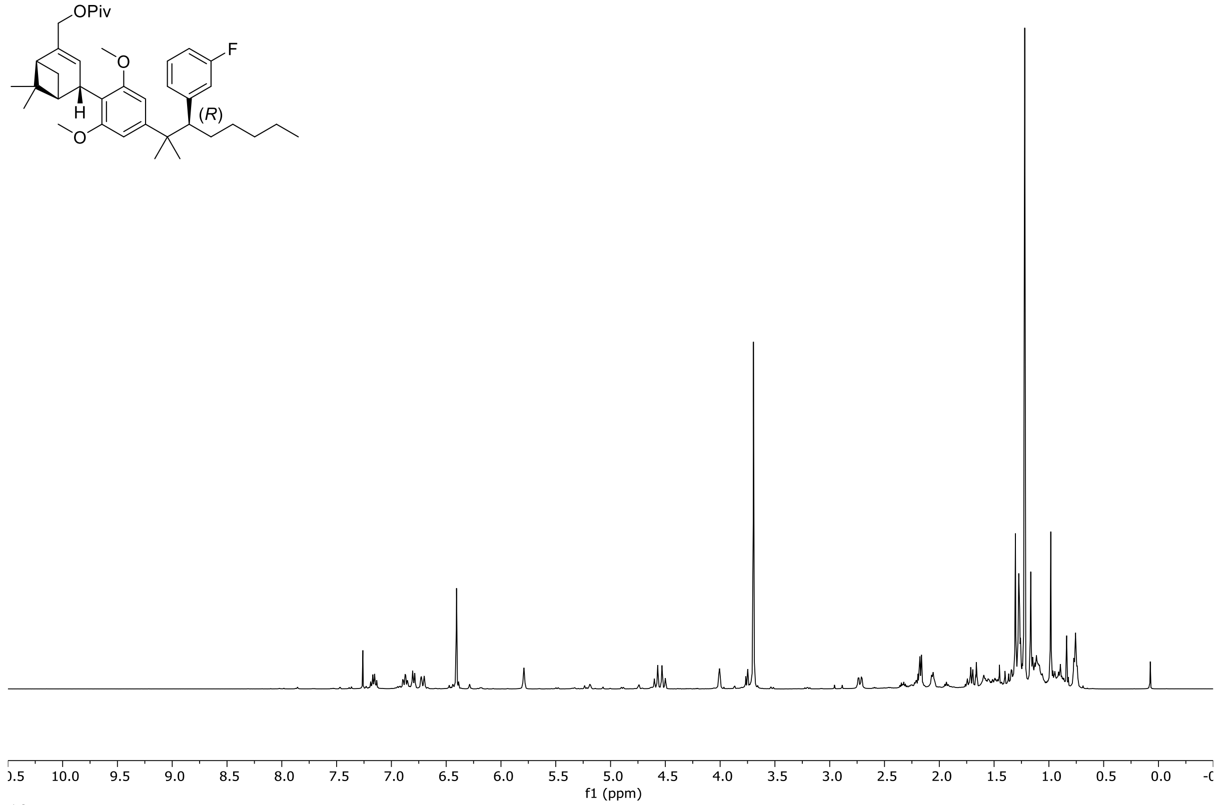

^{13}C NMR (101 MHz, $CDCl_3$) of (*R*)-**10f**

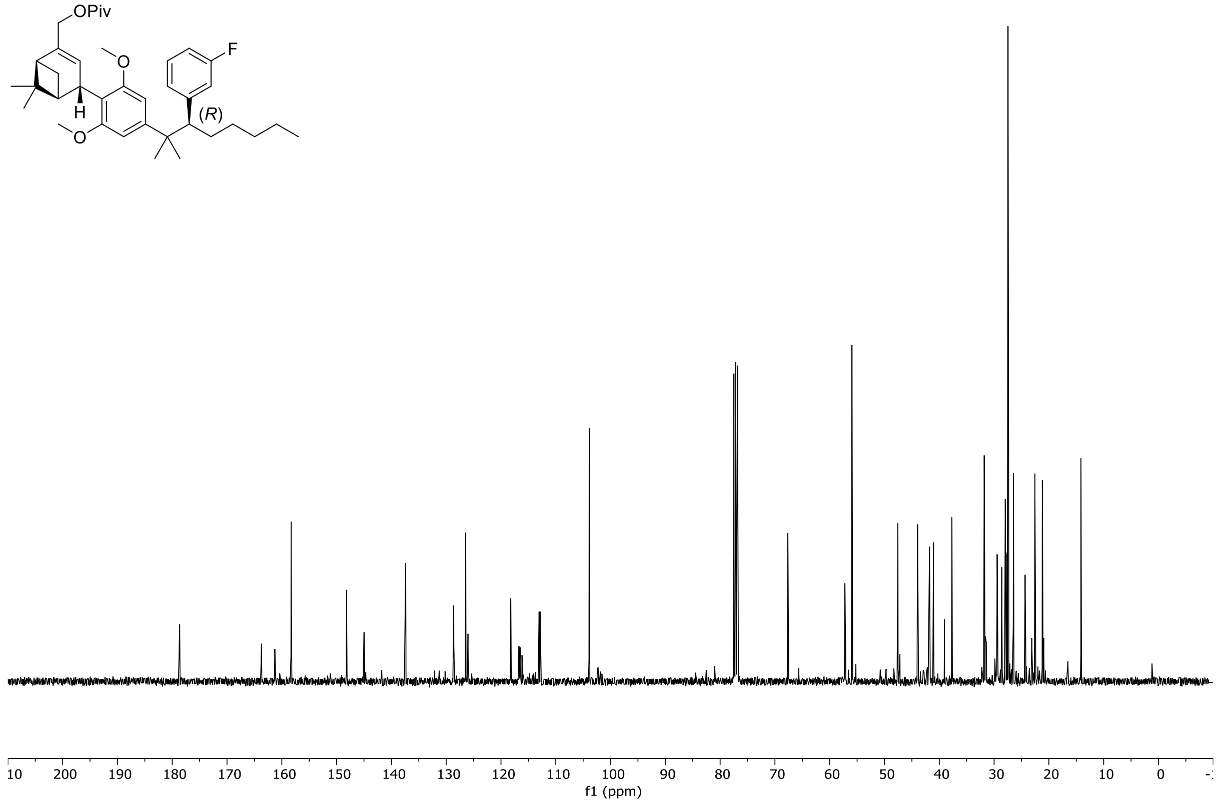

^{19}F NMR (376 MHz, $CDCl_3$) of (*R*)-**10f**

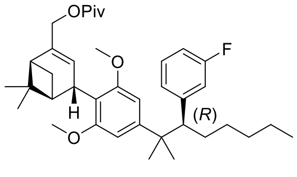

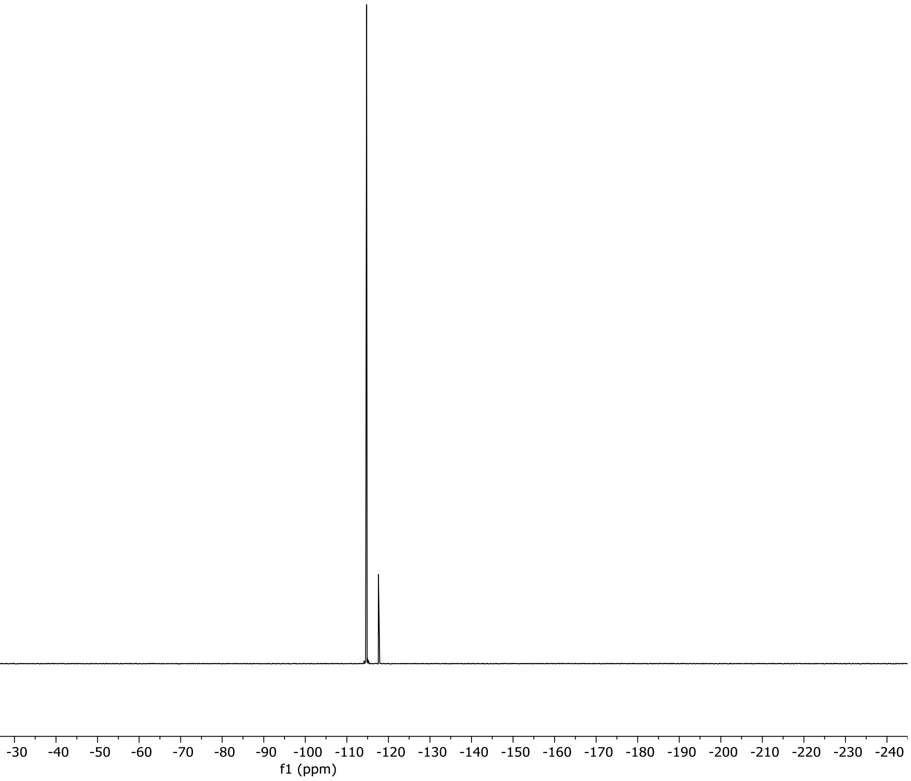

^{1}H NMR (400 MHz, $CDCl_3$) of (*S*)-**6f**

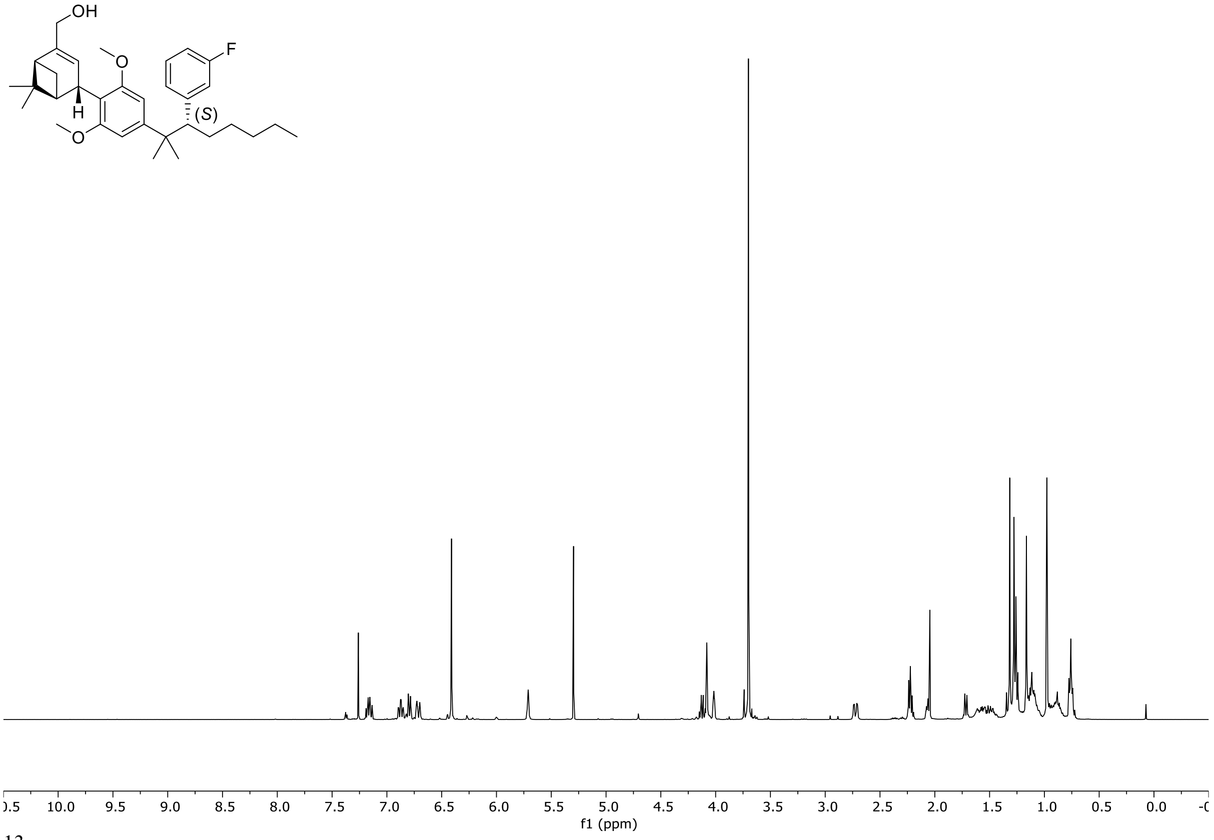

^{13}C NMR (101 MHz, $CDCl_3$) of (*S*)-**6f**

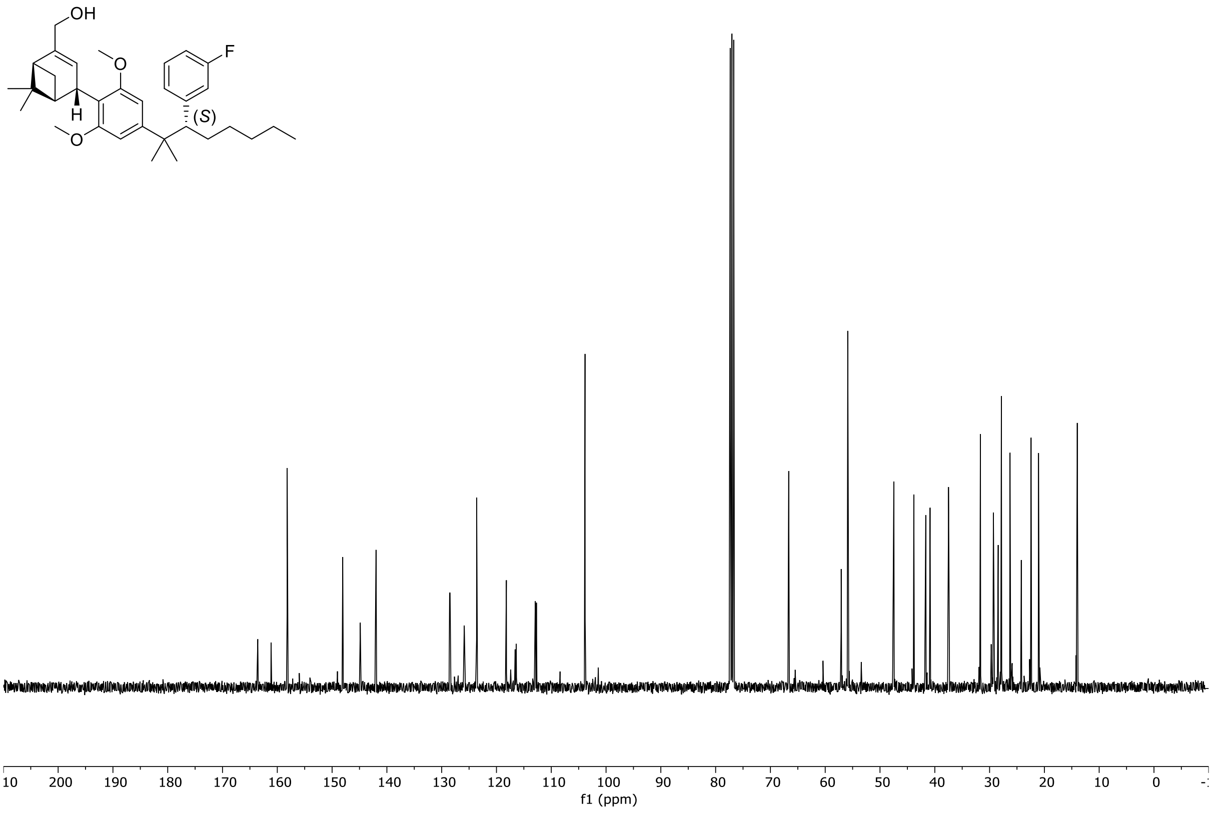

^{19}F NMR (376 MHz, $CDCl_3$) of (*S*)-**6f**

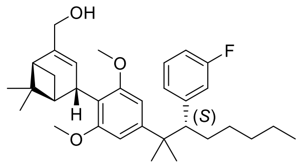

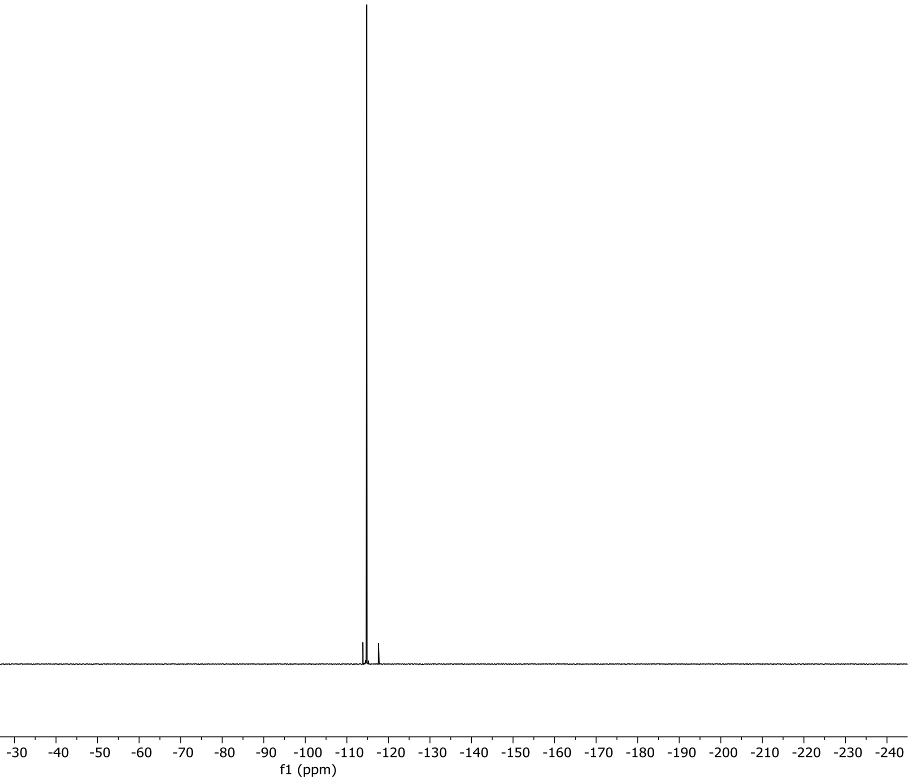

^{1}H NMR (400 MHz, $CDCl_3$) of (*R*)-**6f**

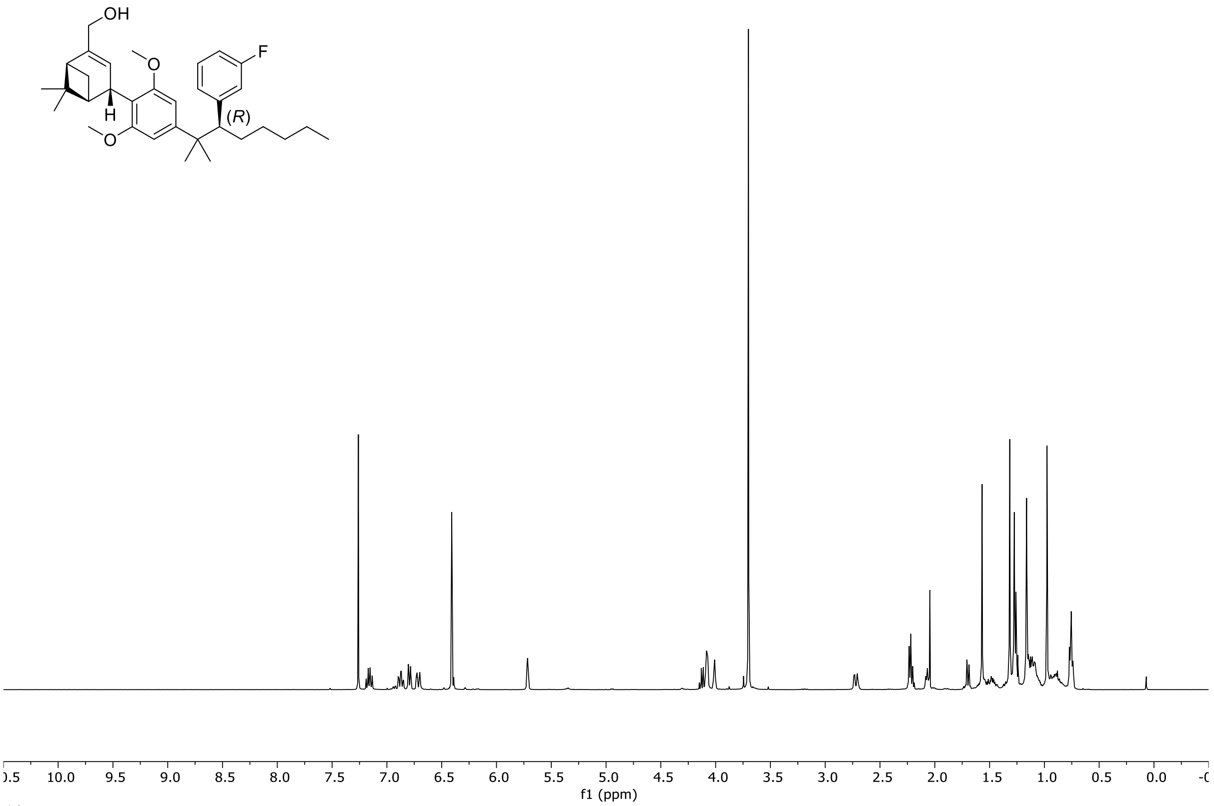

^{13}C NMR (101 MHz, $CDCl_3$) of (*R*)-**6f**

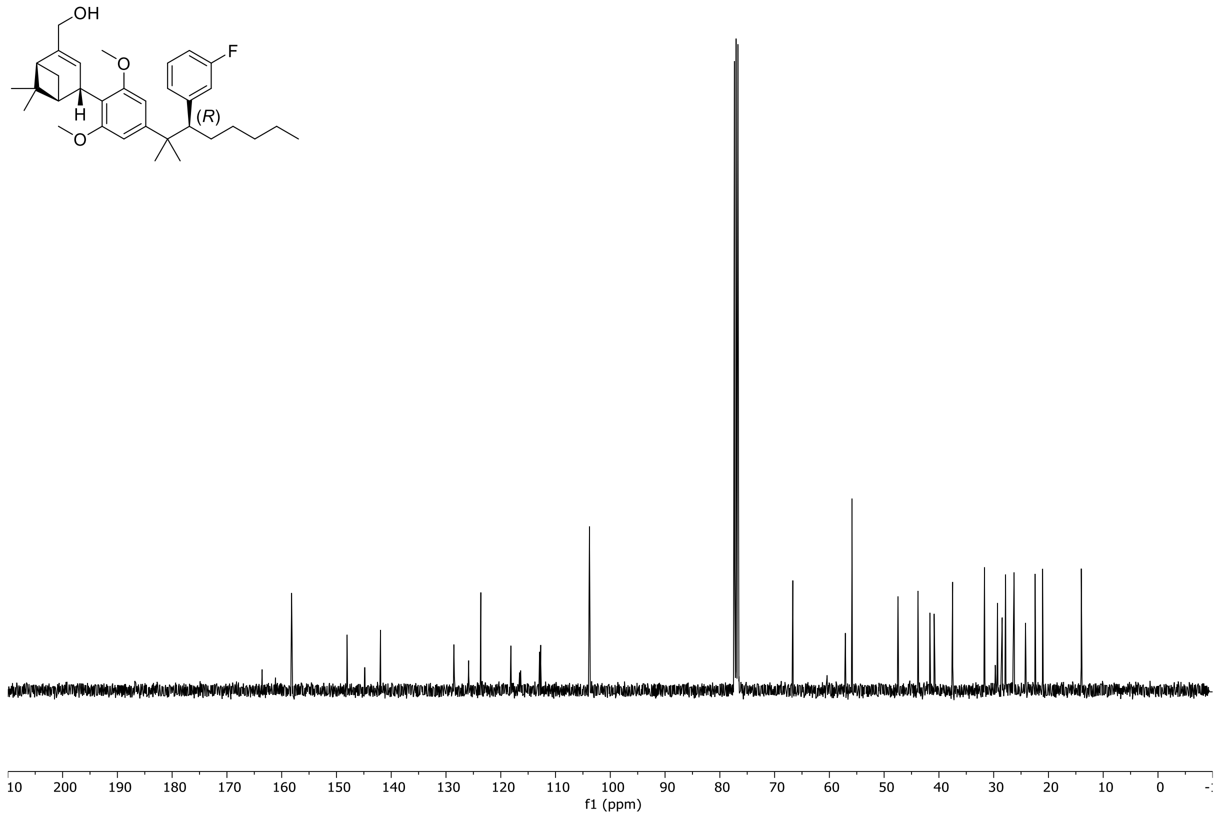

^{19}F NMR (376 MHz, $CDCl_3$) of (*R*)-**6f**

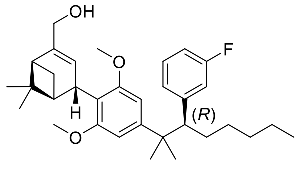

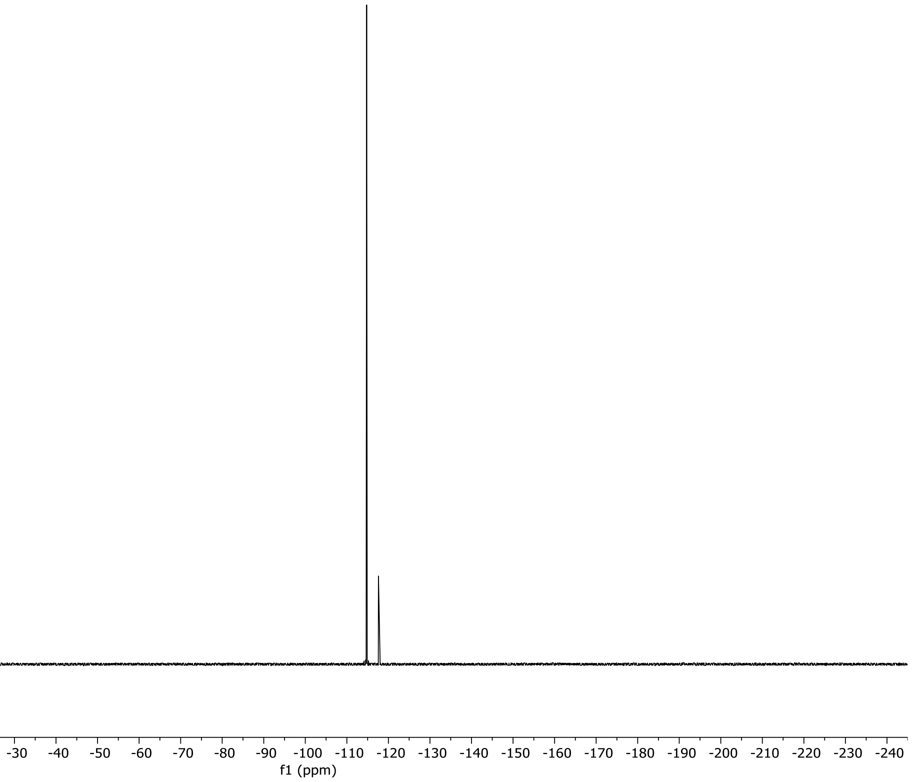

SFC Traces

SFC trace of racemate **4**

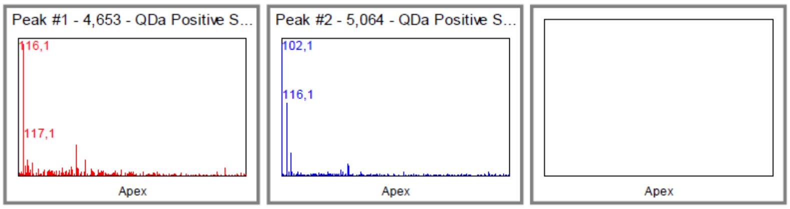

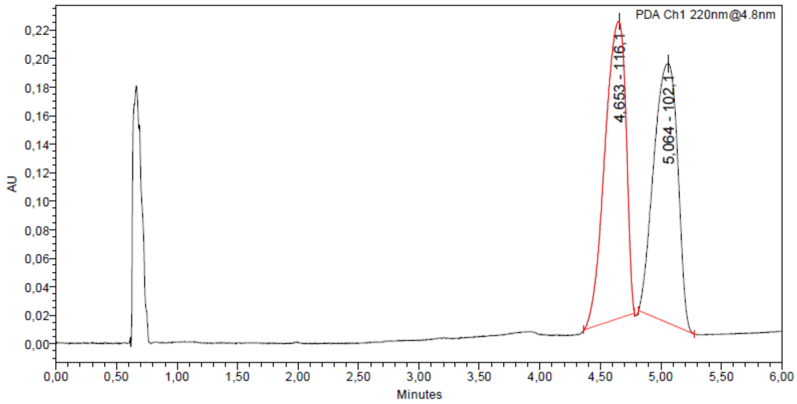

Peak Results

	RT	Area	% Area	Base Peak (m/z)
1	4,653	2517147	50,35	116,1
2	5,064	2482332	49,65	102,1

SFC trace of (+)-(*R*)-**4**

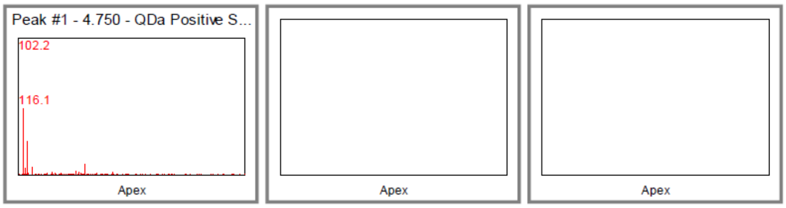

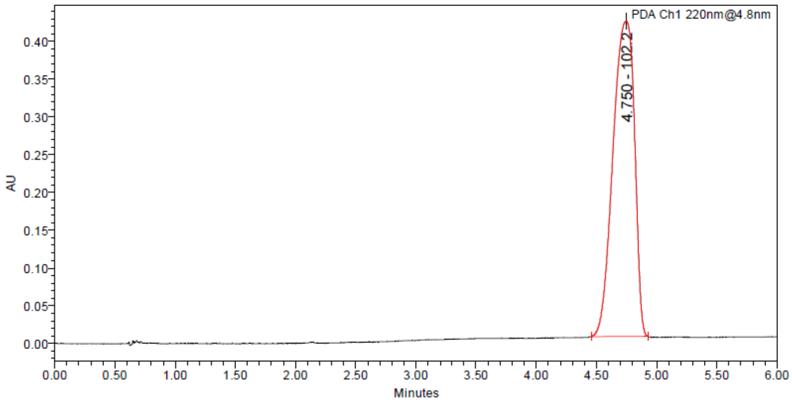

Peak Results

	RT	Area	% Area	Base Peak (m/z)
1	4.750	5180871	100.00	102.2

SFC trace of (–)-(*S*)-**4**

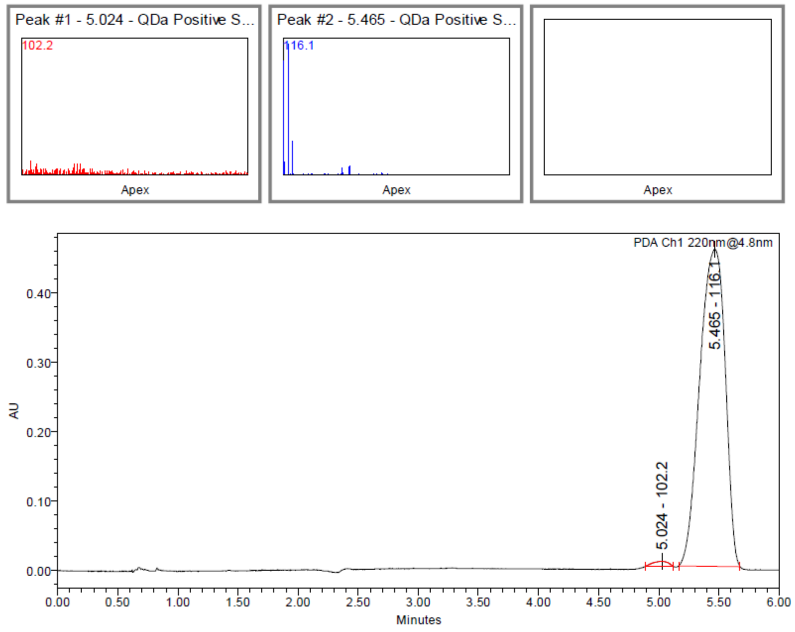

Peak Results

	RT	Area	% Area	Base Peak (m/z)
1	5.024	64599	0.96	102.2
2	5.465	6699611	99.04	116.1

SFC trace of Entry 1 from **Table S2**

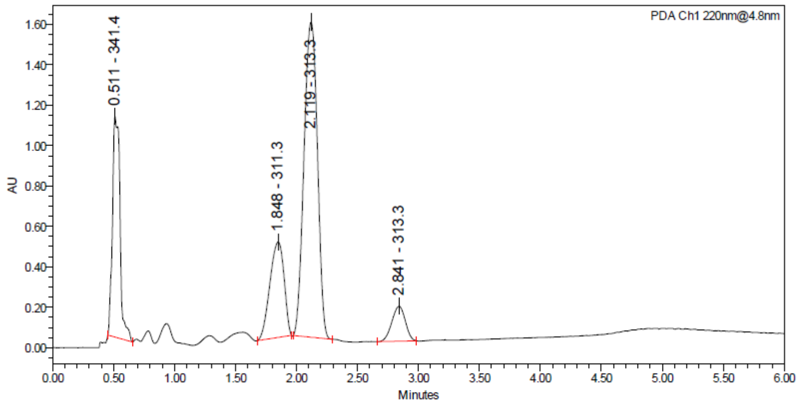

Peak Results

	RT	Area	% Area	Base Peak (m/z)
1	0.511	4728576	21.70	341.4
2	1.848	3813868	17.50	311.3
3	2.119	11925976	54.73	313.3
4	2.841	1320516	6.06	313.3

SFC trace of Entry 11 from **Table S2.**

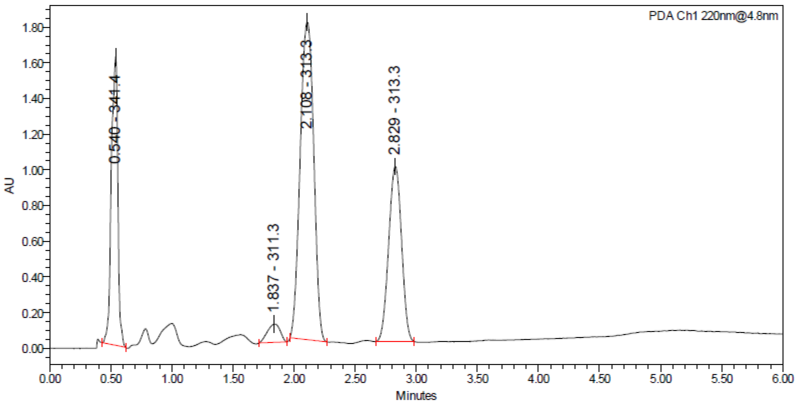

Peak Results

	RT	Area	% Area	Base Peak (m/z)
1	0.540	5946443	21.56	341.4
2	1.837	743517	2.70	311.3
3	2.108	13710163	49.70	313.3
4	2.829	7186566	26.05	313.3

SFC trace of racemate **SI-8**

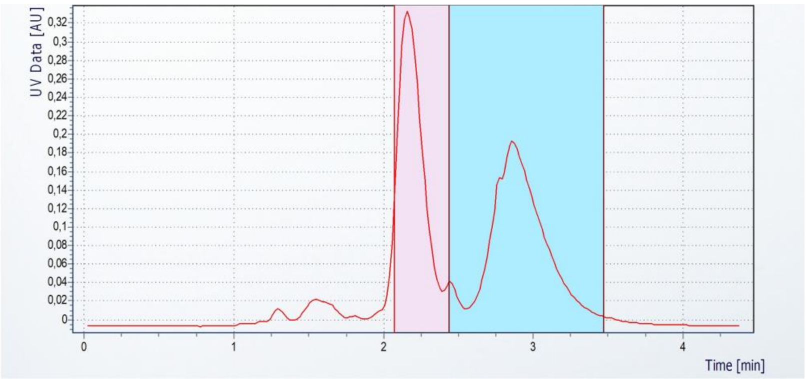

Peak Number	Peak Start	Peak Stop	Vial No.
1	2,07	2,44	1
2	2,44	3,47	2

SFC trace of (A)-**SI-8**

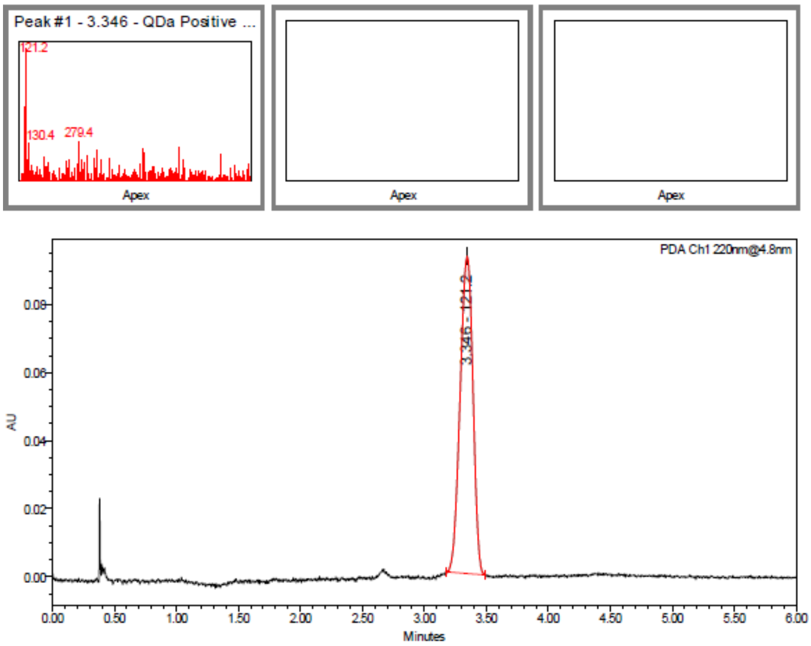

Peak Results

	RT	Area	% Area	Base Peak (m/z)
1	3.346	669130	100.00	121.2

SFC trace of (B)-**SI-8**

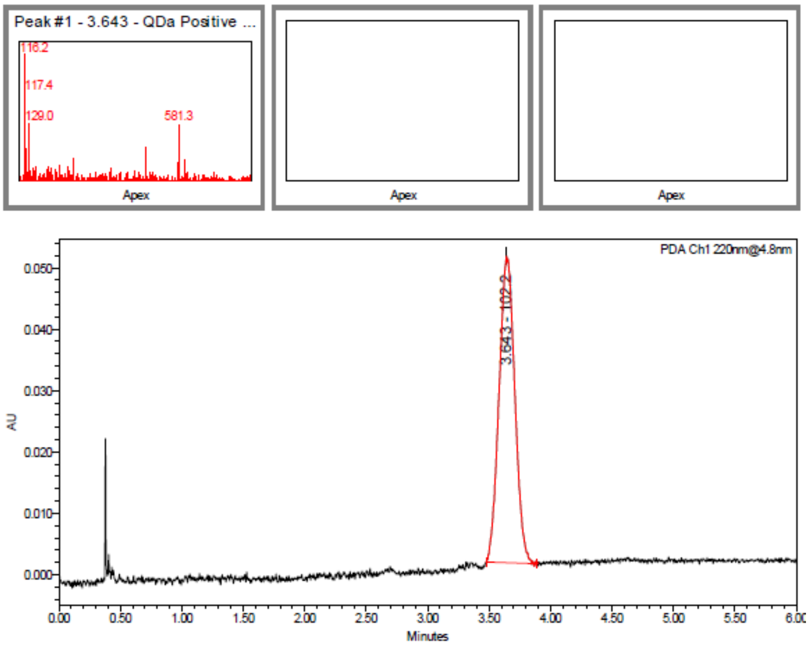

Peak Results

	RT	Area	% Area	Base Peak (m/z)
1	3.643	444337	100.00	102.2

SFC trace of racemate **9d**

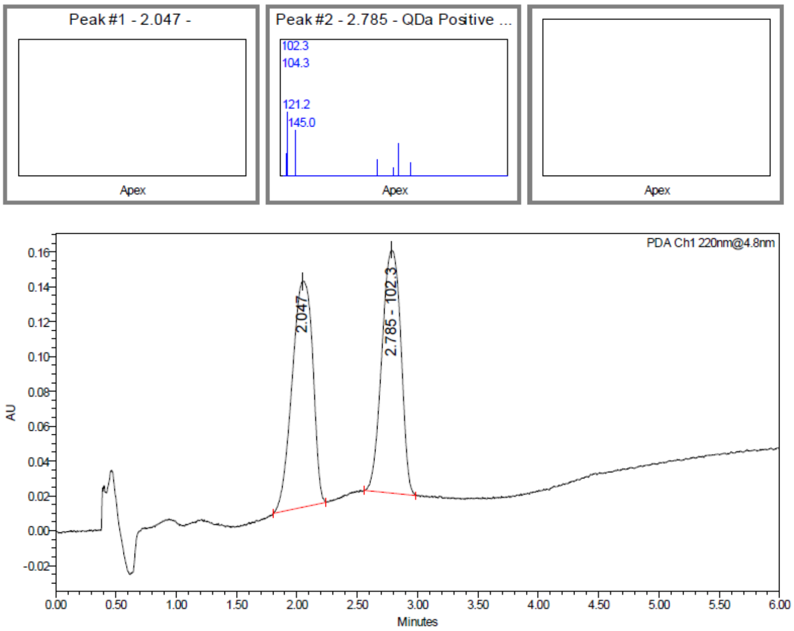

Peak Results

	RT	Area	% Area	Base Peak (m/z)
1	2.047	1548661	50.37	
2	2.785	1525983	49.63	102.3

SFC trace of (–)-(*S*)-**9d**

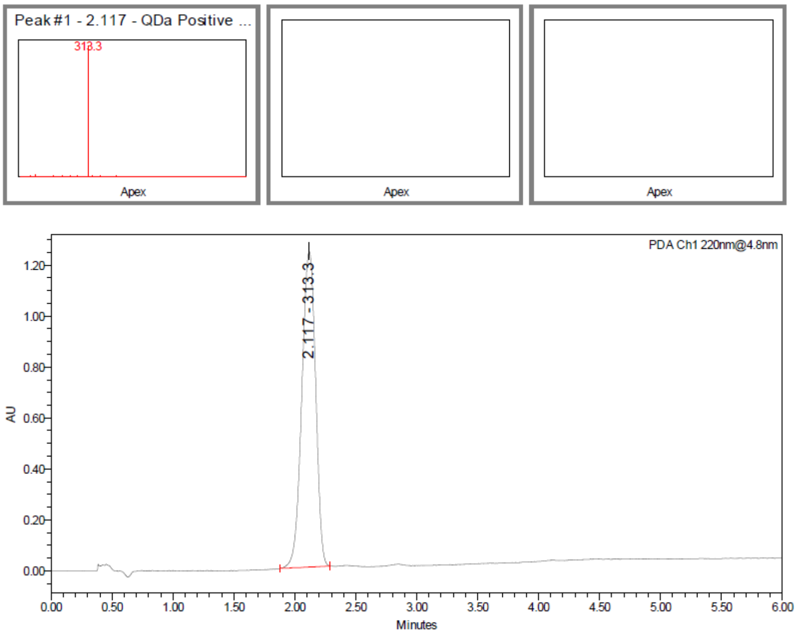

Peak Results

	RT	Area	% Area	Base Peak (m/z)
1	2.117	9741544	100.00	313.3

SFC trace of (+)-(*R*)-**9d**

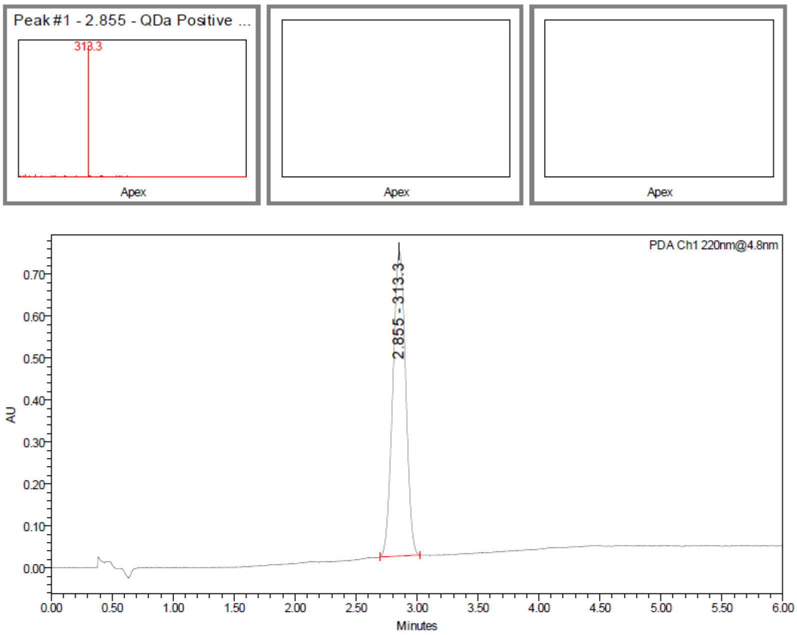

Peak Results

	RT	Area	% Area	Base Peak (m/z)
1	2.855	5305780	100.00	313.3

SFC trace of racemate **9e**

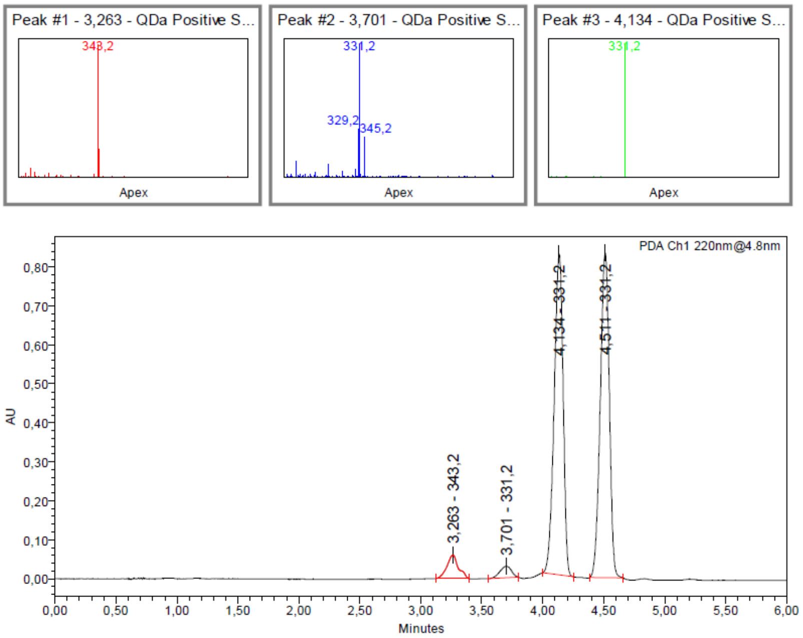

SFC trace of (–)-(*S*)-**9e**

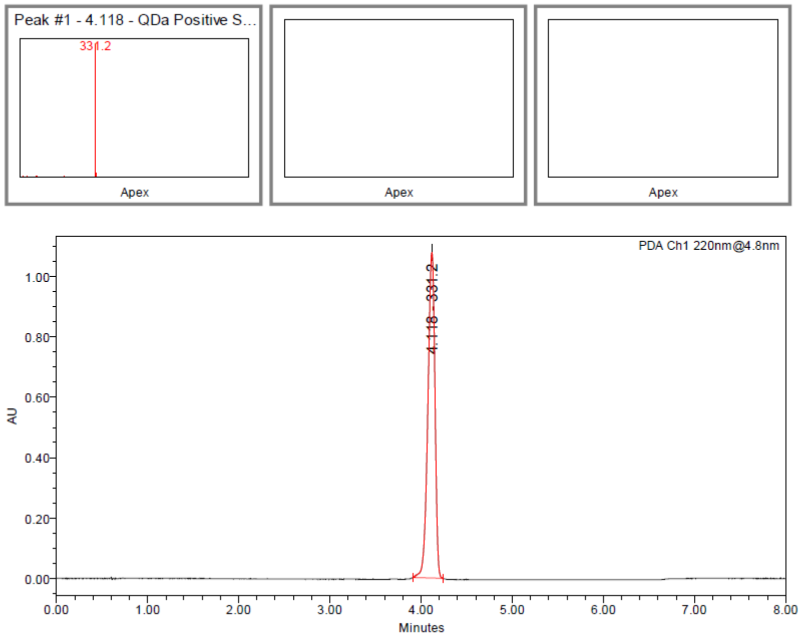

Peak Results

	RT	Area	% Area	Base Peak (m/z)
1	4.118	5751146	100.00	331.2

SFC trace of (+)-(*R*)-**9e**

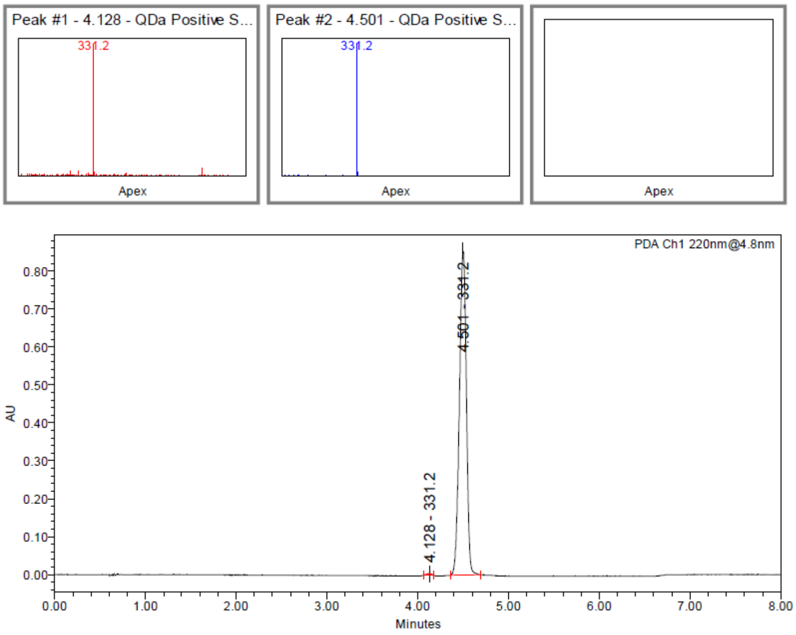

Peak Results

	RT	Area	% Area	Base Peak (m/z)
1	4.128	13746	0.30	331.2
2	4.501	4543976	99.70	331.2

SFC trace of racemate **9f**

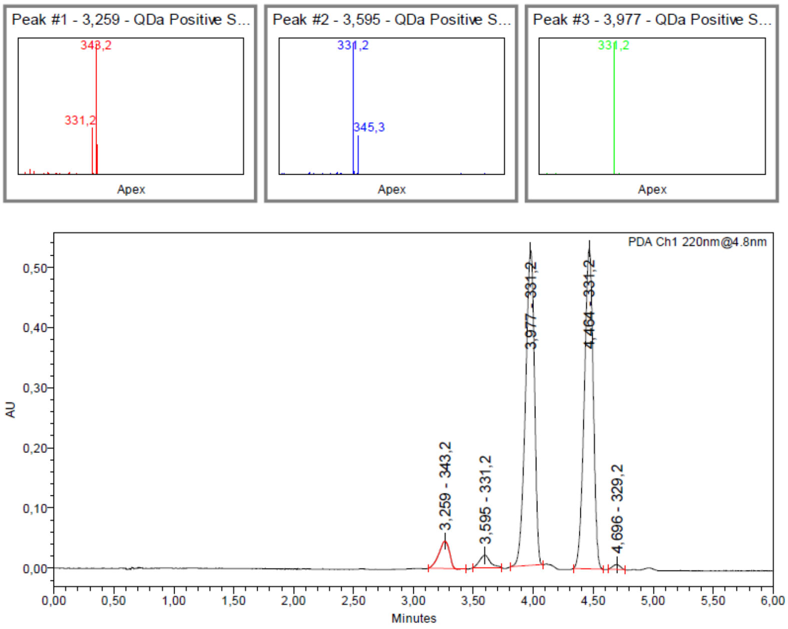

SFC trace of (–)-(*R*)-**9f**

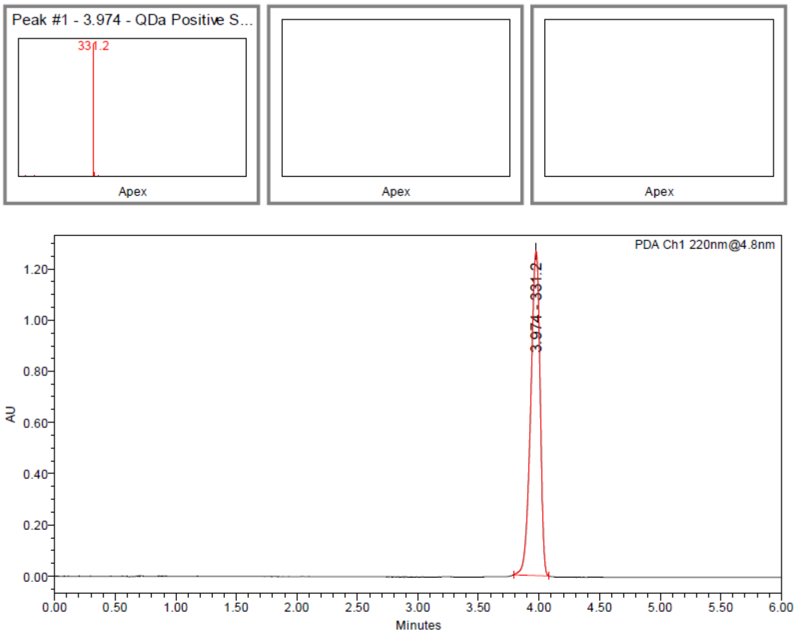

Peak Results

	RT	Area	% Area	Base Peak (m/z)
1	3.974	6754335	100.00	331.2

SFC trace of (+)-(*R*)-**9e**

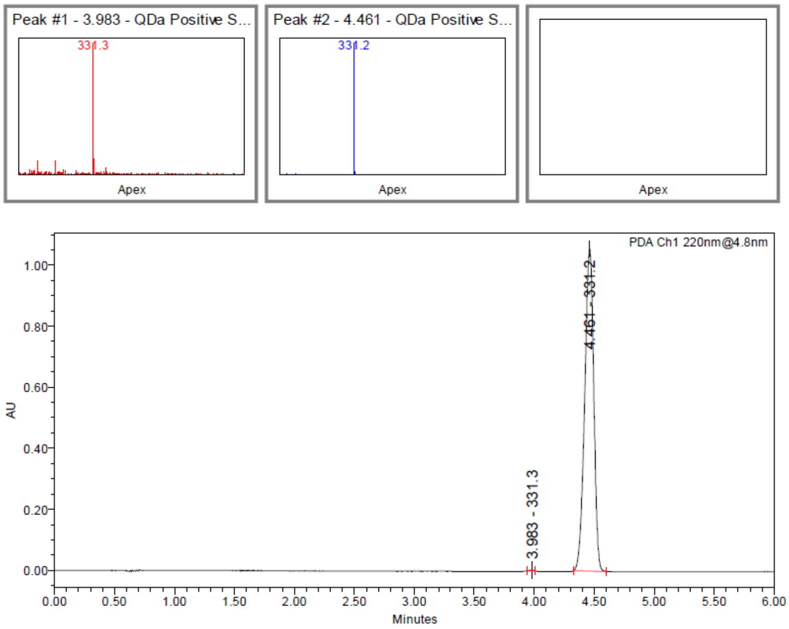

Peak Results

	RT	Area	% Area	Base Peak (m/z)
1	3.983	3237	0.06	331.3
2	4.461	5541013	99.94	331.2

SFC trace of (*S/R*)-**6c**

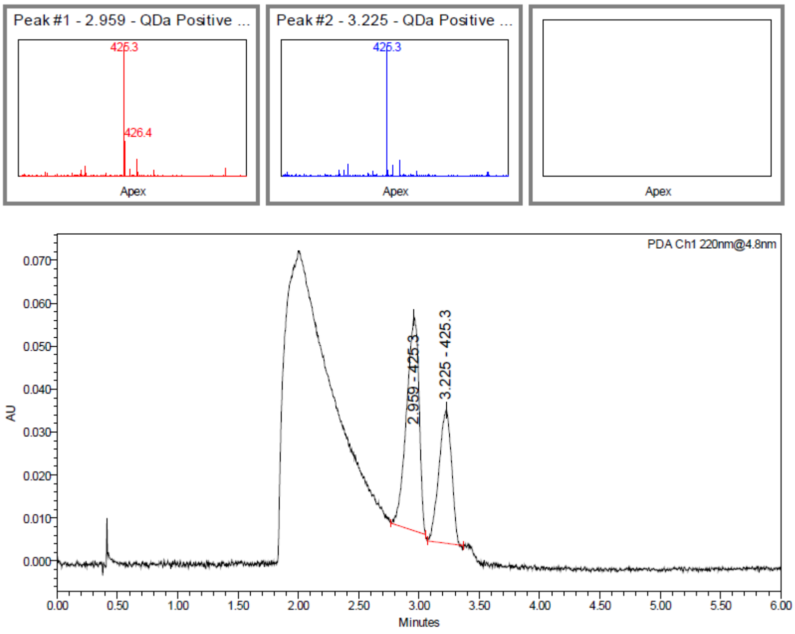

Peak Results

	RT	Area	% Area	Base Peak (m/z)
1	2.959	358897	61	425.3
2	3.225	226044	39	425.3

SFC trace of (*S*)-**6c**

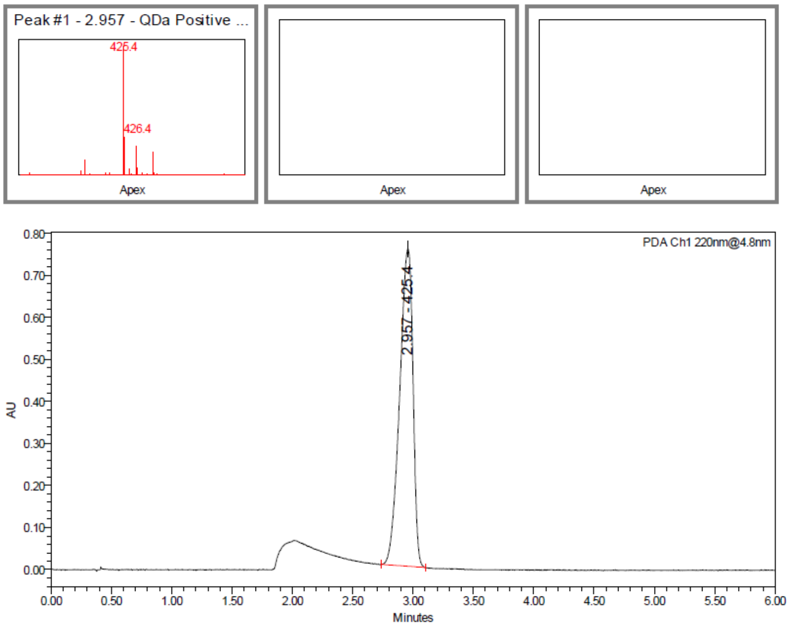

Peak Results

	RT	Area	% Area	Base Peak (m/z)
1	2.957	5777565	100	425.4

Crytallographic Data

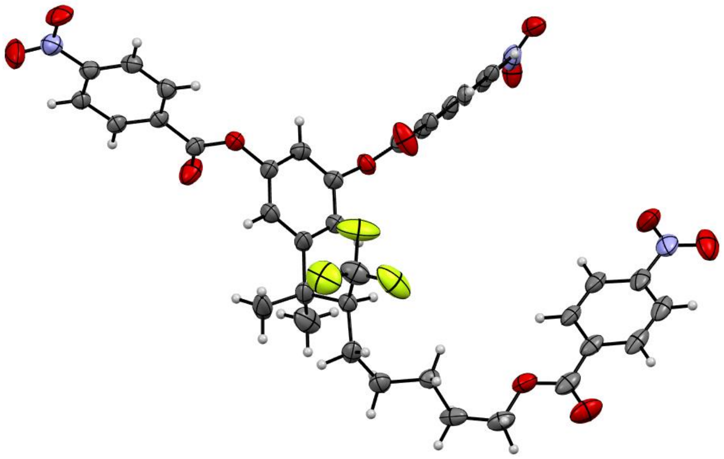

Figure S8. (S)-5-(2-methyl-8-((4-nitrobenzoyl)oxy)-3-(trifluoromethyl)octan-2-yl)-1,3-phenylene bis(4-nitrobenzoate). ORTEP diagram of (*S*)-**SI-9**. Thermal ellipsoids are displayed at 50% probability level. Hydrogen atoms are displayed as spheres with fixed radius (0.15Å)

CCDC number	2450722
Empirical formula	$C_{37}H_{32}N_3O_{12}F_3$
Formula weight	767.65
Temperature/K	100.0(1)
Crystal system	triclinic
Space group	P1
a/Å	7.01380(10)
b/Å	14.10450(10)
c/Å	19.1791(2)
α/°	109.9170(10)
β/°	99.2180(10)
γ/°	92.2350(10)
Volume/Å^3	1751.82(4)
Z	2
ρ_{calc}g/cm^3	1.455
μ/mm^{-1}	1.028
F(000)	796.0
Crystal size/mm^3	0.15 × 0.098 × 0.031
Radiation	Cu Kα (λ = 1.54184)
2Θ range for data collection/°	6.7 to 149.694
Index ranges	$-8 \le h \le 8$, $-17 \le k \le 17$, $-23 \le l \le 23$
Reflections collected	13737
Independent reflections	13737 [R_{int} = 0.0214, R_{sigma} = 0.0123]
Data/restraints/parameters	13737/4082/1540
Goodness-of-fit on F^2	1.019
Final R indexes [I>=2σ (I)]	R_1 = 0.0428, wR_2 = 0.1171
Final R indexes [all data]	R_1 = 0.0462, wR_2 = 0.1206
Largest diff. peak/hole / e Å^{-3}	0.59/-0.24
Flack parameter	0.03(5)

References

1. L. Borrega-Roman, B. L. Hoare, M. Kosar, R. C. Sarott, K. J. Patej, J. Bouma, M. Scott-Dennis, E. J. Koers, T. Gazzi, L. Mach, S. Barrondo, J. Salles, W. Guba, E. Kusznir, M. Nazare, A. C. Rufer, U. Grether, L. H. Heitman, E. M. Carreira, D. A. Sykes and D. B. Veprintsev, A universal cannabinoid CB1 and CB2 receptor TR-FRET kinetic ligand-binding assay, *Front. Pharmacol.*, 2025, **16**, 1469986.
2. M. Kosar, R. C. Sarott, D. A. Sykes, A. E. G. Viray, R. M. Vitale, N. Tomasevic, X. Li, R. L. Z. Ganzoni, B. Kicin, L. Reichert, K. J. Patej, U. Gomez-Bouzo, W. Guba, P. J. McCormick, T. Hua, C. W. Gruber, D. B. Veprintsev, J. A. Frank, U. Grether and E. M. Carreira, Flipping the GPCR Switch: Structure-Based Development of Selective Cannabinoid Receptor 2 Inverse Agonists, *ACS Cent. Sci.*, 2024, **10**, 956-968.
3. M. Scott-Dennis, F. A. Rafani, Y. Yi, T. Perera, C. R. Harwood, W. Guba, A. C. Rufer, U. Grether, D. B. Veprintsev and D. A. Sykes, Development of a membrane-based Gi-CASE biosensor assay for profiling compounds at cannabinoid receptors, *Front. Pharmacol.*, 2023, **14**, 1158091.
4. Optimal for sensitivity of the assay
5. Y. Cheng and W. H. Prusoff, Relationship between the inhibition constant (K1) and the concentration of inhibitor which causes 50 per cent inhibition (I50) of an enzymatic reaction, *Biochem. Pharmacol.*, 1973, **22**, 3099-3108.
6. F. J. Ehlert, M. T. Griffin, G. W. Sawyer and R. Bailon, A simple method for estimation of agonist activity at receptor subtypes: comparison of native and cloned M3 muscarinic receptors in guinea pig ileum and transfected cells, *J. Pharmacol. Exp. Ther.*, 1999, **289**, 981-992.
7. D. Winpenny, M. Clark and D. Cawkill, Biased ligand quantification in drug discovery: from theory to high throughput screening to identify new biased mu opioid receptor agonists, *Br J Pharmacol*, 2016, **173**, 1393-1403.
8. X. Li, H. Chang, J. Bouma, L. V. de Paus, P. Mukhopadhyay, J. Paloczi, M. Mustafa, C. van der Horst, S. S. Kumar, L. Wu, Y. Yu, R. van den Berg, A. P. A. Janssen, A. Lichtman, Z. J. Liu, P. Pacher, M. van der Stelt, L. H. Heitman and T. Hua, Structural basis of selective cannabinoid CB(2) receptor activation, *Nat. Commun.*, 2023, **14**, 1447-1463.
9. X. Li, T. Hua, K. Vemuri, J. H. Ho, Y. Wu, L. Wu, P. Popov, O. Benchama, N. Zvonok, K. Locke, L. Qu, G. W. Han, M. R. Iyer, R. Cinar, N. J. Coffey, J. Wang, M. Wu, V. Katritch, S. Zhao, G. Kunos, L. M. Bohn, A. Makriyannis, R. C. Stevens and Z. J. Liu, Crystal Structure of the Human Cannabinoid Receptor CB2, *Cell*, 2019, **176**, 459-467.
10. ULC, C. C. G. Molecular Operating Environment (MOE), 2022.02, 1010 Sherbrooke St. West, Suite 910, Montreal, QC, Canada, 2022
11. G. Jones, P. Willett, R. C. Glen, A. R. Leach and R. Taylor, Development and validation of a genetic algorithm for flexible docking, *J. Mol. Biol.*, 1997, **267**, 727-748.
12. E. F. Pettersen, T. D. Goddard, C. C. Huang, G. S. Couch, D. M. Greenblatt, E. C. Meng and T. E. Ferrin, UCSF Chimera--a visualization system for exploratory research and analysis, *J. Comput. Chem.*, 2004, **25**, 1605-1612.
13. M. W. Schmidt, K. K. Baldridge, J. A. Boatz, S. T. Elbert, M. S. Gordon, J. H. Jensen, S. Koseki, N. Matsunaga, K. A. Nguyen, S. Su, T. L. Windus, M. Dupuis and J. A. Montgomery Jr, General atomic and molecular electronic structure system, *J. Comput. Chem.*, 1993, **14**, 1347-1363.
14. G. M. Morris, R. Huey, W. Lindstrom, M. F. Sanner, R. K. Belew, D. S. Goodsell and A. J. Olson, AutoDock4 and AutoDockTools4: Automated docking with selective receptor flexibility, *J. Comput. Chem.*, 2009, **30**, 2785-2791.

15. T. Fox and P. A. Kollman, Application of the RESP Methodology in the Parametrization of Organic Solvents, *J. Phys. Chem. B*, 1998, **102**, 8070-8079.
16. D.A. Case, K. Belfon, I.Y. Ben-Shalom, S.R. Brozell, D.S. Cerutti, T.E. Cheatham, III, V.W.D. Cruzeiro,T.A. Darden, R.E. Duke, G. Giambasu, M.K. Gilson, H. Gohlke, A.W. Goetz,R Harris, S. Izadi, S.A. Izmailov, K. Kasavajhala, A. Kovalenko, R. Krasny, T. Kurtzman, T.S. Lee, S. LeGrand, P. Li, C. Lin, J. Liu, T. Luchko, R. Luo, V. Man, K.M. Merz, Y. Miao, O. Mikhailovskii, G. Monard, H. Nguyen, A. Onufriev, F. Pan, S. Pantano, R. Qi, D.R. Roe, A. Roitberg, C. Sagui, S. Schott-Verdugo, J. Shen, C.L. Simmerling, N.R. Skrynnikov, J. Smith, J. Swails, R.C. Walker, J. Wang, L. Wilson, R.M. Wolf, X. Wu, Y. Xiong, Y. Xue, D.M. York and P.A. Kollman (2020), AMBER 2020, University of California, San Francisco.
17. B. R. Miller, 3rd, T. D. McGee, Jr., J. M. Swails, N. Homeyer, H. Gohlke and A. E. Roitberg, MMPBSA.py: An Efficient Program for End-State Free Energy Calculations, *J Chem Theory Comput*, 2012, **8**, 3314-3321.
18. J. Mongan, C. Simmerling, J. A. McCammon, D. A. Case and A. Onufriev, Generalized Born model with a simple, robust molecular volume correction, *J Chem Theory Comput*, 2007, **3**, 156-169.
19. J. Weiser, P. S. Shenkin and W. C. Still, Approximate atomic surfaces from linear combinations of pairwise overlaps (LCPO), *J. Comput. Chem.*, 1999, **20**, 217-230.
20. CrysAlisPro and ABSPACK. Rigaku Oxford Diffraction, 2016.
21. G. M. Sheldrick, SHELXT - integrated space-group and crystal-structure determination, *Acta Crystallogr A Found Adv*, 2015, **71**, 3-8.
22. G. M. Sheldrick, A short history of SHELX, *Acta Crystallogr A*, 2008, **64**, 112-122.
23. G. M. Sheldrick, Crystal structure refinement with SHELXL, *Acta Crystallogr C Struct Chem*, 2015, **71**, 3-8.
24. O. V. Dolomanov, L. J. Bourhis, R. J. Gildea, J. A. K. Howard and H. Puschmann, OLEX2: a complete structure solution, refinement and analysis program, *J. Appl. Crystallogr.*, 2009, **42**, 339-341.
25. S. P. Nikas, R. Sharma, C. A. Paronis, S. Kulkarni, G. A. Thakur, D. Hurst, J. T. Wood, R. S. Gifford, G. Rajarshi, Y. Liu, J. G. Raghav, J. J. Guo, T. U. Jarbe, P. H. Reggio, J. Bergman and A. Makriyannis, Probing the carboxyester side chain in controlled deactivation (-)-delta(8)-tetrahydrocannabinols, *J. Med. Chem.*, 2015, **58**, 665-681.
26. C. Nilewski, N. R. Deprez, T. C. Fessard, D. B. Li, R. W. Geisser and E. M. Carreira, Synthesis of undecachlorosulfolipid A: re-evaluation of the nominal structure, *Angew Chem Int Ed Engl*, 2011, **50**, 7940-7943.
27. M. Soethoudt, S. C. Stolze, M. V. Westphal, L. van Stralen, A. Martella, E. J. van Rooden, W. Guba, Z. V. Varga, H. Deng, S. I. van Kasteren, U. Grether, I. J. AP, P. Pacher, E. M. Carreira, H. S. Overkleeft, A. Ioan-Facsinay, L. H. Heitman and M. van der Stelt, Selective Photoaffinity Probe That Enables Assessment of Cannabinoid CB2 Receptor Expression and Ligand Engagement in Human Cells, *J. Am. Chem. Soc.*, 2018, **140**, 6067-6075.
28. T. L. Church and P. G. Andersson, Iridium catalysts for the asymmetric hydrogenation of olefins with nontraditional functional substituents, *Coord. Chem. Rev.*, 2008, **252**, 513-531.
